# Charting the Chemical Reaction Space around a Multicomponent Combination: Controlled Access to a Diverse Set of Biologically Relevant Scaffolds

**DOI:** 10.1002/ange.202303889

**Published:** 2023-06-02

**Authors:** Pau Nadal Rodríguez, Ouldouz Ghashghaei, Anna M. Schoepf, Sam Benson, Marc Vendrell, Rodolfo Lavilla

**Affiliations:** ^1^ Department of Medicinal Chemistry Faculty of Pharmacy and Food Sciences University of Barcelona and Institute of Biomedicine UB (IBUB) Av. De Joan XXIII, 27–31 08028 Barcelona Spain; ^2^ Centre for Inflammation Research The University of Edinburgh Edinburgh UK

**Keywords:** Green Fluorescent Protein Chromophore, Heterocycles, Isocyanides, Multicomponent Reactions, Reaction Discovery

## Abstract

Charting the chemical reaction space around the combination of carbonyls, amines, and isocyanoacetates allows the description of new multicomponent processes leading to a variety of unsaturated imidazolone scaffolds. The resulting compounds display the chromophore of the green fluorescent protein and the core of the natural product coelenterazine. Despite the competitive nature of the pathways involved, general protocols provide selective access to the desired chemotypes. Moreover, we describe unprecedented reactivity at the C‐2 position of the imidazolone core to directly afford C, S, and N‐derivatives featuring natural products (e.g. leucettamines), potent kinase inhibitors, and fluorescent probes with suitable optical and biological profiles.

## Graphical abstract

Charting the reaction space around a known Multicomponent Reaction (MCR) allows the development of new processes. In this way, a detailed study of the parameters involved in the Orru transformation leads to the generation of alternative connectivities featuring diversely substituted imidazolones, including GFP (Green Fluorescent Protein) chromophores, coelenterazine derivatives, natural products, kinase inhibitors, and bioprobes.

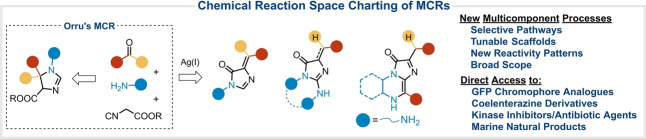

## Introduction

In the effort to colonize meaningful regions of the chemical space,^[1]^ access to new scaffolds is a priority.^[2]^ In this regard, multicomponent reactions (MCRs)^[3]^ offer undeniable advantages to combinatorially attain unconventional molecular connectivities, with high bond formation indexes, and exceptional atom, step, and time economies.^[4]^ These intermolecular domino processes^[5]^ comprise 3 or more reactants that form an adduct in a single operation through a unified reaction mechanism. Due to the complexity of an MCR (substrates, solvents, catalysts, conditions, reactive intermediates), the discovery of new processes is challenging, and frequently associated to serendipity. Efforts in recent years have focused on the rational design of new MCRs, including the single reactant replacement approach.^[6]^ However, the prevailing paradigm in MCR research has been to optimize the formation of a specific adduct and suppress potentially interesting by‐products.

**Figure 1:**
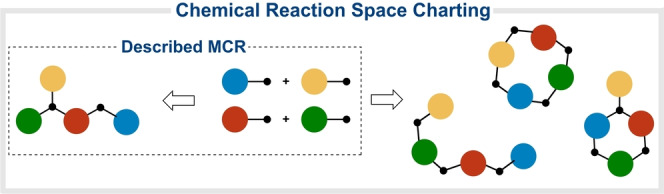
Charting the chemical reaction space of an MCR to generate alternative scaffolds.

To drive MCRs into a Diversity Oriented Synthesis context^[7]^ we propose a thorough charting of the chemical reaction space (defined as the network of feasible interactions connecting all species in a given system).^[8]^ Arguably, this would unravel alternative bond formation patterns, expanding the synthetic reach of MCRs (Figure 1). In our opinion, the kinetic selection of some pathways over the rest may support this hypothesis. This is in line with relevant findings validating the charting approach in other areas.[^9^, ^10^] Initial experiments where an Ugi MCR was switched to a Passerini MCR by using bulky amines seemed to follow this trend [see Supporting Information, Table S1]. Consequently, we propose a systematic screening of the reaction parameters and reactants involved in a given MCR, to rewire known routes and develop new transformations.^[11]^

**Scheme 1:**
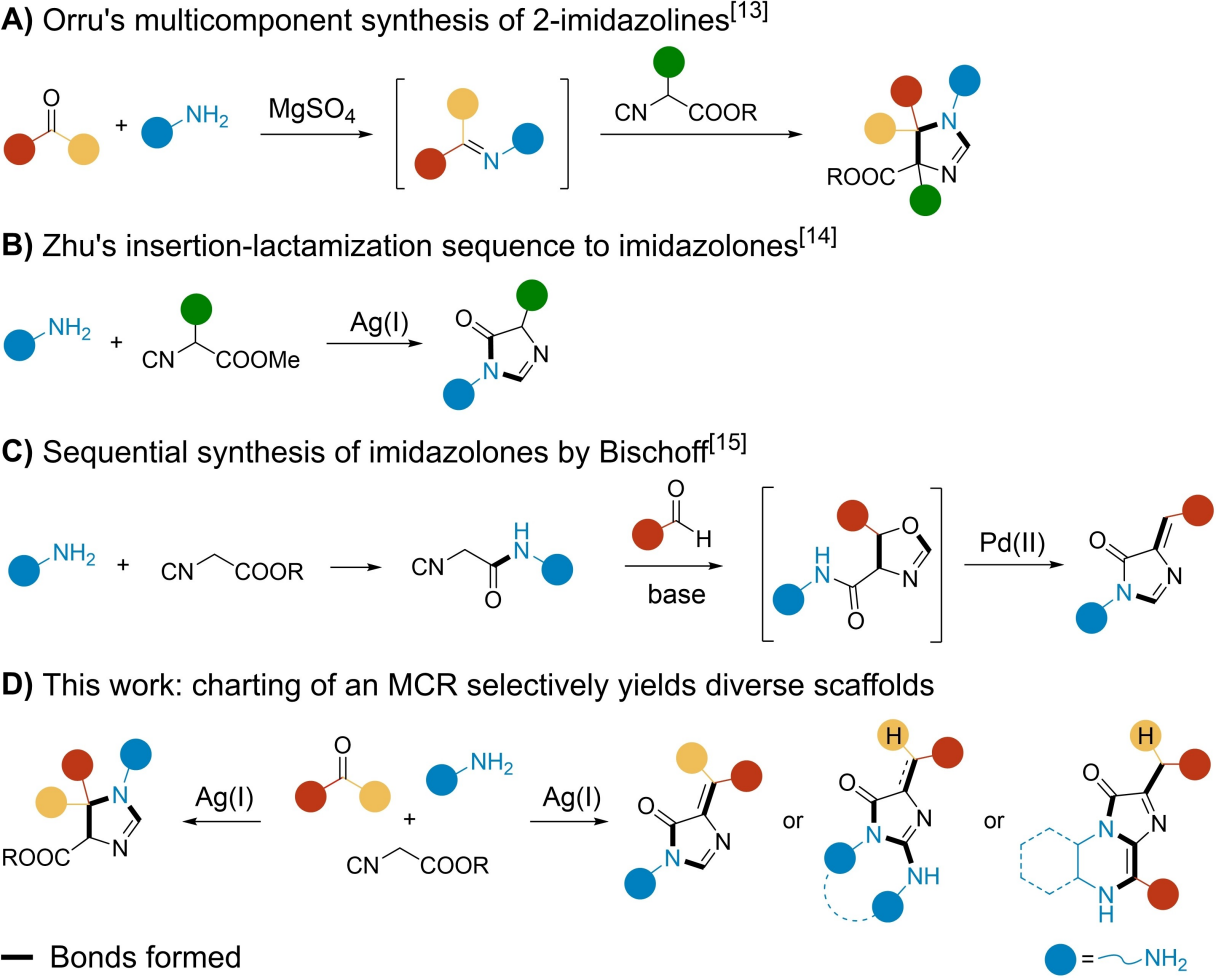
A) MCR to yield 2‐imidazolines by Orru.^[13]^ B) 4‐Imidazolone synthesis by Zhu.^[14]^ C) Sequential synthesis of 4‐imidazolones by Bischoff.^[15]^ D) This work: charting of the chemical space around the interaction of carbonyls, amines, and isocyanoacetates.

As a model combination, we chose carbonyls (aldehydes and ketones), amines, and α‐acidic isocyanides.^[12]^ This MCR was previously reported to yield 2‐imidazolines through the α‐nucleophilic attack on the imine followed by isocyanide insertion. The procedure was developed by Orru et al. and has found wide acceptance in organic chemistry (Scheme 1A).^[13]^ In a related work, Zhu and co‐workers described the reaction between amines and α‐substituted isocyanoacetates to access 4‐imidazolones (Scheme 1B).^[14]^ Moreover, Bischoff et al. recently reported the synthesis of 4‐imidazolones through a multistep approach (Scheme 1C).^[15]^ Herein, we describe an extensive exploration of the chemical reaction space around the aforementioned interaction to selectively yield several synthetic outputs in a controlled manner (Scheme 1D).

## Results and Discussion

### Charting of a Multicomponent Interaction

**Figure 2:**
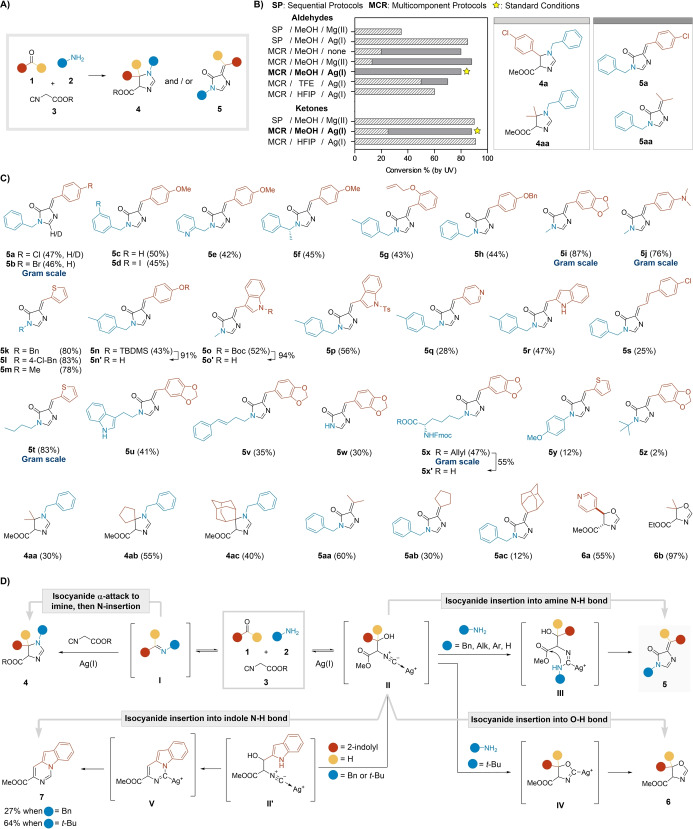
A) Possible outcomes of the MCR between carbonyls, amines, and isocyanoacetates. B) Role of additives and solvents in the process and selected standard conditions. C) Scope of the MCR under the standard conditions [MCR; AgNO_3_ (10 mol %); MeOH (0.2 M); rt for 17 h or 40 °C (μW) for 20 min]. D) Proposed reaction pathways and putative mechanisms.

Our initial experiments with 4‐chlorobenzaldehyde, benzylamine, and methyl isocyanoacetate in MeOH without additives gave the expected 2‐imidazoline **4 a**. However, we also detected the unsaturated imidazolone **5 a** (Figure 2A, B). Incidentally, compound **5 a** features a scaffold analogous to the chromophore of the Green Fluorescent Protein (GFP), one of the most used fluorescent tools in biochemistry and cell biology.^[16]^ These findings indicated that this interaction could be more divergent than previously reported, and could also provide a novel MCR‐based access to GFP fluorophore derivatives.[^15^, ^17^] Thus, we launched an extensive charting of the chemical reaction space around this combination (Figure 2B and Table S2, S3).

Sequential protocols, where the imine was pre‐formed, only generated the 2‐imidazoline **4 a**.^[13]^ In contrast, the process could be tuned to afford imidazolone **5 a** when subjected to a multicomponent procedure. In this context, some additives had a clear impact on the reaction outcome. Catalyst‐free protocols and activation by various metal salts such as Mg^II^, Pd^II^, and Rh^II^ gave a mixture of adducts **4 a** and **5 a**, illustrating the competitive nature of the interaction. Satisfyingly, the process was selectively driven towards the formation of imidazolone **5 a** when the reaction was catalyzed by Ag^I^ or Cu^II^ salts, AgNO_3_ being the most productive. As for the solvents, MeOH gave the highest conversions to adduct **5 a**. The use of other alcohols (EtOH and *i*PrOH) selectively generated compound **5 a** as well. However, their fluorinated counterparts, trifluoroethanol (TFE) and hexafluoroisopropanol (HFIP), remarkably reversed the outcome of the MCR to the formation of the 2‐imidazoline **4 a** in the presence of AgNO_3_, demonstrating the synthetic potential of a simple modification (Figure 2B and Table S2). Additionally, the outcome was analogous at room temperature (rt) or under microwave (μW) irradiation. These results were reproduced with acetone as the carbonyl component. Although AgNO_3_ catalysis gave a mixture of both structural types **4 aa** and **5 aa**, the former could be selectively generated either by pre‐formation of the imine or using HFIP as the solvent (Figure 2B and Table S3). Accordingly, we defined the standard conditions to access the imidazolones **5** as a multicomponent protocol in MeOH under AgNO_3_ catalysis. Notably, the developed procedure conveniently afforded pure analogues after simple filtration, even at gram scale in several cases (see below).

Next, we screened the scope of reactants testing a diverse combination of carbonyls **1 a**–**af** and amines **2 a**–**x** under our defined standard conditions (Figure 2C and Supporting Information, section 2.3). The reaction was consistently directed towards the formation of adducts **5** with aromatic aldehydes. A variety of substituted benzaldehydes gave the desired adducts **5 a**–**h** in moderate yields (42–50 %). Highly electron‐rich aldehydes such as piperonal, 4‐(dimethylamino)benzaldehyde, or thiophene‐2‐carboxaldehyde afforded the respective adducts **5 i**–**m** in higher yields (ca. 80 %). While 4‐hydroxybenzaldehyde and indole‐3‐carboxaldehyde did not react in the MCR (Table S4), their protected analogues gave adducts **5 n**–**p** in decent yields (43–56 %). Subsequent deprotection conveniently afforded compounds **5 n**′ and **5 o**′. Note that Boc removal from compound **5 o** gave derivative **5 o**′ as a mixture of diastereomers (Figure S2). These adducts **5 n**′–**o**′ are of particular importance, as they represent close analogues of the wild type chromophores of the green and cyan fluorescent protein, respectively. Contrarily, the electron‐deficient 4‐formylpyridine reacted poorly in the MCR, but still exclusively yielded adduct **5 q** (28 %). The corresponding imidazolones were also obtained from indole‐2‐carboxaldehyde (**5 r**, 47 %) and *trans*‐4‐chlorocinnamaldehyde (**5 s**, 25 %). Formaldehyde and glucose did not participate in the MCR, and simple aliphatic aldehydes gave complex reaction mixtures (Table S4). Notably, the MCR was stereoselective and only the *Z*‐diastereomer was generated (Figure 2C). The stereochemistry of the double bond was unequivocally determined by X‐ray crystallography of compound **5 h** (Table S9).^[18]^ While representative aromatic ketones did not react in the MCR (Table S4), the aliphatic ones always produced mixtures of scaffolds **4** and **5** under the standard conditions. In this way, 2‐imidazolines **4 aa**–**ac** (27–51 %) and imidazolones **5 aa**–**ac** (12–58 %) were generated from acetone, cyclopentanone, and 2‐adamantanone. Lastly, the incorporation of isatin resulted in barely productive and complex reaction mixtures (Figures S4–S6). In general, the new MCR efficiently leads to the imidazolone scaffold with high appendage diversity in practical yields, which may be further optimized in particular reactant combinations.

The amino input also had a relevant impact on the outcome of the MCR. Imidazolone adducts **5 a**–**v** were obtained from various alkyl amines, including an array of benzylamines, a homoallyl derivative, and tryptamine (25–85 %, Figure 2C). Remarkably, the use of deuterated benzylamine **2 a‐*d***_***2***_ conveniently led to the C‐2 D labeled imidazolone **5 a‐*d*** (47 %). Moreover, an NH_3_ solution in MeOH provided the corresponding *NH* imidazolone **5 w** (30 %). L‐Phenylalanine and its methyl ester did not afford the corresponding imidazolones **5** under standard conditions (Table S4). However, *N* ^α^‐Fmoc‐L‐lysine allyl ester yielded adduct **5 x** (47 %) in gram scale, efficiently linking the imidazolone scaffold to an amino acid. The Pd‐catalyzed deprotection of the allyl residue afforded the GFP chromophore derivative‐lysine conjugate **5 x**′ (55 %). Finally, 4‐methoxyaniline yielded the corresponding adduct **5 y** albeit in a lower yield (12 %), likely due to the decreased insertion rate of the isocyanides into the aniline N−H bond (see the mechanistic implications below).^[14]^ Interestingly, a bulkier input such as *tert*‐butylamine directed the process towards the generation of oxazolines **6**. In this way, compounds **6 a**–**b** were obtained from 4‐formylpyridine and acetone, respectively, in good yields (55–90 %). Even then, traces of the corresponding imidazolones **5** were detected and eventually isolated (**5 z**, 2 %, Table S5).

All these results suggest a complex interaction map of divergent reaction pathways (Figure 2D). Small variations in certain parameters may sufficiently alter the competing reaction rates to generate the observed structural diversity. It seems feasible that depending on the reaction medium, imine **I** and the Knoevenagel intermediate **II** may be reversibly generated or kinetically favored. Under suitable conditions, the formation and trapping of the imine **I** leads to the preferential generation of imidazolines **4**.^[13]^ Contrarily, we propose that isocyanide insertion into the N−H bond of the amine may lead to intermediate **III**, which in turn dehydrates and gives adducts **5** after a final lactamization. However, when N−H insertion is compromised due to the steric hindrance of bulky amines (e.g. *tert*‐butylamine), the reaction pathway is directed towards isocyanide insertion into the generated O−H bond to yield oxazolines **6** (Figure 2D).^[19]^ The input of indole‐2‐carboxaldehyde represented an interesting exception to support this mechanistic hypothesis. In this case, the MCR with benzylamine afforded a mixture of imidazolone **5 r** (47 %) and indolocarbazole **7** (27 %).^[20]^ This outcome is consistent with the intermediacy of putative adduct **II**′ and the subsequent insertion of the isocyanide into the indole N−H bond. Additionally, the use of *tert*‐butylamine, which inhibits the GFP pathway, resulted in an improved conversion to indolocarbazole **7** (60 %, Figures 2D and S7).

**Figure 3:**
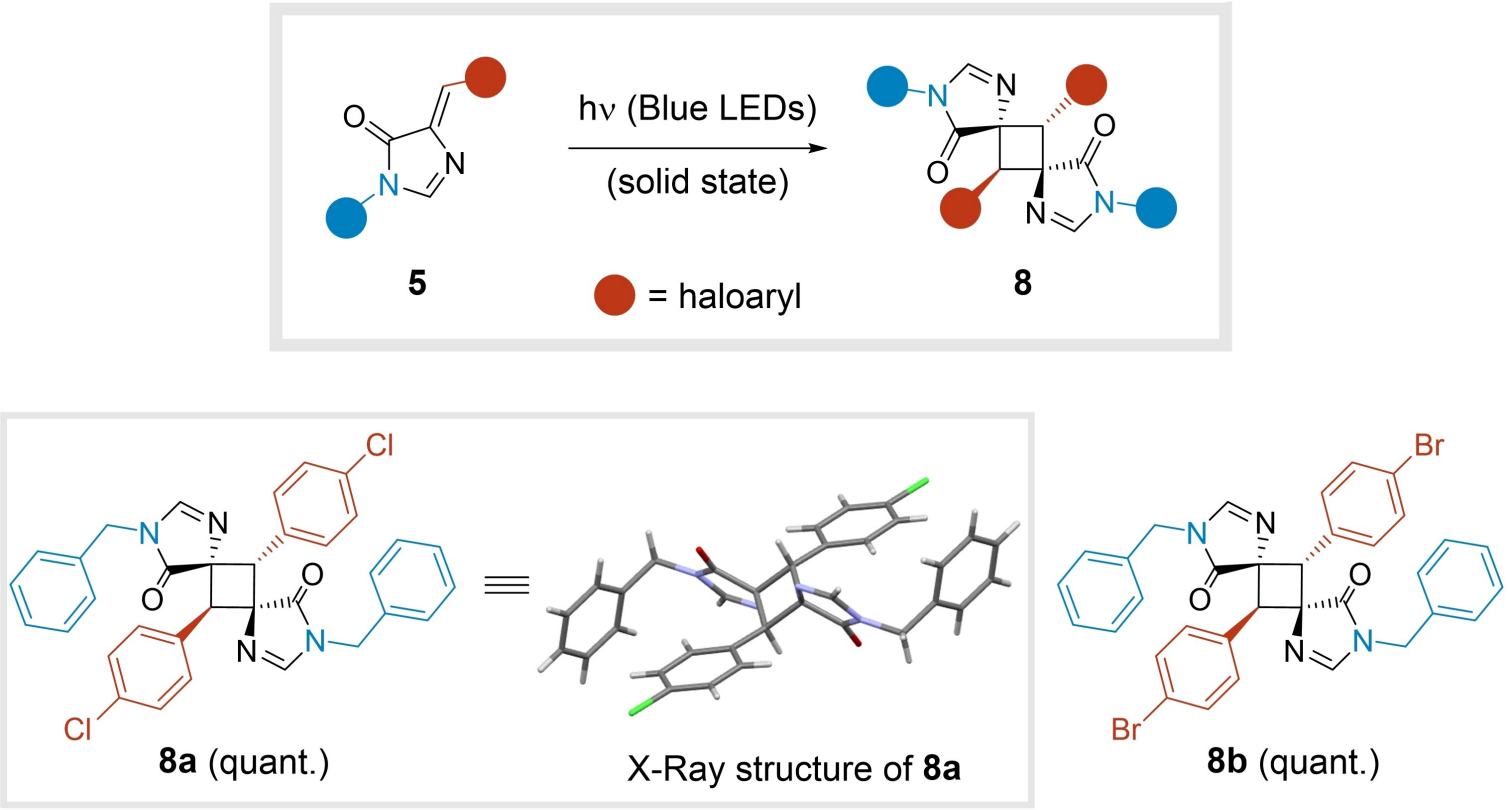
Photocatalyzed dimerization of compounds **5** to give cyclobutanes **8**.

Remarkably, we observed that adducts **5** containing a halogen‐substituted aryl group participated in a [2+2] photocycloaddition to generate cyclobutanes **8** in a stereoselective manner. The structural assignment of the centrosymmetric derivative **8 a** was confirmed by single crystal X‐ray diffraction (Figure 3 and Table S10).^[18]^ Although the reaction takes place spontaneously upon prolonged exposure to sunlight, a blue LED light source served to achieve full dimerization of **5 a**–**b** in the solid state. Compounds **8 a**–**b** were obtained through this simple post‐transformation in quantitative yields, expanding the number of scaffolds generated from the initial MCR (Figure 3). Notably, this transformation has not been described for imidazolones **5**.^[21]^

### Multicomponent Access to Coelenterazine Analogues

**Figure 4:**
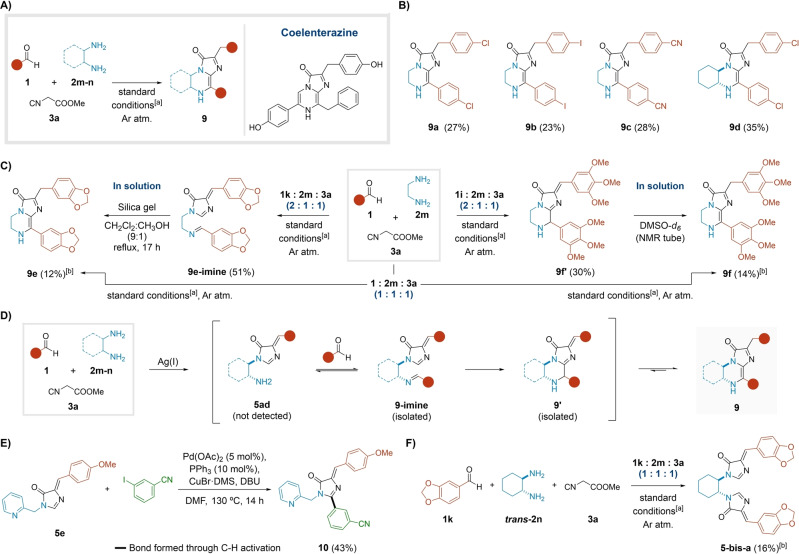
MCR with 1,2‐diamines: access to analogues of the natural product coelenterazine. A) General reaction Scheme and structure of coelenterazine. B) Scope of the process. C) Isolated intermediates and conversion to the coelenterazine derivatives **9**. D) Proposed reaction mechanism for the formation of adducts **9**. E) The C−H activation reaction. F) Formation of bis‐imidazolone adduct **5‐bis‐a**.^[a]^Standard conditions: MCR; AgNO_3_ (10 mol %); MeOH (0.2 M); rt for 17 h or 40 °C (μW) for 20 min.^[b]^Unoptimized yield: the reaction was performed with a 1 : 1 : 1 ratio of reactants.

The participation of bifunctional inputs in MCRs ponders the question of selectivity, usually generating mono‐ or bis‐ adducts.^[22]^ However, the use of ethylenediamine **2 m** in our MCR resulted in an extended multicomponent process^[23]^ to give adducts **9**. This process incorporates two aldehyde units in an ABB′C fashion, featuring an impressive bond formation index (Figure 4A). Compounds **9** are analogues of the luciferin coelenterazine, responsible for the bioluminescence in several aquatic organisms (Figure 4A).^[24]^ Remarkably, this scaffold was generated in one step, in a strikingly distinct approach from the biomimetic and reported synthetic procedures.[^24b^, ^25^]

After adjusting the stoichiometry, we prepared compounds **9 a**–**d** in moderate yields (ca. 30 %) from a variety of benzaldehydes **1**, ethylenediamine **2 m** or (±)‐*trans*‐1,2*‐*diaminocyclohexane ***trans‐*** **2 n**, and methyl isocyanoacetate **3 a**, under the standard conditions (Figure 4B). Unexpectedly, with piperonal **1 j** and 3,4,5‐trimethoxy benzaldehyde **1 z**, the desired coelenterazine analogues **9 e**–**f** were only generated under the suboptimal stoichiometry 1 : 1 : 1 and consequently isolated in lower yields (ca. 13 %, Figure 4B). Instead, when applying the optimal ratio of 2 : 1 : 1, adducts **9 e‐imine** (51 %) and **9 f**′ (30 %) precipitated from the reaction mixture, respectively (Figure 4C). To justify the observed trends, we propose the following unified mechanism. Likely, the process starts with the silver‐mediated formation of the imidazolone precursor **5 ad**, whose immediate condensation with a second unit of aldehyde unit gives the intermediate **9‐imine**. The activated imine presumably yields the bicyclic intermediate **9′**, which in turn may evolve towards species **9** under acidic catalysis (Figure 4D). The putative mechanism features the intriguing transformation of intermediate **9‐imine** to tautomer **9′**. Although arguable, our hypothesis contemplates an unprecedented intramolecular attack of a nucleophilic imidazolone upon an electrophilic activated imine (Figure S12). The potentially nucleophilic nature of imidazolones may be supported by a reported Pd‐catalyzed C−H activation mechanism at the C‐2 position,^[26]^ which served to synthesize compound **10** in 43 % yield (Figure 4E, for further mechanistic reflections see Supporting Information, section 2.5.1). As for the isomerization of **9′** to **9**, preliminary computational studies suggested a variety of tautomeric structures with comparable stabilities, likely favoring the natural connectivity of adducts **9** (Figures 4D and S14).

According to our empirical observations, we speculate that the precipitation of intermediates **9 e‐imine** and **9 f**′ in the reaction media might play a role in the interruption of the dynamic pathway towards adducts **9**. Consistently, both intermediates were fully converted to their corresponding coelenterazine isomers **9** in solution (Figures 4C and S12, S13). Interestingly, in an independent experiment with equimolar amounts of piperonal **1 k**, (±)‐*trans*‐1,2‐diaminocyclohexane ***trans**‐* **2 n**, and methyl isocyanoacetate **3 a**, we isolated the double imidazolone derivative **5‐bis‐a** (16 %, Figures 4F and S8) as a precipitate.

### Nucleophilic Addition to the Imidazolone Core

**Figure 5:**
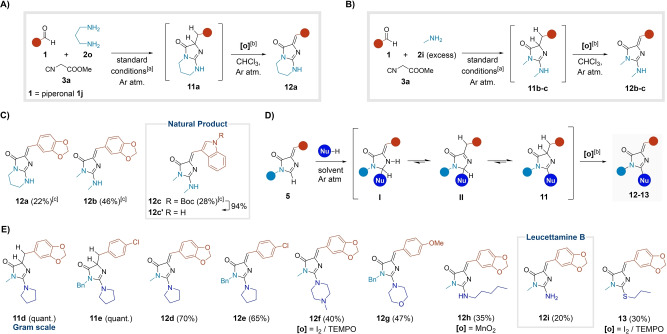
Addition of nucleophiles to the GFP core. A) New MCR with 1,3‐diaminopropane. B) Use of an excess of methylamine in the MCR. C) Examples derived from the new MCR‐oxidation process. D) Sequential protocol leading to adducts **12** and **13**. E) Scope of the sequential protocol.^[a]^Standard conditions: MCR; AgNO_3_ (10 mol %); MeOH (0.2 M); rt for 17 h or 40 °C (μW) for 20 min.^[b]^Unless otherwise stated, **[o]**=TEMPO.^[c]^Yield calculated over 2 steps (MCR‐oxidation).

Next, we evaluated the impact of longer chain diamines, on the MCR. In the case of 1,6‐hexanediamine, no imidazolone‐type species were formed (Figure S8). However, with 1,3‐propylenediamine **2 o**, the MCR unexpectedly yielded guanidine **11 a**. Moreover, we observed trace amounts (ca. 10 %) of the corresponding dehydrogenated 2‐aminoimidazolone **12 a** (Figures 5A and S8). Incidentally, this nucleus has wide‐ranging biological and pharmacological relevance. Several natural products from marine sponges and coral species feature this scaffold.^[27]^ Moreover, various analogues have been reported to have promising therapeutic profiles as antibiotics and neuroprotective agents, being potent and selective kinase inhibitors.^[28]^ Finally, they have also been studied as fluorescent probes.^[29]^ In addition, the oxidative conversion of **11 a** to **12 a** remarkably mimics the biosynthesis of the GFP chromophore,^[30]^ rendering a novel MCR‐based approach to 2‐aminoimidazolones synthesis.

We were able to reproduce the process intermolecularly by using an excess of methylamine **2 i** (10 equiv) in the MCR under the standard conditions. The corresponding intermediates **11 b**–**c** were formed, and again we detected trace amounts of **12 b**–**c** (Figure 5B). Switching to an inert atmosphere allowed for a cleaner formation of guanidines **11**. Several oxidation protocols fully converted compounds **11** to 2‐aminoimidazolones **12** (Figure 5A, B and Table S7). Among them, the use stoichiometric amounts of TEMPO minimized a known oxidative cleavage of the imidazolone,^[30]^ providing compounds **12 a**–**c** with fair yields (ca. 60 % for the oxidation step, Figure 5C). Notably, Boc‐deprotection of **12 c** directly afforded the natural product **12 c′**, an alkaloid present in the *Dendrophyllia* corals (26 % overall yield for 3 steps, Figure 5C).^[27a]^

Moreover, we developed a convenient protocol in which a previously isolated imidazolone **5** was reacted with a nucleophile under inert atmosphere to give adducts **11** quantitively, after simple evaporation or aqueous work‐up (Figure 5D). The addition reaction was strongly dependent on the nature of the incoming species (Table S6). Pyrrolidine reacted at room temperature, while more deactivated secondary amines and amylamine needed thermal activation. The reaction with ammonia required copper to promote the addition. Among other nucleophiles, 1‐propanethiol was successfully added to the imidazolone scaffold in basic conditions. In contrast, the incorporation of phenols and diethyl phosphite resulted in complex reaction mixtures (see Supporting Information, section 2.4). Finally, anilines, sulfinates, or 2‐methylindole did not react with adducts **5** under the conditions tested. In this way, guanidines **11 d**–**e** were isolated in quantitative yields and characterized as representative examples (Figure 5E). However, in most cases the crude intermediates **11** were directly dehydrogenated to yield the 2‐aminoimidazolones **12 d**–**h** and the sulfa adduct **13** (30–70 %, Figure 5E). Notably, the natural product leucettamine B (**12 i**, from *Leucetta* sponges)^[27b]^ was obtained in a single step from the MCR adduct **5 i** and ammonia, likely because the copper additive also promoted the in situ conversion to the oxidized product (20 %, Figure 5E).

We assume that scaffold **11** is generated through the unprecedented addition of a second amino functionality to the amidine moiety of the imidazolone core,[^28a^, ^31^] leading to the intermediate **I**. In turn, a series of tautomeric equilibria via the imine tautomer **II** yields the presumably more stable guanidine **11** (Figure 5D). Preliminary computational results and experiments with deuterated species supported the proposed addition mechanism, likely involving the intermediacy of radical species (see Supporting Information, Section 2.5.2).^[32]^

### Fluorescence Applications of (2‐Amino)Imidazolones

**Figure 6:**
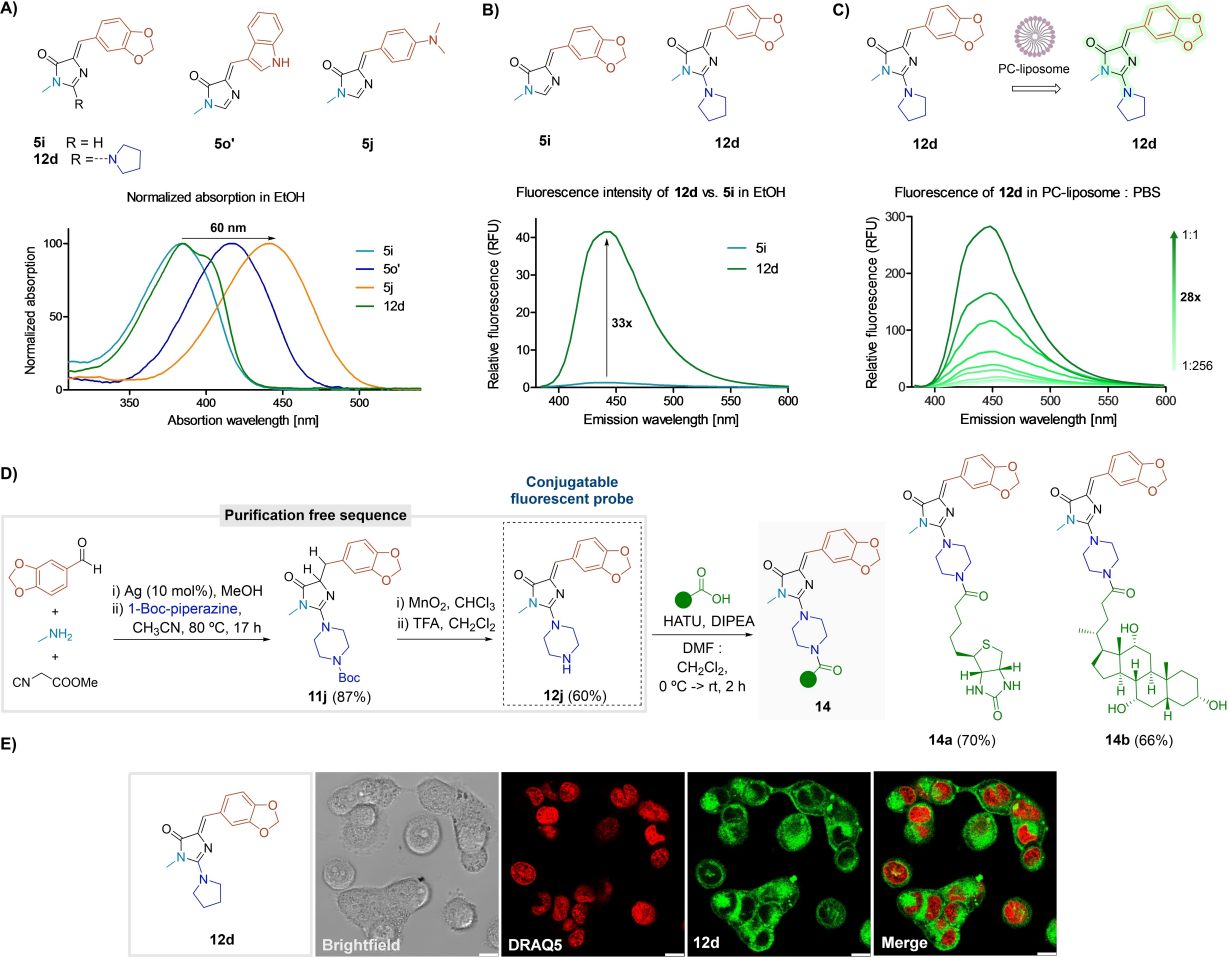
Photophysical studies. A) Normalized absorption spectra of compounds **5 i**, **5 o′**, **5 j**, and **12 d** in EtOH (100 μM). B) Emission spectra of **5 i** and **12 d** in EtOH (100 μM, *λ*_ext_: 360 nm). C) Emission spectra of **12 d** in increasing concentrations of phosphatidylcholine (PC)‐liposome in PBS (100 μM, *λ*_ext_: 360 nm). D) Synthesis of probe **12 j** and conjugation with biologically relevant carboxylic acids. E) Fluorescence images of **MDA‐MB‐231** human cancer cells after incubation with compound **12 d** (100 μM, 1 h, green) and **DRAQ5** (red) as a nuclear counterstain. Scale bar: 10 μm.

Considering the close analogy of the synthesized adducts to the GFP chromophore, we investigated their potential as fluorescent probes.^[33]^ The convenient structural tuning of our compounds allows for rapid modifications in their photophysical behavior. For instance, we observed a 60 nm bathochromic shift in the absorption maximum from adduct **5 i** to **5 j**. Compound **12 d** did not show significant differences to the chromophores **5** in the absorption pattern (Figure 6A).

The GFP chromophore does not emit in solution, as its fluorescence emanates from its fixed position in the protein environment.^[34]^ However, it has been shown that modifications such as tuning the central core or restricting the double bond rotation can lead to improved fluorogenic properties.[^29^, ^35^] Recently, this was also achieved through binding of the chromophore to other proteins.^[36]^ Indeed, our GFP chromophore‐type adducts **5** did not exhibit significant fluorescence in any of the tested solvents (Figure S26). Yet, the incorporation of the amino moiety at the C‐2 of the imidazolone resulted in a substantial increase of the fluorescence, as compound **12 d** showed over 30‐fold increase in the emission intensity in EtOH in respect to its parent adduct **5 i** (Figure 6B). In addition, the fluorescence of imidazolone **5 i** and its amino derivative **12 d** was remarkably increased in hydrophobic environments in comparison to aqueous media (Figures 6C and S27). Interestingly, we observed a significant decrease in the fluorescence intensity of compound **12 d** at low pH values (Figure S27).

Given the suitable photophysical properties of 2‐aminoimidazolones **12**, we envisaged their participation in a bioconjugation process. As a proof of concept, we obtained compound **12 j** in a convenient purification‐free synthesis with a decent global yield of 52 % and coupled it to biotin and cholic acid to give the final conjugates **14 a**–**b** (ca. 70 %, Figure 6D). The process was not detrimental to the emission of the final adducts (Figure S26).

Lastly, we demonstrated the compatibility of compound **12 d** for live‐cell imaging by incubating MDA‐MB‐231 cells followed by confocal fluorescence microscopy (Figure 6E). Fluorophore **12 d** showed strong intracellular signals, confirming cell permeability and suitability for fluorescence microscopy assays. Altogether, these results indicate the potential of 2‐aminoimidazolones **12** as tunable fluorescent motifs with excellent features for bioimaging applications.

## Conclusion

In summary, we have described how a systematic charting of the chemical reaction space around a known MCR not only defines the synthetic reach but, more importantly, leads to the discovery of rerouted and extended processes. In this way, the combination of carbonyls, amines, and isocyanoacetates was inspected to selectively access a wide array of biologically relevant heterocyclic scaffolds: GFP chromophore derivatives, coelenterazine analogues, imidazolines, oxazolines, etc. Incidentally, our approach has also prompted the discovery of new fundamental reactivity of the imidazolone scaffold. In our opinion, subtle kinetic changes within the chemical reaction space dictate the divergency of the MCR. In that sense, the charting approach provides a unified understanding of the possible interactions within a multicomponent combination and may lead to the discovery of new processes. We advocate for such explorations in MCRs to map the still dark regions of the chemical reaction space around these important transformations and expand their impact in Diversity Oriented Synthesis.

## Conflict of interest

The authors declare no conflict of interest.

1

## Supporting information

As a service to our authors and readers, this journal provides supporting information supplied by the authors. Such materials are peer reviewed and may be re‐organized for online delivery, but are not copy‐edited or typeset. Technical support issues arising from supporting information (other than missing files) should be addressed to the authors.

Supporting Information

Supporting Information

Supporting Information

## Data Availability

The data that support the findings of this study are available in the Supporting Information of this article.
