# Supplementary material for: Charting the Chemical Reaction Space around a Multicomponent Combination: Controlled Access to a Diverse Set of Biologically Relevant Scaffolds
Source: Angew Chem Weinheim Bergstr Ger. 2023 Jun 2;135(41):e202303889. doi: 10.1002/ange.202303889 (PMC10952208; doi:10.1002/ange.202303889)

checkCIF/PLATON report

Structure factors have been supplied for datablock(s) 1_a

THIS REPORT IS FOR GUIDANCE ONLY. IF USED AS PART OF A REVIEW PROCEDURE FOR PUBLICATION, IT SHOULD NOT REPLACE THE EXPERTISE OF AN EXPERIENCED CRYSTALLOGRAPHIC REFEREE.

No syntax errors found. CIF dictionary Interpreting this report

Datablock: 1_a

Bond precision:	C-C = 0.0040 A		Wavelength=1.54178
Cell:	a=5.9189(7)	b=7.4198(8)	c=21.621(2)
	alpha=90	beta=97.764(4)	gamma=90
Temperature:	180 K		

	Calculated	Reported
Volume	940.83(17)	940.84(18)
Space group	P 21	P 1 21 1
Hall group	P 2yb	P 2yb
Moiety formula	C24 H20 N2 O2	C24 H20 N2 O2
Sum formula	C24 H20 N2 O2	C24 H20 N2 O2
Mr	368.42	368.42
Dx,g cm-3	1.301	1.300
Z	2	2
Mu (mm-1)	0.664	0.664
F000	388.0	388.0
F000’	389.13	
h,k,lmax	7,9,26	7,9,26
Nref	3771[2039]	3583
Tmin,Tmax	0.887,0.967	0.532,0.754
Tmin’	0.767	

Correction method= # Reported T Limits: Tmin=0.532 Tmax=0.754
AbsCorr = MULTI-SCAN

Data completeness= 1.76/0.95 Theta(max)= 72.940

R(reflections)= 0.0426(3341) wR2(reflections)= 0.1143(3583)

S = 1.040 Npar= 255

The following ALERTS were generated. Each ALERT has the format
test-name_ALERT_alert-type_alert-level.
Click on the hyperlinks for more details of the test.

Alert level C

```
PLAT911_ALERT_3_C Missing FCF Refl Between Thmin & STh/L=    0.600         28 Report
PLAT913_ALERT_3_C Missing # of Very Strong Reflections in FCF ....         11 Note
```

Alert level G

```
PLAT032_ALERT_4_G Std. Uncertainty on Flack Parameter Value High .      0.300 Report
PLAT870_ALERT_4_G ALERTS Related to Twinning Effects Suppressed ..          ! Info
PLAT910_ALERT_3_G Missing # of FCF Reflection(s) Below Theta(Min).          2 Note
PLAT912_ALERT_4_G Missing # of FCF Reflections Above STh/L=  0.600         21 Note
PLAT916_ALERT_2_G Hooft y and Flack x Parameter Values Differ by .       0.20 Check
PLAT933_ALERT_2_G Number of OMIT Records in Embedded .res File ...          2 Note
```

```
   0 ALERT level A = Most likely a serious problem - resolve or explain
   0 ALERT level B = A potentially serious problem, consider carefully
   2 ALERT level C = Check. Ensure it is not caused by an omission or oversight
   6 ALERT level G = General information/check it is not something unexpected

   0 ALERT type 1 CIF construction/syntax error, inconsistent or missing data
   2 ALERT type 2 Indicator that the structure model may be wrong or deficient
   3 ALERT type 3 Indicator that the structure quality may be low
   3 ALERT type 4 Improvement, methodology, query or suggestion
   0 ALERT type 5 Informative message, check
```

It is advisable to attempt to resolve as many as possible of the alerts in all categories. Often the minor alerts point to easily fixed oversights, errors and omissions in your CIF or refinement strategy, so attention to these fine details can be worthwhile. In order to resolve some of the more serious problems it may be necessary to carry out additional measurements or structure refinements. However, the purpose of your study may justify the reported deviations and the more serious of these should normally be commented upon in the discussion or experimental section of a paper or in the "special_details" fields of the CIF. checkCIF was carefully designed to identify outliers and unusual parameters, but every test has its limitations and alerts that are not important in a particular case may appear. Conversely, the absence of alerts does not guarantee there are no aspects of the results needing attention. It is up to the individual to critically assess their own results and, if necessary, seek expert advice.

Publication of your CIF in IUCr journals

A basic structural check has been run on your CIF. These basic checks will be run on all CIFs submitted for publication in IUCr journals (*Acta Crystallographica*, *Journal of Applied Crystallography*, *Journal of Synchrotron Radiation*); however, if you intend to submit to *Acta Crystallographica Section C* or *E* or *IUCrData*, you should make sure that full publication checks are run on the final version of your CIF prior to submission.

Publication of your CIF in other journals

Please refer to the *Notes for Authors* of the relevant journal for any special instructions relating to CIF submission.

PLATON version of 05/12/2020; check.def file version of 05/12/2020

Datablock 1_a - ellipsoid plot

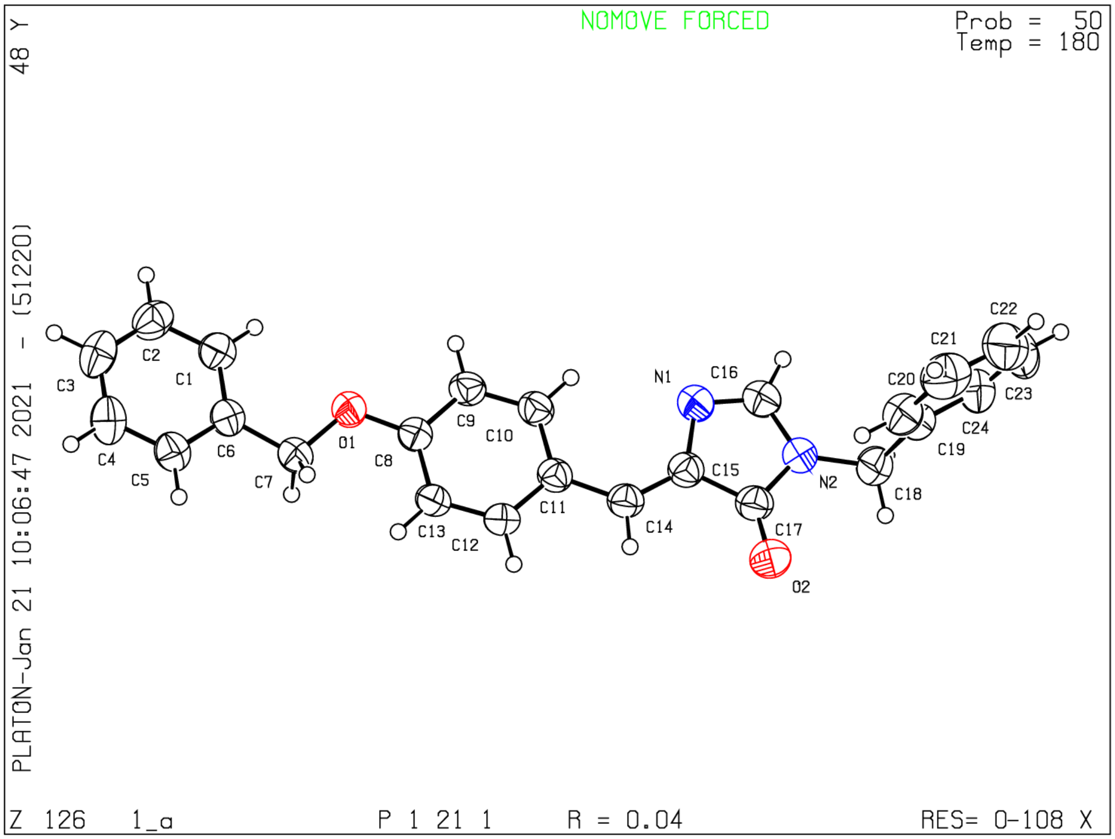

Supplement: Supplementary file 2 — Supporting Information [file ANGE-135-0-s003.pdf]
