# Supplementary material for: Charting the Chemical Reaction Space around a Multicomponent Combination: Controlled Access to a Diverse Set of Biologically Relevant Scaffolds
Source: Angew Chem Weinheim Bergstr Ger. 2023 Jun 2;135(41):e202303889. doi: 10.1002/ange.202303889 (PMC10952208; doi:10.1002/ange.202303889)

Angewandte Chemie
Eine Zeitschrift der Gesellschaft Deutscher Chemiker

Supporting Information

Charting the Chemical Reaction Space around a Multicomponent Combination: Controlled Access to a Diverse Set of Biologically Relevant Scaffolds

P. Nadal Rodríguez, O. Ghashghaei, A. M. Schoepf, S. Benson, M. Vendrell, R. Lavilla**

Table of Contents

1. General Information 4
2. Results and Discussion 5
 2.1. Preliminary Charting Experiments 5
 2.2. Charting of the MCR 6
  2.3.1. Inputs of the MCR 6
  2.3.2. Screening the Reaction Conditions 7
  2.3.3. Exploring the Scope of the MCR 9
 2.4. Study of the Addition-Dehydrogenation Sequence 14
  2.4.1. Inputs of the Nucleophilic Addition 14
  2.4.2. Screening the Nucleophilic Addition Conditions 15
  2.4.3. Dehydrogenation of Compounds **11** to Compounds **12** 16
 2.5. Mechanistic Studies 17
  2.5.1. Mechanistic Considerations on the Formation of Compounds **9** 17
  2.5.2. Studies on the Addition of Nucleophiles into the imidazolone core 21
3. Synthetic Procedures and Characterization Data 25
 3.3. General Synthetic Procedures 25
  3.3.1. General Procedure A: Synthesis of Compounds **4**-**7** and **9** 25
  3.3.2. General Procedure B: Sequential Synthesis of Compounds **4** 25
  3.3.3. General Procedure C: Synthesis of Compounds **8** 25
  3.3.4. General Procedure D: Synthesis of Compounds **12a**-**c** 26
  3.3.5. General Procedure E: Synthesis of Compounds **12d**-**h** and **13** 26
  3.3.6. General Procedure F: Synthesis of Compounds **14** 27
  3.3.7. Special Case I: Synthesis of Compound **10** 27
  3.3.8. Special Case II: Synthesis of Compound **12j** 27
 3.4. Synthesis and Characterization Data of Non-Commercial Starting Materials 28
 3.5. Characterization Data of Isolated Compounds 31
  3.5.1. Compounds **4** 31
  3.5.2. Compounds **5** 32
  3.5.3. Compounds **6-8** 42
  3.5.4. Compounds **9** 43
  3.5.5. Compound **10** 46
  3.5.6. Compounds **11** 46
  3.5.7. Compounds **12-13** 48
  3.5.8. Compounds **14** 52
  3.5.9. Compounds **S** 53
4. Fluorescence Studies 55
 4.3. In vitro Spectroscopy 55
 4.4. Confocal microscopy of MDA-MB-231 cells 56
5. Crystallographic Data 57
 5.3. Compound **5h** 57
 5.4. Compound **8a** 58
6. References 59
7. Author Contributions 59
8. Copies of NMR Spectra 60
 8.1. Non-Commercial Starting Materials 60

8.2. Compounds **4** 65
8.3. Compounds **5** 69
8.4. Compounds **6**-**8** 103
8.5. Compounds **9** 107
8.6. Compound **10** 117
8.7. Compounds **11** 118
8.8. Compounds **12**-**13** 124
8.9. Compounds **14** 136
8.10. Compounds **S** 138

1. General Information

Unless otherwise stated, all reactions were carried out under normal atmosphere in dried glassware. All chemicals were purchased from commercial sources and were used as received unless otherwise mentioned. Microwave reactions were performed on a Biotage Initiator Classic. Flash column chromatographies were performed on an Isolera Prime Biotage provided with dual UV detection over normal phase silica gel columns (prepacked: 5, 10 and 25 g; or refillable cartridges: 5 – 50 g), reverse-phase C18 columns (prepacked: 6, 12 and 30 g), or neutral aluminum oxide (Al_2O_3) columns (prepacked: 4 g, or refillable cartridges: 5 – 20 g). Thin layer chromatographies were performed on pre-coated Merk silica gel 60 F254 plates and visualized under UV light at 254 nm and 365 nm.

1.1. Analysis

The ^{1}H NMR spectra were recorded on a 400 MHz or 500 MHz NMR spectrometer. The ^{13}C NMR spectra were recorded at 101 MHz or 126 MHz. Chemical shifts were reported in ppm(δ) as s (singlet), d (doublet), t (triplet), dd (doublet of doublets), m (multiplet), br s (broad singlet), etc. The residual solvent signals were used as references. The University of Barcelona Mass Spectrometry Service performed the High-Resolution Mass Spectrometry analyses. LC-MS spectra were carried out on Agilent 1260 Infinity II. The analysis was conducted on a Poroshell 120 EC-C15 (4.6 mm × 50 mm, 2.7 µm) at 40 °C with mobile phase A (H_2O + 0.05% formic acid) and B (CH_3CN + 0.05% formic acid) using a gradient elution and flow rate 0.6 mL/min. The DAD detector was set at 254 and 365 nm. The injection volume was 5 µL. EPR spectra were recorded on a Bruker EMX-Plus 10/12 Bruker BioSpin spectrometer with a X-band microwave bridge EMX Premium X, a 10″ERO73 magnet and a power supply of 12 kW (ERO83). The temperature was controlled by a Bruker ER 4111 VT temperature control system. The Science and Technology Park (University of Burgos, Spain) carried out X-ray diffractions.

1.2. Photocatalyzed Reactions Set-Up

Flexible blue LED strips of 120 cm from buyledstrip.com (Netherlands) were used as the light source for photocatalyzed reactions. The light source was placed in a large crystallizing dish, around its inner circumference. The vial or round bottom flask containing the solid to be irradiated was placed in the center of the dish to ensure even distribution of the irradiation (~ 5 cm of distance between the vessel and the light source). The device was covered with aluminum foil to increase the reaction irradiation and avoid any dangerous exposure to LEDs.

2. Results and Discussion

2.1. Preliminary Charting Experiments

In preliminary experiments, while performing the following Ugi-4CR with sterically hindered amine and aldehyde components and an aromatic isocyanide (4-methoxyphenyl isocyanide), we also detected the formation of the Passerini adduct in competing yields (Entry 1, Table S1). Interestingly, as we switched to cyclohexyl isocyanide, the formation of the Ugi adduct was fully suppressed and only the Passerini Adduct was detected (Entry 2, Table S1). As the formation of the Ugi adduct is the result of isocyanide attack to the formed imine, we presume that bulky residues slow down/disfavor this attack and give rise to the alternative Passerini pathway. This effect is especially dominant in the case of the non-flat cyclohexyl residue in comparison with a flat methoxyphenyl one. In our opinion these results indicated that an MCR pathway can be rewired by slight alterations, rationalizing the potential usefulness of systematic charting studies around a known MCR combination.

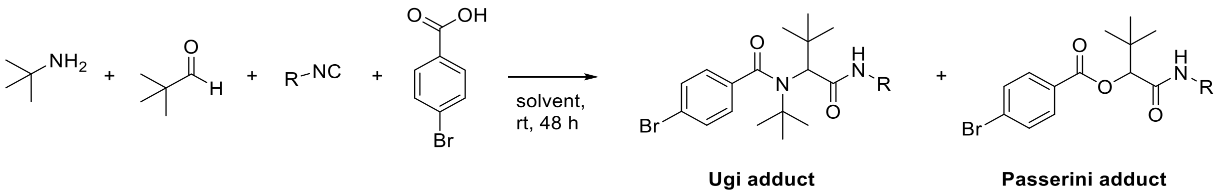

Table S1. Preliminary experiments supporting the need for charting an MCR.

Entry	R	Solvent	Ugi Adduct (%)[a]	Passerini Adduct (%)[a]
1	4-methoxyphenyl	MeOH	28	15
2	cyclohexyl	MeOH	0	25

[a]Conversion % calculated by LC-MS.

2.2. Charting of the MCR

2.3.1. Inputs of the MCR

Carbonyls (1a-1af)

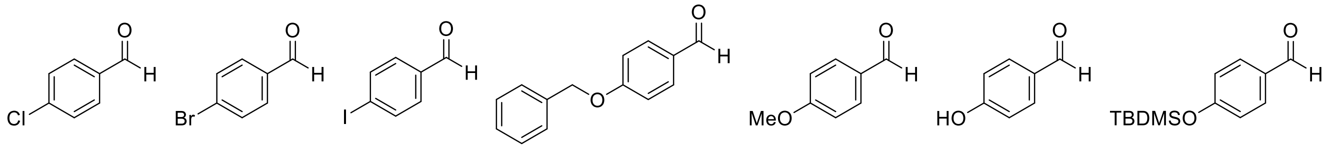

1a **1b** **1c** **1d** **1e** **1f** **1g**

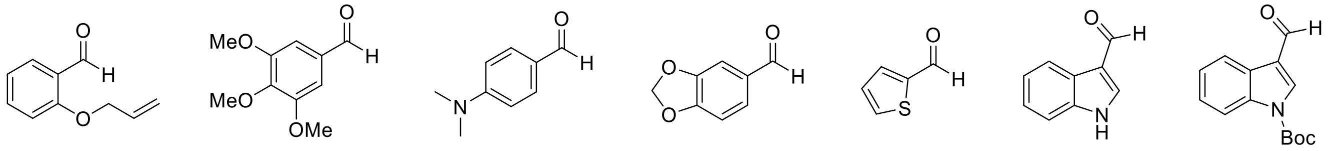

1h **1i** **1j** **1k** **1l** **1m** **1n**

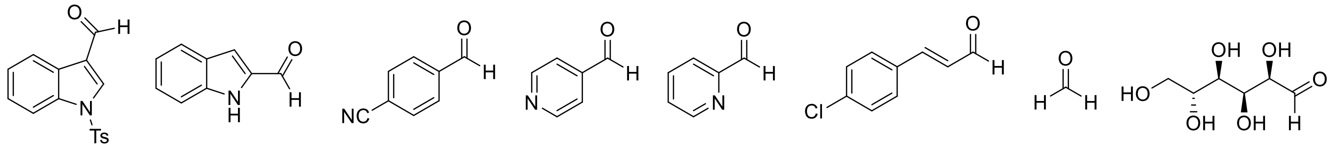

1o **1p** **1q** **1r** **1s** **1t** **1u** **1v**

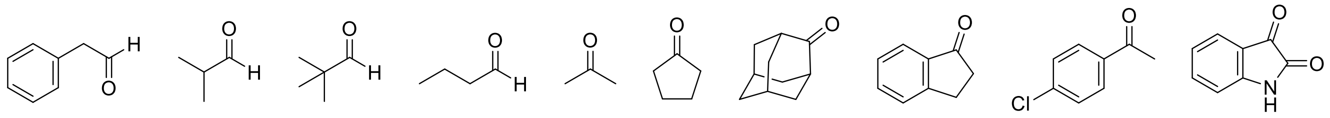

1w **1x** **1y** **1z** **1aa** **1ab** **1ac** **1ad** **1ae** **1af**

Amines and surrogates (2a-2x)

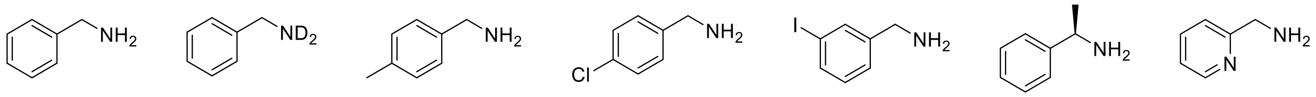

2a **2a-*d*2** **2b** **2c** **2d** **2e** **2f**

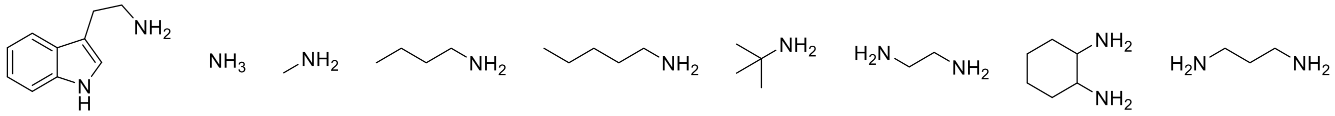

2g **2h** **2i** **2j** **2k** **2l** **2m** ***cis*-2n** ***trans*-2n** **2o**

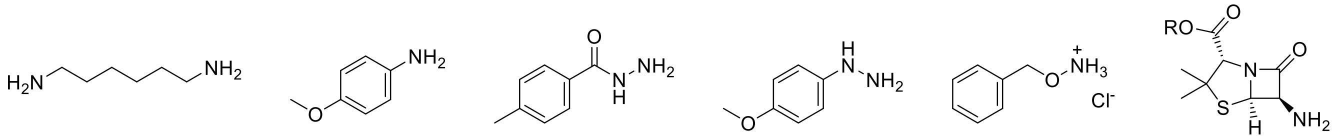

2p **2q** **2r** **2s** **2t** **2u** (R = H or Me)

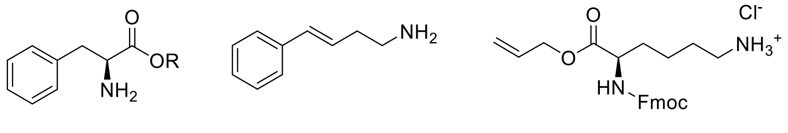

2v (R = H or Me) **2w** **2x**

Isocyanoacetates (3a-3b)

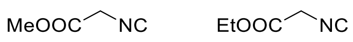

3a **3b**

2.3.2. Screening the Reaction Conditions

We studied the outcome of the MCR involving carbonyls, amines and isocyanoacectates depending on reaction conditions, solvent, and catalyst. We started charting the MCR with 4-chlorobenzaldehyde **1a**, benzylamine **2a**, and methyl isocyanoacetate **3a** (Table S2). Experiments in which the imine was preformed, either prior or *in situ*, with or without $MgSO_4$ (entries 1-3) only generated **4a**. Multicomponent protocols with $MgSO_4$ or without additives produced mixtures of **4a** and **5a** (entries 4-5). The use of $AgNO_3$ in a multicomponent protocol resulted in the selective formation of **5a**. Entries 6-10 show how varying the temperature, time and catalyst loading did not affect productivity. However, a change in the stoichiometry (excess of **3a**) decreased the yield of the MCR, although it was still selective towards **5a** (entry 11). As for the solvent, alcohols were the solvents of choice for the formation of **5a**, and addition of water as cosolvent was not detrimental (entries 12-14). Aprotic solvents such as CH_3CN and CH_2Cl_2 were unproductive and gave mostly imine (entries 15-16). The MCR with fluorinated alcohols (and $AgNO_3$) reversed the outcome of the reaction to the formation of **4a** (entries 17-18). Lastly, the effect of the catalyst was studied. Other silver and copper salts selectively gave **5a**, but with lower conversions than $AgNO_3$ (entries 19-22). Other metals gave either low production mixtures of **4a** and **5a** (entries 23-24) or unproductive reactions (entry 25).

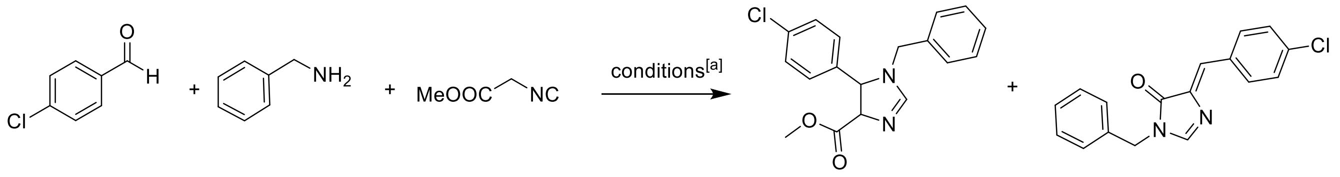

1a **2a** **3a** **4a** **5a**

Table S2. Charting of the MCR with 4-chlorobenzaldehyde **1a**.

Entry	Protocol	Solvent	Catalyst (10 mol%)[b]	Temperature (ºC)	Time	Conversion % by LC-MS (isolated yield)	
						4a	5a
1	One-pot sequential[c]	MeOH	$MgSO_4$ (1.5 eq.)	rt	48 h	45	0
2	One-pot sequential[c]	MeOH	$AgNO_3$	rt	17 h	85 (70%)	0
3	Stepwise (pre-formed imine)[d]	MeOH	$MgSO_4$ (1.5 eq.)	rt	17 h	65	0
4	MCR	MeOH	$MgSO_4$ (1.5 eq.)	rt	17 h	13	75
5	MCR	MeOH	-	rt	17 h	20	60
6	MCR	MeOH	$AgNO_3$	rt	17 h	0	75 (47%)
7	MCR	MeOH	$AgNO_3$	80	17 h	0	73
8	MCR	MeOH	$AgNO_3$ (20 mol%)	rt	17 h	0	76
9	MCR	MeOH	$AgNO_3$	40 (μW)	20 min	0	76 (46%)
10	MCR	MeOH	$AgNO_3$	80 (μW)	10 min	0	75
11	MCR[e]	MeOH	$AgNO_3$	rt	17 h	0	58 (21%)
12	MCR[f]	EtOH	$AgNO_3$	100 (μW)	10 min	0	69
13	MCR	*i*-PrOH	$AgNO_3$	120 (μW)	10 min	0	64
14	MCR	MeOH / H_2O (8 : 2)	$AgNO_3$	rt	17 h	0	80 (45%)
15	MCR	CH_3CN	$AgNO_3$	40 (μW)	20 min	0	0[g]
16	MCR	CH_2Cl_2	$AgNO_3$	rt	17 h	31	0[g]
17	MCR	TFE	$AgNO_3$	40 (μW)	20 min	50	20

18	MCR	HFIP	$AgNO_3$	40 (μW)	20 min	60	0
19	MCR	MeOH	Ag_2CO_3	40 (μW)	20 min	0	72
20	MCR	MeOH	AgOAc	40 (μW)	20 min	0	56
21	MCR	MeOH	$CuCl_2$	40 (μW)	20 min	0	39
22	MCR	MeOH	$Cu(OAc)_2$	40 (μW)	20 min	0	42
23	MCR	MeOH	$Rh_2(OAc)_4$	40 (μW)	20 min	10	25 (22%)[g]
24	MCR	MeOH	$PdCl_2$	40 (μW)	20 min	10	15 (10%)[g]
25	MCR	MeOH	$AuCl_3$	40 (μW)	20 min	0	0[g]

[a]Unless otherwise stated: **1a** (1.1 mmol), **2a** (1.1 mmol), **3a** (1 mmol), catalyst (0.1 mmol), solvent (5 mL). [b]Amount of catalyst was 10 mol% unless otherwise stated. [c]One-pot sequential protocol: **1a** (1.1 mmol), **2a** (1.1 mmol) and catalyst stirred for 4 h, then added **3a** (1 mmol), solvent (5 mL). [d]Stepwise protocol: *N*-benzyl-1-(4-chlorophenyl)methanimine (1.1 mmol), **3a** (1 mmol), catalyst, solvent (5 mL). [e]Different reaction stoichiometry: **1a** (1 mmol), **2a** (1 mmol), **3a** (1.1 mmol), catalyst (0.1 mmol), solvent (5 mL). [f]Ethyl isocyanoacetate **3b** (1 mmol) was used instead of **3a**. [g]Unproductive reactions (low or no amount of **4a** and **5a**) mostly produced *N*-benzyl-1-(4-chlorophenyl)methanimine.

Next, we selected key experiments from the charting with 4-chlorobenzaldehyde **1a** (entries 2, 6 and 18, Table S2) and reproduced them with acetone **1v** (Table S3). The results were comparable. With preformed imine *in situ*, the reaction only yielded **4aa** (entry 1). The MCR with $AgNO_3$ afforded a mixture of **4a** and **5a** (entry 2). Finally, the MCR with HFIP only yielded **4a** (entry 3).

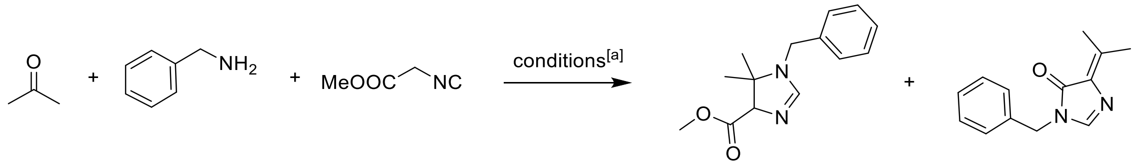

1v **2a** **3a** **4aa** **5aa**

Table S3. Charting of the MCR with acetone **1v**.

Entry	Protocol	Solvent	Catalyst (10 mol%)	Temperature (ºC)	Time	Conversion % by LC-MS (isolated yield)	
						**4aa**	**5aa**
1	One-pot sequential[b]	MeOH	$AgNO_3$	rt	48 h	90 (90%)	0
2	MCR	MeOH	$AgNO_3$	40 (μW)	20 min	30 (27%)	63 (58%)
3	MCR	HFIP	$AgNO_3$	40 (μW)	20 min	91	0

[a]**1v** (1.1 mmol), **2a** (1.1 mmol), **3a** (1 mmol), catalyst (0.1 mmol), solvent (5 mL). [b]One-pot sequential protocol: **1a** (1.1 mmol), **2a** (1.1 mmol) stirred for 4 h, then added **3a** (1 mmol) and catalyst (10 mol%), solvent (5 mL).

2.3.3. Exploring the Scope of the MCR

The scope of the MCR was explored with two goals: to assess the limitations of the new process, and to study the impact of the inputs of the MCR on its outcome, as part of the charting of the MCR. Unless otherwise stated, all experiments reported in this section were performed under the described standard reaction conditions: **1** (1.1 mmol), **2** (1.1 mmol), **3** (1 mmol) and $AgNO_3$ (10 mol%) in MeOH (0.2 M); rt for 17 h or 40 ºC (μW) for 20 min.

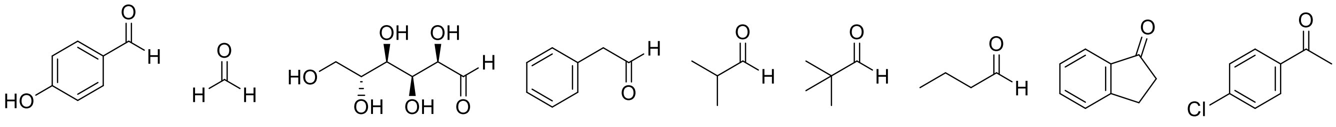

1f **1u** **1v** **1w** **1x** **1y** **1z** **1ad** **1ae**

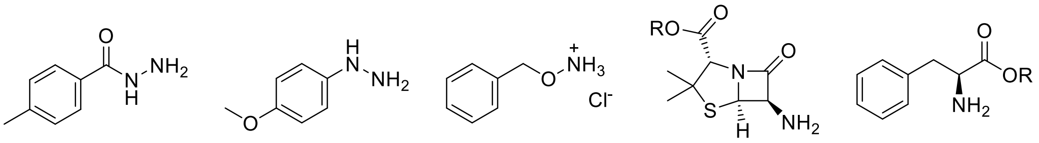

2r **2s** **2t** **2u** (R = H or Me) **2v** (R = H or Me)

Table S4. The following inputs did not yield any of the expected adducts **4-6** under the described standard conditions.

Entry	Input		Result
1	**1f**	4-Hydrozybenzaldehyde	No meaningful reaction was observed under the standard conditions. The lack of the reactivity of 4-hydroxybenzaldehyde **1f** in the MCR may correlate with the failure to generate the Knovenagel adducts under the standard conditions.[1]
2	**1u**	Formaldehyde[a]	Complex reaction mixtures. Likely formation of polymeric adducts.
3	**1v**	Glucose	No reaction observed.
4	**1w-1z**	Aliphatic aldehydes	Complex reaction mixtures. No detection of MCR adducts under the standard conditions.
5	**1ad-1ae**	Aromatic ketones	No reaction observed under the standard conditions. The low level of reactivity of aromatic ketones is likely due to their decreased electrophilicity, compared to dialkyl ketones.[2]
6	**2r**	Hydrazide	No detection of MCR adducts under the standard conditions. Mostly unreacted starting material recovered.
7	**2s**	Hydrazine	No detection of MCR adducts under the standard conditions. Mostly unreacted starting material recovered.
8	**2t**	Hydroxylamine[b]	No detection of MCR adducts under the standard conditions. Mostly unreacted starting material recovered.
9	**2u**	6-Aminopenicillanic acid (6-APA) or 6-APA methyl ester[b]	No reaction observed under the standard conditions. Deactivated primary amines do not yield the MCR adducts, likely due to their reduced nucleophilicity.
10	**2v**	L-phenylalanine or L-phenylalanine methyl ester[b]	No reaction observed under the standard conditions. Deactivated primary amines do not yield the MCR adducts, likely due to their reduced nucleophilicity.

[a]Formaldehyde **1t** was used as its polymer form: paraformaldehyde. [b]The O-benzylhydroxylamine **2t**, the 6-APA methyl ester (**2u**, R = Me), and the L-phenylalanine methyl ester (**2v**, R = Me) were used as the hydrochloride form, which was neutralized by addition of 1 eq. of trimethylamine in the MCR.

Some inputs did react in the MCR but resulted in unconventional results (Figure S1). We describe the corresponding outcome in each particular case.

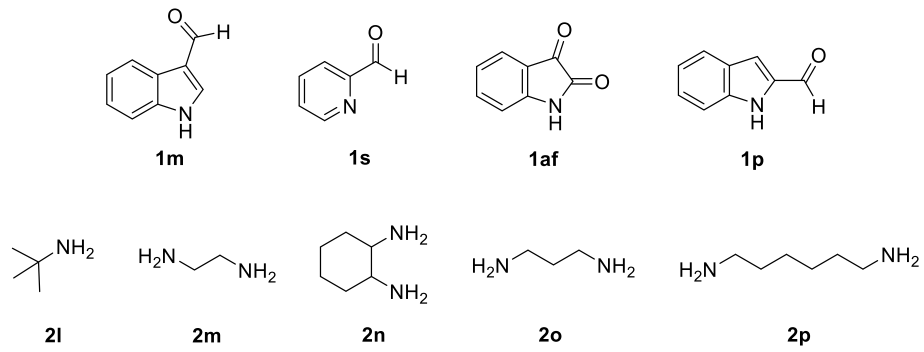

Figure S1. Inputs that resulted in unconventional results.

<u>The case of indole-3-carboxaldehyde **1m**</u>

The unprotected indole-3-carboxaldehyde **1m** did not react in the MCR under the standard conditions. The Boc- and Ts- protected indole-3-carboxaldehydes **1n-o** gave the desired imidazolones **5o-p** in good yields and, consistently with the general trend, were generated as a single diastereomer. However, deprotection of the adduct **5o** gave **5o'** as a mixture of diastereomers (Figure S2).[2]

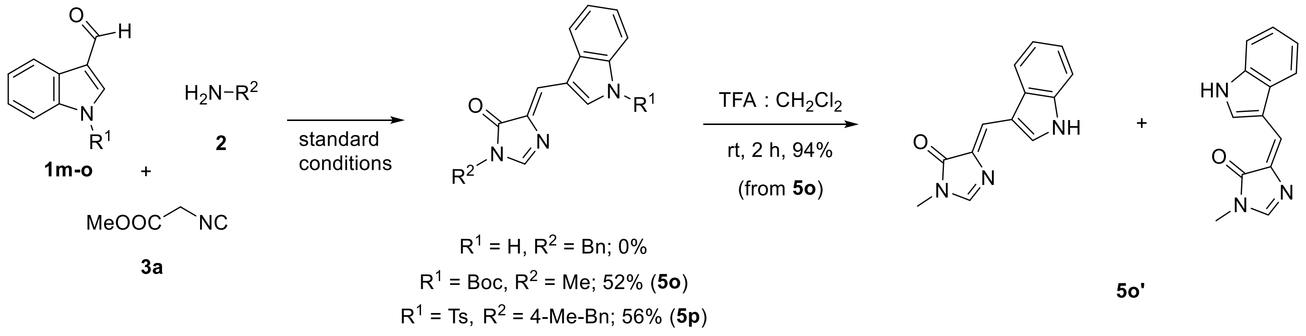

Figure S2. MCR with (un)protected indole-3-carboxaldehyde, amines and methyl isocyanoacetate **3a**.

<u>The cases of 2- formylpyridine **1r** and 4-formylpyridine **1s**</u>

Electron deficient aromatic aldehydes reacted poorly in the MCR under the standard conditions. In this way, the adduct **5q** was formed in 28% yield form 4-formylpyridine **1r**. With 2-formylpyridine **1s**, we detected the expected *m/z* 298 $[M+H]^+$ by LC-MS. However, the ^1H NMR of the reaction crude suggested a highly complex reaction mixture and the expected imidazolone **S1** could not be isolated after several attempts to purify with chromatography and recrystallization (Figure S3).

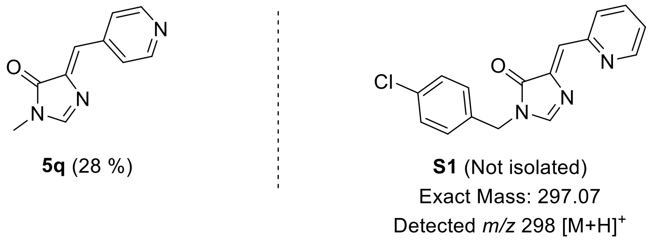

Figure S3. Outcomes of the MCR with pyridine aldehydes.

The case of isatin **1af**

We investigated the participation of an activated carbonyl such as isatin **1af** in the MCR. In the first experiments with an alkyl amine, we did not detect the expected imidazolone adducts **S3,** and LC-MS analysis suggested the presence of a second unit of amine (even when only 1 equivalent of the amine was used in the MCR). We hypothesized that due to the higher reactivity of the imidazolone scaffold with the isatin moiety, the addition of a second unit of the amine (as described in the main text and SI: sections 2.4 and 3.1) was faster than the MCR. Thus, the corresponding adducts **S3** were never observed and the 2-aminoimidazolone **S2** was detected (Figure S4).

The conversion to these presumed adducts was very low and its isolation was problematic, which resulted in very low amounts of impure products after chromatography. Nonetheless, the ^{1}H NMR and LC-MS data of the impure isolated products showed evidence that is consistent with this hypothesis.

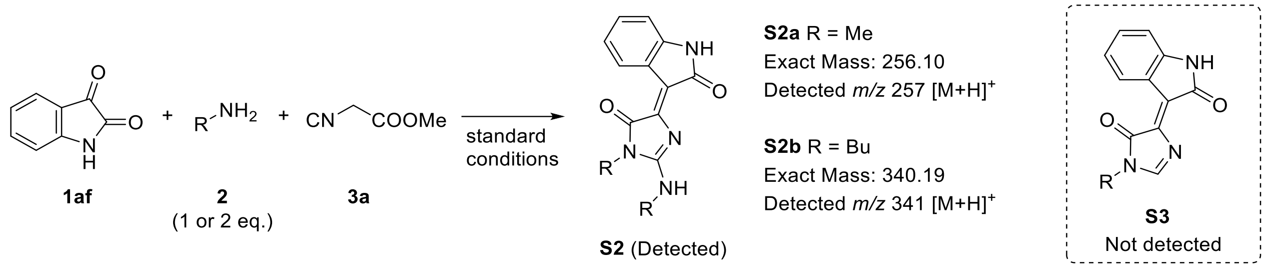

Figure S4. MCR with isatin **1af**, primary aliphatic amines, and methyl isocyanoacetate **3a**.

We also reacted isatin **1af** with 1,3-diaminopropane **2o**, and methyl isocyanoacetate **3a** in an attempt to emulate the result we obtained with piperonal **1j** (see main text, compounds **11a** and **12a**). Although the desired *m/z* was detected by LC-MS, again only a very low amount of impure product consistent with **S4** was obtained (Figure S5).

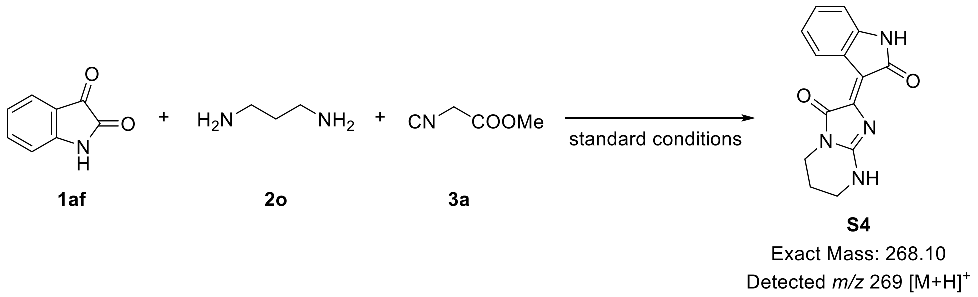

Figure S5. MCR with isatin **1af**, 1,3-propylenediamine **2n**, and methyl isocyanoacetate **3a**.

Lastly, we pondered whether we could develop a new 4-CR by reacting isatin **1af** with butylamine **2j** and 4-methoxyaniline **2q**. The hypothesis was that butylamine **2j** would react in the MCR and the 4-methoxyaniline **2q** would add into the generated imidazolone. This hypothesis was supported by the fact that isocyanides have a lower insertion rate into aromatic N-H bonds.[3] Similar to the other experiments, we obtained very low amounts of an impure sample – although presumably consistent with the expected adduct **S5** – from a highly complex reaction mixture (Figure S6).

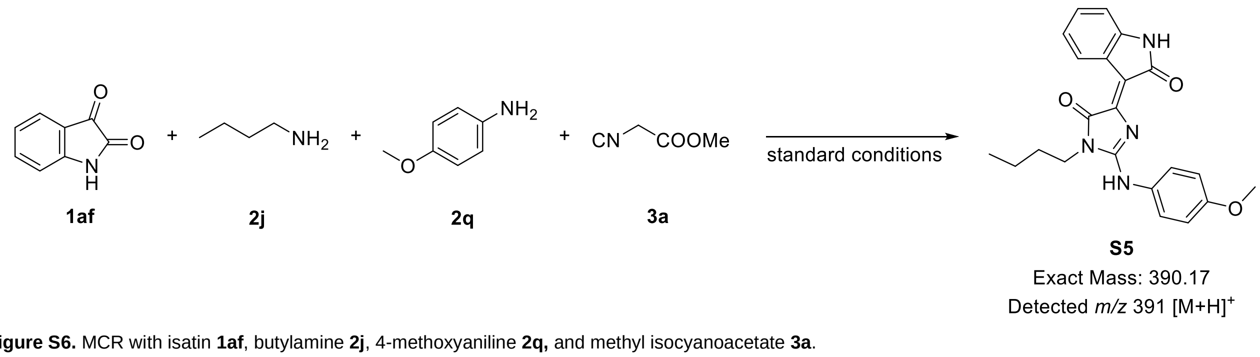

Figure S6. MCR with isatin **1af**, butylamine **2j**, 4-methoxyaniline **2q,** and methyl isocyanoacetate **3a**.

The case of indole-2-carboxaldehyde **1p**

The MCR with indole-2-carboxalehyde **1p** and benzylamine **1a** under the standard conditions gave the expected adduct **5r** (47%) together with indolocarbazole **7** (27%, Figure S7). The formation of this mixture results from the competitive nature of the isocyanide insertion into the benzylamine N-H bond and the indole N-H bond.

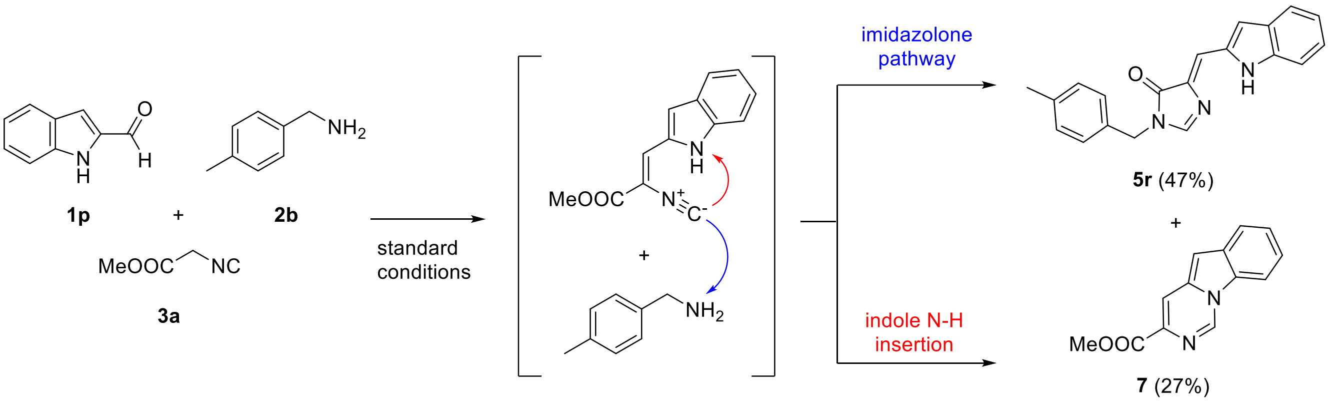

Figure S7. MCR with indole-2-carboxalehyde **1p,** benzylamine **1a** and methyl isocyanoacetate **3a**.

The case of *tert*-butylamine **2l**

The sterically hindered *tert*-butylamine **2l** was subjected to the MCR with a range of carbonyls. We first performed the MCR with acetone **1aa**. In this case neither the imidazoline **4** nor the imidazolone **5** were detected, and oxazoline **6b** was isolated almost quantitatively (Table S5, Entry 1). Then we performed the MCR with aromatic aldehydes. Again, the major products of the MCR were the corresponding oxazolines **6** (Entries 2-3). However, with piperonal **1k** traces of the adduct **5z** were detected, and it could be isolated in 2% yield (Entry 3).

Finally, we pondered whether the formation of indolocarbazole **7** (see above) could be promoted by the participation of *tert*-butylamine **2l** in the MCR with indole-2-carboxalehyde **1p**. Indeed, the formation of indolocarbazole **7** was increased to a 64% yield, and traces of the corresponding oxazoline **6** were detected. The corresponding compounds **4-5** were not detected in this case.

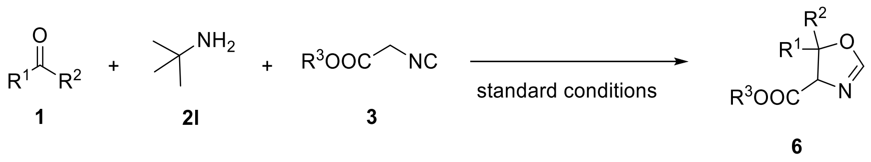

Table S5. Results of the MCR with *tert*-butylamine **1l** and different carbonyls.

Entry	Carbonyl **1**	Isocyanoacetate **3**	Conversion % by LC-MS (isolated yield)			
			2-imidazoline **4**	imidazolone **5**	oxazoline **6**	indolocarbazole **7**
1[a]	**1aa**	**3b**	0	0	**6b** 99 (97%)	- [b]
2	**1r**	**3a**	0	0	**6a** 60 (55%)	- [b]
3	**1k**	**3a**	0	**5z** traces (2%)	58 (not isolated)	- [b]
4	**1p**	**3a**	0	0	traces	**7** 75 (64%)

[a]The result was analogous when a sequential protocol was applied: **1aa**, **2l** and $MgSO_4$ in MeOH stirred for 4h, then **3b**. [b]The generation of compound **7** was not possible.

The case of diamines

The incorporation of diamines in the MCR was a clear example of the importance of charting the chemical reaction space around an MCR. By only altering one parameter in the reaction (i.e. the type of diamine input), the outcome of the MCR was completely altered. Moreover, these variations were in principle unpredictable. Remarkably, the outcome in each scenario was highly selective, as in most cases no mixtures were detected. Here we summarize the results of screening the MCR with equimolar amounts of piperonal **1k**, methyl isocyanoacetate **3a**, and a variety of diamines (Figure S8A).

The experiment with ethylenediamine **2m** exclusively afforded the coelenterazine analogue **9e** and neither of the expected mono- or bis-imidazolone adducts were detected. (See section 3.1 for details).

In the case of (±)-*trans*-1,2-diaminocyclohexane ***trans*-2n**, the bis-imidazolone adduct **5-bis-a** was isolated as the single adduct (16%). However the *m/z* consistent with the corresponding adduct **5** (*m/z* 314 $[M+H]^+$) and coelenterazine analogue **9** (*m/z* 446 $[M+H]^+$) were also detected by LC-MS, albeit in trace amounts. In contrast, the *cis*-1,2-diaminocyclohexane ***cis*-2n** resulted in a highly complex reaction mixture and none of the imidazolone-type derivatives were detected by LC-MS.

In the case of 1,3-propylenediamine **2o**, the formation of the imidazolone was followed by the intra molecular nucleophilic addition of the second NH_2 to the imidazolone scaffold. (See section 3.2 for details).

Finally, with the longer chain 1,6-hexanediamine **2p**, a highly insoluble solid – which accounted for 53% of the mass balance – was recovered from the reaction. We hypothesize that the bis-imine **S6** precipitated from the reaction mixture, stopping the evolution to any of the other possible adducts. Structure confirmation of the proposed bis-imine **S6** was challenging due to the low solubility of the product. LC-MS analysis showed the presence of piperonal **1k**, consistent with *in-situ* hydrolysis of the bis-imine in the acidic aqueous HPLC method.

Note that the use of (±)-*trans*-1,2-diaminocyclohexane ***trans*-2n** in the MCR with 2 equivalents of 4-chlorobenzaldehhde **4a** resulted in the formation of compound **9d**. In this case no species/intermediates precipitated from the reaction mixture and therefore the MCR progressed to the formation of the colenterazine analogue (Figure S8B; see main text and SI, section 3.1 for details).

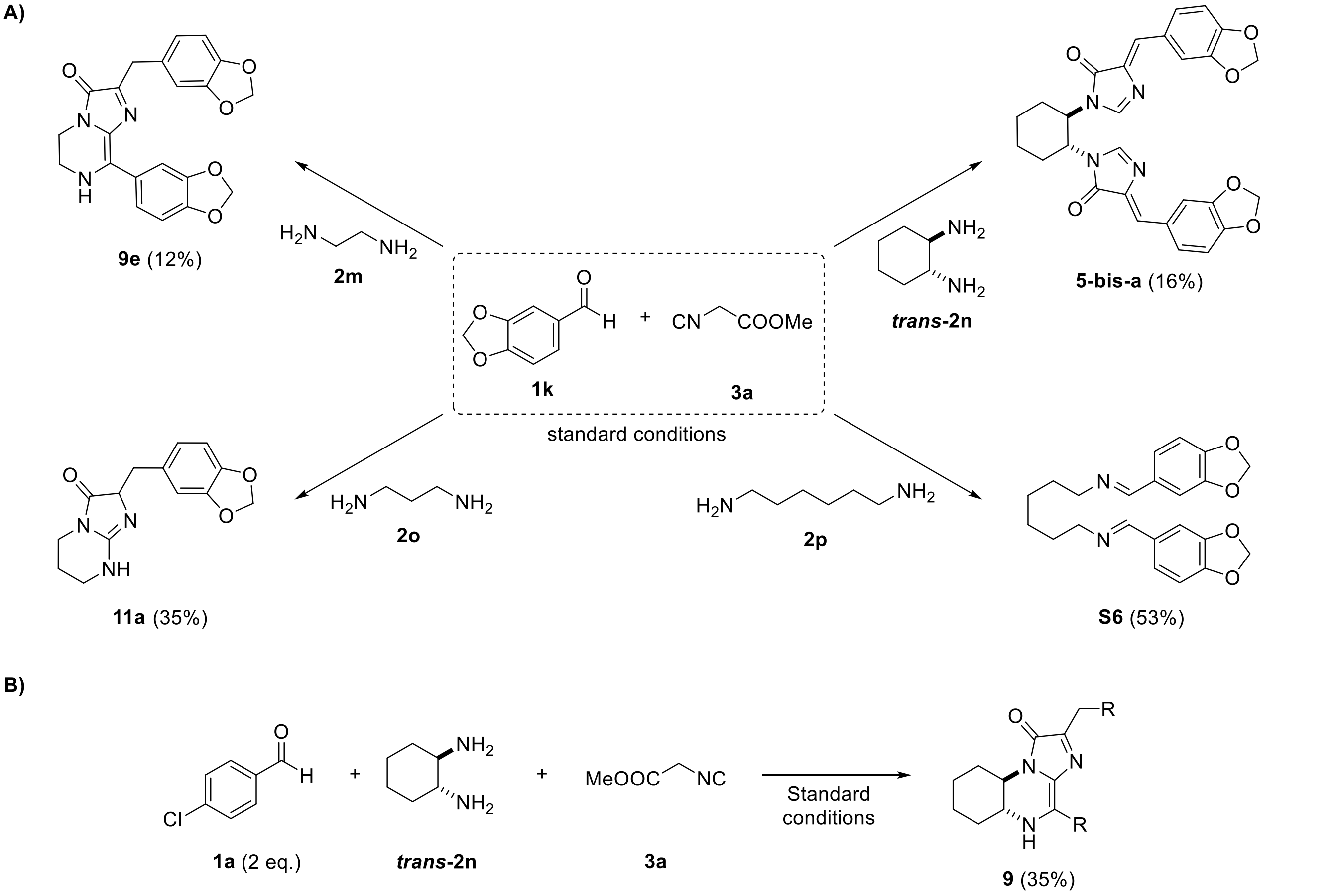

Figure S8. A) Variations in the outcome of the MCR with piperonal **1k**, methyl isocyanoacetate **3a**, and a range of diamines under the standard conditions. B) The MCR with 2 eq. of 4-chlorobenzalehyde **1a**, (±)-*trans*-1,2-diaminocyclohexane ***trans*-2n**, and methyl isocyanoacetate **3a** under the standard conditions.

2.4. Study of the Addition-Dehydrogenation Sequence

2.4.1. Inputs of the Nucleophilic Addition

The following nucleophiles were successfully added to an imidazolone adduct **5** under described conditions (See section 2.4.2).

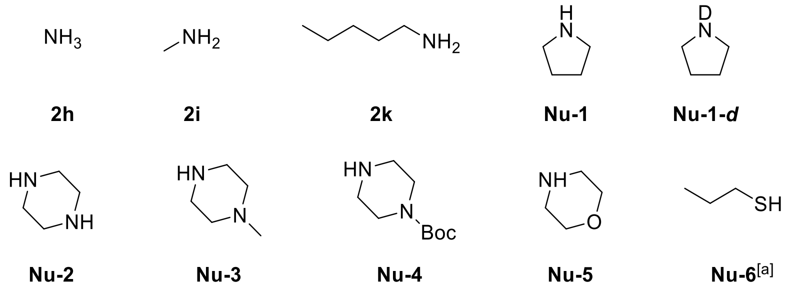

Figure S9. Nucleophiles successfully added to the imidazolone core. [a] Caesium carbonate was added in the reaction.

The following nucleophiles did not give the expected adducts **11** (Figure S10). Generally, the starting material **5** was recovered unreacted. However, with phenols **Nu-13** and **Nu-14** and diethyl phosphite **Nu-15**, the imidazolone **5** was consumed, but the reaction generated highly complex crudes and the corresponding adducts **11** were not detected.

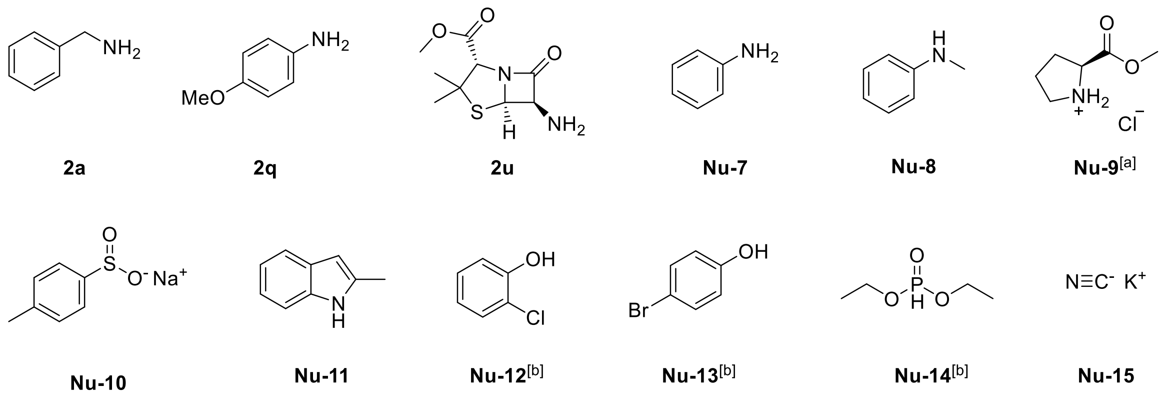

Figure S10. Nucleophiles that failed to give adducts **11**. [a]Triethylamine was added in the reaction. [b]Caesium carbonate was added in the reaction.

2.4.2. Screening the Nucleophilic Addition Conditions

The reaction of imidazolones **5** with a variety of nucleophiles gave adducts **11**. The conditions necessary for the addition varied depending on the nucleophile (Table S6).

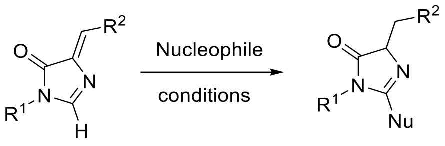

5 **11**

Table S6. Summary of applied reaction conditions for the nucleophilic addition to the imidazolone scaffold **5**. All reactions were performed in CH_3CN (0.1 M) and under argon atmosphere.

Entry	Substrate (imidazolones **5**)	Nucleophile	Equivalents of nucleophile	Temperature	Time	Additive	Conversion % to **11** (by LC-MS)
1a	**5i**	NH_3[a]	30	rt	17 h	-	0
1b	**5i**	NH_3[a]	30	80 ºC (μW)	30 min	$CuCl_2$ (1 eq.)	60
2	**5i**	CH_3NH_2[b]	10	rt	17 h	-	100
3a	**5i**	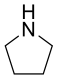	1	rt	17 h	-	80
3b	**5i**	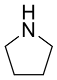	5	rt	8 h	-	100
4a	**5i**	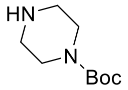	1	80 ºC	17 h	-	20
4b	**5i**	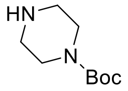	5	80 ºC	17 h	-	100
5a	**5i**	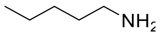	5	rt	17 h	-	0
5b	**5i**	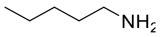	5	80 ºC	17 h	-	100
6a	**5c**	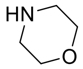	5	rt	17 h	-	0
6b	**5c**	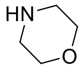	5	80 ºC	17 h	-	100
7	**5i**	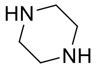	5	80 ºC	17 h	-	100
8a	**5i**	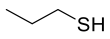	1	rt	17 h	-	0
8b	**5i**	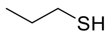	1	rt	3 h	Cs_2CO_3[c] (1.1 eq.)	100

[a]NH_3 was used as a 7 M solution in MeOH. [b]CH_3NH_2 was used as a 9.8 M solution in MeOH.

2.4.3. Dehydrogenation of Compounds 11 to Compounds 12

We observed that adducts **11** were spontaneously converted to **12**, either when the addition reaction was performed under open air or when adducts **11** were stored as solids in open air. In these cases, the average conversion was *ca*. 10% (Figure S11).

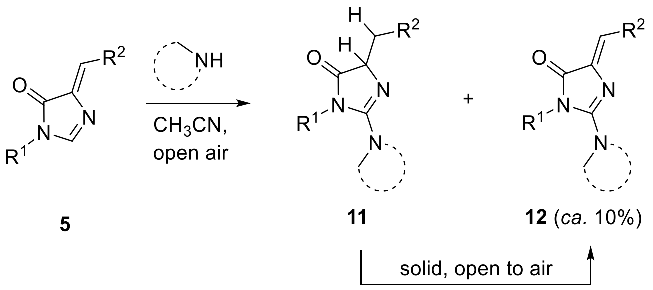

5 **11** **12** (*ca.* 10%)

Figure S11. Addition of nucleophiles to the imidazolone scaffold and spontaneous dehydrogenation under open air.

A range of oxidants were tested to transform adduct **11d** to its conjugated counterpart **12d** (Table S7). The use of Iodine or MnO_2 as oxidants resulted in some degradation of imidazolone scaffold via cleavage of the conjugated double bond. This degradation was consistent with a thorough report by Tsuji *et al*. on the oxidation mechanism of the GFP chromophore.[4] The use of a stoichiometric amounts of TEMPO minimized the observed degradation.

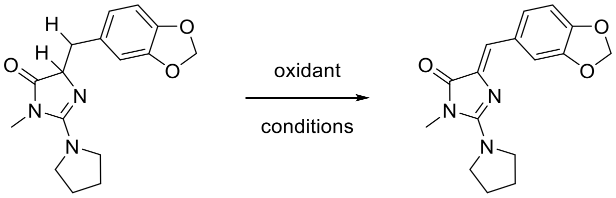

11d **12d**

Table S7. Study of dehydrogenation conditions to convert compound **11d** to compound **12d**. Unless otherwise stated, all reactions were performed under inert atmosphere (argon) for 17 h (overnight).

Entry	Oxidant (eq.)	Solvent	Temperature	Conversion % to **12d** (by LC-MS)
1	Air	$CHCl_3$	rt	10
2[a]	Air	-	rt	10
3	I_2 (1)	$CHCl_3$	rt	75
4	I_2 / TEMPO (1 / 0.1)	$CHCl_3$	rt	87
5	TEMPO (1)	$CHCl_3$	rt	100
6	DDQ (2)	$CHCl_3$	rt	0
7	$FeCl_3$ (3)	$CHCl_3$	rt	0
8	MnO_2 (50)	$CHCl_3$	rt	80
9	H_2O_2 (20)	Water	rt	0
10	H_2O_2 (20)	HFIP	rt	0

[a]**11c** was left as a solid in open air. The conversion did not change after 1 week.

2.5. Mechanistic Studies

2.5.1. Mechanistic Considerations on the Formation of Compounds 9

We propose that the formation of coelenterazine analogues **9** starts with the generation of the corresponding imidazolone **5**, which condenses with a second unit of aldehyde **1** to give the imine intermediate **9-imine**. This intermediate then progresses through an intramolecular cyclization to give coelenterazine tautomer **9'** which finally evolves towards the final coelenterazine adduct **9** (Figure S12A). This process may involve a stepwise tautomerization through the species **9''** or a direct transformation through a formal 1,5-proton shift involving imine-enamine equilibria, likely via acid-base catalysis.

No reports in the literature were found for the transformation of intermediate **9-imine** to **9'**. We assume that the observed reactivity may be due to the nucleophilic attack of the imidazolone ring upon the activated electrophilic imine moiety. The resonance form depicted in Figure S12B could explain the possible nucleophilic nature of the ring, perhaps via an intramolecular SET process.

In most cases, the MCR progressed directly to the final adduct **9** under the optimal stoichiometries of 2:1:1 for the reagents **1**, **2** and **3** respectively. However, in the case of piperonal **1k,** the MCR gave the imine intermediate **9e-imine**, and with 3,4,5-trimethoxybenzaldehyde **1i** we isolated the tautomer **9f'**. Notably, in both cases these adducts precipitated from the reaction mixture and were easily isolated by filtration under vacuum. Thus, we propose that precipitation of these intermediates during the reaction stops their progression to their corresponding coelenterazine analogues **9**. Moreover, we observed that when a 1:1:1 ratio of reactants was used with piperonal **1l** or 3,4,5-trimethoxybenzaldehyde **1i**, the isolated products in both cases were the corresponding compounds **9e-f** (in lower yields due to the suboptimal stoichiometry), and neither one of the intermediates were detected. We believe that in these cases, intermediates **9e-imine** and **9f'** were present in lower concentrations, they did not precipitate from the reaction media and were directly converted to the corresponding compounds **9e-f**. Satisfyingly, we accomplished full conversion of both intermediates **9e-imine** and **9f'** to their respective compounds **9e-f**. Noteworthy, this conversion was only observed when the intermediates were fully dissolved in the reaction mixture, further supporting the hypothesis that precipitation interrupts the reaction pathway and allows the isolation of the intermediates. (Figure S12C).

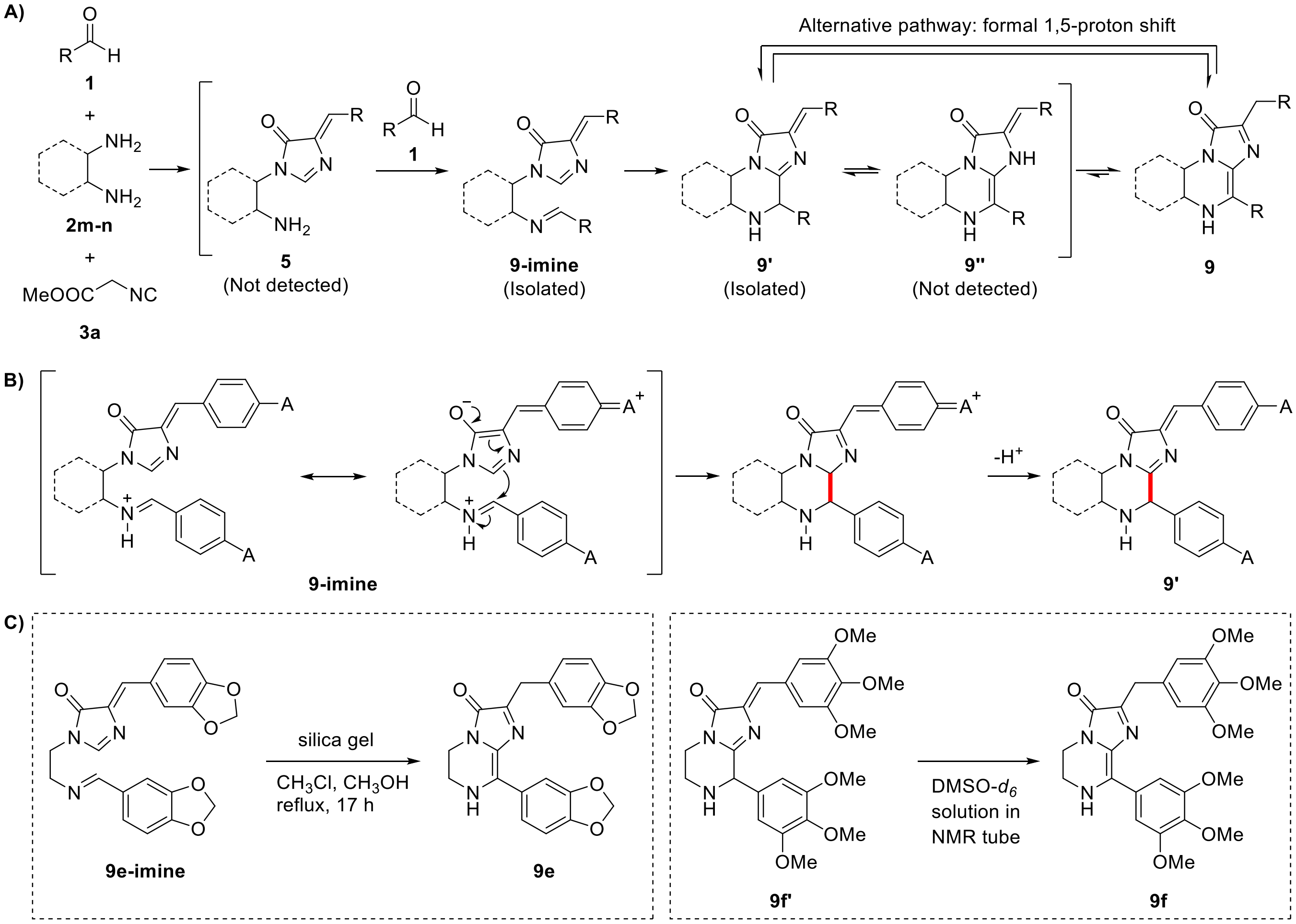

Figure S12. A) Proposed reaction mechanism of the MCR between aldehydes **1**, diamines **2m-n** and methyl isocyanoacetate **3a**. B) Putative transformation of intermediate **9-imine** to **9'**. C) Conversion of intermediates **9e-imine** and **9f'** to the corresponding coelenterazine analogues **9**.

We observed that compound **9f'** spontaneously converted to the coelenterazine analogue **9f** when left to stand in solution (Figure S13A). ^{1}H NMR analysis after 30 min of sample preparation showed the pure compound **9f'** (Figure S13B). However, the ^{1}H NMR spectrum of the same sample after standing in the DMSO-*d*₆ solution for 6 days showed complete conversion to compound **9f**, as well as some degradation by-products (Figure S13C). The spectrum was compared to one of pure compound **9f** (Figure S13D).

A)

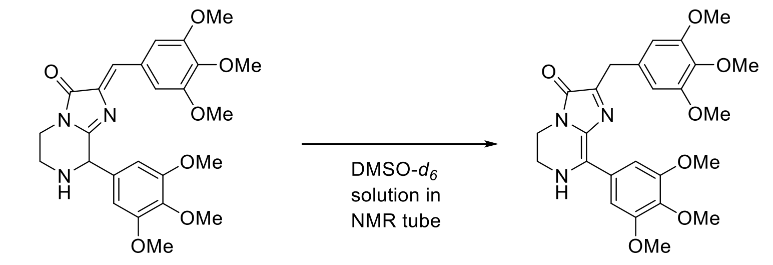

9f' **9f**

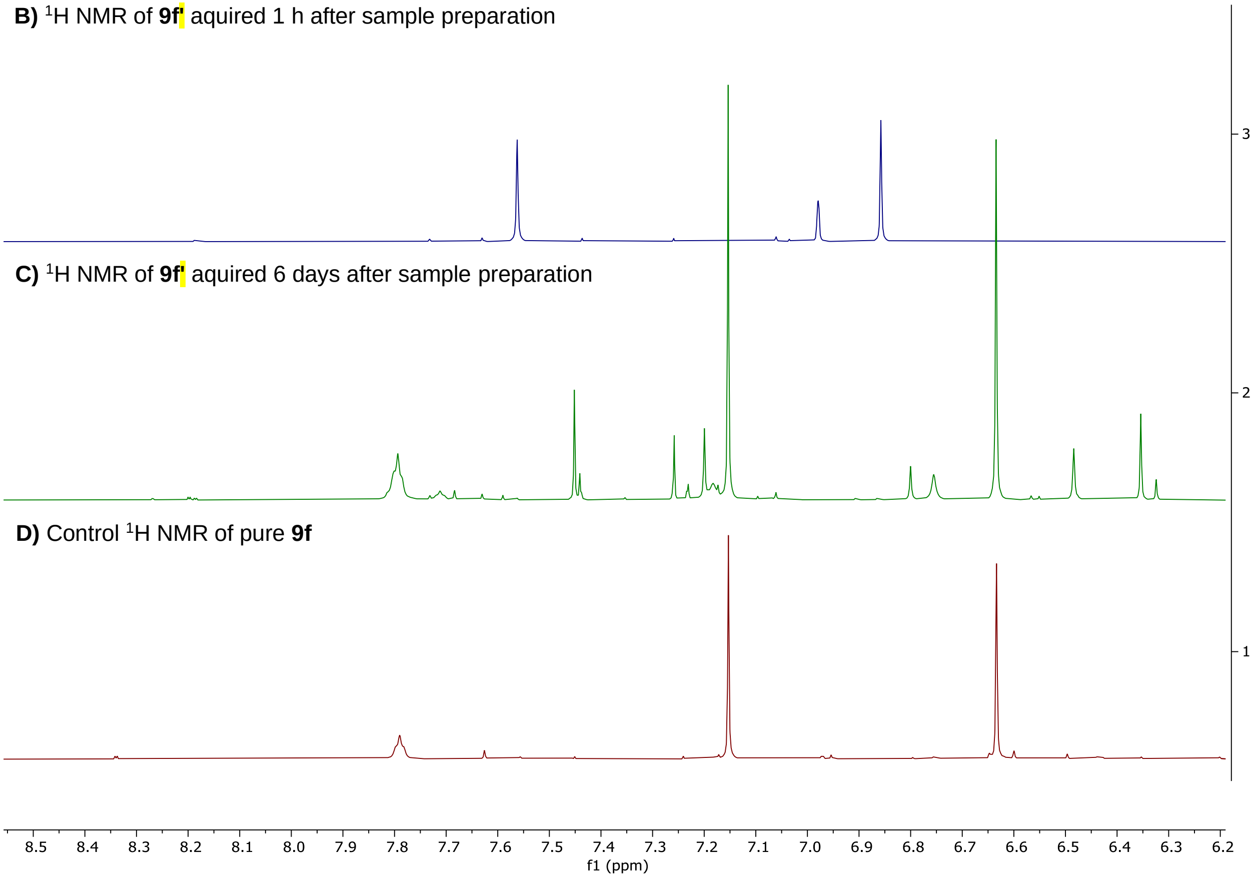

Figure S13. A) Conversion of **9f'** to **9f**. Stacked NMR spectra of B) Compound **9f'** 1 h after sample preparation; C) Compound **9f'** 6 days after sample preparation; D) Pure compound **9f**.

Preliminary computational studies on the conversion of intermediate **9f'** to compound **9f** were consistent with the empirical results. Four possible tautomers of the compounds **9** were modeled in a Spartan suite. We built the structures and minimized the energy with molecular mechanics (MMFF) and semiempirical methods (NMDO) and analyzed the equilibrium tautomers. As a model, we used a benzene ring as the aryl substituent to simplify the calculations (Figure S14). We observed a decrease in energy from intermediate **9'** to the coelenterazine analogue **9**.

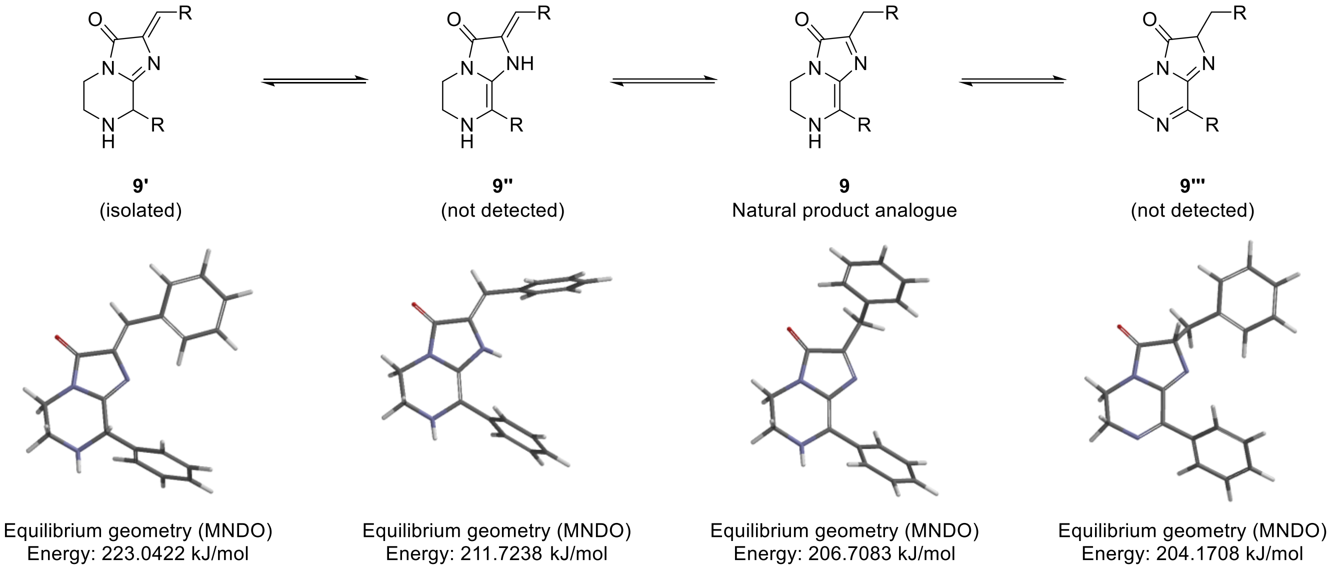

Figure S14. Computational estimated stabilities of the tautomers of coelenterazine analogues **9**.

The following experiment further supported the precipitation hypothesis, as performing the MCR under the standard conditions but in an ultrasonic bath, promoted the precipitation of bis-imine **S7** – a potential by-product of this reaction –, and reduced the conversion to the expected compound **9b** (Figure S15).

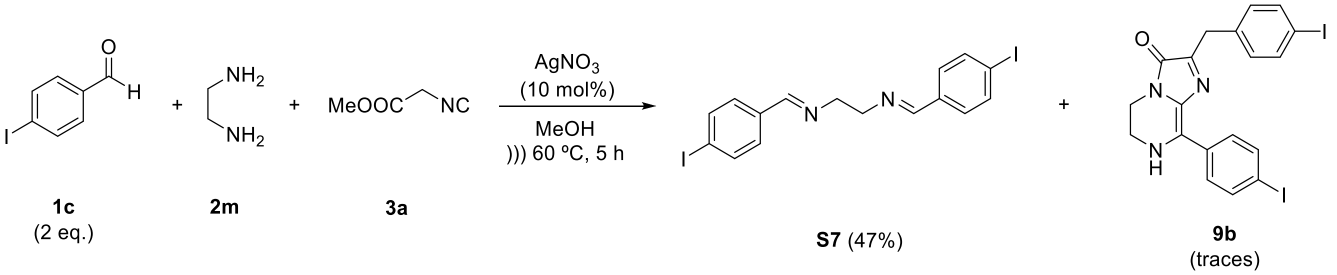

Figure S15. MCR with 4-iodobenzaldehyde **1s**, ethylenediamine **2m**, and methyl isocyanoacetate **3a** in an ultrasonic bath.

Noteworthy, a related MCR reported by Shaabani *et* al. involving carbonyls, diamines, and isocyanides and mediated by acid catalysis yields a similar scaffold. [5,6] In this case, the transformation starts with the formation of the imine **I** – in equilibrium with its cyclic aminal surrogate **II** – followed by nucleophilic isocyanide attack. The generated nitrilium cation **III** is finally trapped intramolecularly by the free amine to give the final diaza-heterocycle **IV,** which tautomerizes to the isolated adducts **V** (Figure S16A).

Presumably, if the isocyanide input was an isocyanoacetate, this MCR could in principle progress to the formation of our described coelenterazine analogues **9** after lactamization and incorporation of the second unit of aldehyde *via* a Knoevenagel condensation or vice versa. (Figure S16B). Note that there is one example of this MCR with methyl isocyanoacetate in the literature and it has been reported to give adduct **V**.[6]

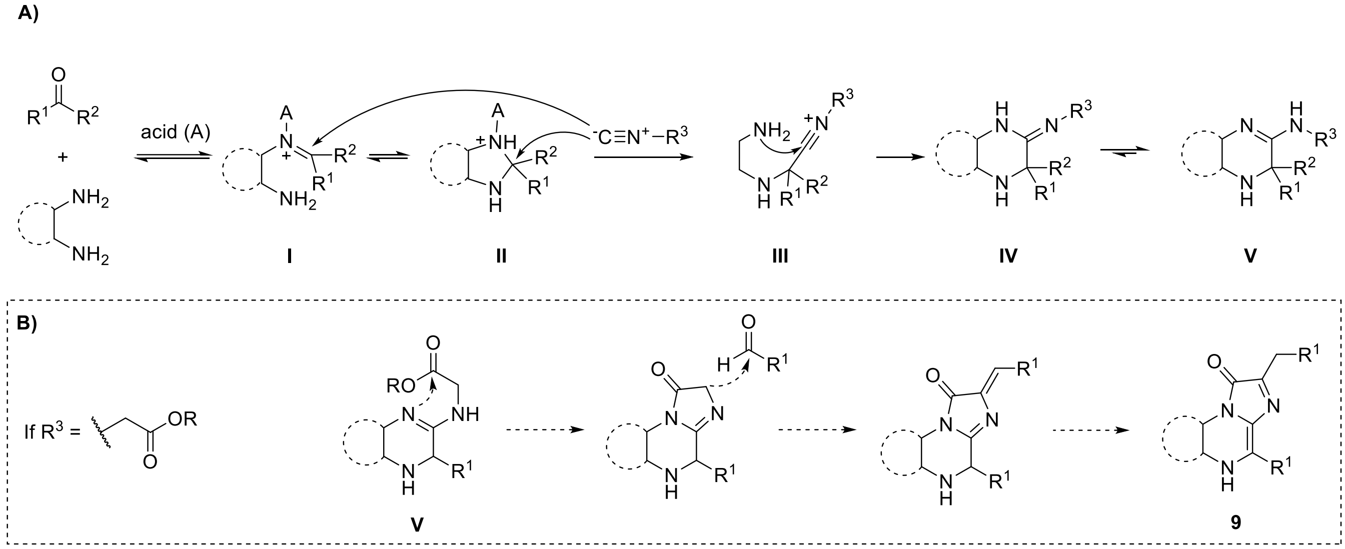

Figure S16. A) Reaction mechanism of the MCR described by Shaabani *et al*. B) Potential evolution of adduct **V** to compound **9** with isocyanoacetates.

To confirm that our MCR does not follow this mechanism, we reproduced Shabaani's conditions by performing our MCR under acid catalysis (Figure S17). We isolated an unstable material in almost quantitative yield, whose structure was assigned to aminal **S8** using ^{1}H NMR, suggesting that isocyanide **3a** does not perform the nucleophilic attack upon the aminal to follow Shabaani's pathway.

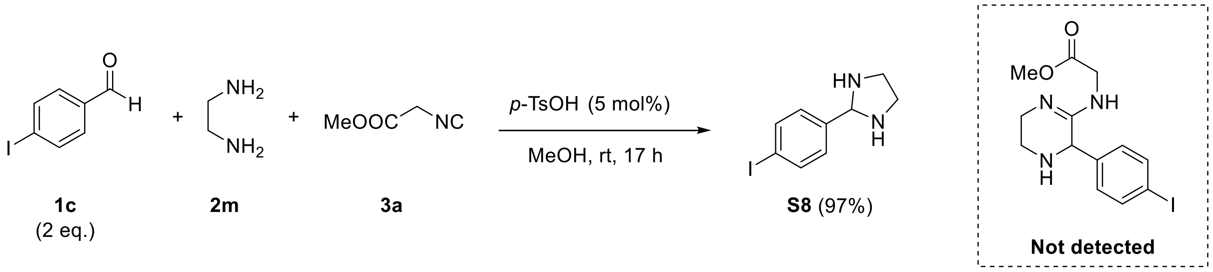

Figure S17. MCR with 4-iodobenzaldehyde **1c**, ethylenediamine **2m**, and methyl isocyanoacetate **3a**.

Moreover, when we changed the isocyanide to cyclohexyl isocyanide – which does not contain an α-acidic position and therefore cannot participate in the Knoevenagel condensation step – the MCR progressed to give the bis-imine **S9** in 86% yield (Figure S18). Thus, we confirmed that silver does not properly catalyze the MCR described by Shabaani, suggesting that the formation mechanism of our MCR differs from the one previously described.

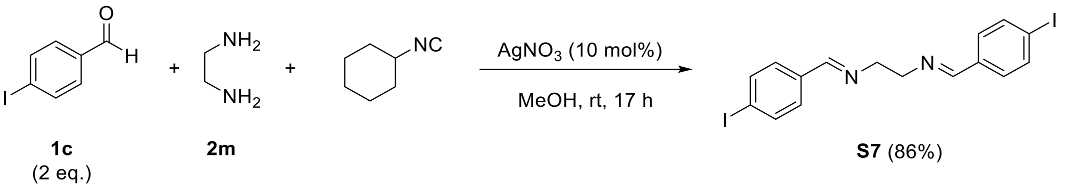

Figure S18. MCR with 4-iodobenzaldehyde **1c**, ethylenediamine **2m**, and cyclohexyl isocyanide **3a**.

2.5.2. Studies on the Addition of Nucleophiles into the Imidazolone Scaffold

(i) Mechanism of the Nucleophilic Addition

Regarding the addition of nucleophilic species into the imidazolones **5**, the productivity of transformation depended on the nucleophilicity of the added species: better nucleophiles reacted at rt while poorer nucleophiles needed thermal activation or did not react at all (see SI section 2.4.1). This study was consistent with the addition mechanism being of nucleophilic nature (Figure S19A).

Interestingly, we observed that upon addition of a nucleophile to a suspension of imidazolone **5** in CH_3CN under argon atmosphere, the mixture quickly turned to a highly intense dark violet color. Moreover, the violet color quickly faded when the reaction was opened to air. This suggested that the transformation may involve radical species (Figure S19B). A precedent in the literature reported a photocatalyzed cross dehydrogenative coupling amination in a similar system.[7] In our case, we established that the transformation was not photocatalyzed as the addition still took place when the reaction was performed in the dark, and the presence of a photosensitizer – $Ir(dFppy)_3$ – did not affect the reaction rate or conversion.

A)

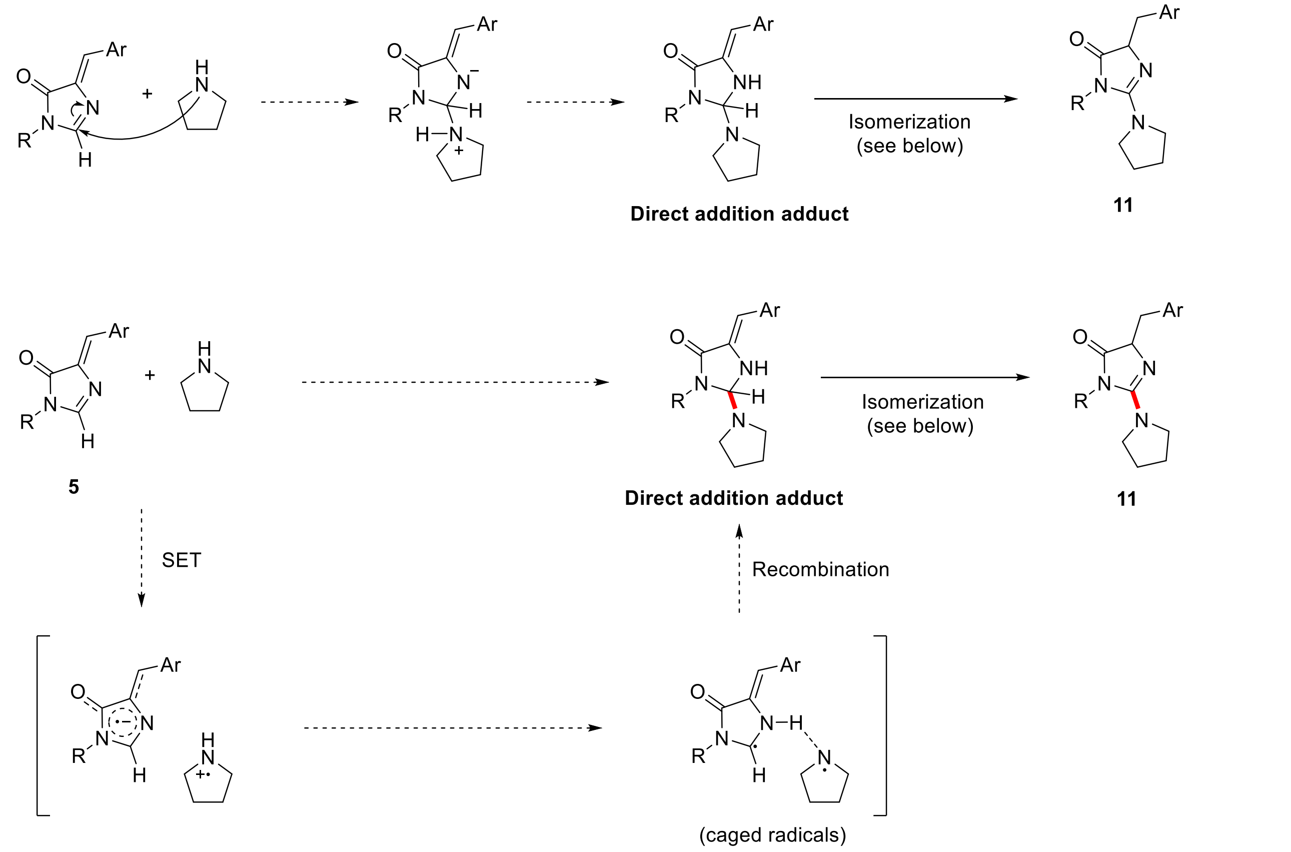

Figure S19. A) Putative ionic mechanism of the addition of nucleophiles to the imidazolone scaffold. B) Participation of complexes and radical intermediates, from SET step.

We performed a series of experiments to study whether the mechanism of the addition involves a radical process. First, considering the remarkable change in color of the reaction mixture upon addition of a nucleophile, we focused on the detection of the potential occurrence of UV-Vis absorbent charge transfer complex. UV-Vis spectra of all reactants, the product and their combination were recorded to determine whether there were light absorbing species in the reaction mixture (Figure S20). No significant differences were observed in the absorption spectra of the reactant and the reaction mixture. However, it is important to note that the quick loss of coloring after opening the reaction mixture to air – presumably due to radial quenching by atmospheric O_2 –complicated the obtention of representative data.

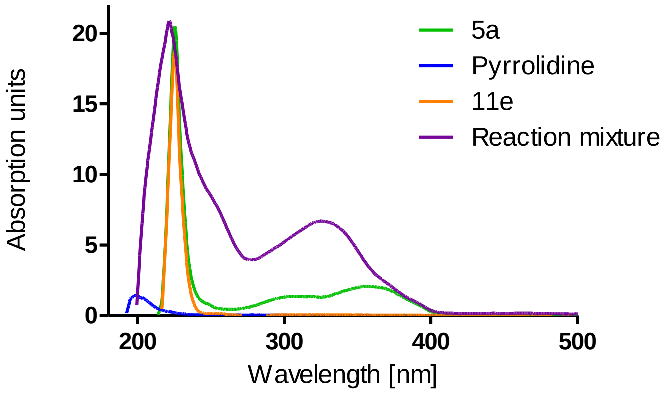

Figure S20. Absorption spectra of the components of the reaction and the reaction mixture.

Next, we focused on trapping the putative radical species, either inter- or intramolecularly. First, we performed the reaction in the presence of the stable radical TEMPO, expecting the reaction to be inhibited by trapping of the putative radical with TEMPO (Figure S21A). However, the product of the reaction was the 2-aminoimidazolone **12e**, suggesting that the addition progressed normally, and TEMPO just acted as an oxidant not trapping the putative radical. Also, we attempted to trap the putative radical species intramolecularly using the adduct **5v** as the substrate. Presumably, the styrene moiety – a known radical trap – would capture the putative radical to give the fused [5,5] adduct. However, this adduct was not detected (Figure S21B).

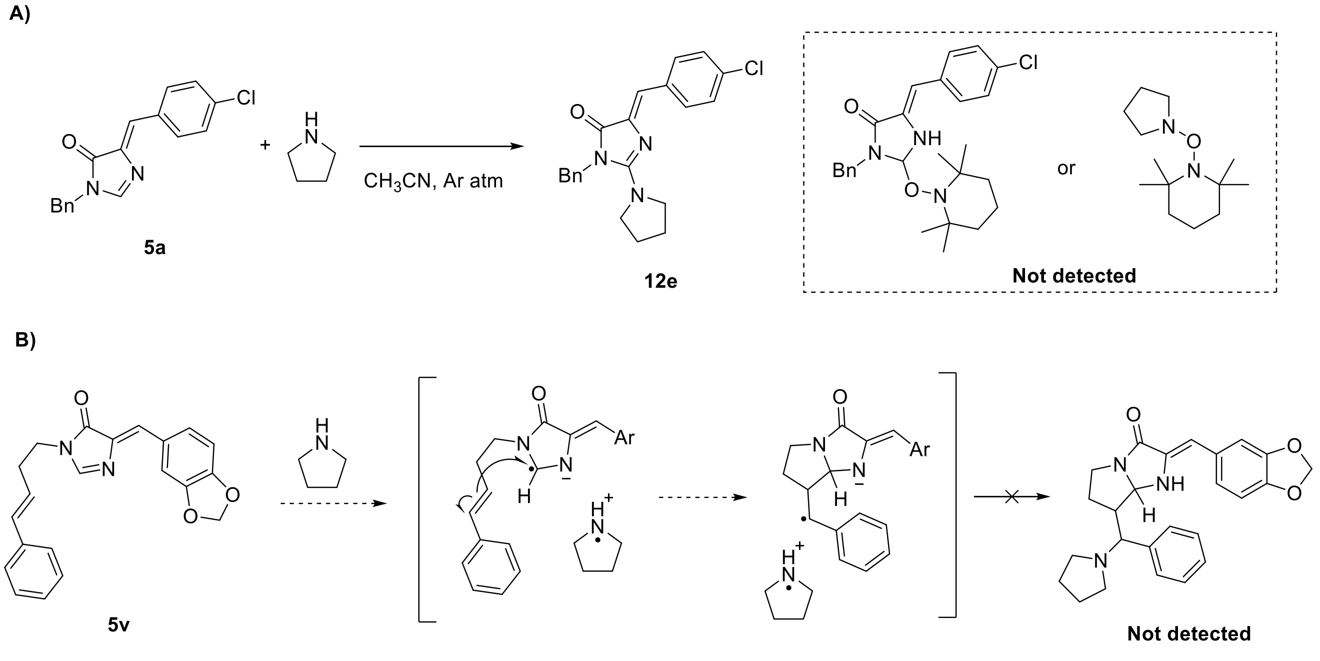

Figure S21. Mechanistic studies on the radical pathway.

Inability to capture the putative radical suggested a very short-lived radical species. Thus, we hypothesized that the use of a spin trap would generate a longer lived radical which could potentially be detected by electron paramagnetic resonance (EPR). According to the proposed hypothesis, the incorporation of DMPO into the reaction mixture would in theory result in the trapping of either the pyrrolidine-DMPO radical or the imidazolone-DMPO radical (Figure S22A). To our delight, we detected a signal, confirming the presence of a radical species in the reaction. The obtained signal was consistent with the pyrrolidine-DMPO radical, as compared with a simulated spectrum from data found in the literature (with morpholine instead of pyrrolidine, Figure S22B).[7]

Experimental procedure for acquiring the EPR spectrum: an EPR tube was purged with cycles of vacuum and argon, and was charged with CH_3CN, compound **5a** (1 eq.), pyrrolidine (10 eq.) and DMPO (20 eq.) and the EPR spectrum was recorded at room temperature. Spectrometer parameters for acquiring spectra: magnetic field, 3520 G with a sweep width of 100 G. A 5.02 mW microwave power at a frequency of 9.858 GHz was used with a modulation amplitude of 2 G and a frequency of 100 kHz. The morpholine-DMPO EPR spectrum was simulated with software from Bruker WIN-EPR system (v 2.22 Rev. 12).

A)

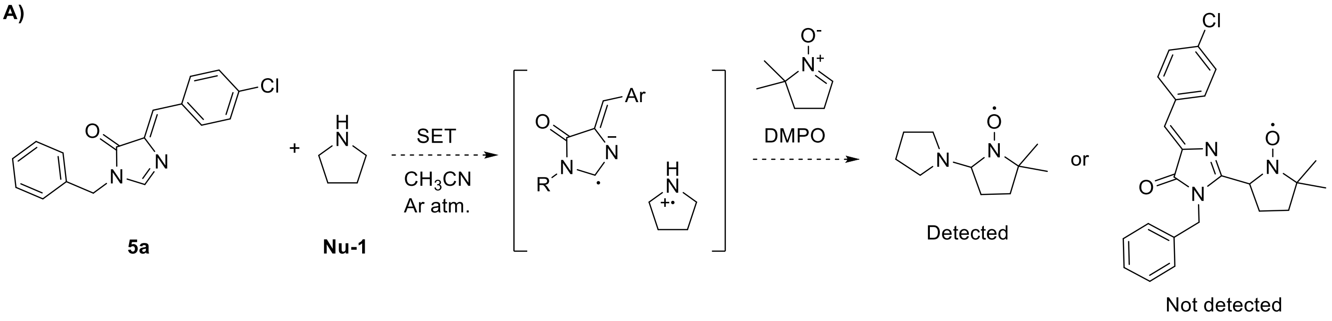

B)

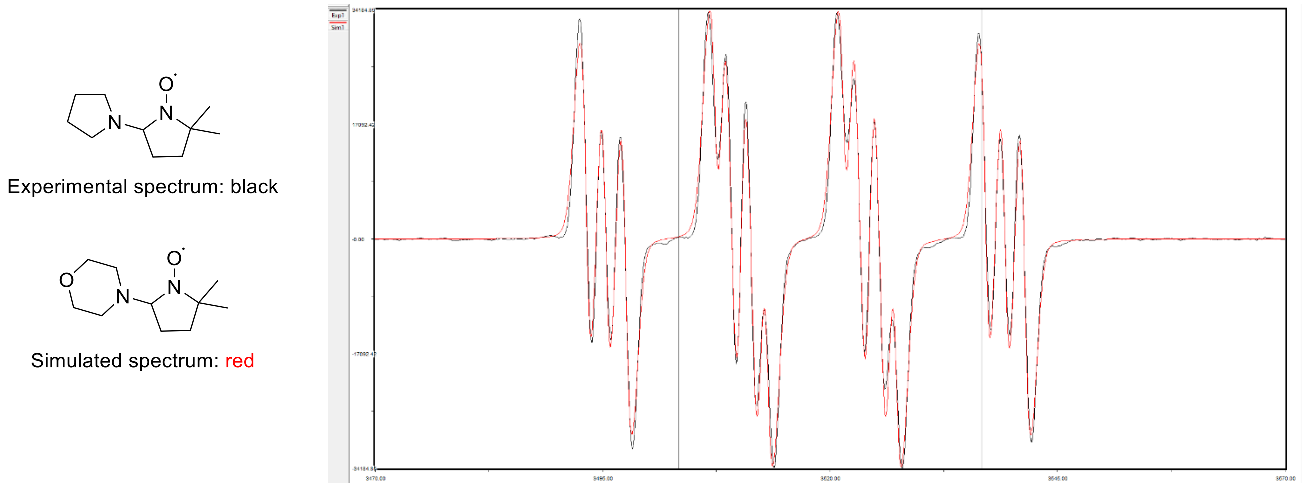

Figure S22. Trapping of the intermediate radical with DMPO.

(ii) Structural Elucidation of Compounds 11

According to the proposed addition of nucleophiles to the imidazolone **5**, the transformation should result in the direct addition adducts **A**. However, this intermediate was never detected and instead, the reaction readily afforded its tautomer adduct **11**. The structure of compounds **11** was confirmed with spectroscopic methods (see characterization data of compound **11d**). Therefore, we hypothesized that after the addition, a series of protic (acid/base) catalyzed tautomeric equilibria converts the putative intermediates **A** to the isolated compounds **11**.

We modeled the following adducts and intermediates in a Spartan suite using molecular mechanics and semiempirical method. We built the structures and minimized the energy with molecular mechanics (MMFF) and semi-empirical methods (PM3) and analyzed the equilibrium tautomers. We used the addition of pyrrolidine **2z** to adduct **5i** as the model. The computational calculations suggested a decrease in energy in each of the proposed tautomeric equilibria to reach scaffold **11** as the lowest energy structure, being consistent with the proposed hypothesis (Figure S23).

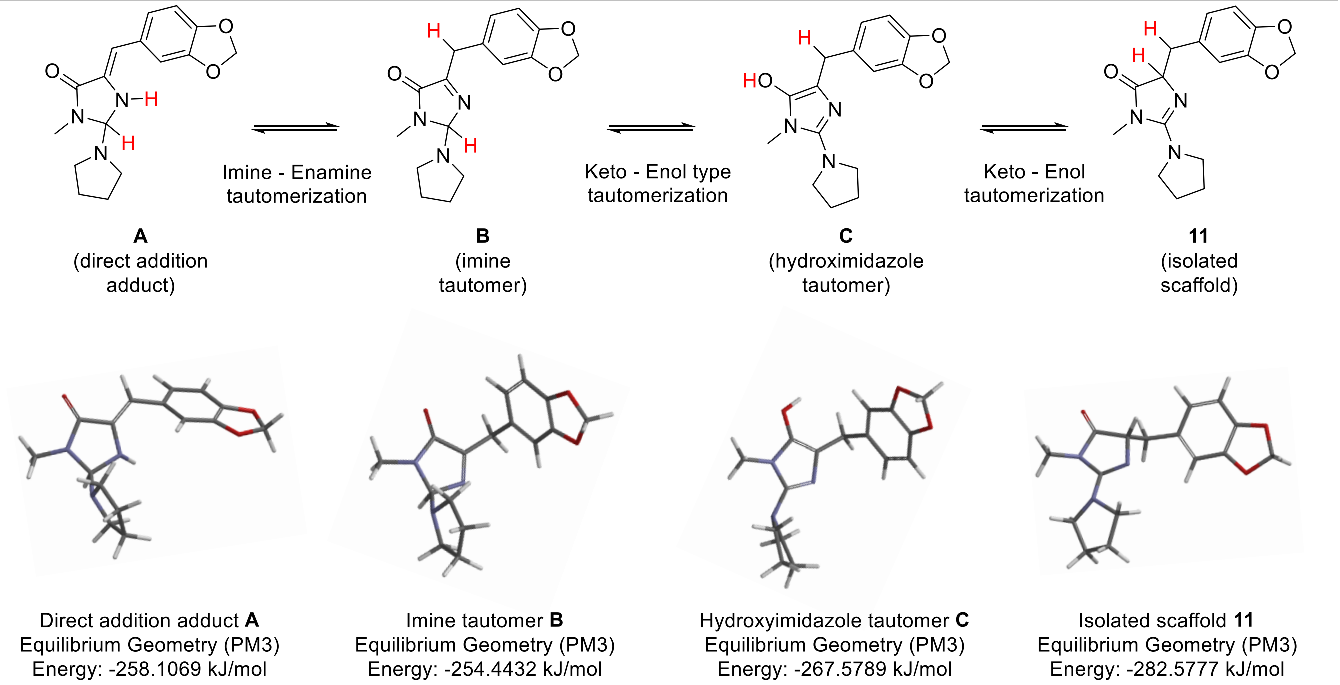

Figure S23. Tautomeric equilibria towards addition product **11** and computationally estimated stabilities.

To further study this hypothesis, we performed a series of experiments using deuterated solvents, as well as D-labelled substrate and nucleophile species. We reacted imidazolone **5a** with an excess of pyrrolidine **Nu-1** in protic and non-protic solvents. The performed experiments suggested extensive hydrogen exchange with either the solvent – depending on its nature – or the excess pyrrolidine.

We observed clear differences between protic (methanol) and aprotic (acetonitrile) solvents. In this regard, the experiments with CD_3CN (which does not undergo hydrogen exchange) and non-deuterated pyrrolidine **Nu-1** resulted in the formation of the non-deuterated compound **11e**, even if the substrate was deuterated (Figure S24A) However, the use of CD_3OD (capable of hydrogen exchange) resulted in the obtention of the deuterated adduct **11-*d*₂** from non-deuterated reagents (Figure S24B).

Lastly, we detected the triple deuterated adduct **11e-d_3** by LC-MS after reaction of deuterated imidazolone **5a-*d*** with deuterated pyrrolidine **Nu-1-*d*** in deuterated acetonitrile (CD_3CN). However, after neutral Al_2O_3 flash chromatography we observed replacement of the α deuterium for a proton and isolated adduct **11-d_2** (Figure S24C).

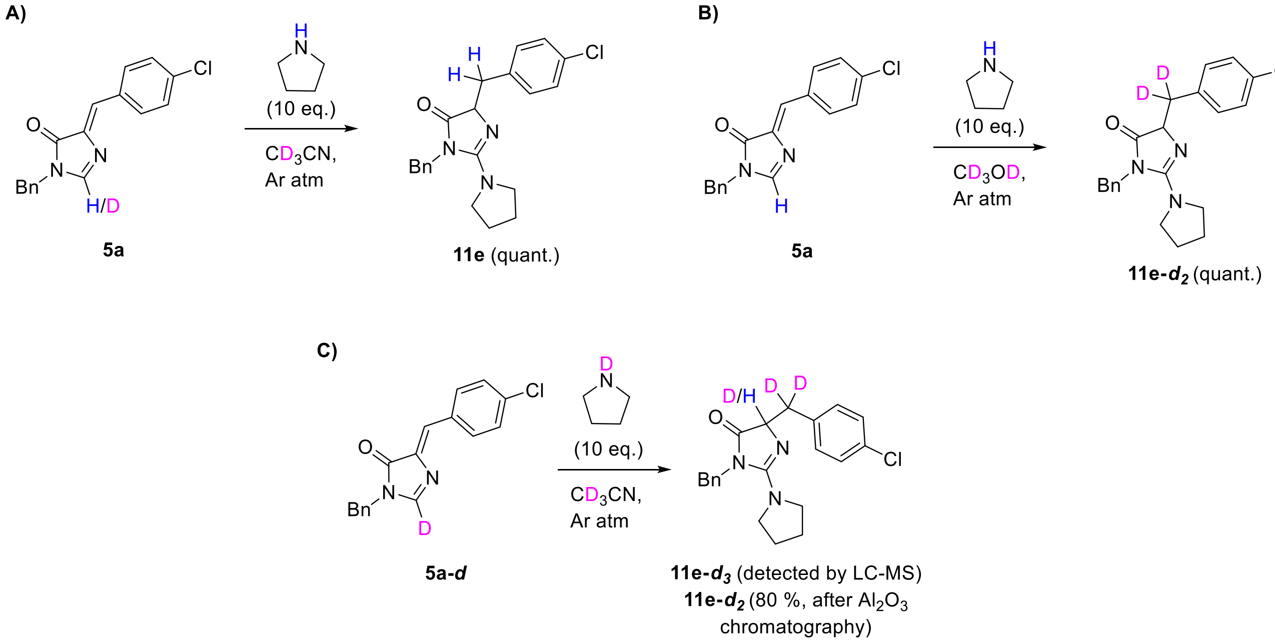

Figure S24. Deuteration experiments.

3. Synthetic Procedures and Characterization Data

3.3. General Synthetic Procedures

3.3.1. General Procedure A: Synthesis of Compounds 4-7 and 9

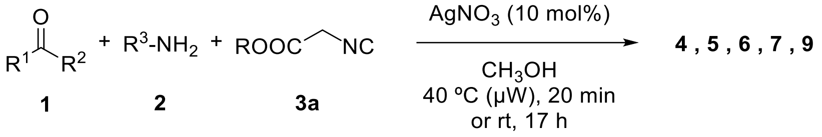

To a mixture of cabonyl **1** (1.1 or 2 eq.), methyl isocyanoacetate **3a** (1 eq.) and $AgNO_3$ (0.1 eq.) in CH_3OH (0.2 M) was added amine **2** (1.1 – 10 eq.). The reaction was either heated to 40 ºC for 20 min under microwave (μW) irradiation (sealed vial) or was stirred at room temperature (rt) for 17 h. After reaction completion (TLC or LC-MS control) the reaction mixture was filtered through Celite® washing with CH_2Cl_2 – to remove the remains of the silver catalyst – and concentrated under reduced pressure to obtain a dark oil. The crude reaction mixture was purified *via* flash chromatography using the indicated solvent system to afford the pure products **4**-**7** and **9**. In the stated cases, compounds **5** or **9** precipitated during the reaction and were collected by filtration under reduced pressure washing with cold MeOH. The solid was dissolved in CH_2Cl_2 and the resulting solution was filtered through Celite® washing with CH_2Cl_2 – to remove the remains of the silver catalyst – and concentrated under reduced pressure to give the pure product **5** or **9**.

3.3.2. General Procedure B: Sequential Synthesis of Compounds 4

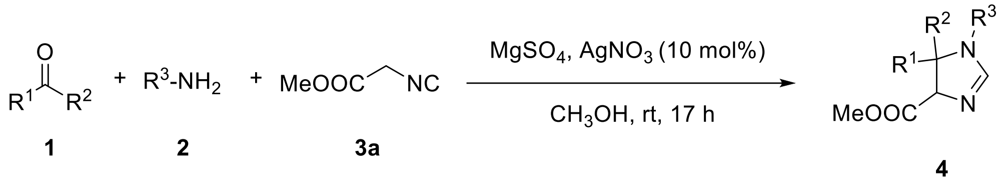

To a solution of aldehyde **1** (1.1 eq.) and amine **2** (1.1 eq.) in CH_3OH (0.2 M) was added $MgSO_4$ (1.5 eq.) and the mixture was stirred at rt for 4 h. After this time, methyl isocyanoacetate **3a** (1 eq.) and $AgNO_3$ (10 mol%) were added as specified, and the reaction was stirred overnight at rt. After reaction completion (TLC control) the mixture was filtered to remove inorganics and the solvent was evaporated under reduced pressure. The residue was absorbed onto silica and purified via silica gel flash chromatography using the indicated solvent system to afford the pure product **4**.

3.3.3. General Procedure C: Synthesis of Compounds 8

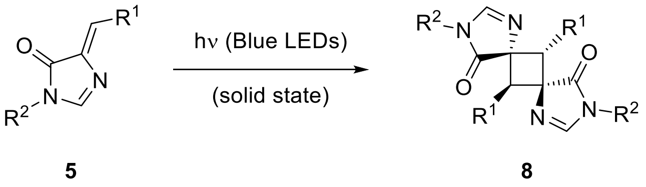

Compound **5** was placed as a solid – in a vial or round bottom flask – under irradiation with a Blue LEDs strip for 12 – 17 h (until analysis by TLC or LC-MS suggested full conversion) to give the pure product **8** without the need for purification.

3.3.4. General Procedure D: Synthesis of Compounds 12a-c

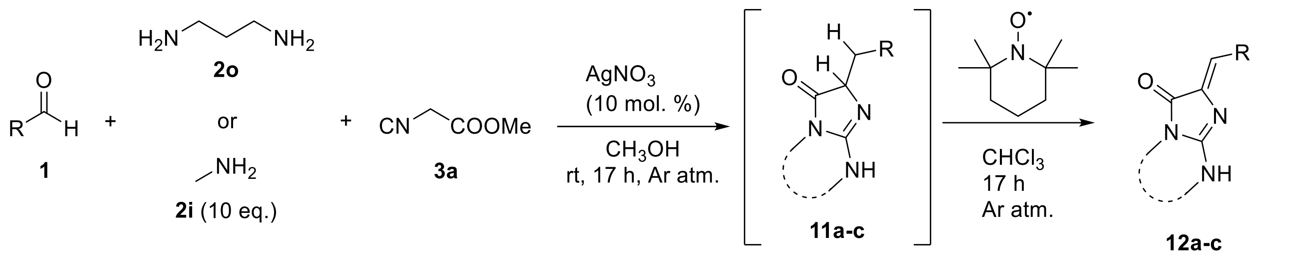

(i) To a mixture of aldehyde **1** (1.1 eq.), methyl isocyanoacetate **3a** (1 eq.), and $AgNO_3$ (0.1 eq.) in CH_3OH (0.2 M) under argon atmosphere was added 1,3-diaminopropane **2o** (1.1 eq.) or a 9.8 M solution of methylamine **2i** in MeOH (10 eq.). The reaction was stirred at room temperature (rt) for 17 h. After reaction completion (LC-MS control), the reaction mixture was filtered through a pad of Celite® washing with CH_2Cl_2. The filtrate was extracted with a 0.5 N aqueous HCl solution (3x). The aqueous layers were combined, basified to pH = 9 and extracted with CH_2Cl_2 (3x). The organic layers were combined, washed with saturated brine, dried over Na_2SO_4, filtered, and concentrated under reduced pressure to give intermediates **11a-c**. Intermediates **11a-c** contained traces of compounds **12a-c** and were used in the next step without further purification.

(ii) To a solution of intermediate **11a-c** (1 eq.) in dry CH_3Cl (0.1 M) under argon atmosphere was added TEMPO (2 eq.) and the reaction was stirred at room temperature (rt) for 17 h. The pure products **12a-c** precipitated from the reaction mixture and were collected by filtration under reduced pressure washing with cold $CHCl_3$.

3.3.5. General Procedure E: Synthesis of Compounds 12d-h and 13

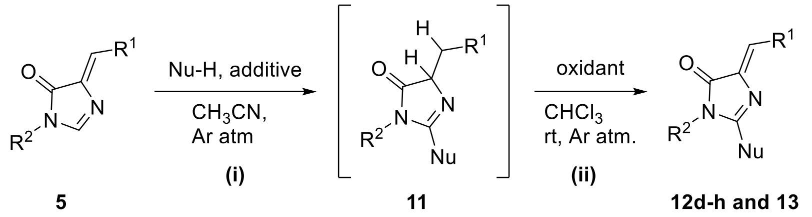

(i) To a suspension of **5** (1 eq.) in CH_3CN (0.1 M) under argon atmosphere was added the corresponding nucleophile (1.1 – 10 eq.) and an additive when specified. Upon addition the mixture turned to a dark purple solution. The reaction was stirred at the indicated temperature for 17 h. After reaction completion (TLC or LC-MS control) the solvent was evaporated under reduced pressure and the excess nucleophile was removed by evaporation under reduced pressure or *via* aqueous work-up, to give the corresponding intermediate **11**. In most cases, intermediates **11** were only checked by LC-MS and directly subjected to the dehydrogenation protocol. Compounds **11d-e** and **11j** were characterized without the need for further purification.

(ii) To a solution of intermediate **11** (1 eq.) in dry CH_3Cl (0.1 M) under argon atmosphere was added the indicated oxidant and the reaction was stirred at room temperature until analysis by LC-MS suggested consumption of starting material. The appropriate work-up was performed according to the oxidant used in each case. The crude product was purified *via* neutral Al_2O_3 flash chromatography using the indicated solvent system to afford the pure products **12d-h** and **13**.

Experimental procedures according to the oxidant used:

TEMPO: TEMPO (2 eq.) was added to a solution of **5** in $CHCl_3$ under argon atmosphere. After reaction completion (LC-MS control), the reaction was diluted with CH_2Cl_2 and washed with a 1:1 mixture of a saturated aqueous solution of $Na_2S_2O_3$ and a saturated aqueous solution of Na_2CO_3. The organic layer was washed with saturated brine, dried over Na_2SO_4, filtered, and concentrated under reduced pressure to afford the crude product.

I_2 / TEMPO: A solution of I_2 (0.9 eq.) in $CHCl_3$ and TEMPO (0.1 eq.) were added to a solution of **5** in $CHCl_3$ under argon atmosphere. After reaction completion (LC-MS control), the reaction was diluted with CH_2Cl_2 and washed with a 1:1 mixture of a saturated aqueous solution of $Na_2S_2O_3$ and a saturated aqueous solution of Na_2CO_3. The organic layer was washed with saturated brine, dried over Na_2SO_4, filtered, and concentrated under reduced pressure to afford the crude product.

MnO_2: MnO_2 (50 eq.) was added to a solution of **5** in $CHCl_3$ under argon atmosphere. After reaction completion (LC-MS control), the reaction was filtered through a pad of Celite® washing with CH_2Cl_2. The filtrate was concentrated under reduced pressure to afford the crude product.

3.3.6. General Procedure F: Synthesis of Compounds 14

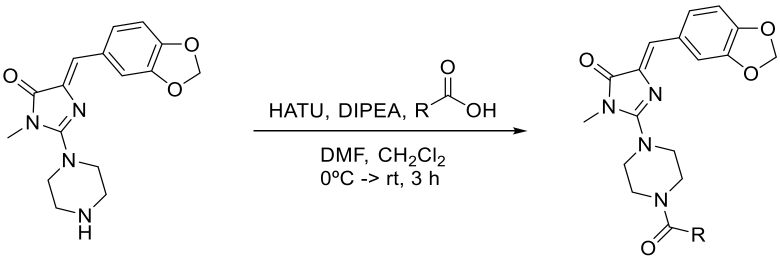

12j **14**

A Schlenk flask in an ice bath was charged with DMF (0.1 M), the corresponding carboxylic acid (1.2 eq.), HATU (1.2 eq.), and DIPEA (4 eq.). The reaction was purged with cycles of vacuum and argon, was allowed to warm up to rt and was stirred for 1 h. After this time the reaction was cooled to 0 ºC and a solution of **12j** (1 eq.) in CH_2Cl_2 (0.2 M) was added dropwise. The reaction was allowed to warm up to rt and was stirred for 2 h. The reaction was diluted with CH_2Cl_2 and washed with a saturated aqueous Na_2CO_3 solution and saturated brine. The organic layer was dried over Na_2SO_4, filtered, and concentrated under reduced pressure. The crude product was purified *via* neutral Al_2O_3 flash chromatography using the indicated solvent system to afford the pure product **14**.

3.3.7. Special Case I: Synthesis of Compound 10

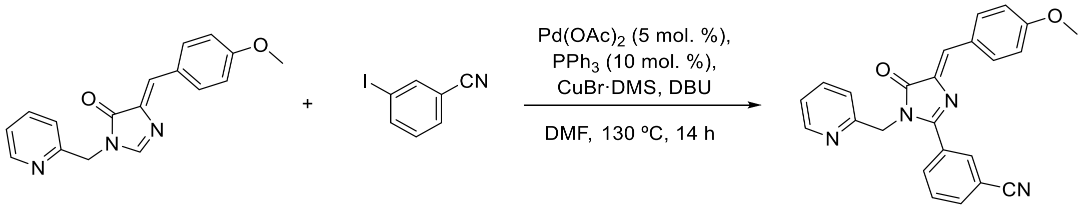

5e **10**

The title compound **10** was synthesized following a protocol described in the literature.[8]

A Schlenk tube was sequentially charged with **5e** (1 eq.), $Pd(OAc)_2$ (0.05 eq.), PPh_3 (0.1 eq.), DBU (1 eq.), CuBr·DMS (1 eq.), and a solution of 3-iodobenzonitrile (1 eq.) in DMF (0.05 M). The reaction was purged with cycles of argon and vacuum (3x) and was heated to 130 ºC and stirred for 14 h. After reaction completion (TLC control), the reaction mixture diluted with CH_2Cl_2 was filtered through Celite® washing with CH_2Cl_2. The filtrate was washed with a 10% aqueous NH_3 solution (3x) and saturated brine. The organic layer was dried over Na_2SO_4, filtered, and concentrated under reduced pressure. The crude product was purified *via* silica gel flash chromatography (EtOAc/hexane gradient from 0:100 to 50:50 v/v) to afford the pure product **10**.

3.3.8. Special Case II: Synthesis of Compound 12j

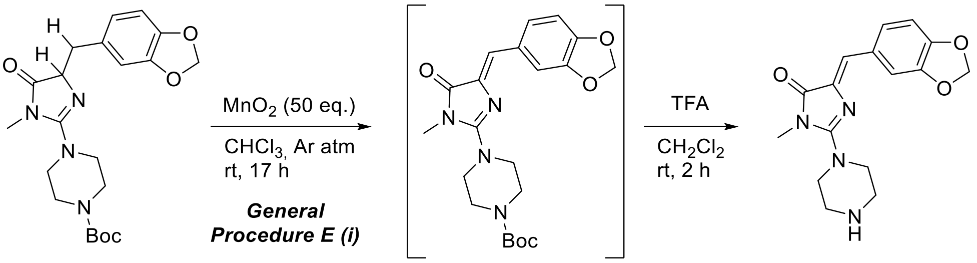

11j **Boc-12j (not isolated)** **12j**

To a solution of **11j** (1 eq.) in dry $CHCl_3$ (0.1 M) was added MnO_2 (50 eq.) and the reaction was stirred at rt for 17 h. After reaction completion (LC-MS control), the inorganics were filtered with a pad of Celite® washing with CH_2Cl_2. The filtrate was concentrated under reduced pressure to give the intermediate **Boc-12j** which was not isolated. The crude product was dissolved in CH_2Cl_2 (0.1 M) and was added TFA (100 eq.) and the reaction was stirred at rt for 2h, until analysis by TLC suggested consumption of starting material. The reaction mixture was partitioned between CH_2Cl_2 and water. The aqueous layer (containing the ionized title compound **12j**) was basified by the portion wise addition of solid Na_2CO_3 until pH = 11 and was extracted with CH_2Cl_2 (3x). The combined organic layers were washed with saturated brine, dried over Na_2SO_4, filtered, and concentrated under reduced pressure to afford the title compound **12j**.

3.4. Synthesis and Characterization Data of Non-Commercial Starting Materials

4-((*tert*-Butyldimethylsilyl)oxy)benzaldehyde (1g)

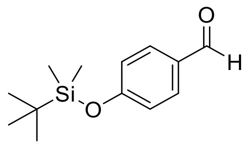

To a mixture of **1f** (617 mg, 5.05 mmol, 1 eq.) and triethylamine (2.1 mL, 15.16 mmol, 3 eq.) in dry CH_2Cl_2 (10 mL) at 0 ºC, was added dropwise a solution of *tert*-butyldimethylchlorosilane (965 mg, 6.40 mmol, 1.3 eq.) in CH_2Cl_2 (5 mL) under nitrogen atmosphere. The reaction mixture was left to warm to room temperature and stirred for 2 h, until analysis by TLC suggested consumption of starting material. The reaction was quenched with water and the aqueous layer was extracted with CH_2Cl_2. The organic layer was washed with saturated $NaHCO_3$ aqueous solution and saturated brine, dried over Na_2SO_4, filtered, and concentrated under reduced pressure to afford the title compound **1g** as a pale-yellow oil (1.2 g, 96%), which was used in the MCR without further purification. **^{1}H NMR** (400 MHz, $CDCl_3$) δ 9.88 (s, 1H), 7.79 (d, *J* = 8.3 Hz, 2H), 6.94 (d, *J* = 8.4 Hz, 2H), 0.99 (s, 9H), 0.25 (s, 6H). NMR data are in agreement with those previously reported in literature.[9]

***tert*-Butyl 3-formyl-1*H*-indole-1-carboxylate (1n)**

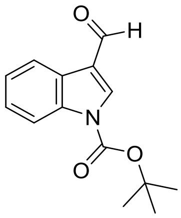

To a suspension of **1m** (850 mg, 5.86 mmol, 1 eq.) and triethylamine (1.0 mL, 7.19 mmol, 1.23 eq.) in dry CH_2Cl_2 (20 mL) at 0 ºC, was added Boc_2O (1.4 g, 6.30 mmol, 1.1 eq.) and DMAP (36 mg, 0.29 mmol, 0.05 eq.). The reaction was allowed to warm up to room temperature and left to stir for 1 h. The reaction was diluted with CH_2Cl_2 (100 mL) and washed sequentially with saturated $NaHCO_3$ aqueous solution (3 x 30 mL), saturated NH_4Cl aqueous solution (3 x 30 mL), and saturated brine (30 mL), dried over Na_2SO_4, filtered, and concentrated under reduced pressure to afford the title compound **1n** as a white solid (1.4 g, 91%), which was used in the MCR without further purification. **^{1}H NMR** (400 MHz, $CDCl_3$) δ 10.10 (s, 1H), 8.29 (d, *J* = 7.6 Hz, 1H), 8.23 (s, 1H), 8.15 (d, *J* = 8.1 Hz, 1H), 7.44 – 7.34 (m, 2H), 1.71 (s, 9H). NMR data are in agreement with those previously reported in literature.[10]

1-Tosyl-1*H*-indole-3-carboxaldehyde (1o)

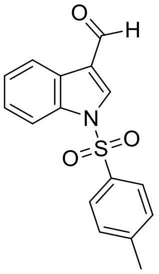

To a suspension of **1m** (624 mg, 4.30 mmol, 1 eq.) and triethylamine (1.8 mL, 12.90 mmol, 3 eq.) in dry CH_2Cl_2 (15 mL) at 0 ºC, was added portion-wise p-toluenesulfonyl chloride (1.23 g, 6.45 mmol, 1.5 eq.). The reaction was allowed to warm to room temperature and left to stir overnight. The reaction mixture was diluted with CH_2Cl_2 and washed with saturated aqueous $NaHCO_3$ and 5N aqueous HCl. The crude product was purified *via* silica gel flash chromatography (EtOAc/hexane gradient form 0:100 to 10:90 v/v) to afford the title compound **1o** as a white solid (0.97 g, 75%). **^{1}H NMR** (400 MHz, $CDCl_3$) δ 10.10 (s, 1H), 8.25 (d, *J* = 7.9 Hz, 1H), 8.23 (s, 1H), 7.95 (d, *J* = 8.3 Hz, 1H), 7.85 (d, *J* = 8.2 Hz, 2H), 7.44 – 7.33 (m, 2H), 7.30 (d, *J* = 8.1 Hz, 2H), 2.38 (s, 3H). NMR data are in agreement to those previously reported in literature.[10]

2-(Allyloxy)benzaldehyde (1h)

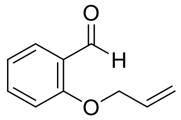

A mixture of salicylaldehyde (0.5 mL, 4.71 mmol, 1 eq.) and K_2CO_3 (1.02 g, 5.29 mmol, 1.1 eq.) in dry CH_3CN (25 mL) was heated to 80 ºC. Then was added dropwise allyl bromide (0.45 mL, 7.20 mmol, 1.5 eq.) and the reaction was stirred at 80 ºC for 5 h. After reaction completion (TLC control) the solvent was evaporated under reduced pressure and the residue was dissolved in CH_2Cl_2 (100 mL) and washed with saturated $NaHCO_3$ aqueous solution (3 x 30 mL) and saturated brine (30 mL). The organic layer was dried over Na_2SO_4, filtered, and concentrated under reduced pressure to afford the title compound **1h** as an orange oil (800 mg, 94%), which was used in the MCR without further purification. **^{1}H NMR** (400 MHz, $CDCl_3$) δ 10.54 (d, *J* = 0.8 Hz, 1H), 7.84 (dd, *J* = 7.7, 1.9 Hz, 1H), 7.53 (td, *J* = 7.3, 1.9 Hz, 1H), 7.03 (td, *J* = 7.5, 1.0 Hz, 1H), 6.98 (dd, *J* = 8.5, 0.9 Hz, 1H), 6.15 – 6.01 (m, 1H), 5.46 (dq, *J* = 17.3, 1.6 Hz, 1H), 5.34 (dq, *J* = 10.6, 1.4 Hz, 1H), 4.66 (dt, *J* = 5.2, 1.6 Hz, 2H). NMR data are in agreement with those previously reported in literature.[11]

Benzylamine-*d₂* (2a-*d₂*)

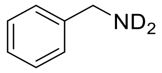

2a (1.1 mL, 10 mmol), $CDCl_3$ (5 mL) and CD_3OD (5 mL) were charged into a 25 mL round bottom flask. The solution was purged with cycles of vacuum and argon (3x) and was stirred under argon atmosphere for 3 h. The solvent was evaporated under reduced pressure. Fresh solvents were charged into the flask and the process was repeated three times. After final evaporation, the title compound **2a-*d*$_2$** was obtained as a clear liquid in quantitative yields. **^{1}H NMR** (400 MHz, $CDCl_3$) δ 7.37 – 7.29 (m, 4H), 7.28 – 7.22 (m, 1H), 3.86 (s, 2H). NMR data are in agreement with those previously reported in literature.[12]

(*E*)-4-phenylbut-3-en-1-amine (2w)

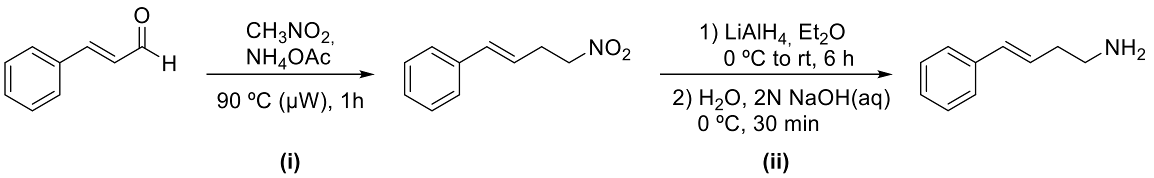

(i) Following a procedure reported in the literature,[13] a 10 – 20 mL Biotage® MW vial was charged with *trans*-cinnamaldehyde (650 µL, 5.2 mmol, 1 eq.), nitromethane (15.5 mL, 288.6 mmol, 56 eq.) and ammonium acetate (199 mg, 2.6 mmol, 0.5 eq.). The vial was sealed, and the mixture was irradiated at 90 ºC for 1 h in a microwave reactor. After reaction completion (TLC control) the reaction mixture was concentrated under reduced pressure. The residue disoved in CH_2Cl_2 and washed with water and saturated brine. The organic layer was dried over Na_2SO_4, filtered, and concentrated under reduced pressure. The crude product was purified *via* silica gel flash chromatography (EtOAc/hexane gradient form 0:100 to 5:95 v/v) to afford (*E*)-(4-nitrobut-1-en-1-yl)benzene as an off white solid (650 mg, 72%). **^{1}H NMR** (400 MHz, $CDCl_3$) δ 7.78 (t, *J* = 11.7 Hz, 1H), 7.55 – 7.49 (m, 2H), 7.44 – 7.36 (m, 3H), 7.24 (d, *J* = 13.1 Hz, 1H), 7.15 (d, *J* = 15.5 Hz, 1H), 6.86 (dd, *J* = 15.5, 11.6 Hz, 1H). NMR data are in agreement with those previously reported in literature.[13]

(ii) To a 0 ºC suspension of $LiAlH_4$ (156 mg, 4.1 mmol, 1.5 eq.) in Et_2O (12 mL) under argon atmosphere was added a solution of (*E*)-(4-nitrobut-1-en-1-yl)benzene (48 mg, 2.7 mmol, 1 eq.) in Et_2O (8 mL). The reaction was left to warm to rt and was stirred for 6 h. After reaction completion (TLC control) the mixture was cooled to 0 ºC and was sequentially added water (0.2 mL), 2 N aqueous NaOH (0.2 mL) and water (0.5 mL) and was stirred at rt for 30 min. After this time the mixture was diluted with EtOAc and filtered washing with EtOAc. The filtrate was dried over Na_2SO_4, filtered, and concentrated under reduced pressure to afford the title compound **2w** as an orange oil (260 mg, 65%), which was used in the MCR without further purification. **^{1}H NMR** (400 MHz, DMSO-*d*$_6$) δ 7.38 (d, *J* = 7.2 Hz, 2H), 7.30 (t, *J* = 7.5 Hz, 2H), 7.19 (t, *J* = 7.2 Hz, 1H), 6.41 (d, *J* = 16.0 Hz, 1H), 6.30 (dt, *J* = 15.9, 6.7 Hz, 1H), 2.66 (t, *J* = 6.9 Hz, 2H), 2.25 (q, *J* = 6.8 Hz, 2H). NMR data are in agreement with those previously reported in literature.[14]

N^{α}-Fmoc-L-lysine allyl ester hydrochoride (2x)

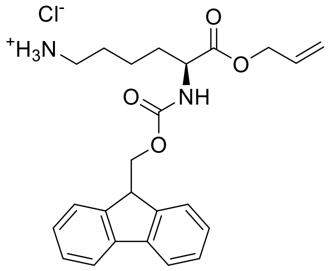

To a suspension of N^{α}-Fmoc-Lysine-OH (1.67 g, 4.5 mmol, 1 eq.) in allyl alcohol (20 mL) was slowly added $SOCl_2$ (0.85 mL, 11.6 mmol, 2.6 eq.). The resulting clear solution was stirred at reflux temperature for 5 h. After reaction completion (TLC control) the reaction mixture was concentrated under reduced pressure. To the residue was added Et_2O and a white precipitate was formed. The solid was washed several times with Et_2O and dried under reduced pressure to give the title compound **2x** as a white solid (1.9 g, 90%), which was used in the MCR without further purification. **^{1}H NMR** (400 MHz, $CDCl_3$) δ 8.08 (br s, 3H), 7.72 (d, *J* = 7.5 Hz, 2H), 7.64 – 7.46 (m, 2H), 7.36 (t, *J* = 7.4 Hz, 2H), 7.31 – 7.25 (m, 2H), 5.93 – 5.74 (m, 2H), 5.32 – 5.24 (m, 1H), 5.23 – 5.16 (m, 1H), 4.62 – 4.50 (m, 2H), 4.38 – 4.28 (m, 2H), 4.23 – 3.99 (m, 2H), 3.00 (s, 2H), 1.94 – 1.62 (m, 4H), 1.55 – 1.39 (m, 2H). NMR data are in agreement with those previously reported in literature.[15]

Pyrrolidine-1-*d* (Nu-1-*d*)

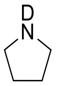

Nu-1 (2 mL, 24.2 mmol), $CDCl_3$ (5 mL) and CD_3OD (5 mL) were charged into a 25 mL round bottom flask. The solution was purged with cycle of vacuum and argon three times and was stirred under argon atmosphere for 3 h. The solvent was evaporated under reduced pressure. Fresh solvents were charged into the flask and the process was repeated 3 times. After final evaporation, the title compound **Nu-1-*d*** was obtained as a clear liquid (1.7 mL, 84%) **^{1}H NMR** (400 MHz, $CDCl_3$) δ 2.88 – 2.79 (m, 4H), 1.71 – 1.63 (m, 4H). NMR data are in agreement with those previously reported in literature for non-deuterated pyrrolidine without the NH signal at 2.23 ppm.[16]

3.5. Characterization Data of Isolated Compounds

3.5.1. Compounds 4

Methyl 1-benzyl-5-(4-chlorophenyl)-4,5-dihydro-1*H*-imidazole-4-carboxylate (4a)

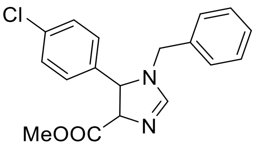

Following *General Procedure B*, compound **4a** was obtained as a pale brown oil (500 mg, 76%) from **1a** (309 mg, 2.2 mmol), **2a** (235 μL, 2.2 mmol), **3a** (181 μL, 2 mmol), Mg_2SO_4 (361 mg, 3 mmol) and $AgNO_3$ (34 mg, 0.2 mmol) in CH_3OH (10 mL) after flash chromatography purification ($MeOH/CH_2Cl_2$ gradient from 0:100 to 10:90 v/v). Compound **4a** was obtained as an 85 : 15 mixture of *trans* : *cis* diastereomers. NMR data reported of the major diastereomer. **^{1}H NMR** (400 MHz, $CDCl_3$) δ 7.36 – 7.28 (m, 5H), 7.25 – 7.21 (m, 2H), 7.12 (dd, *J* = 4.7, 2.0 Hz, 2H), 7.09 (d, *J* = 1.6 Hz, 1H), 4.66 (d, *J* = 9.5 Hz, 1H), 4.52 (dd, *J* = 9.5, 1.9 Hz, 1H), 4.40 (d, *J* = 15.0 Hz, 1H), 3.95 (d, *J* = 14.9 Hz, 1H), 3.75 (s, 3H). **^{13}C NMR** (101 MHz, $CDCl_3$) δ 171.90, 156.85, 138.16, 135.31, 134.12, 129.21, 128.89, 128.71, 128.06, 128.02, 78.05, 64.88, 52.57, 49.27. **HRMS:** *m/z* calcd. for $C_{18}H_{18}ClN_2O_2^+$ [M+H]$^+$: 329.1051; found: 329.1052.

Methyl 1-benzyl-5,5-dimethyl-4,5-dihydro-1H-imidazole-4-carboxylate (4aa)

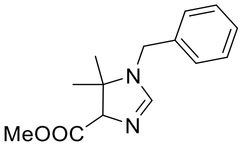

Following *General Procedure A* under μW irradiation, compound **4aa** was obtained as a brown oil (73 mg, 30%) from **1aa** (81 μL, 1.1 mmol), **2a** (120 μL, 2.2 mmol), **3a** (91 μL, 1 mmol) and $AgNO_3$ (17 mg, 0.1 mmol) in CH_3OH (5 mL) after flash chromatography purification (MeOH/EtOAc gradient from 0:100 to 10:90 v/v). **^{1}H NMR** (400 MHz, $CDCl_3$) δ 7.38 – 7.30 (m, 3H), 7.29 (d, *J* = 1.4 Hz, 1H), 7.27 – 7.24 (m, 2H), 4.47 (d, *J* = 1.4 Hz, 1H), 4.32 (d, *J* = 15.1 Hz, 1H), 4.26 (d, *J* = 15.1 Hz, 1H), 3.76 (s, 3H), 1.38 (s, 3H), 1.13 (s, 3H). NMR data are in agreement with those previously reported in literature.[17]

Compound **4aa** was isolated from the same reaction mixture as compound **5aa**.

Methyl 1-benzyl-1,3-diazaspiro[4.4]non-2-ene-4-carboxylate (4ab)

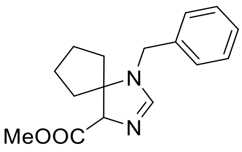

Following *General Procedure A* under μW irradiation, compound **4ab** was obtained as a brown oil (300 mg, 55%) from **1ab** (195 μL, 2.2 mmol), **2a** (240 μL, 2.2 mmol), **3a** (181 μL, 2 mmol) and $AgNO_3$ (34 mg, 0.2 mmol) in CH_3OH (10 mL) after flash chromatography purification (MeOH/EtOAc gradient from 0:100 to 10:90 v/v). **^{1}H NMR** (400 MHz, DMSO-*d$_6$*) δ 7.37 – 7.23 (m, 5H), 7.12 (d, *J* = 1.4 Hz, 1H), 4.40 (d, *J* = 16.4 Hz, 1H), 4.33 (d, *J* = 1.5 Hz, 1H), 4.30 (d, *J* = 16.5 Hz, 1H), 3.63 (s, 3H), 1.79 – 1.70 (m, 1H), 1.64 – 1.51 (m, 5H), 1.42 – 1.30 (m, 2H). **^{13}C NMR** (101 MHz, DMSO-*d$_6$*) δ 171.34, 157.74, 139.80, 128.42, 127.03, 126.87, 78.57, 73.81, 51.31, 44.65, 36.34, 30.26, 22.96, 22.17. **HRMS:** *m/z* calcd. for $C_{16}H_{21}N_2O_2^+$ [M+H]$^+$: 273.1598; found: 273.1598.

Compound **4ab** was isolated from the same reaction mixture as compound **5ab**.

Methyl (1r,3r,5r,7r)-3'-benzyl-3',5'-dihydrospiro[adamantane-2,4'-imidazole]-5'-carboxylate (4ac)

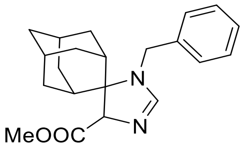

Following *General Procedure A* under μW irradiation, compound **4ac** was obtained as a pale brown oil (270 mg, 40%) from **1ac** (330 mg, 2.2 mmol), **2a** (240 μL, 2.2 mmol), **3a** (181 μL, 2 mmol) and $AgNO_3$ (34 mg, 0.2 mmol) in CH_3OH (10 mL) after flash chromatography purification (EtOAc/hexane gradient from 0:100 to 100:0 v/v). **^{1}H NMR** (400 MHz, $CDCl_3$) δ 7.48 – 7.42 (m, 2H), 7.40 – 7.33 (m, 2H), 7.31 – 7.27 (m, 1H), 6.93 (d, *J* = 1.1 Hz, 1H), 4.85 (s, 2H), 4.79 (d, *J* = 1.1 Hz, 1H), 3.73 (s, 3H), 2.25 – 2.14 (m, 4H), 2.02 – 1.96 (m, 1H), 1.95 – 1.89 (m, 1H), 1.86 – 1.81 (m, 1H), 1.80 – 1.77 (m, 1H), 1.76 – 1.69 (m, 4H), 1.66 – 1.60 (m, 1H), 1.53 – 1.46 (m, 1H). **^{13}C NMR** (101 MHz, $CDCl_3$) δ 171.50, 160.40, 139.30, 128.59, 127.33, 126.96, 76.37, 71.99, 51.86, 51.74, 38.79, 37.44, 36.26, 35.15, 33.59, 32.76, 31.21, 26.80, 26.60. **HRMS:** *m/z* calcd. for $C_{21}H_{27}N_2O_2^+$ [M+H]$^+$: 339.2067; found: 339.2078.

Compound **4ac** was isolated from the same reaction mixture as compound **5ac**.

3.5.2. Compounds 5

(*Z*)-3-Benzyl-5-(4-chlorobenzylidene)-3,5-dihydro-4*H*-imidazol-4-one (5a)

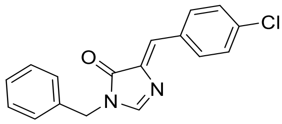

Following *General Procedure A* under μW irradiation, compound **5a** was obtained as a golden colored solid (0.95 g, 47%) from **1a** (1.02 g, 7.3 mmol), **2a** (793 μL, 7.3 mmol), **3a** (600 μL, 6.6 mmol) and $AgNO_3$ (112 mg, 0.66 mmol) in CH_3OH (20 mL, 0.33 M). The product precipitated from the reaction mixture and was isolated by filtration under reduced pressure. **^{1}H NMR** (400 MHz, DMSO-*d6*) δ 8.44 (d, *J* = 1.7 Hz, 1H), 8.29 (d, *J* = 8.6 Hz, 2H), 7.56 (d, *J* = 8.7 Hz, 2H), 7.46 – 7.38 (m, 2H), 7.38 – 7.30 (m, 3H), 7.22 (d, *J* = 1.7 Hz, 1H), 4.84 (s, 2H). **^{13}C NMR** (101 MHz, DMSO-d_6) δ 169.55, 156.66, 139.41, 137.14, 135.70, 134.33, 133.09, 129.35, 129.22, 128.17, 127.90, 127.42, 44.66. **HRMS**: *m/z* calcd. for $C_{17}H_{14}ClN_2O^+$ [M+H]$^+$: 297.0789; found: 297.0797.

(*Z*)-3-Benzyl-5-(4-chlorobenzylidene)-3,5-dihydro-4*H*-imidazol-4-one-2-*d* (5a-*d*)

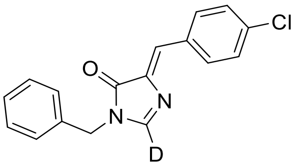

Following *General Procedure A* under μW irradiation, compound **5a** was obtained as a golden colored solid (850 mg, 47%) from **1a** (0.93 mg, 6.6 mmol), **2a-*d₂*** (734 μL, 6.6 mmol), **3a** (544 μL, 6 mmol) and $AgNO_3$ (102 mg, 0.6 mmol) in CD_3OD (20 mL, 0.3 M). The product precipitated from the reaction mixture and was isolated by filtration under reduced pressure. **^{1}H NMR** (500 MHz, $CDCl_3$) δ 8.09 – 8.05 (m, 2H), 7.41 – 7.38 (m, 2H), 7.38 – 7.31 (m, 3H), 7.30 – 7.27 (m, 2H), 7.20 (s, 1H), 4.77 (s, 2H). **^{13}C NMR** (126 MHz, $CDCl_3$) δ 168.49, 152.11 (t), 137.69, 135.84, 134.36, 132.63, 131.23, 128.24, 128.11, 128.08, 127.34, 126.77, 44.00. **HRMS:** *m/z* calcd. for $C_{17}H_{13}DClN_2O^+$ [M+H]$^+$: 298.0852; found: 298.0851.

(*Z*)-3-Benzyl-5-(4-chlorobenzylidene)-3,5-dihydro-4*H*-imidazol-4-one (5b)

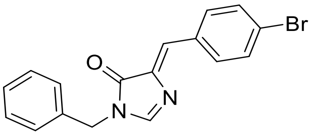

Following *General Procedure A* at rt, compound **5b** was obtained as a golden colored solid (1.05 g, 46%) from **1b** (1.35 mg, 7.3 mmol), **2a** (794 μL, 7.3 mmol), **3a** (600 μL, 6.6 mmol) and $AgNO_3$ (112 mg, 0.66 mmol) in CH_3OH (20 mL, 0.3 M). The product precipitated from the reaction mixture and was isolated by filtration under reduced pressure. **^{1}H NMR** (400 MHz, $CDCl_3$) δ 8.03 – 7.97 (m, 2H), 7.71 (d, *J* = 1.7 Hz, 1H), 7.58 – 7.53 (m, 2H), 7.41 – 7.27 (m, 5H), 7.19 (d, *J* = 1.7 Hz, 1H), 4.77 (s, 2H). **^{13}C NMR** (101 MHz, $CDCl_3$)

δ 169.65, 153.59, 138.99, 135.51, 133.95, 132.78, 132.22, 129.48, 129.27, 128.51, 127.94, 125.63, 45.19. **HRMS:** *m/z* calcd. for $C_{17}H_{14}BrN_2O^+$ [M+H]$^+$: 341.0284; found: 341.0291.

(*Z*)-3-Benzyl-5-(4-methoxybenzylidene)-3,5-dihydro-4*H*-imidazol-4-one (5c)

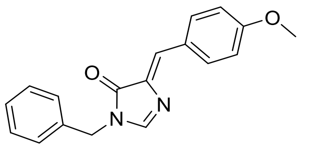

Following *General Procedure A* at rt, compound **5c** was obtained as a yellow solid (730 mg, 50%) from **1e** (670 μL, 5.5 mmol), **2a** (600 μL, 5.5 mmol), **3a** (454 μL, 5 mmol) and $AgNO_3$ (85 mg, 0.5 mmol) in CH_3OH (15 mL, 0.3 M). The product precipitated from the reaction mixture and was isolated by filtration under reduced pressure. **^{1}H NMR** (400 MHz, $CDCl_3$) δ 8.08 – 8.01 (m, 2H), 7.59 (d, *J* = 1.7 Hz, 1H), 7.32 – 7.24 (m, 3H), 7.23 (d, *J* = 1.8 Hz, 1H), 7.22 – 7.18 (m, 2H), 6.92 – 6.84 (m, 2H), 4.71 (s, 2H), 3.79 (s, 3H). NMR data are in agreement with those previously reported in literature.[8]

(*Z*)-3-(3-iodobenzyl)-5-(4-methoxybenzylidene)-3,5-dihydro-4*H*-imidazol-4-one (5d)

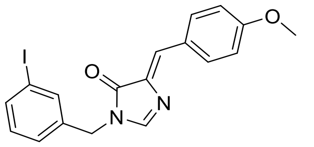

Following *General Procedure A* under μW irradiation, compound **5d** was obtained as a dark yellow solid (565 mg, 45%) from **1e** (402 μL, 3.3 mmol), **2d** (440 μL, 3.3 mmol), **3a** (272 μL, 3 mmol) and $AgNO_3$ (51 mg, 0.3 mmol) in CH_3OH (10 mL, 0.3 M) after flash chromatography purification (EtOAc/hexane gradient from 0:100 to 40:60 v/v). **^{1}H NMR** (400 MHz, DMSO-*d$_6$*) δ 8.30 (d, *J* = 1.6 Hz, 1H), 8.26 – 8.17 (m, 2H), 7.71 (t, *J* = 1.8 Hz, 1H), 7.70 – 7.64 (m, 1H), 7.36 – 7.29 (m, 1H), 7.17 (t, *J* = 7.8 Hz, 1H), 7.13 (d, *J* = 1.7 Hz, 1H), 7.08 – 7.01 (m, 2H), 4.75 (s, 2H), 3.83 (s, 3H). **^{13}C NMR** (101 MHz, DMSO-*d$_6$*) δ 169.57, 161.86, 154.53, 139.92, 136.89, 136.86, 136.51, 134.92, 131.37, 129.42, 127.39, 126.86, 114.92, 95.52, 55.89, 43.88. **HRMS:** *m/z* calcd. for $C_{18}H_{16}IN_2O_2^+$ [M+H]$^+$: 419.0251; found: 419.0252.

(*Z*)-5-(4-Methoxybenzylidene)-3-(pyridin-2-ylmethyl)-3,5-dihydro-4*H*-imidazol-4-one (5e)

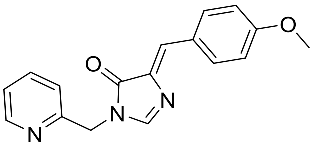

Following *General Procedure A* under μW irradiation, compound **5e** was obtained as a yellow solid (370 mg, 42%) from **1e** (400 μL, 3.3 mmol), **2f** (310 μL, 3.3 mmol), **3a** (272 μL, 3 mmol) and $AgNO_3$ (51 mg, 0.3 mmol) in CH_3OH (15 mL). The product precipitated from the reaction mixture and was isolated by filtration under reduced pressure and used in the next step without further purification. **^{1}H NMR** (500 MHz, DMSO-*d$_6$*) δ 8.51 (d, *J* = 4.8 Hz, 1H), 8.27 (d, *J* = 1.8 Hz, 1H), 8.22 (d, *J* = 8.9 Hz, 2H), 7.80 (td, *J* = 7.6, 1.8 Hz, 1H), 7.35 (d, *J* = 7.7 Hz, 1H), 7.34 – 7.29 (m, 1H), 7.11 (d, *J* = 1.8 Hz, 1H), 7.04 (d, *J* = 9.0 Hz, 2H), 4.90 (s, 2H), 3.83 (s, 3H). NMR data are in agreement to those previously reported in literature.[8]

(*R,Z*)-5-(4-Methoxybenzylidene)-3-(1-phenylethyl)-3,5-dihydro-4*H*-imidazol-4-one (5f)

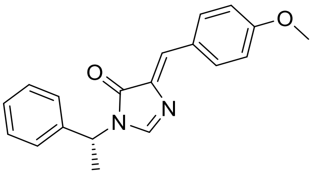

Following *General Procedure A* at rt, compound **5f** was obtained as a yellow solid (140 mg, 45%) from **1e** (134 μL, 1.1 mmol) **2e** (140 μL, 1.1 mmol), **3a** (91 μL, 1 mmol) and $AgNO_3$ (17 mg, 0.1 mmol) in CH_3OH (5 mL) after flash chromatography purification (EtOAc/hexane gradient from 0:100 to 20:80 v/v). **^{1}H NMR** (400 MHz, DMSO-*d$_6$*) δ 8.45 (d, *J* = 1.7 Hz, 1H), 8.21 (d, *J* = 9.1 Hz, 2H), 7.42 – 7.24 (m, 5H), 7.12 (d, *J* = 1.6 Hz, 1H), 7.04 (d, *J* = 9.1 Hz, 2H), 5.24 (q, *J* = 7.2 Hz, 1H), 3.83 (s, 3H), 1.73 (d, *J* = 7.3 Hz, 3H).

^{13}**C NMR** (101 MHz, DMSO-*d6*) δ 169.13, 161.78, 153.03, 141.85, 137.46, 134.85, 129.17, 129.07, 128.06, 126.98, 126.68, 114.91, 55.87, 50.95, 20.13. **HRMS:** *m/z* calcd. for $C_{19}H_{19}N_2O_2^+$ [M+H]$^+$: 307.1441; found: 307.1440.

(*Z*)-5-(2-(allyloxy)benzylidene)-3-(4-methylbenzyl)-3,5-dihydro-4*H*-imidazol-4-one (5g)

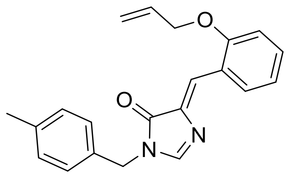

Following *General Procedure A* under μW irradiation, compound **5g** was obtained as a yellow solid (150 mg, 43%) from **1h** (178 mg, 1.1 mmol), **2b** (140 μL, 1.1 mmol), **3a** (91 μL, 1 mmol) and $AgNO_3$ (17 mg, 0.1 mmol) in CH_3OH (5 mL) after flash chromatography purification (EtOAc/hexane gradient from 0:100 to 30:70 v/v). ^{1}**H NMR** (400 MHz, $CDCl_3$) δ 8.68 (dd, *J* = 7.9, 1.7 Hz, 1H), 7.90 (d, *J* = 1.7 Hz, 1H), 7.67 (d, *J* = 1.8 Hz, 1H), 7.34 (ddd, *J* = 8.7, 7.3, 1.8 Hz, 1H), 7.22 – 7.14 (m, 5H), 7.05 – 6.98 (m, 1H), 6.89 (dd, *J* = 8.4, 1.1 Hz, 1H), 6.08 (ddt, *J* = 17.3, 10.4, 5.2 Hz, 1H), 5.43 (dq, *J* = 17.3, 1.6 Hz, 1H), 5.31 (dq, *J* = 10.2, 1.2 Hz, 1H), 4.73 (s, 2H), 4.63 (dt, *J* = 5.2, 1.6 Hz, 2H), 2.34 (s, 3H). ^{13}**C NMR** (101 MHz, $CDCl_3$) δ 169.64, 158.41, 152.61, 138.05, 138.03, 133.26, 132.85, 132.65, 132.28, 129.72, 127.83, 125.11, 123.12, 121.06, 117.91, 112.03, 69.28, 44.77, 21.14. **HRMS:** *m/z* calcd. for $C_{21}H_{21}N_2O_2^+$ [M+H]$^+$: 333.1598; found: 333.1600.

(*Z*)-3-Benzyl-5-(4-(benzyloxy)benzylidene)-3,5-dihydro-4*H*-imidazol-4-one (5h)

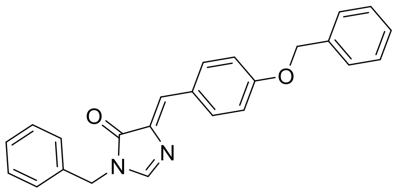

Following *General Procedure A* at rt, compound **5h** was obtained as a yellow solid (171 mg, 44%) from **1d** (234 mg, 1.1 mmol), **2a** (120 μL, 1.1 mmol), **3a** (91 μL, 1 mmol) and $AgNO_3$ (17 mg, 0.1 mmol) in CH_3OH (5 mL) after flash chromatography purification (EtOAc/hexane gradient from 0:100 to 20:80 v/v). ^{1}**H NMR** (400 MHz, DMSO-*d6*) δ 8.34 (d, *J* = 1.6 Hz, 1H), 8.26 (d, *J* = 8.9 Hz, 2H), 7.55 – 7.48 (m, 2H), 7.48 – 7.38 (m, 5H), 7.18 (d, *J* = 1.5 Hz, 1H), 7.16 (d, *J* = 8.8 Hz, 2H), 5.23 (s, 2H), 4.83 (s, 2H). ^{13}**C NMR** (101 MHz, DMSO-*d6*) δ 169.57, 160.88, 154.79, 137.37, 137.12, 137.05, 134.86, 129.20, 129.10, 128.96, 128.47, 128.32, 128.11, 127.87, 127.12, 115.70, 69.90, 44.55. **HRMS:** *m/z* calcd. for $C_2H_{21}N_2O_2^+$ [M+H]$^+$: 369.1598; found: 369.1601.

Crystals suitable for single X-ray diffraction were grown by slow diffusion of Et_2O into a CH_2Cl_2 solution of the pure compound **5h** through layering.

(*Z*)-5-(Benzo[d][1,3]dioxol-5-ylmethylene)-3-methyl-3,5-dihydro-4*H*-imidazol-4-one (5i)

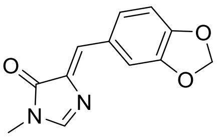

Following *General Procedure A* under μW irradiation, compound **5i** was obtained as a brown solid (2.05 g, 87%) from **1k** (1.65 g, 11 mmol), **2i** (9.8 M solution in MeOH, 1.1 mL, 11 mmol), **3a** (0.91 mL, 10 mmol) and $AgNO_3$ (170 mg, 1 mmol) in CH_3OH (20 mL, 0.5 M). The product precipitated from the reaction mixture and was isolated by filtration under reduced pressure. ^{1}**H NMR** (400 MHz, $CDCl_3$) δ 7.90 (d, *J* = 1.7 Hz, 1H), 7.62 (d, *J* = 1.7 Hz, 1H), 7.37 (dd, *J* = 8.1, 1.7 Hz, 1H), 7.07 (d, *J* = 1.7 Hz, 1H), 6.78 (d, *J* = 8.1 Hz, 1H), 5.95 (s, 2H), 3.16 (s, 3H). ^{13}**C NMR** (101 MHz, $CDCl_3$) δ 170.15, 152.82, 150.12, 148.27, 136.78, 130.57, 129.16, 128.36, 111.40, 108.59, 101.66, 27.74. **HRMS:** *m/z* calcd. for $C_{12}H_{11}N_2O_3^+$ [M+H]$^+$: 231.0764; found: 231.0762.

(*Z*)-5-(4-(Dimethylamino)benzylidene)-3-methyl-3,5-dihydro-4*H*-imidazol-4-one (5j)

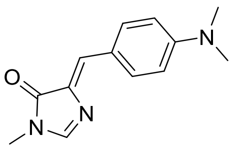

Following *General Procedure A* under μW irradiation, compound **5j** was obtained as an orange solid (0.92 g, 76%) from **1j** (789 mg, 5.8 mmol), **2i** (9.8 M solution in MeOH, 594 μL, 5.8 mmol), **3a** (480 μL, 5.3 mmol) and $AgNO_3$ (95 mg, 0.5 mmol) in CH_3OH (15 mL, 0.35 M). The product precipitated from the reaction mixture and was isolated by filtration under reduced pressure. **^{1}H NMR** (400 MHz, $CDCl_3$) δ 8.08 – 8.02 (m, 2H), 7.60 (d, *J* = 1.6 Hz, 1H), 7.19 (d, *J* = 1.6 Hz, 1H), 6.72 – 6.66 (m, 2H), 3.22 (s, 3H), 3.05 (s, 6H). **^{13}C NMR** (101 MHz, $CDCl_3$) δ 170.16, 151.95, 150.49, 134.73, 134.33, 132.03, 121.84, 111.73, 40.04, 27.68. **HRMS:** *m/z* calcd. for $C_{13}H_{16}N_3O^+$ $[M+H]^+$: 230.1288; found: 230.1286.

(*Z*)-3-benzyl-5-(thiophen-2-ylmethylene)-3,5-dihydro-4*H*-imidazol-4-one (5k)

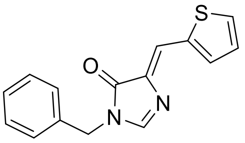

Following *General Procedure A* at rt, compound **5k** was obtained as a yellow solid (215 mg, 80%) from **1l** (93 mg, 1.1 mmol), **2a** (120 μL, 1.1 mmol), **3a** (91 μL, 1 mmol) and $AgNO_3$ (17 mg, 0.1 mmol) in CH_3OH (5 mL) after flash chromatography purification (EtOAc/hexane gradient from 0:100 to 25:75 v/v). **^{1}H NMR** (400 MHz, $CDCl_3$) δ 7.58 (s, 1H), 7.57 (d, *J* = 3.5 Hz, 1H), 7.46 (d, *J* = 3.7 Hz, 1H), 7.43 (s, 1H), 7.30 – 7.22 (m, 3H), 7.22 – 7.17 (m, 2H), 7.03 (t, *J* = 3.7 Hz, 1H), 4.69 (s, 2H). **^{13}C NMR** (101 MHz, $CDCl_3$) δ 168.86, 151.78, 137.42, 136.12, 135.53, 135.44, 134.72, 129.10, 128.29, 127.79, 127.71, 124.22, 45.03. **HRMS:** *m/z* calcd. for $C_{15}H_{13}N_2OS^+$ $[M+H]^+$: 269.0743; found: 269.0746.

(*Z*)-3-(4-chlorobenzyl)-5-(thiophen-2-ylmethylene)-3,5-dihydro-4*H*-imidazol-4-one (5l)

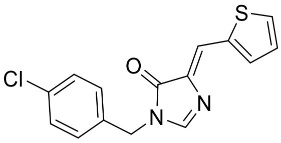

Following *General Procedure A* at rt, compound **5l** was obtained as a yellow solid (250 mg, 83%) from **1l** (93 mg, 1.1 mmol), **2c** (134 μL, 1.1 mmol), **3a** (91 μL, 1 mmol) and $AgNO_3$ (17 mg, 0.1 mmol) in CH_3OH (5 mL) after flash chromatography purification (EtOAc/hexane gradient from 0:100 to 25:75 v/v). **^{1}H NMR** (400 MHz, $CDCl_3$) δ 7.60 (d, *J* = 5.1, 1H), 7.59 (s, 1H), 7.48 (d, *J* = 3.7 Hz, 1H), 7.44 (s, 1H), 7.27 (d, *J* = 8.5 Hz, 2H), 7.16 (d, *J* = 8.5 Hz, 1H), 7.05 (dd, *J* = 5.1, 3.7 Hz, 1H), 4.67 (s, 2H). **^{13}C NMR** (101 MHz, $CDCl_3$) δ 168.79, 151.37, 137.33, 135.86, 135.66, 134.96, 134.27, 134.10, 129.27, 129.11, 127.77, 124.57, 44.39. **HRMS:** *m/z* calcd. for $C_{15}H_{12}ClN_2OS^+$ $[M+H]^+$: 303.0353; found: 303.0359.

(*Z*)-3-methyl-5-(thiophen-2-ylmethylene)-3,5-dihydro-4*H*-imidazol-4-one (5m)

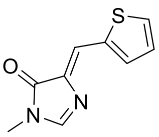

Following *General Procedure A* under μW irradiation, compound **5m** was obtained as a yellow solid (60 mg, 78%) from **1l** (41 mg, 0.44 mmol), **2i** (9.8 M solution in MeOH, 41 μL, 0.44 mmol), **3a** (36 μL, 0.4 mmol) and $AgNO_3$ (6.8 mg, 0.04 mmol) in CH_3OH (2 mL) after flash chromatography purification (EtOAc/hexane gradient from 0:100 to 50:50 v/v). **^{1}H NMR** (400 MHz, $CDCl_3$) δ 7.69 (d, *J* = 1.7 Hz, 1H), 7.65 (dt, *J* = 5.0, 1.1 Hz, 1H), 7.55 – 7.49 (m, 1H), 7.46 (d, *J* = 1.7 Hz, 1H), 7.11 (dd, *J* = 5.1, 3.7 Hz, 1H), 3.22 (s, 3H). **^{13}C NMR** (101 MHz, $CDCl_3$) δ 169.41, 152.66, 137.39, 136.10, 135.32, 134.54, 127.68, 123.86, 27.78. **HRMS:** *m/z* calcd. for $C_9H_9N_2OS^+$ $[M+H]^+$: 193.0430; found: 193.0432.

(*Z*)-5-(4-((*tert*-Butyldimethylsilyl)oxy)benzylidene)-3-(4-methylbenzyl)-3,5-dihydro-4*H*-imidazol-4-one (5n)

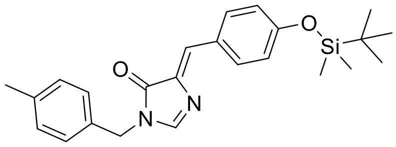

Following *General Procedure A* at rt, compound **5n** was obtained as a pale-yellow solid (185 mg, 43%) from **1g** (236 mg, 1.1 mmol), **2b** (140 μL, 1.1 mmol), **3a** (91 μL, 1 mmol) and $AgNO_3$ (17 mg, 0.1 mmol) in CH_3OH (5 mL) after flash chromatography purification (EtOAc/hexane gradient from 0:100 to 30:70 v/v). **^{1}H NMR** (400 MHz, $CDCl_3$) δ 8.05 (d, *J* = 8.5 Hz, 2H), 7.65 (s, 1H), 7.24 (s, 1H), 7.17 (s, 4H), 6.88 (d, *J* = 8.4 Hz, 2H), 4.73 (s, 2H), 2.34 (s, 3H), 0.98 (s, 9H), 0.23 (s, 6H). **^{13}C NMR** (101 MHz, $CDCl_3$) δ 169.62, 158.57, 151.93, 138.08, 136.57, 134.55, 132.58, 131.01, 129.73, 127.81, 127.25, 120.62, 44.76, 25.63, 21.13, 18.29, -4.32. **HRMS:** *m/z* calcd. for $C_{24}H_{31}N_2O_2Si^+$ [M+H]$^+$: 407.2149; found: 407.2150.

(*Z*)-5-(4-Hydroxybenzylidene)-3-(4-methylbenzyl)-3,5-dihydro-4*H*-imidazol-4-one (5n')

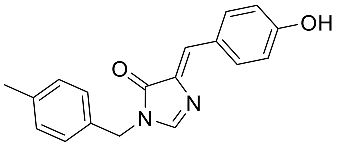

To a 0 ºC solution of compound **5n** (52 mg, 0.13 mmol, 1 eq.) in THF (1.5 mL) was added a solution of TBAF·$3H_2O$ (60 mg, 0.19 mmol, 1.5 eq.) in THF (0.5 mL). The reaction was stirred at 0 ºC for 30 min. After consumption of starting material (TLC control), the reaction was quenched with the addition of water (20 mL) and the mixture was partitioned between EtOAc (60 mL) and water. The organic layer was washed with saturated NH_4Cl aqueous solution (3 x 20 mL) and saturated brine, dried over Na_2SO_4, filtered, and concentrated under reduced pressure to afford the title compound **5n'** as a yellow solid (33 mg, 91%). **^{1}H NMR** (400 MHz, DMSO-*d*$_6$) δ 10.23 (s, 1H), 8.21 (s, 1H), 8.10 (d, *J* = 8.4 Hz, 2H), 7.22 – 7.13 (m, 4H), 7.07 (s, 1H), 6.85 (d, *J* = 8.8, 2H), 4.71 (s, 2H), 2.27 (s, 3H). **^{13}C NMR** (101 MHz, DMSO-*d*$_6$) δ 169.52, 160.71, 154.06, 137.32, 136.33, 135.17, 134.42, 129.71, 129.64, 127.89, 125.46, 116.36, 44.25, 21.14. **HRMS:** *m/z* calcd. for $C_{18}H_{17}N_2O_2^+$ [M+H]$^+$: 293.1285; found: 293.1286.

Compound **5n'** was also obtained as an "*in-situ* deprotection" minor by-product in the synthesis of **5n**, in a 5% yield.

***Tert*-butyl (*Z*)-3-((1-methyl-5-oxo-1,5-dihydro-4*H*-imidazol-4-ylidene)methyl)-1*H*-indole-1-carboxylate (5o)**

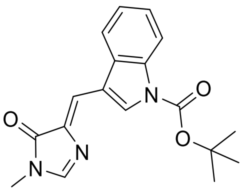

Following *General Procedure A* at rt, compound **5o** was obtained as a yellow solid (170 mg, 52%) from **1n** (269 mg, 1.1 mmol), **2i** (9.8 M solution in MeOH, 112 μL, 1.1 mmol) **3a** (91 μL, 1 mmol) and $AgNO_3$ (17 mg, 0.1 mmol) in CH_3OH (5 mL) after flash chromatography purification (EtOAc/hexane gradient from 0:100 to 20:80 v/v). **^{1}H NMR** (400 MHz, $CDCl_3$) δ 8.69 (d, *J* = 0.9 Hz, 1H), 8.21 – 8.16 (m, 1H), 7.97 – 7.92 (m, 1H), 7.68 (d, *J* = 1.7 Hz, 1H), 7.50 (dd, *J* = 1.7, 0.9 Hz, 1H), 7.36 (pd, *J* = 7.3, 1.5 Hz, 2H), 3.25 (s, 3H), 1.70 (s, 9H). **^{13}C NMR** (101 MHz, $CDCl_3$) δ 169.55, 152.43, 149.19, 137.58, 135.37, 131.97, 129.39, 125.21, 123.62, 121.45, 119.25, 115.42, 115.13, 84.79, 28.13, 27.76. **HRMS:** *m/z* calcd. for $C_{18}H_{20}N_3O_3^+$ [M+H]$^+$: 326.1499; found: 326.1501.

(*Z*)-5-((1H-indol-3-yl)methylene)-3-methyl-3,5-dihydro-4H-imidazol-4-one (5o')

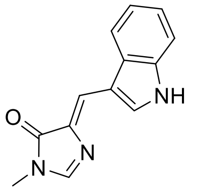

To a 0 ºC solution of compound **5o** (100 mg, 0.307 mmol) in CH_2Cl_2 (4 mL) was slowly added TFA (2.4 mL). The reaction was allowed to warm up to room temperature and was stirred for 2 h, until analysis by TLC suggested consumption of starting material. The solvent

and excess TFA were evaporated with a stream of argon. The residue was triturated with CH_2Cl_2 / hexanes and evaporated to dryness to give the pure product **5o'** (65 mg, 94%) as a bright orange solid. Compound **5o'** was obtained as a 90:10 mixture of diastereomers. NMR data is only given of the major diastereomer due to peaks overlapping. **^{1}H NMR** (400 MHz, DMSO-*d$_6$*) δ 12.05 (s, 1H), 8.39 (s, 1H), 8.31 (d, *J* = 7.3 Hz, 1H), 8.01 (d, *J* = 1.6 Hz, 1H), 7.47 (ddd, *J* = 8.0, 1.4, 0.8 Hz, 1H), 7.43 (dd, *J* = 1.6, 0.7 Hz, 1H), 7.24 – 7.14 (m, 2H), 3.14 (s, 3H). **^{13}C NMR** (101 MHz, DMSO-*d$_6$*) δ 169.43, 152.01, 137.06, 134.41, 133.98, 127.03, 123.32, 123.20, 121.44, 120.61, 112.71, 111.74, 27.81. **HRMS:** *m/z* calcd. for $C_{13}H_{12}N_3O^+$ $[M+H]^+$: 226.0975; found: 226.0973.

(*Z*)-3-(4-Methylbenzyl)-5-((1-tosyl-1*H*-indol-3-yl)methylene)-3,5-dihydro-4*H*-imidazol-4-one (5p)

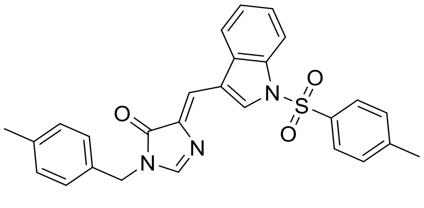

Following *General Procedure A* at rt, compound **5p** was obtained as a yellow solid (263 mg, 56%) from **1o** (330 mg, 1.1 mmol), **2b** (140 μL, 1.1 mmol) **3a** (91 μL, 1 mmol) and $AgNO_3$ (17 mg, 0.1 mmol) in CH_3OH (5 mL) after flash chromatography purification (EtOAc/hexane gradient from 0:100 to 25:75 v/v). **^{1}H NMR** (400 MHz, $CDCl_3$) δ 8.75 (s, 1H), 7.99 (d, *J* = 7.4 Hz, 1H), 7.90 – 7.81 (m, 3H, overlapped signals), 7.69 (d, *J* = 1.7 Hz, 1H), 7.46 (s, 1H), 7.41 – 7.30 (m, 2H), 7.23 (d, *J* = 8.1 Hz, 2H), 7.22 – 7.15 (m, 4H), 4.74 (s, 2H), 2.35 (s, 3H), 2.33 (s, 3H). **^{13}C NMR** (101 MHz, $CDCl_3$) δ 168.80, 152.24, 145.52, 138.17, 137.97, 134.83, 134.64, 132.49, 131.81, 130.11, 129.77, 129.61, 127.91, 127.15, 125.41, 124.07, 120.59, 119.60, 116.23, 113.63, 44.87, 21.62, 21.15. **HRMS:** *m/z* calcd. for $C_{27}H_{24}N_3O_3S^+$ $[M+H]^+$: 470.1533; found: 470.1542.

(*Z*)-3-(4-Methylbenzyl)-5-(pyridin-4-ylmethylene)-3,5-dihydro-4*H*-imidazol-4-one (5q)

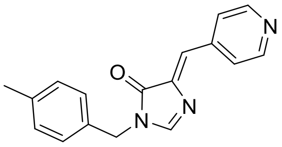

Following *General Procedure A* under μW irradiation, compound **5q** was obtained as a pale brown solid (70 mg, 28%) from **1r** (104 mg, 1.1 mmol), **2b** (140 μL, 1.1 mmol), **3a** (91 μL, 1 mmol) and $AgNO_3$ (17 mg, 0.1 mmol) in CH_3OH (5 mL) after flash chromatography purification (EtOAc/hexane gradient from 10:90 to 60:40 v/v). **^{1}H NMR** (400 MHz, $CDCl_3$) δ 8.70 – 8.66 (m, 2H), 7.94 – 7.90 (m, 2H), 7.75 (d, *J* = 1.7 Hz, 1H), 7.18 (s, 4H), 7.13 (d, *J* = 1.7 Hz, 1H), 4.73 (s, 2H), 2.35 (s, 3H). **^{13}C NMR** (101 MHz, $CDCl_3$) δ 169.21, 155.40, 150.34, 141.97, 140.55, 138.41, 131.98, 129.86, 127.88, 126.97, 125.40, 44.97, 21.14. **HRMS:** *m/z* calcd. for $C_{17}H_{16}N_3O^+$ $[M+H]^+$: 278.1288; found: 278.1228.

(*Z*)-5-((1*H*-Indol-2-yl)methylene)-3-(4-methylbenzyl)-3,5-dihydro-4*H*-imidazol-4-one (5r)

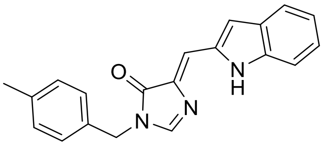

Following *General Procedure A* under μW irradiation, compound **5r** was obtained as a pale green solid (59 mg, 47%) from **1p** (64 mg, 0.44 mmol), **2b** (56 μL, 0.44 mmol), **3a** (36 μL, 0.4 mmol) and $AgNO_3$ (7 mg, 0.04 mmol) in CH_3OH (2 mL). The product precipitated from the reaction mixture and was isolated by filtration under reduced pressure. **^{1}H NMR** (400 MHz, $CDCl_3$) δ 10.77 (s, 1H), 7.67 (d, *J* = 1.6 Hz, 1H), 7.64 (dq, *J* = 8.1, 1.0 Hz, 1H), 7.41 (dq, *J* = 8.4, 1.0 Hz, 1H), 7.33 (d, *J* = 1.6 Hz, 1H), 7.31 – 7.26 (m, 1H), 7.23 – 7.16 (m, 4H), 7.14 – 7.05 (m, 1H), 7.01 (d, *J* = 2.1 Hz, 1H), 4.76 (s, 2H), 2.35 (s, 3H). **^{13}C NMR** (101 MHz, $CDCl_3$) δ 168.43, 150.81, 139.33, 138.20, 136.35, 133.90, 132.50, 129.79, 128.00, 127.90, 125.67, 122.04, 120.54, 120.27, 113.32, 111.65, 44.83, 21.15. **HRMS:** *m/z* calcd. for $C_{20}H_{18}N_3O^+$ $[M+H]^+$: 316.1444; found: 316.1448.

(*Z*)-3-Benzyl-5-((*E*)-3-(4-chlorophenyl)allylidene)-3,5-dihydro-4*H*-imidazol-4-one (5s)

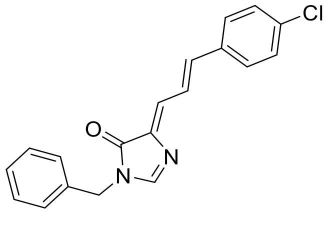

Following *General Procedure A* at rt, compound **5s** was obtained as an orange solid (64 mg, 20%) from **1t** (183 mg, 1.1 mmol), **2a** (120 µL, 1.1 mmol), **3a** (91 µL, 1 mmol) and $AgNO_3$ (17 mg, 0.1 mmol) in CH_3OH (5 mL) after flash chromatography purification (EtOAc/hexane gradient from 20:80 to 80:20 v/v). **^{1}H NMR** (400 MHz, $CDCl_3$) δ 7.55 (d, *J* = 1.6 Hz, 1H), 7.52 (dd, *J* = 15.7, 11.6 Hz, 1H), 7.45 – 7.40 (m, 2H), 7.33 – 7.25 (m, 5H, complex overlapped aromatic signals), 7.23 – 7.20 (m, 2H), 7.06 (dt, *J* = 11.7, 1.3 Hz, 1H), 6.97 (d, *J* = 15.7 Hz, 1H), 4.69 (s, 2H). **^{13}C NMR** (101 MHz, $CDCl_3$) δ 168.48, 151.59, 142.02, 139.40, 135.50, 134.60, 131.63, 129.16, 129.12, 128.90, 128.33, 127.82, 123.65, 45.01. **HRMS:** *m/z* calcd. for $C_{19}H_{16}ClN_2O^+$ [M+H]$^+$: 323.0946; found: 323.0951.

(*Z*)-3-Butyl-5-(thiophen-2-ylmethylene)-3,5-dihydro-4*H*-imidazol-4-one (5t)

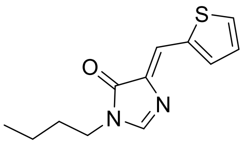

Following *General Procedure A* under µW irradiation, compound **5t** was obtained as a golden colored solid (0.97 g, 83%) from **1l** (514 mg, 5.5 mmol), **2j** (541 µL, 5.5 mmol), **3a** (454 µL, 5 mmol) and $AgNO_3$ (85 mg, 0.5 mmol) in CH_3OH (15 mL, 0.33 M) after flash chromatography purification (EtOAc/hexane gradient from 0:100 to 15:85 v/v). **1H NMR** (400 MHz, CDCl3) δ 7.65 (d, J = 1.6 Hz, 1H), 7.59 (d, J = 5.0 Hz, 1H), 7.46 (d, J = 3.8 Hz, 1H), 7.39 (d, J = 1.7 Hz, 1H), 7.05 (dd, J = 5.1, 3.7 Hz, 1H), 3.53 (t, J = 7.2 Hz, 2H), 1.65 – 1.53 (m, 3H), 1.37 – 1.24 (m, 2H), 0.88 (t, J = 7.4 Hz, 3H). **^{13}C NMR** (101 MHz, $CDCl_3$) δ 169.14, 152.35, 137.47, 136.45, 135.21, 134.44, 127.66, 123.66, 41.19, 31.33, 19.85, 13.55. **HRMS:** *m/z* calcd. for $C_{12}H_{15}N_2OS^+$ [M+H]$^+$: 235.0900; found: 235.0906.

(*Z*)-3-(2-(1*H*-Indol-3-yl)ethyl)-5-(benzo[*d*][1,3]dioxol-5-ylmethylene)-3,5-dihydro-4*H*-imidazol-4-one (5u)

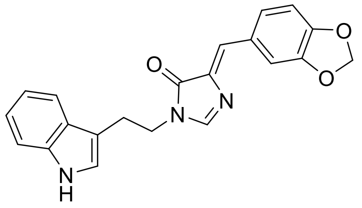

Following *General Procedure A* under µW irradiation, compound **5u** was obtained as a yellow solid (160 mg, 42%) from **1k** (165 mg, 1.1 mmol), **2g** (176 mg, 1.1 mmol), **3a** (91 µL, 1 mmol) and $AgNO_3$ (17 mg, 0.1 mmol) in CH_3OH (5 mL) after flash chromatography purification (EtOAc/hexane gradient from 0:100 to 45:55 v/v). **^{1}H NMR** (400 MHz, DMSO-*d$_6$*) δ 10.87 (s, 1H), 8.01 (d, *J* = 14.2, 2H), 7.61 (d, *J* = 8.3, 1H), 7.58 (d, *J* = 7.8 Hz, 1H), 7.35 (d, *J* = 8.0 Hz, 1H), 7.15 (s, 1H), 7.12 – 6.95 (m, 4H, complex signal), 6.10 (s, 2H), 3.84 (t, *J* = 7.2 Hz, 2H), 3.05 (t, *J* = 7.2 Hz, 2H). **^{13}C NMR** (101 MHz, DMSO-*d$_6$*) δ 169.67, 155.15, 149.91, 148.19, 137.69, 136.72, 129.32, 128.66, 128.36, 127.43, 123.62, 121.55, 118.88, 118.70, 111.94, 111.10, 110.85, 109.11, 102.21, 41.78, 24.95. **HRMS:** *m/z* calcd. for $C_{21}H_{18}N_3O_3^+$ [M+H]$^+$: 360.1343; found: 360.1344.

(*Z*)-5-(Benzo[d][1,3]dioxol-5-ylmethylene)-3-((*E*)-4-phenylbut-3-en-1-yl)-3,5-dihydro-4*H*-imidazol-4-one (5v)

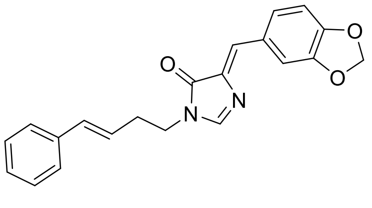

Following *General Procedure A* at rt, compound **5v** was obtained as a golden colored solid (120 mg, 35%) from **1k** (164 mg, 1.1 mmol), **2w** (165 mg, 1.1 mmol), **3a** (91 µL, 1 mmol) and $AgNO_3$ (17 mg, 0.1 mmol) in CH_3OH (5 mL). The product precipitated from the reaction

mixture and was isolated by filtration under reduced pressure. **^{1}H NMR** (400 MHz, $CDCl_3$) δ 7.95 (d, *J* = 1.6 Hz, 1H), 7.70 (d, *J* = 1.7 Hz, 1H), 7.44 (dd, *J* = 8.2, 1.7 Hz, 1H), 7.35 – 7.27 (m, 5H), 7.25 – 7.19 (m, 1H), 7.15 (d, *J* = 1.6 Hz, 1H), 6.85 (d, *J* = 8.1 Hz, 1H), 6.46 (d, *J* = 15.9 Hz, 1H), 6.19 – 6.09 (m, 1H), 6.02 (s, 2H), 3.75 (t, *J* = 6.9 Hz, 2H), 2.64 – 2.56 (m, 2H). **^{13}C NMR** (101 MHz, $CDCl_3$) δ 169.82, 152.29, 150.09, 148.26, 137.01, 136.85, 133.52, 130.52, 129.13, 128.58, 128.39, 127.55, 126.18, 125.19, 111.39, 108.57, 101.64, 41.20, 32.87. **HRMS:** *m/z* calcd. for $C_{21}H_{19}N_2O_3^+$ [M+H]$^+$: 347.1390; found: 347.1394.

(*Z*)-5-(Benzo[d][1,3]dioxol-5-ylmethylene)-3,5-dihydro-4*H*-imidazol-4-one (5w)

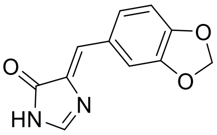

Following *General Procedure A* at rt, compound **5w** was obtained as a light brown solid (130 mg, 30%) from **1k** (330 mg, 2.2 mmol), **2h** (7 M solution in MeOH, 290 µL, 2.2 mmol), **3a** (181 µL, 2 mmol) and $AgNO_3$ (34 mg, 0.2 mmol) in CH_3OH (10 mL) after flash chromatography purification (EtOAc/hexane gradient from 25:75 to 75:25 v/v). **^{1}H NMR** (400 MHz, DMSO-*d$_6$*) δ 11.33 (brs, 1H), 8.13 (d, *J* = 1.7 Hz, 1H), 8.01 (d, *J* = 1.6 Hz, 1H), 7.60 (dd, *J* = 8.3, 1.7 Hz, 1H), 7.00 (d, *J* = 8.1 Hz, 1H), 6.96 (d, *J* = 1.7 Hz, 1H), 6.10 (s, 2H). **^{13}C NMR** (101 MHz, DMSO-*d$_6$*) δ 171.47, 153.73, 149.71, 148.18, 137.63, 129.04, 128.78, 127.19, 110.99, 109.09, 102.15. **HRMS:** *m/z* calcd. for $C_{11}H_9N_2O_3^+$ [M+H]$^+$: 217.0608; found: 217.0611.

Allyl (*S,Z*)-2-((((9*H*-fluoren-9-yl)methoxy)carbonyl)amino)-6-(4-(benzo[*d*][1,3]dioxol-5-ylmethylene)-5-oxo-4,5-dihydro-1*H*-imidazol-1-yl)hexanoate (5x)

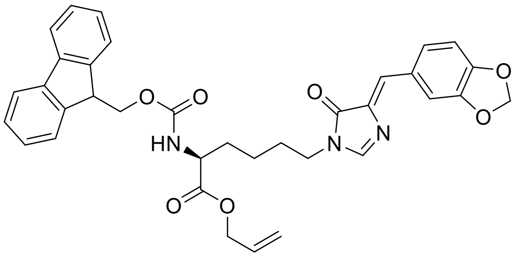

Following *General Procedure A* at rt, compound **5x** was obtained as a light brown solid (0.95 g, 47%) from **1k** (553 mg, 3.7 mmol), **2x** (1.64 g, 1.1 mmol), **3a** (304 µL, 1 mmol), $AgNO_3$ (114 mg, 0.1 mmol) and triethylamine (536 µL, 1.2 mmol) in CH_3OH (17 mL) after flash chromatography purification (EtOAc/hexane gradient from 0:100 to 35:65 v/v). **^{1}H NMR** (400 MHz, DMSO-*d$_6$*) δ 8.22 (d, *J* = 1.6 Hz, 1H), 8.02 (d, *J* = 1.6 Hz, 1H), 7.88 (d, *J* = 7.5 Hz, 2H), 7.80 (d, *J* = 7.8 Hz, 1H), 7.73 – 7.67 (m, 2H), 7.62 (dd, *J* = 8.3, 1.7 Hz, 1H), 7.40 (t, *J* = 7.4 Hz, 2H), 7.34 – 7.29 (m, 2H), 7.05 (d, *J* = 1.6 Hz, 1H), 7.01 (d, *J* = 8.1 Hz, 1H), 6.10 (s, 2H), 5.93 – 5.81 (m, 1H), 5.29 (dq, *J* = 17.3, 1.7 Hz, 1H), 5.18 (dq, *J* = 10.6, 1.5 Hz, 1H), 4.56 (dq, *J* = 5.5, 1.4 Hz, 2H), 4.29 (d, *J* = 6.3 Hz, 2H), 4.21 (t, *J* = 7.0 Hz, 1H), 4.07 – 3.99 (m, 1H), 3.53 (t, *J* = 6.9 Hz, 2H), 1.79 – 1.55 (m, 4H), 1.37 – 1.26 (m, 2H). **^{13}C NMR** (101 MHz, DMSO-*d$_6$*) δ 172.50, 169.73, 156.62, 155.25, 149.93, 148.20, 144.25, 141.20, 137.67, 132.84, 129.35, 128.67, 128.49, 128.09, 127.52, 125.68, 120.58, 118.22, 111.09, 109.11, 102.22, 66.12, 65.25, 54.28, 47.09, 40.85, 30.63, 28.54, 23.11. **HRMS:** *m/z* calcd. for $C_{35}H_{34}N_3O_7^+$ [M+H]$^+$: 608.2391; found: 608.2393.

(*S,Z*)-2-((((9*H*-fluoren-9-yl)methoxy)carbonyl)amino)-6-(4-(benzo[*d*][1,3]dioxol-5-ylmethylene)-5-oxo-4,5-dihydro-1*H*-imidazol-1-yl)hexanoic acid (5x')

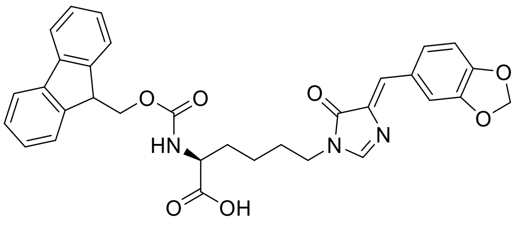

To a solution of **5x** (156 mg, 0.26 mmol, 1 eq.) and tetrakis(triphenylphosphine)palladium(0) (119 mg, 0.1 mmol, 0.4 eq.) in dry CH_2Cl_2 (5.1 mL, 0.05 M) under argon atmosphere, was added 1,3-dimethylbarbituric acid (200 mg, 1.3 mmol, 5 eq.) and the reaction was stirred at rt for 1 h. After reaction completion (LC-MS control) the reaction mixture was concentrated under reduced pressure. The crude product was purified *via* reverse phase C18 chromatography (CH_3CN + 0.1% HCOOH / H_2O + 0.1% HCOOH gradient from 5:95 to 80:20 v/v) to afford the title compound **5x'** as a pale-yellow solid (80 mg, 55%). **^{1}H NMR** (400 MHz, DMSO-*d$_6$*) δ 8.22 (d, *J* = 1.7 Hz, 1H), 8.01 (d, *J* = 1.6 Hz, 1H), 7.88 (d, *J* = 7.5 Hz, 2H), 7.70 (d, *J* = 7.5 Hz, 2H), 7.64 – 7.59 (m, 2H), 7.53 – 7.48 (m, 1H), 7.39 (t, *J* =

7.4 Hz, 2H), 7.32 (td, *J* = 7.2, 2.3 Hz, 2H), 7.05 (d, *J* = 1.7 Hz, 1H), 7.00 (d, *J* = 8.0 Hz, 1H), 6.10 (s, 2H), 4.28 – 4.19 (m, 3H), 3.94 – 3.84 (m, 1H), 3.53 (t, *J* = 6.9 Hz, 2H), 1.77 – 1.54 (m, 4H), 1.37 – 1.27 (m, 2H). **^{13}C NMR** (101 MHz, DMSO-*d$_6$*) δ 174.37, 169.71, 156.57, 155.28, 149.92, 148.20, 144.28, 141.16, 137.67, 131.91, 129.29, 128.47, 128.08, 127.53, 125.71, 120.56, 111.09, 109.11, 102.21, 66.04, 54.25, 47.12, 40.90, 30.81, 28.64, 23.19. **HRMS:** *m/z* calcd. for $C_{32}H_{27}N_3O_7^-$ [M-H]$^-$: 566.1933; found: 567.1931.

(*Z*)-3-(4-Methoxyphenyl)-5-(thiophen-2-ylmethylene)-3,5-dihydro-4*H*-imidazol-4-one (5y)

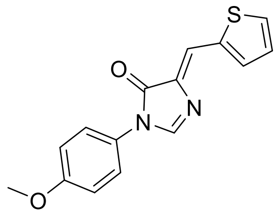

Following *General Procedure A* at rt, compound **5y** was obtained as a light brown solid (35 mg, 12%) from **1l** (103 μL, 1.1 mmol), **2q** (135 mg, 1.1 mmol), **3a** (91 μL, 1 mmol) and $AgNO_3$ (17 mg, 0.1 mmol) in CH_3OH (5 mL). The product and unreacted aniline were collected by filtration washing with cold MeOH. The solid was dissolved in CH_2Cl_2 and was filtered through Celite®® – to remove the remains of the silver catalyst. The solution was washed with 0.5 M aqueous HCl (3x) and saturated brine. The organic layer was dried over Na_2SO_4, filtered, and concentrated under reduced pressure to give the crude product **5y**. The pure product **5y** was obtained after crystallization by slow diffusion of Et_2O into a CH_2Cl_2 solution of the pure product through layering. **^{1}H NMR** (400 MHz, $CDCl_3$) δ 7.95 (d, *J* = 1.6 Hz, 1H), 7.70 (dt, *J* = 5.1, 1.0 Hz, 1H), 7.59 – 7.55 (m, 2H, overlapped signals), 7.40 – 7.33 (m, 2H), 7.14 (dd, *J* = 5.1, 3.7 Hz, 1H), 7.03 – 6.96 (m, 2H), 3.84 (s, 3H). **^{13}C NMR** (101 MHz, $CDCl_3$) δ 167.68, 158.94, 151.09, 137.42, 136.20, 135.56, 134.84, 127.76, 126.83, 124.64, 124.25, 114.85, 55.59. **HRMS:** *m/z* calcd. for $C_{15}H_{13}N_2O_2S^+$ [M+H]$^+$: 285.0692; found: 285.0693.

(*Z*)-5-(Benzo[d][1,3]dioxol-5-ylmethylene)-3-(tert-butyl)-3,5-dihydro-4*H*-imidazol-4-one (5z)

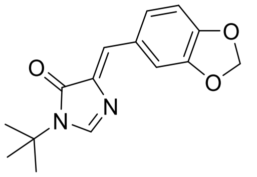

Following *General Procedure A* under μW irradiation, compound **5z** was obtained as a pale yellow solid (10 mg, 2%) from **1k** (330 mg, 2.2 mmol), **2l** (231 μL, 2.2 mmol), **3a** (181 μL, 2 mmol) and $AgNO_3$ (34 mg, 0.2 mmol) in CH_3OH (10 mL) after flash chromatography purification (EtOAc/hexane gradient from 20:80 to 70:30 v/v). **^{1}H NMR** (400 MHz, $CDCl_3$) δ 7.95 (d, *J* = 1.7 Hz, 1H), 7.83 (d, *J* = 1.7 Hz, 1H), 7.43 (dd, *J* = 8.1, 1.6 Hz, 1H), 7.05 (d, *J* = 1.6 Hz, 1H), 6.85 (d, *J* = 8.1 Hz, 1H), 6.02 (s, 2H), 1.57 (s, 9H). **^{13}C NMR** (101 MHz, $CDCl_3$) δ 170.35, 151.14, 149.82, 148.21, 138.47, 129.47, 128.77, 128.63, 111.27, 108.55, 101.58, 55.11, 28.16. **HRMS:** *m/z* calcd. for $C_{15}H_{17}N_2O_3^+$ [M+H]$^+$: 273.1234; found: 273.1237.

3-Benzyl-5-(propan-2-ylidene)-3,5-dihydro-4*H*-imidazol-4-one (5aa)

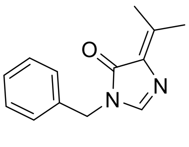

Following *General Procedure A* under μW irradiation, compound **5aa** was obtained as a pale brown oil (130 mg, 60%) from **1aa** (81 μL, 1.1 mmol), **2a** (120 μL, 2.2 mmol), **3a** (91 μL, 1 mmol) and $AgNO_3$ (17 mg, 0.1 mmol) in CH_3OH (5 mL) after flash chromatography purification (EtOAc/hexane gradient from 0:100 to 60:40 v/v). **^{1}H NMR** (400 MHz, $CDCl_3$) δ 7.46 (s, 1H), 7.38 – 7.29 (m, 3H), 7.27 – 7.24 (m, 2H), 4.71 (s, 2H), 2.44 (s, 3H), 2.27 (s, 3H). **^{13}C NMR** (101 MHz, $CDCl_3$) δ 167.40, 152.84, 147.89, 136.69, 135.92, 129.00, 128.10, 127.74, 44.65, 22.44, 19.62. **HRMS:** *m/z* calcd. for $C_{18}H_{15}N_2O^+$ [M+H]$^+$: 215.1179; found: 215.1177.

Compound **5aa** was isolated from the same reaction mixture as compound **4aa**.

3-Benzyl-5-cyclopentylidene-3,5-dihydro-4*H*-imidazol-4-one (5ab)

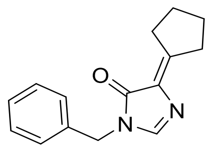

Following *General Procedure A* under μW irradiation, compound **5ab** was obtained as a pale brown oil (146 mg, 30%) from **1ab** (195 μL, 2.2 mmol), **2a** (240 μL, 2.2 mmol), **3a** (181 μL, 2 mmol) and $AgNO_3$ (34 mg, 0.2 mmol) in CH_3OH (10 mL) after flash chromatography purification (EtOAc/hexane gradient from 0:100 to 60:40 v/v). **^{1}H NMR** (400 MHz, DMSO-*d₆* with a drop of $CDCl_3$) δ 7.50 (s, 1H), 7.36 – 7.27 (m, 3H), 7.26 – 7.22 (m, 2H), 4.70 (s, 2H), 2.99 – 2.90 (m, 2H), 2.88 – 2.79 (m, 2H), 1.88 – 1.73 (m, 4H). **^{13}C NMR** (101 MHz, DMSO-d_6) δ 166.83, 160.92, 150.22, 137.16, 133.34, 128.67, 127.54, 127.38, 43.77, 32.84, 31.38, 26.07, 24.87. **HRMS:** *m/z* calcd. for $C_{15}H_{17}N_2O^+$ $[M+H]^+$: 240.1335; found: 240.1336.

Compound **5ab** was isolated from the same reaction mixture as compound **4ab**.

5-((1*r*,3*r*)-adamantan-2-ylidene)-3-benzyl-3,5-dihydro-4*H*-imidazol-4-one (5ac)

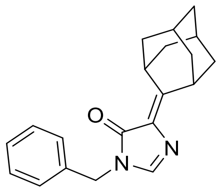

Following *General Procedure A* under μW irradiation, compound **5ac** was obtained as an off-white oil (75 mg, 12%) from **1ac** (330 mg, 2.2 mmol), **2a** (240 μL, 2.2 mmol), **3a** (181 μL, 2 mmol) and $AgNO_3$ (34 mg, 0.2 mmol) in CH_3OH (10 mL) after flash chromatography purification (EtOAc/hexane gradient from 0:100 to 40:60 v/v). **^{1}H NMR** (400 MHz, $CDCl_3$) δ 7.41 (s, 1H), 7.38 – 7.26 (m, 5H), 4.71 (s, 2H), 4.45 – 4.40 (m, 1H), 3.64 – 3.54 (m, 1H), 2.12 – 2.04 (m, 4H), 2.04 – 1.98 (m, 2H), 1.97 – 1.89 (m, 6H). **^{13}C NMR** (101 MHz, $CDCl_3$) δ 168.86, 166.90, 146.59, 134.94, 130.03, 127.95, 127.05, 126.87, 43.61, 38.99, 38.91, 35.76, 33.71, 30.62, 26.78. **HRMS:** *m/z* calcd. for $C_{20}H_{23}N_2O^+$ $[M+H]^+$: 307.1805; found: 307.1805.

Compound **5ac** was isolated from the same reaction mixture as compound **4ac**.

(5Z,5'Z)-3,3'-(Cyclohexane-1,2-diyl)bis(5-(benzo[d][1,3]dioxol-5-ylmethylene)-3,5-dihydro-4H-imidazol-4-one) (5-bis-a)

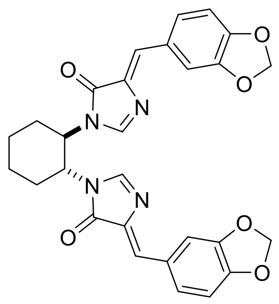

Following *General Procedure A* at rt, compound **9** was obtained as a yellow solid (120 mg, 19%) from **1k** (330 mg, 2.2 mmol), ***trans*-2n** (251 mg, 2.2 mmol), **3a** (181 μL, 2 mmol) and $AgNO_3$ (34 mg, 0.2 mmol) in CH_3OH (10 mL). The precipitate formed during the reaction was filtered under vacuum to give the crude product as a pale-yellow solid. The pure product **9** was obtained after crystallization by slow diffusion of Et_2O into a CH_2Cl_2 solution of the crude product through layering. **^{1}H NMR** (400 MHz, DMSO-d_6) δ 8.47 (d, *J* = 1.6 Hz, 1H), 8.00 (d, *J* = 1.6 Hz, 1H), 7.63 (dd, *J* = 8.2, 1.7 Hz, 1H), 7.04 (d, *J* = 8.1 Hz, 1H, overlapped), 7.03 (d, *J* = 1.6 Hz, 1H, overlapped), 6.15 (s, 2H), 4.32 – 4.22 (m, 1H), 2.10 – 1.99 (m, 1H), 2.00 – 1.92 (m, 1H), 1.92 – 1.85 (m, 1H), 1.56 – 1.47 (m, 1H). **^{13}C NMR** (101 MHz, DMSO-d_6) δ 173.67, 157.67, 154.76, 152.93, 141.95, 134.22, 133.65, 133.23, 115.87, 113.87, 106.97, 58.02, 35.63, 29.52. **HRMS:** *m/z* calcd. for $C_{28}H_{25}N_4O_6^+$ $[M+H]^+$: 513.1769; found: 513.1766.

3.5.3. Compounds 6-8

Methyl 5-(pyridin-4-yl)-4,5-dihydrooxazole-4-carboxylate (6a)

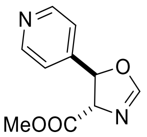

Following *General Procedure A* under μW irradiation, compound **6a** was obtained as a single diastereomer as a yellow solid (53 mg, 51%) from **1r** (52 μL, 0.55 mmol), **2l** (58 μL, 0.55 mmol), **3a** (45 μL, 0.5 mmol) and $AgNO_3$ (8.5 mg, 0.05 mmol) in CH_3OH (2.5 mL, 0.2 M) after flash chromatography purification (EtOAc/hexane gradient from 20:80 to 70:30 v/v). **^{1}H NMR** (400 MHz, $CDCl_3$) δ 8.58 (d, *J* = 6.1 Hz, 2H), 7.19 (d, *J* = 6.0 Hz, 2H, overlapped with residual solvent peak), 7.05 (d, *J* = 2.2 Hz, 1H), 5.64 (d, *J* = 7.9 Hz, 1H), 4.51 (dd, *J* = 7.8, 2.2 Hz, 1H), 3.81 (s, 3H). NMR data are in agreement with those found in the literature.[18]

Ethyl 5,5-dimethyl-4,5-dihydrooxazole-4-carboxylate (6b)

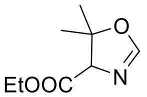

Following *General Procedure A* under μW irradiation, compound **6b** was obtained as a pale-yellow oil (83 mg, 97%) from **1aa** (40 μL, 0.55 mmol), **2l** (58 μL, 0.55 mmol), **3b** (55 μL, 0.5 mmol) and $AgNO_3$ (8.5 mg, 0.05 mmol) in CH_3OH (2.5 mL, 0.2 M). The reaction was diluted with CH_2Cl_2, filtered through Celite®® – to remove the remains of the silver catalyst –, and concentrated under reduced pressure to give the pure product **6b**. **^{1}H NMR** (400 MHz, $CDCl_3$) δ 6.92 (d, *J* = 2.0 Hz, 1H), 4.36 (d, *J* = 2.0 Hz, 1H), 4.27 – 4.18 (m, 2H), 1.54 (s, 3H), 1.32 (s, 3H, overlapped), 1.28 (t, *J* = 7.1 Hz, 3H, overlapped). NMR data are in agreement with those found in the literature.[19]

Methyl pyrimido[1,6-*a*]indole-3-carboxylate (7)

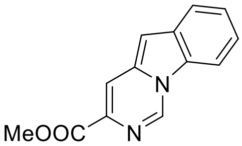

Following *Special Case II: Synthesis of Compound **7***, compound **7** was obtained as a yellow solid (46 mg, 64%) from **1p** (50 mg, 0.35 mmol), **2l** (36 μ, 0.35 mmol), **3a** (29 μL, 0.31 mmol) and $AgNO_3$ (5 mg, 0.03 mmol) in CH_3OH (1.6 mL, 0.2 M) after flash chromatography purification (EtOAc/hexane gradient from 10:90 to 50:50 v/v). **^{1}H NMR** (400 MHz, $CDCl_3$) δ 9.11 (s, 1H), 8.16 (d, *J* = 1.6 Hz, 1H), 7.99 – 7.93 (m, 1H), 7.81 – 7.74 (m, 1H), 7.46 – 7.35 (m, 2H), 6.86 (s, 1H), 3.93 (s, 3H). NMR data are in agreement with those found in the literature.[20] Compound **7** was also obtained as a sub-product in the synthesis of compound **5r** in 27% yield.

(5*R*,6*R*,7*S*,12*S*)-3,10-Dibenzyl-6,12-bis(4-chlorophenyl)-1,3,8,10-tetraazadispiro[4.1.4^7.1^5]dodeca-1,8-diene-4,11-dione (8a)

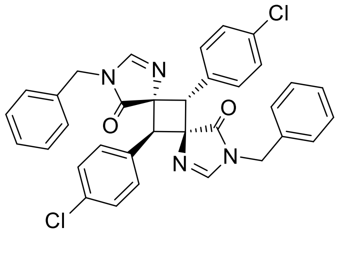

Compound **5a** (150 mg, 0.5 mmol) was dissolved in CH_2Cl_2 and evaporated under reduced pressure using a 50 mL RBF, ensuring an even distribution along the flask. Compound **5a** had a golden color. The flask was then placed under blue LEDs irradiation for 17 h. After irradiation the solid was recovered to give the title compound **8a** as a white solid (150 mg, 100%). **^{1}H NMR** (400 MHz, DMSO-*d$_6$*) δ 8.15 (s, 1H), 7.52 – 7.47 (m, 2H), 7.32 – 7.25 (m, 2H), 7.24 – 7.18 (m, 1H), 7.18 – 7.12 (m, 2H), 6.72 – 6.67 (m, 2H), 4.61 (s, 1H), 4.53 (d, *J* = 15.8 Hz, 1H), 4.43 (d, *J* = 15.9 Hz, 1H). **^{13}C NMR** (101 MHz, DMSO-*d$_6$*) δ 179.98, 155.89, 136.77, 132.93, 132.83, 132.60, 128.83, 128.03, 127.80, 127.05, 73.89, 53.10, 44.06. **HRMS**: *m/z* calcd. for $C_{34}H_{27}Cl_2N_4O_2$ [M+H]$^+$: 593.1506; found: 593.1495.

Crystals suitable for single X-ray diffraction were grown by slow diffusion of Et_2O into a $CHCl_3$ solution of the pure compound **8a** through layering.

(5*R*,6*R*,7*S*,12*S*)-3,10-Dibenzyl-6,12-bis(4-bromophenyl)-1,3,8,10-tetraazadispiro[4.1.4[7].1[5]]dodeca-1,8-diene-4,11-dione (8b)

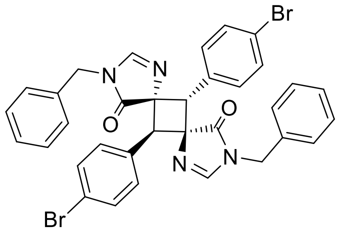

Compound **5c** (150 mg, 0.44 mmol) was suspended in CH_2Cl_2 and evaporated under reduced pressure using a 50 mL RBF, ensuring an even distribution along the flask. Compound **5c** had a golden color. The flask was then placed under blue LEDs irradiation for 17 h. After irradiation the solid was recovered to give the title compound **8b** as a white solid (150 mg, 100%). **^{1}H NMR** (400 MHz, $CDCl_3$) δ 7.62 (s, 1H), 7.45 – 7.41 (m, 2H), 7.41 – 7.37 (m, 2H), 7.26 – 7.18 (m, 3H), 6.79 – 6.73 (m, 2H), 4.72 (s, 1H), 4.63 (d, *J* = 15.4 Hz, 1H), 4.26 (d, *J* = 15.4 Hz, 1H). **^{13}C NMR** (101 MHz, $CDCl_3$) δ 179.41, 153.87, 135.11, 132.15, 132.05, 131.05, 128.93, 128.11, 127.22, 122.33, 74.29, 53.83, 44.68. **HRMS**: *m/z* calcd. for $C_{34}H_{27}Cl_2N_4O_2^+$ $[M+H]^+$: 681.0495; found: 681.0487.

3.5.4. Compounds 9

Due to the low stability of compounds **9**, cleaner NMR spectra were obtained when acquired immediately after purification. Therefore, the NMR spectra of some analogues contain residual solvent from the chromatography. Under strict solvent removal meaningful decomposition was observed in some cases.

2-(4-Chlorobenzyl)-8-(4-chlorophenyl)-6,7-dihydroimidazo[1,2-*a*]pyrazin-3(5*H*)-one (9a)

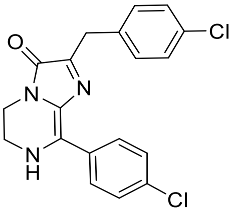

Following *General Procedure A* at rt under argon atm., compound **9a** was obtained as a yellow solid (30 mg, 8%) from **1a** (140 mg, 1.1 mmol), **2m** (74 μL, 1.1 mmol), **3a** (91 μL, 1 mmol) and $AgNO_3$ (17 mg, 0.1 mmol) in CH_3OH (5 mL) after flash chromatography purification (CH_2Cl_2/hexane gradient from 0:100 to 80:20 v/v). **^{1}H NMR** (400 MHz, DMSO-*d$_6$*) δ 7.95 (br s, 1H), 7.83 – 7.78 (m, 2H), 7.60 – 7.54 (m, 2H), 7.34 – 7.27 (m, 4H), 3.82 (s, 2H), 3.81 – 3.74 (m, 2H), 3.59 – 3.54 (m, 2H). **^{13}C NMR** (101 MHz, DMSO-*d$_6$*) δ 159.97, 150.09, 137.88, 135.18, 133.91, 131.53, 131.25, 131.01, 130.84, 128.79, 128.69, 126.58, 40.67 (overlapped with the residual solvent signal), 38.32, 33.18. **HRMS:** *m/z* calcd. for $C_{19}H_{16}Cl_2N_3O$ $[M+H]^+$: 372.0665; found: 372.0670.

2-(4-Iodobenzyl)-8-(4-iodophenyl)-6,7-dihydroimidazo[1,2-*a*]pyrazin-3(5*H*)-one (9b)

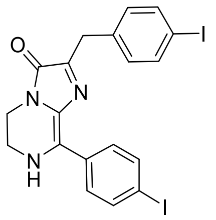

Following *General Procedure A* at rt under argon atm., compound **9b** was obtained as an orange solid (74 mg, 7%) from **1c** (510 mg, 2.2 mmol), **2m** (147 μL, 2.2 mmol), **3a** (182 μL, 2 mmol) and $AgNO_3$ (36 mg, 0.21 mmol) in CH_3OH (10 mL) after flash chromatography purification (CH_2Cl_2/hexane gradient from 0:100 to 90:10 v/v). **^{1}H NMR** (400 MHz, DMSO-*d$_6$*) δ 7.91 (t, *J* = 3.2 Hz, 1H), 7.87 – 7.83 (m, 2H), 7.63 – 7.59 (m, 2H), 7.59 – 7.54 (m, 2H), 7.12 – 7.06 (m, 2H), 3,76 (s, 2H, overlapped), 3.78 – 3.70 (m, 2H, overlapped), 3.54 (m, 2H). **^{13}C NMR** (101 MHz, DMSO-*d$_6$*) δ 159.49, 149.52, 138.24, 137.09, 137.02, 133.77, 131.19, 131.15, 131.03, 126.05, 97.11, 91.83, 37.83, 32.90. **HRMS:** *m/z* calcd. for $C_{19}H_{16}I_2N_3O^+$ $[M+H]^+$: 555.9377; found: 555.9377.

The missing CH_2 signal in ^{13}C NMR is likely overlapped with residual solvent signal, in agreement with the spectra of the other compounds in this series.

4-(2-(4-Cyanobenzyl)-3-oxo-3,5,6,7-tetrahydroimidazo[1,2-*a*]pyrazin-8-yl)benzonitrile (9c)

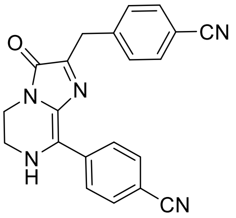

Following *General Procedure A* at rt under argon atm., compound **9c** was obtained as a bright orange solid (80 mg, 9%) from **1q** (360 mg, 2.75 mmol), **2m** (184 μL, 2.75 mmol), **3a** (227 μL, 2.5 mmol) and $AgNO_3$ (43 mg, 0.25 mmol) in CH_3OH (12.5 mL) after flash chromatography (CH_2Cl_2/hexane gradient from 0:100 to 80:20 v/v). **^{1}H NMR** (400 MHz, DMSO-*d₆*) δ 8.05 (br s, 1H), 7.99 – 7.93 (m, 4H), 7.76 – 7.71 (m, 2H), 7.51 – 7.46 (m, 2H), 3.95 (s, 2H), 3.79 (dd, *J* = 6.6, 4.6 Hz, 2H), 3.58 (t, *J* = 5.7 Hz, 2H). ^{13}C NMR (101 MHz, DMSO-d_6) δ 160.01, 150.47, 144.58, 136.42, 133.19, 132.74, 132.53, 130.50, 130.29, 127.49, 119.41, 118.99, 112.51, 109.55, 40.67 (overlapped with the residual solvent signal), 38.32, 33.96. **HRMS:** *m/z* calcd. for $C_{21}H_{15}N_5O$ $[M+H]^+$: 354.1349; found: 354.1350.

(5a*R*,9a*R*)-2-(4-Chlorobenzyl)-4-(4-chlorophenyl)-5a,6,7,8,9,9a-hexahydroimidazo[1,2-*a*]quinoxalin-1(5*H*)-one (9d)

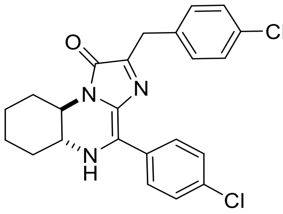

Following *General Procedure A* at rt under argon atm., compound **9c** was obtained as a yellow solid (150 mg, 35%) from **1a** (281 mg, 2 mmol), **2m** (126 mg, 1.1 mmol), **3a** (91 μL, 1 mmol) and $AgNO_3$ (17 mg, 0.1 mmol) in CH_3OH (5 mL) after flash chromatography purification (CH_2Cl_2/hexane gradient from 0:100 to 50:50 v/v). **^{1}H NMR** (400 MHz, DMSO-d_6) δ 7.78 – 7.72 (m, 2H), 7.63 (br s, 1H), 7.57 – 7.51 (m, 2H), 7.35 – 7.30 (m, 2H), 7.30 – 7.25 (m, 2H), 3.78 (d, *J* = 14.9 Hz, 1H), 3.73 (d, *J* = 14.9 Hz, 1H), 3.65 (td, *J* = 11.0, 3.7 Hz, 1H), 3.30 – 3.23 (m, 1H), 2.18 – 2.10 (m, 1H), 1.80 – 1.69 (m, 2H), 1.54 – 1.22 (m, 5H). **^{13}C NMR** (101 MHz, DMSO-d_6) δ 161.50, 151.17, 137.80, 134.91, 133.79, 131.91, 131.25, 131.05, 131.02, 128.69, 128.54, 127.53, 58.30, 56.68, 32.99, 29.28, 27.10, 24.04, 23.94. **HRMS:** *m/z* calcd. for $C_{23}H_{22}Cl_2N_3O^+$ $[M+H]^+$: 426.1134; found: 426.1136.

8-(Benzo[d][1,3]dioxol-5-yl)-2-(benzo[d][1,3]dioxol-5-ylmethyl)-6,7-dihydroimidazo[1,2-*a*]pyrazin-3(5*H*)-one (9e)

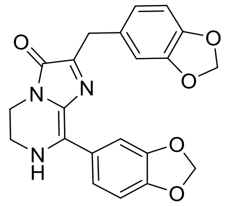

Following *General Procedure A* at rt under argon atm., compound **9e** was obtained as a bright yellow solid (230 mg, 12%) from **1k** (826 mg, 5.5 mmol), **2m** (367 μL, 5.5 mmol), **3a** (454 μL, 5 mmol) and $AgNO_3$ (85 mg, 0.5 mmol) in CH_3OH (25 mL) after flash chromatography purification (MeOH/CH_2Cl_2 gradient from 0:100 to 5:100 v/v). **^{1}H NMR** (400 MHz, $CDCl_3$) δ 7.29 (d, *J* = 1.8 Hz, 1H), 7.20 (dd, *J* = 8.2, 1.8 Hz, 1H), 6.91 (d, *J* = 1.7 Hz, 1H), 6.86 (d, *J* = 8.2 Hz, 1H, overlapped), 6.85 (dd, *J* = 7.9, 1.7 Hz, 1H, overlapped) 6.71 (d, *J* = 7.9 Hz, 1H), 6.01 (s, 2H), 5.88 (s, 2H), 5.02 (t, *J* = 3.5 Hz, 1H), 3.88 (s, 2H), 3.88 – 3.85 (m, 2H), 3.63 – 3.57 (m, 2H). **^{13}C NMR** (101 MHz, $CDCl_3$) δ 160.61, 153.27, 149.62, 148.01, 147.52, 146.03, 133.14, 131.58, 127.14, 125.76, 123.20, 122.07, 109.82, 109.68, 108.57, 108.19, 101.66, 100.77, 40.58, 38.48, 33.93. **HRMS:** *m/z* calcd. for $C_{21}H_{18}N_3O_5$ $[M+H]^+$: 392.1241; found: 392.1241. Compound **9e** contains residual solvent from chromatography. The connectivity of compound **9e** was further studied with bidimensional NMR spectroscopy (COSY).

2-(3,4,5-Trimethoxybenzyl)-8-(3,4,5-trimethoxyphenyl)-6,7-dihydroimidazo[1,2-*a*]pyrazin-3(5*H*)-one (9f)

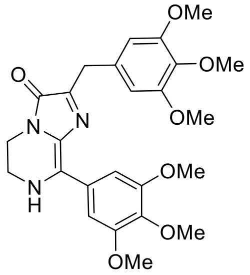

Following *General Procedure A* at rt under argon atm, compound **9f** was obtained as a greenish solid (70 mg, 14%) from **1i** (215 mg, 1.1 mmol), **2m** (73 µL, 1.1 mmol), **3a** (91 µL, 1 mmol) and $AgNO_3$ (17 mg, 0.1 mmol) in CH_3OH (5 mL) after flash chromatography purification eluting with (CH_2Cl_2/hexane gradient from 0:100 to 80:20 v/v). **^{1}H NMR** (400 MHz, DMSO-*d$_6$*) δ 7.80 (t, *J* = 3.3 Hz, 1H), 7.15 (s, 2H), 6.63 (s, 2H), 3.77 – 3.74 (m, 10H; complex signal), 3.71 (s, 6H), 3.71 (s, 3H), 3.61 (s, 3H), 3.58 – 3.55 (m, 2H). **^{13}C NMR** (101 MHz, DMSO-*d$_6$*) δ 159.92, 153.10, 152.82, 149.96, 139.51, 136.39, 134.57, 134.55, 127.14, 126.28, 107.57, 106.88, 60.60, 60.41, 56.30, 56.23, 40.67 (overlapped with residual solvent signal), 38.37, 34.33. **HRMS:** *m/z* calcd. for $C_{25}H_{30}N_3O_7^+$ [M+H]$^+$: 484.2078; found: 484.2081.

Compound **9f** contains residual solvent from chromatography.

(*Z*)-5-(benzo[*d*][1,3]dioxol-5-ylmethylene)-3-(2-(((*E*)-benzo[*d*][1,3]dioxol-5-ylmethylene)amino)ethyl)-3,5-dihydro-4*H*-imidazol-4-one (9e-imine)

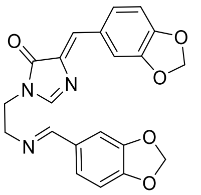

Following *General Procedure A* at rt under argon atm., compound **9e-imine** was obtained as a light brown solid (200 mg, 51%) from **1k** (300 mg, 2 mmol), **2m** (73 µL, 1.1 mmol), **3a** (91 µL, 1 mmol) and $AgNO_3$ (17 mg, 0.1 mmol) in CH_3OH (5 mL). The product precipitated from the reaction mixture and was isolated by filtration under reduced pressure. **^{1}H NMR** (400 MHz, DMSO-*d$_6$*) δ 8.20 (br s, 1H), 8.13 (d, *J* = 1.6 Hz, 1H), 7.98 (d, *J* = 1.6 Hz, 1H), 7.61 (dd, *J* = 8.2, 1.7 Hz, 1H), 7.29 (d, *J* = 1.5 Hz, 1H), 7.17 (dd, *J* = 8.1, 1.6 Hz, 1H), 7.05 (d, *J* = 1.7 Hz, 1H), 7.00 (d, *J* = 8.1 Hz, 1H), 6.96 (d, *J* = 8.0 Hz, 1H), 6.09 (s, 2H), 6.07 (s, 2H), 3.87 – 3.81 (m, 2H), 3.76 – 3.70 (m, 2H). **^{13}C NMR** (101 MHz, DMSO-*d$_6$*) δ 169.56, 162.46, 155.54, 150.14, 149.94, 148.35, 148.20, 137.52, 131.02, 129.35, 128.60, 128.42, 124.94, 111.09, 109.12, 108.68, 106.35, 102.21, 102.01, 58.73, 42.11. **HRMS:** *m/z* calcd. for $C_{21}H_{18}N_3O_5^+$ [M+H]$^+$: 392.1241; found: 392.1245. The connectivity of compound **9e-imine** was further studied with bidimensional NMR spectroscopy (HSQC, HMBC).

(*Z*)-2-(3,4,5-Trimethoxybenzylidene)-8-(3,4,5-trimethoxyphenyl)-5,6,7,8-tetrahydroimidazo[1,2-*a*]pyrazin-3(2*H*)-one (9f')

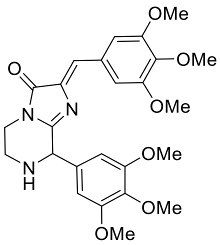

Following *General Procedure A* at rt under argon atm., compound **9f'** was obtained as a pale-yellow solid (200 mg, 30%) from **1i** (550 mg, 2.8 mmol), **2m** (103 µL, 1.54 mmol), **3a** (127 µL, 1.4 mmol) and $AgNO_3$ (24 mg, 0.14 mmol) in CH_3OH (8 mL, 0.175 M). The product precipitated from the reaction mixture and was isolated by filtration under reduced pressure. **^{1}H NMR** (400 MHz, DMSO-*d$_6$*) δ 7.56 (s, 2H), 6.98 (s, 1H), 6.86 (s, 2H), 4.92 (d, *J* = 5.3 Hz, 1H), 3.75 (s, 6H), 3.68 (s, 3H), 3.65 (s, 3H), 3.64 (s, 6H), 3.63 – 3.59 (m, 1H), 3.51 – 3.42 (m, 1H), 3.29 – 3.23 (m, 1H), 3.10 – 2.99 (m, 1H). **HRMS**: *m/z* calcd. for $C_{25}H_{30}N_3O_7^+$ [M+H]$^+$: 484.2078; found: 484.2082. Compound **9f'** converted to compound **9f** in the NMR tube. As a result, a clean ^{13}C NMR spectrum could not be obtained.

3.5.5. Compound 10

(*Z*)-3-(4-(4-methoxybenzylidene)-5-oxo-1-(pyridin-2-ylmethyl)-4,5-dihydro-1*H*-imidazol-2-yl)benzonitrile (10)

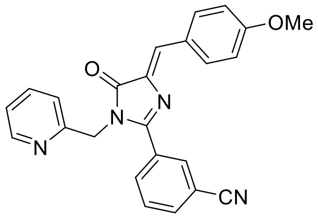

Following *Special Case I*, compound **10** was obtained as a yellow solid (65 mg, 43%) from **5e** (113 mg, 0.38 mmol), $Pd(OAc)_2$ (4.3 mg, 0.02 mmol9), PPh_3 (10 mg, 0.04 mmol), DBU (58 µL, 0.39 mmol), CuBr·DMS (79 mg, 0.39 mmol), 3-iodobenzonitrile (88 mg, 0.39) in DMF (7.7mL). **^{1}H NMR** (400 MHz, DMSO-*d₆*) δ 8.42 (d, *J* = 4.3 Hz, 1H), 8.31 (d, *J* = 8.8 Hz, 2H), 8.27 (t, *J* = 1.6 Hz, 1H), 8.12 (dt, *J* = 8.1, 1.3 Hz, 1H), 7.99 (dt, *J* = 7.8, 1.3 Hz, 1H), 7.75 – 7.65 (m, 2H), 7.30 (d, *J* = 7.8 Hz, 1H), 7.27 (s, 1H), 7.23 (dd, *J* = 7.5, 4.9 Hz, 1H), 7.08 (d, *J* = 8.6 Hz, 2H), 5.10 (s, 2H), 3.84 (s, 3H). NMR data are in agreement with those previously reported in literature.[8]

3.5.6. Compounds 11

Note: Compounds **11a-c** were synthesized in a multicomponent fashion following the first step of the *General Procedure D* and were used in the dehydrogenation step without further purification. Intermediate **11a** was characterized by ^{1}H NMR and HRMS. Intermediates **11b-c** were only checked by LC-MS and directly used in the next step, and therefore their characterization data is not reported.

2-(Benzo[*d*][1,3]dioxol-5-ylmethyl)-5,6,7,8-tetrahydroimidazo[1,2-*a*]pyrimidin-3(2*H*)-one (11a)

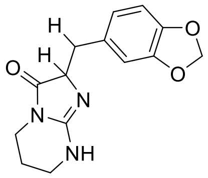

Following the *General Procedure D (i)*, compound **11a** was obtained as a golden solid from **1k** (826 mg, 5.5 mmol), **2o** (459 µL, 5.5 mmol), **3a** (454 µL, 5 mmol) and $AgNO_3$ (85 mg, 0.5 mmol) in MeOH (25 mL). Intermediate **11a** contained traces of compound **12a** and was used in the next step without further purification. **^{1}H NMR** (400 MHz, DMSO-*d₆*) δ 6.80 (d, J = 1.6 Hz, 1H), 6.77 (d, J = 7.9 Hz, 1H), 6.65 (dd, J = 7.9, 1.7 Hz, 1H), 5.94 (q, J = 1.0 Hz, 2H), 3.99 (dd, J = 7.8, 4.3 Hz, 1H), 3.16 – 3.01 (m, 3H), 2.89 (dd, J = 13.8, 4.3 Hz, 1H), 2.60 (dd, J = 13.8, 7.8 Hz, 1H), 1.79 – 1.56 (m, 2H). **HRMS:** *m/z* calcd. for $C_{14}H_{16}N_3O_3^+$ [M+H]$^+$: 274.1186; found: 274.1181.

5-(Benzo[*d*][1,3]dioxol-5-ylmethyl)-3-methyl-2-(methylamino)-3,5-dihydro-4*H*-imidazol-4-one (11b)

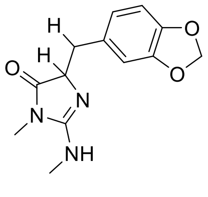

Following the *General Procedure D (i)*, intermediate **11b** was obtained as a light brown syrup from **1k** (165 mg, 1.1 mmol), **2i** (1 mL, 10 mmol), **3a** (91 µL, 1 mmol) and $AgNO_3$ (17 mg, 0.1 mmol) in MeOH (5 mL). Intermediate **11b** contained traces of compound **11b** and was used in the next step without further purification. Intermediate **11b** was only checked by LC-MS before being subjected to the dehydrogenation protocol.

tert-Butyl 3-((1-methyl-2-(methylamino)-5-oxo-4,5-dihydro-1*H*-imidazol-4-yl)methyl)-1*H*-indole-1-carboxylate (11c)

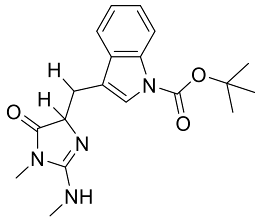

Following the *General Procedure D (i),* intermediate **11c** was obtained as a dark brown syrup from **1n** (270 mg, 1.1 mmol), **2i** (1 mL, 10 mmol), **3a** (91 μL, 1 mmol) and $AgNO_3$ (17 mg, 0.1 mmol) in MeOH (5 mL). Intermediate **11c** contained traces of compound **11c** and was used in the next step without further purification. Intermediate **11c** was only checked by LC-MS before being subjected to the dehydrogenation protocol.

Note: Compounds **11d-e** and **11j** were synthesized following the first step of *General Procedure E* and were characterized before being subjected to the dehydrogenation protocol.

5-(Benzo[*d*][1,3]dioxol-5-ylmethyl)-3-methyl-2-(pyrrolidin-1-yl)-3,5-dihydro-4*H*-imidazol-4-one (11d)

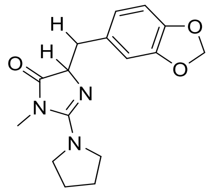

Following the *General Procedure (i)* at rt, compound **11d** was obtained as a pale brown solid (1.06 g, 100%) from **5i** (806 mg, 3.5 mmol) and **Nu-1** (578 μL, 7 mmol, 2 eq.) in CH_3CN (35 mL). The solvent and excess pyrrolidine were evaporated, and the residue was triturated with CH_2Cl_2 / hexanes to afford the pure compound **11d** without further purification. **^{1}H NMR** (500 MHz, $CDCl_3$) δ 6.75 (t, *J* = 1.0 Hz, 1H), 6.66 (d, *J* = 1.0 Hz, 2H), 5.89 – 5.87 (m, 2H), 4.22 (t, *J* = 5.3 Hz, 1H), 3.45 – 3.38 (m, 2H), 3.38 – 3.32 (m, 2H), 3.07 (dd, *J* = 13.6, 5.0 Hz, 1H), 2.96 (s, 3H), 2.94 (dd, *J* = 13.5, 5.6 Hz, 1H), 1.93 – 1.86 (m, 4H). **^{13}C NMR** (101 MHz, $CDCl_3$) δ 182.03, 157.96, 146.92, 146.06, 130.10, 122.93, 110.43, 107.62, 100.67, 66.29, 48.71, 37.59, 28.62, 25.35. **HRMS:** *m/z* calcd. for $C_{16}H_{20}N_3O_3^+$ $[M+H]^+$: 302.1499; found: 302.1506.

The connectivity of compound **11d** was further studied with bidimensional NMR spectroscopy (**COSY**, **HSQC**, **HMBC**).

3-Benzyl-5-(4-chlorobenzyl)-2-(pyrrolidin-1-yl)-3,5-dihydro-4*H*-imidazol-4-one (11e)

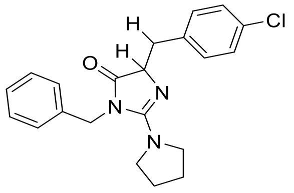

Following the *General Procedure E (i)* at rt, compound **11e** was obtained as a light brown solid (131 mg, 100%) from **5a** (100 mg, 0.34 mmol) and **Nu-1** (278 μL, 3.4 mmol, 10 eq.) in CH_3CN (3.4 mL). The solvent and excess pyrrolidine were evaporated and the residue was triturated with CH_2Cl_2 / hexanes to afford the pure compound **11e** without further purification. **^{1}H NMR** (400 MHz, $CDCl_3$) δ 7.21 (s, 4H), 7.20 – 7.18 (m, 3H), 6.54 – 6.48 (m, 2H), 4.83 (d, *J* = 17.0 Hz, 1H), 4.46 (d, *J* = 16.9 Hz, 1H), 4.43 (t, *J* = 4.7 Hz, 1H), 3.34 – 3.22 (m, 3H), 3.20 – 3.13 (m, 3H), 1.78 – 1.72 (m, 4H). **^{13}C NMR** (101 MHz, $CDCl_3$) δ 182.67, 157.23, 136.10, 134.79, 132.50, 131.81, 128.63, 127.98, 127.19, 125.27, 66.28, 48.31, 44.74, 36.69, 25.24. **HRMS:** *m/z* calcd. for $C_{21}H_{23}ClN_3O^+$ $[M+H]^+$: 368.1524; found: 368.1528.

3-Benzyl-5-((4-chlorophenyl)methyl-*d₂*)-2-(pyrrolidin-1-yl)-3,5-dihydro-4*H*-imidazol-4-one (11e-*d₂*)

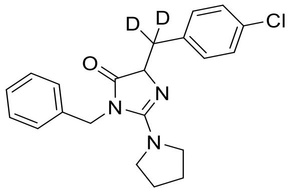

Following the *General Procedure E (i)* at rt, compound **11e-*d₂*** was obtained as a light brown solid (117 mg, 99%) from **5a** (95 mg, 0.32 mmol), **Nu-1** (266 μL, 3.2 mmol, 10 eq.) in CD_3OD (3 mL). The solvent and excess pyrrolidine were evaporated and the residue was triturated with CH_2Cl_2 / hexanes to afford the pure compound **11e-*d₂*** without further purification. **^{1}H NMR** (400 MHz, $CDCl_3$) δ 7.22 (s, 4H), 7.20 – 7.17 (m, 3H), 6.54 – 6.47 (m, 2H), 4.83 (d, *J* = 16.8 Hz, 1H), 4.46 (d, *J* = 17.0 Hz, 1H), 4.42 (s, 1H), 3.34 – 3.25 (m, 2H), 3.21 – 3.12 (m, 2H), 1.77 – 1.72 (m, 4H). **^{13}C NMR** (101 MHz, $CDCl_3$) δ 182.70, 157.24, 136.10, 134.71, 132.48, 131.78, 128.61, 127.96, 127.17, 125.27, 66.18, 48.29, 44.72, 25.23, 10.12. **HRMS:** *m/z* calcd. for $C_{21}H_{21}D_2ClN_3O^+$ $[M+H]^+$: 370.1650; found: 370.1650.

***tert*-Butyl 4-(4-(benzo[*d*][1,3]dioxol-5-ylmethyl)-1-methyl-5-oxo-4,5-dihydro-1*H*-imidazol-2-yl)piperazine-1-carboxylate (11j)**

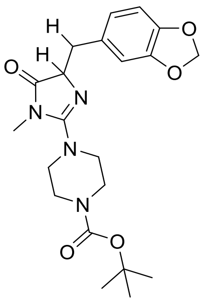

Following the *General Procedure E (i)* at 80 ºC, compound **11j** was obtained as a pale brown solid (1.2 g, 99%) from **5i** (666 mg, 2.9 mmol) and **Nu-4** (1.08 g, 5.8 mmol, 2 eq.) in CH_3CN (29 mL). After reaction completion (LC-MS control) the reaction mixture was concentrated under reduced pressure. The residue was dissolved in CH_2Cl_2 and washed with a saturated aqueous solution of $NaHCO_3$, water, and saturated brine. The organic layer was dried over Na_2SO_4, filtered, and concentrated under reduced pressure to give the crude product. Trituration of the crude product with CH_2Cl_2 / hexanes afforded the title compound **11j** without further purification. **^{1}H NMR** (400 MHz, $CDCl_3$) δ 6.70 (d, *J* = 1.6 Hz, 1H), 6.67 (d, *J* = 8.0 Hz, 3H), 6.63 (dd, *J* = 8.0, 1.6 Hz, 1H), 5.89 (s, 2H), 4.22 (t, *J* = 6.2 Hz, 1H), 3.53 – 3.44 (m, 2H), 3.45 – 3.37 (m, 2H), 3.11 (dd, *J* = 13.6, 5.0 Hz, 1H, overlapped), 3.08 – 3.03 (m, 4H, overlapped), 2.98 (dd, *J* = 13.7, 5.5 Hz, 1H), 2.88 (s, 3H), 1.47 (s, 9H). **^{13}C NMR** (101 MHz, $CDCl_3$) δ 182.90, 160.78, 154.67, 146.98, 146.18, 129.88, 122.81, 110.38, 107.69, 100.76, 80.27, 77.22 (overlapped with residual solvent signal), 67.37, 47.58, 37.17, 28.46, 28.38. **HRMS:** *m/z* calcd. for $C_{21}H_{29}N_4O_5^+$ $[M+H]^+$: 417.2132; found: 417.2129.

3.5.7. Compounds 12-13

Note: Compound **12a-c** were synthesized following the *General Procedure D* without full characterization of their corresponding intermediates **11a-c**. Consequently, the reported yields for compounds **12a-c** are calculated over two steps (MCR + dehydrogenation), in relation to the limiting reactant of the MCR (**3a**).

(*Z*)-2-(benzo[*d*][1,3]dioxol-5-ylmethylene)-5,6,7,8-tetrahydroimidazo[1,2-*a*]pyrimidin-3(2*H*)-one (12a)

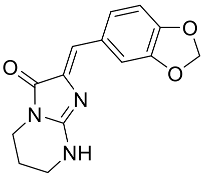

Following the *General Procedure D*, compound **12a** was obtained as a light-yellow solid (290 mg, 22% over two steps). The crude intermediate **11a** (480 mg, 1.76 mmol) was subjected to the dehydrogenation protocol with TEMPO (550 mg, 3.52 mmol) in $CHCl_3$ (18 mL) to afford the title compound **12a**. **^{1}H NMR** (400 MHz, DMSO-*d₆*) δ 8.30 (s, 1H), 7.89 (d, *J* = 1.6 Hz, 1H), 7.37 (dd, *J* = 8.5, 1.6 Hz, 1H), 6.89 (d, *J* = 8.1 Hz, 1H), 6.28 (s, 1H), 6.00 (s, 2H), 3.52 (t, *J* = 5.8 Hz, 2H), 3.30 – 3.25 (m, 2H), 1.96 – 1.87 (m, 2H). **^{13}C NMR** (101 MHz, DMSO-*d₆*) δ 168.66, 156.07, 147.20, 146.44, 138.95, 130.54, 124.80, 111.34, 109.33, 108.25, 100.98, 38.26, 37.40, 20.61. **HRMS:** *m/z* calcd.for $C_{14}H_{14}N_3O_3^+$ $[M+H]^+$: 272.1030; found: 272.1032.

(*Z*)-5-(Benzo[*d*][1,3]dioxol-5-ylmethylene)-3-methyl-2-(methylamino)-3,5-dihydro-4*H*-imidazol-4-one (12b)

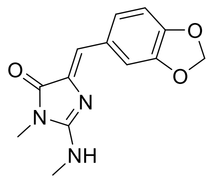

Following the *General Procedure D*, compound **12b** was obtained as a yellow solid (120 mg, 46% over two steps). The crude intermediate **11b** (210 mg, 0.8 mmol) was subjected to the dehydrogenation protocol with TEMPO (250 mg, 1.6 mmol) in $CHCl_3$ (8 mL) to afford the title compound **12b**. **^{1}H NMR** (400 MHz, DMSO-d_6) δ 7.98 (d, *J* = 1.6 Hz, 1H), 7.62 (q, *J* = 4.7 Hz, 1H), 7.43 (dd, *J* = 8.2, 1.6 Hz, 1H), 6.92 (d, *J* = 8.1 Hz, 1H), 6.37 (s, 1H), 6.03 (s, 2H), 3.03 (s, 3H), 2.97 (d, *J* = 4.6 Hz, 3H). NMR data are in agreement with those previously reported in literature.[21]

***tert*-Butyl (*Z*)-3-((1-methyl-2-(methylamino)-5-oxo-1,5-dihydro-4*H*-imidazol-4-ylidene)methyl)-1*H*-indole-1-carboxylate (12c)**

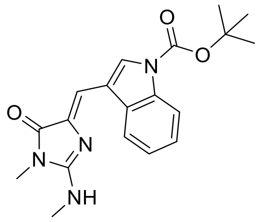

Following the *General Procedure D*, compound **12c** was obtained as a yellow solid (100 mg, 28% over two steps). The crude intermediate **11c** (173 mg, 0.49 mmol) was subjected to the dehydrogenation protocol with TEMPO (153 mg, 0.98 mmol) in $CHCl_3$ (5 mL) to afford the title compound **12c**. **^{1}H NMR** (400 MHz, DMSO-d_6) δ 8.71 (d, *J* = 0.8 Hz, 1H), 8.10 (d, *J* = 8.2 Hz, 1H), 7.96 (d, *J* = 7.9 Hz, 1H), 7.74 (br s, 1H), 7.37 (td, *J* = 7.2, 1.4 Hz, 1H), 7.31 (td, *J* = 7.5, 1.2 Hz, 1H), 6.66 (d, *J* = 0.8 Hz, 1H), 3.06 (s, 3H), 3.02 (s, 3H), 1.65 (s, 9H). **^{13}C NMR** (101 MHz, DMSO-d_6) δ 169.23, 158.96, 149.35, 140.76, 134.90, 129.85, 128.22, 125.17, 123.50, 119.73, 116.65, 115.18, 102.91, 84.29, 28.07, 28.03, 25.95. **HRMS:** *m/z* calcd. for $C_{19}H_{23}N_4O_3^+$ $[M+H]^+$: 355.1765; found: 355.1770.

(*Z*)-5-((1*H*-Indol-3-yl)methylene)-3-methyl-2-(methylamino)-3,5-dihydro-4*H*-imidazol-4-one (12c')

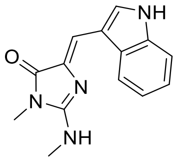

To a solution of **12c** (19 mg, 0.05 mmol) in CH_2Cl_2 (1.1 mL, 0.05 mmol) was added TFA (411 μL, 5.4 mmol, 100 eq.) and the reaction was stirred at rt for 2 h, until analysis by TLC suggested consumption of starting material. The reaction was diluted with EtOAc and it was slowly added a saturated aqueous solution of Na_2CO_3 until pH = 11. The aqueous layer was extracted with EtOAc (3x). The organic layers were combined, washed with saturated brine, dried over Na_2SO_4, filtered, and concentrated under reduced pressure to afford the title compound **12c'** as a yellow solid (13 mg, 94%). **^{1}H NMR** (400 MHz, DMSO-d_6) δ 11.49 (br s, 1H), 8.29 (d, *J* = 2.7 Hz, 1H), 7.95 (d, *J* = 8.1 Hz, 1H), 7.41 (dt, *J* = 8.0, 1.0 Hz, 1H), 7.36 (q, *J* = 4.6 Hz, 1H), 7.14 (ddd, *J* = 8.1, 7.0, 1.3 Hz, 1H), 7.08 (ddd, *J* = 8.0, 7.0, 1.2 Hz, 1H), 6.78 (s, 1H), 3.03 (s, 3H), 2.99 (d, *J* = 4.6 Hz, 3H). NMR data are in agreement with those previously reported in literature.[22]

Note: Compounds **12d-e** were synthesized following the second step of the *General Procedure E* from the corresponding characterized compounds **11d-e**. Compound **12j** was synthesized following the *Special Case I* from the characterized compound **11j**. Compounds **12f-h** and **13** were synthesized following the *General Procedure E* without characterization of the corresponding intermediates **11**. The reported yields for compounds **12f-h** are calculated over two steps (addition + dehydrogenation) in relation to the parent imidazolone **5**.

(*Z*)-5-(Benzo[*d*][1,3]dioxol-5-ylmethylene)-3-methyl-2-(pyrrolidin-1-yl)-3,5-dihydro-4*H*-imidazol-4-one (12d)

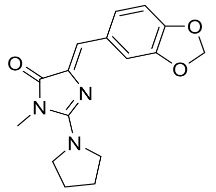

Following the *General Procedure E (ii)*, compound **12d** was obtained as a light brown solid (70 mg, 71%) from **11d** (100 mg, 0.33 mmol) and TEMPO (104 mg, 0.66 mmol) in $CHCl_3$ (3.3 mL) after flash chromatography purification (EtOAc/hexane gradient from 0:100 to 40:60 v/v). **^{1}H NMR** (400 MHz, $CDCl_3$) δ 7.98 (d, *J* = 1.6 Hz, 1H), 7.35 (dd, *J* = 8.1, 1.7 Hz, 1H), 6.81 (d, *J* = 8.1 Hz, 1H), 6.61 (s, 1H), 5.97 (s, 2H), 3.80 – 3.70 (m, 4H), 3.33 (s, 3H), 2.05 – 1.97 (m, 4H). **^{13}C NMR** (101 MHz, $CDCl_3$) δ 171.82, 157.99, 147.67, 147.37, 137.94, 130.57, 126.02, 116.29, 110.25, 108.34, 101.03, 48.81, 28.87, 25.44. **HRMS:** *m/z* calcd. for $C_{16}H_{18}N_3O_3^+$ [M+H]$^+$: 300.1343; found: 300.1351.

(*Z*)-3-Benzyl-5-(4-chlorobenzylidene)-2-(pyrrolidin-1-yl)-3,5-dihydro-4*H*-imidazol-4-one (12e)

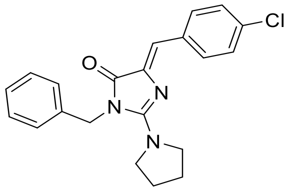

Following the *General Procedure E (ii)*, compound **12e** was obtained as a light brown solid (32 mg, 65%) from **11e** (50 mg, 0.14 mmol) and TEMPO (43 mg, 0.27 mmol) in $CHCl_3$ (1.4 mL) after flash chromatography purification (EtOAc/hexane gradient from 0:100 to 40:60 v/v). **^{1}H NMR** (400 MHz, $CDCl_3$) δ 8.09 – 8.03 (m, 2H), 7.38 – 7.32 (m, 2H, overlapped), 7.35 – 7.24 (m, 3H, overlapped), 7.17 – 7.12 (m, 2H), 6.67 (s, 1H), 5.04 (s, 2H), 3.69 – 3.55 (m, 4H), 1.93 – 1.84 (m, 4H). **^{13}C NMR** (101 MHz, $CDCl_3$) δ 172.02, 157.72, 139.33, 136.71, 134.60, 133.30, 131.87, 128.99, 128.61, 127.59, 125.66, 114.42, 48.71, 44.97, 25.30. **HRMS:** *m/z* calcd. for $C_{21}H_{21}ClN_3O^+$ [M+H]$^+$: 366.1368; found: 366.1366.

(*Z*)-5-(Benzo[*d*][1,3]dioxol-5-ylmethylene)-3-methyl-2-(4-methylpiperazin-1-yl)-3,5-dihydro-4*H*-imidazol-4-one (12f)

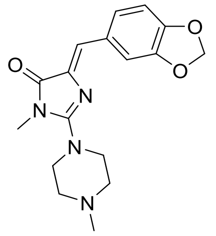

Following the *General Procedure E*, compound **12f** was obtained as a light-yellow solid (40 mg, 40% over two steps). The corresponding intermediate **11** was obtained in quantitative conversion following the *General Procedure E (i)* from **5i** (70 mg, 0.3 mmol) and **Nu-3** (169 μL, 1.5 mmol, 5 eq.) in CH_3CN (3 mL) at 80 ºC for 17 h. The crude intermediate was directly subjected to the dehydrogenation protocol with I_2 (70 mg, 0.27 mmol) / TEMPO (5 mg, 0.03 mmol) in $CHCl_3$ (3 mL) which afforded the title compound **12f** after flash chromatography purification (EtOAc/hexane gradient from 0:100 to 40:60 v/v). **^{1}H NMR** (400 MHz, $CDCl_3$) δ 7.90 (d, *J* = 1.6 Hz, 1H), 7.28 (dd, *J* = 8.2, 1.6 Hz, 1H), 6.75 (d, *J* = 8.1 Hz, 1H), 6.68 (s, 1H), 5.92 (s, 2H), 3.56 – 3.45 (m, 4H), 3.16 (s, 3H), 2.51 (t, *J* = 5.0 Hz, 4H), 2.30 (s, 3H). **^{13}C NMR** (101 MHz, $CDCl_3$) δ 171.96, 160.63, 148.04, 147.77, 137.12, 129.89, 126.77, 119.57, 110.44, 108.39, 101.18, 54.28, 47.32, 46.07, 29.71. **HRMS:** *m/z* calcd. for $C_{17}H_{21}N_4O_3^+$ [M+H]$^+$: 329.1608; found: 329.1610.

(*Z*)-3-Benzyl-5-(4-methoxybenzylidene)-2-morpholino-3,5-dihydro-4*H*-imidazol-4-one (12g)

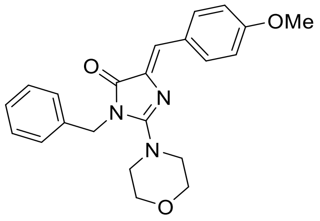

Following the *General Procedure E*, compound **12g** was obtained as a yellow solid (90 mg, 47% over two steps). The corresponding intermediate **11** was obtained in quantitative conversion following the *General Procedure E (i)* from **5c** (150 mg, 0.51 mmol) and **Nu-5** (447 μL, 5.1 mmol, 10 eq.) in CH_3CN (5.1 mL) at 80 ºC for 17 h. The crude intermediate was directly subjected to the dehydrogenation protocol with TEMPO (160 mg, 1.03 mmol) in $CHCl_3$ (5 mL) which afforded the title compound **12g** after flash chromatography purification (EtOAc/hexane gradient from 0:100 to 40:60 v/v). **^{1}H NMR** (400 MHz, $CDCl_3$) δ 8.11 – 8.05 (m, 2H), 7.37 – 7.27 (m, 3H), 7.21 – 7.15 (m, 2H), 6.96 – 6.91 (m, 2H), 6.90 (s, 1H), 4.88 (s, 2H), 3.85 (s, 3H), 3.66 – 3.60 (m, 4H), 3.43 – 3.38 (m, 4H). **^{13}C NMR** (101 MHz, $CDCl_3$) δ 172.18, 160.22, 159.75, 136.12, 136.03, 133.00, 128.96, 128.09, 127.74, 126.21, 120.64, 114.13, 66.09, 55.31, 47.94, 46.04. **HRMS:** *m/z* calcd. for $C_{22}H_{24}N_3O_3^+$ $[M+H]^+$: 378.1812; found: 378.1814.

(*Z*)-5-(Benzo[*d*][1,3]dioxol-5-ylmethylene)-3-methyl-2-(pentylamino)-3,5-dihydro-4*H*-imidazol-4-oneone (12h)

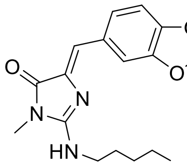

Following the *General Procedure E*, compound **12g** was obtained as a yellow solid (58 mg, 37% over two steps). The corresponding intermediate **11** was obtained in quantitative conversion following the *General Procedure E (i)* from **5i** (115 mg, 0.5 mmol) and **2k** (290 μL, 2.5 mmol, 5 eq.) in CH_3CN (5 mL) at 80 ºC for 17 h. The crude intermediate was directly subjected to the dehydrogenation protocol with MnO_2 (2.2 g, 25 mmol) in $CHCl_3$ (5 mL) which afforded the title compound **12g** after flash chromatography purification (EtOAc/hexane gradient from 0:100 to 40:60 v/v). **^{1}H NMR** (400 MHz, $CDCl_3$) δ 7.98 (d, *J* = 1.6 Hz, 1H), 7.35 (dd, *J* = 8.1, 1.6 Hz, 1H), 6.81 (d, *J* = 8.1 Hz, 1H), 6.65 (s, 1H), 5.98 (s, 2H), 4.49 (br s, 1H), 3.63 – 3.53 (m, 2H), 3.11 (s, 3H), 1.74 – 1.67 (m, 2H), 1.44 – 1.36 (m, 4H), 0.98 – 0.91 (m, 3H). **^{13}C NMR** (126 MHz, $CDCl_3$) δ 170.22, 157.06, 147.71, 147.57, 138.04, 130.26, 126.15, 117.11, 110.35, 108.33, 101.07, 42.00, 29.28, 29.02, 25.08, 22.36, 13.99. **HRMS:** *m/z* calcd. for $C_{17}H_{22}N_3O_3^+$ $[M+H]^+$: 316.1655; found: 316.1656.

(*Z*)-2-Amino-5-(benzo[*d*][1,3]dioxol-5-ylmethylene)-3-methyl-3,5-dihydro-4*H*-imidazol-4-one (12i)

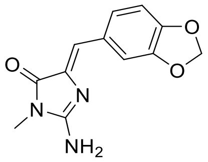

A 0.5-2 mL Biotage microwave vial was charged with **5i** (50 mg, 0.22 mmol, 1 eq.), a 7M NH_3 solution in CH_3OH (1 mL, 7 mmol, 32 eq.) and $CuCl_2$ (29 mg, 0.22 mmol, 1 eq.). The vial was sealed, and the reaction was stirred at 80 ºC for 30 min under μW irradiation. After reaction completion (LC-MS control), the reaction mixture was diluted with CH_2Cl_2 and washed with a saturated aqueous solution of NH_4Cl (5x), water and saturated brine. The organic layer was dried over Na_2SO_4, filtered, and concentrated under reduced pressure to give a dark solid. The crude product was purified *via* neutral Al_2O_3 flash chromatography (EtOAc/hexane gradient from 30:70 to 100:0 v/v) to afford the title compound **12i** as a yellow solid (11 mg, 20 %). **^{1}H NMR** (500 MHz, DMSO-d_6) δ 7.93 (d, *J* = 1.6 Hz, 1H), 7.50 (s, 2H), 7.40 (dd, *J* = 8.1, 1.5 Hz, 1H), 6.91 (d, *J* = 8.0 Hz, 1H), 6.34 (s, 1H), 6.02 (s, 2H), 3.04 (s, 3H). NMR data are in agreement with those previously reported in literature.[21]

The ^{1}H NMR spectra of compound **12i** contains 0.5 eq. of THF which was not removed under high vacuum.

(*Z*)-5-(Benzo[*d*][1,3]dioxol-5-ylmethylene)-3-methyl-2-(piperazin-1-yl)-3,5-dihydro-4*H*-imidazol-4-one (12j)

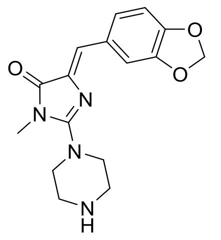

Following the *Special Case I*, compound **12j** was obtained as a yellow solid (295 mg, 60%). The intermediate **Boc-12j** was obtained from **11j** (650 mg, 1.6 mmol) and MnO_2 (6.8 g, 78 mmol) in $CHCl_3$ (15.6 mL) at rt for 17 h. The title compound **12j** was obtained from Boc-deprotection of intermediate **Boc-12j** with TFA (12 mL, 156 mmol) in CH_2Cl_2 (15 mL) at rt for 2 h. Compound **12j** was used in the next step (Synthesis of Compounds **14**) without further purification. **^{1}H NMR** (400 MHz, $CDCl_3$) δ 7.97 (d, *J* = 1.7 Hz, 1H), 7.35 (dd, *J* = 8.1, 1.6 Hz, 1H), 6.82 (d, *J* = 8.1 Hz, 1H), 6.75 (s, 1H), 5.99 (s, 2H), 3.51 (dd, *J* = 6.1, 3.9 Hz, 4H), 3.23 (s, 3H), 3.03 (dd, *J* = 6.2, 3.7 Hz, 5H). **^{13}C NMR** (101 MHz, $CDCl_3$) δ 172.00, 160.89, 148.04, 147.78, 137.14, 129.91, 126.78, 119.63, 110.46, 108.40, 101.18, 48.68, 45.52, 29.74. **HRMS:** *m/z* calcd. for $C_{16}H_{19}N_4O_3^+$ [M+H]$^+$: 315.1452; found: 315.1452.

(*Z*)-5-(Benzo[*d*][1,3]dioxol-5-ylmethylene)-3-methyl-2-(propylthio)-3,5-dihydro-4*H*-imidazol-4-one (13)

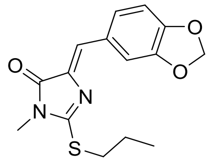

Following the *General Procedure E*, compound **12g** was obtained as a yellow solid (58 mg, 37% over two steps). The corresponding intermediate **11** was obtained in quantitative conversion following the *General Procedure E (i)* from **5i** (50 mg, 0.22 mmol), **Nu-6** (23 μL, 0.25 mmol, 1.2 eq.) in CH_3CN (2 mL) at rt for 3 h. The crude intermediate was directly subjected to the dehydrogenation protocol with I_2 (50 mg, 0.2 mmol) / TEMPO (4 mg, 0.02 mmol) in $CDCl_3$ (2 mL) which afforded the title compound **12g** after flash chromatography purification (EtOAc/hexane gradient from 0:100 to 20:80 v/v). **^{1}H NMR** (400 MHz, $CDCl_3$) δ 8.06 (t, *J* = 1.3 Hz, 1H), 7.37 (dt, *J* = 8.4, 1.1 Hz, 1H), 6.86 (s, 1H), 6.83 (dd, *J* = 8.1, 1.0 Hz, 1H), 6.01 (d, *J* = 1.1 Hz, 2H), 3.32 (td, *J* = 7.2, 1.0 Hz, 2H), 3.15 (d, *J* = 1.0 Hz, 3H), 1.89 (hd, *J* = 7.4, 1.0 Hz, 2H), 1.11 (td, *J* = 7.4, 1.0 Hz, 3H). **^{13}C NMR** (101 MHz, $CDCl_3$) δ 169.94, 163.87, 149.07, 147.99, 137.14, 129.19, 127.98, 123.58, 110.83, 108.44, 101.41, 32.61, 26.53, 22.54, 13.47. **HRMS:** *m/z* calcd. for $C_{15}H_{17}N_2O_3S^+$ [M+H]$^+$: 305.0954; found: 305.0959.

3.5.8. Compounds 14

(3a*S*,4*R*,6a*R*)-4-(5-(4-((*Z*)-4-(Benzo[*d*][1,3]dioxol-5-ylmethylene)-1-methyl-5-oxo-4,5-dihydro-1*H*-imidazol-2-yl)piperazin-1-yl)-5-oxopentyl)tetrahydro-1*H*-thieno[3,4-*d*]imidazol-2(3*H*)-one (14a)

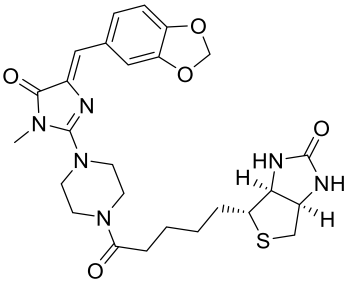

Following the *General Procedure F*, compound **14a** was obtained as a pale yellow solid (30 mg, 70%) from **12j** (25 mg, 0.08 mmol), biotin (23 mg, 0.095 mmol), HATU (36 mg, 0.095 mmol), and DIPEA (55 μL, 0.32 mmol) in DMF (0.8 mL) and CH_2Cl_2 (0.4 mL) after flash chromatography purification (EtOAc/hexane gradient from 0:100 to 30:70 v/v). **^{1}H NMR** (400 MHz, $CDCl_3$) δ 7.93 (d, *J* = 1.6 Hz, 1H), 7.34 (dd, *J* = 8.4, 1.6 Hz, 1H), 6.82 (d, *J* = 8.1 Hz, 1H), 6.79 (s, 1H), 5.99 (s, 2H), 5.79 (s, 1H), 5.10 (s, 1H), 4.53 – 4.45 (m, 1H), 4.36 – 4.28 (m, 1H), 3.84 – 3.74 (m, 2H), 3.71 – 3.63 (m, 2H), 3.63 – 3.54 (m, 2H), 3.48 (t, *J* = 5.2 Hz, 2H), 3.23 (s, 3H), 3.21 – 3.14 (m, 1H), 2.90 (dd, *J* = 12.8, 5.0 Hz, 1H), 2.72 (d, *J* = 12.9 Hz, 1H), 2.41 (t, *J* = 7.4 Hz, 2H), 1.84 – 1.62 (m, 4H, overlapped with water signal), 1.57 – 1.42 (m, 2H). **^{13}C NMR** (101 MHz, $CDCl_3$) δ 171.72, 171.62, 163.36, 160.60, 148.36, 147.84, 136.70, 129.57, 127.08, 120.88, 110.46, 108.47, 101.28, 61.81, 60.11, 55.29, 47.55, 44.83, 40.85, 40.57, 32.59, 29.44, 28.28, 24.96. **HRMS:** *m/z* calcd. for $C_{26}H_{33}N_6O_5S^+$ [M+H]$^+$: 541.2228; found: 541.2227.

(*Z*)-5-(Benzo[*d*][1,3]dioxol-5-ylmethylene)-3-methyl-2-(4-((*R*)-4-((3*S*,5*R*,7*S*,8*S*,9*R*,10*R*,12*R*,13*S*,14*R*,17*S*)-3,7,12-trihydroxy-10,13-dimethylhexadecahydro-1*H*-cyclopenta[*a*]phenanthren-17-yl)pentanoyl)piperazin-1-yl)-3,5-dihydro-4*H*-imidazol-4-one (14b)

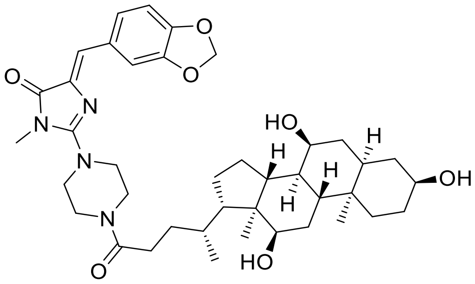

Following the *General Procedure F*, compound **14b** was obtained as a pale yellow solid (30 mg, 70%) from **12j** (25 mg, 0.08 mmol), biotin (23 mg, 0.095 mmol), HATU (36 mg, 0.095 mmol), and DIPEA (55 μL, 0.32 mmol) in DMF (0.8 mL) and CH_2Cl_2 (0.4 mL) after flash chromatography purification (EtOAc/hexane gradient from 0:100 to 30:70 v/v). **^{1}H NMR** (400 MHz, $CDCl_3$) δ 7.94 (d, *J* = 1.6 Hz, 1H), 7.34 (dd, *J* = 8.5, 1.7 Hz, 1H), 6.82 (d, *J* = 8.1 Hz, 1H), 6.80 (s, 1H), 5.99 (s, 2H), 4.01 – 3.96 (m, 1H), 3.87 – 3.83 (m, 1H), 3.80 – 3.75 (m, 2H), 3.70 – 3.64 (m, 2H), 3.60 – 3.54 (m, 2H), 3.49 – 3.44 (m, 2H), 3.24 (s, 3H), 2.50 – 2.40 (m, 1H), 2.33 – 2.16 (m, 4H), 1.98 – 1.65 (m, 15H, complex signal), 1.60 – 1.26 (m, 12H, complex signal), 1.19 – 1.07 (m, 1H), 1.03 (d, *J* = 6.3 Hz, 3H), 0.93 – 1.02 (m, 1H), 0.89 (m, 5H, overlapped signals), 0.70 (s, 3H). **^{13}C NMR** (101 MHz, $CDCl_3$) δ 172.52, 171.65, 160.59, 148.36, 147.84, 136.71, 129.59, 127.09, 120.93, 110.49, 108.46, 101.28, 72.98, 71.93, 68.40, 47.57, 46.89, 46.51, 44.83, 41.90, 41.45, 40.83, 39.71, 39.55, 35.38, 35.24, 34.71, 31.21, 30.53, 29.99, 29.44, 28.33, 27.57, 26.57, 23.24, 22.52, 17.57, 12.57. **HRMS:** *m/z* calcd. for $C_{40}H_{56}N_4O_7^+$ $[M+H]^+$: 705.4222; found: 705.4214.

The spectra contain traces of hexane (from the chromatography) and pentane (used in an attempt to remove the remaining hexane), which were not removed under high vacuum.

3.5.9. Compounds S

(1*E*,1'*E*)-*N*,*N*'-(ethane-1,2-diyl)bis(1-(4-iodophenyl)methanimine) (S7)

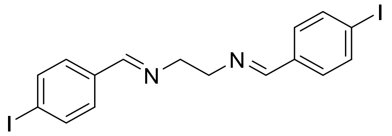

The title compound **S7** was obtained in two different experiments to study the mechanism of formation of compound **9** (see SI, section 3.1).

(i) A mixture of **1c** (464 mg, 2 mmol, 2 eq.), **2m** (73 μl, 1.1 mmol, 1.1 eq.), **3a** (91 μL, 1 mmol, 1 eq.), and $AgNO_3$ (17 mg, 0.1 mmol, 0.1 eq.) in MeOH (5 mL, 0.2 M) under argon atmosphere was stirred in an ultrasonic bath for 2 h. Although the batch was not heated, after 2 h the water temperature was at 65 ºC. After this time a precipitate was formed, and it was filtered washing with cold MeOH. The solid was dried under reduced pressure to afford the title compound **S7** as an off-white solid (230 mg, 47%).

(ii) A mixture of **1c** (464 mg, 2 mmol, 2 eq.), **2m** (73 μl, 1.1 mmol, 1.1 eq.), cyclohexyl isocyanide (124 μL, 1 mmol, 1 eq.), and $AgNO_3$ (17 mg, 0.1 mmol, 0.1 eq.) in MeOH (5 mL, 0.2 M) under argon atmosphere was stirred at room temperature for 17 h. After this time a precipitate was formed, and it was filtered washing with cold MeOH. The solid was dried under reduced pressure to afford the title compound **S7** as a white solid (420 mg, 86%).

^{1}H NMR (400 MHz, DMSO-*d₆*) δ 8.28 (s, 1H), 7.84 – 7.74 (m, 2H), 7.54 – 7.42 (m, 2H), 3.85 (s, 2H). NMR data are in agreement to those previously reported in literature for an analogous compound with a 4-chlorophenyl (1*E*,1'*E*)-*N,N'*-(ethane-1,2-diyl)bis(1-(4-chlorophenyl)methanimine).[23]

2-(4-Iodophenyl)imidazolidine (S8)

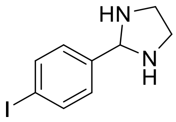

To a mixture of **1c** (464 mg, 2 mmol, 2 eq.), **3a** (91 μL, 1 mmol, 1 eq.), and $AgNO_3$ (17 mg, 0.1 mmol, 0.1 eq.) in MeOH (5 mL, 0.2 M) was added **2n** (73 μL, 1 mmol, 1 eq.) under argon atmosphere and the reaction was stirred at room temperature for 17 h. After reaction completion, the reaction was concentrated under reduced pressure and the residue was subjected to neutral Al_2O_3 flash chromatography (CH_2Cl_2 100 %) to afford the title compound as a yellow oil (268 mg, 97 %). **^{1}H NMR** (500 MHz, DMSO-d_6) δ 7.76 (d, J = 8.4 Hz, 2H), 7.19 (d, J = 8.0 Hz, 2H), 5.36 (s, 1H), 3.23 (s, 6H).

4. Fluorescence Studies

4.1. In vitro Spectroscopy

A representative set of the synthesized compounds were chosen for spectroscopic studies (Figure S25). Absorption spectra were recorded with NanoDrop. Step 0.5 nm. Emission spectra were recorded with Varioskan. (Step: 5 nm, excitation bandwidth: 12 nm, measurement time: 100 ms, dynamic range: automatic). Selected compounds were dissolved in DMSO and were diluted to 100 µM. The spectra were recorded at room temperature. The spectra are represented as means from two independent experiments with n=3 (Figure S26).

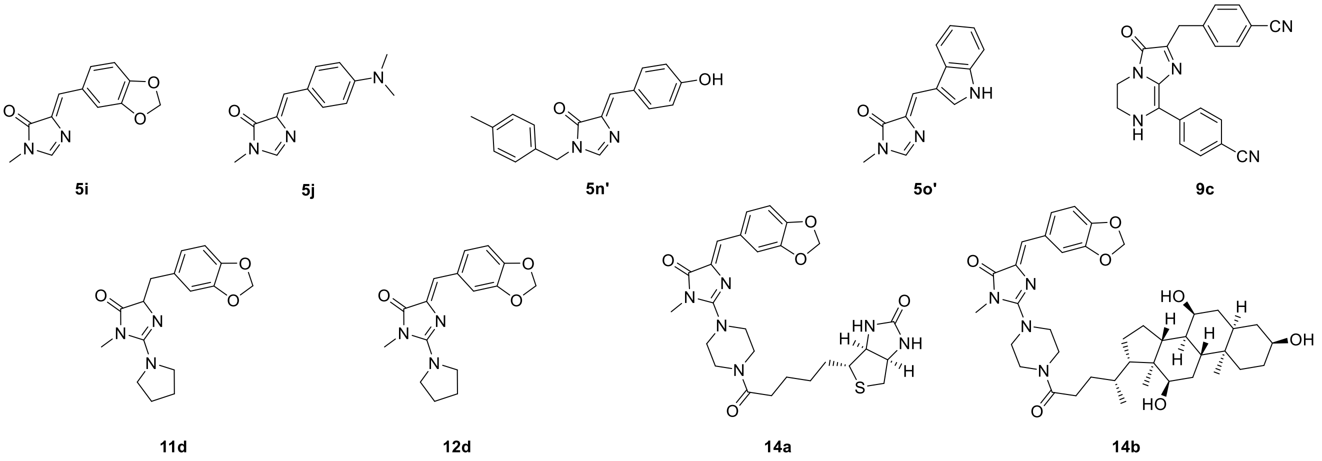

Figure S25. Selected compounds for UV-Vis spectroscopy studies.

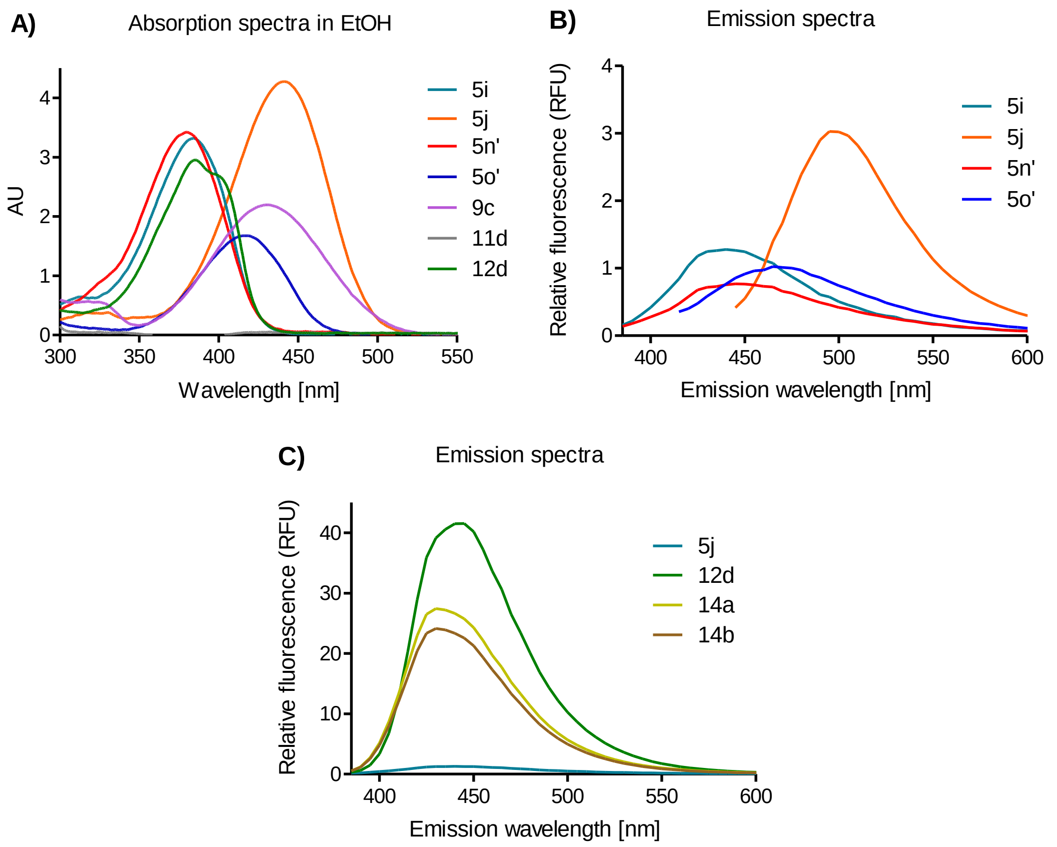

Figure S26. Photophysical properties of the selected compounds (100 µM). A) Absorption spectra. B) Emission spectra in EtOH of compounds **5i** (λ_{exc}: 360 nm), **5j** (λ_{exc}: 420 nm), **5n'** (λ_{exc}: 355 nm), and **5o'** (λ_{exc}: 390 nm). C) Emission spectra in EtOH of compounds **5i**, **12d**, and **14a-b** (λ_{exc}: 360 nm).

Table S8. Absorption and emission maxima values of selected compounds in different solvents (100 μM).

Compound	Absorption maxima wavelength (nm)			Emission maxima wavelength (nm)			Excitation wavelength (nm)
	DMSO	EtOH	PBS	DMSO	EtOH	PBS	
**5i**	385	385	387	450	440	465	360
**5j**	437	441	455	505	495	530	420
**5n'**	383	380	376	445	445	465	355
**5o'**	412	416	414	465	465	450	390
**9c**	439	430	424	610	595	595	400
**12d**	388	386	392	450	445	460	360
**14a**	-	383	-	-	430	-	360
**14b**	-	385	-	-	430	-	360

The fluorescence intensity of compounds **5i** and **12d** was significally increased the presence of phosphatidylcholine (PC)-based lyposome, a viscous hydrophobic environment that restricts the rotation around the aryl-alkene bond (Figure S27B). Moreover, the fluorescence of 2-aminoimidazolone **12d** was reduced at lower pH values (Figure S27A).

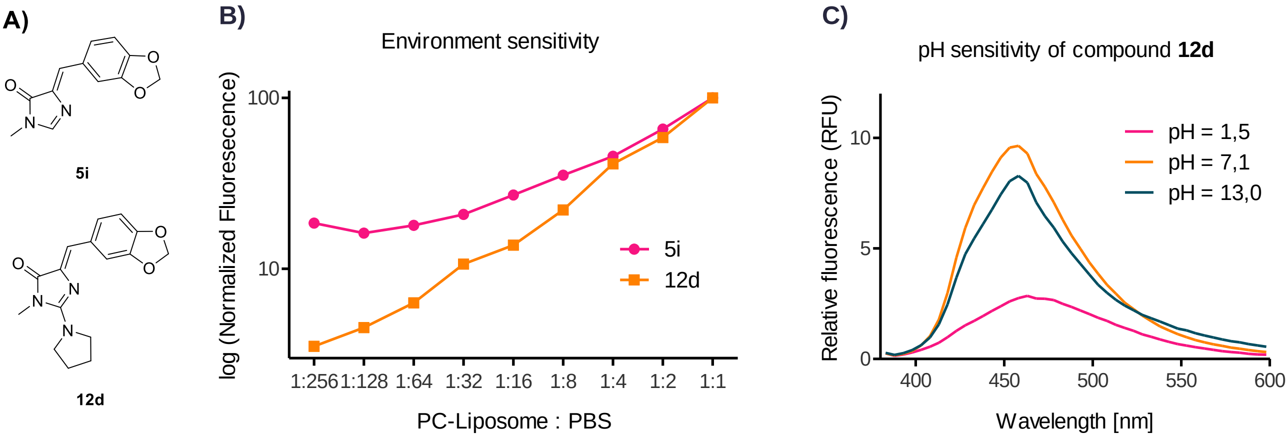

Figure S27. Environment-sensitive fluorescence of compounds 5i and 12d (100 μM). A) Structure of compounds **5i** and **12d**. B) Fluorescence emission at different PC-liposome: PBS ratios (λ_{exc}: 360 nm). C) Fluorescence emission of compound **12d** in 1 :1 mixtures of MeOH : water at different pH values (λ_{exc}: 360 nm).

4.2. Confocal microscopy of MDA MB231 cells

MDA-MB-231 cells were plated at 5,000 cells per well in an 18 well glass bottomed confocal chamber in complete DMEM (DMEM + 10% FBS, Pen/strep, L-glutamine) and incubated at 37 ºC, 5% CO_2 for 24 h. Cells were incubated with compound **12d** (100 μM) for 1h in complete DMEM. Imaged on a Leica SP8 (HC PL APO CS2 63x 1.40 oil, HyD detectors). A minimum of 3 fields of view were captured per condition and repeated independently three times. Images were processed using the Leica Application Suite X (LAS X) software.

5. Crystallographic Data

5.1. Compound 5h

Crystals suitable for single X-ray diffraction were grown by slow diffusion of Et_2O into a CH_2Cl_2 solution of the pure compound **5h** through layering.

Table S9. Crystal data and structure refinement of compound **5h.**

**CCDD Number**	**2240654**[25]
**Crystal data**	
Chemical formula	$C_{24}H_{20}N_2O_2$
M_r	368.42
Crystal system, space group	Monoclinic, $P2_1$
Temperature (K)	180
a, *b*, *c* (Å)	5.9189 (7), 7.4198 (8), 21.621 (2)
β (°)	97.764 (4)
V ($Å^3$)	940.84 (18)
Z	2
Radiation type	Cu *K*α
μ (mm^{-1})	0.66
Crystal size (mm)	0.4 × 0.15 × 0.05
**Data collection**	
Diffractometer	Bruker *APEX*-II CCD
Absorption correction	Multi-scan<br>*SADABS2016*/2 (Bruker,2016/2) was used for absorption correction. wR2(int) was 0.1552 before and 0.0979 after correction. The Ratio of minimum to maximum transmission is 0.7054. The λ/2 correction factor is Not present.
T_{min}, T_{max}	0.532, 0.754
No. of measured, independent and observed [$I > 2\sigma(I)$] reflections	16392, 3583, 3341
R_{int}	0.067
$(\sin \theta/\lambda)_{max}$ ($Å^{-1}$)	0.620
**Refinement**	
$R[F^2 > 2\sigma(F^2)]$, $wR(F^2)$, S	0.043, 0.114, 1.04
No. of reflections	3583
No. of parameters	255
No. of restraints	1
H-atom treatment	H-atom parameters constrained
$\Delta\rho_{max}$, $\Delta\rho_{min}$ (e $Å^{-3}$)	0.15, −0.16
Absolute structure	Refined as an inversion twin.
Absolute structure parameter	−0.1 (3)

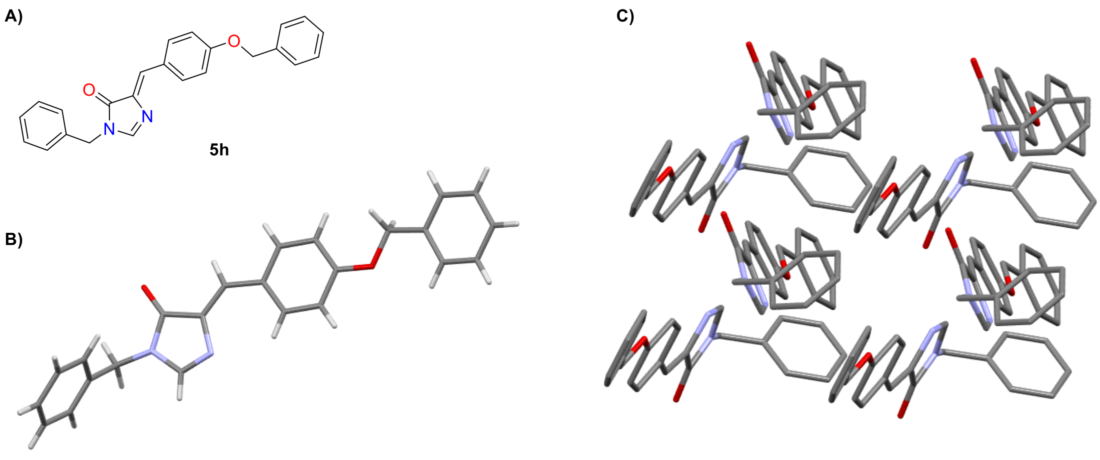

Figure S28 A) Structure of **5h**. B) Molecular structure. C) Packing arrangement of **5i** in the solid-state. Hyrodgen atoms were ommited for clarity.

5.2. Compound 8a

Crystals suitable for single X-ray diffraction were grown by slow diffusion of Et_2O into a $CHCl_3$ solution of the pure compound **8a** through layering.

Table S10. Crystal data and structure refinement of compound **8a.**

**CCDC number**	**2240652**[25]
**Crystal data**	
Chemical formula	$C_{34}H_{26}Cl_2N_4O_2$
M_r	593.49
Crystal system, space group	Monoclinic, $P2_1/c$
Temperature (K)	180
a, *b*, *c* (Å)	15.815 (2), 7.4619 (10), 12.7516 (16)
β (°)	103.016 (9)
V (Å^3)	1466.2 (3)
Z	2
Radiation type	Cu $K\alpha$
μ (mm^{-1})	2.30
Crystal size (mm)	0.1 × 0.08 × 0.01
**Data collection**	
Diffractometer	Bruker *APEX*-II CCD
Absorption correction	Multi-scan *SADABS2016*/2 (Bruker,2016/2) was used for absorption correction. wR2(int) was 0.1374 before and 0.0955 after correction. The Ratio of minimum to maximum transmission is 0.7157. The λ/2 correction factor is Not present.
T_{min}, T_{max}	0.538, 0.752
No. of measured, independent and observed [$I > 2\sigma(I)$] reflections	20711, 2182, 1356
R_{int}	0.094
θ_{max} (°)	60.1
$(\sin \theta/\lambda)_{max}$ (Å^{-1})	0.562
**Refinement**	
$R[F^2 > 2\sigma(F^2)]$, $wR(F^2)$, S	0.065, 0.225, 1.10
No. of reflections	2182
No. of parameters	190
H-atom treatment	H-atom parameters constrained
$\Delta\rho_{max}$, $\Delta\rho_{min}$ (e Å^{-3})	0.30, −0.36

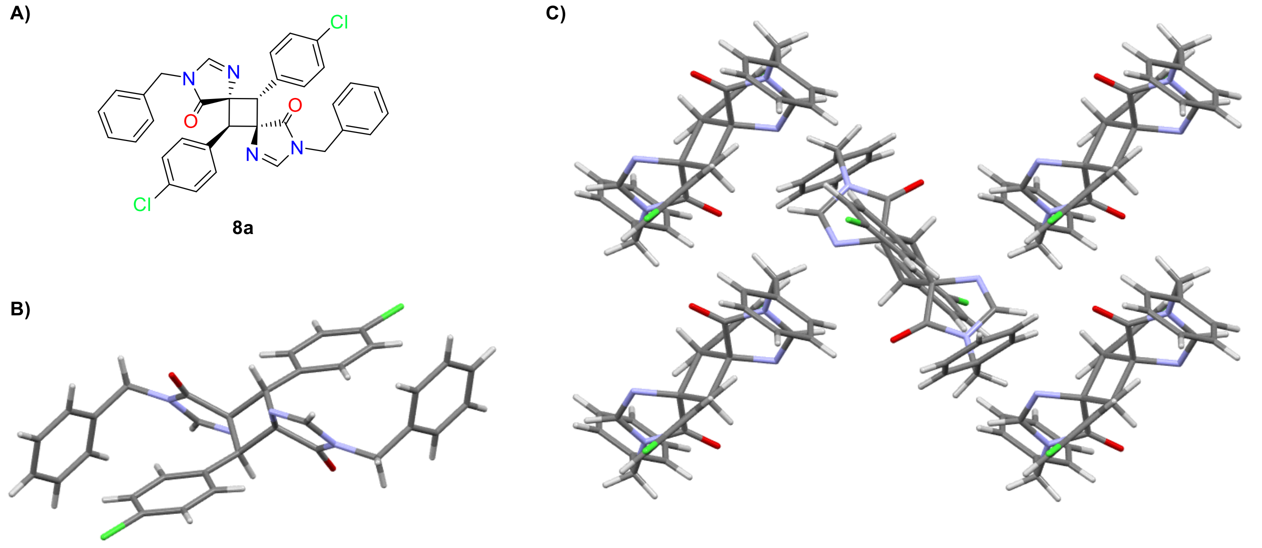

Figure S29. A) Structure of **8a**. B) Molecular structure. C) Packing arrangement of 8a in the solid-state.

6. References

[1] G. Jones, in *Organic Reactions*, John Wiley & Sons, Inc., Hoboken, **2011**, pp. 204–599.

[2] M. B. Smith, J. March, in *March's Advanced Organic Chemistry: Reactions, Mechanisms, and Structure, 5th ed.*, John Wiley & Sons, Inc., Hoboken, **2006**, p. 1174.

[3] V. Voliani, R. Bizzarri, R. Nifosì, S. Abbruzzetti, E. Grandi, C. Viappiani, F. Beltram, *J. Phys. Chem. B.* **2008**, *112*, 10714–10722.

[4] A. Clemenceau, Q. Wang, J. Zhu, *Org. Lett.* **2017**, *19*, 4872–4875.

[5] S. Kojima, T. Hirano, H. Niwa, M. Ohashi, S. Inouye, F. I. Tsuji, *Tetrahedron Lett.* **1997**, *38*, 2875–2878.

[6] A. Shaabani, A. Maleki, H. Mofakham, H. R. Khavasi, *J. Comb. Chem.* **2008**, *10*, 323–326.

[7] V. Kysil, A. Khvat, S. Tsirulnikov, S. Tkachenko, C. Williams, M. Churakova, A. Ivachtchenko, *Eur. J. Org. Chem.* **2010**, *2010*, 1525–1543.

[8] W. Wei, L. Wang, P. Bao, Y. Shao, H. Yue, D. Yang, X. Yang, X. Zhao, H. Wang, *Org. Lett.* **2018**, *20*, 7125–7130.

[9] M. Muselli, C. Baudequin, C. Perrio, C. Hoarau, L. Bischoff, *Chem. Eur. J.* **2016**, *22*, 5520–5524.

[10] M. Trobe, T. Schreiner, M. Vareka, S. Grimm, B. Wölfl, R. Breinbauer, *Eur. J. Org. Chem.* **2022**, *2022*, e202101280.

[11] F. Ling, D. Cheng, T. Liu, L. Liu, Y. Li, J. Li, W. Zhong, *Green Chem.* **2021**, *23*, 4107–4113.

[12] J. J. Ciardiello, W. R. J. D. Galloway, C. J. O'connor, H. F. Sore, J. E. Stokes, Y. Wu, D. R. Spring, *Tetrahedron* **2016**, *72*, 3567–3578.

[13] S. Semwal, J. Choudhury, *Angew. Chem. Int. Ed.* **2017**, *56*, 5556–5560; *Angew. Chem*. **2017**, *129*, 5648–5652.

[14] J. M. Rodríguez, M. Dolors Pujol, *Tetrahedron Lett.* **2011**, *52*, 2629–2632.

[15] Y. Imada, O. Shibata, S.-I. Murahashi, *J Organomet Chem* **1993**, *451*, 183–194.

[16] N. Scornet, S. Delarue-Cochin, M. E. Azoury, M. Mignon, J.-A. Chemelle, E. Nony, B. Maillère, R. Terreux, M. Pallardy, D. Joseph, *Bioconjug Chem* **2016**, *27*, 2629–2645.

[17] Y. Pan, Z. Luo, J. Han, X. Xu, C. Chen, H. Zhao, L. Xu, Q. Fan, J. Xiao, *Adv. Synth. Catal.* **2019**, *361*, 2301–2308.

[18] N. Elders, R. F. Schmitz, F. J. J. de Kanter, E. Ruijter, M. B. Groen, R. V. A. Orru, *J Org Chem* **2007**, *72*, 6135–6142.

[19] H. Ohta, Y. Uozumi, Y. M. A. Yamada, *Chem Asian J* **2011**, *6*, 2545–2549.

[20] H. von Dieter, U. Schollkopf, *Liebigs Ann. Chem* **1972**, *763*, 1–16.

[21] M. M. Moro´n, C. Burgos, J. Alvarez-Builla, A. Salgado, M. E. G. Mosquera, J. J. Vaquero, *Chem. Commun* **2012**, *48*, 9171–9173.

[22] M. Debdab, F. Carreaux, S. Renault, M. Soundararajan, O. Fedorov, P. Filippakopoulos, O. Lozach, L. Babault, T. Tahtouh, B. Baratte, Y. Ogawa, M. Hagiwara, A. Eisenreich, U. Rauch, S. Knapp, L. Meijer, J.-P. Bazureau, *J Med Chem* **2011**, *54*, 4172–4186.

[23] G. Guella, I. Mancini, H. Zibrowius, F. Pietra, *Helv Chim Acta* **1989**, *72*, 1444–1450.

[24] A. M. Mansour, K. Radacki, R. M. Khaled, M. H. Soliman, N. T. Abdel-Ghani, *J. Biol. Inorg. Chem*. **2021**, *26*, 135–147.

[25] Compound **5h**: Rodolfo Lavilla; 2023; CCDC 2240654; Experimental Crystal Structure Determination; DOI: 10.5517/ccdc.csd.cc2f6l5f. Compound **8a**: Rodolfo Lavilla; 2023; CCDC 2240652; Experimental Crystal Structure Determination; DOI: 10.5517/ccdc.csd.cc2f6l3c.

7. Author Contributions

Conceptualization: P.N.R., O.G., and R.L. Funding acquisition: M.V. and R.L. Investigation: Organic Chemistry: P.N.R., O.G., A.M.S., and R.L. Fluorescence Studies and Bioimaging: P.N.R., O.G., S.B., and M.V. Supervision: O.G., M.V., and R.L. Writing of original draft: P.N.R., O.G., and R.L. Review and editing: all authors.

8. Copies of NMR Spectra

8.1. Non-Commercial Starting Materials

4-((*tert*-Butyldimethylsilyl)oxy)benzaldehyde (1f)

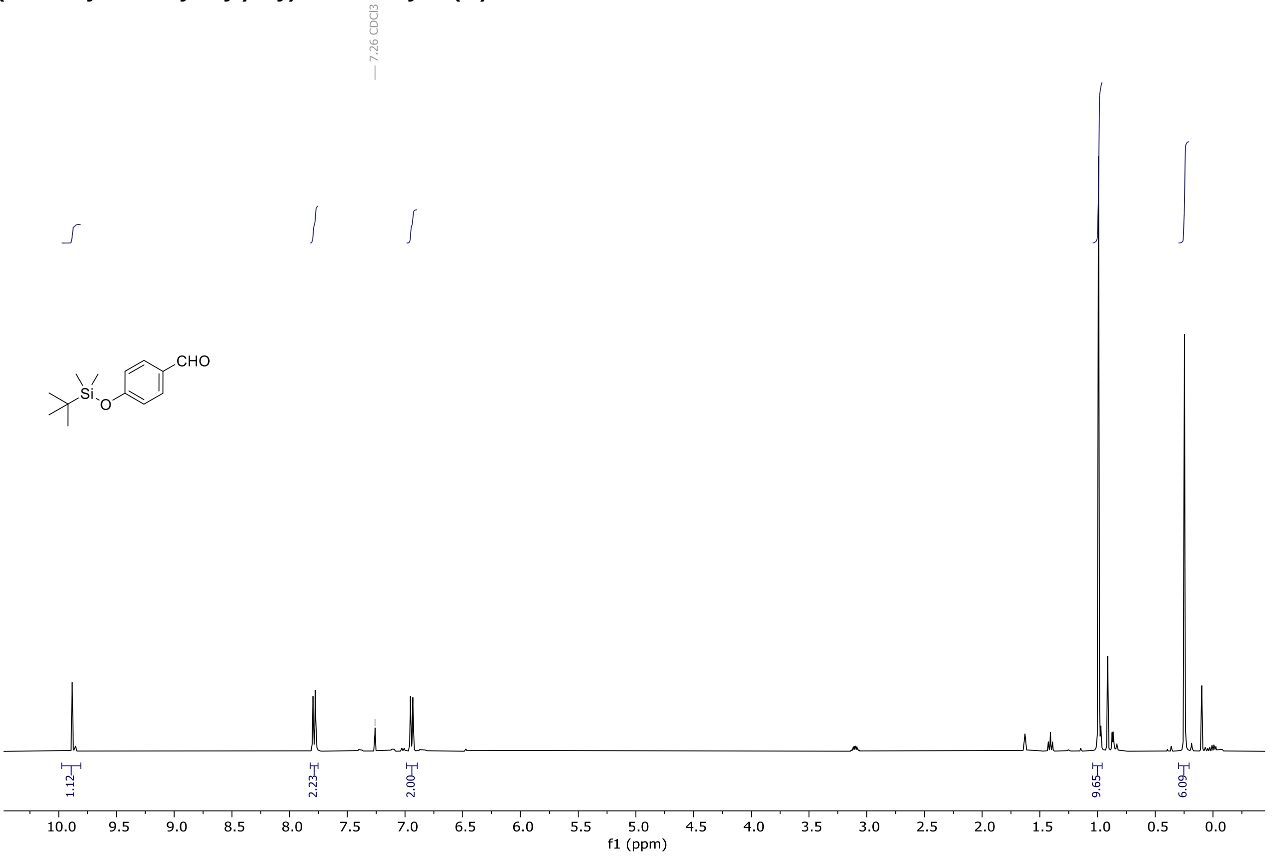

2-(Allyloxy)benzaldehyde (1g)

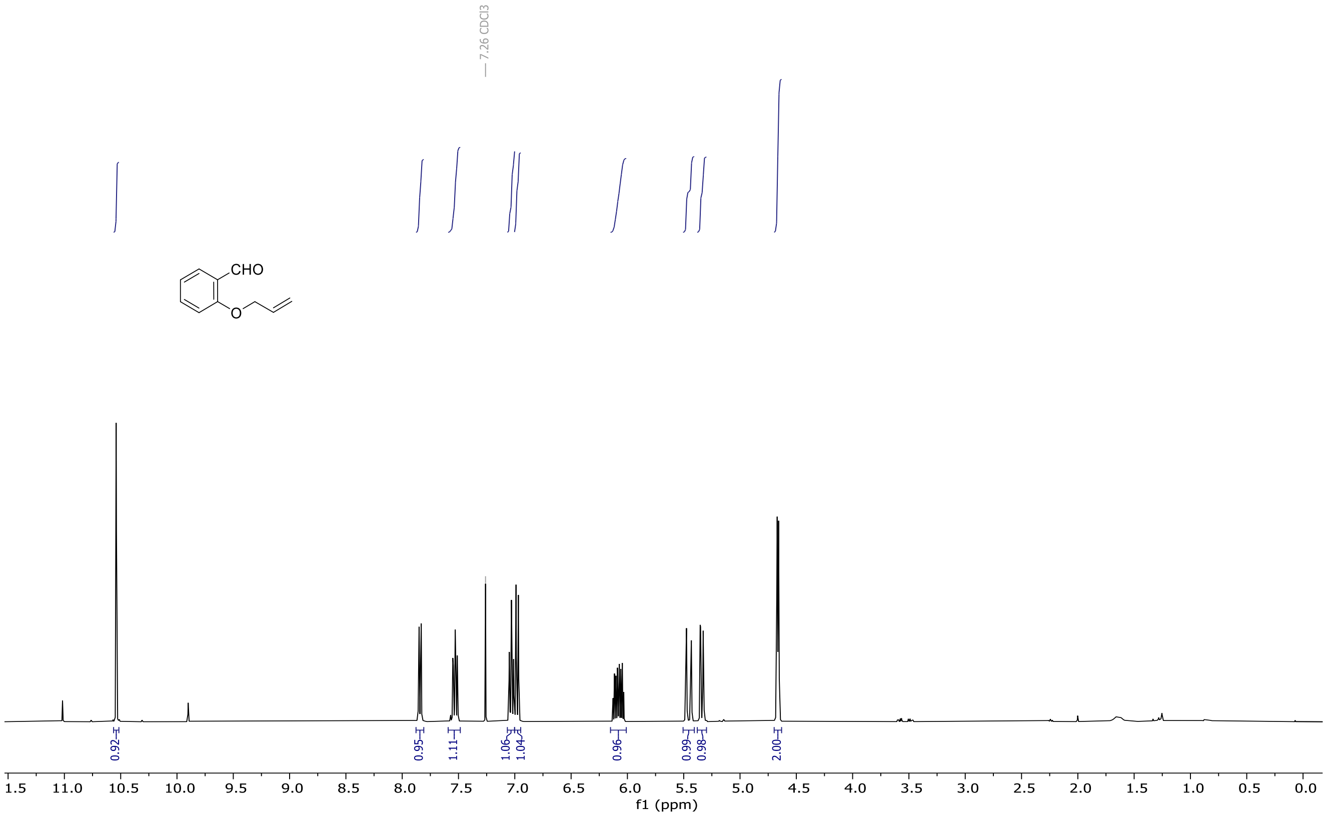

tert-Butyl 3-formyl-1H-indole-1-carboxylate (1m)

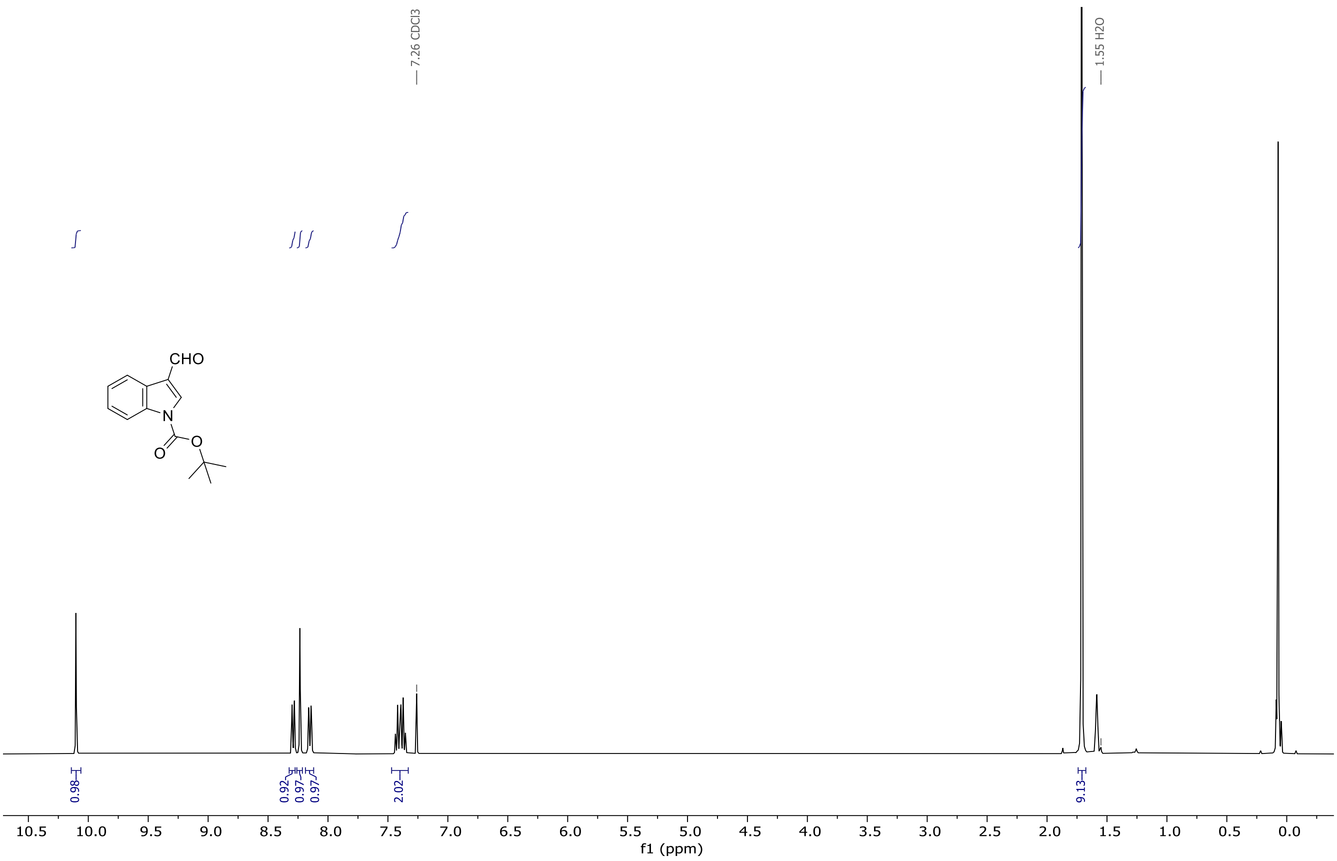

1-Tosyl-1H-indole-3-carboxaldehyde (1n)

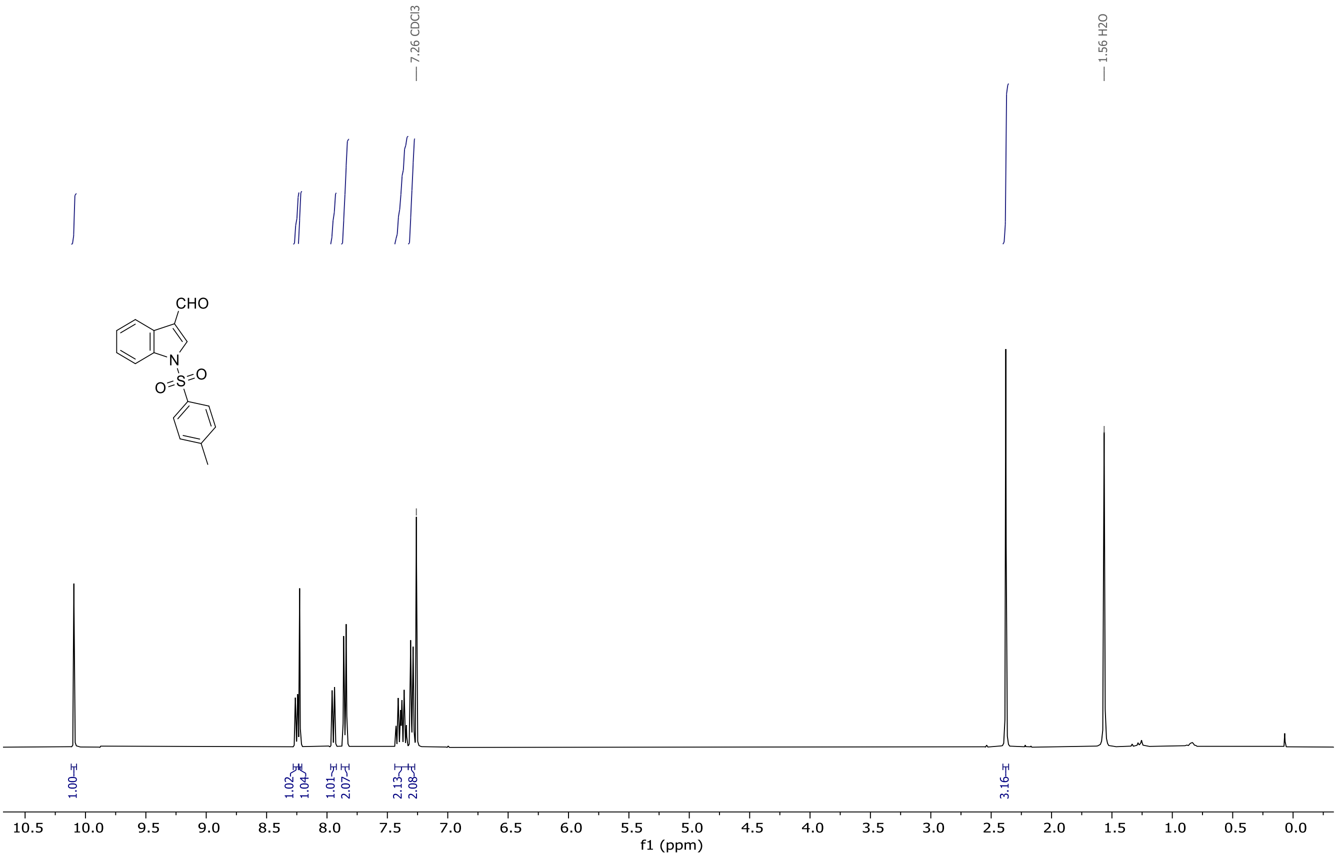

Benzylamine-*d*$_2$ (2a-*d*$_2$)

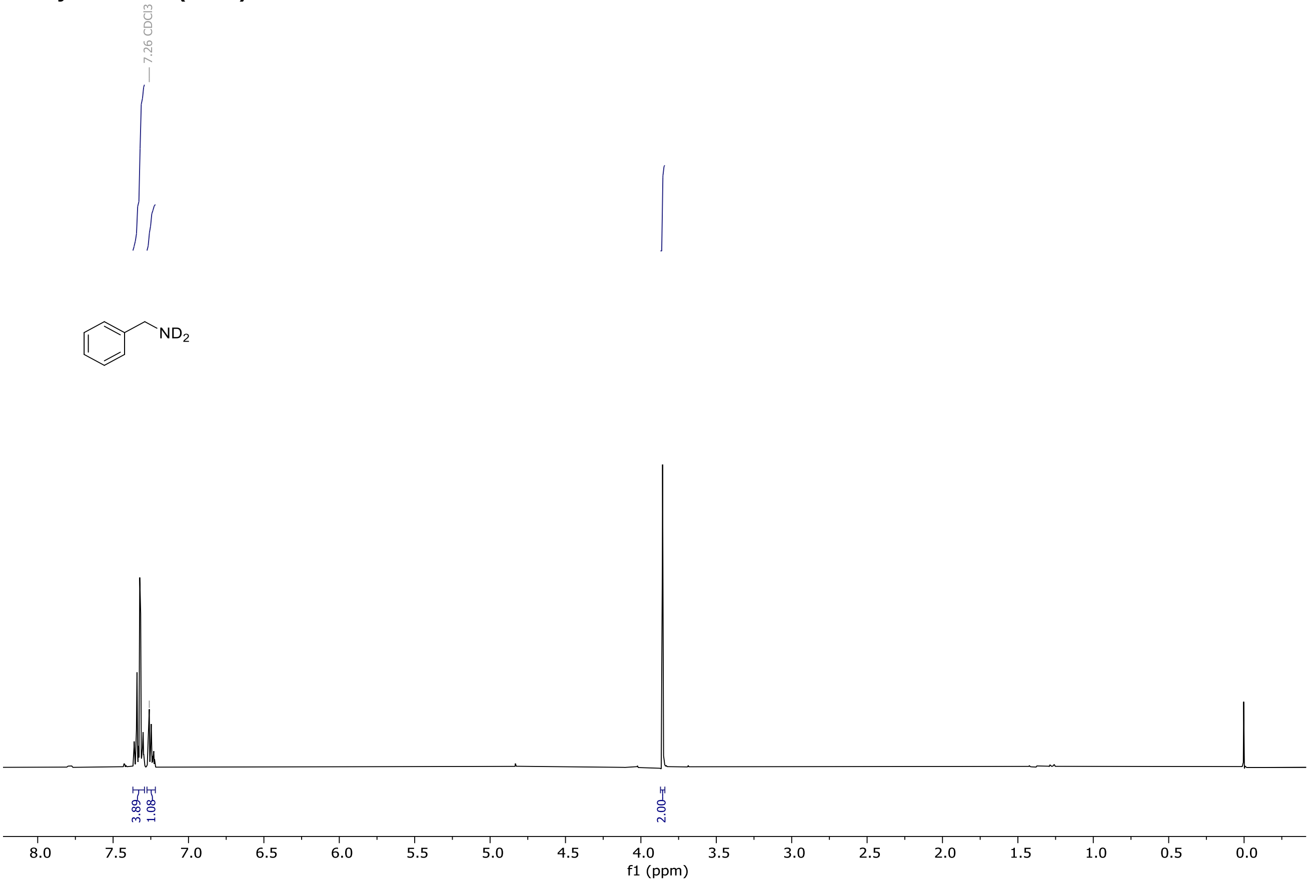

(*E*)-(4-nitrobut-1-en-1-yl)benzene

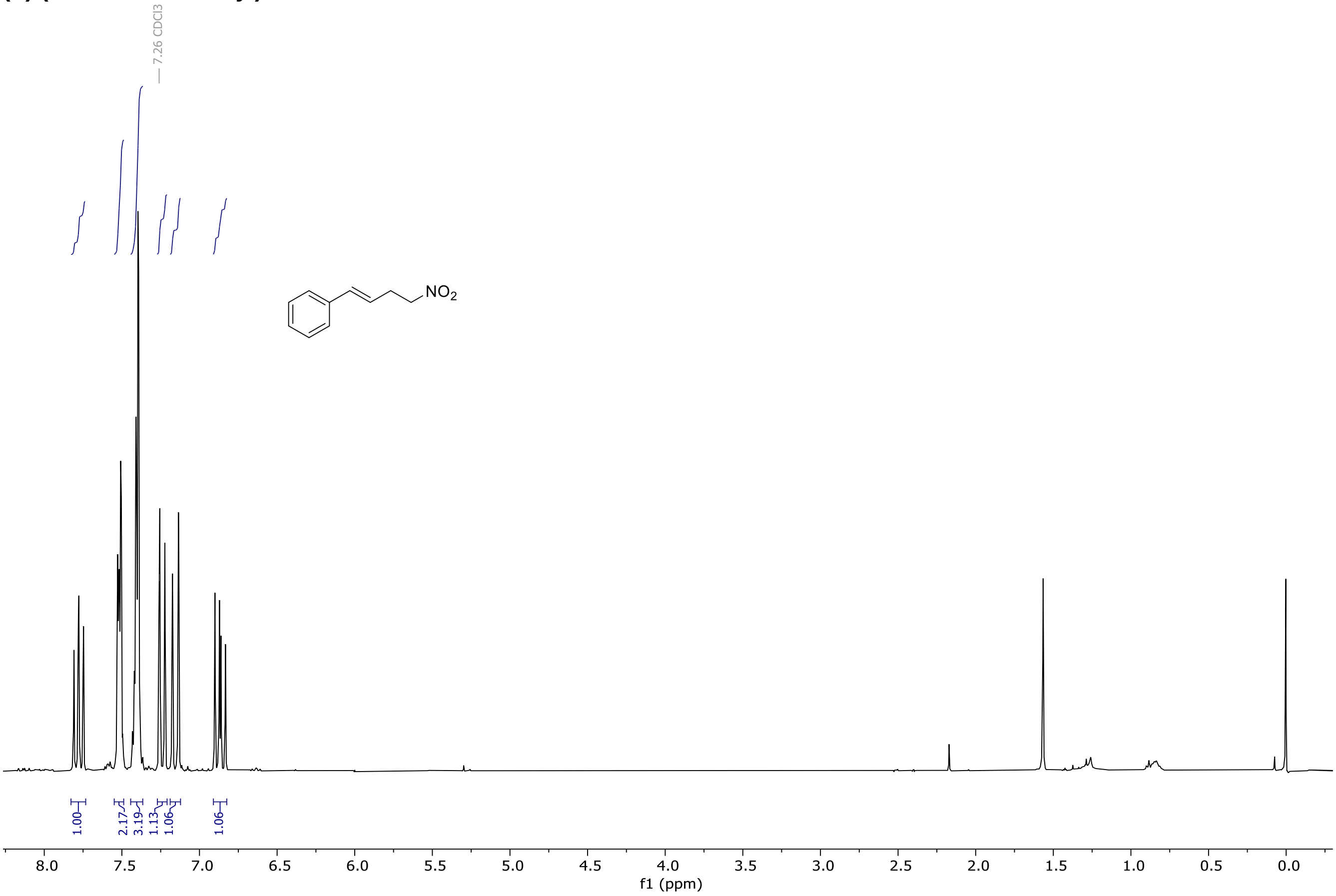

(*E*)-4-phenylbut-3-en-1-amine (2w)

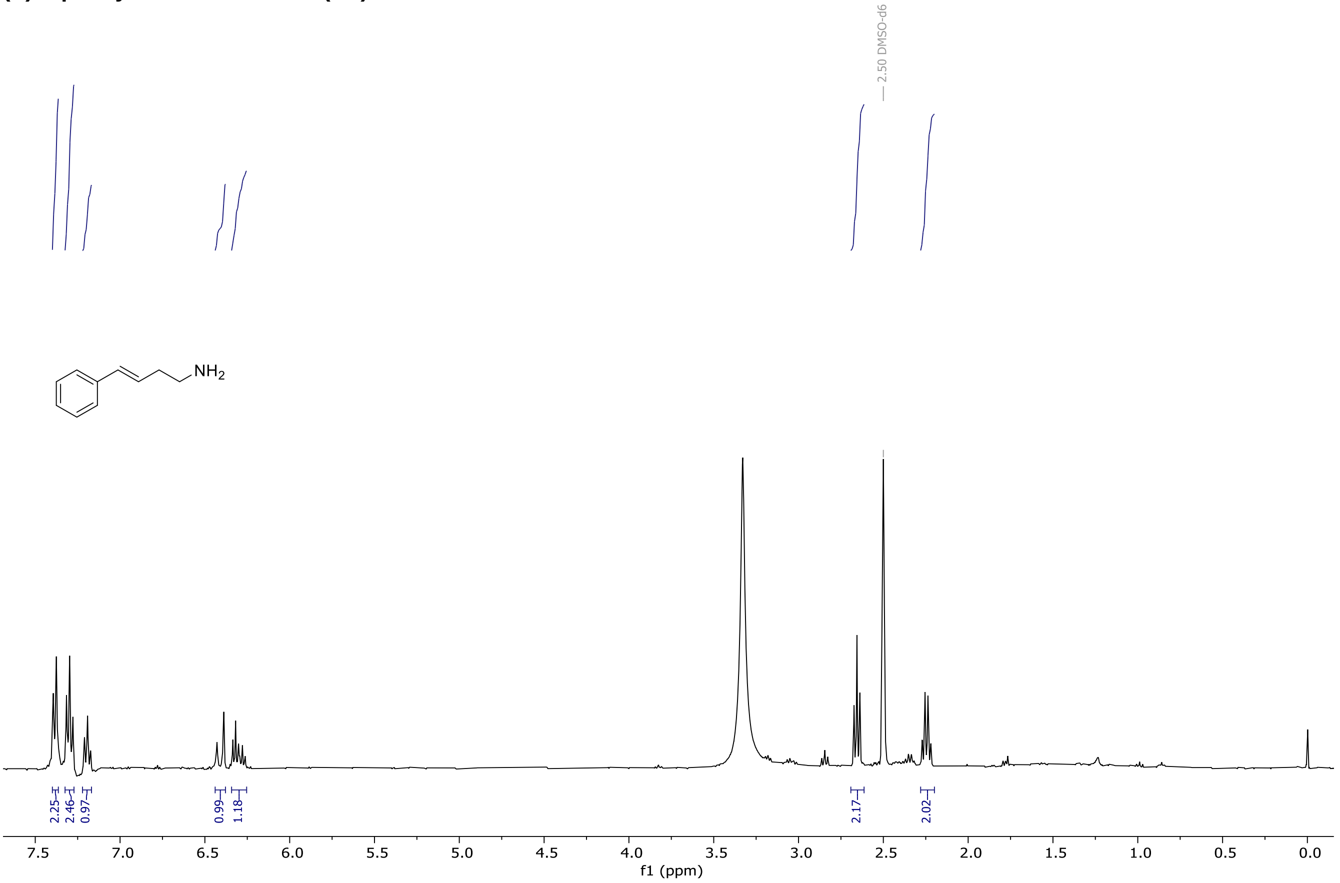

***N*$^{\alpha}$-Fmoc-L-lysine allyl ester hydrochoride (2x)**

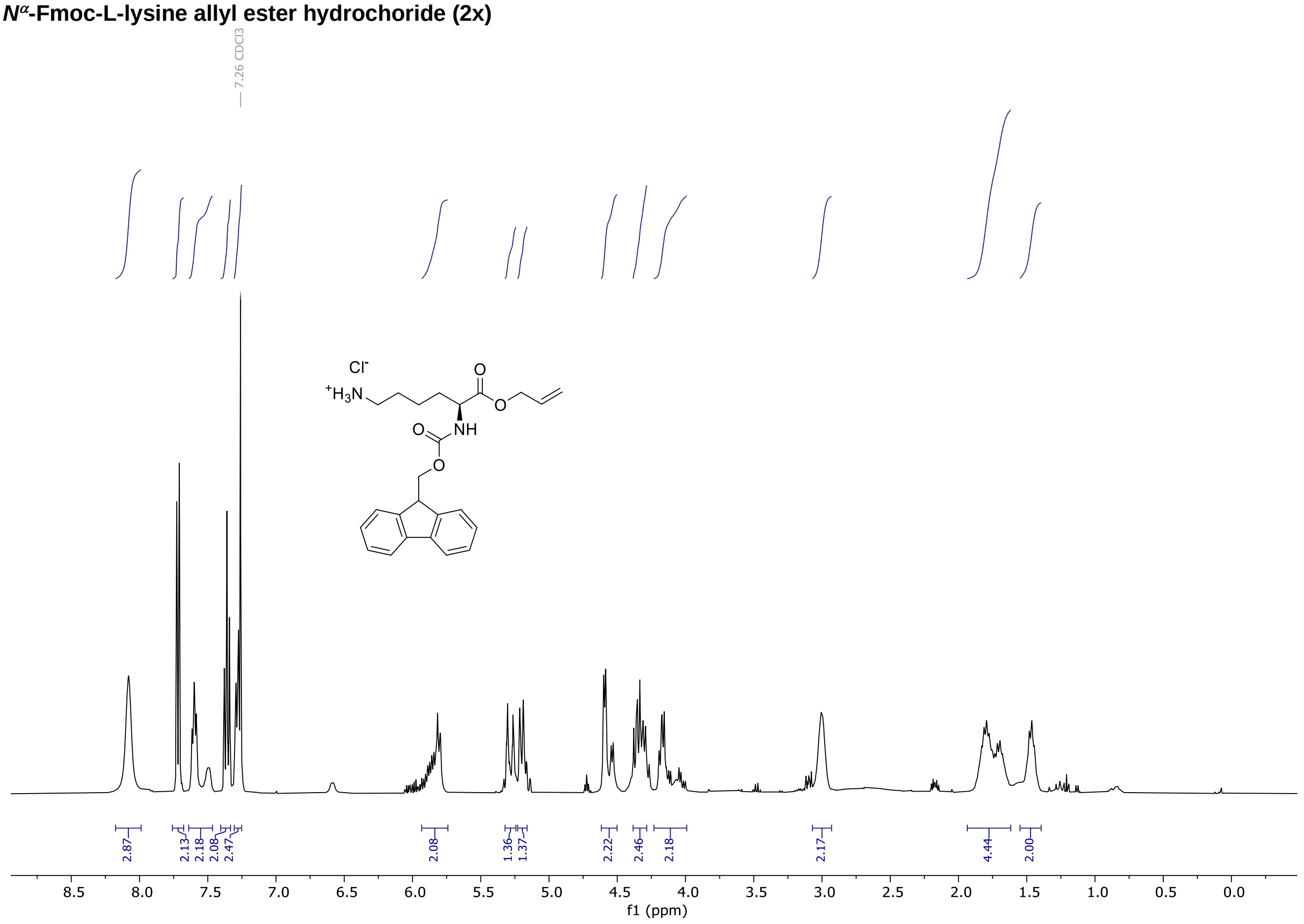

Pyrrolidine-1-*d* (Nu-1-*d*)

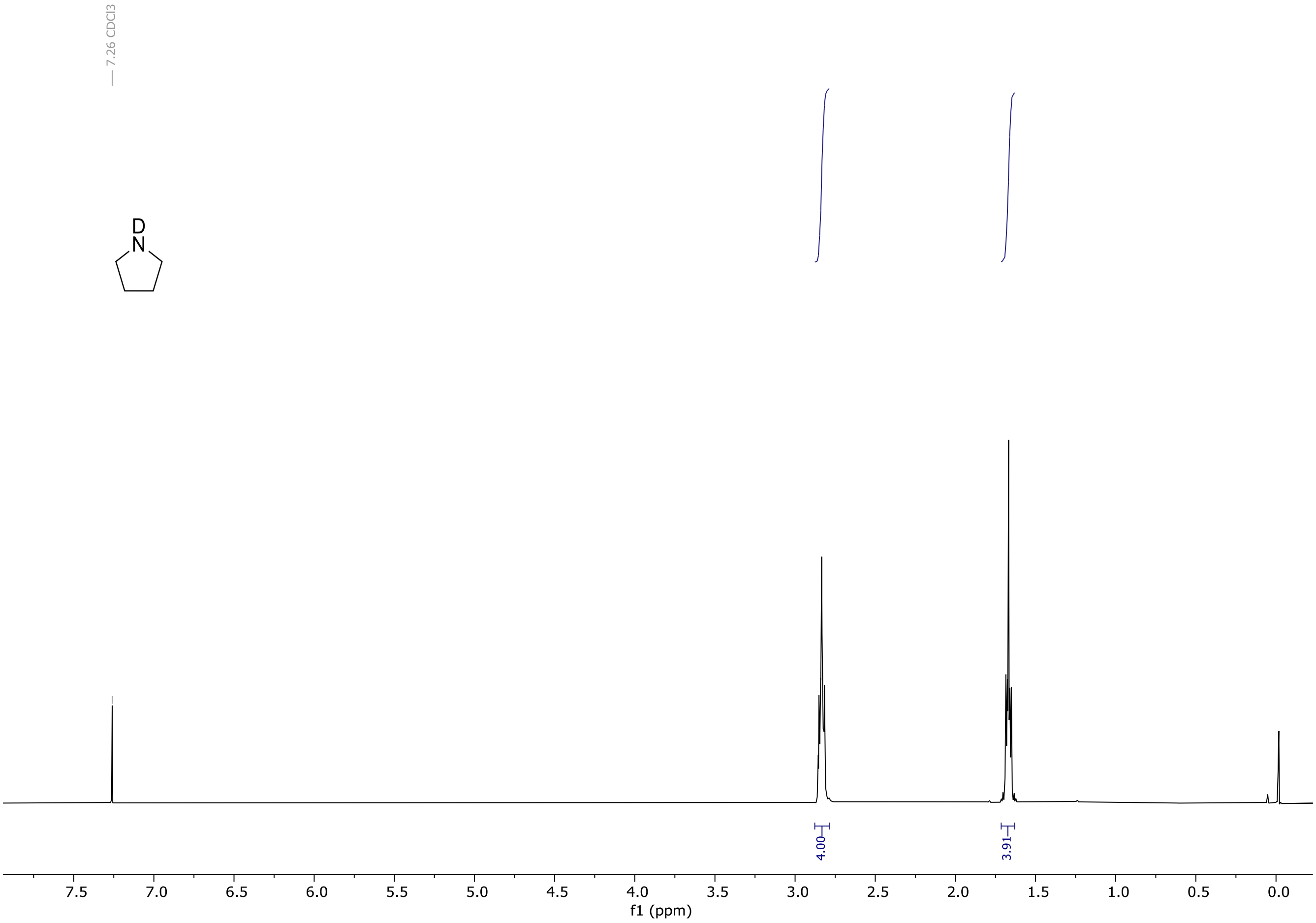

8.2. Compounds 4

Methyl 1-benzyl-5-(4-chlorophenyl)-4,5-dihydro-1*H*-imidazole-4-carboxylate (4a)

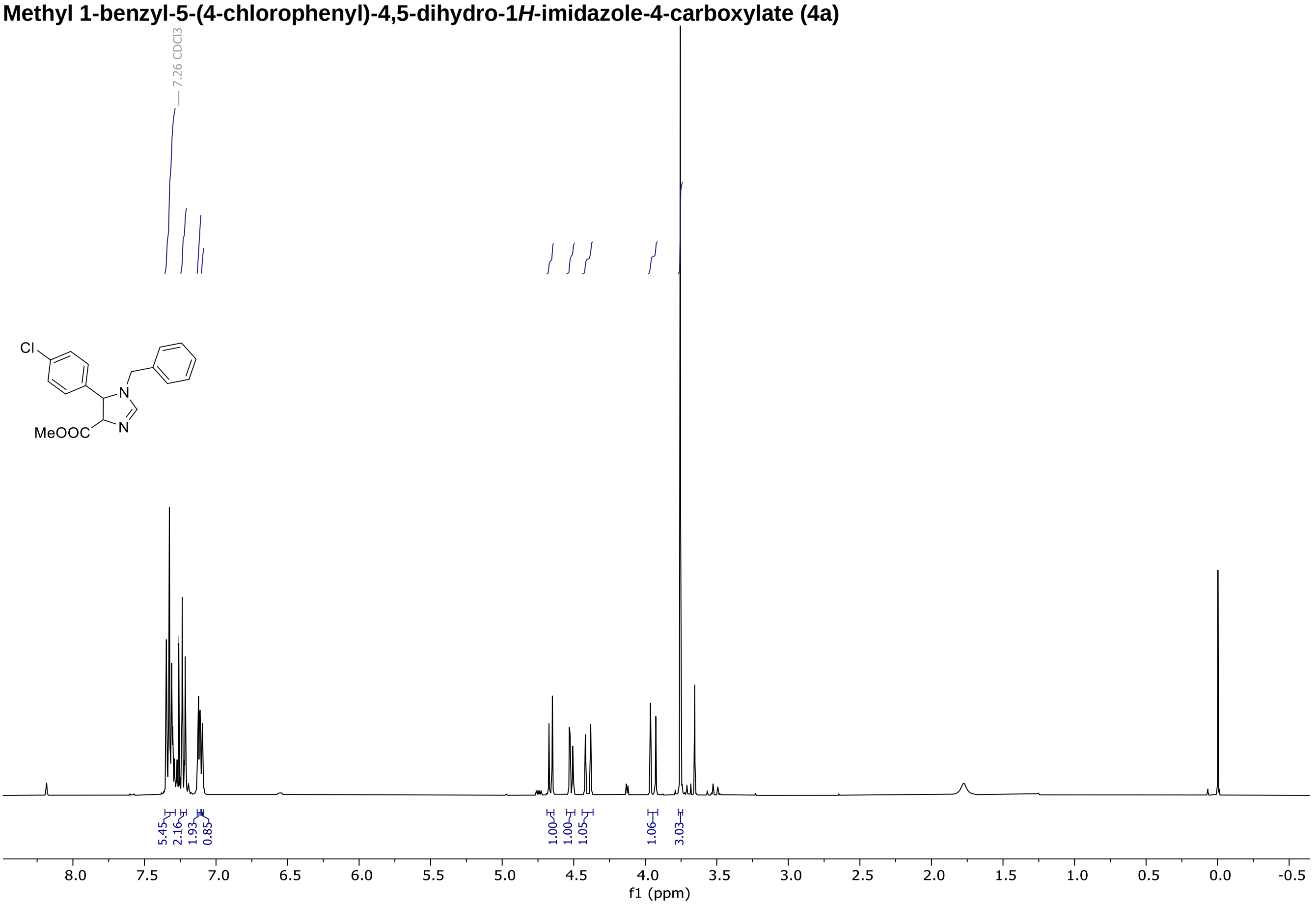

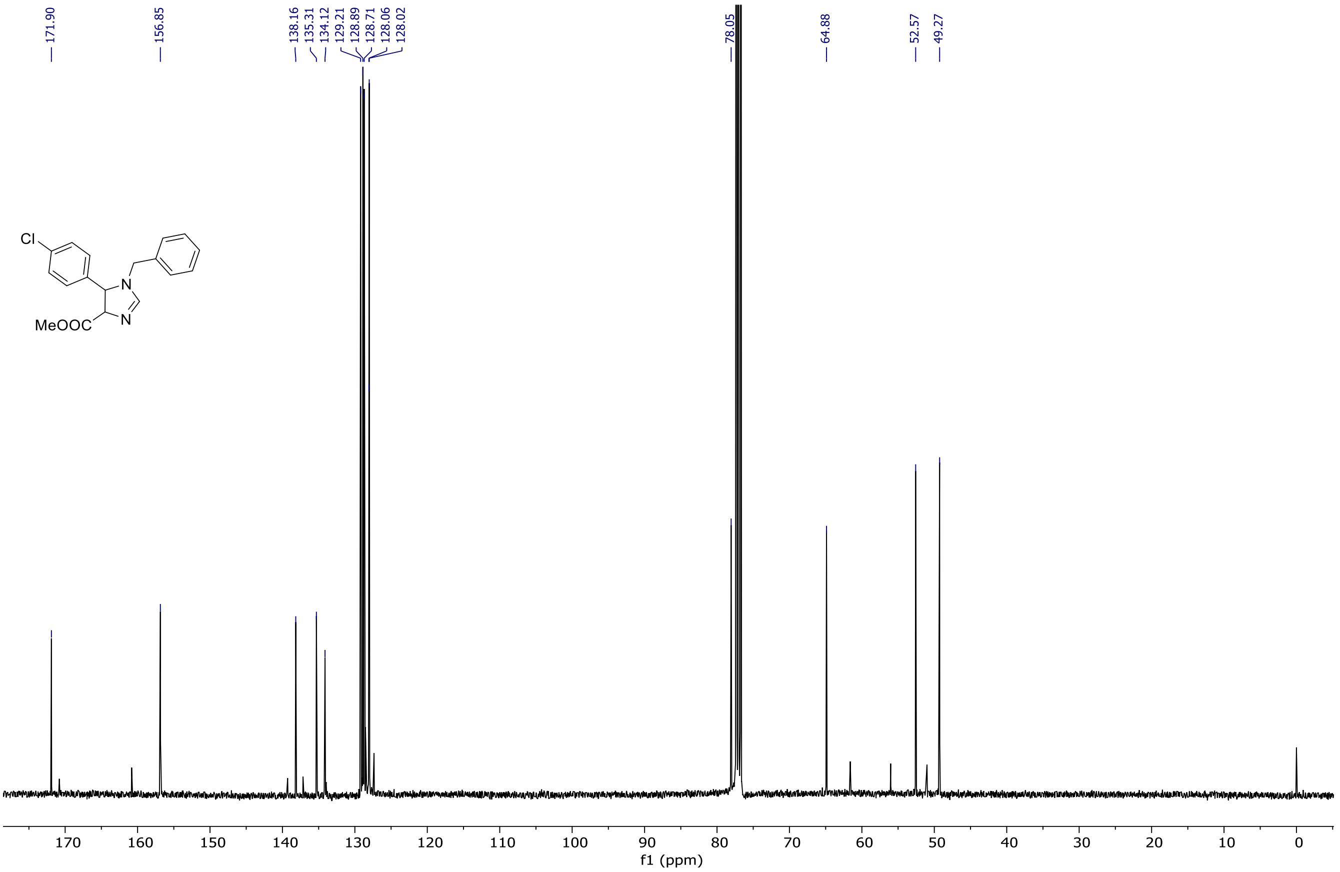

Methyl 1-benzyl-5,5-dimethyl-4,5-dihydro-1H-imidazole-4-carboxylate (4aa)

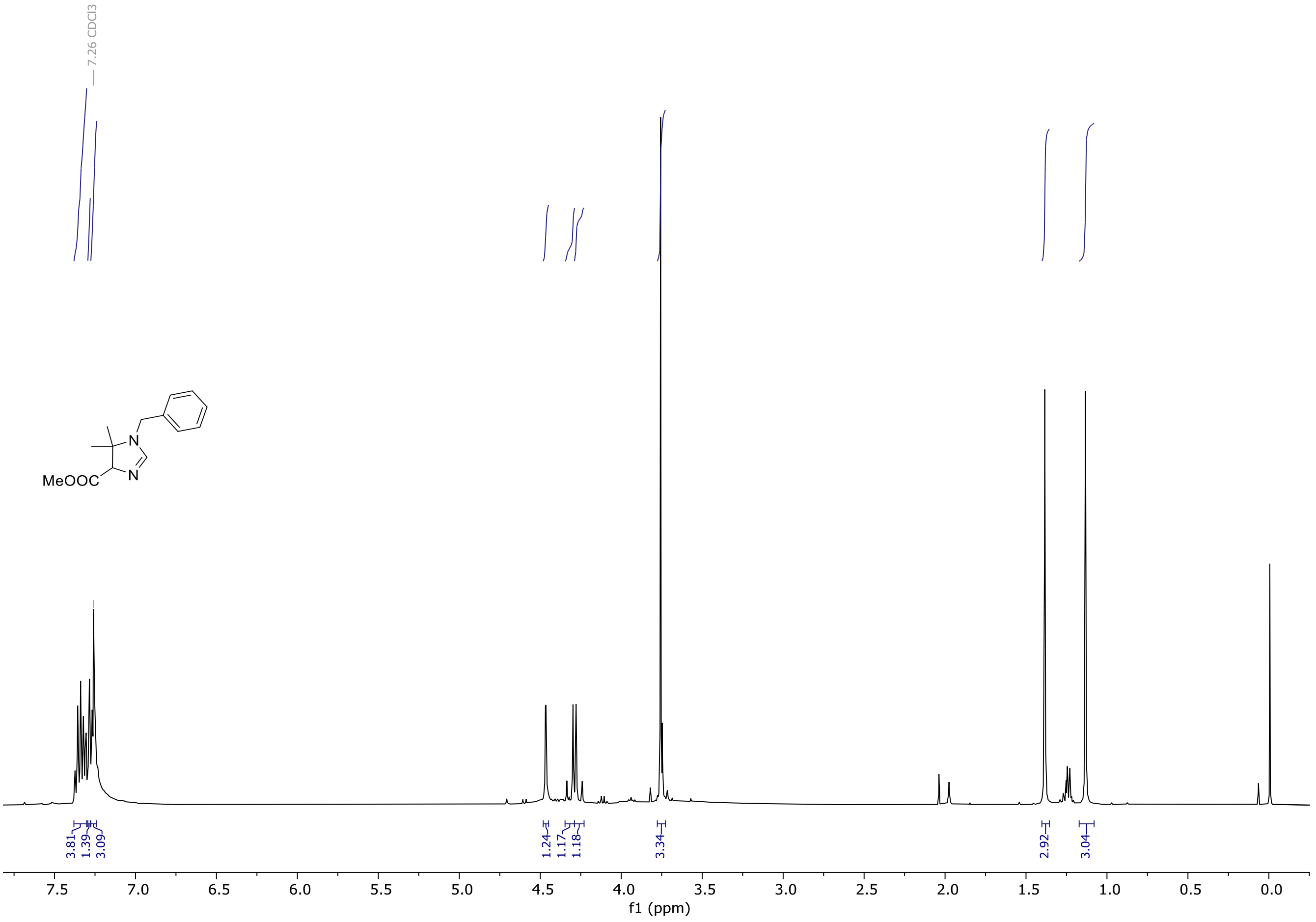

Methyl 1-benzyl-1,3-diazaspiro[4.4]non-2-ene-4-carboxylate (4ab)

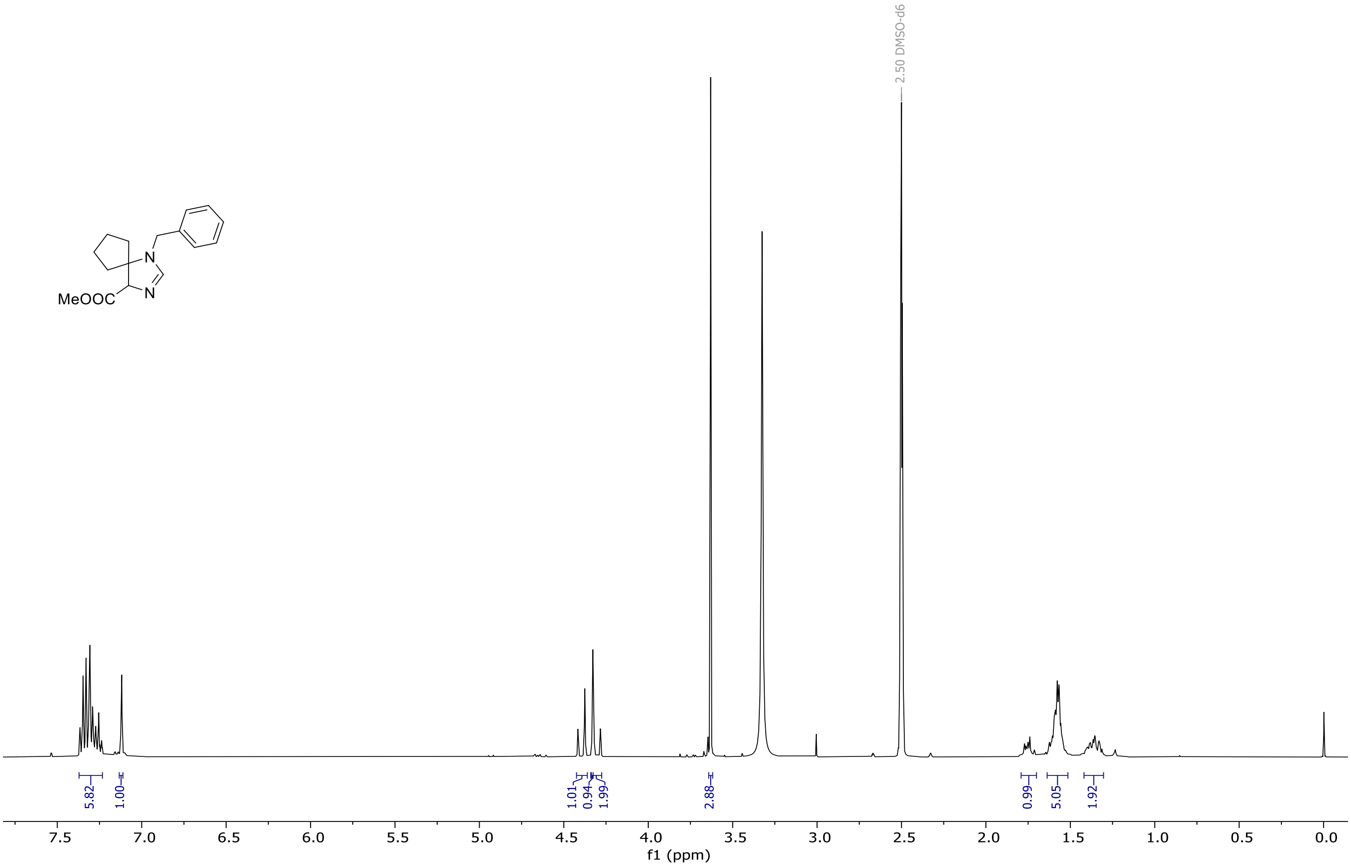

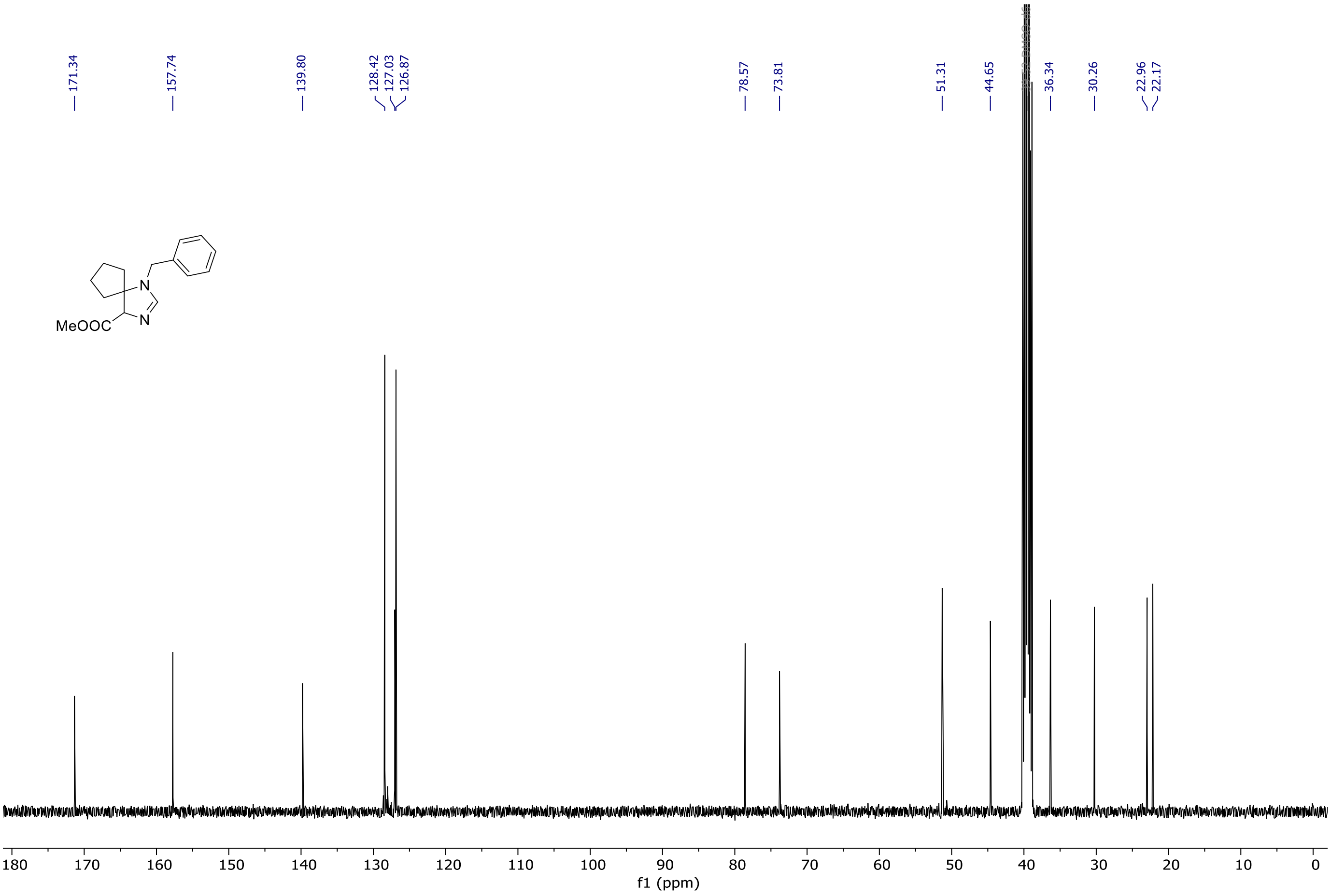

Methyl (1r,3r,5r,7r)-3'-benzyl-3',5'-dihydrospiro[adamantane-2,4'-imidazole]-5'-carboxylate (4ac)

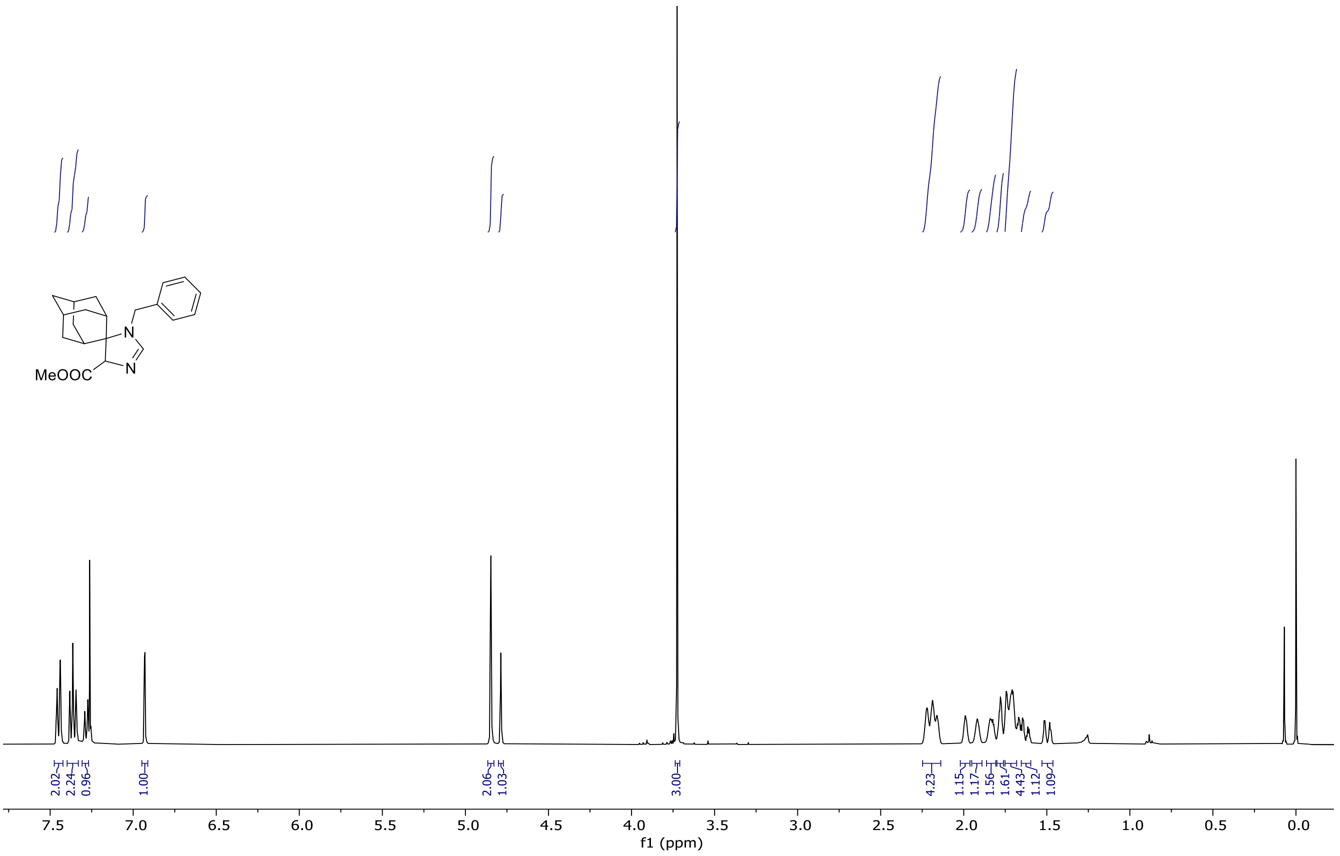

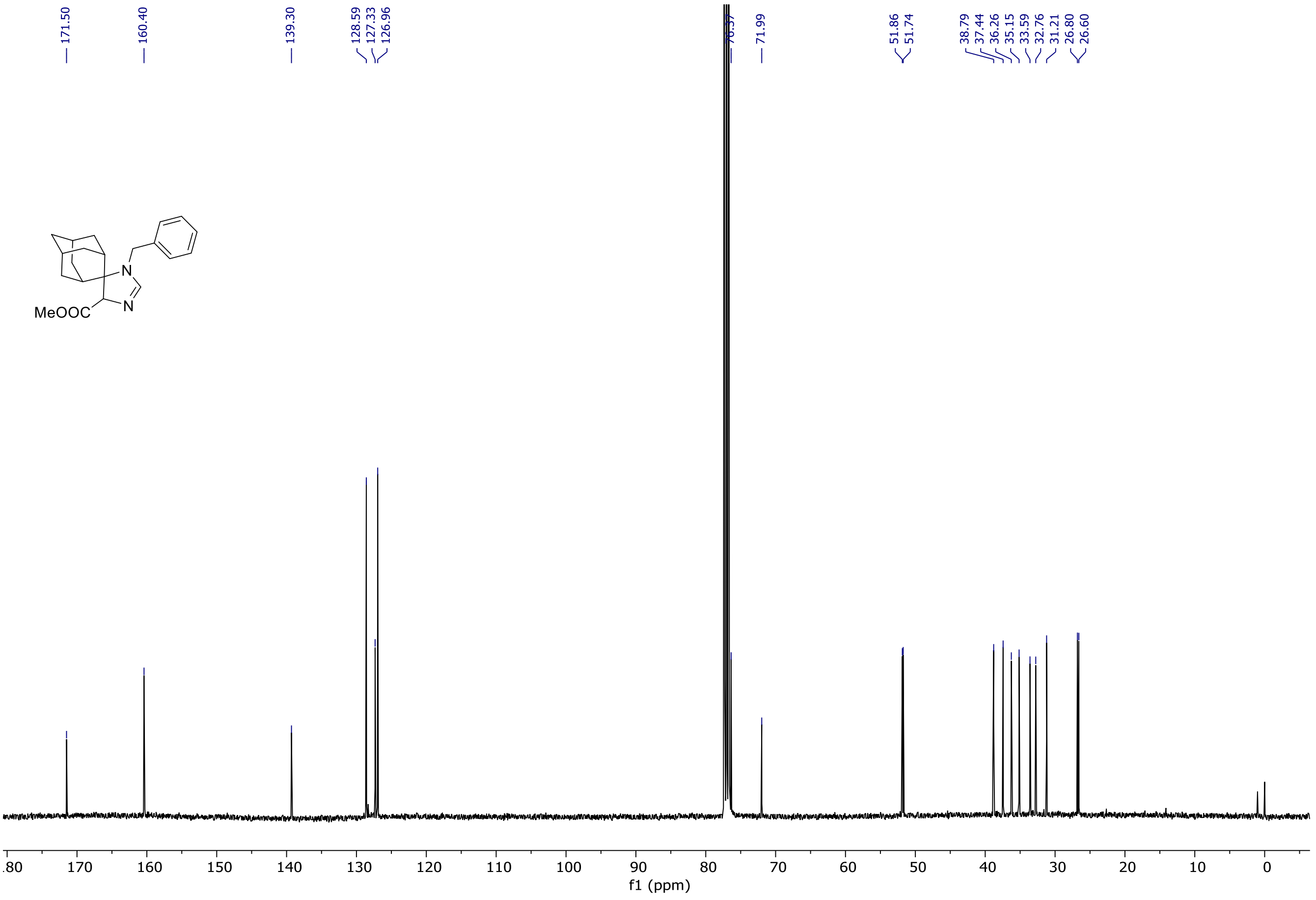

8.3. Compounds 5

(*Z*)-3-benzyl-5-(4-chlorobenzylidene)-3,5-dihydro-4*H*-imidazol-4-one (5a)

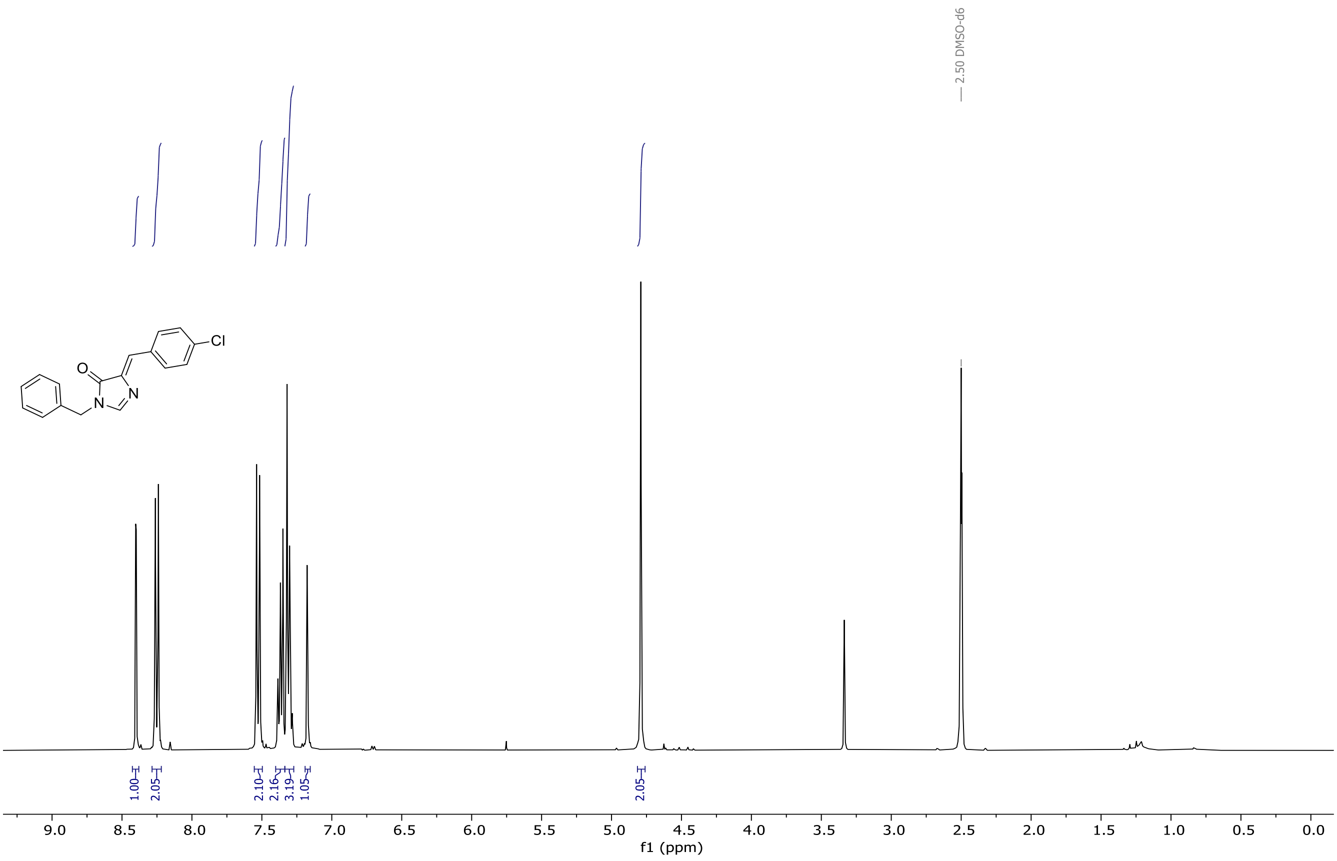

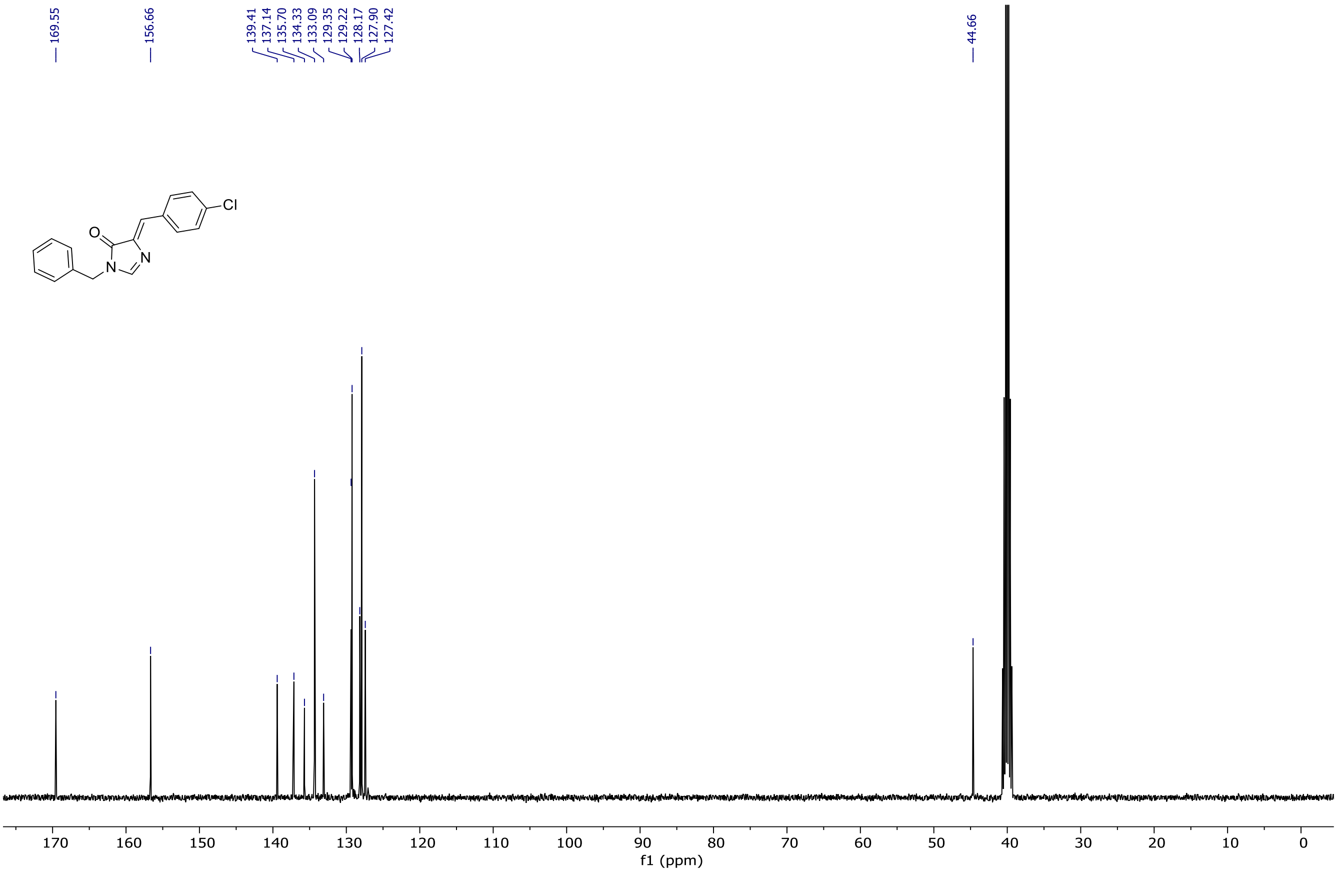

(*Z*)-3-benzyl-5-(4-chlorobenzylidene)-3,5-dihydro-4*H*-imidazol-4-one-2-*d* (5a-*d*)

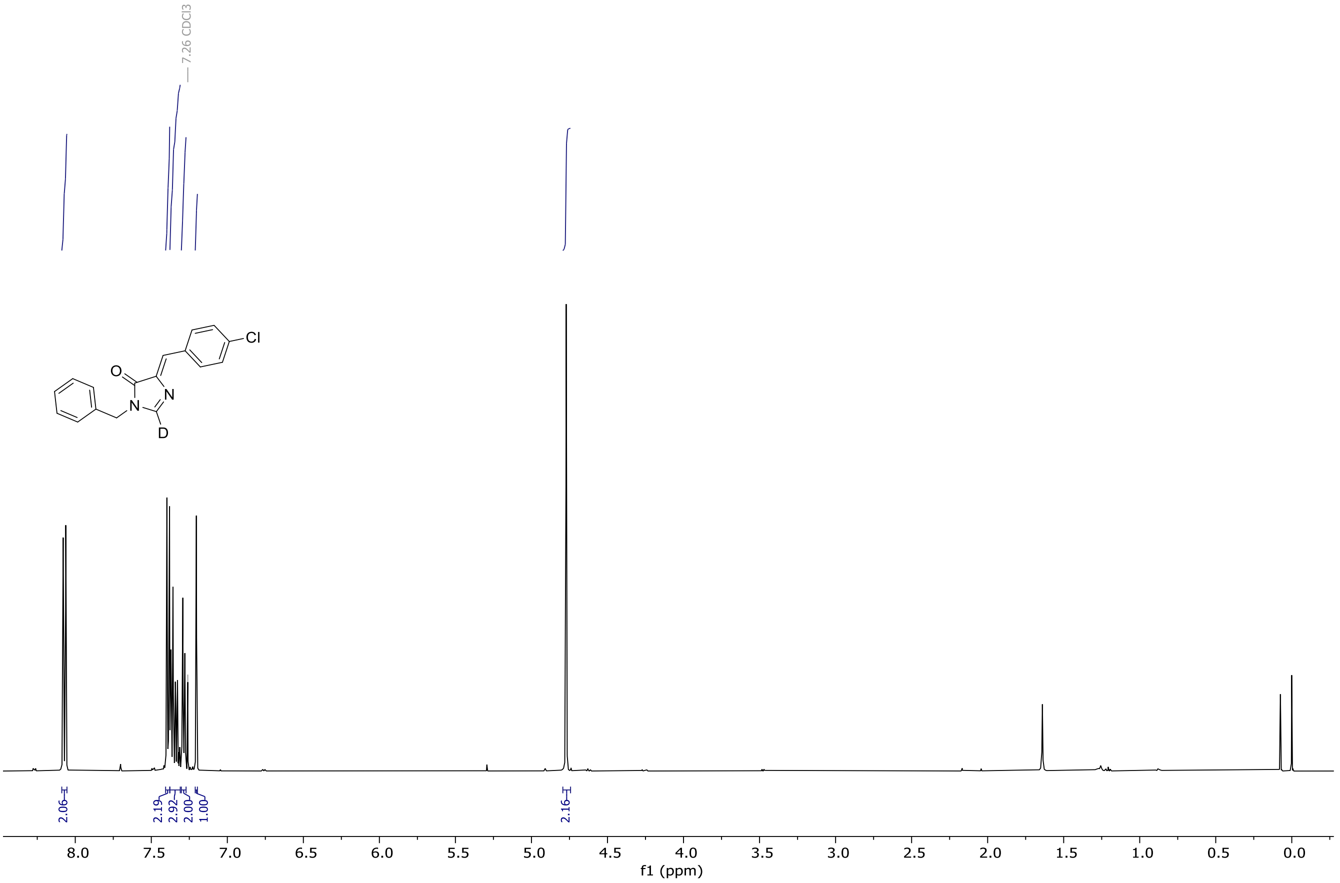

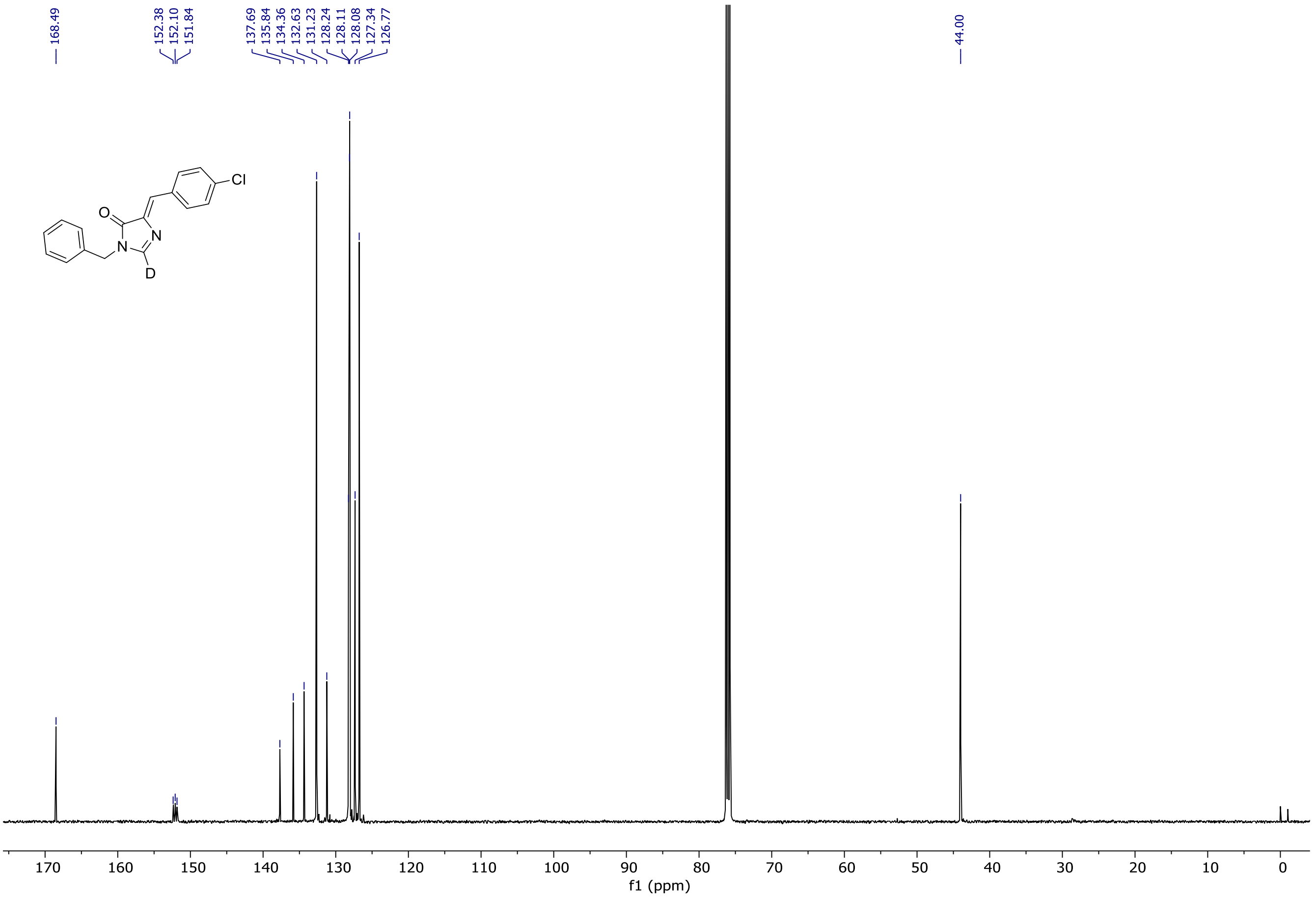

(*Z*)-3-Benzyl-5-(4-chlorobenzylidene)-3,5-dihydro-4*H*-imidazol-4-one (5b)

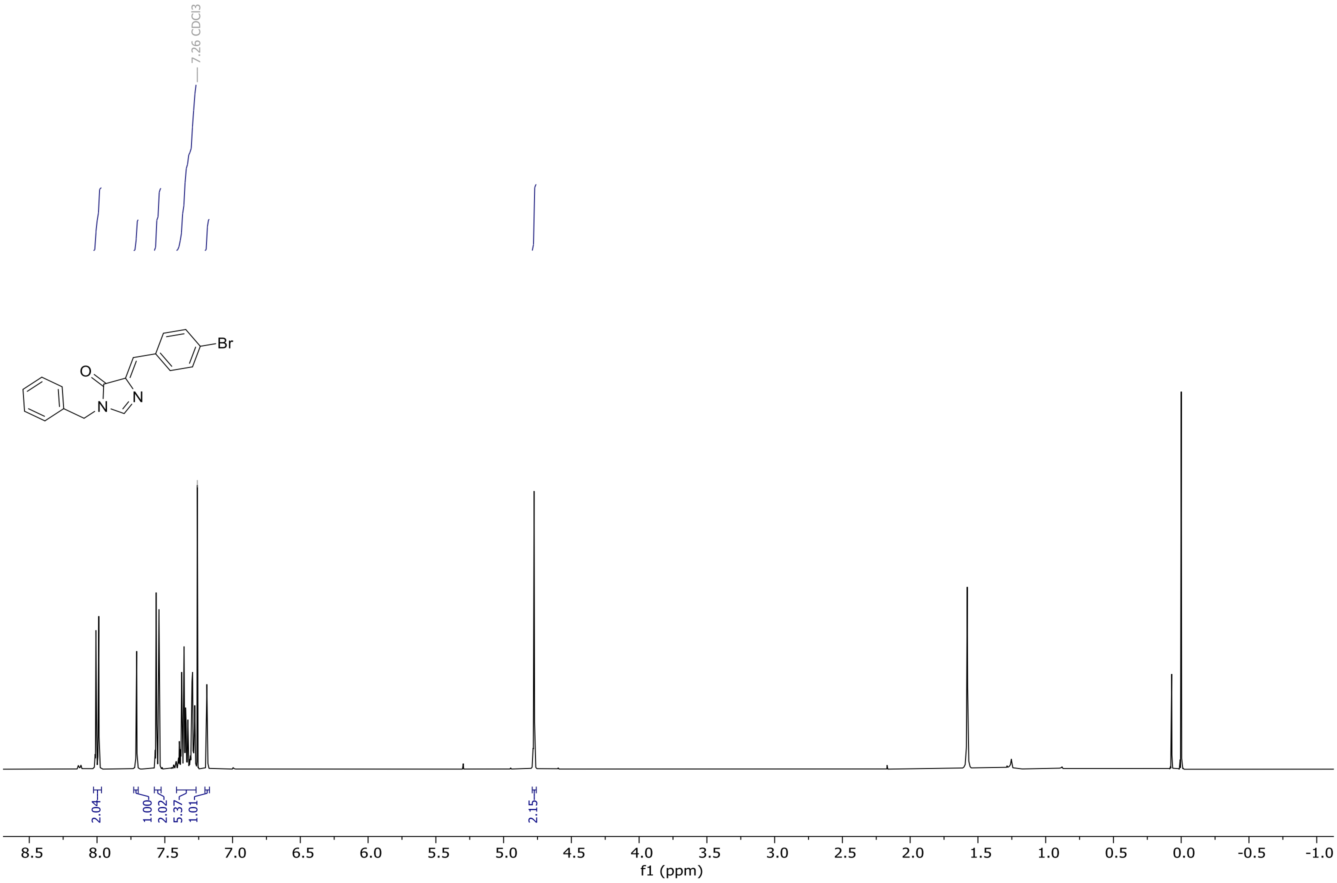

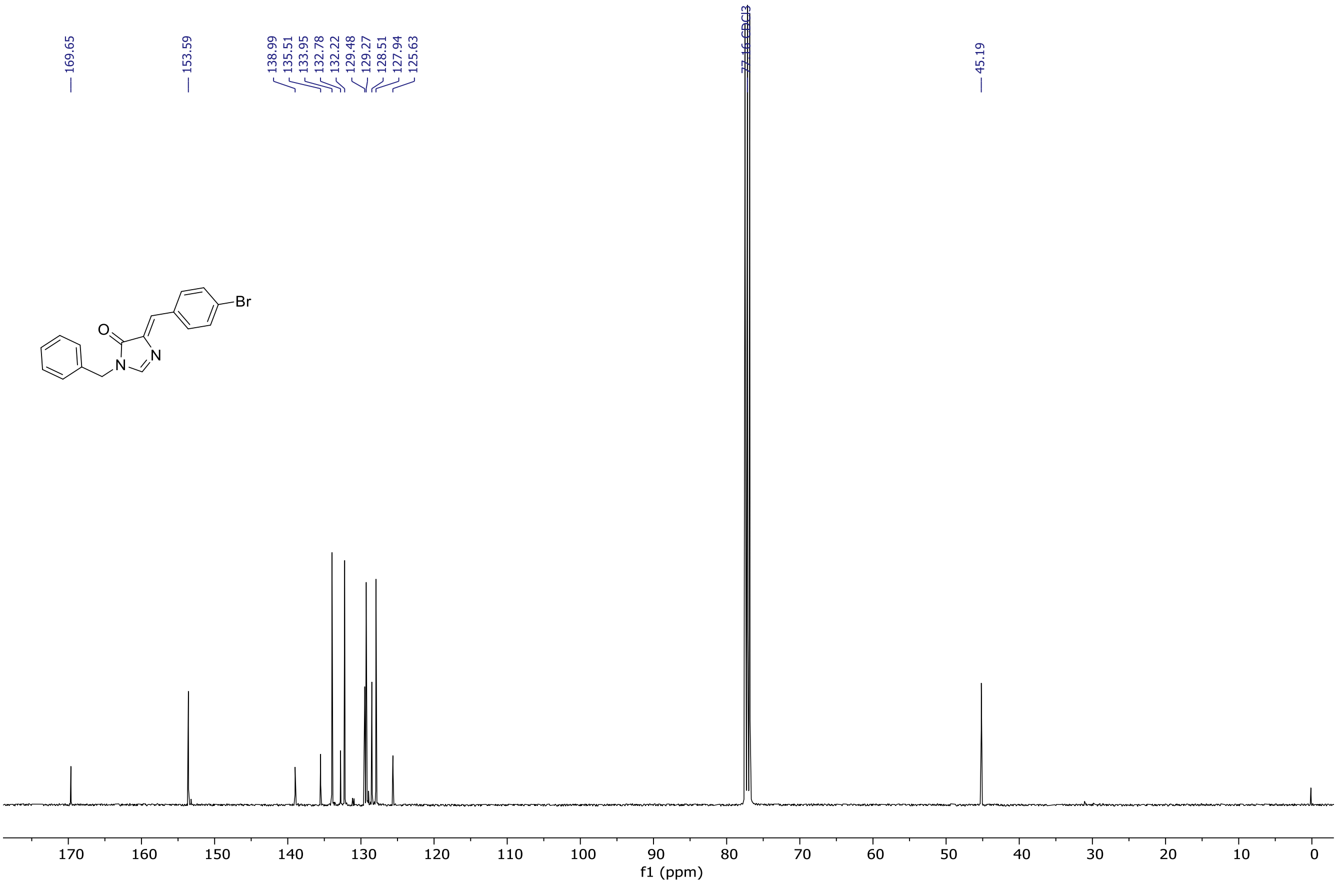

(Z)-3-Benzyl-5-(4-methoxybenzylidene)-3,5-dihydro-4H-imidazol-4-one (5c)

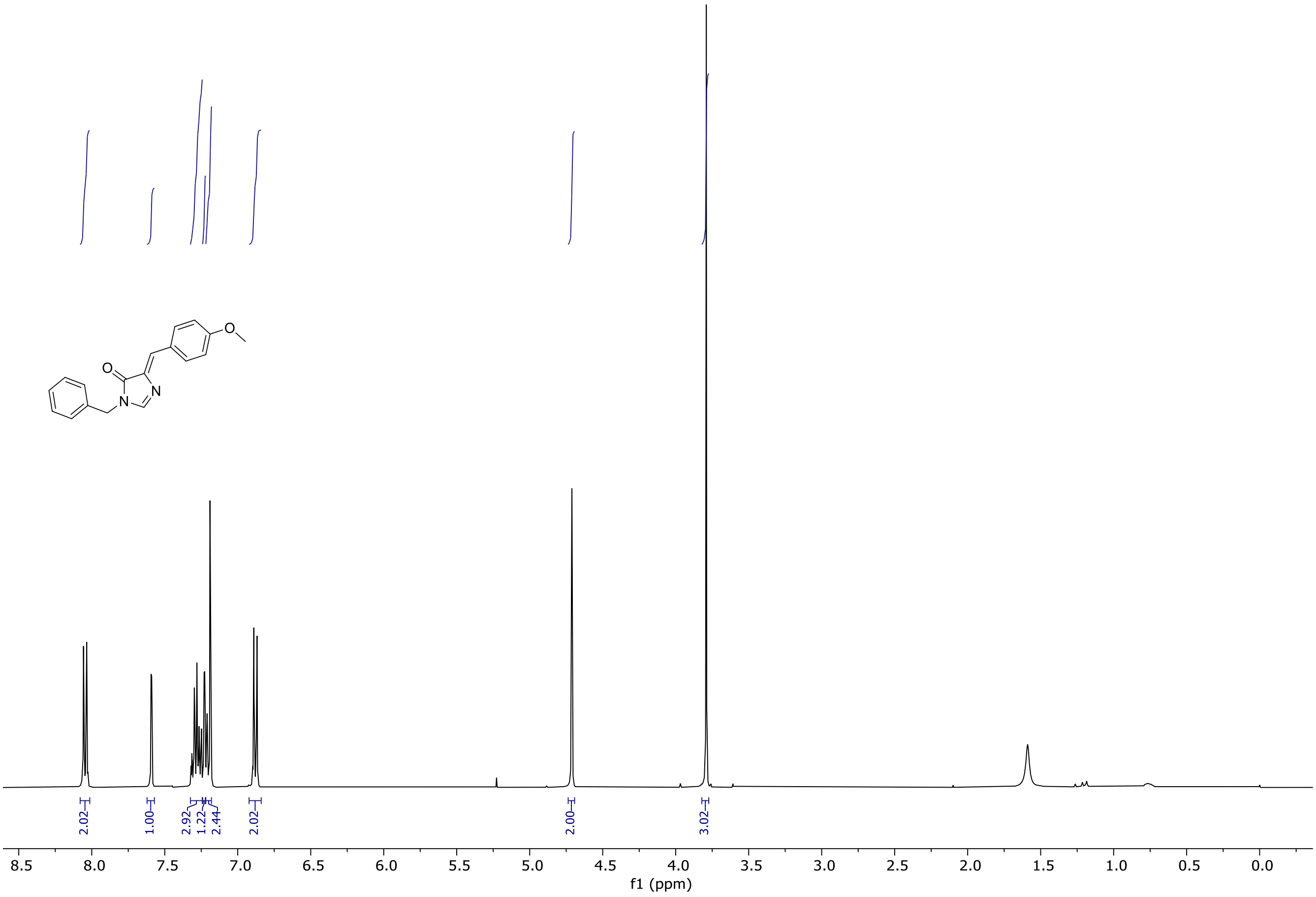

(*Z*)-3-(3-iodobenzyl)-5-(4-methoxybenzylidene)-3,5-dihydro-4*H*-imidazol-4-one (5d)

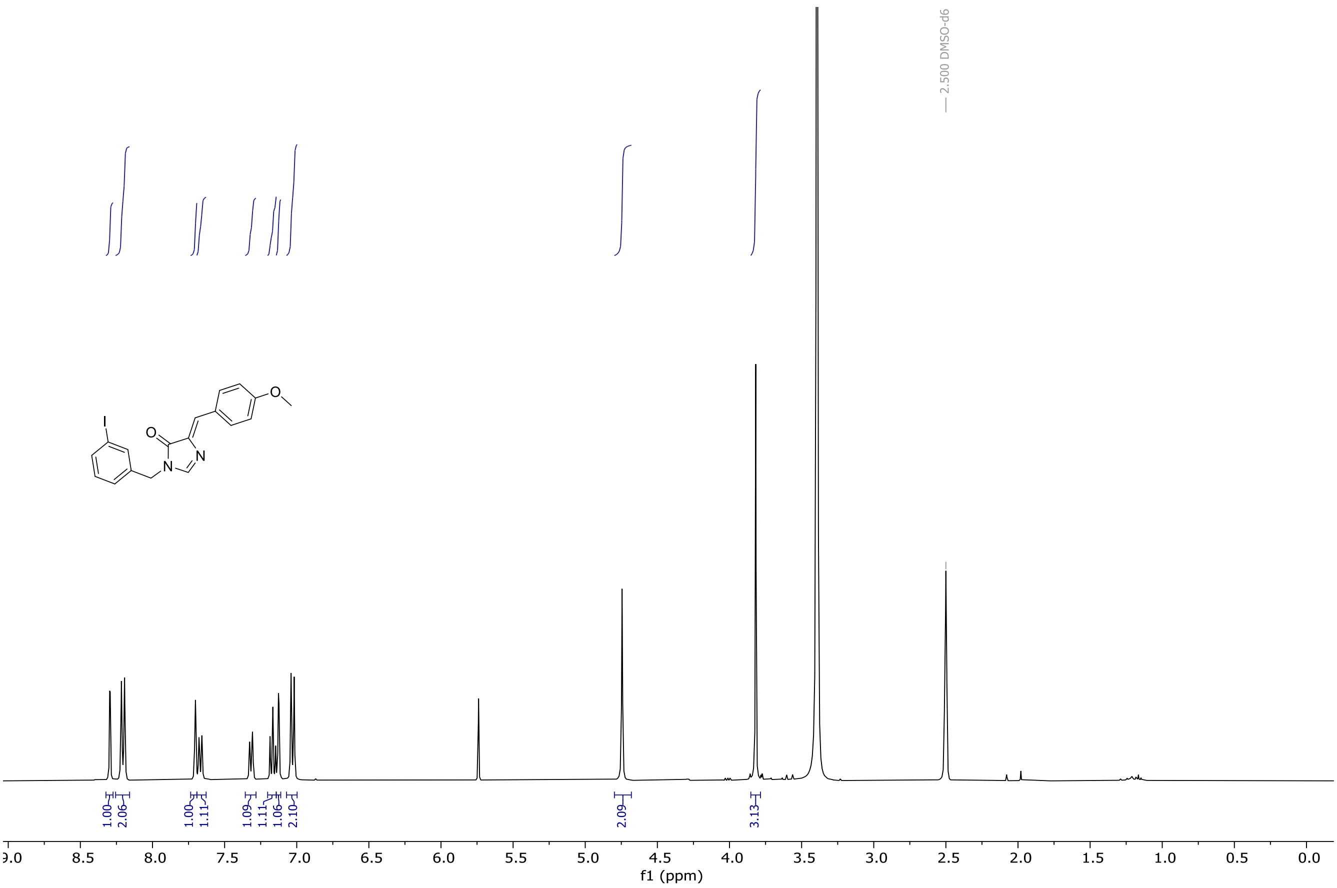

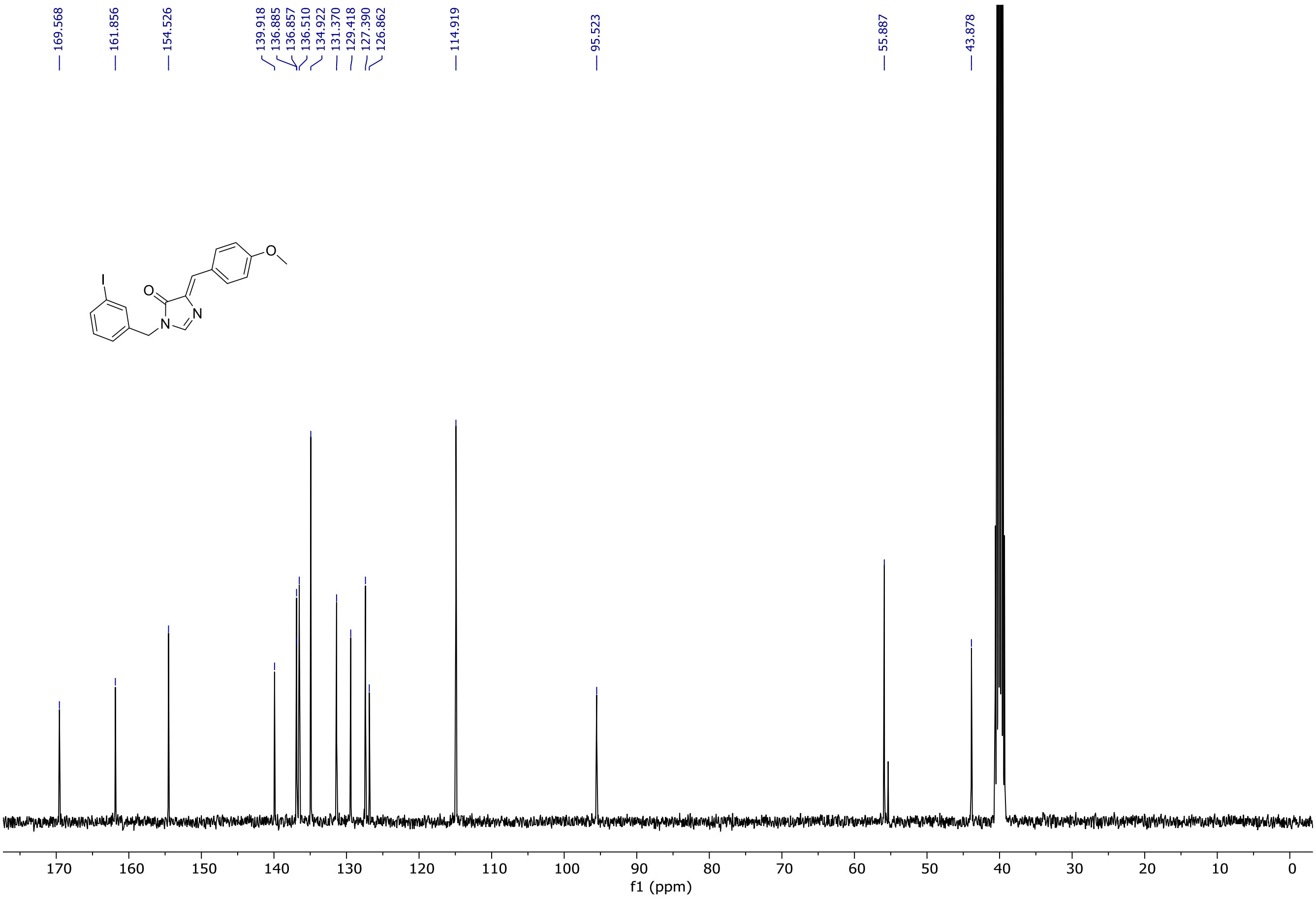

(*Z*)-5-(4-methoxybenzylidene)-3-(pyridin-2-ylmethyl)-3,5-dihydro-4*H*-imidazol-4-one (5e)

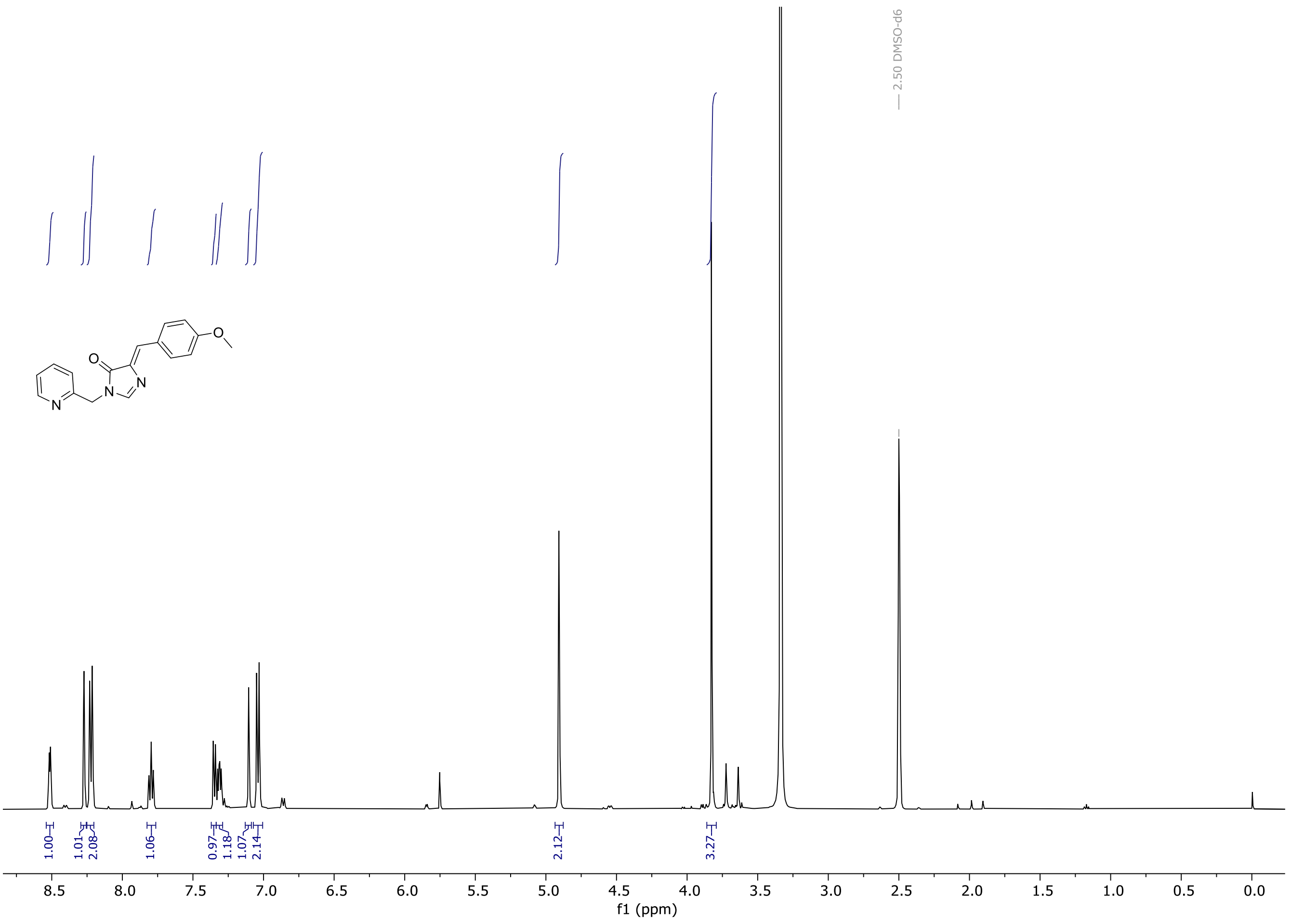

(*R,Z*)-5-(4-Methoxybenzylidene)-3-(1-phenylethyl)-3,5-dihydro-4*H*-imidazol-4-one (5f)

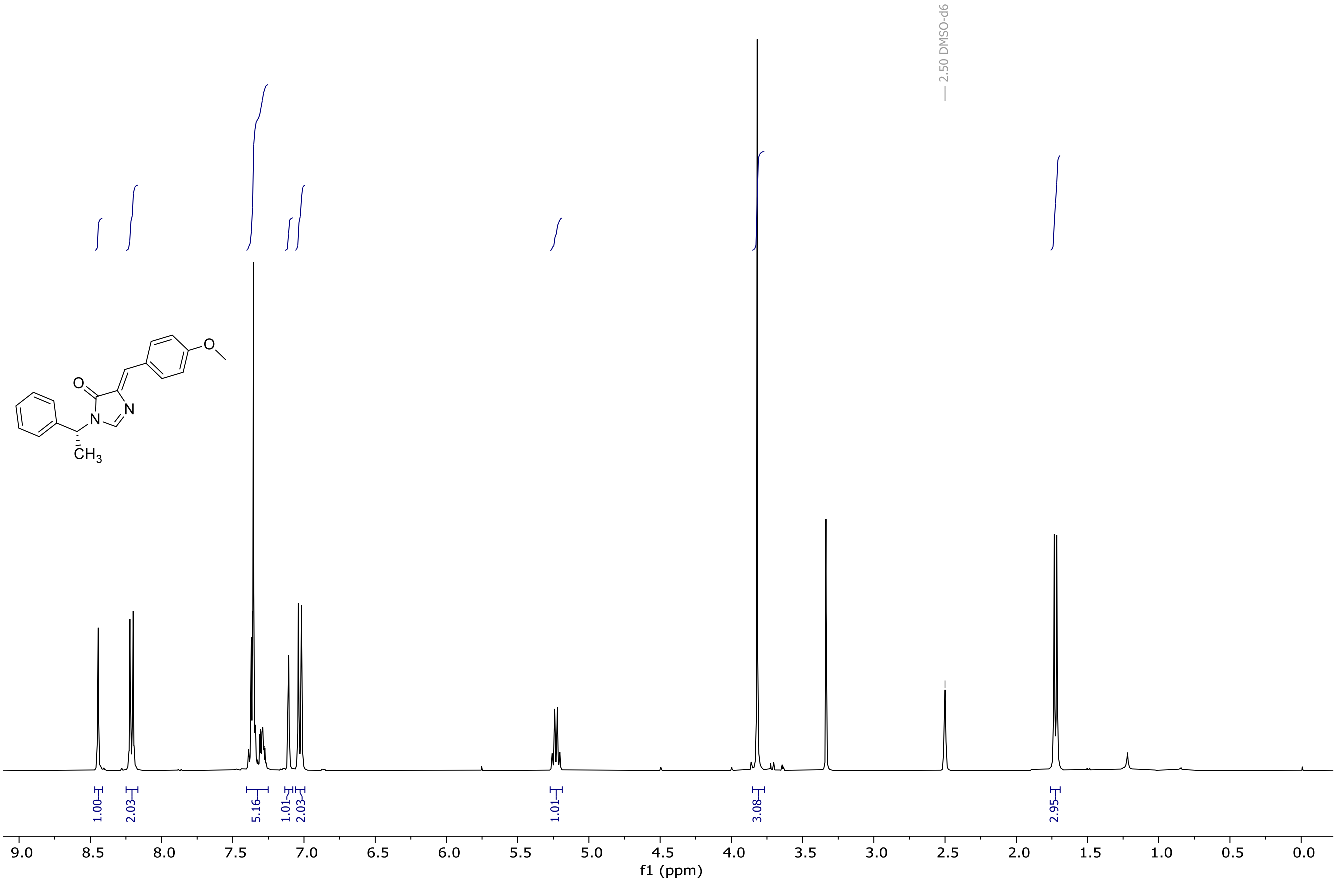

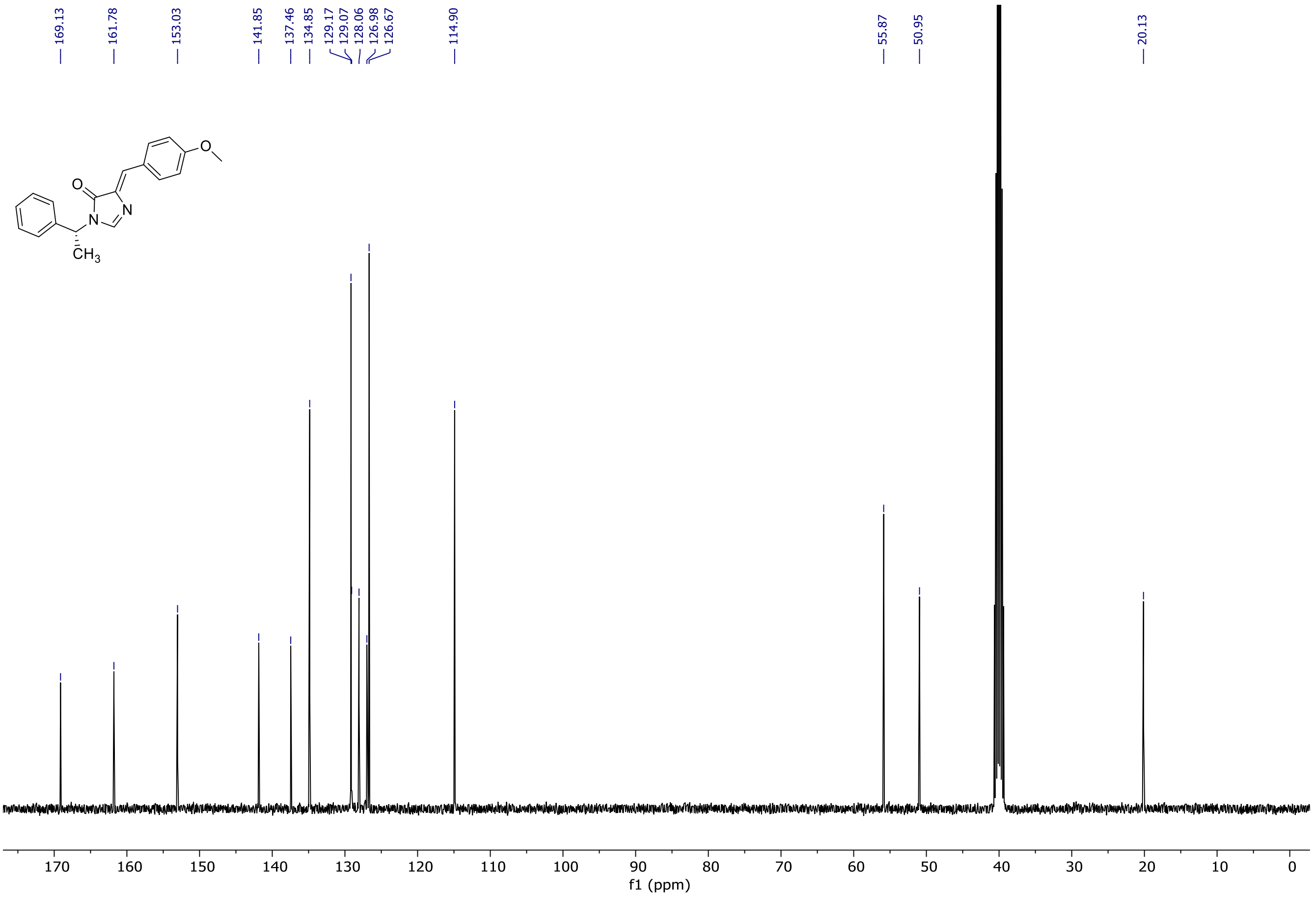

(*Z*)-5-(2-(allyloxy)benzylidene)-3-(4-methylbenzyl)-3,5-dihydro-4*H*-imidazol-4-one (5g)

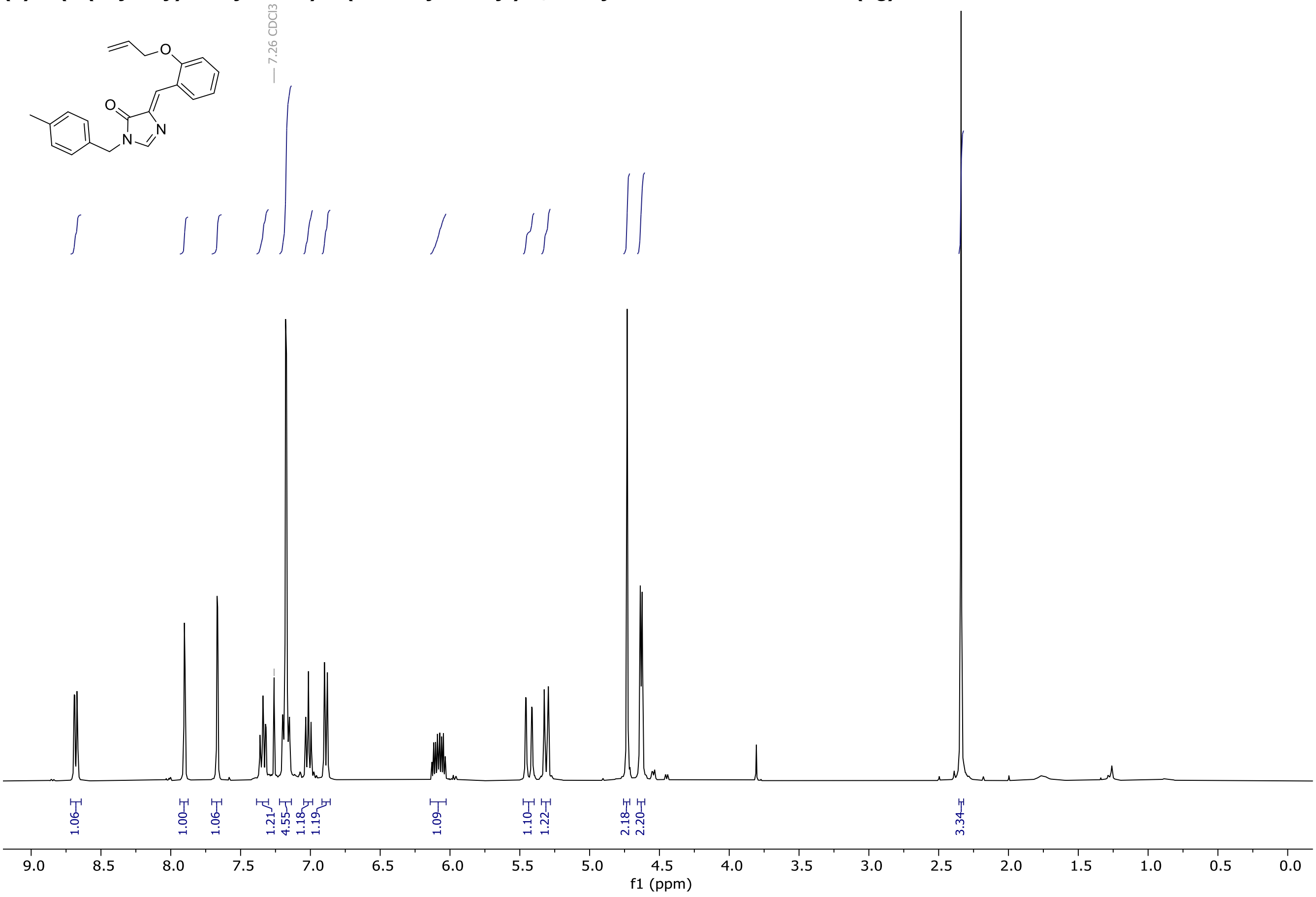

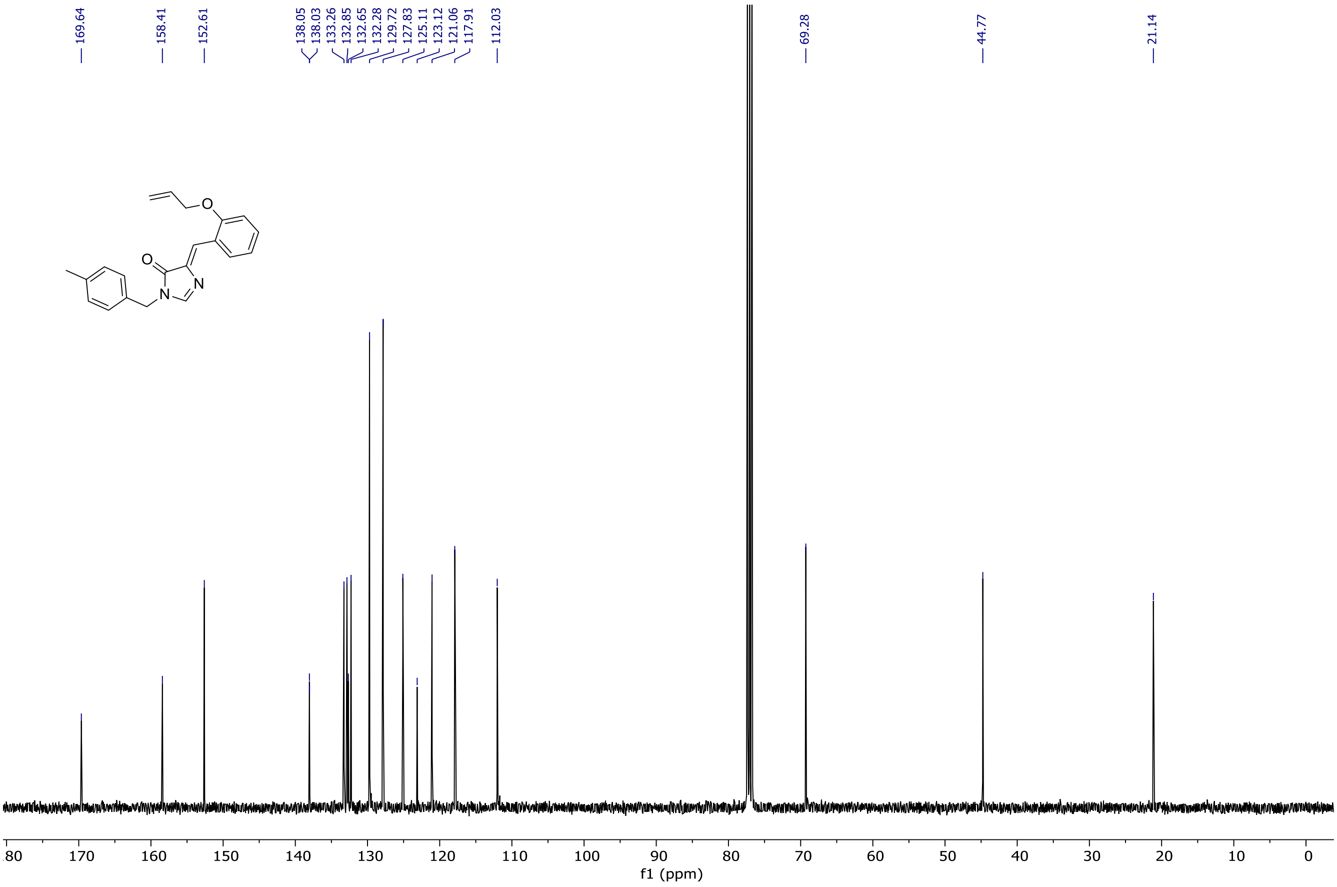

(*Z*)-3-Benzyl-5-(4-(benzyloxy)benzylidene)-3,5-dihydro-4*H*-imidazol-4-one (5h)

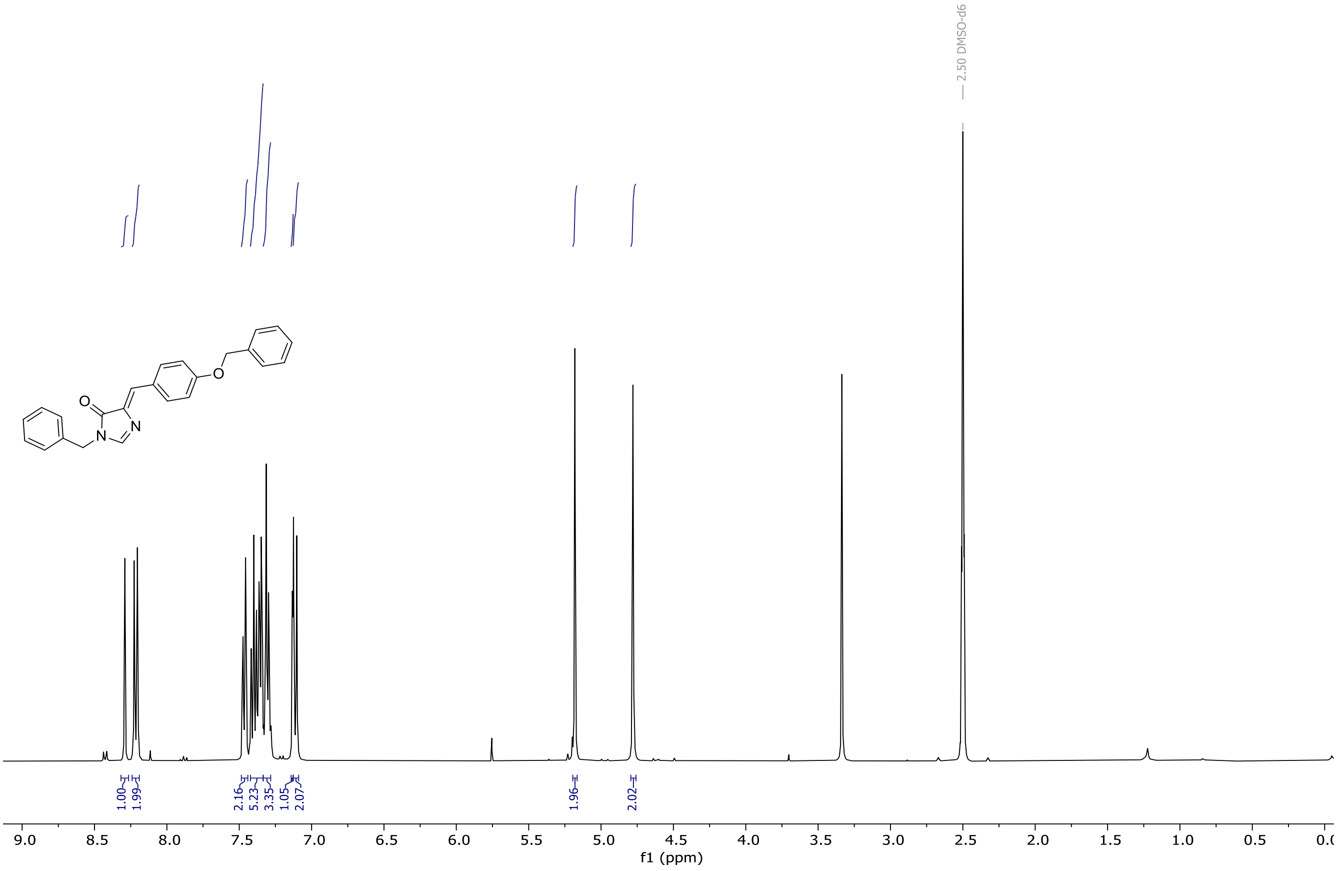

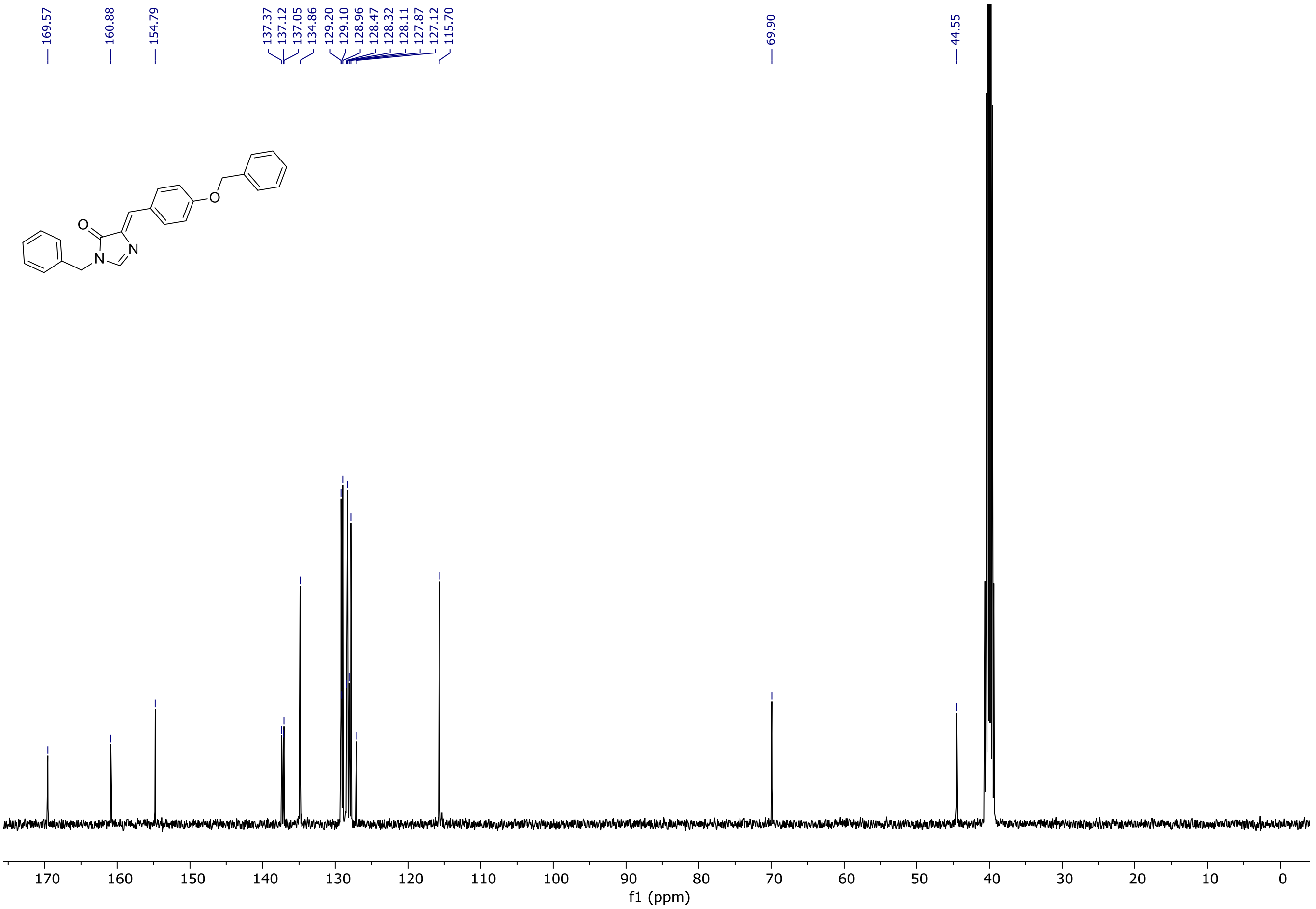

(*Z*)-5-(benzo[d][1,3]dioxol-5-ylmethylene)-3-methyl-3,5-dihydro-4*H*-imidazol-4-one (5i)

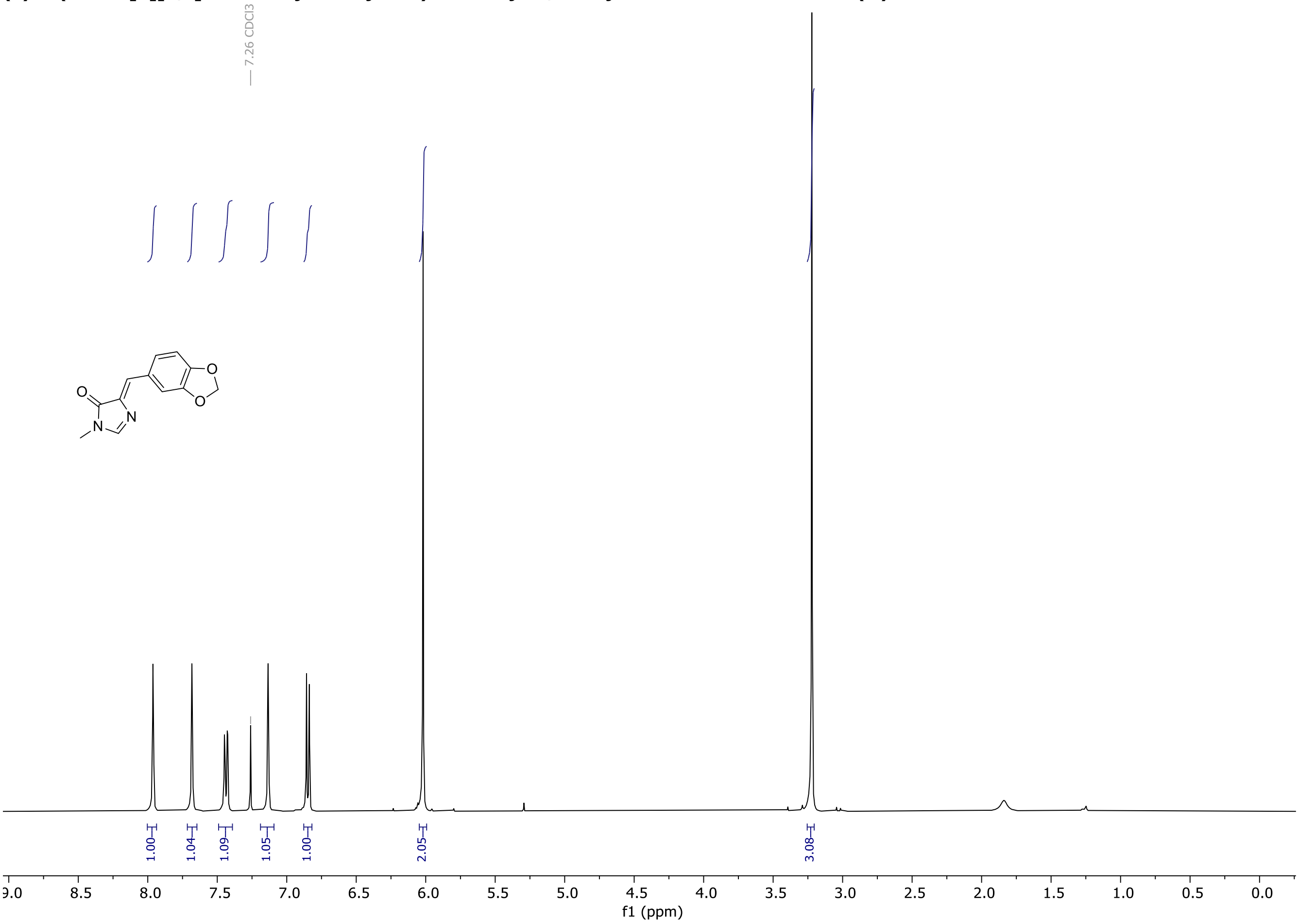

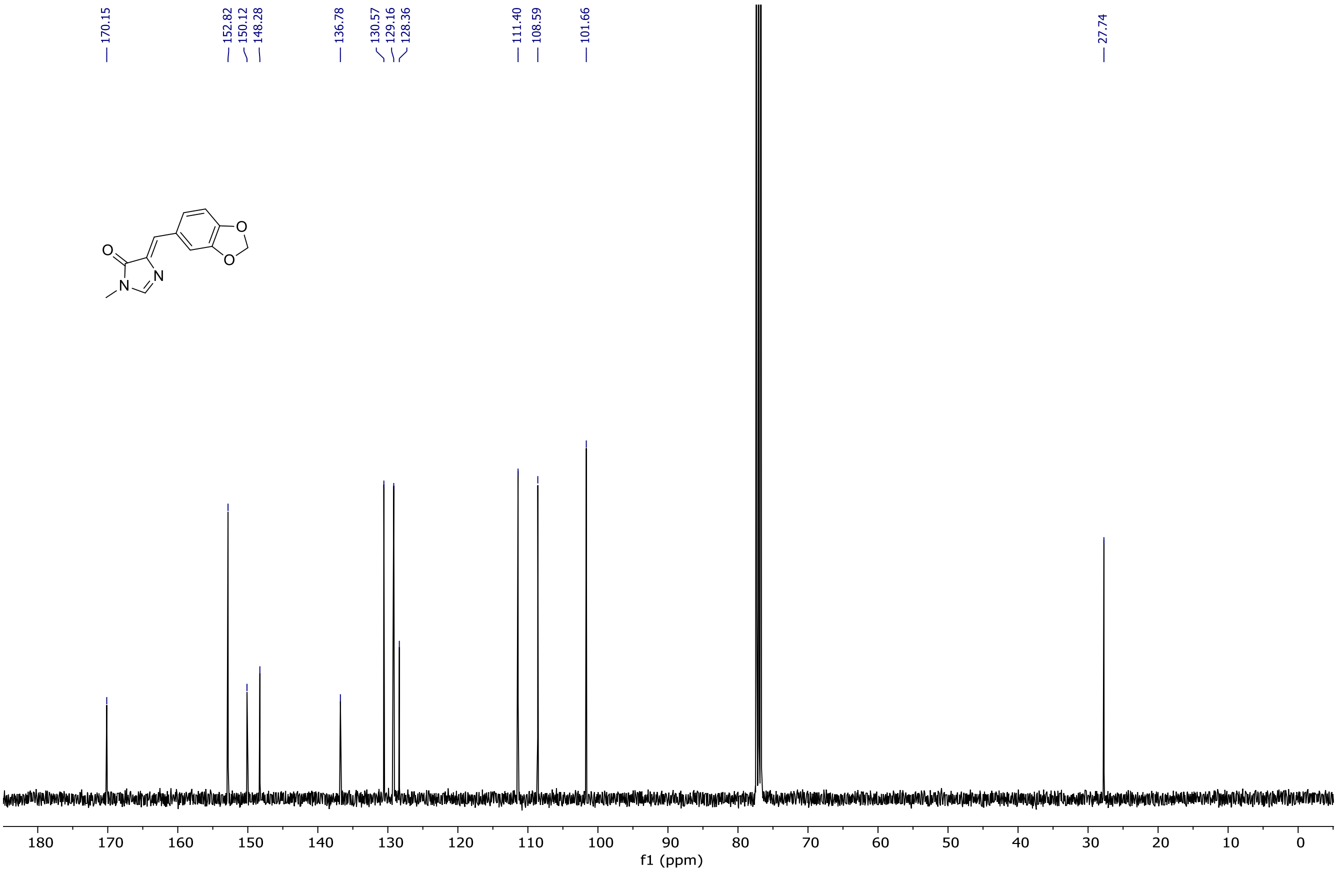

(*Z*)-5-(4-(Dimethylamino)benzylidene)-3-methyl-3,5-dihydro-4*H*-imidazol-4-one (5j)

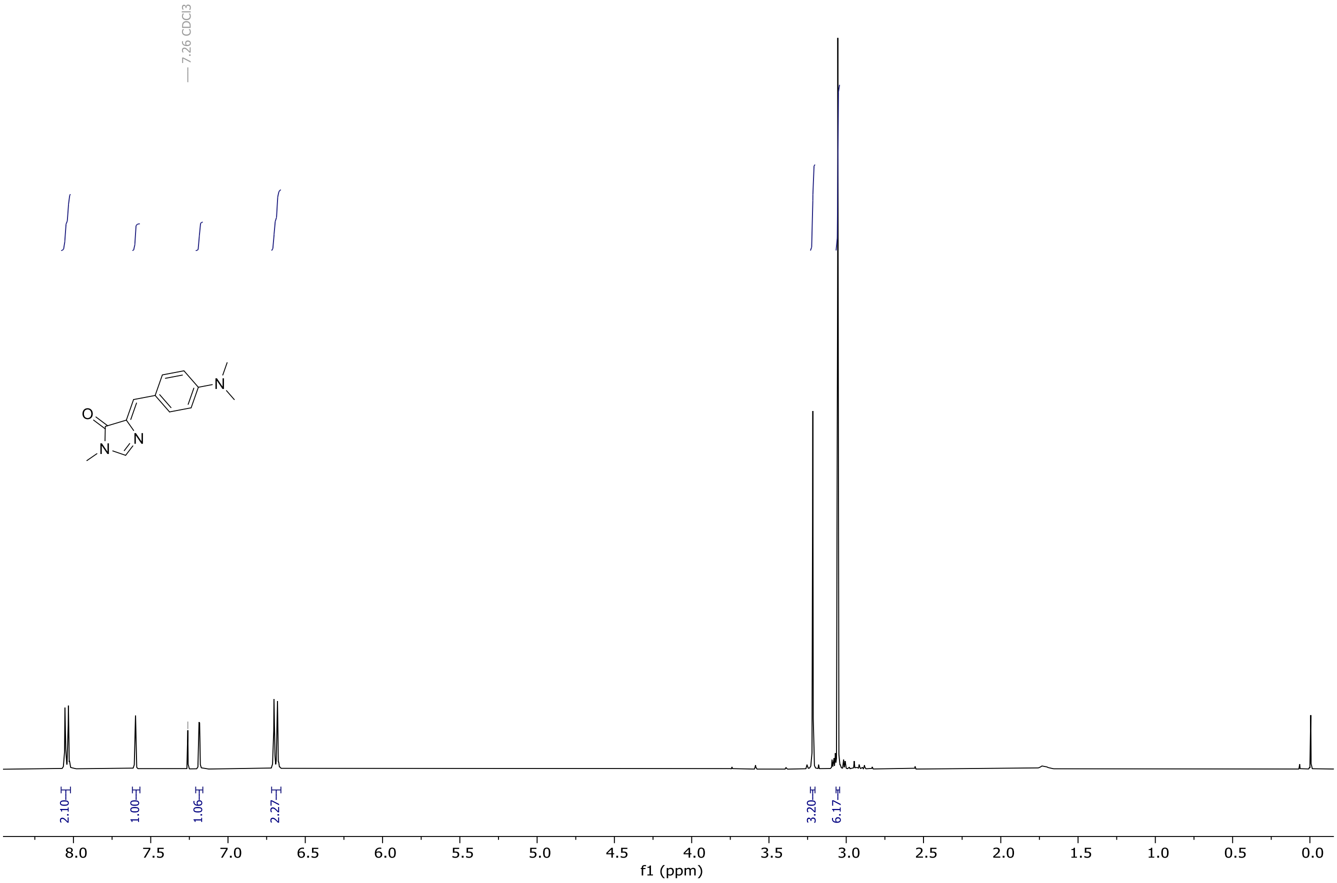

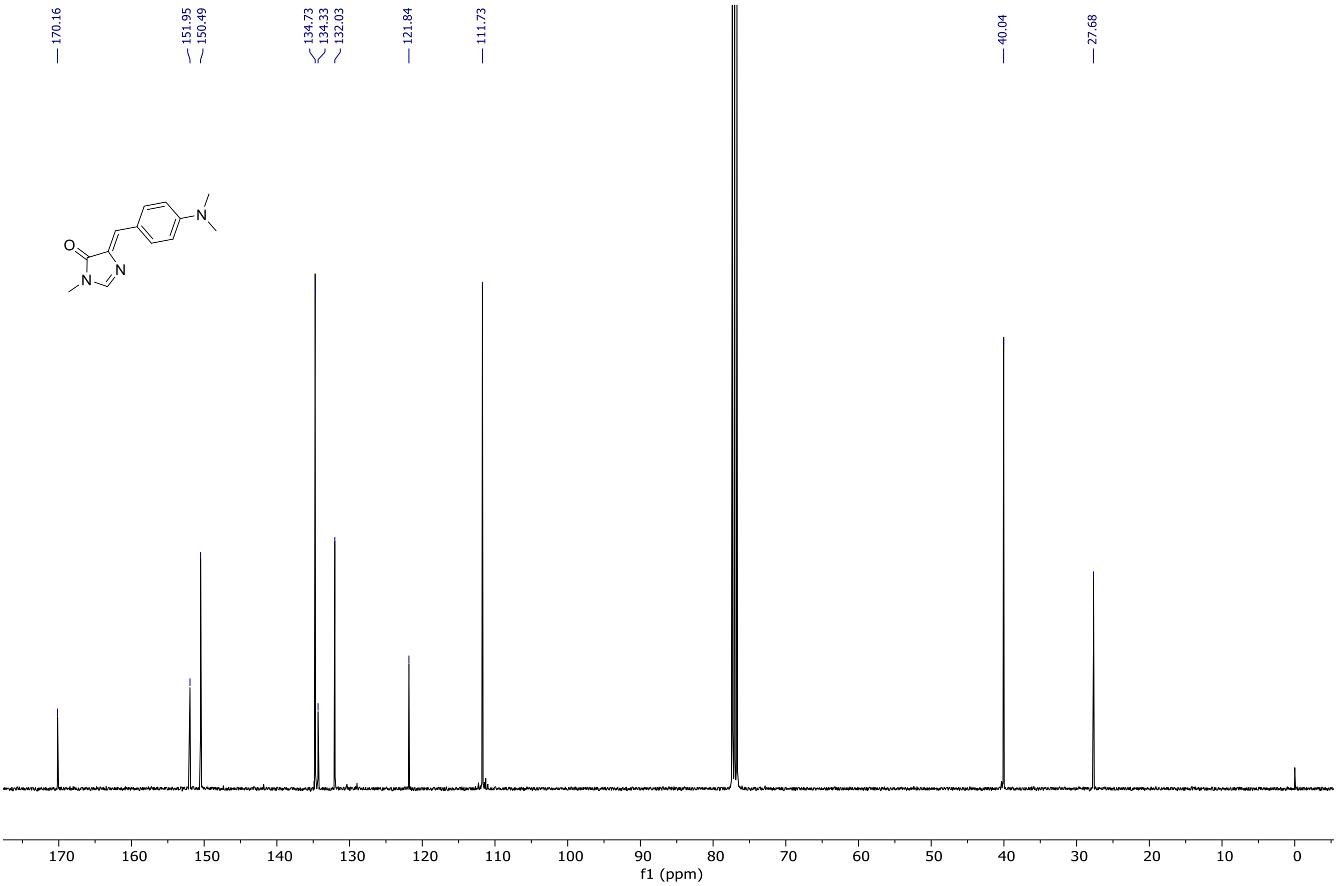

(*Z*)-3-benzyl-5-(thiophen-2-ylmethylene)-3,5-dihydro-4*H*-imidazol-4-one (5k)

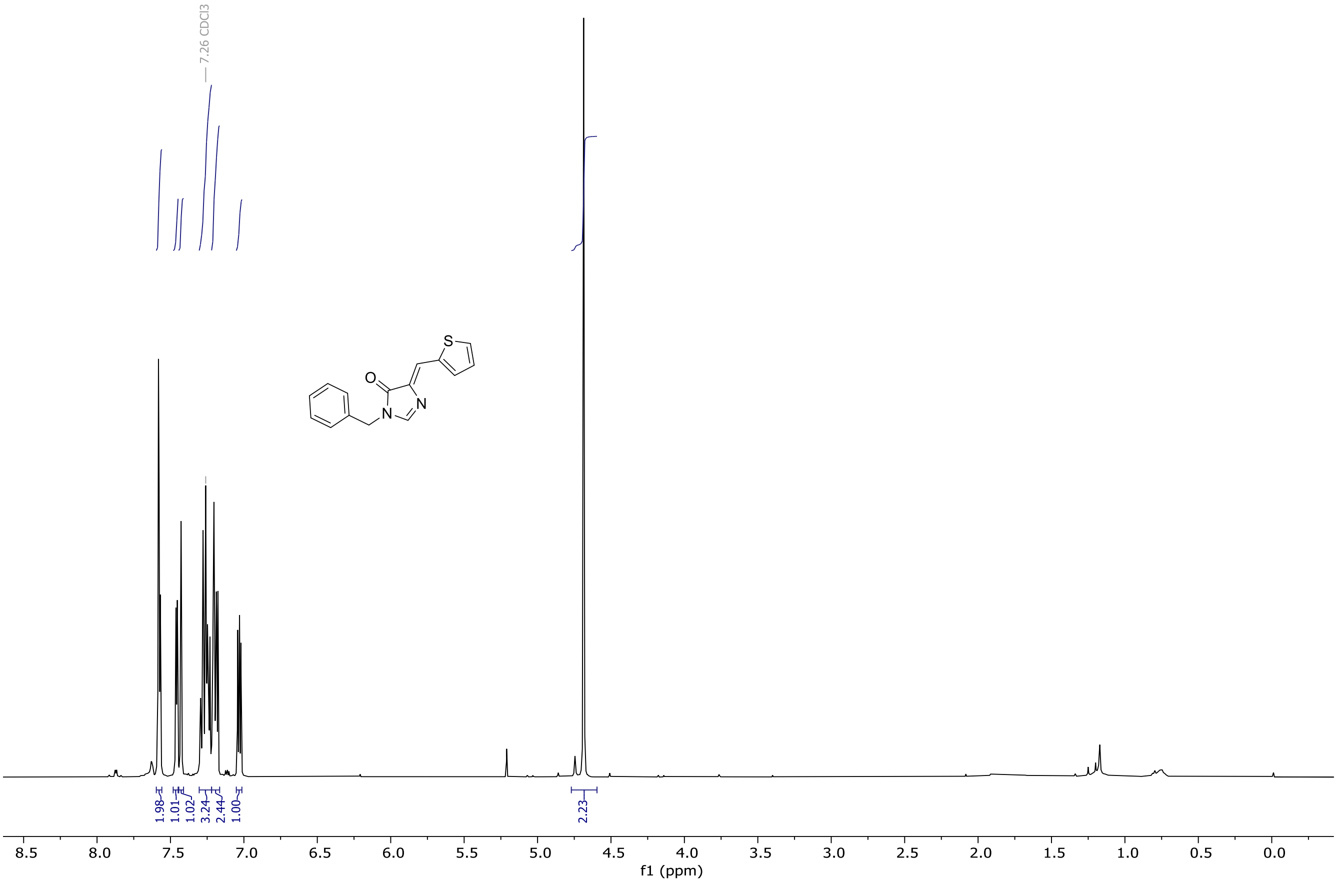

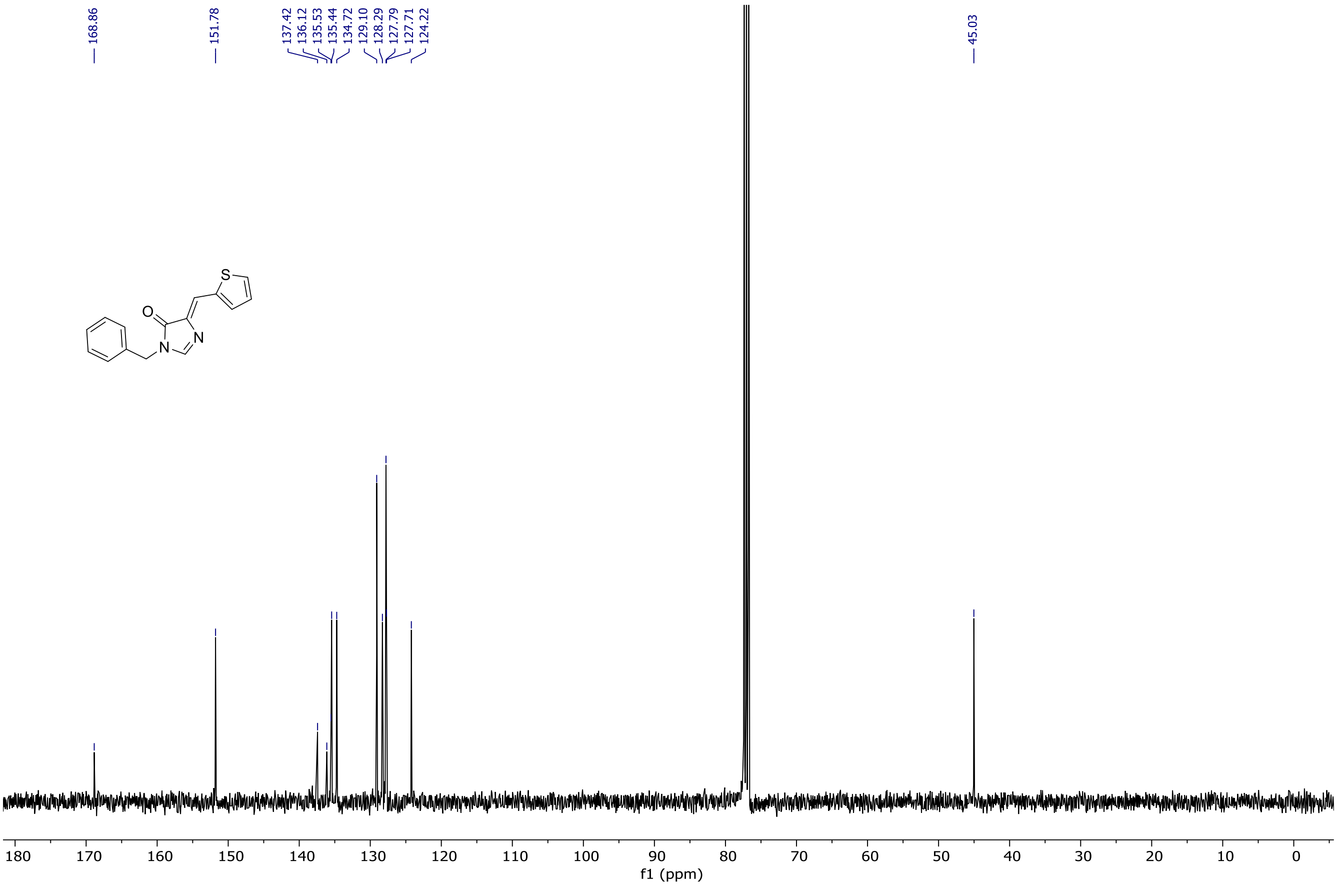

(*Z*)-3-(4-chlorobenzyl)-5-(thiophen-2-ylmethylene)-3,5-dihydro-4*H*-imidazol-4-one (5l)

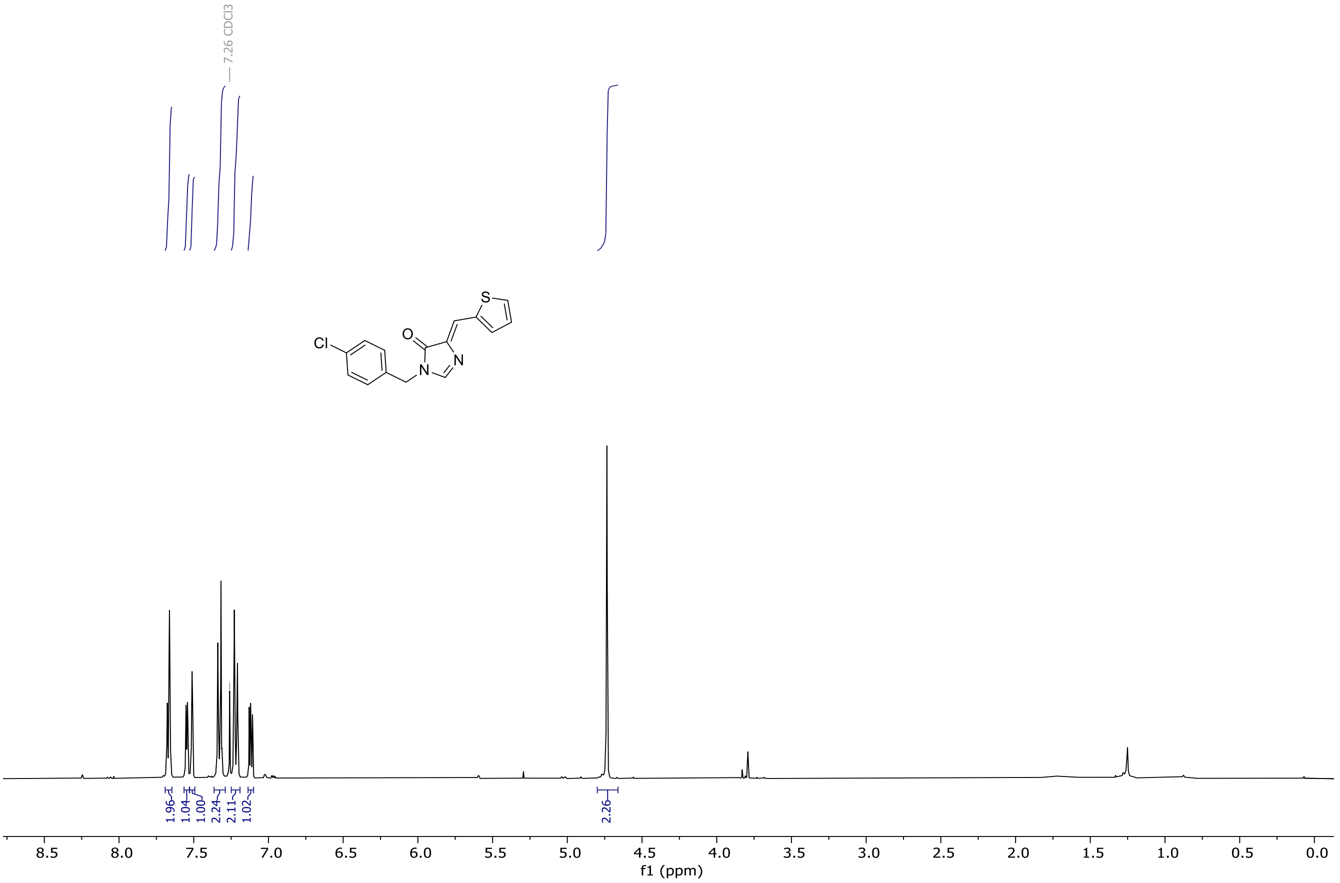

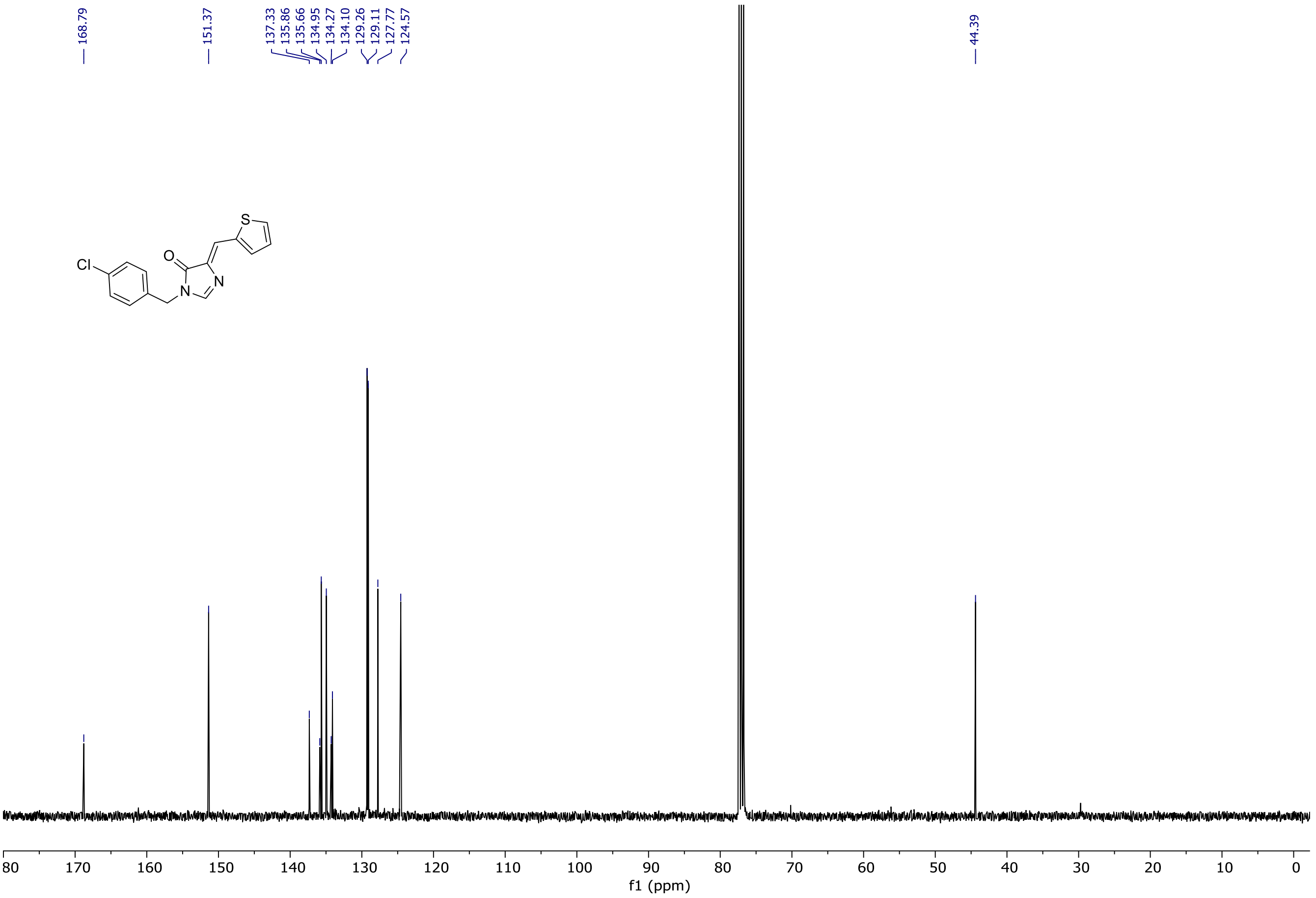

(*Z*)-3-methyl-5-(thiophen-2-ylmethylene)-3,5-dihydro-4*H*-imidazol-4-one (5m)

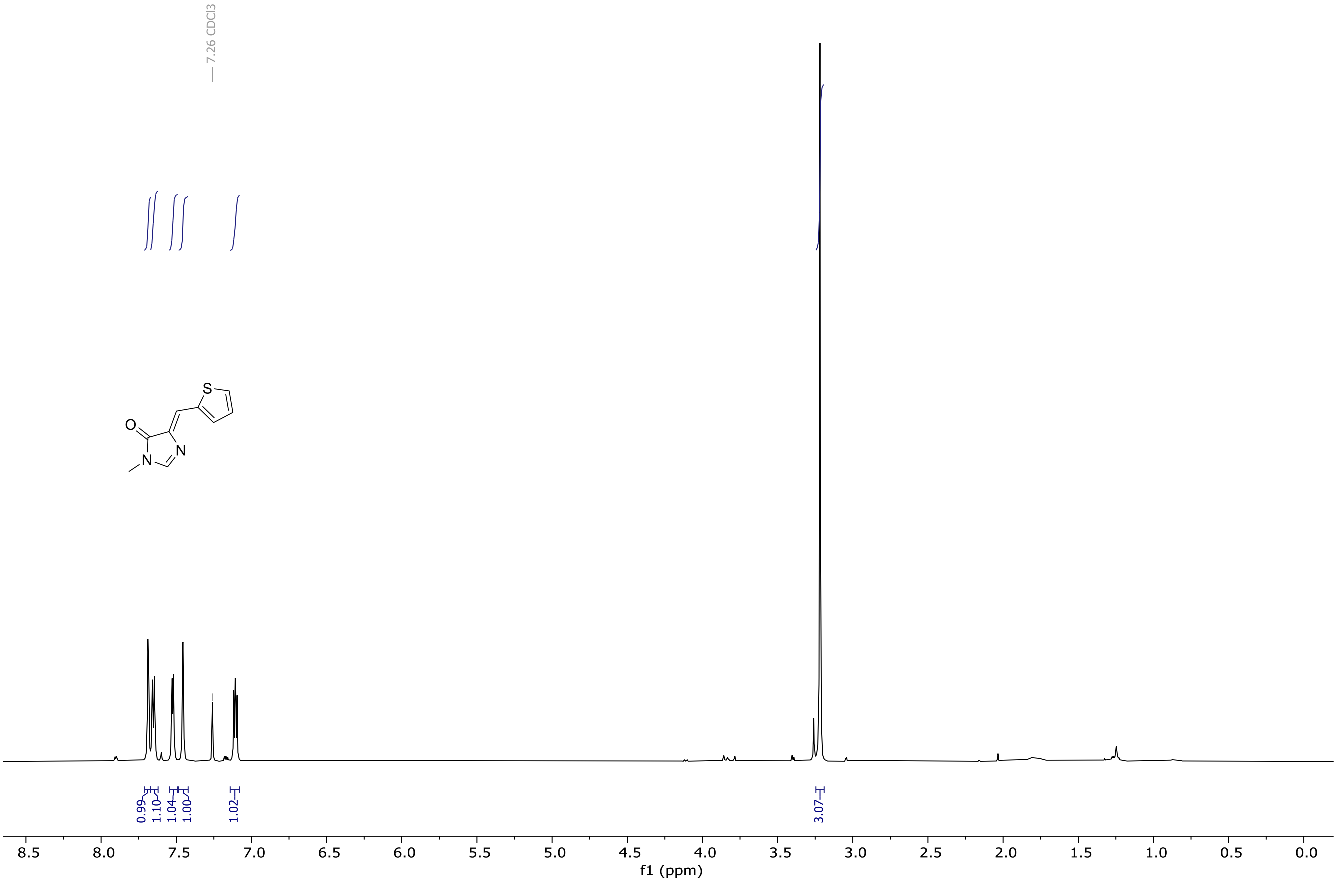

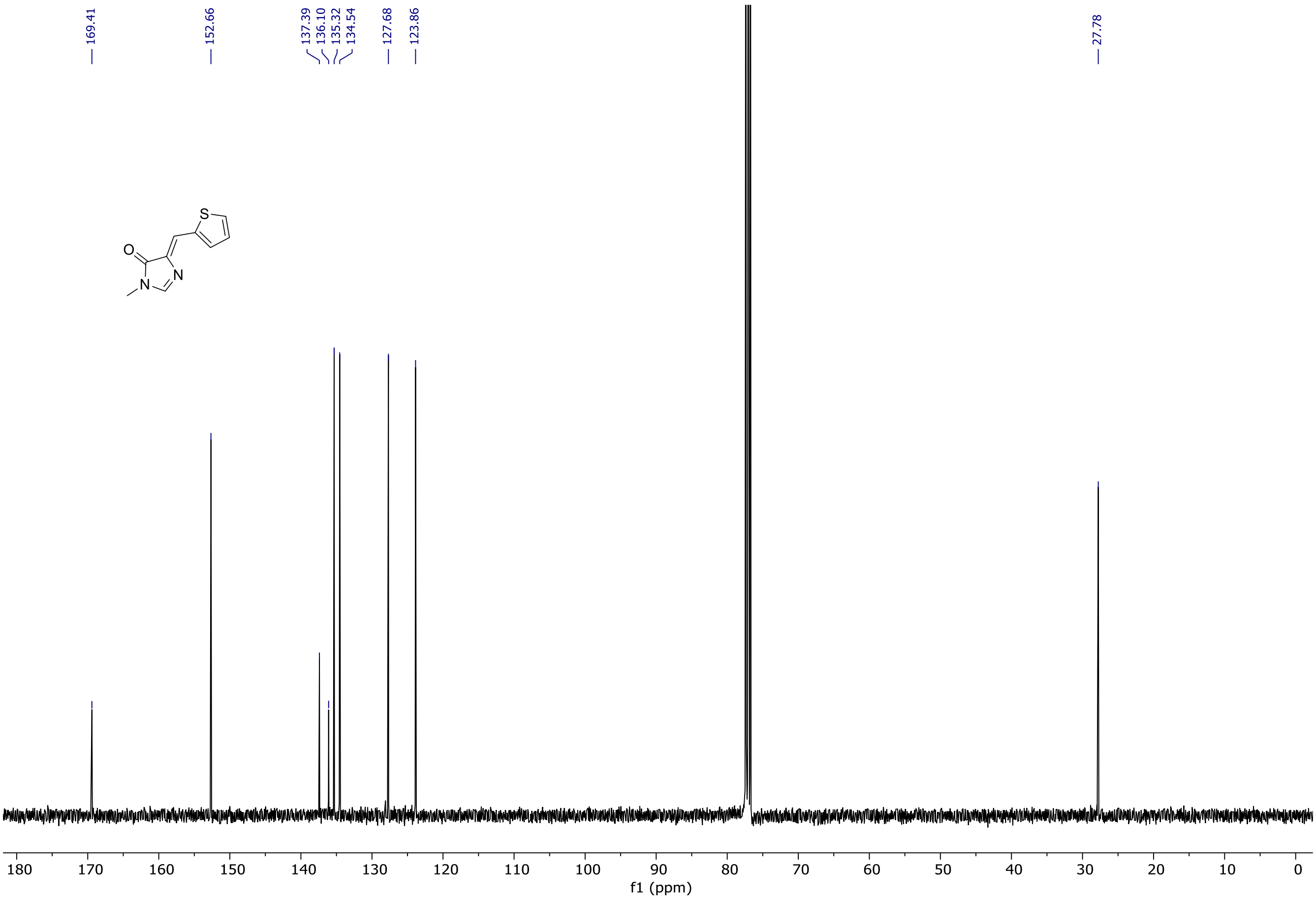

(*Z*)-5-(4-((*tert*-Butyldimethylsilyl)oxy)benzylidene)-3-(4-methylbenzyl)-3,5-dihydro-4*H*-imidazol-4-one (5n)

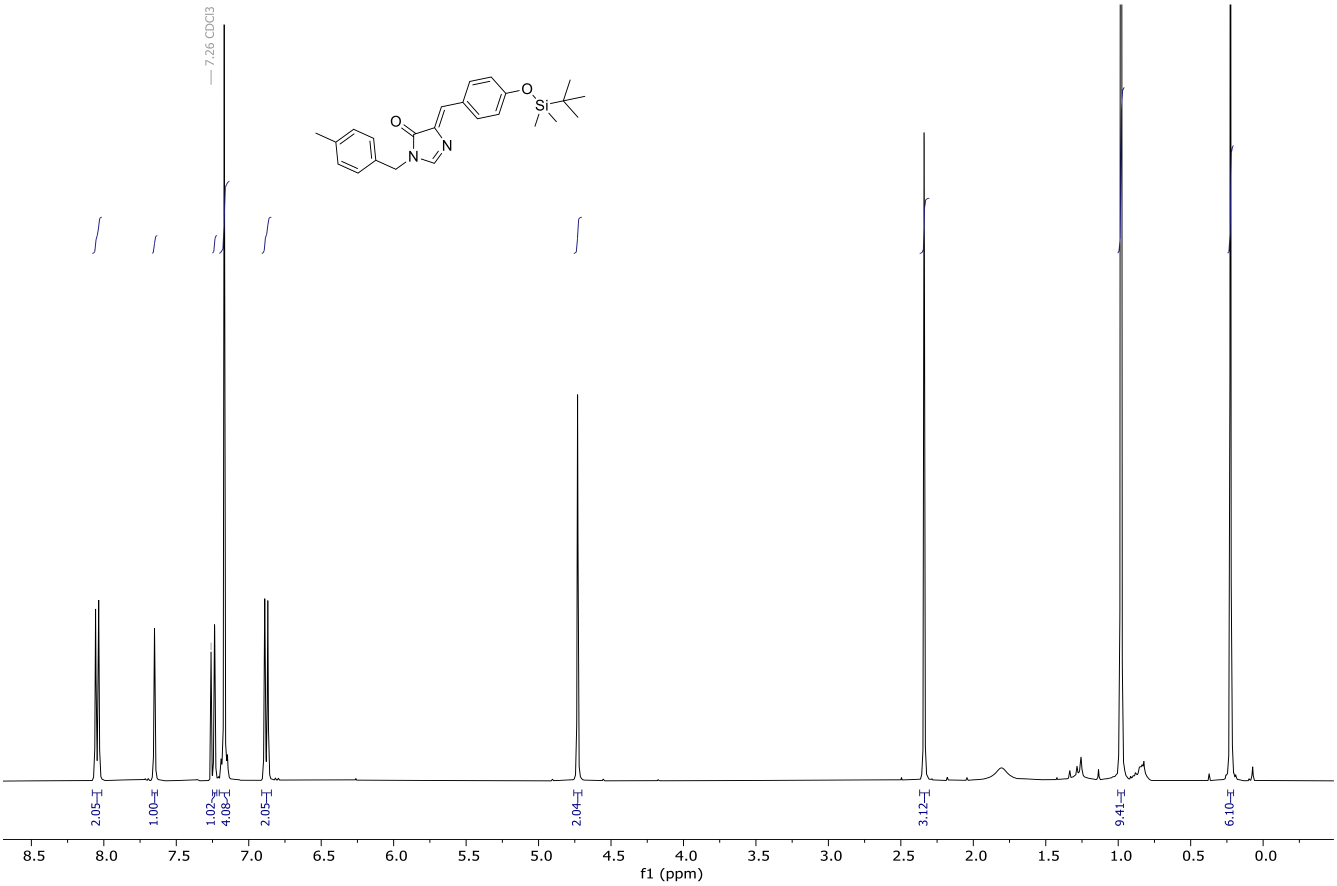

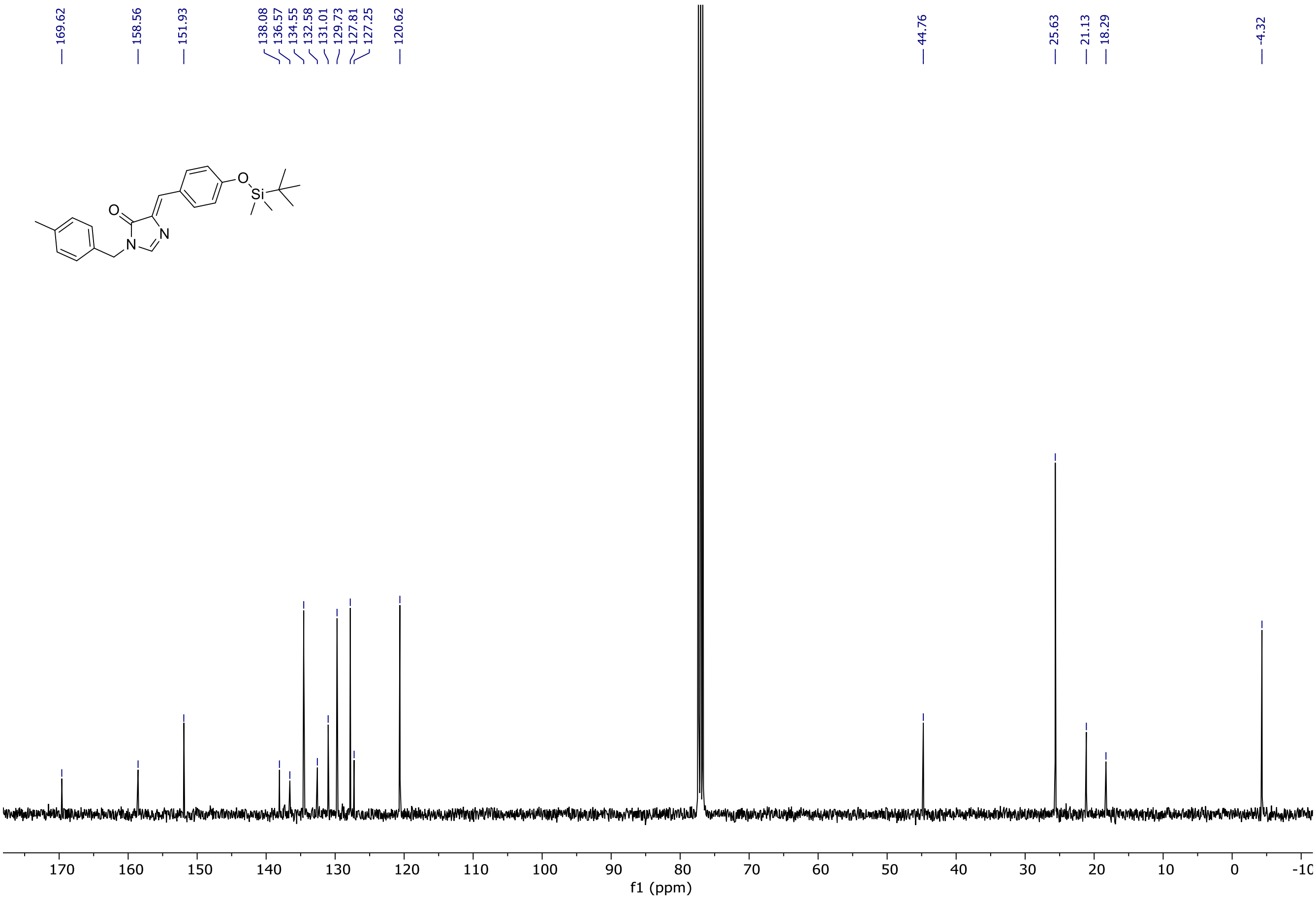

(*Z*)-5-(4-Hydroxybenzylidene)-3-(4-methylbenzyl)-3,5-dihydro-4*H*-imidazol-4-one (5n')

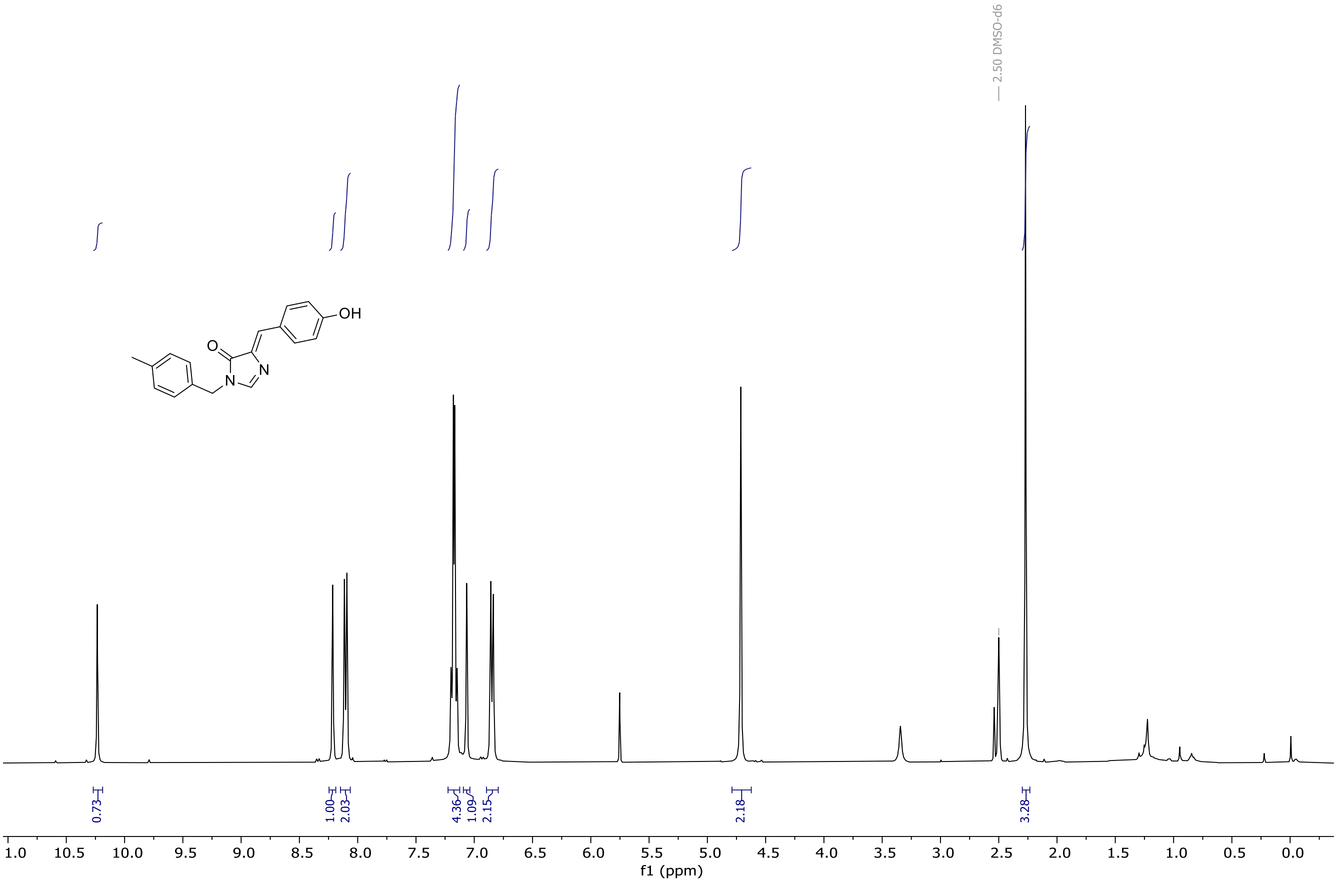

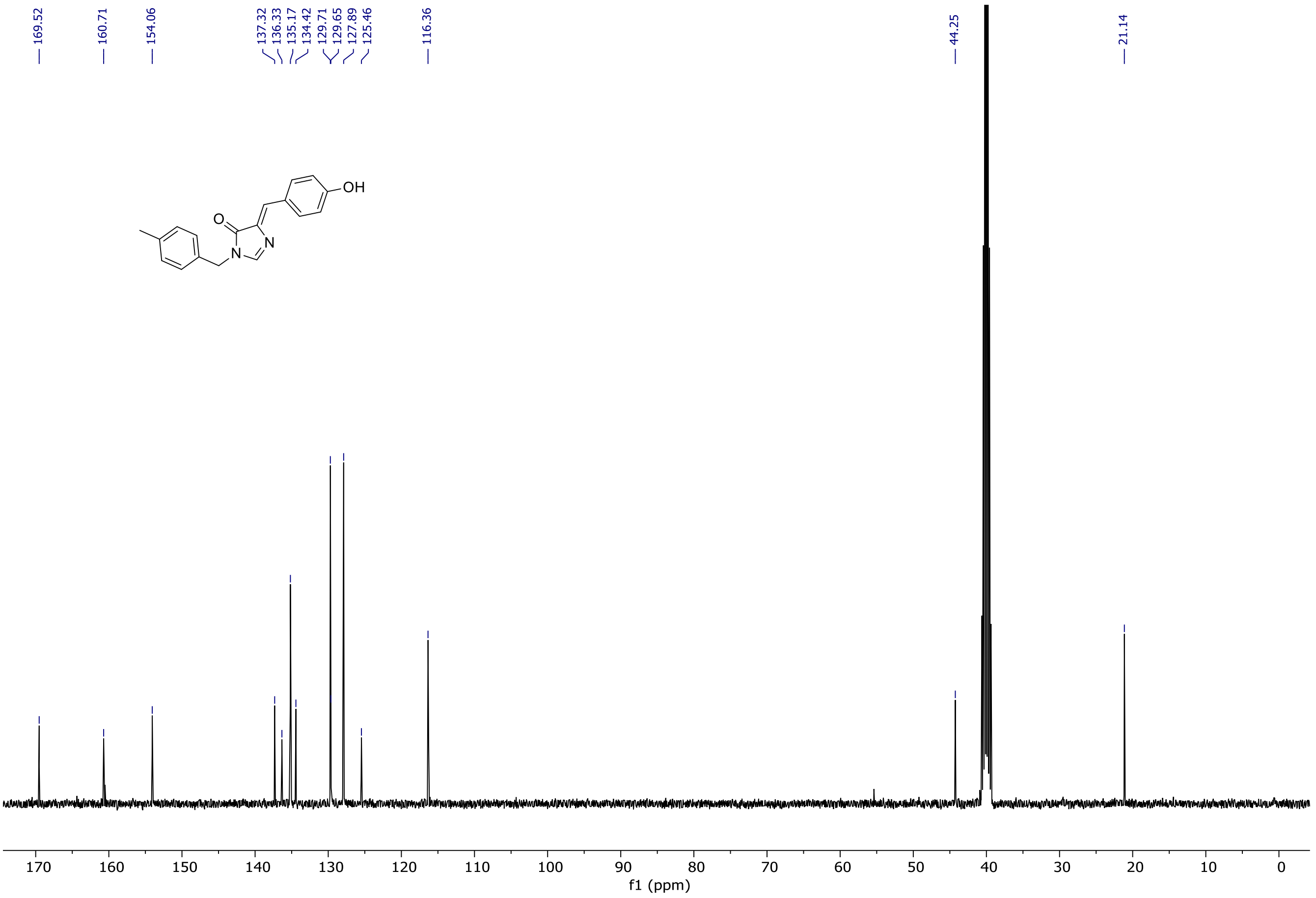

Tert-butyl (_Z_)-3-((1-methyl-5-oxo-1,5-dihydro-4_H_-imidazol-4-ylidene)methyl)-1_H_-indole-1-carboxylate (5o')

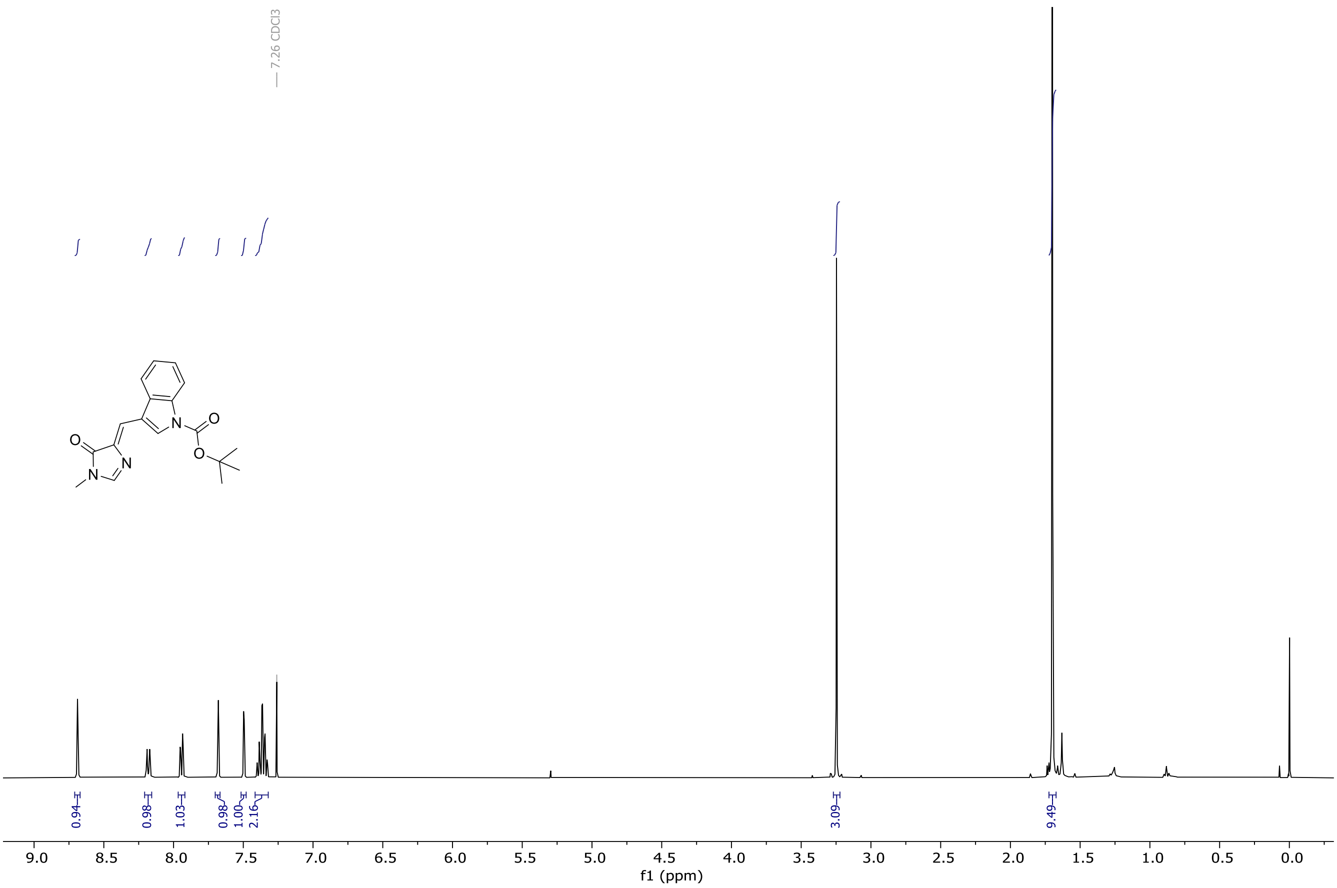

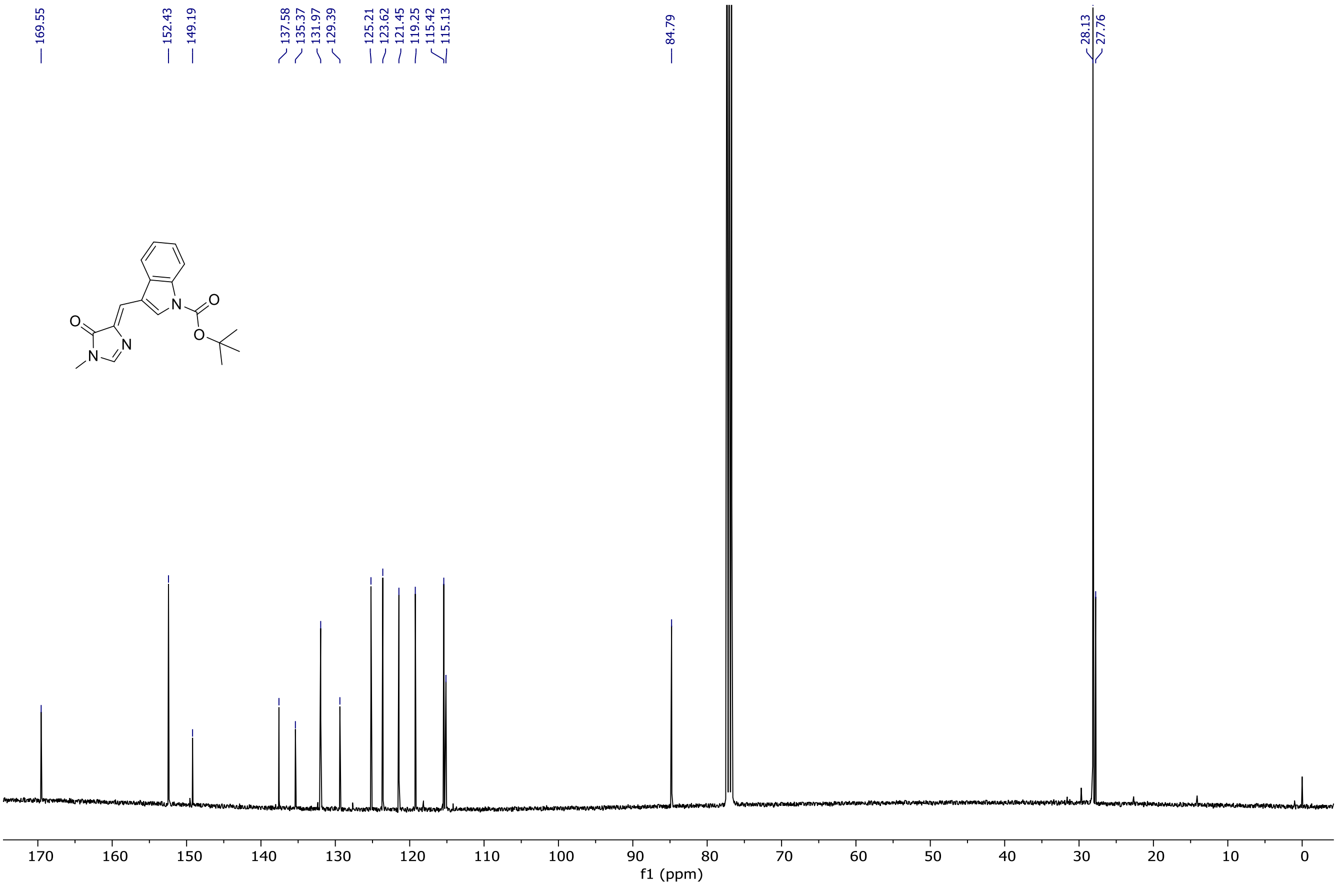

(*Z*)-5-((1H-indol-3-yl)methylene)-3-methyl-3,5-dihydro-4H-imidazol-4-one (5o')

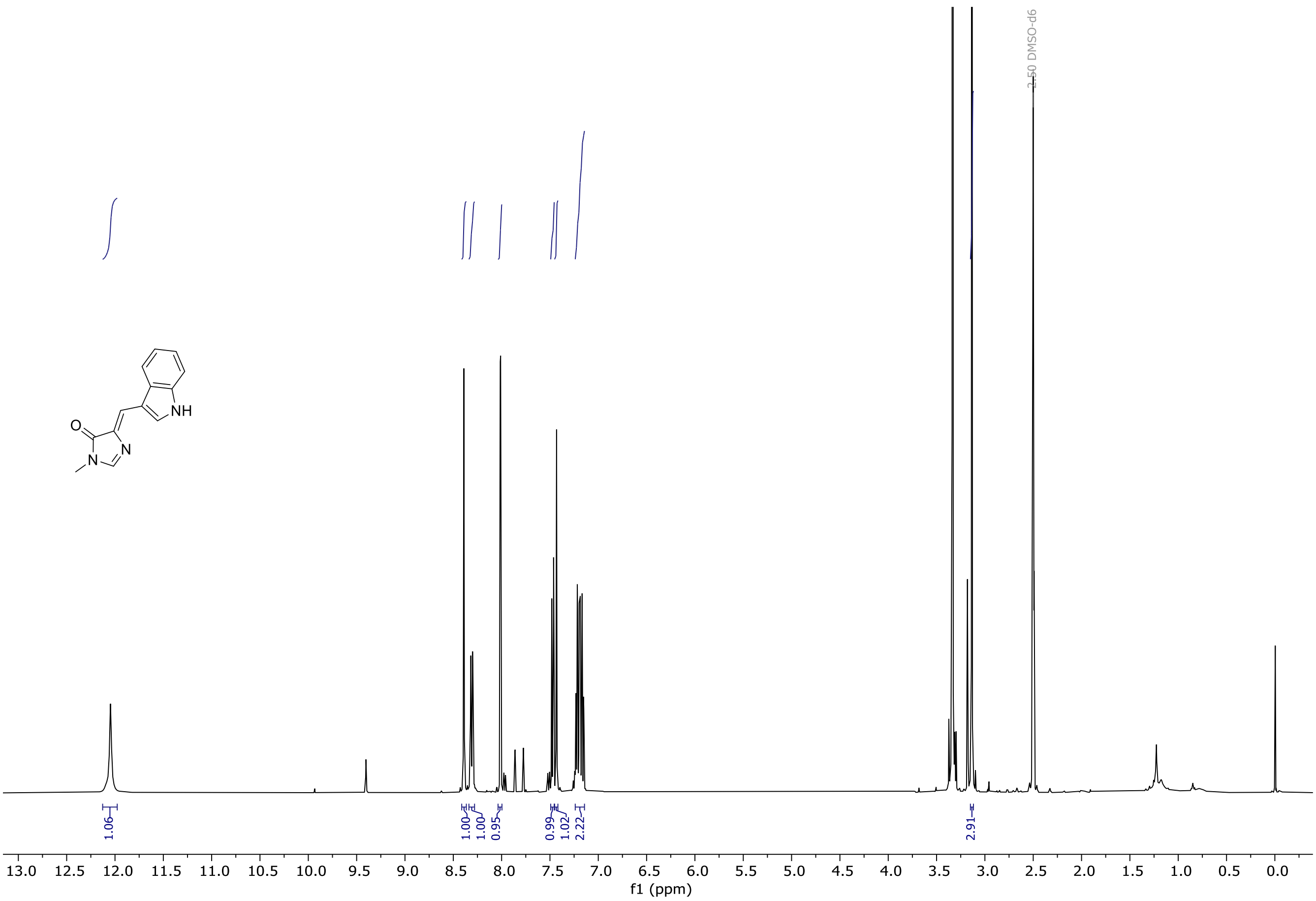

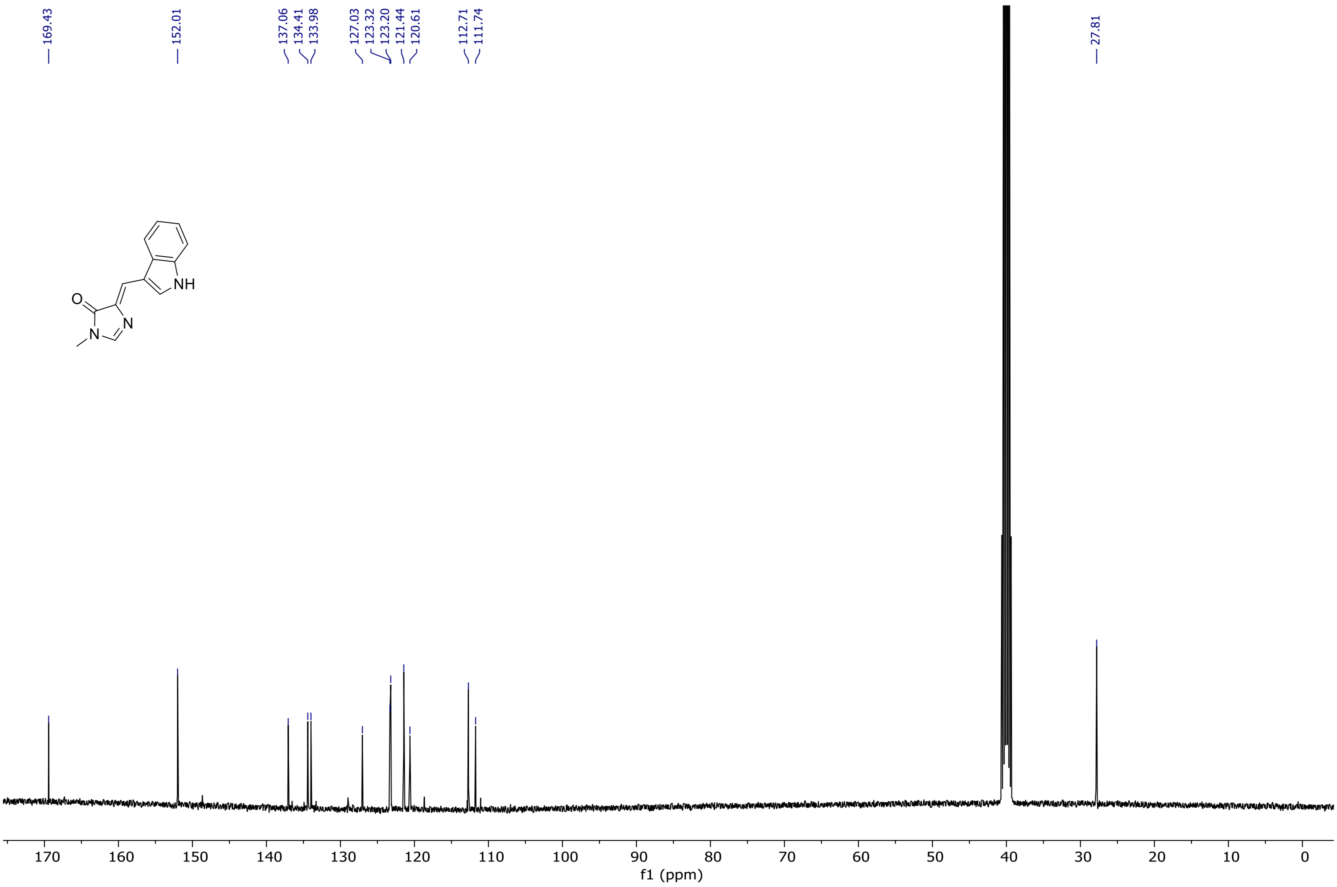

(*Z*)-3-(4-Methylbenzyl)-5-((1-tosyl-1*H*-indol-3-yl)methylene)-3,5-dihydro-4*H*-imidazol-4-one (5p)

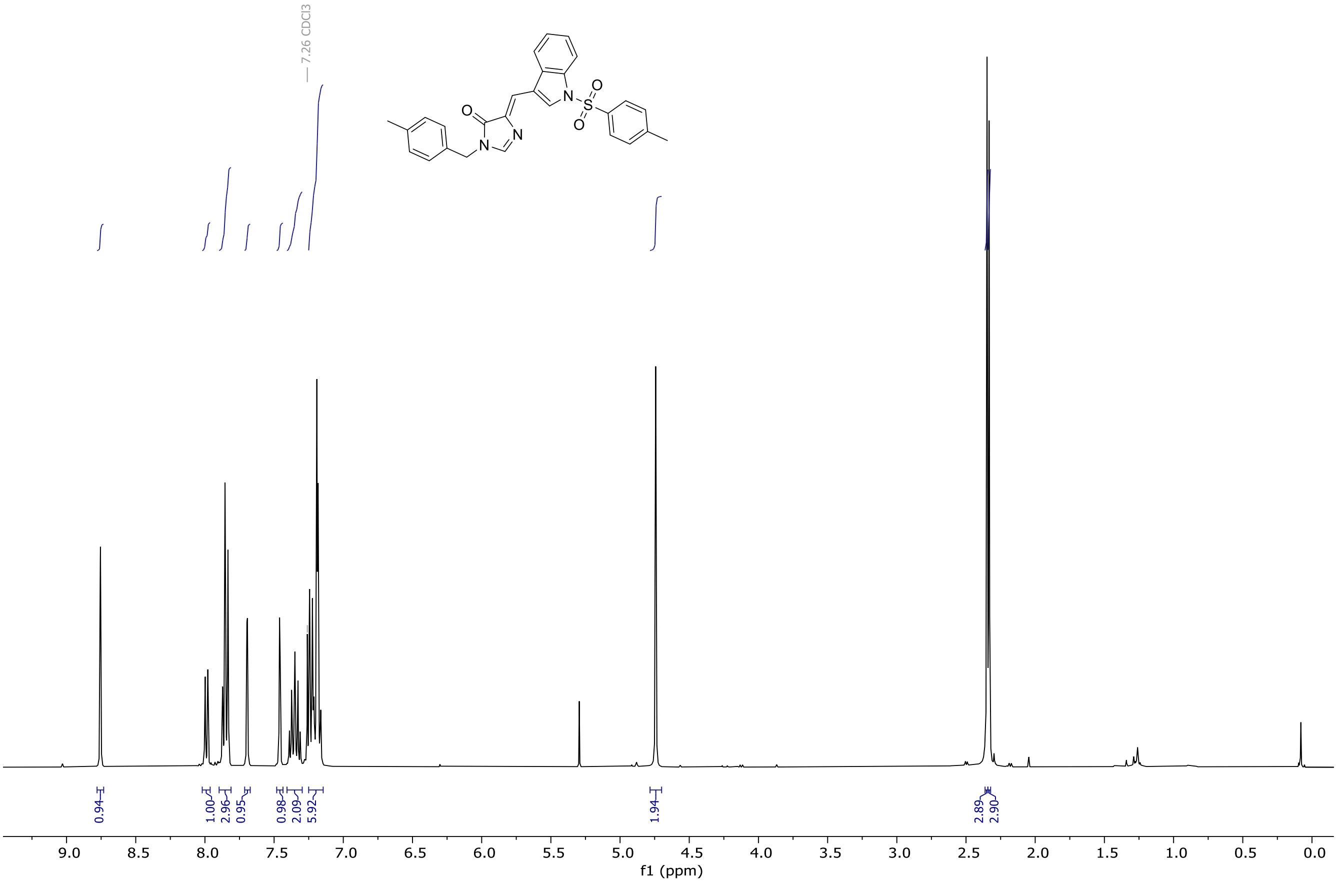

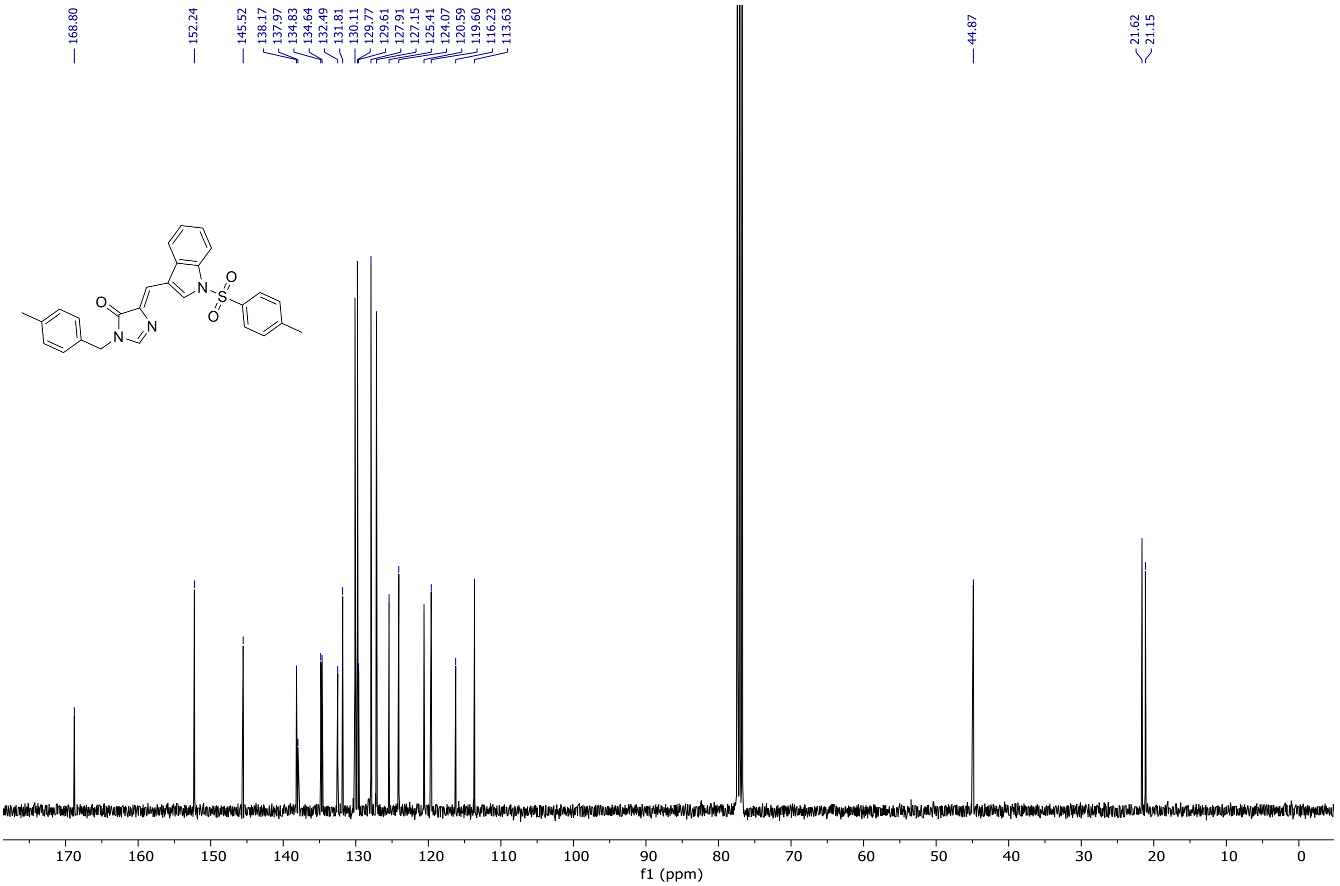

(*Z*)-3-(4-methylbenzyl)-5-(pyridin-4-ylmethylene)-3,5-dihydro-4*H*-imidazol-4-one (5q)

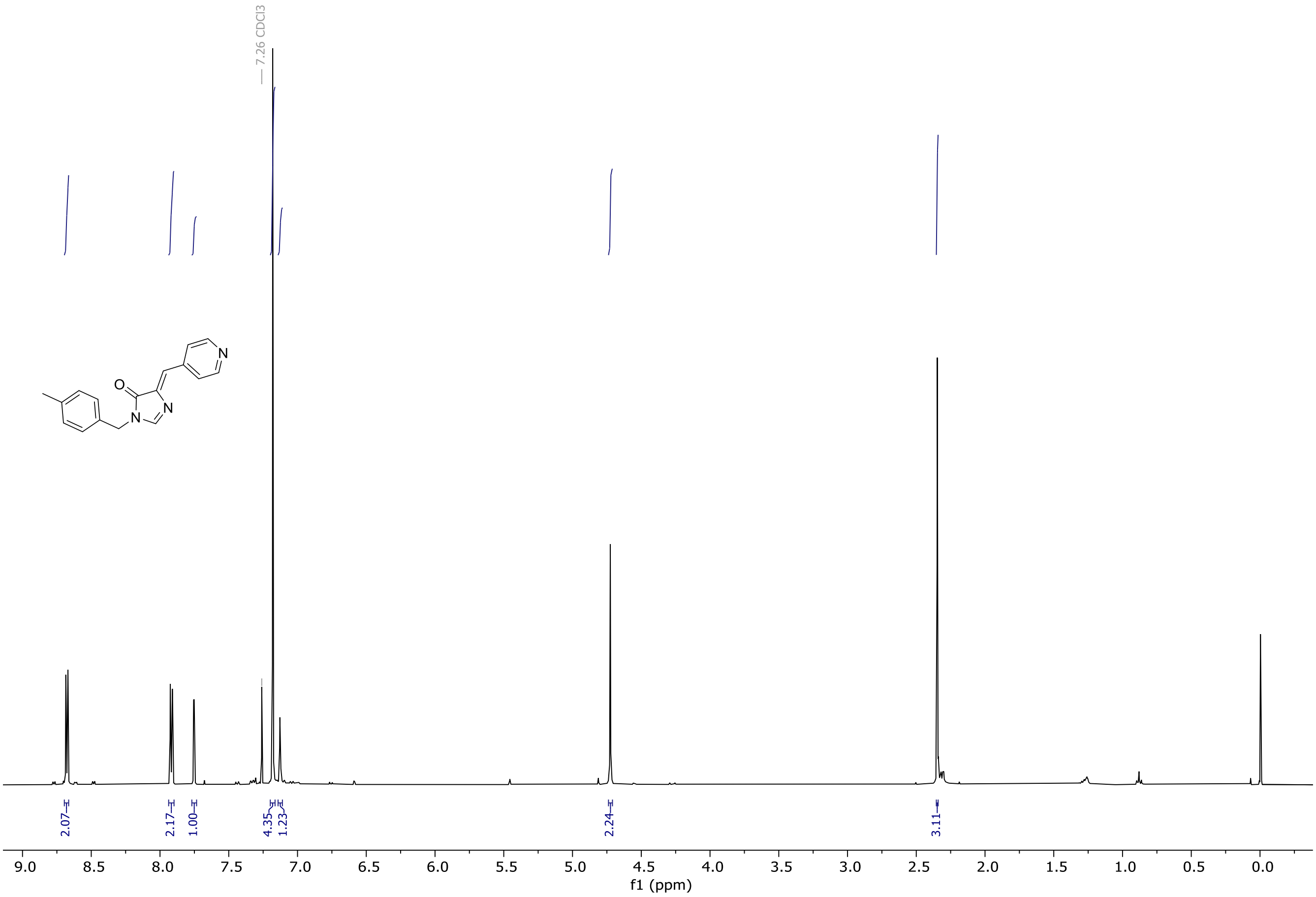

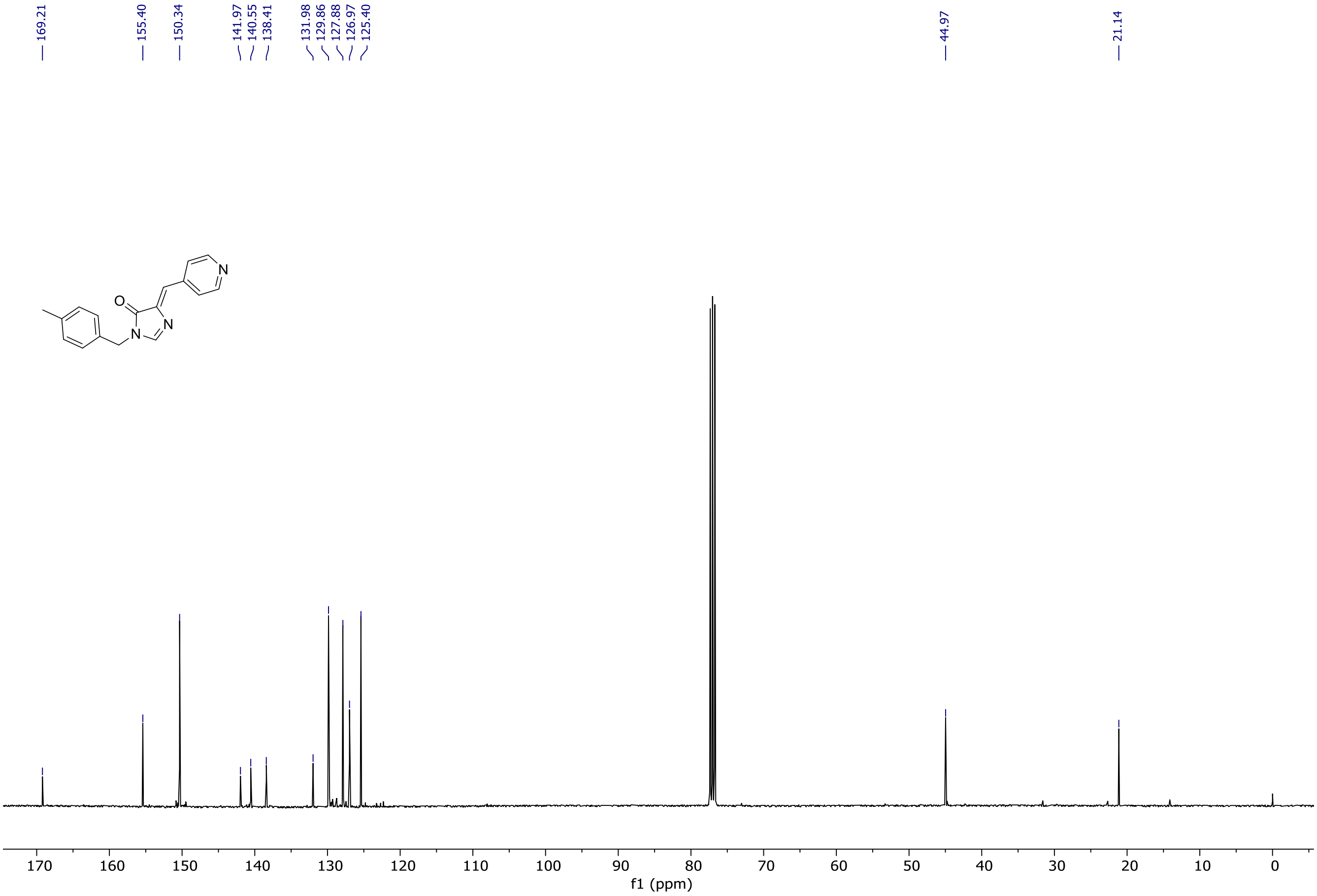

(*Z*)-5-((1*H*-indol-2-yl)methylene)-3-(4-methylbenzyl)-3,5-dihydro-4*H*-imidazol-4-one (5r)

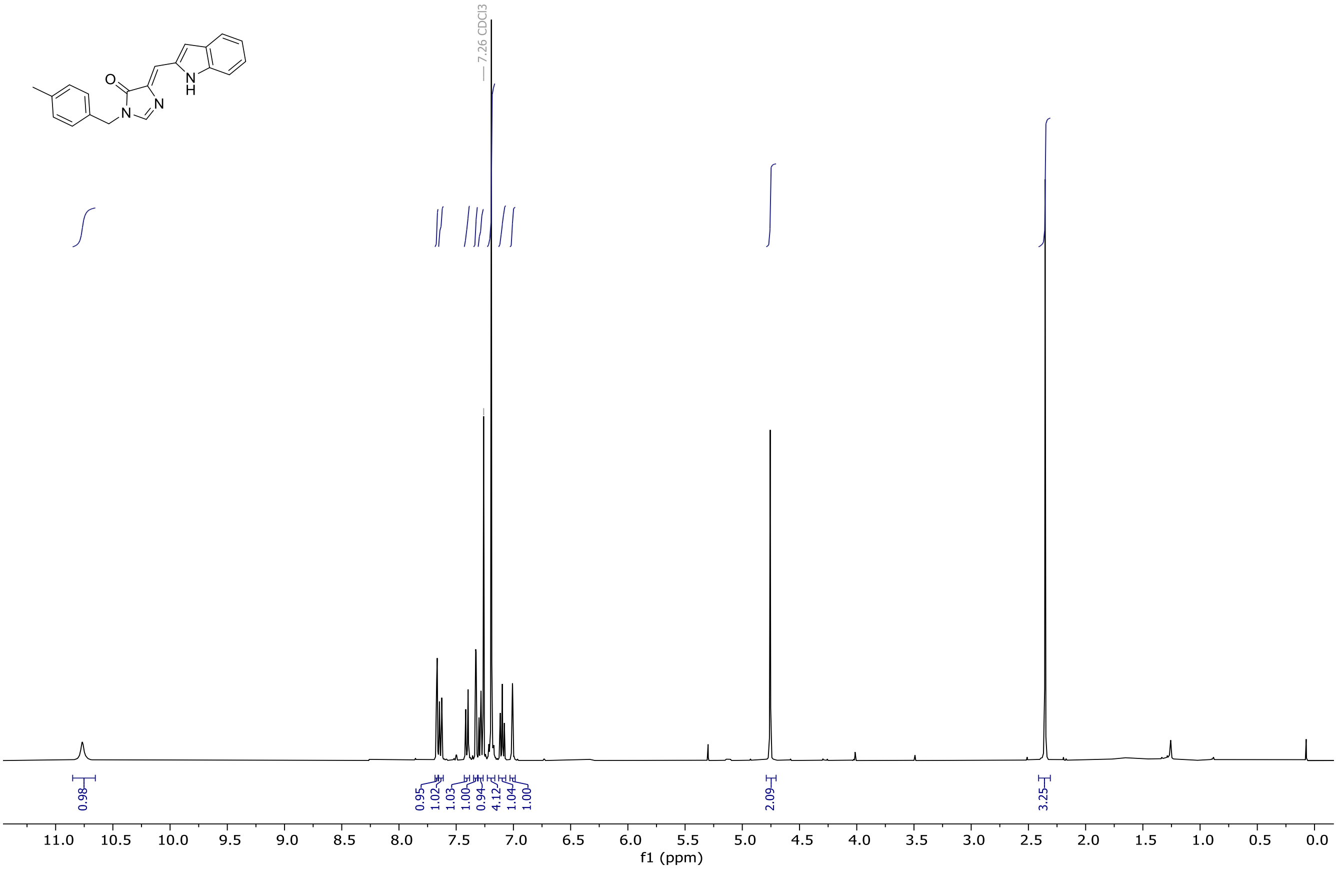

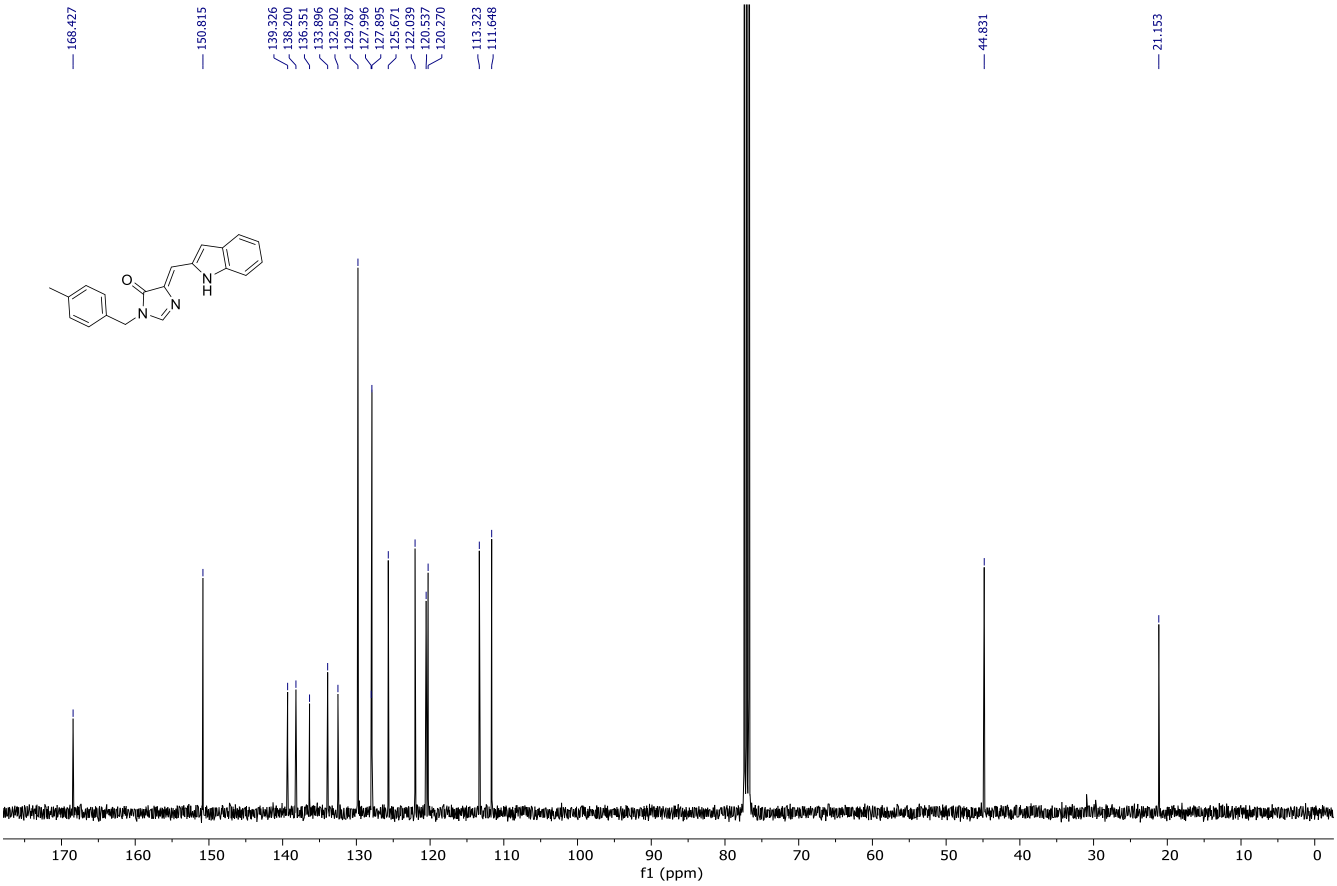

(*Z*)-3-Benzyl-5-((*E*)-3-(4-chlorophenyl)allylidene)-3,5-dihydro-4*H*-imidazol-4-one (5s)

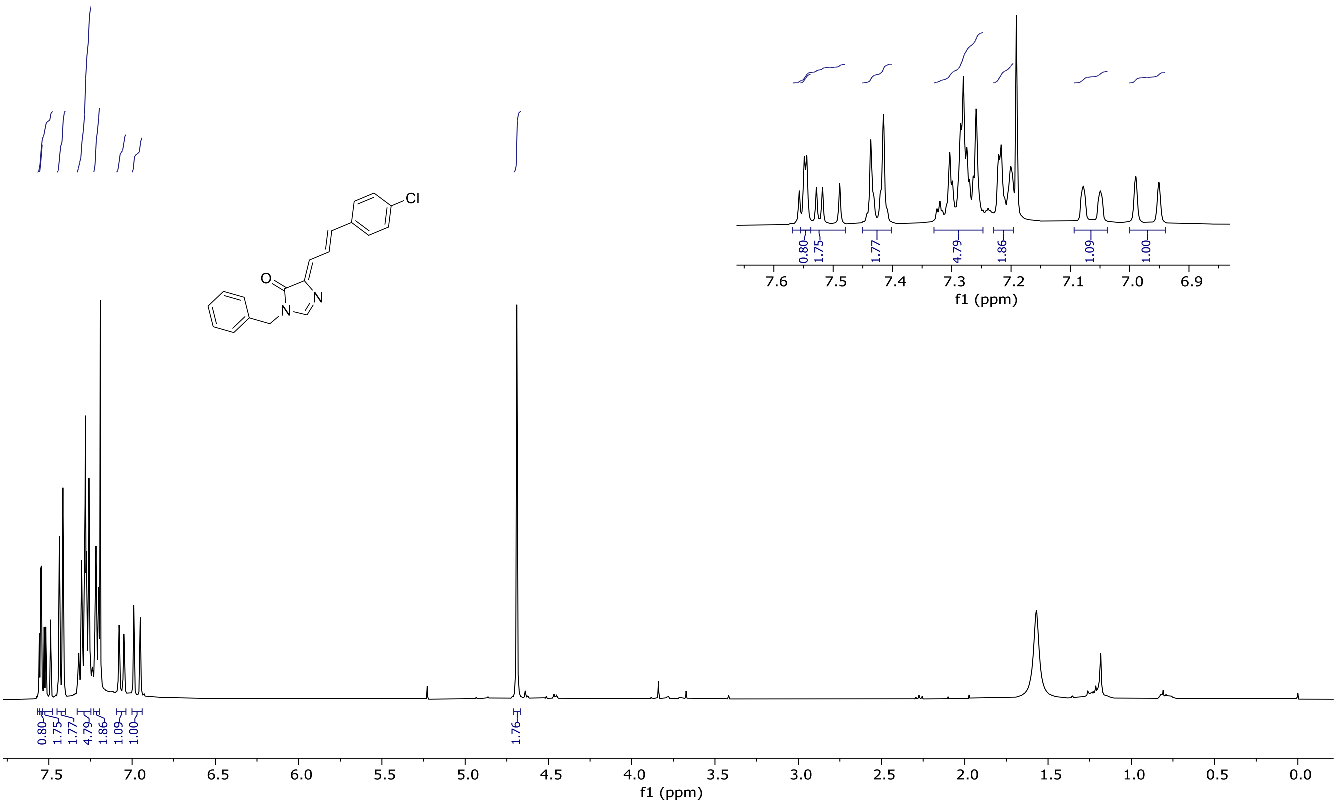

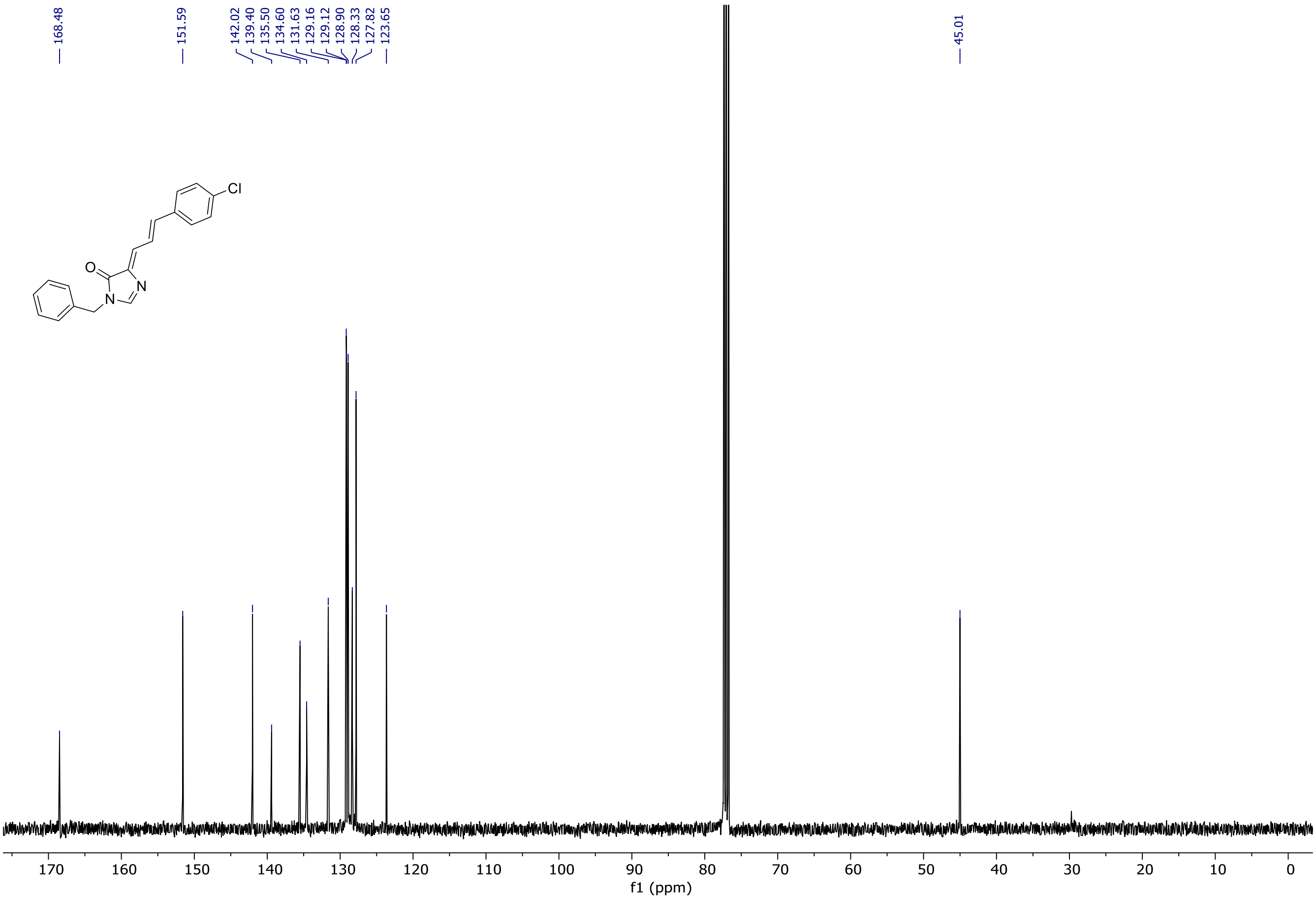

(*Z*)-3-butyl-5-(thiophen-2-ylmethylene)-3,5-dihydro-4*H*-imidazol-4-one (5t)

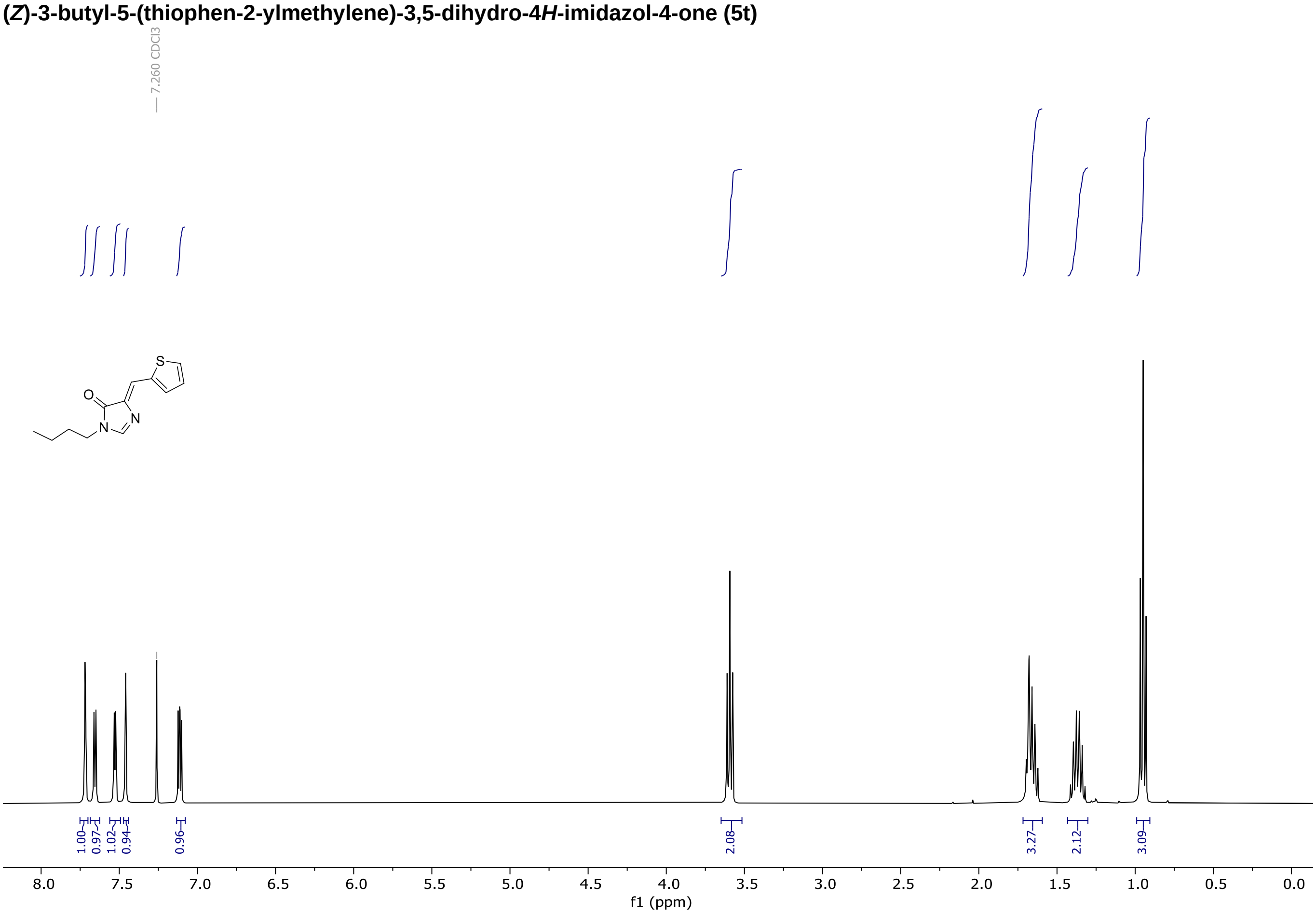

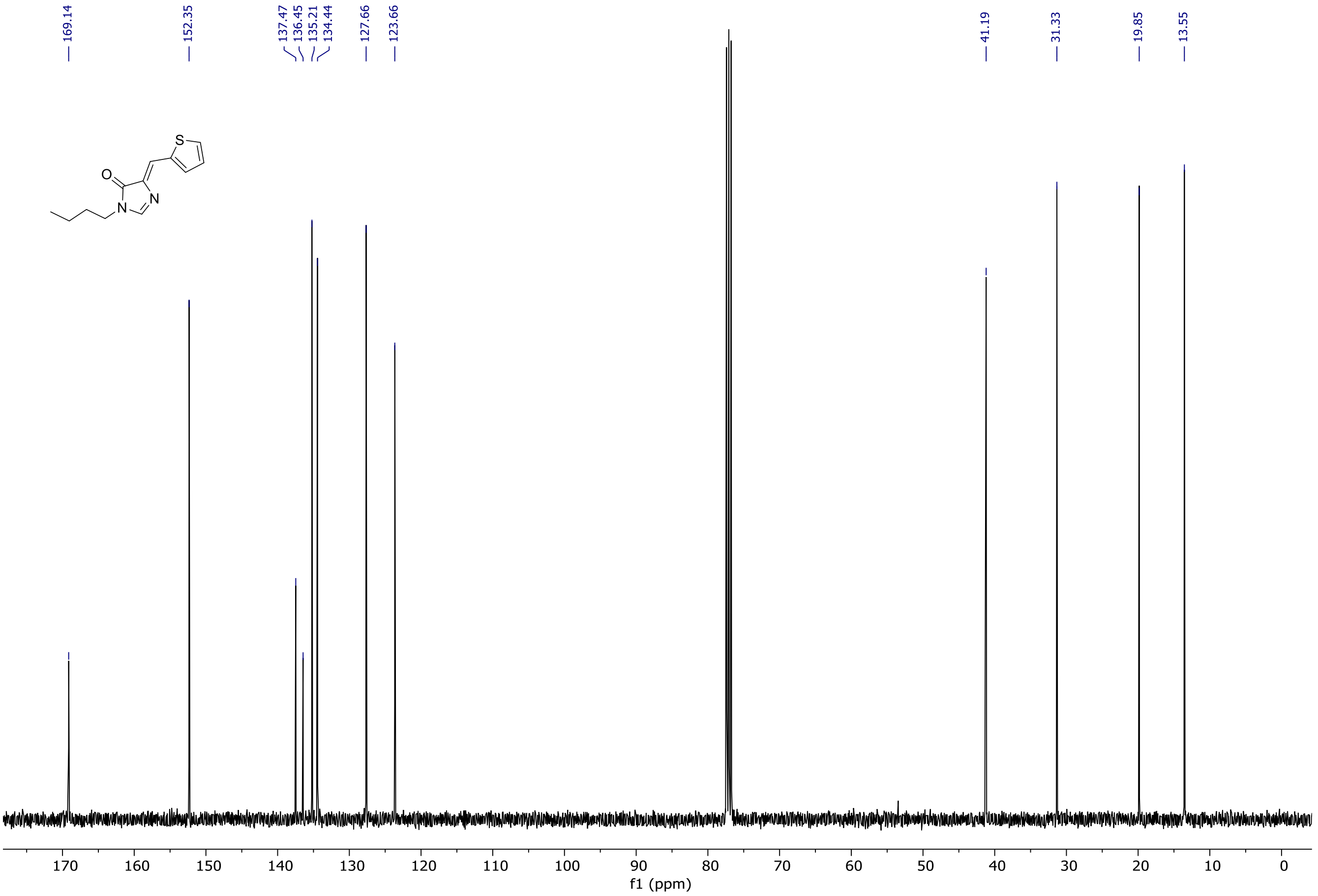

(*Z*)-3-(2-(1*H*-Indol-3-yl)ethyl)-5-(benzo[*d*][1,3]dioxol-5-ylmethylene)-3,5-dihydro-4*H*-imidazol-4-one (5u)

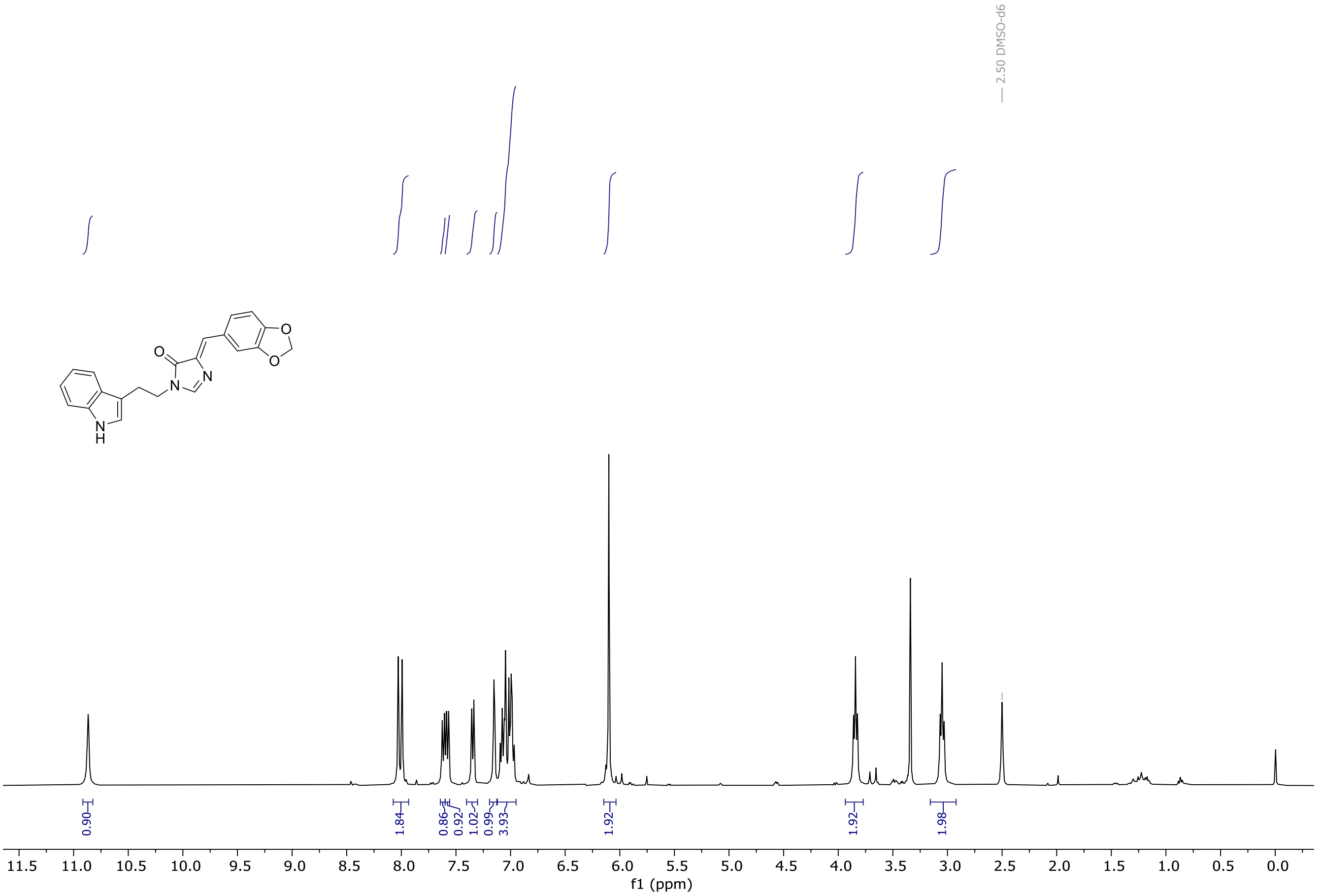

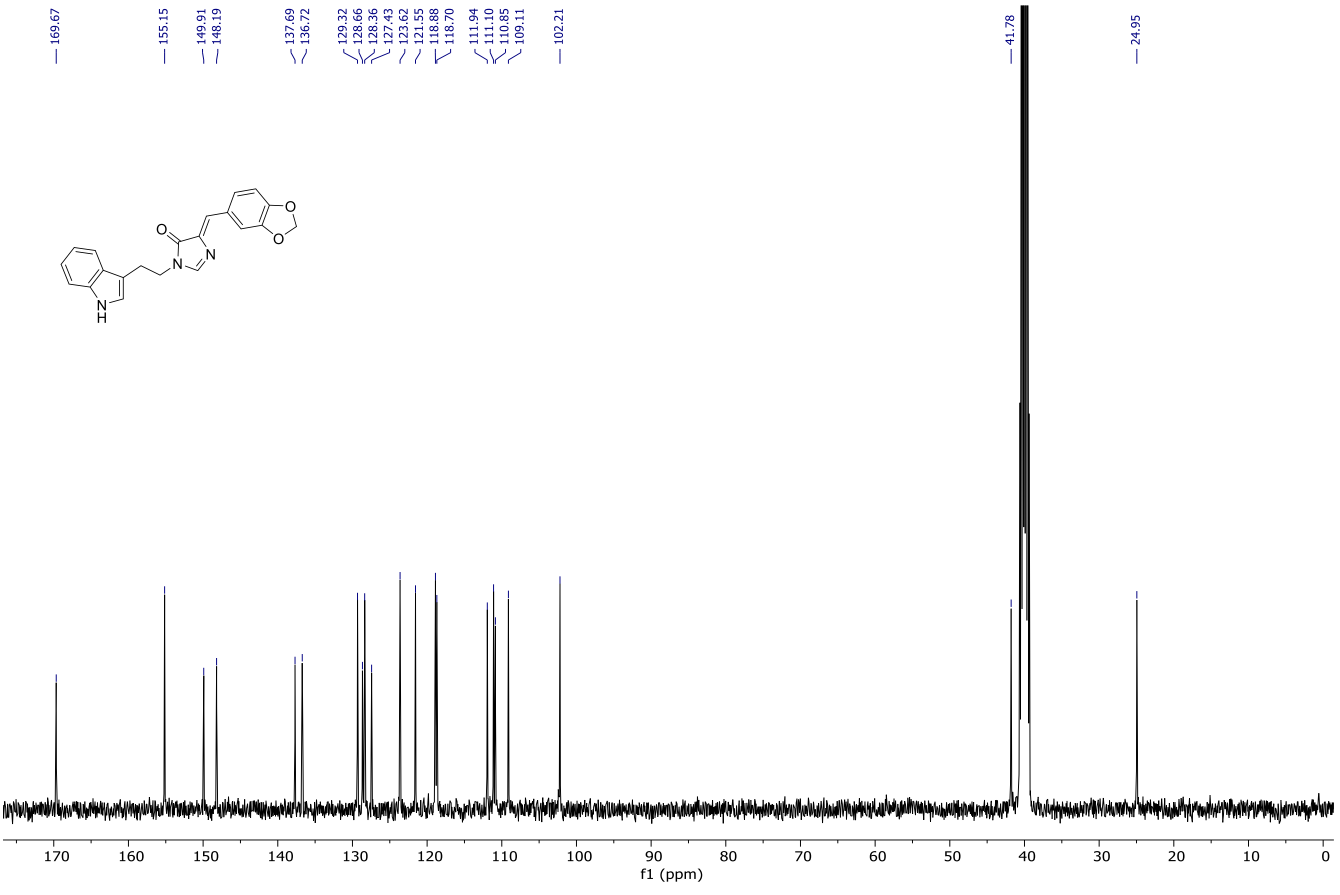

(*Z*)-5-(Benzo[d][1,3]dioxol-5-ylmethylene)-3-((*E*)-4-phenylbut-3-en-1-yl)-3,5-dihydro-4*H*-imidazol-4-one (5v)

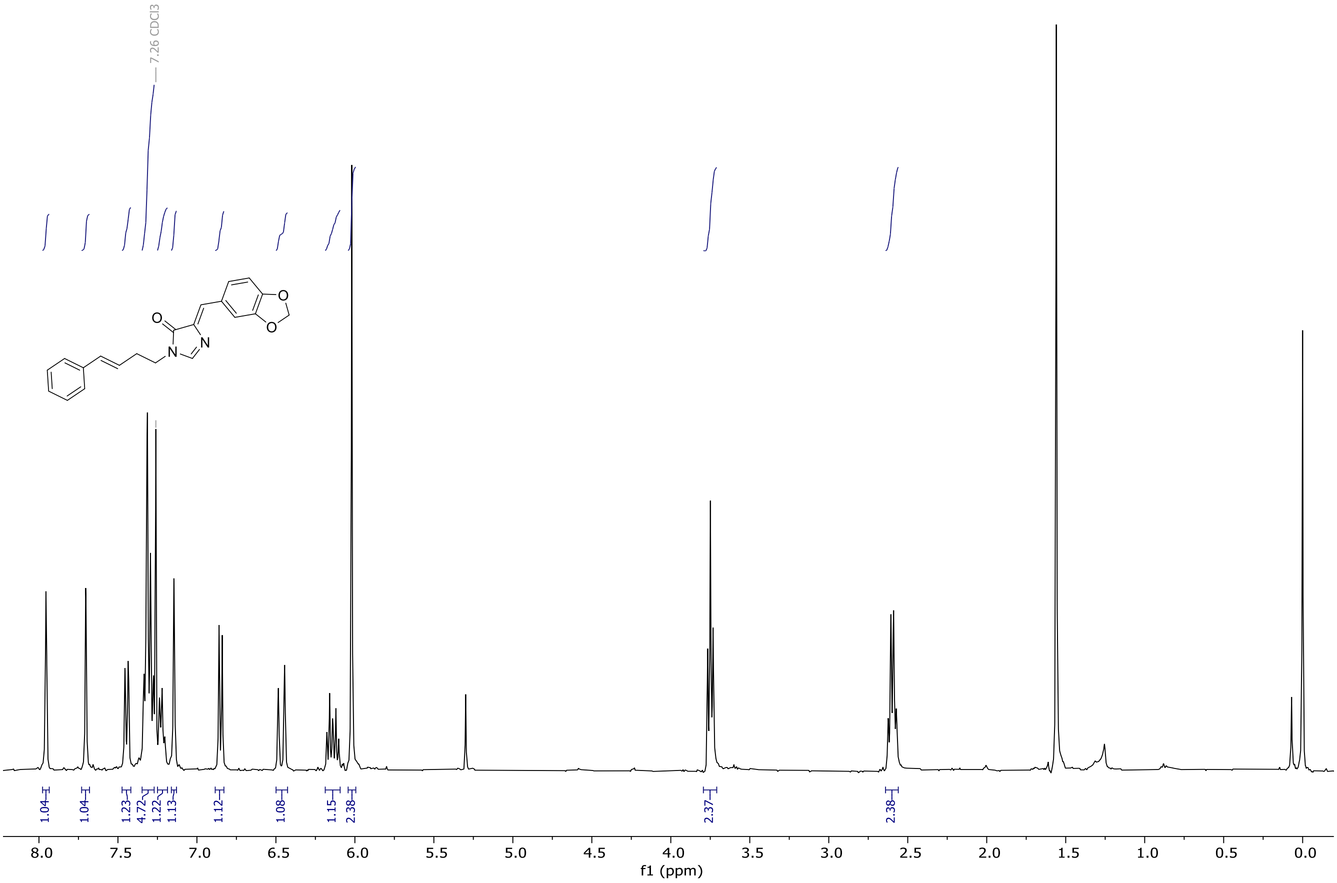

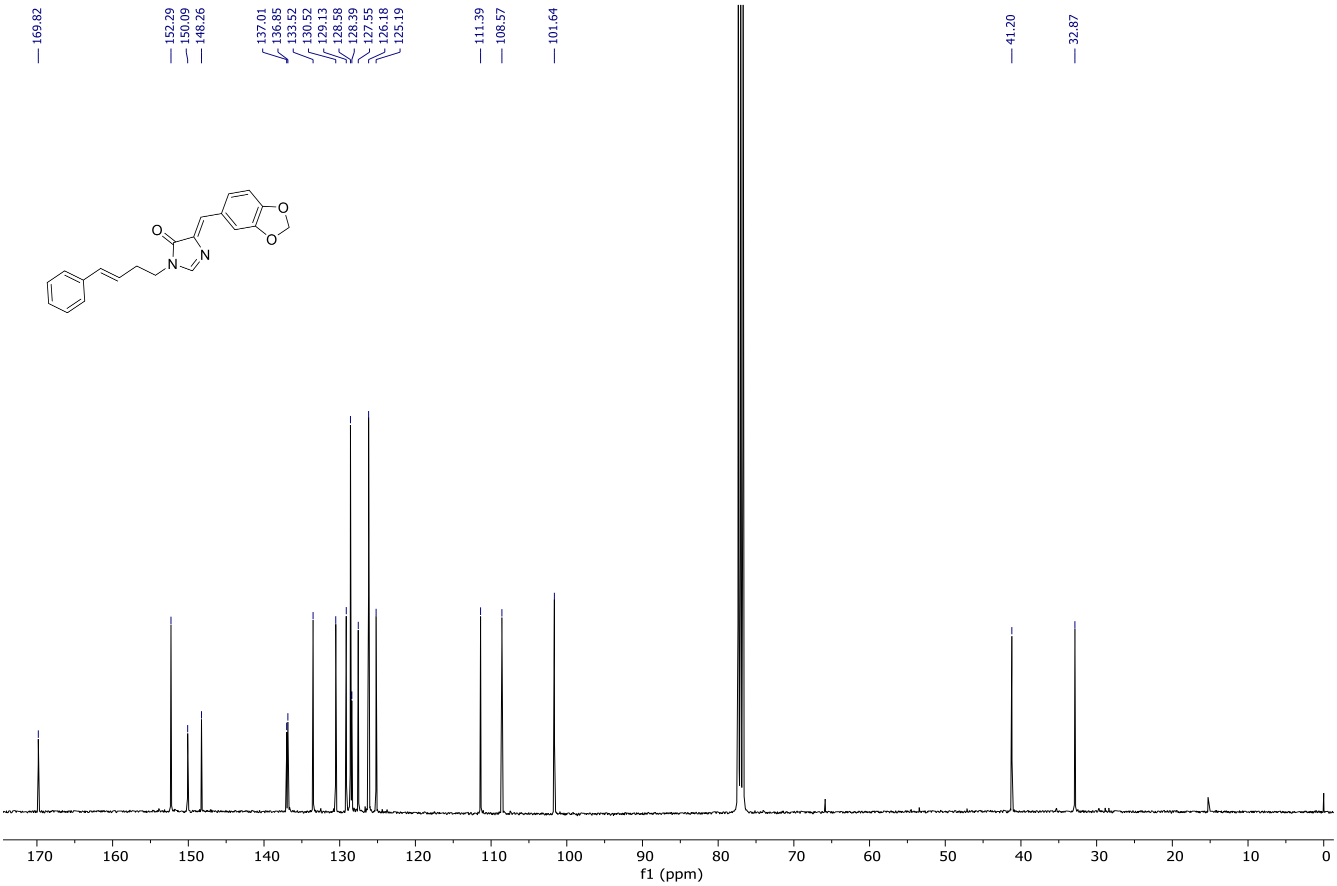

(*Z*)-5-(benzo[d][1,3]dioxol-5-ylmethylene)-3,5-dihydro-4*H*-imidazol-4-one (5w)

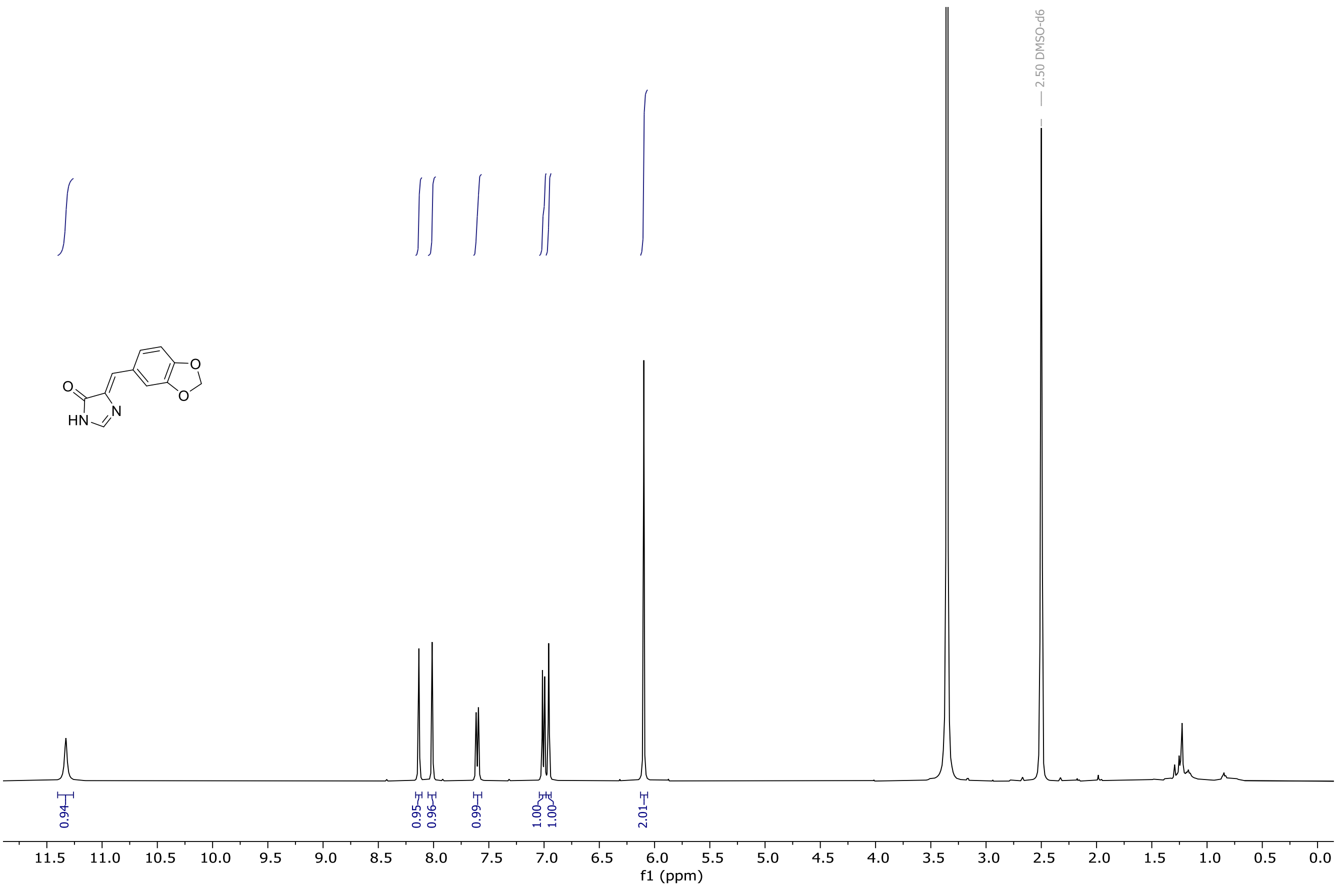

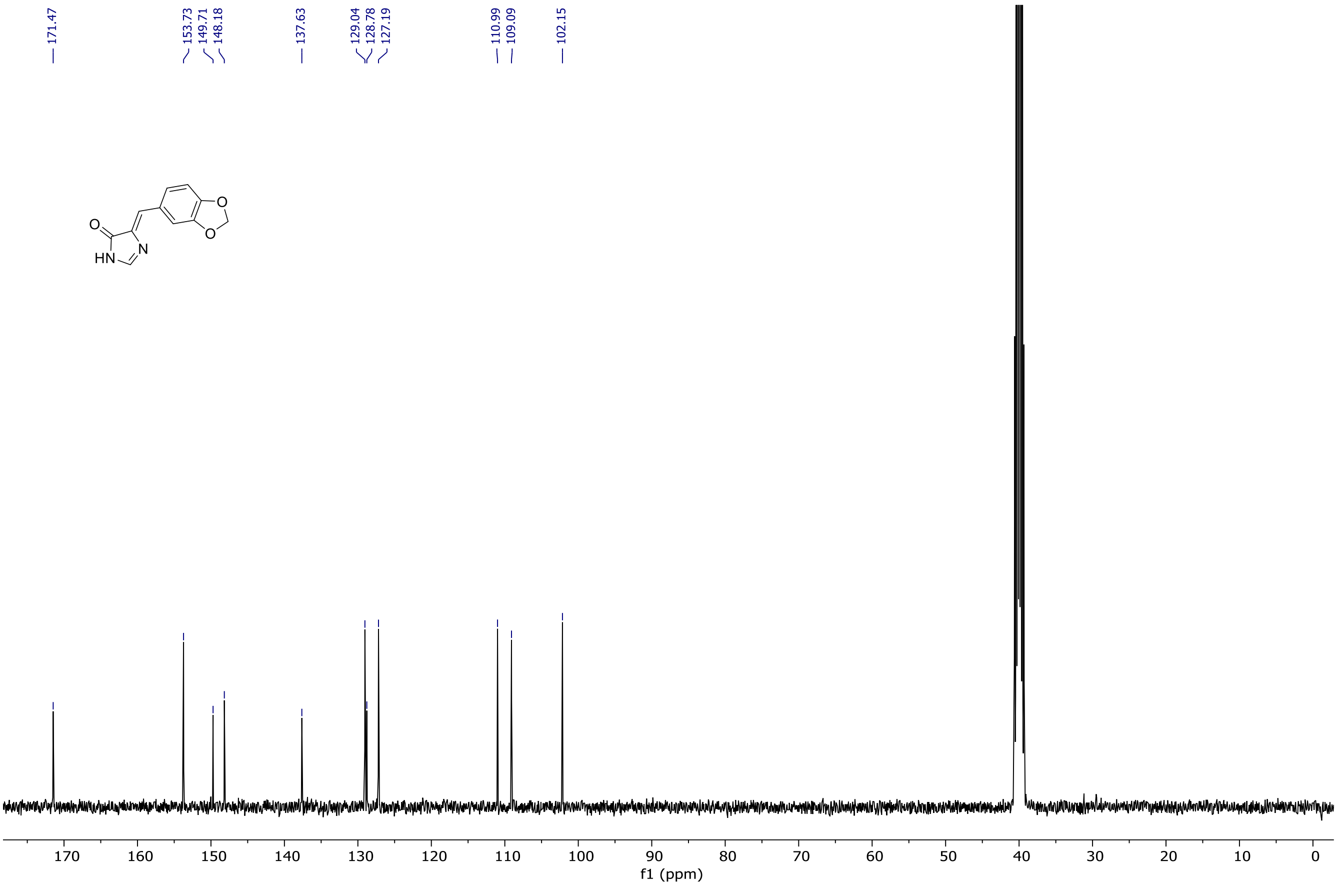

Allyl (*S,Z*)-2-((((9*H*-fluoren-9-yl)methoxy)carbonyl)amino)-6-(4-(benzo[*d*][1,3]dioxol-5-ylmethylene)-5-oxo-4,5-dihydro-1*H*-imidazol-1-yl)hexanoate (5x)

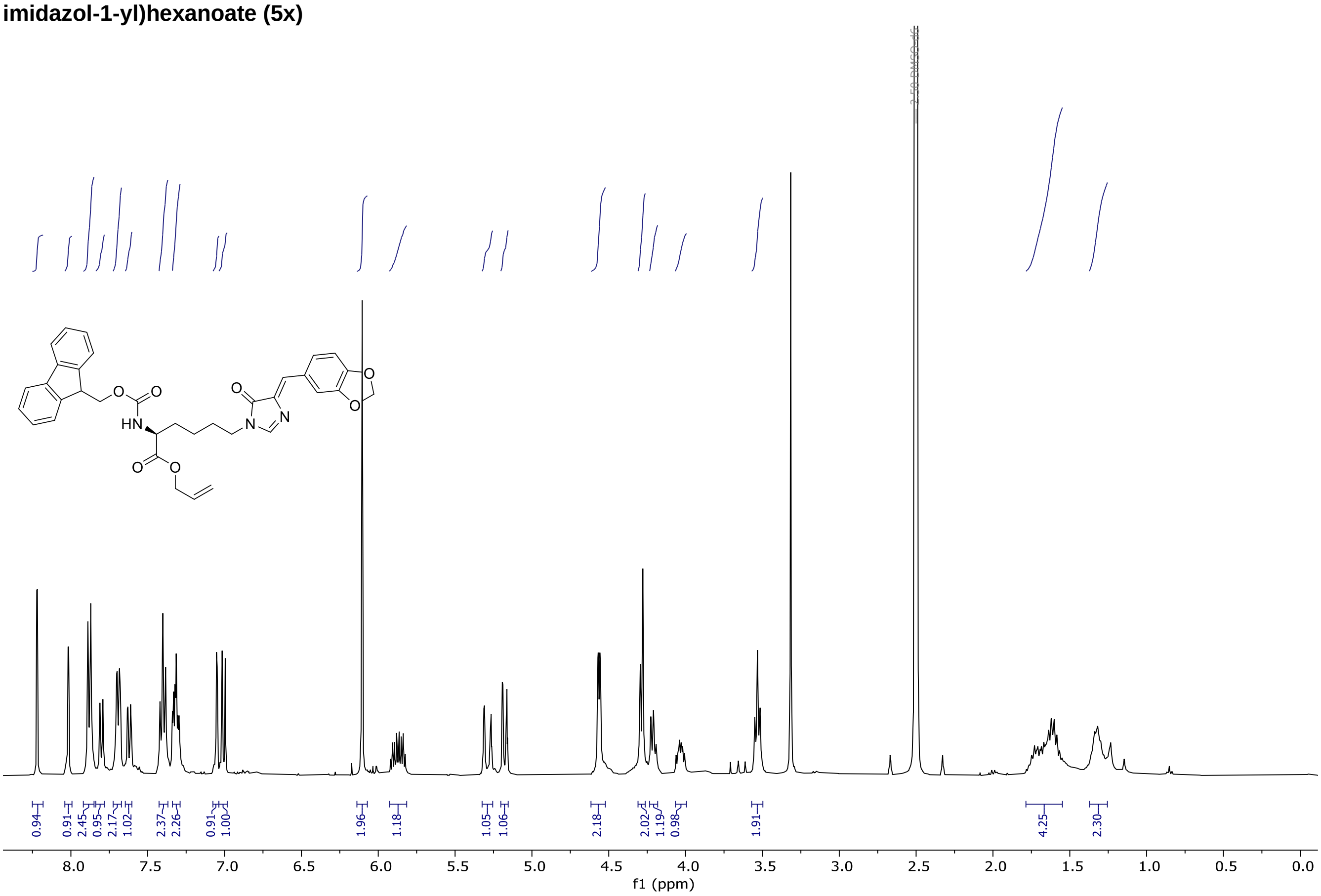

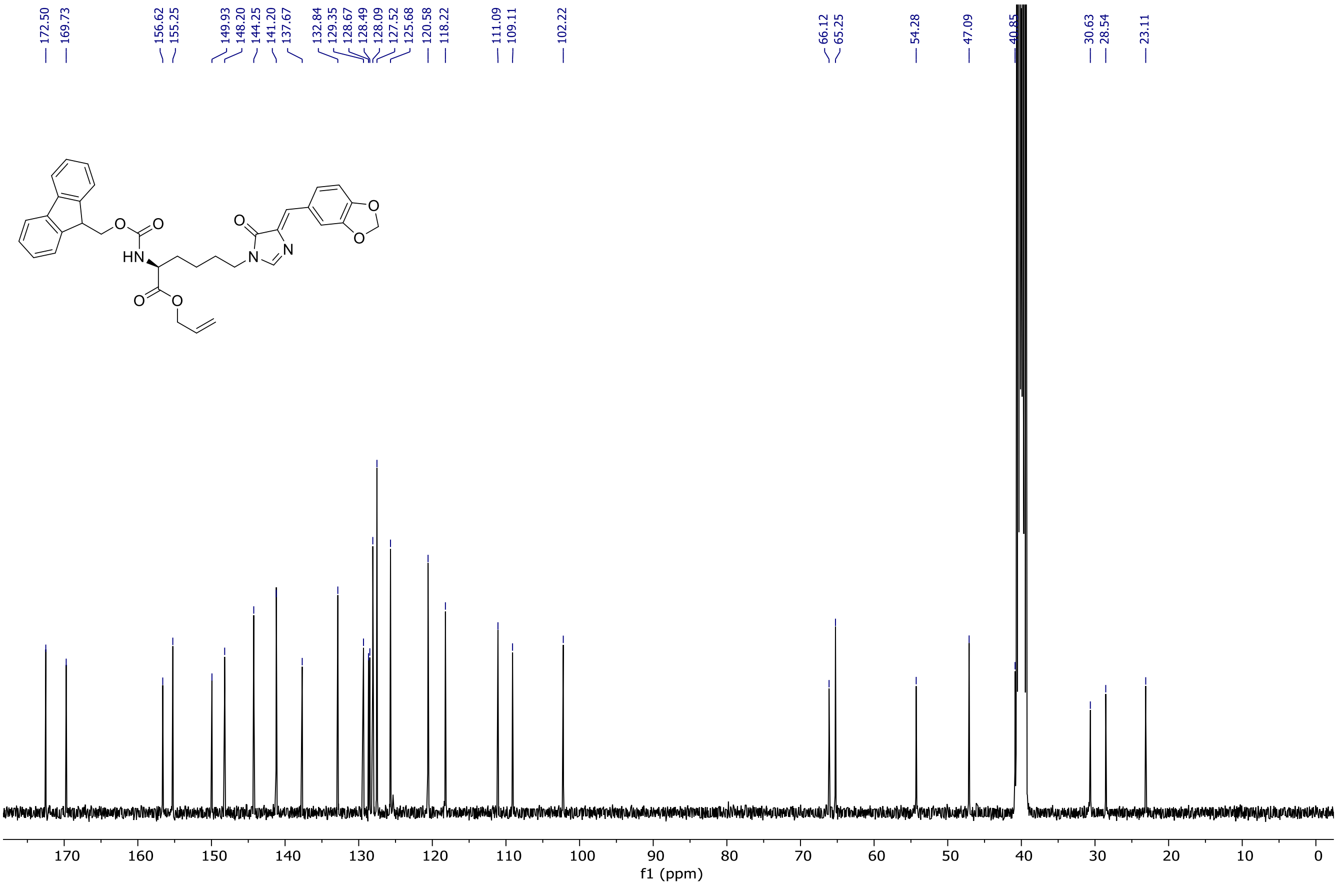

(*S,Z*)-2-((((9*H*-Fluoren-9-yl)methoxy)carbonyl)amino)-6-(4-(benzo[*d*][1,3]dioxol-5-ylmethylene)-5-oxo-4,5-dihydro-1*H*-imidazol-1-yl)hexanoic acid (5x')

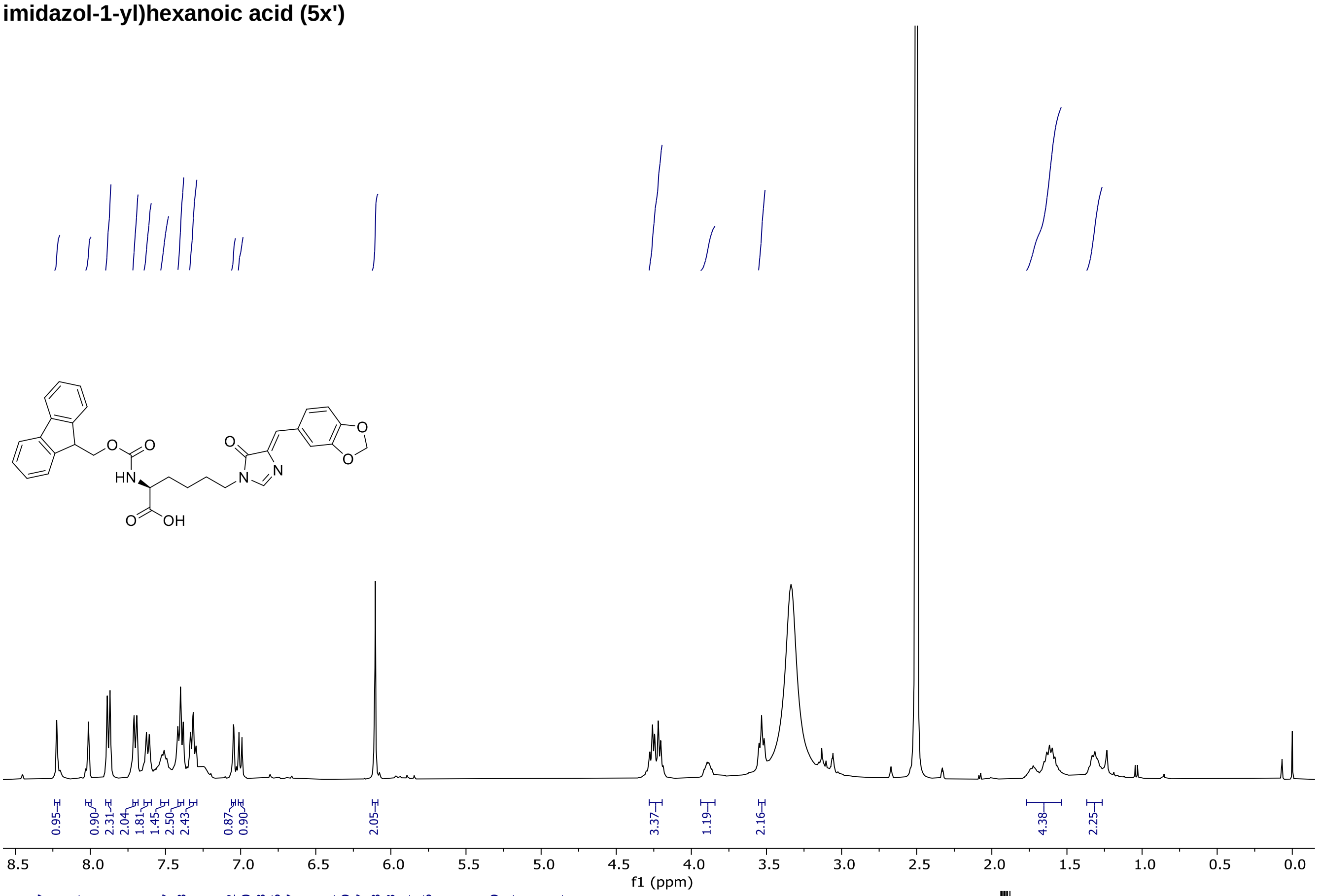

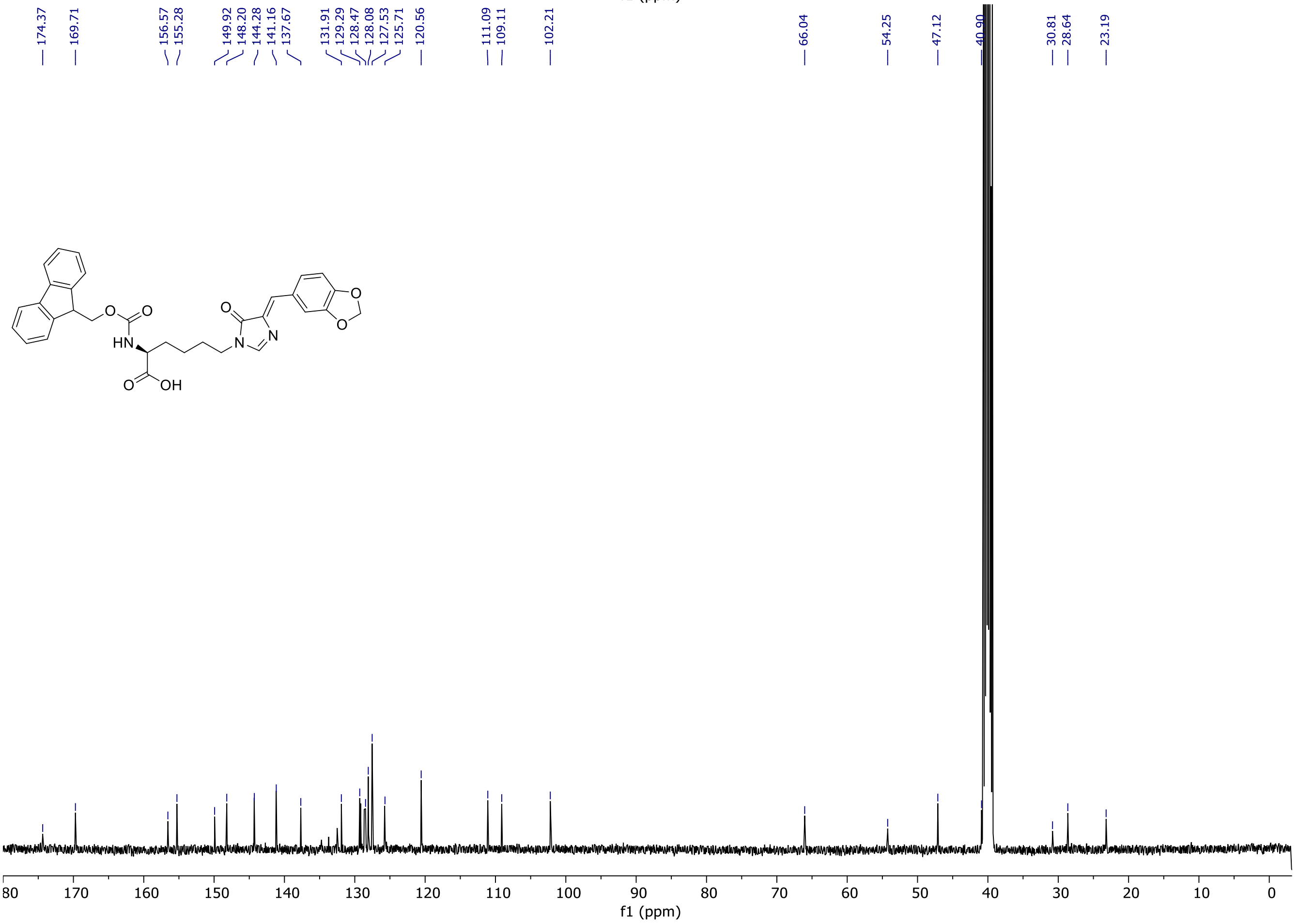

(*Z*)-3-(4-Methoxyphenyl)-5-(thiophen-2-ylmethylene)-3,5-dihydro-4*H*-imidazol-4-one (5y)

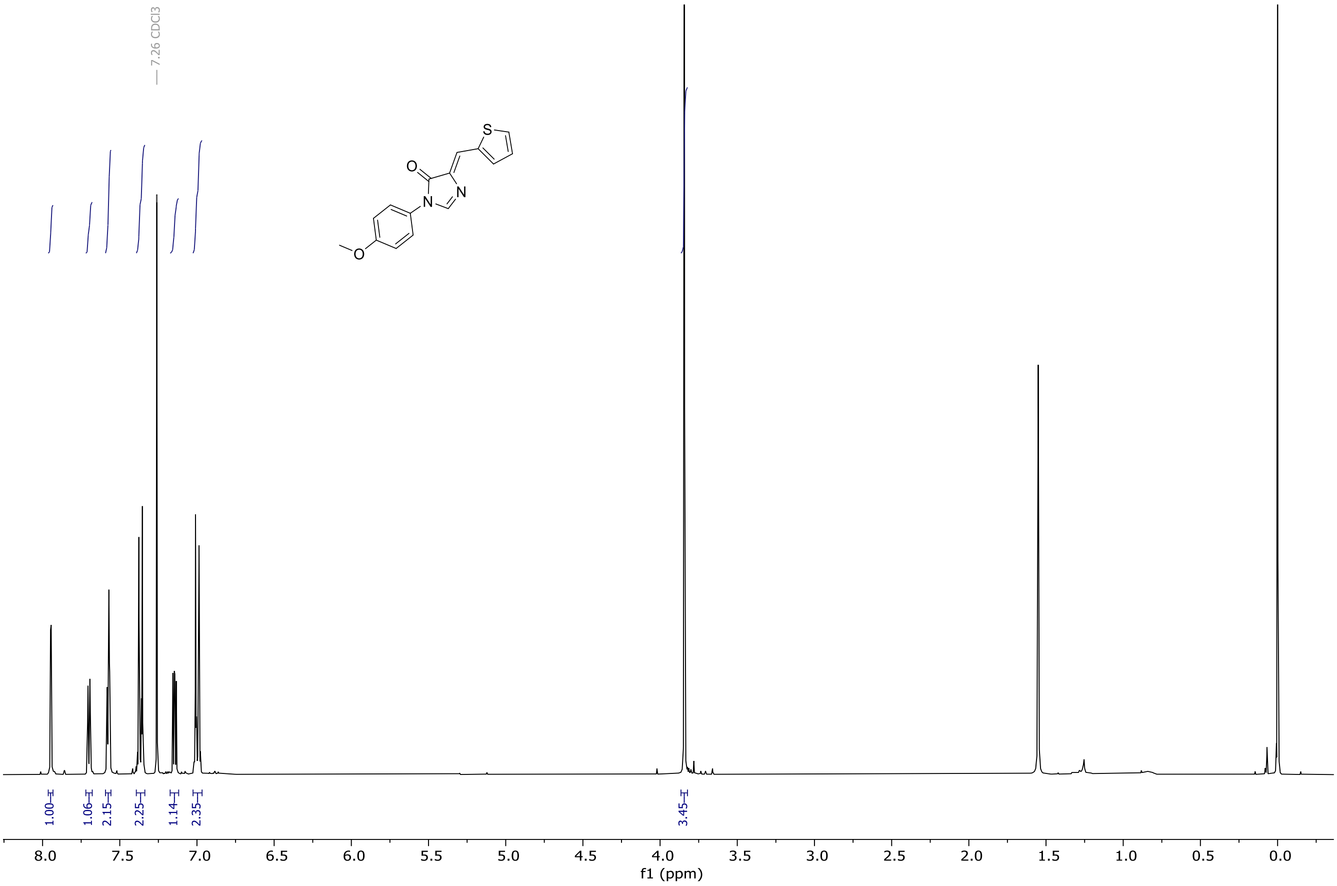

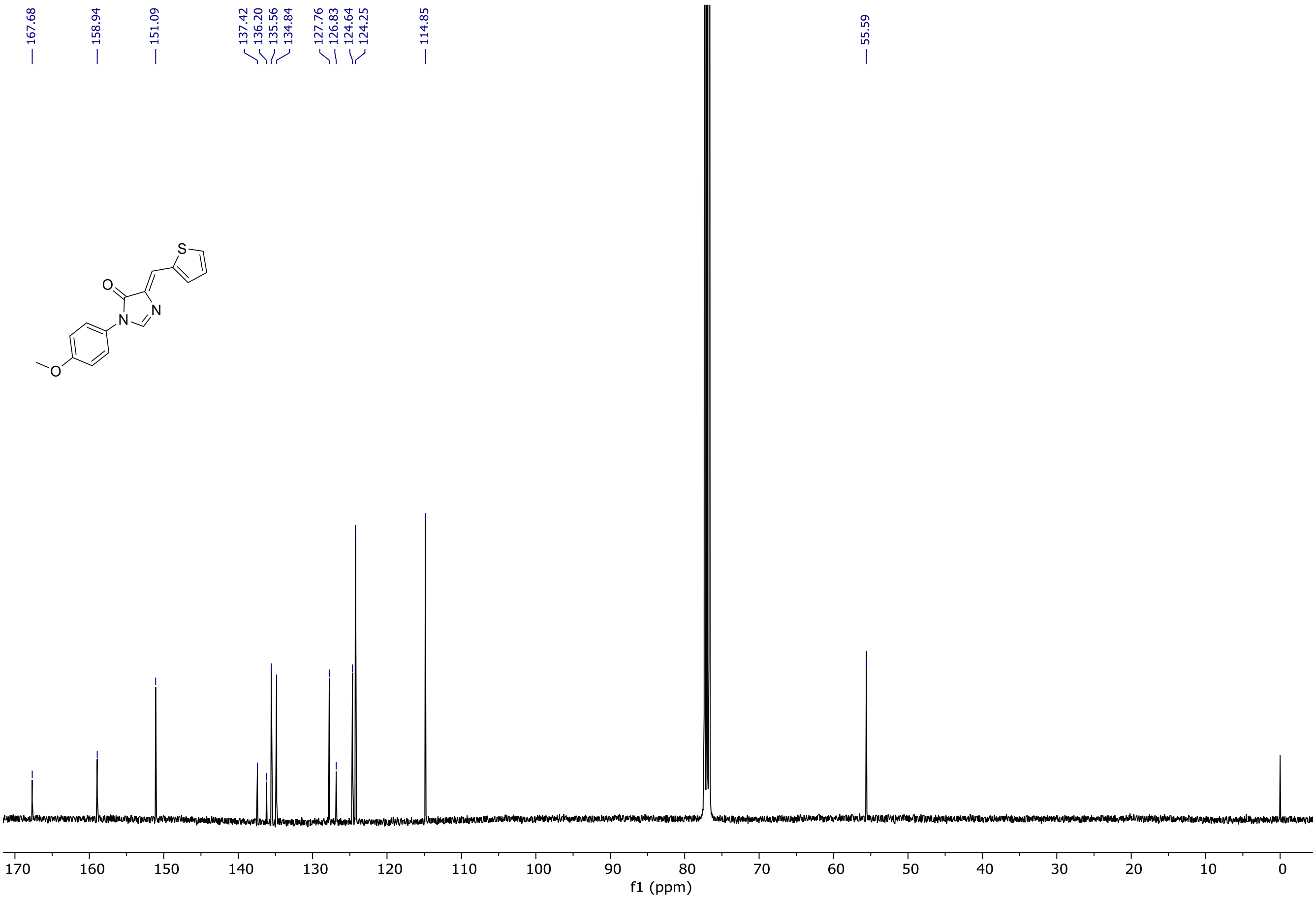

(*Z*)-5-(Benzo[d][1,3]dioxol-5-ylmethylene)-3-(tert-butyl)-3,5-dihydro-4*H*-imidazol-4-one (5z)

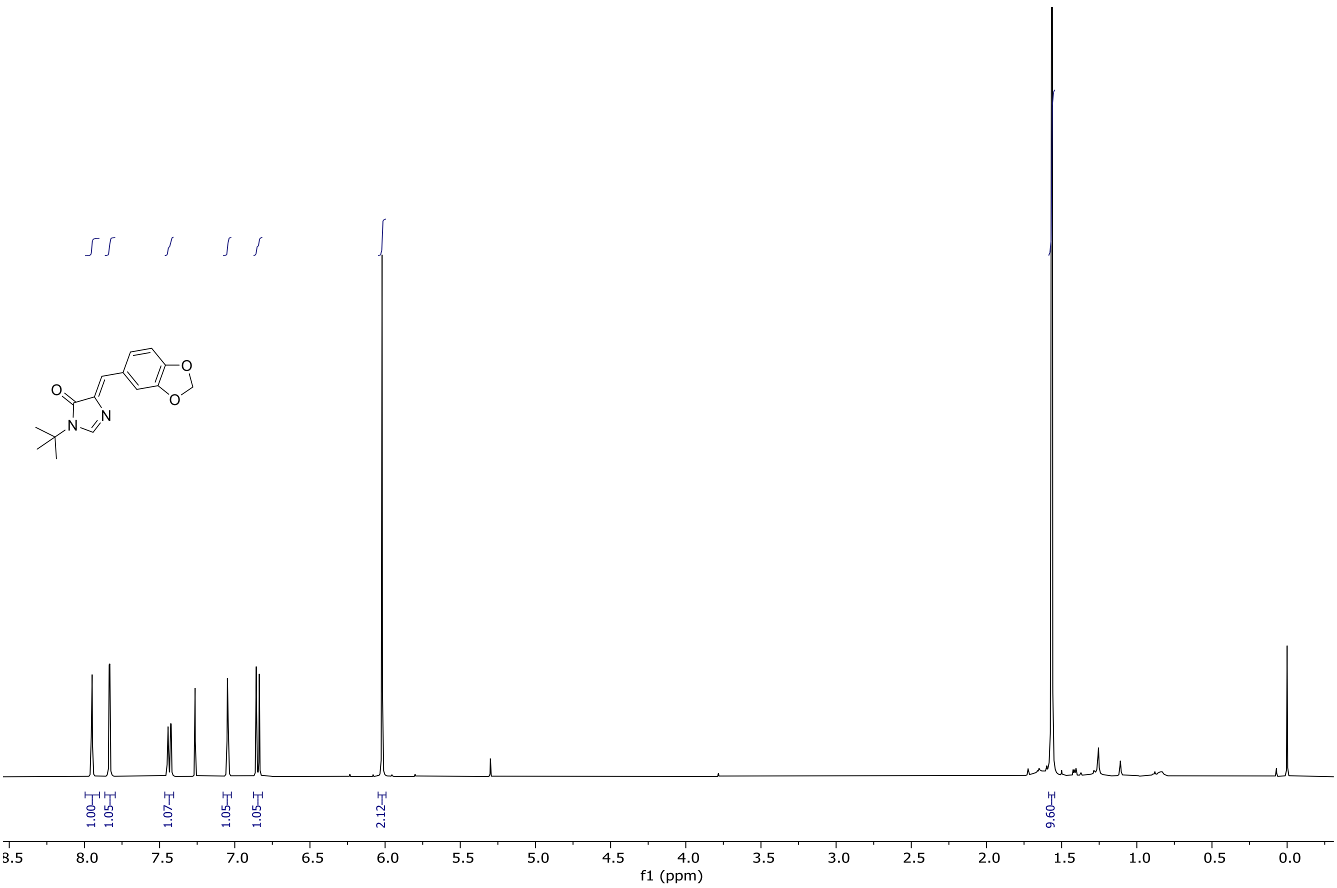

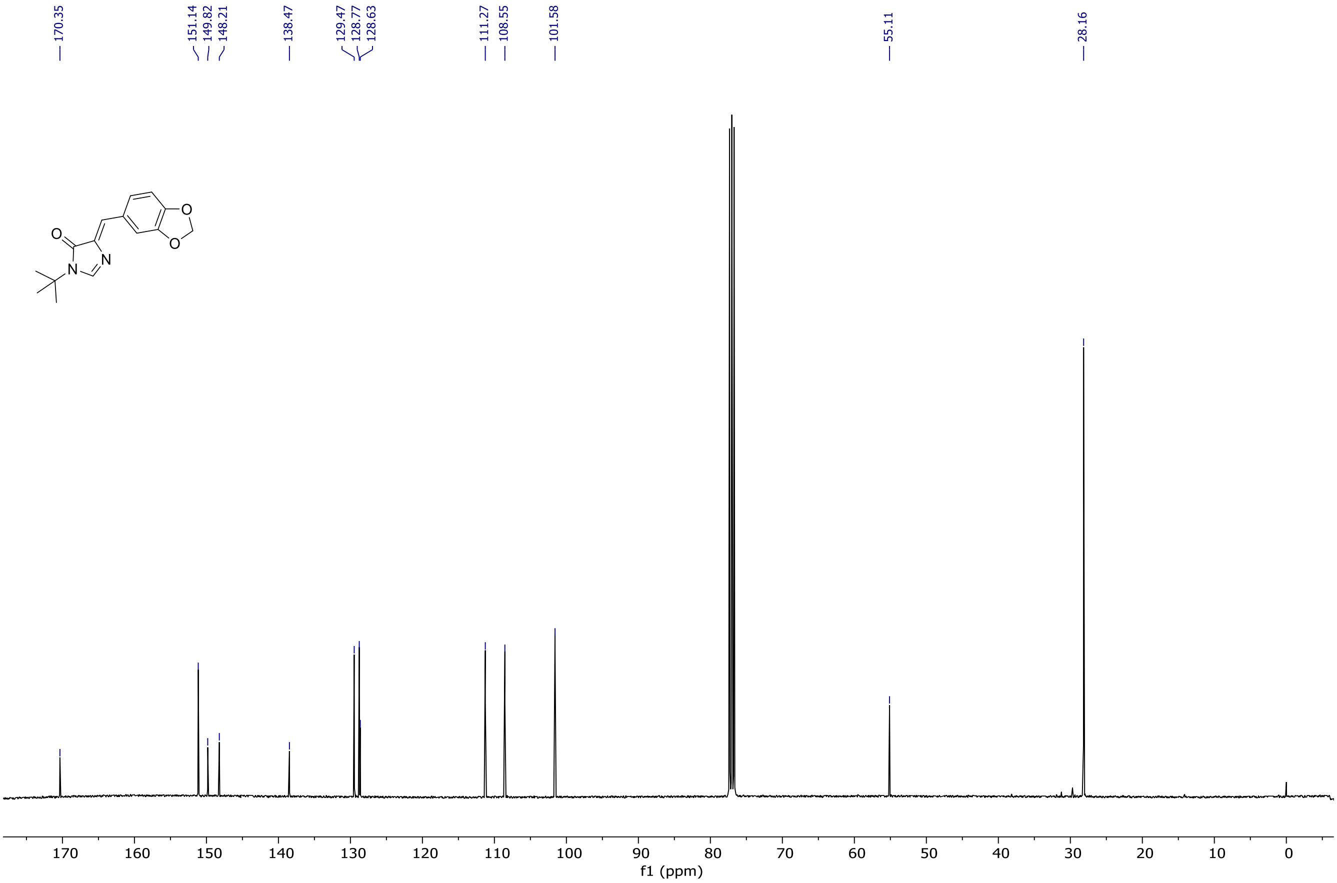

3-Benzyl-5-(propan-2-ylidene)-3,5-dihydro-4*H*-imidazol-4-one (5aa)

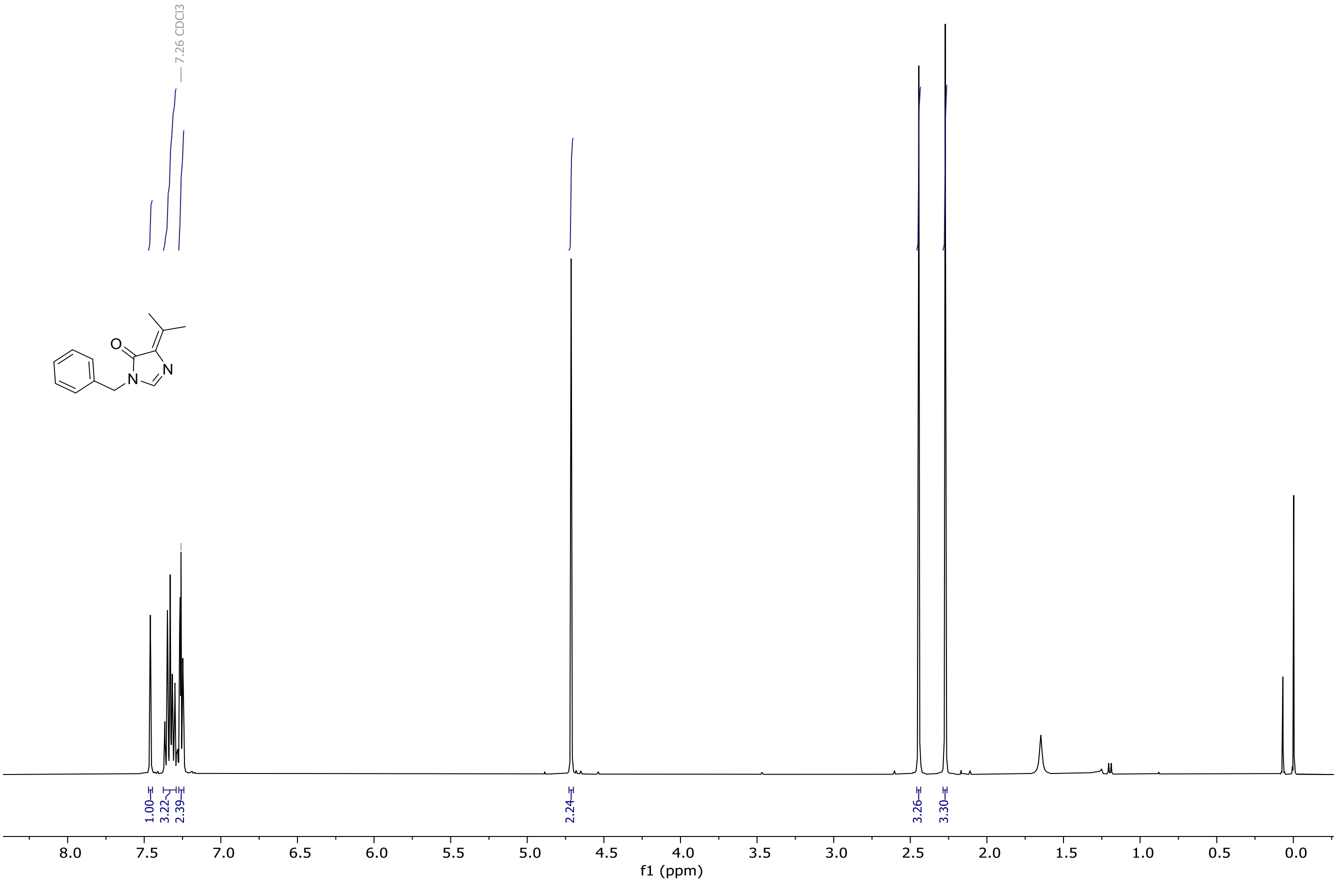

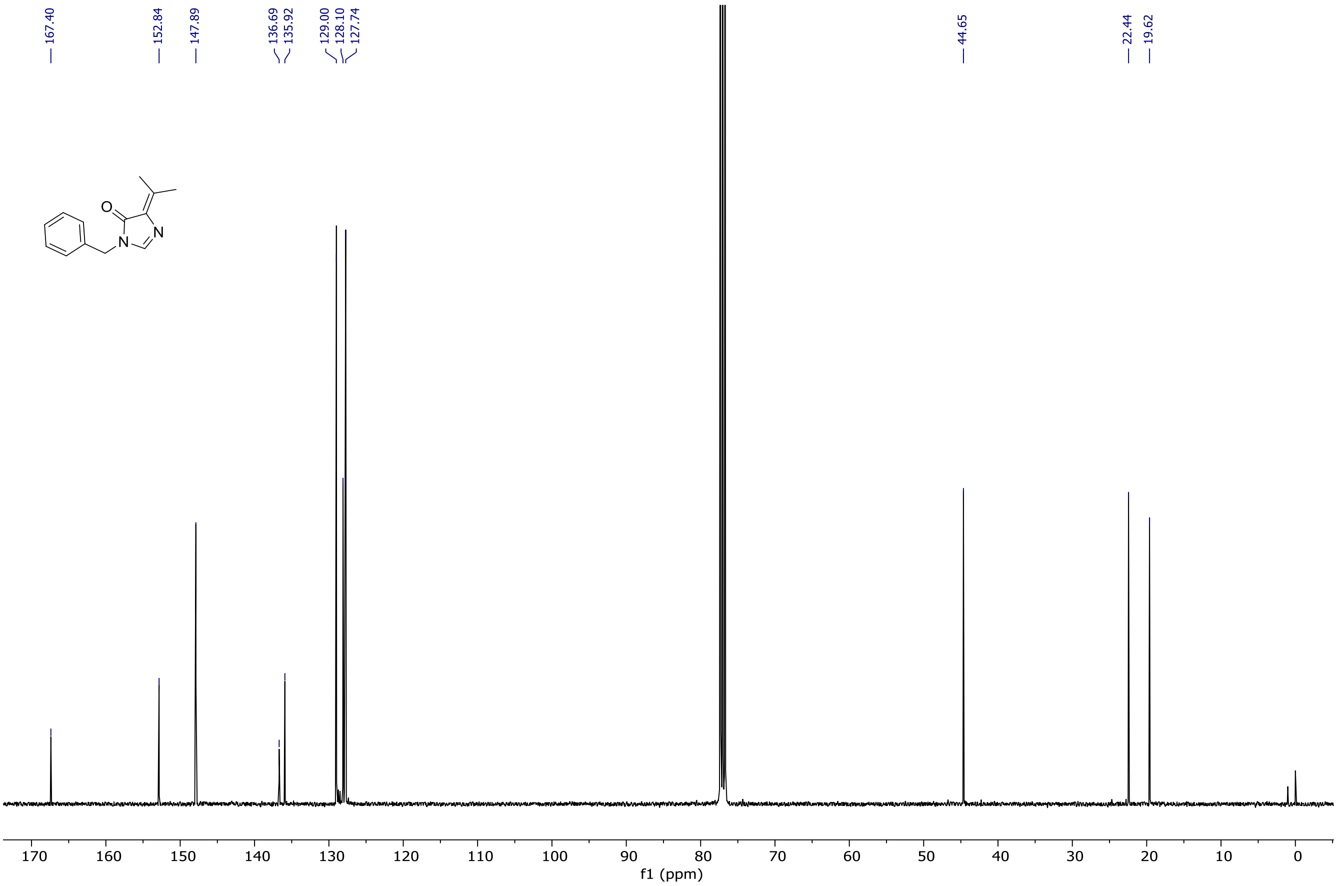

3-Benzyl-5-cyclopentylidene-3,5-dihydro-4*H*-imidazol-4-one (5ab)

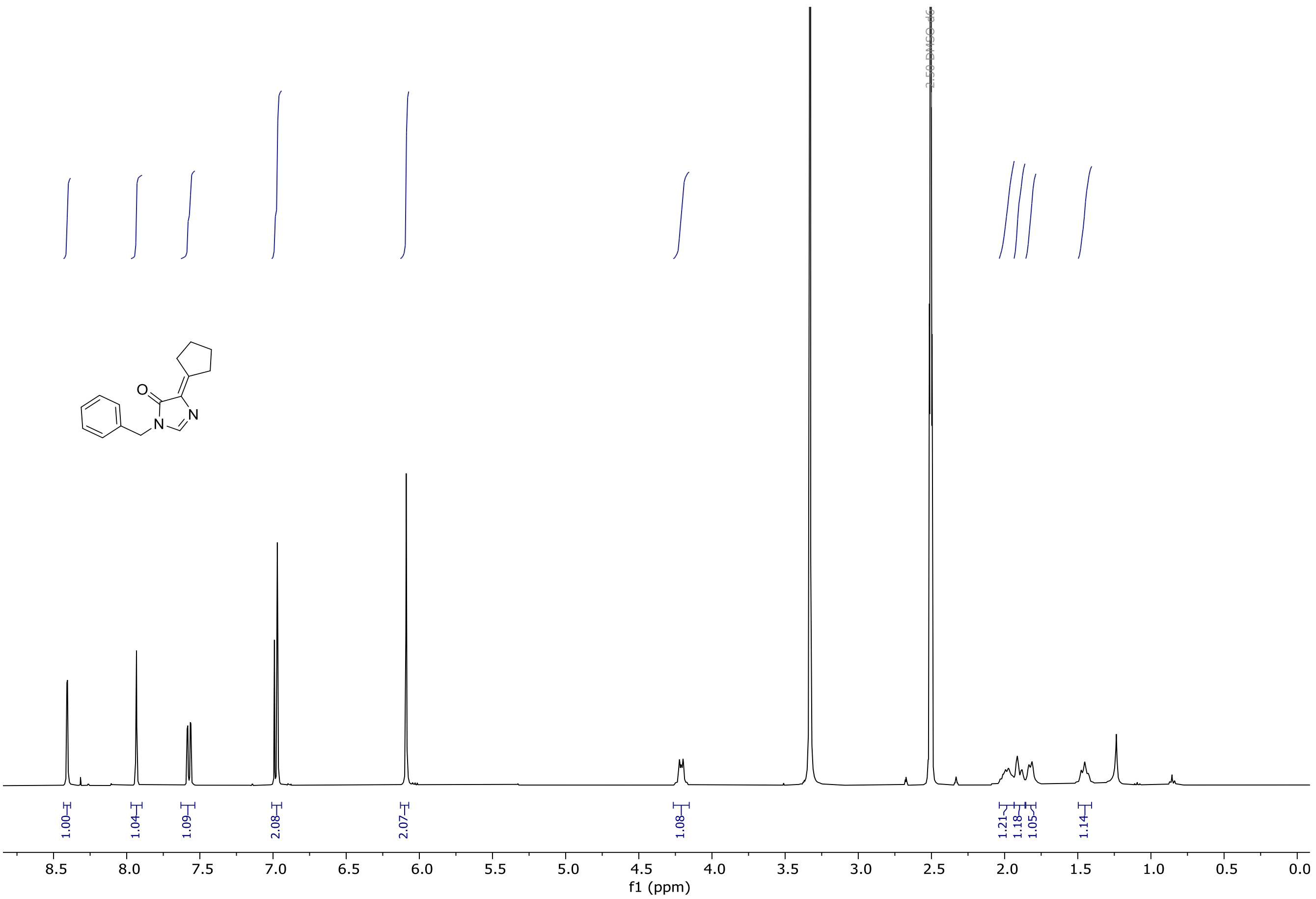

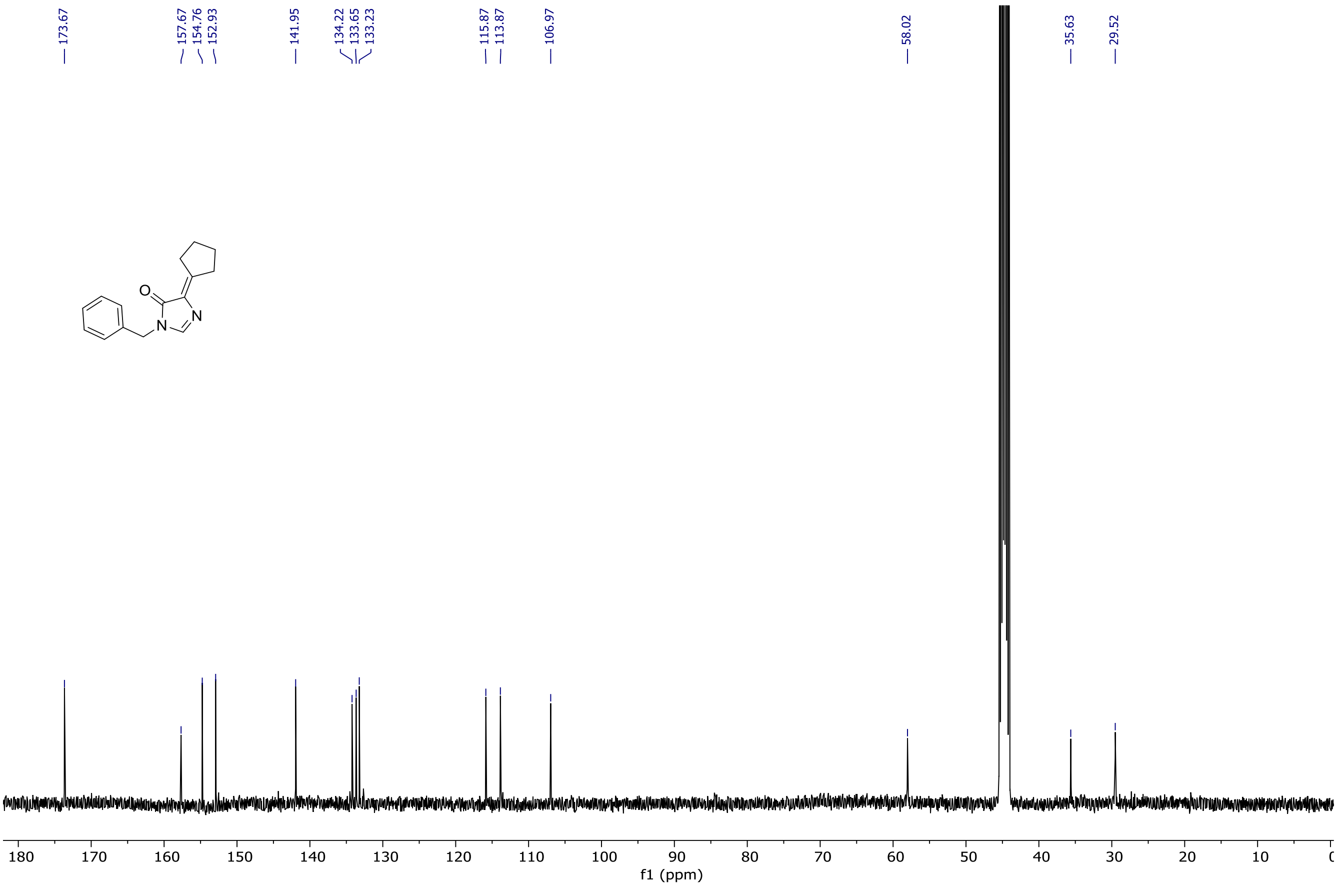

5-((1*r*,3*r*)-adamantan-2-ylidene)-3-benzyl-3,5-dihydro-4*H*-imidazol-4-one (5ac)

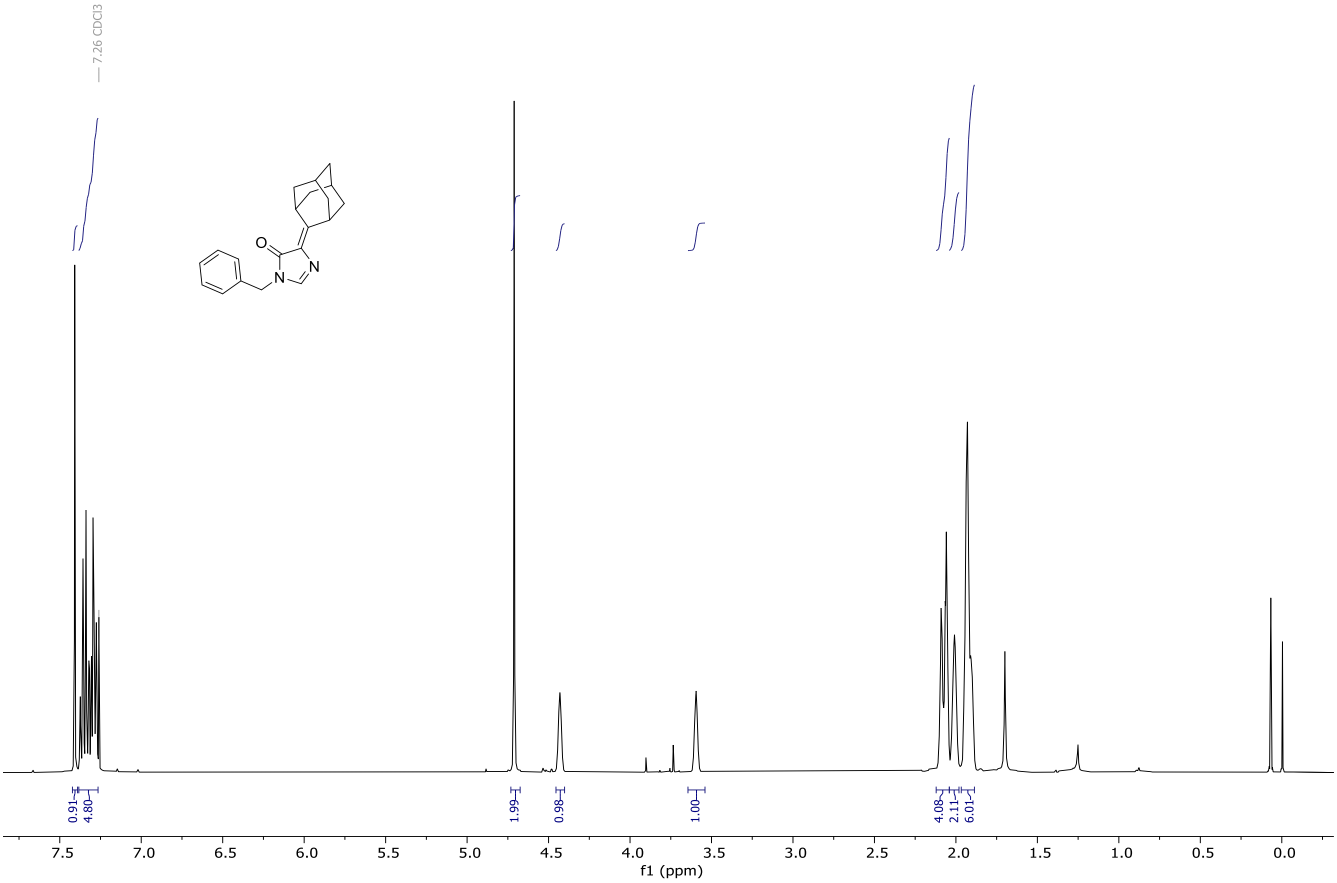

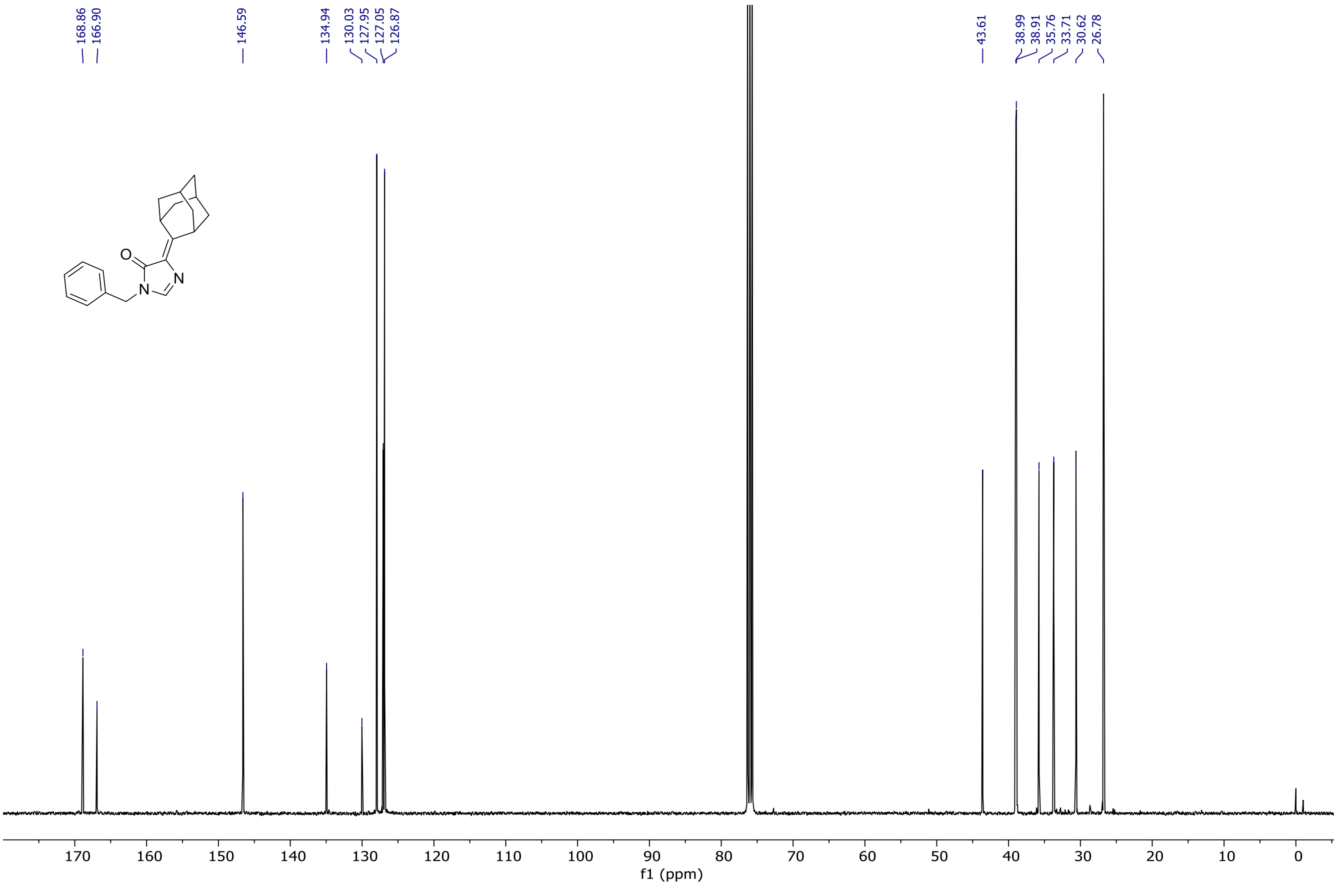

(5Z,5'Z)-3,3'-(Cyclohexane-1,2-diyl)bis(5-(benzo[d][1,3]dioxol-5-ylmethylene)-3,5-dihydro-4H-imidazol-4-one) (5-bis-a)

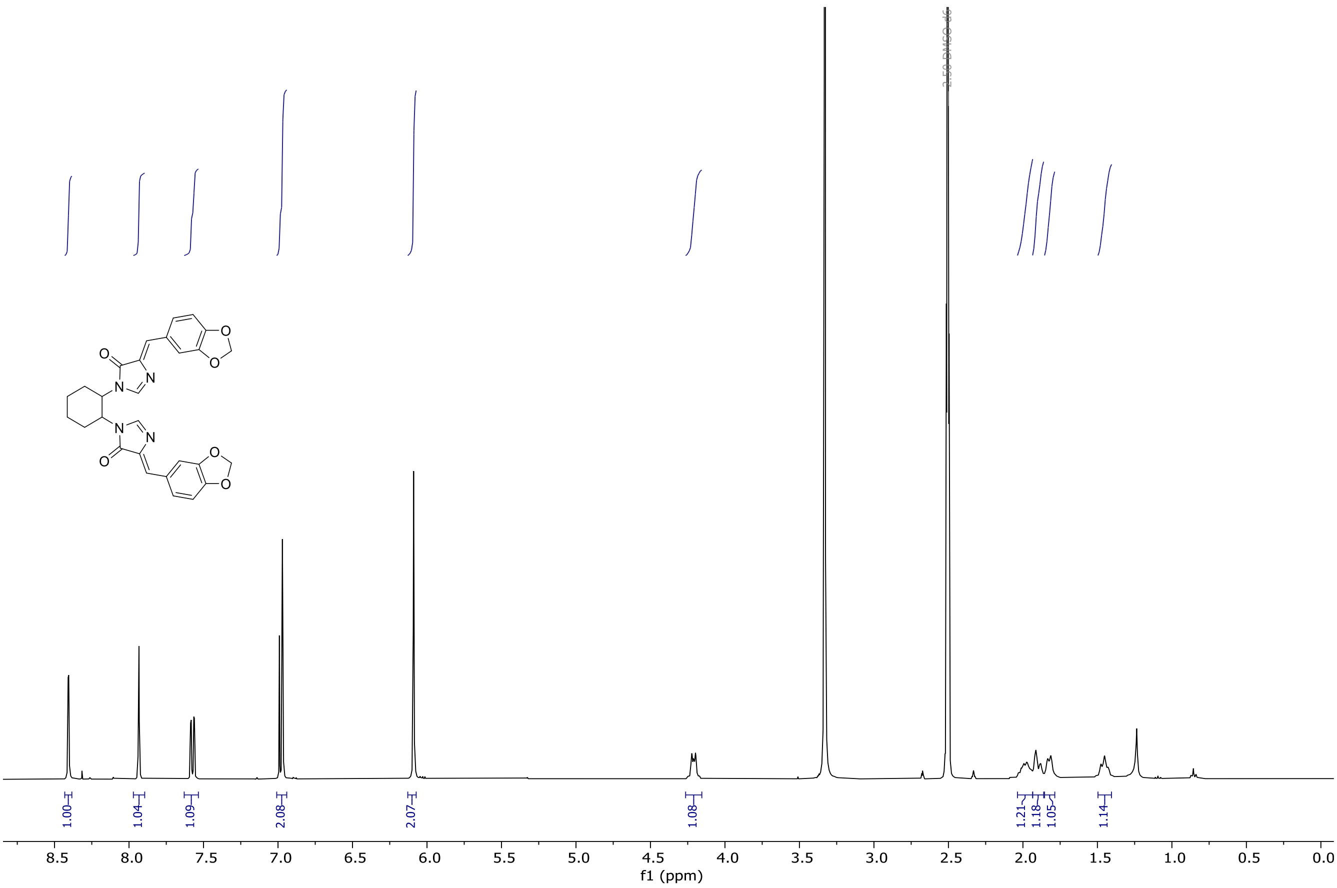

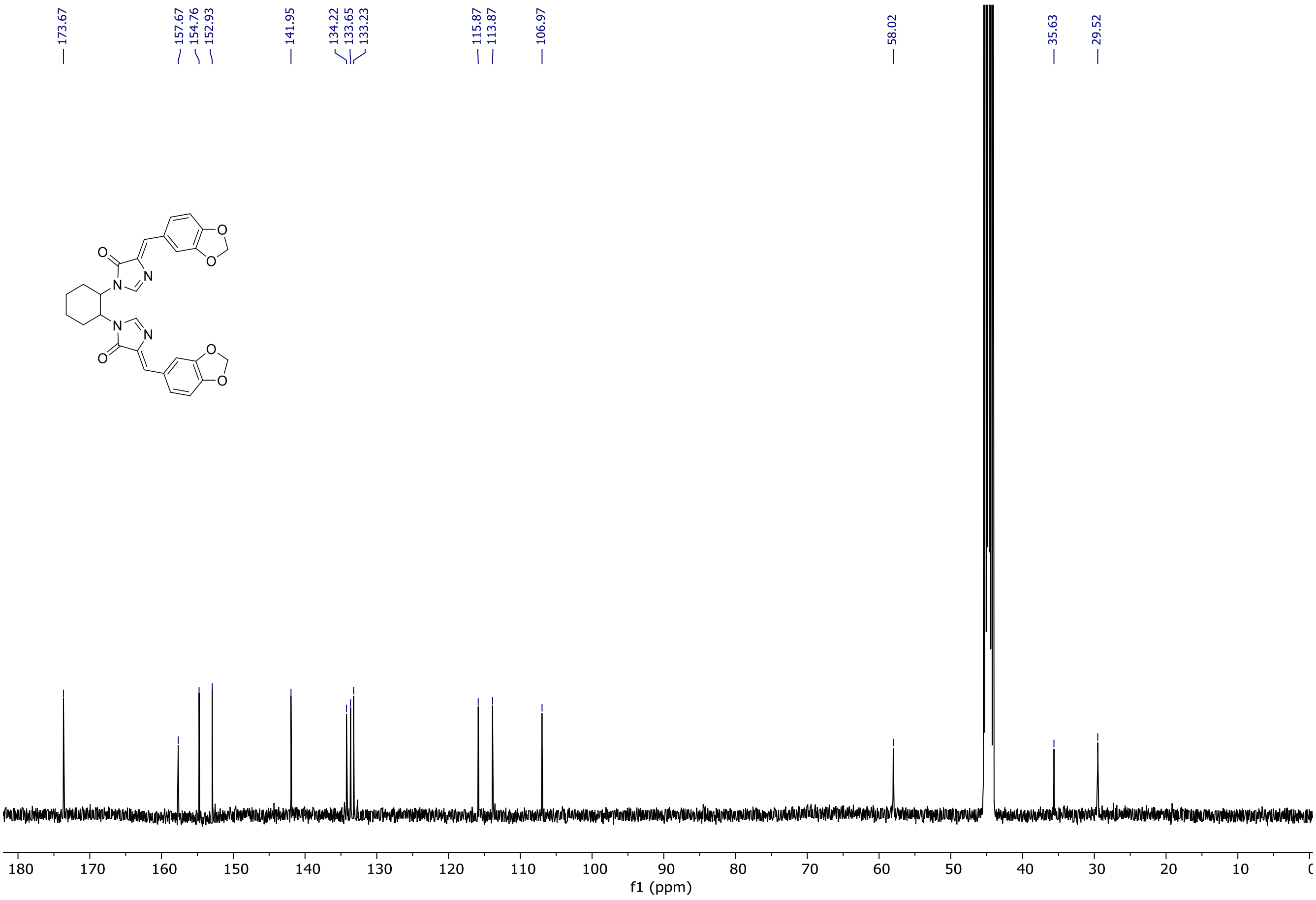

8.4. Compounds 6-8

Methyl 5-(pyridin-4-yl)-4,5-dihydrooxazole-4-carboxylate (6a)

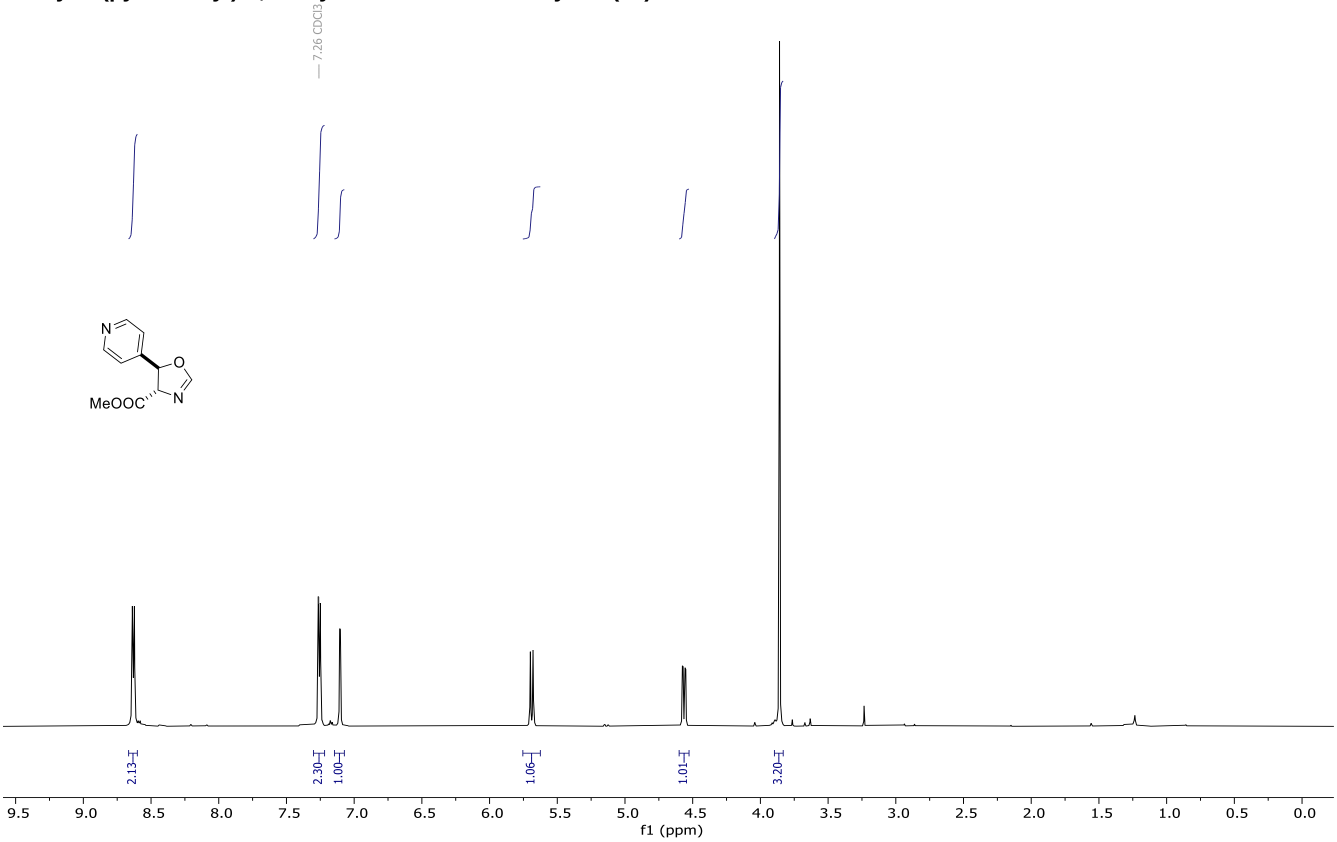

Ethyl 5,5-dimethyl-4,5-dihydrooxazole-4-carboxylate (6b)

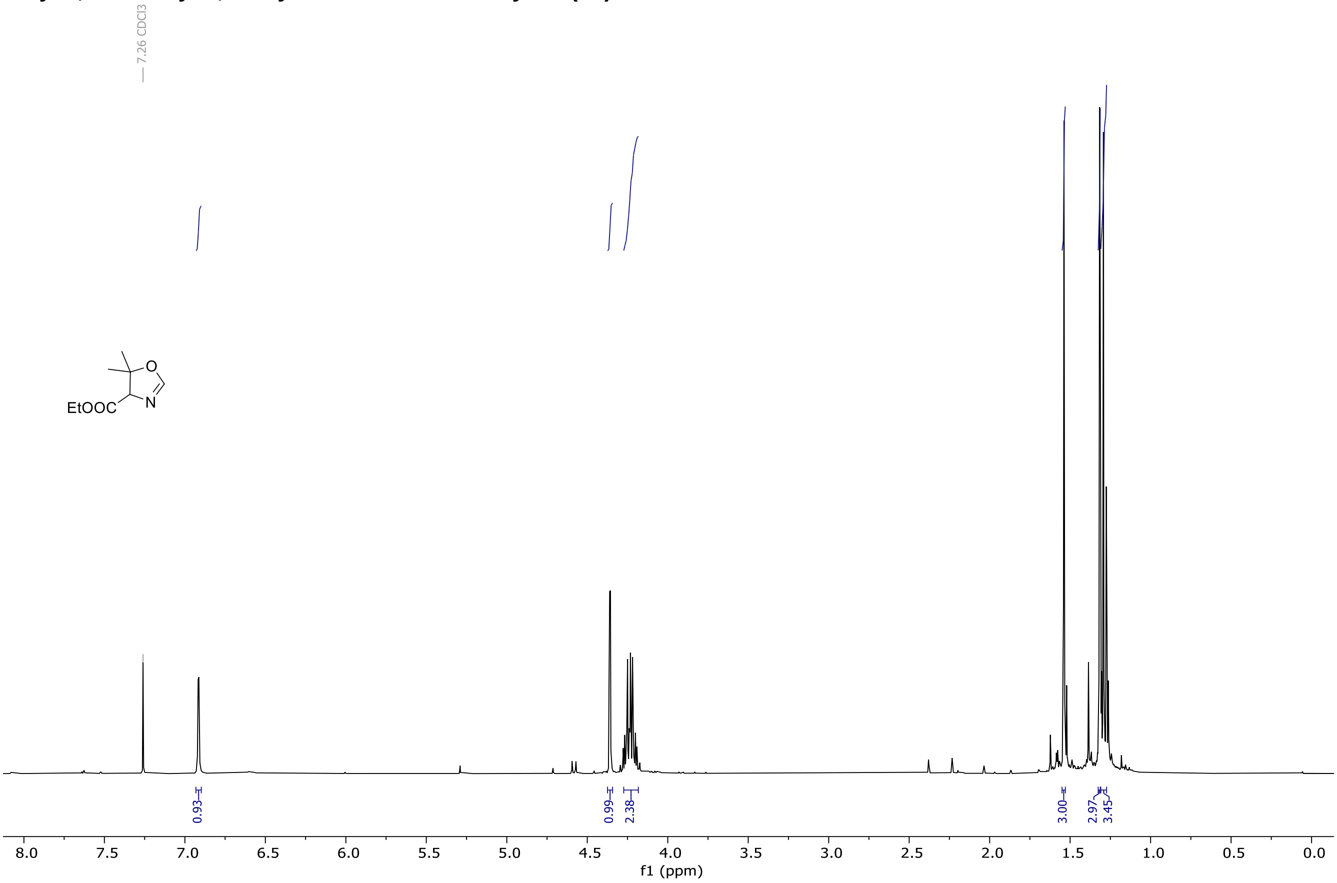

Methyl pyrimido[1,6-*a*]indole-3-carboxylate (7)

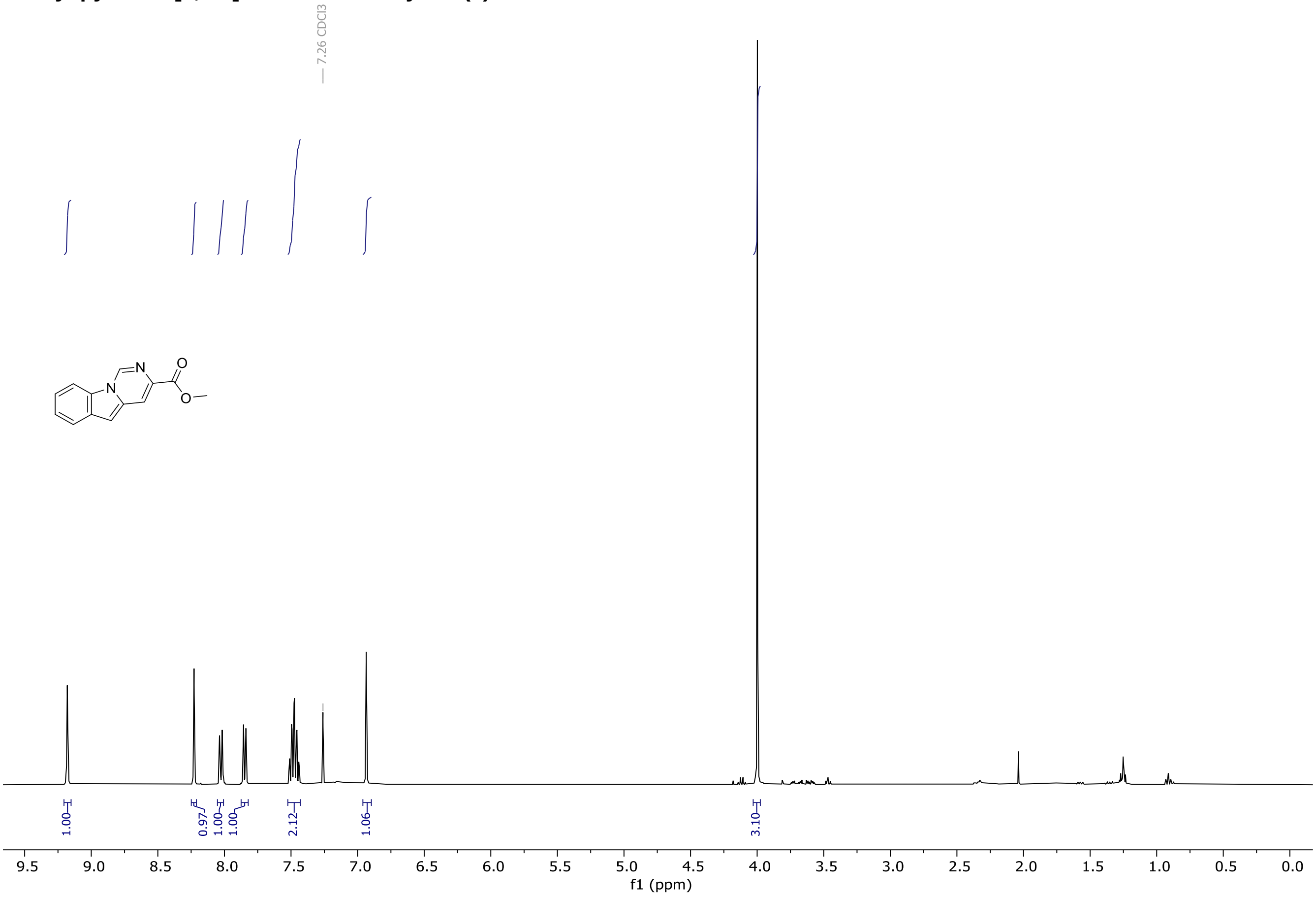

(5*R*,6*R*,7*S*,12*S*)-3,10-dibenzyl-6,12-bis(4-chlorophenyl)-1,3,8,10-tetraazadispiro[4.1.4^{7}.1^{5}]dodeca-1,8-diene-4,11-dione (8a)

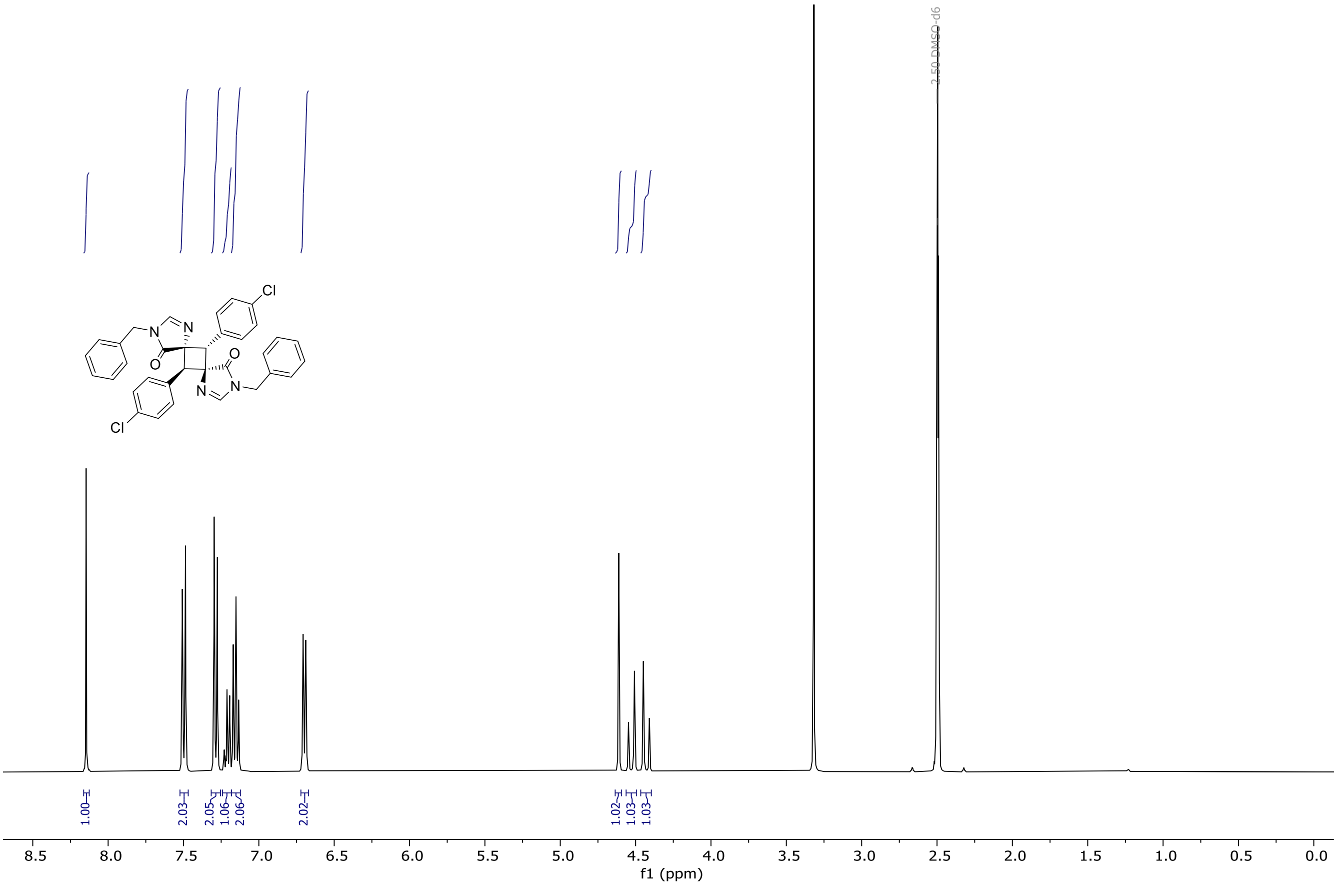

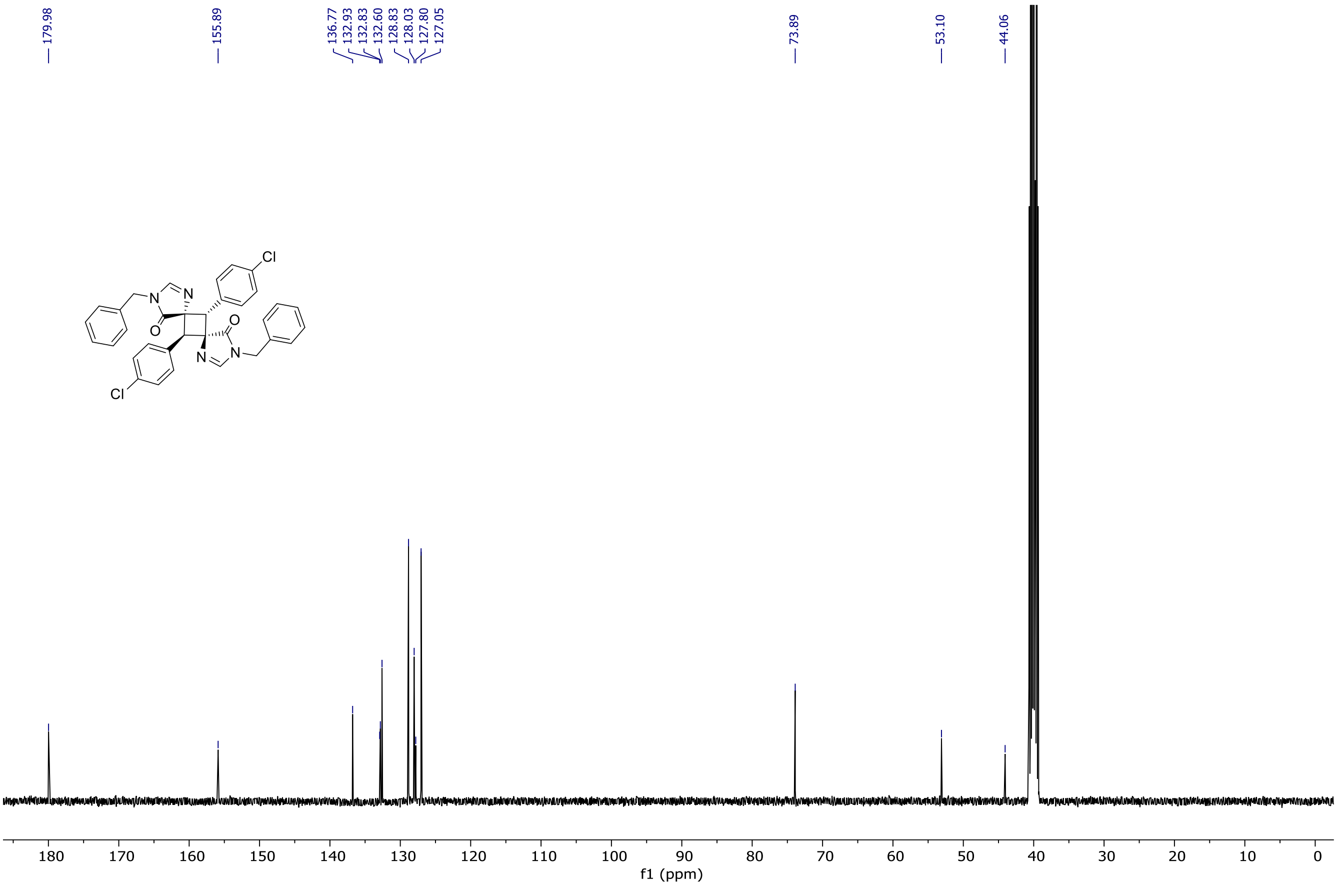

(5*R*,6*R*,7*S*,12*S*)-3,10-Dibenzyl-6,12-bis(4-bromophenyl)-1,3,8,10-tetraazadispiro[4.1.4^7^.1^5^]dodeca-1,8-diene-4,11-dione (8b)

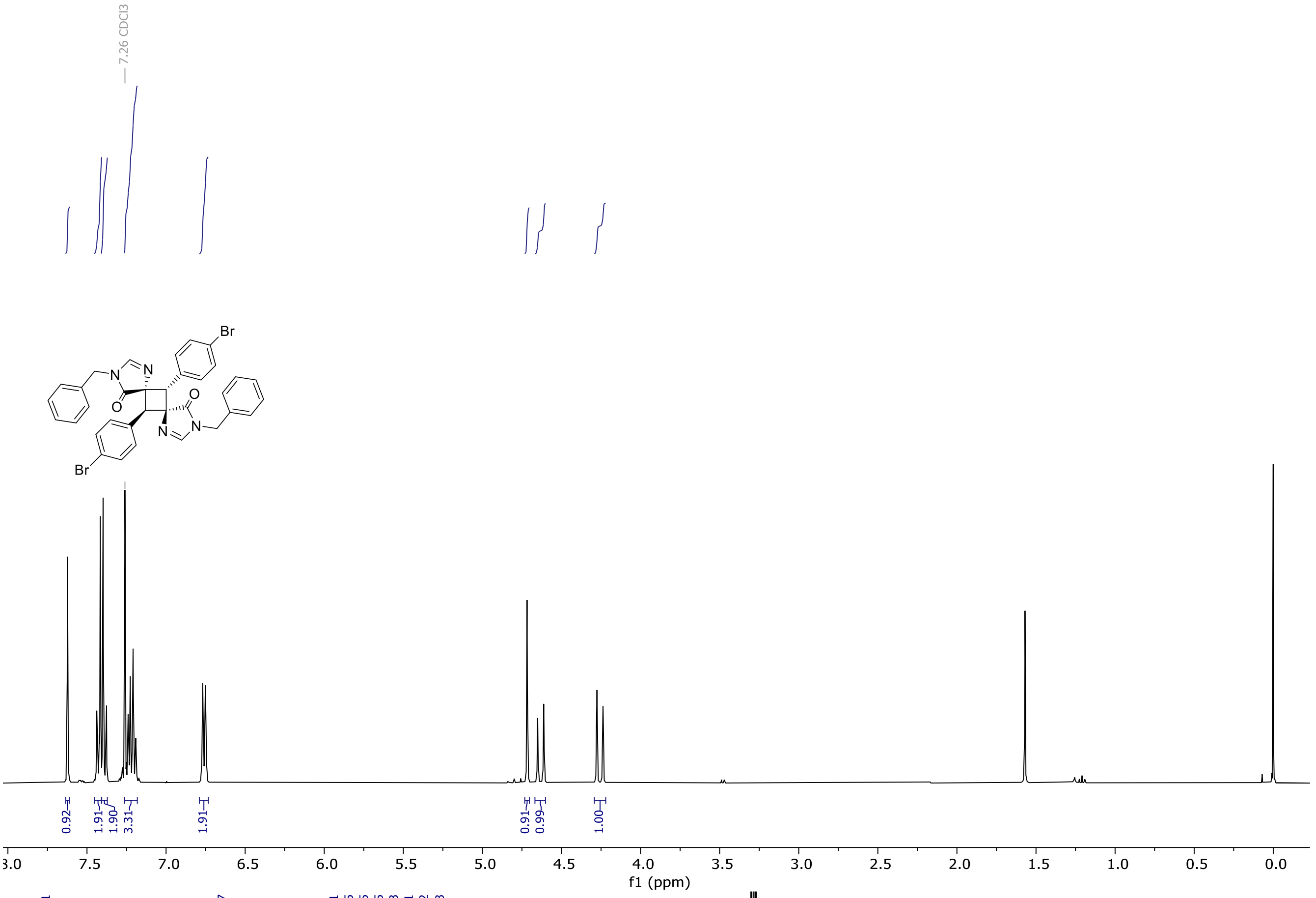

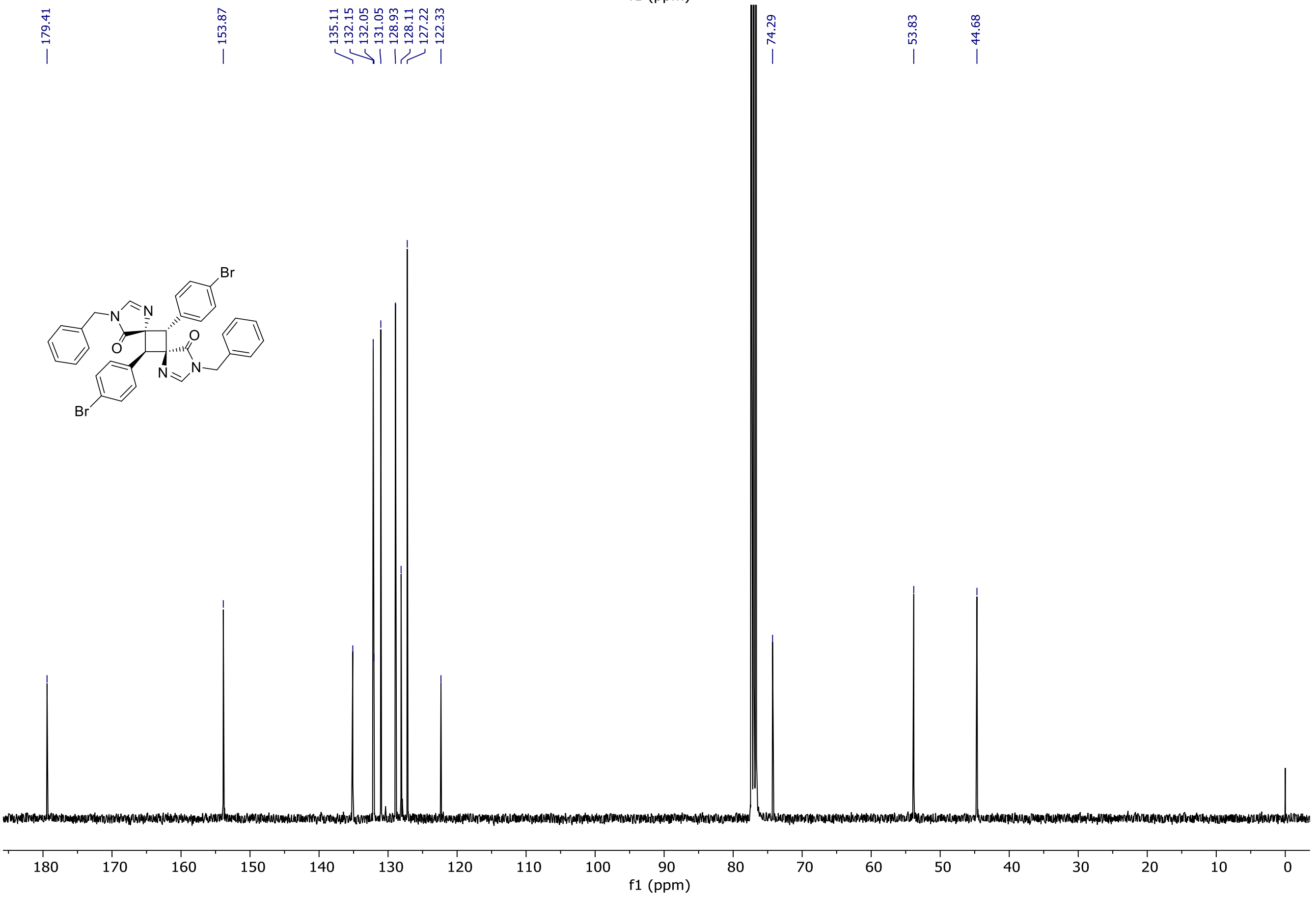

8.5. Compounds 9

2-(4-Chlorobenzyl)-8-(4-chlorophenyl)-6,7-dihydroimidazo[1,2-a]pyrazin-3(5*H*)-one (9a)

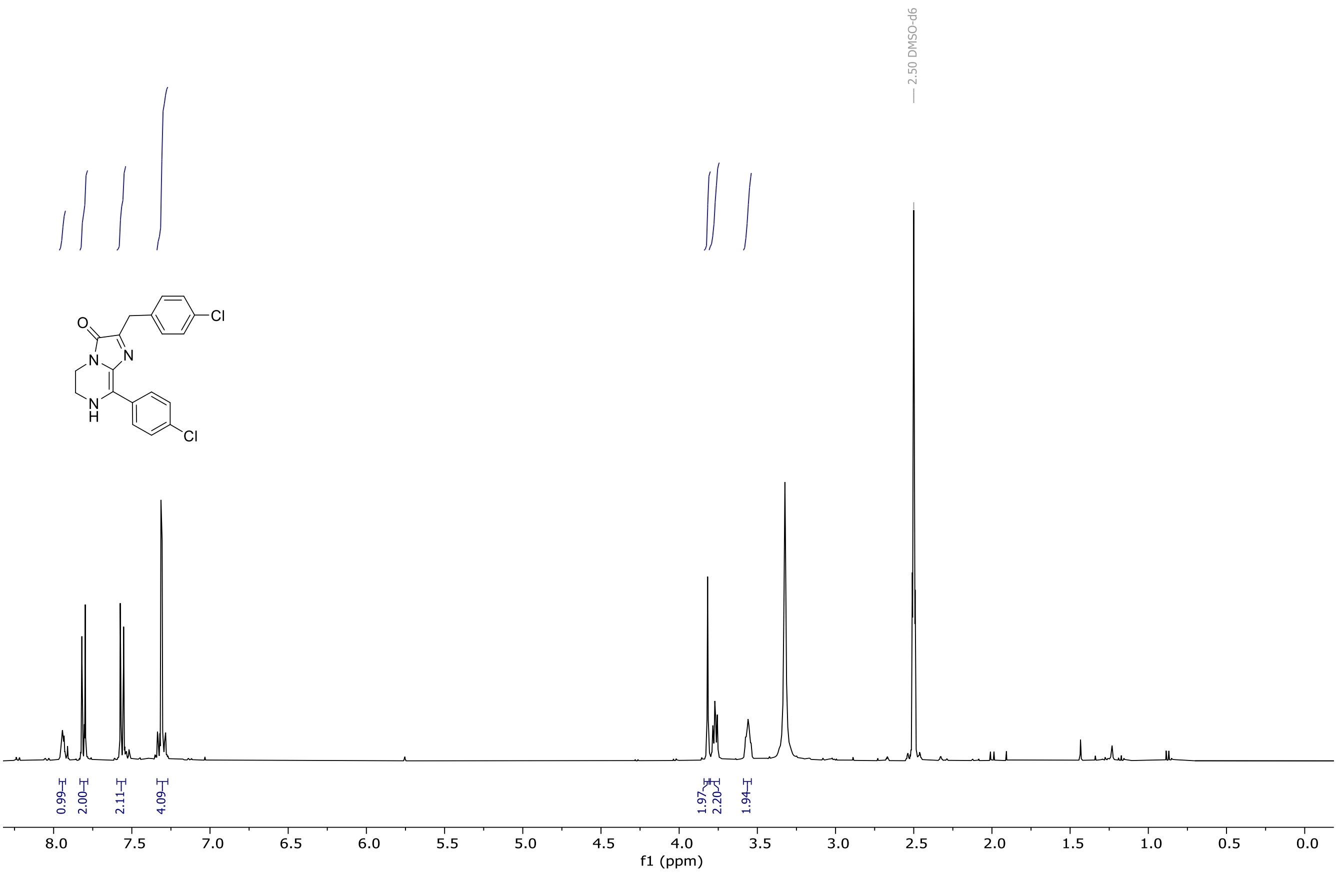

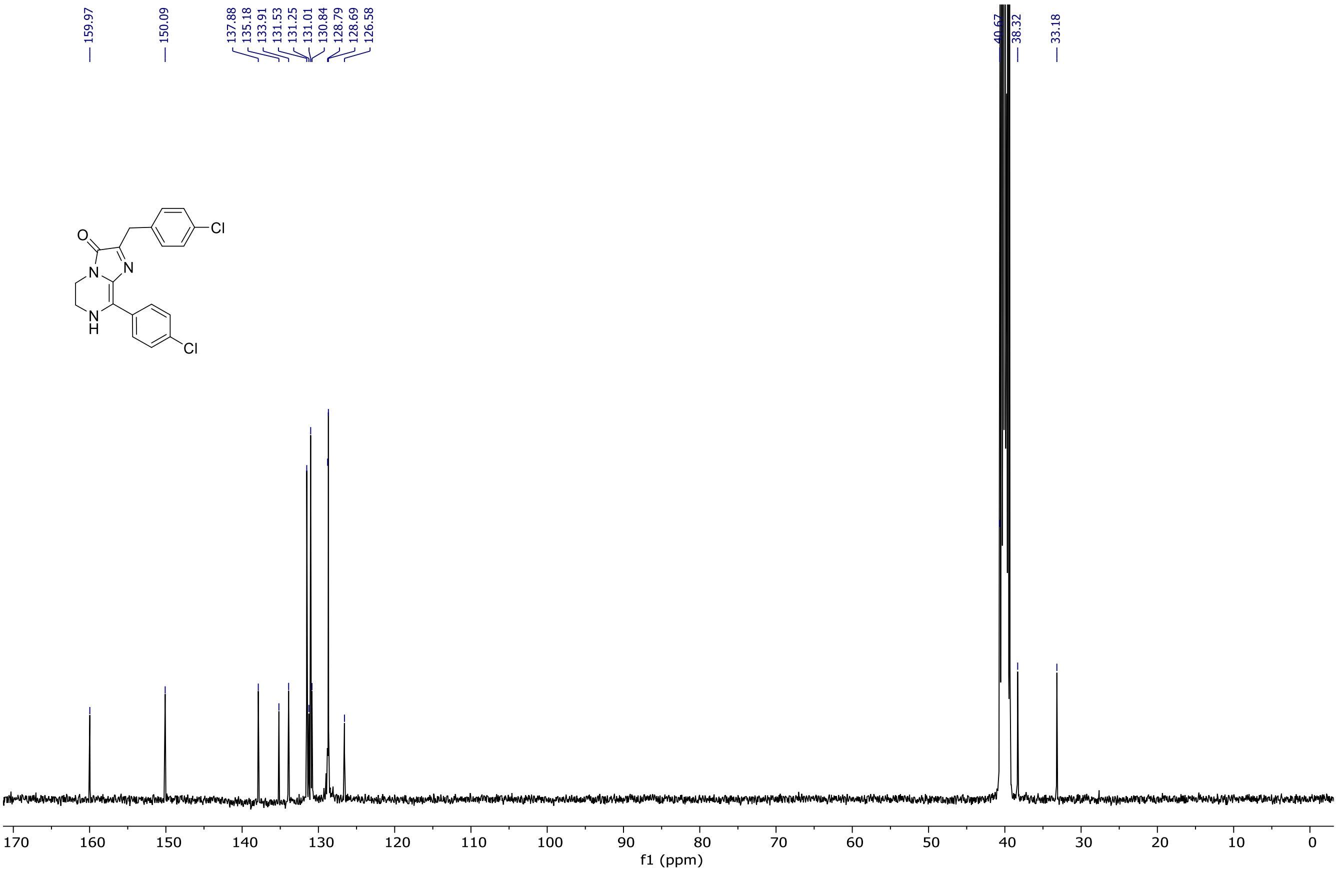

2-(4-Iodobenzyl)-8-(4-iodophenyl)-6,7-dihydroimidazo[1,2-a]pyrazin-3(5H)-one (9b)

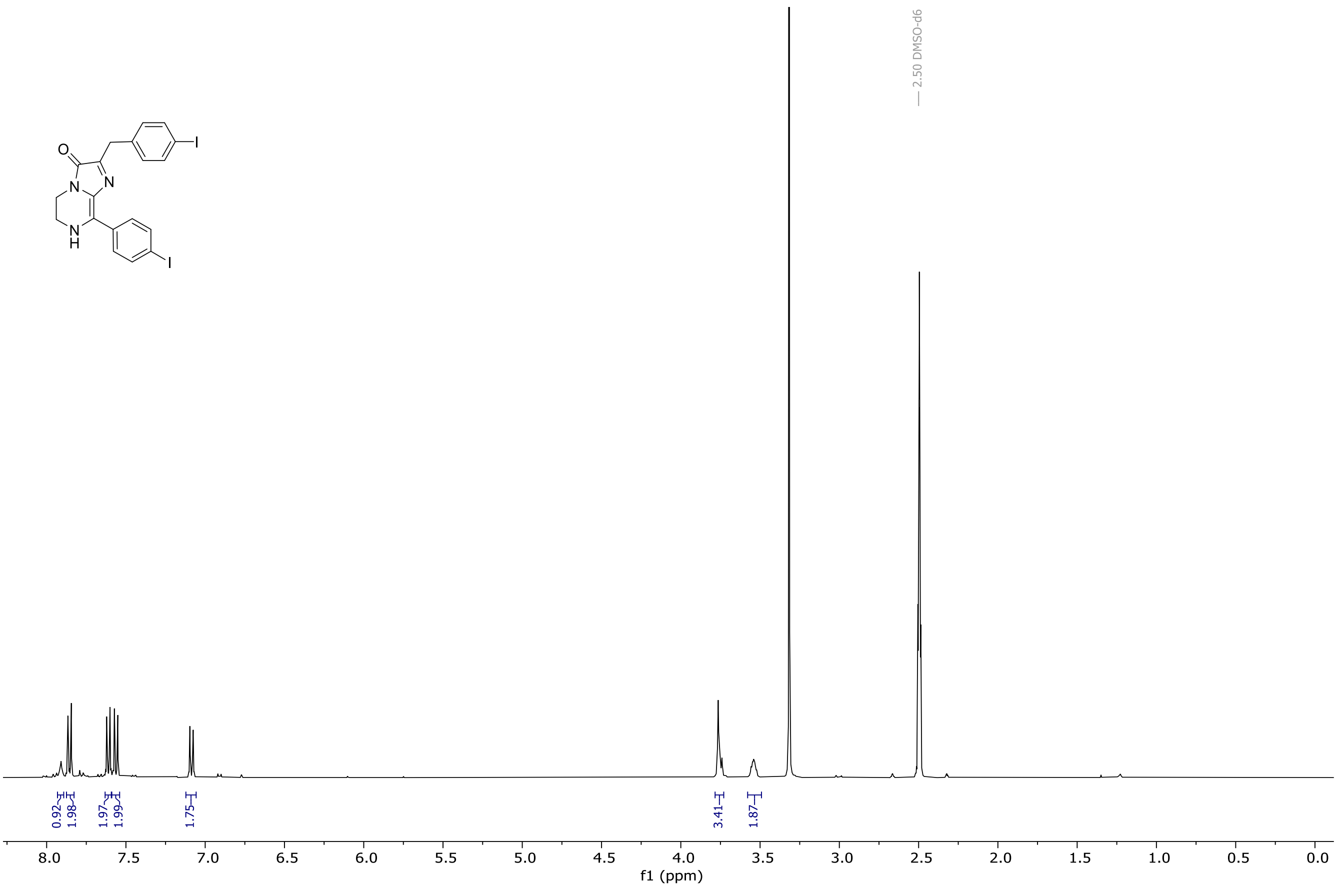

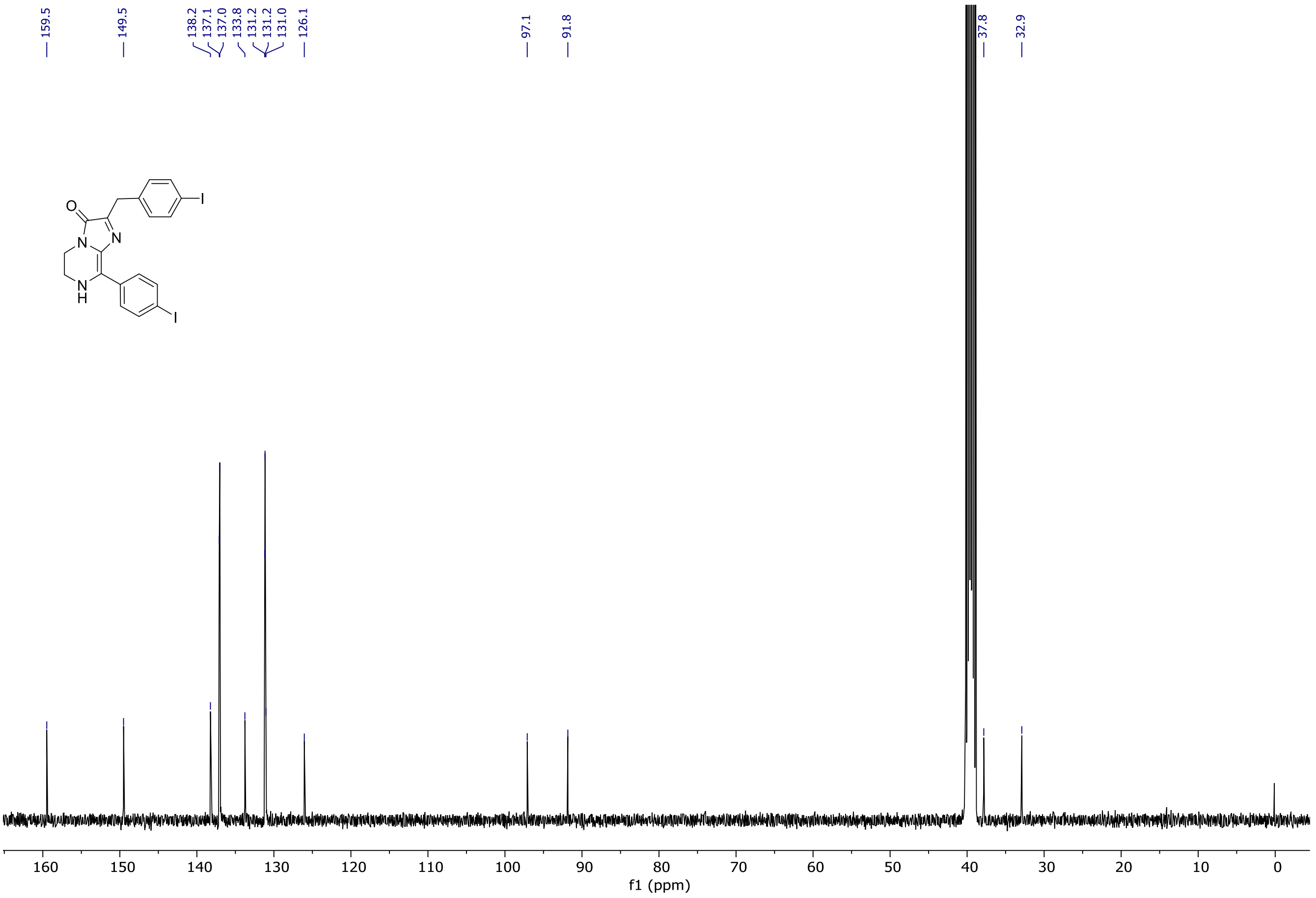

4-(2-(4-Cyanobenzyl)-3-oxo-3,5,6,7-tetrahydroimidazo[1,2-a]pyrazin-8-yl)benzonitrile (9c)

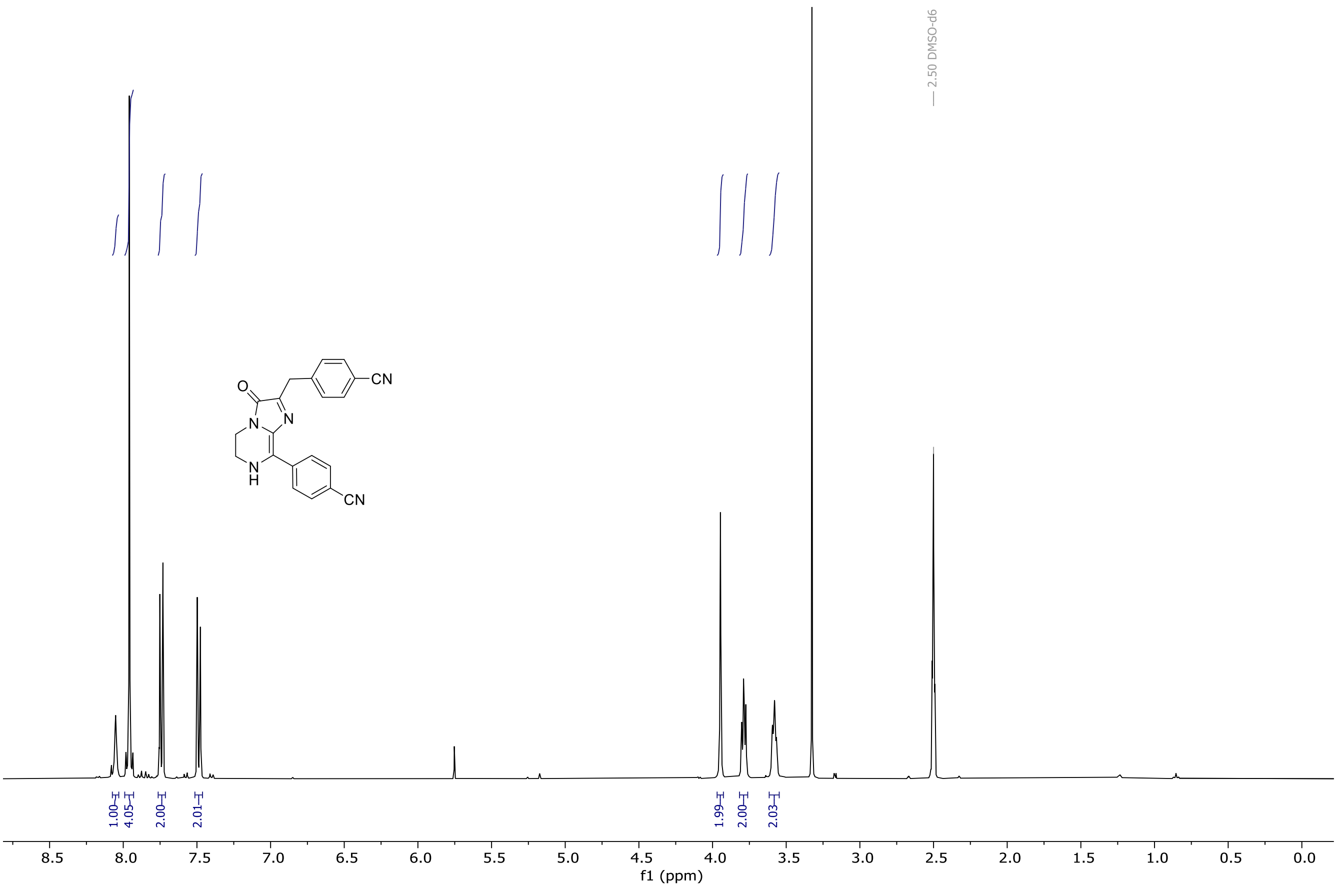

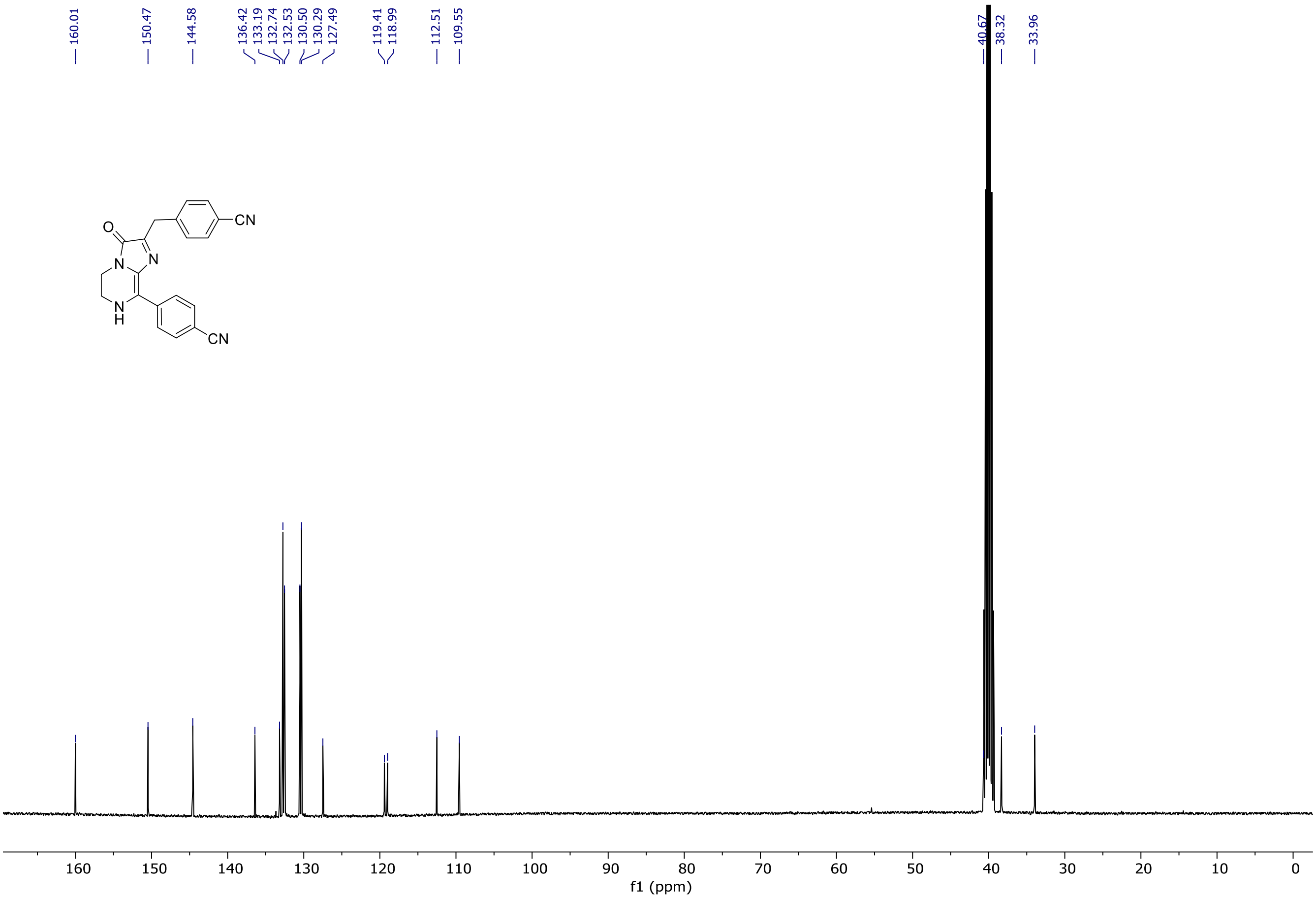

(5a*R*,9a*R*)-2-(4-Chlorobenzyl)-4-(4-chlorophenyl)-5a,6,7,8,9,9a-hexahydroimidazo[1,2-*a*]quinoxalin-1(5*H*)-one (9d)

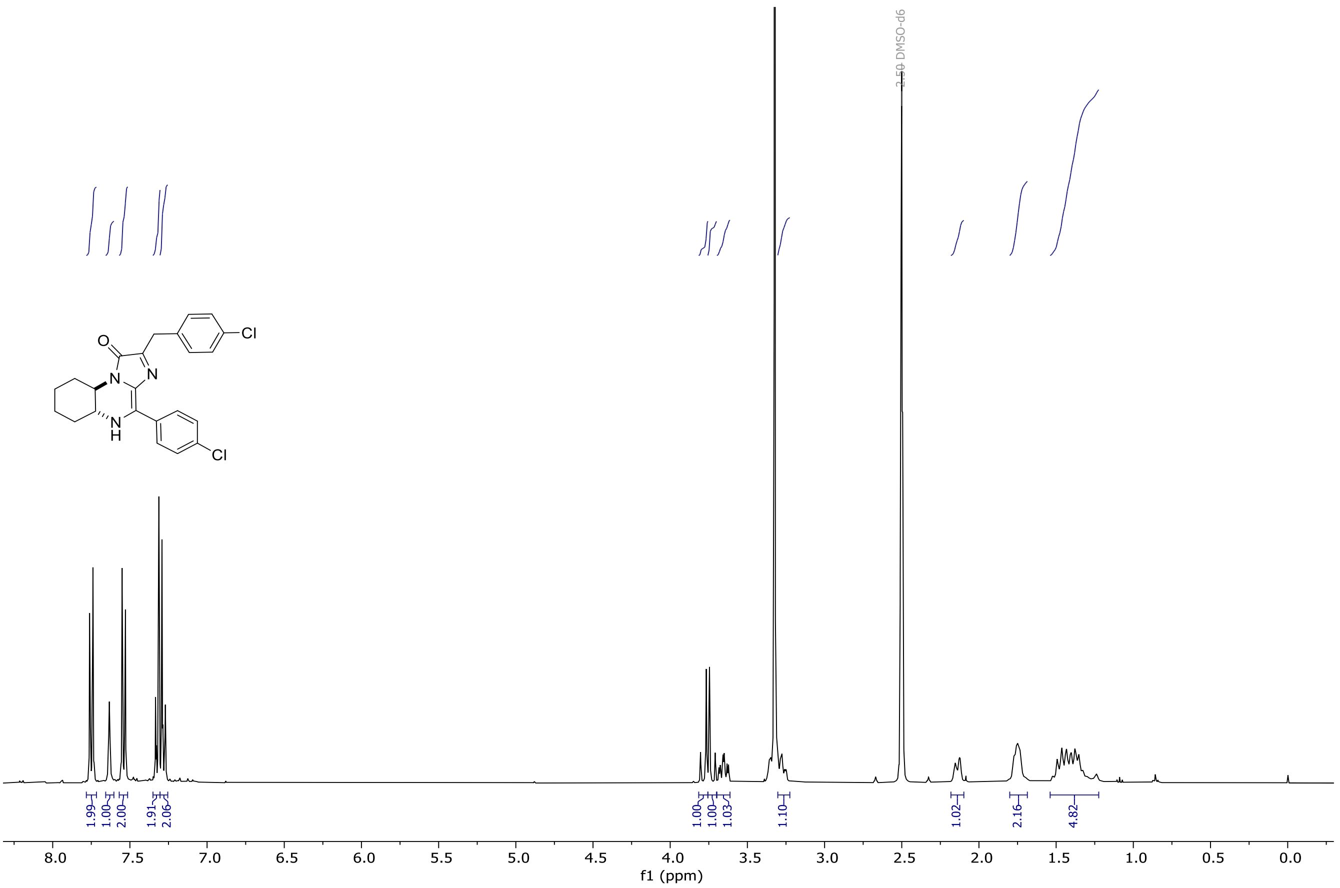

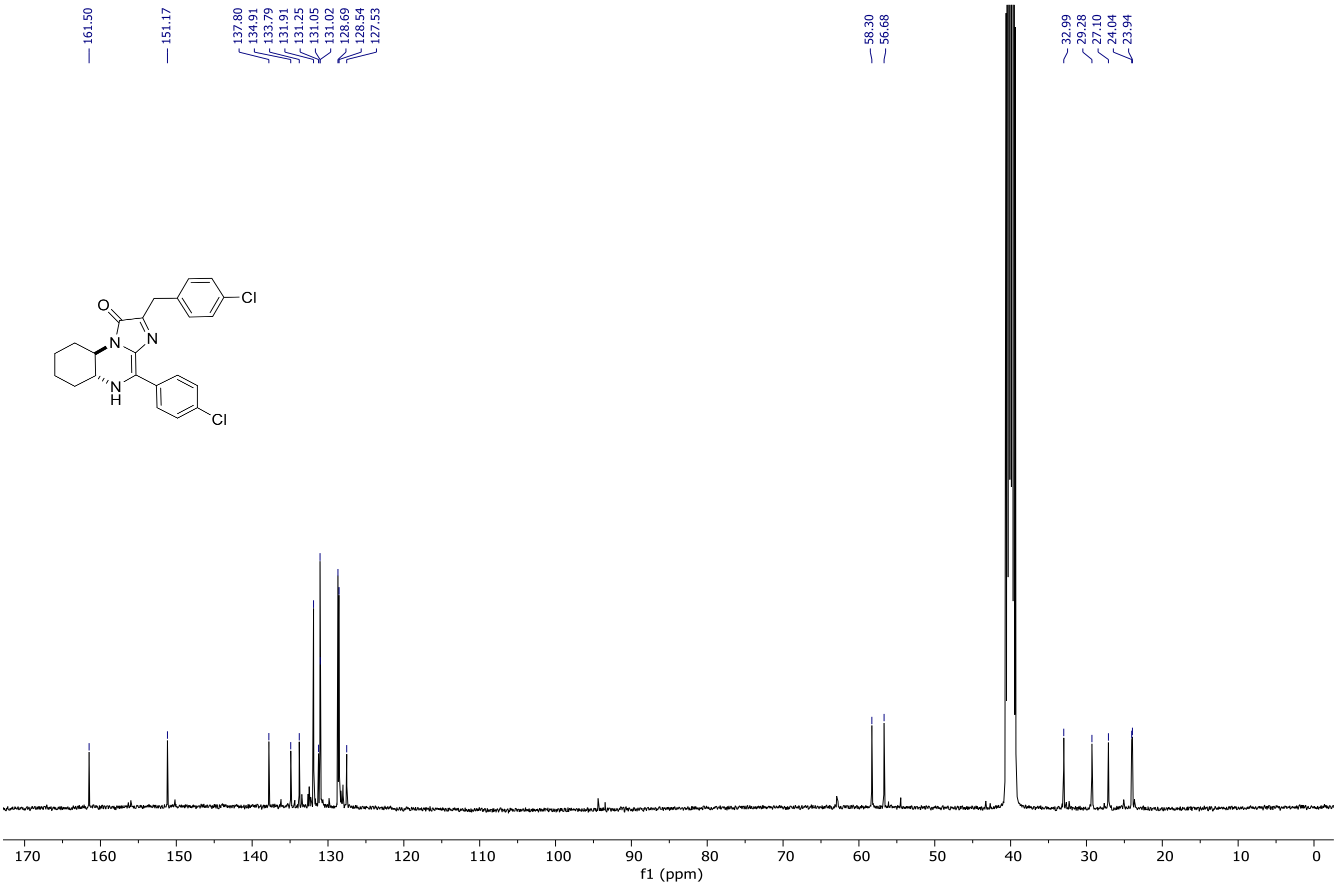

8-(Benzo[d][1,3]dioxol-5-yl)-2-(benzo[d][1,3]dioxol-5-ylmethyl)-6,7-dihydroimidazo[1,2-a]pyrazin-3(5*H*)-one (9e)

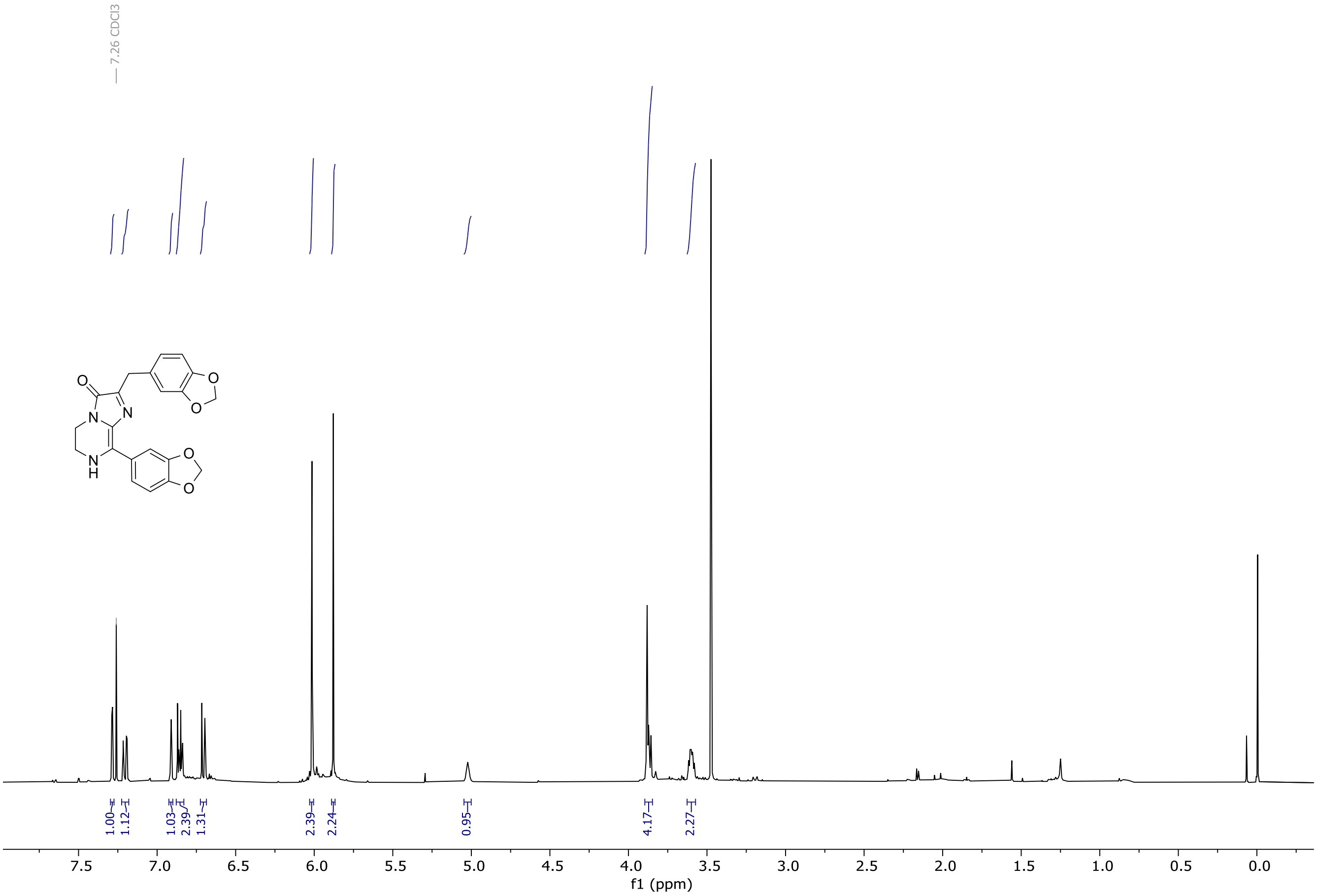

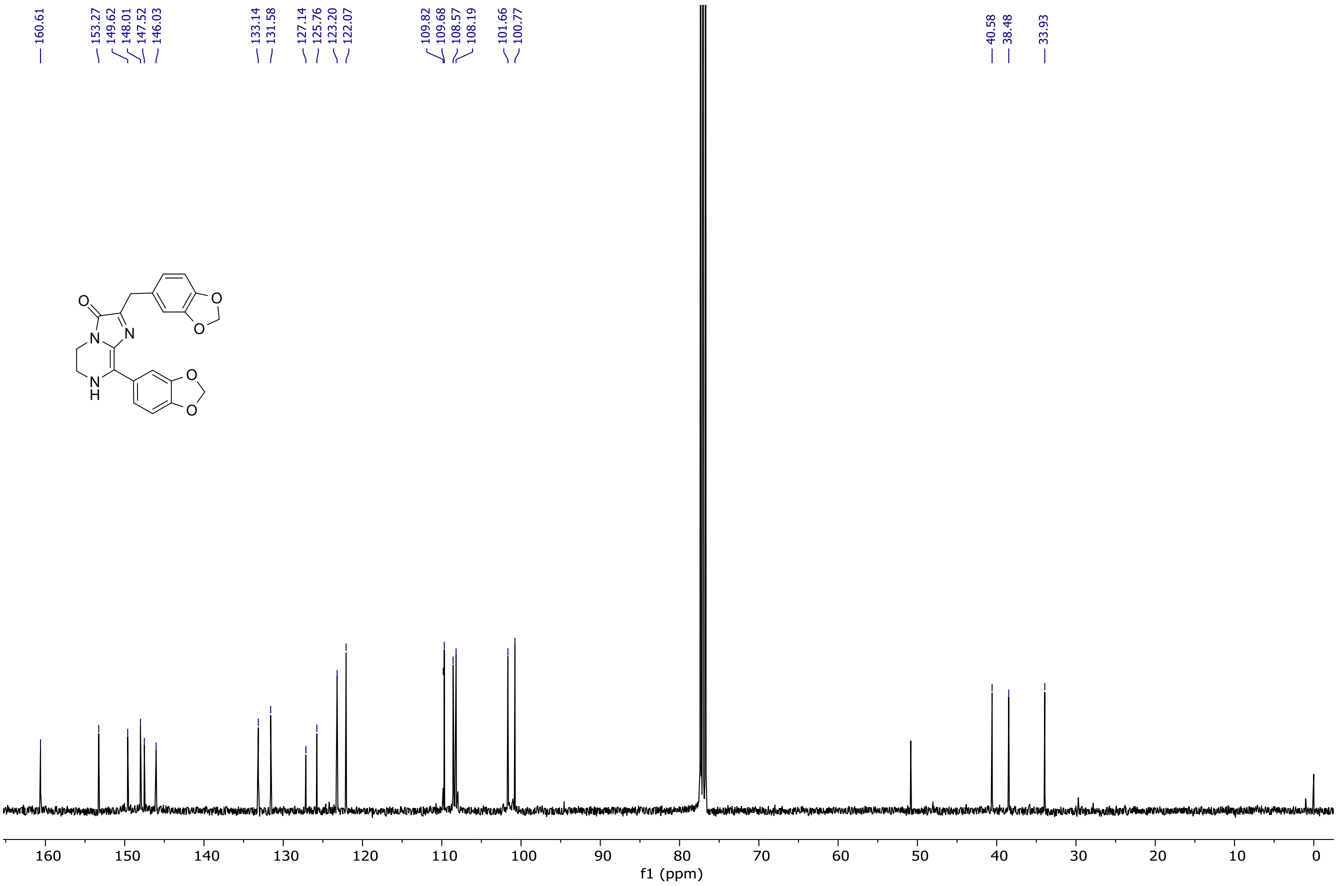

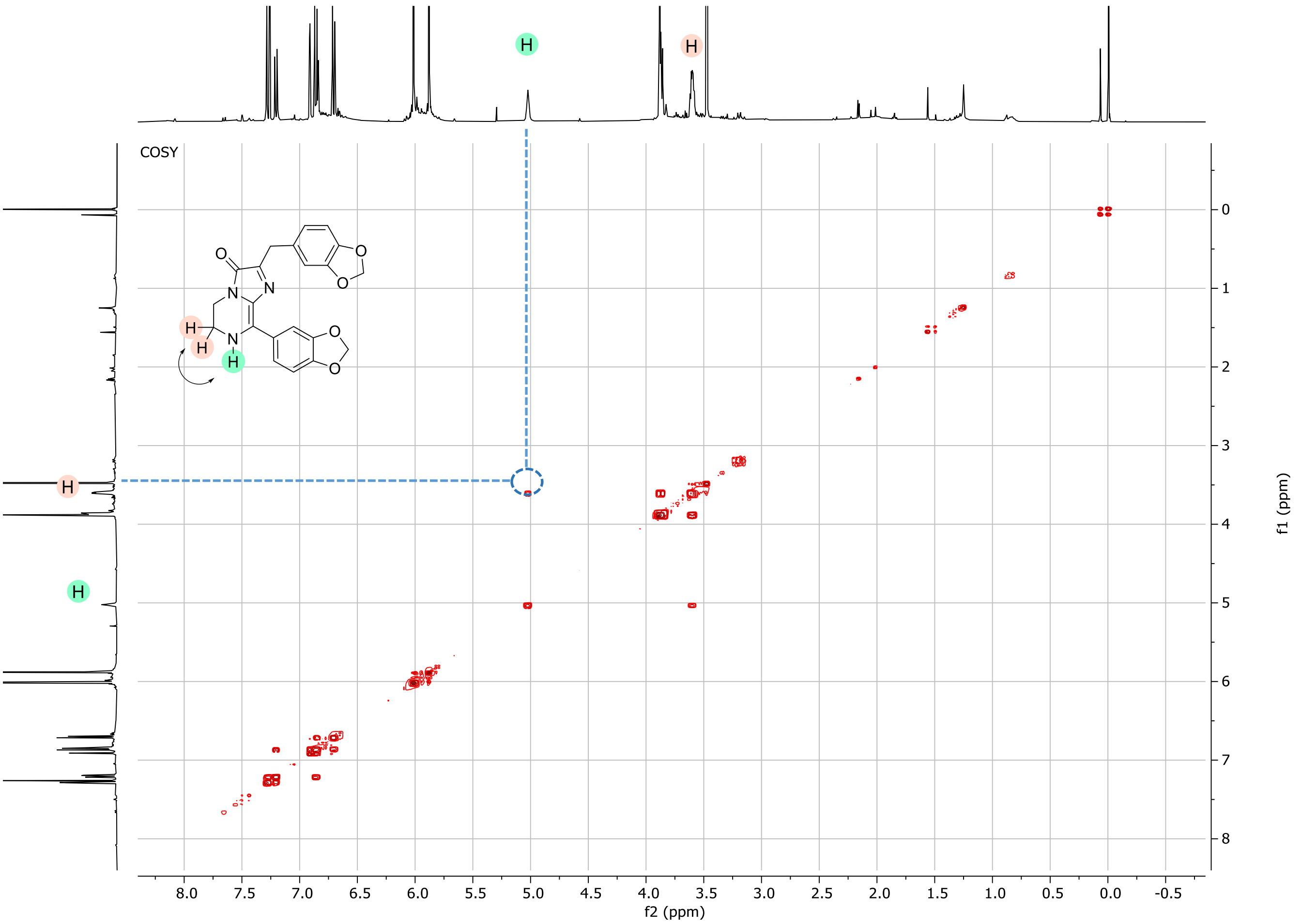
COSY
H
H
f2 (ppm)
f1 (ppm)
8.0 7.5 7.0 6.5 6.0 5.5 5.0 4.5 4.0 3.5 3.0 2.5 2.0 1.5 1.0 0.5 0.0 -0.5
0 1 2 3 4 5 6 7 8

2-(3,4,5-Trimethoxybenzyl)-8-(3,4,5-trimethoxyphenyl)-6,7-dihydroimidazo[1,2-*a*]pyrazin-3(5*H*)-one (9f)

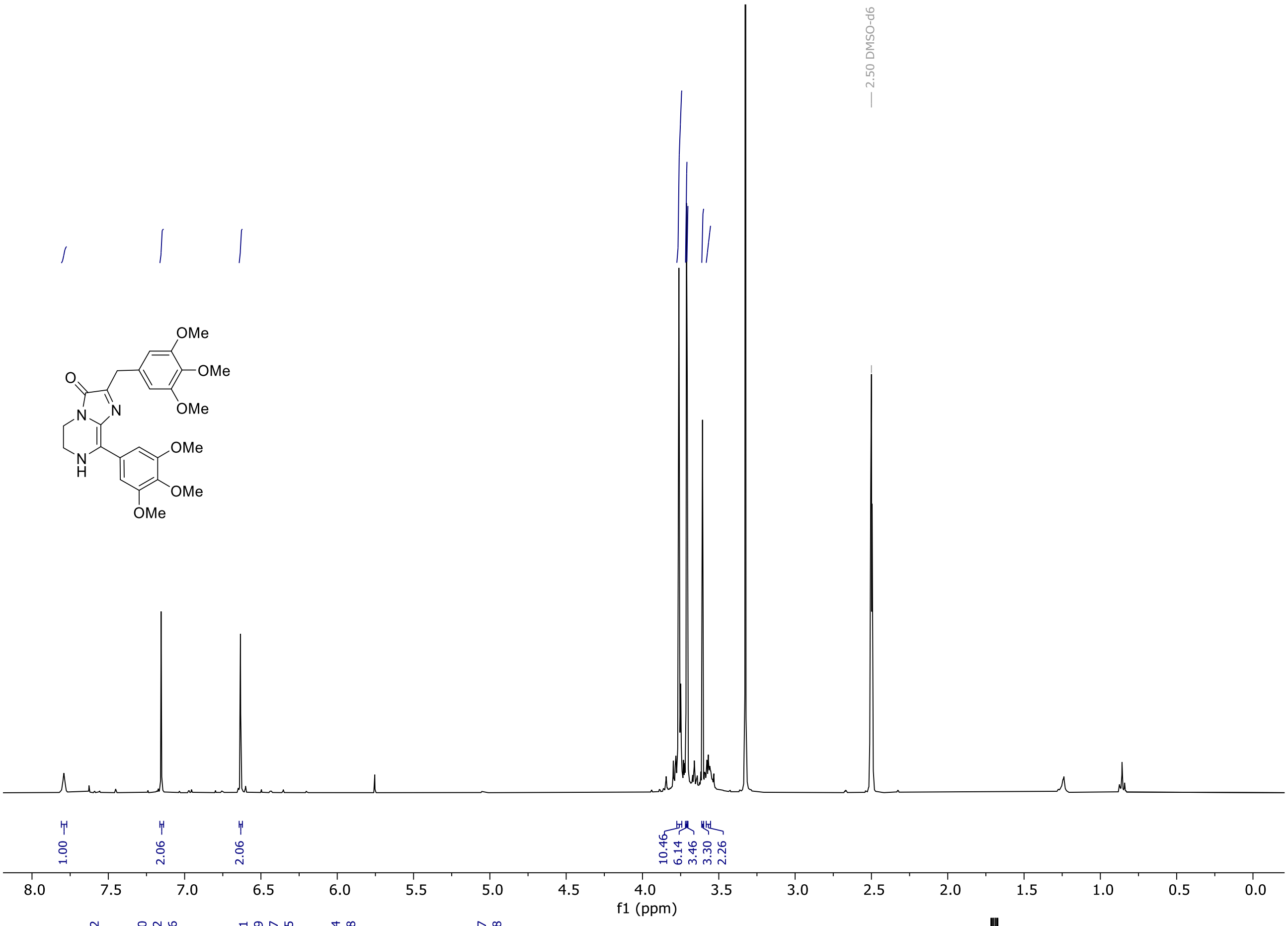

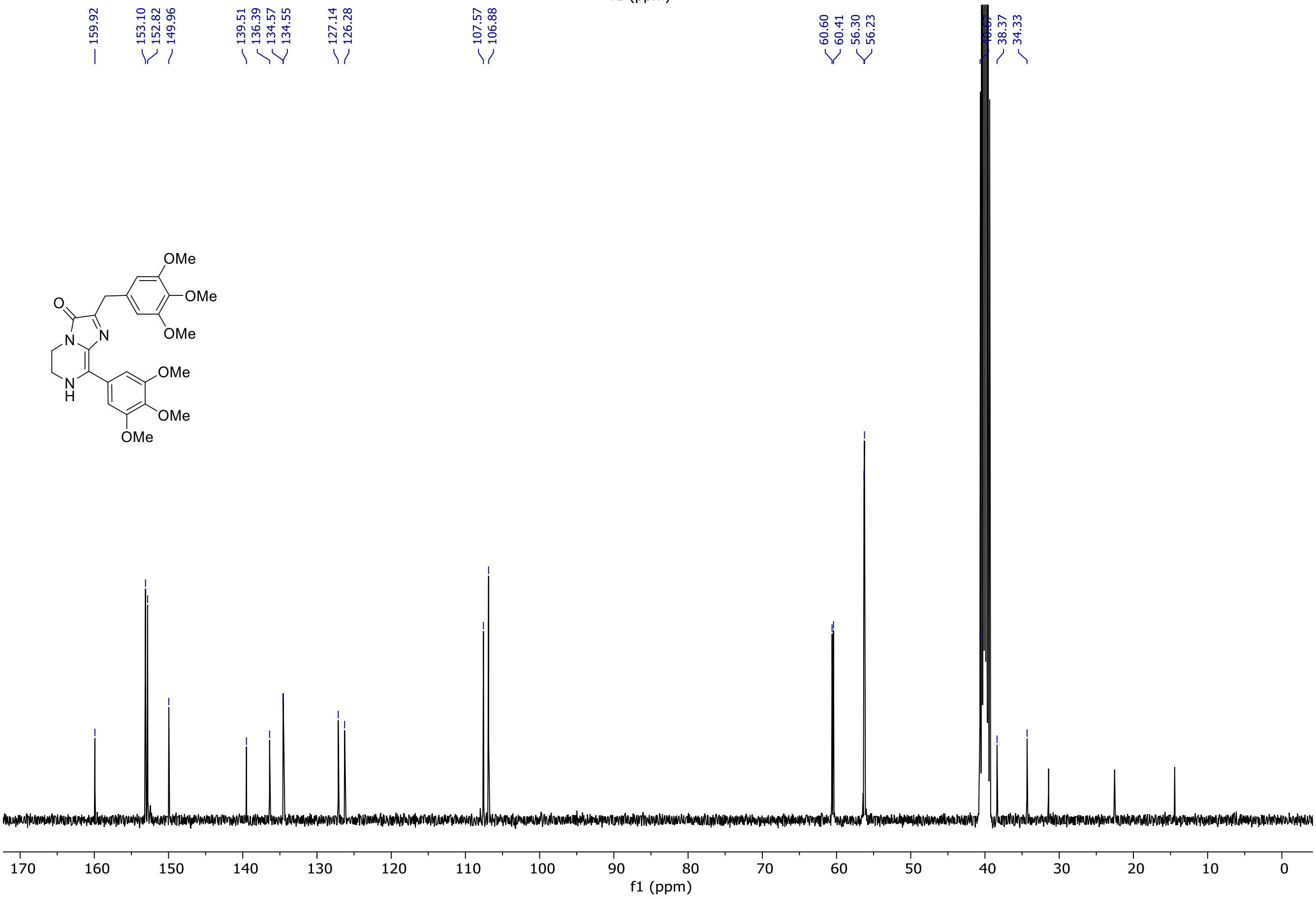

(*Z*)-8-(benzo[*d*][1,3]dioxol-5-yl)-2-(benzo[*d*][1,3]dioxol-5-ylmethylene)-1,5,6,7-tetrahydroimidazo[1,2-*a*]pyrazin-3(2*H*)-one (9e-imine)

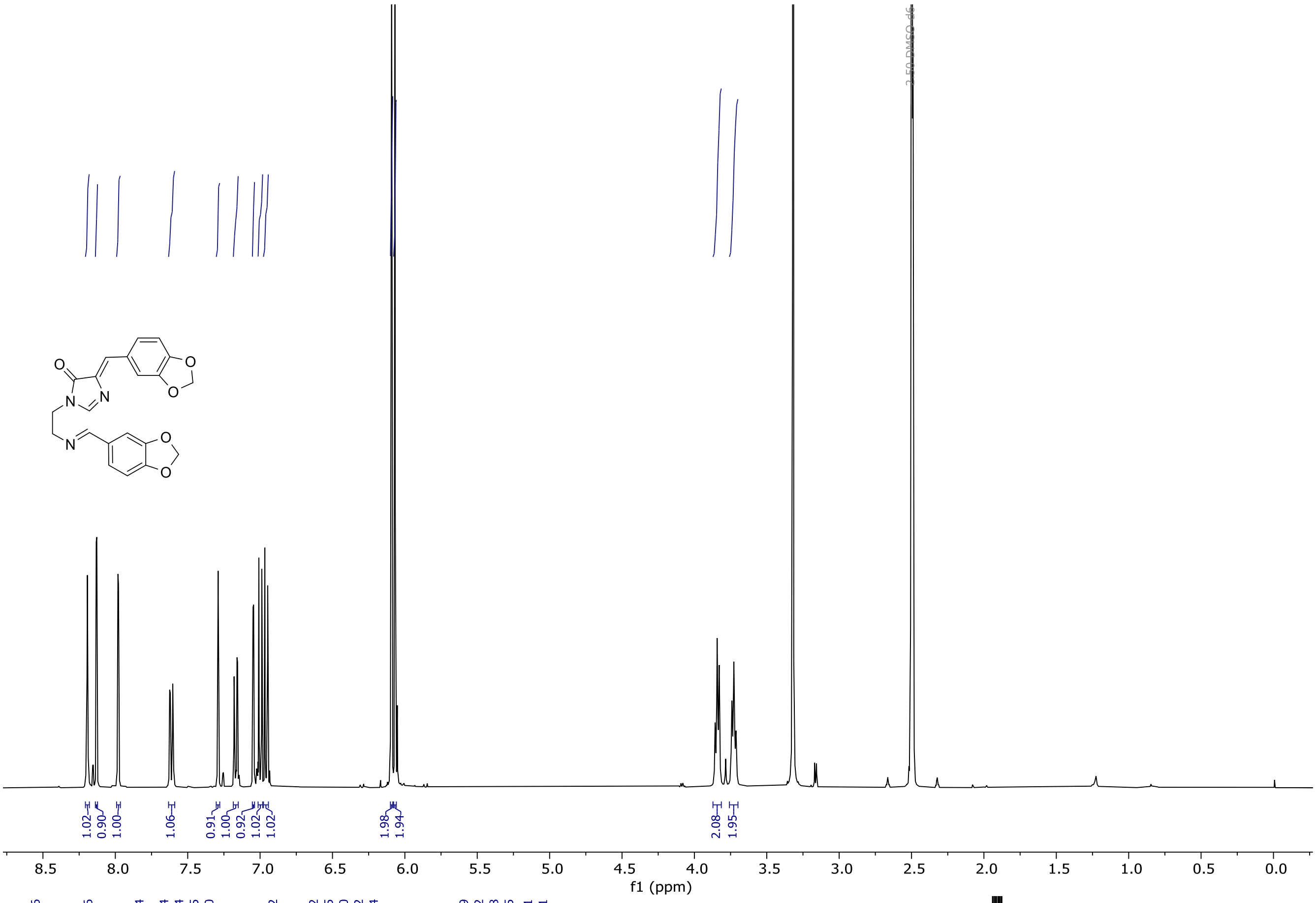

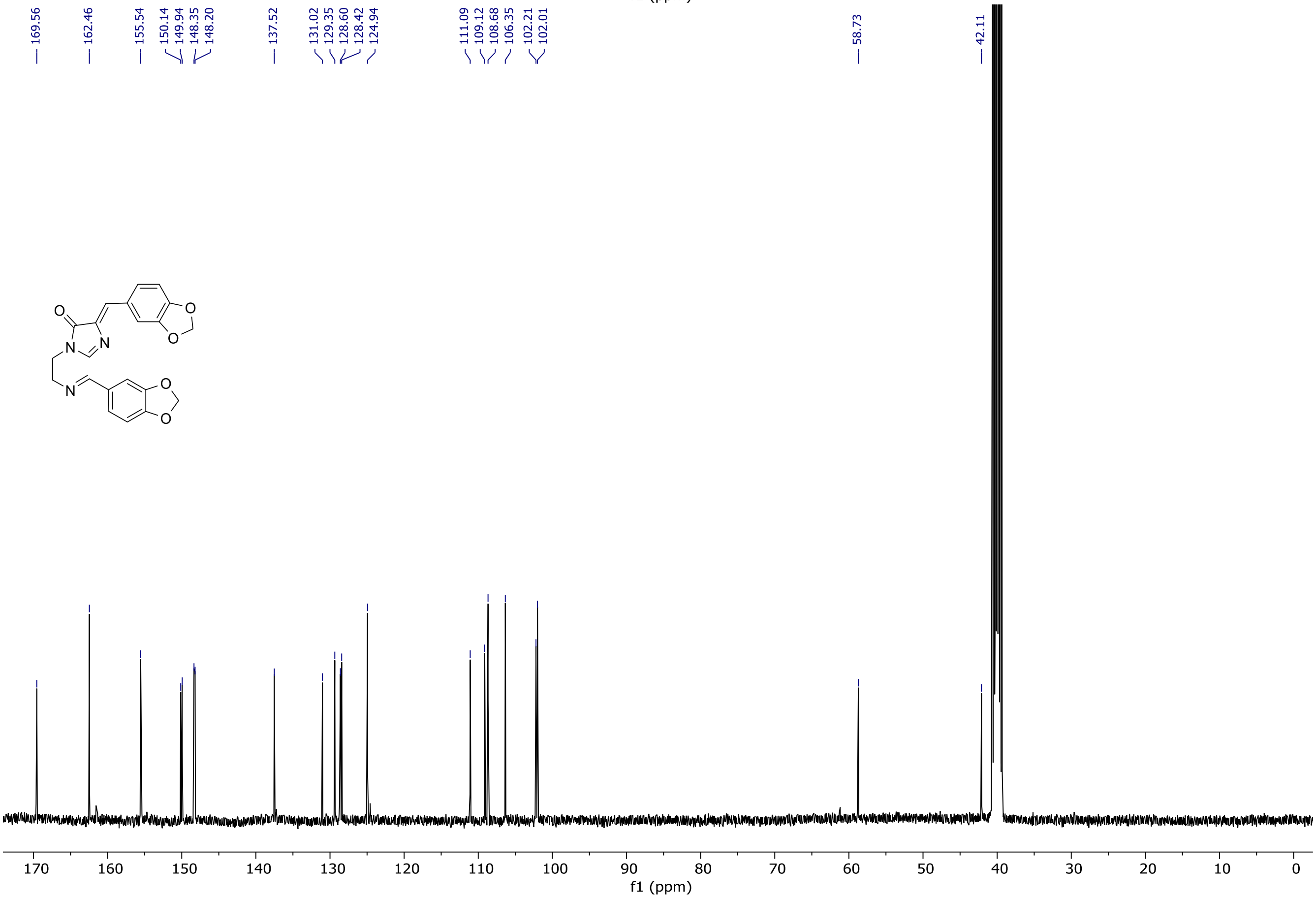

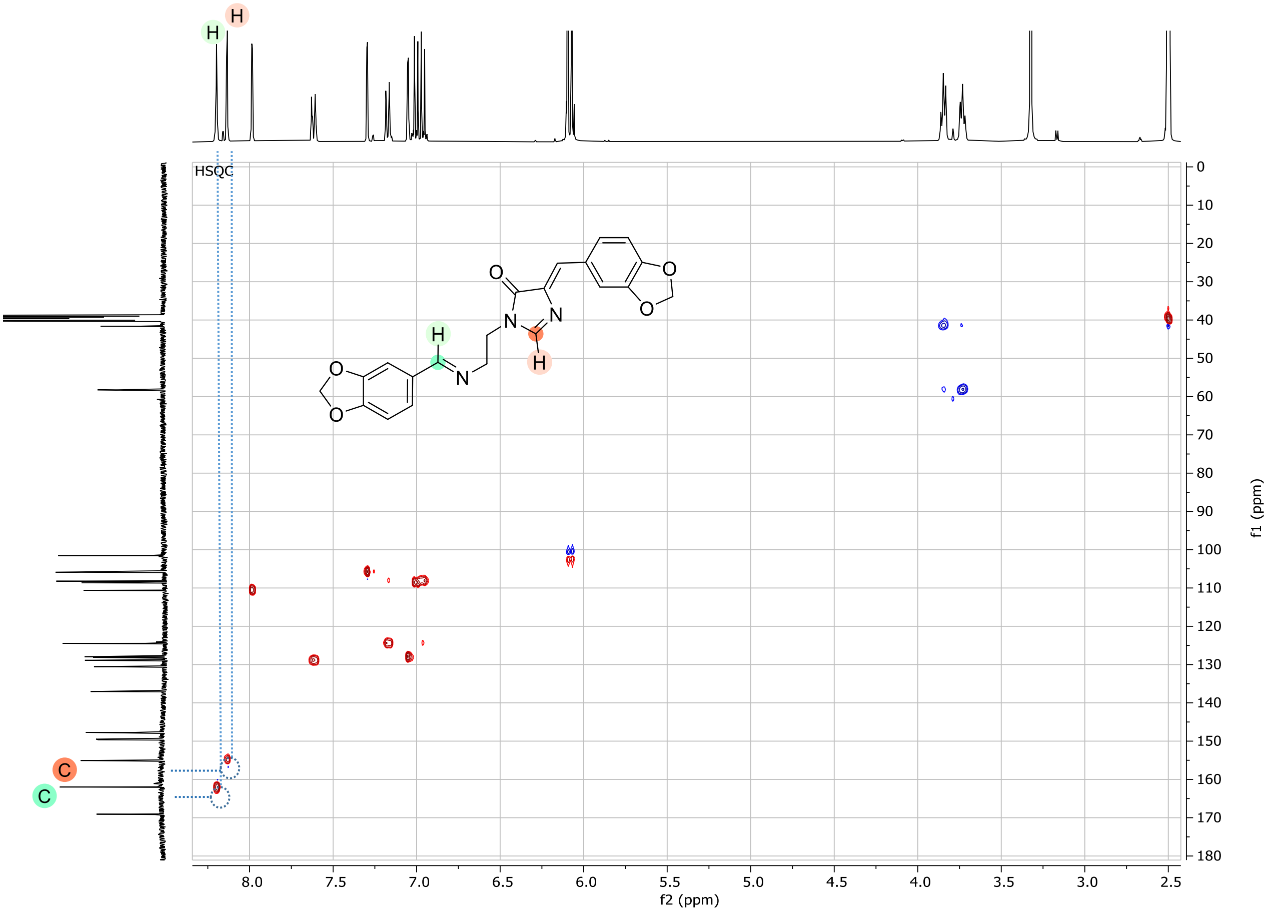
HSQC
f2 (ppm)
f1 (ppm)

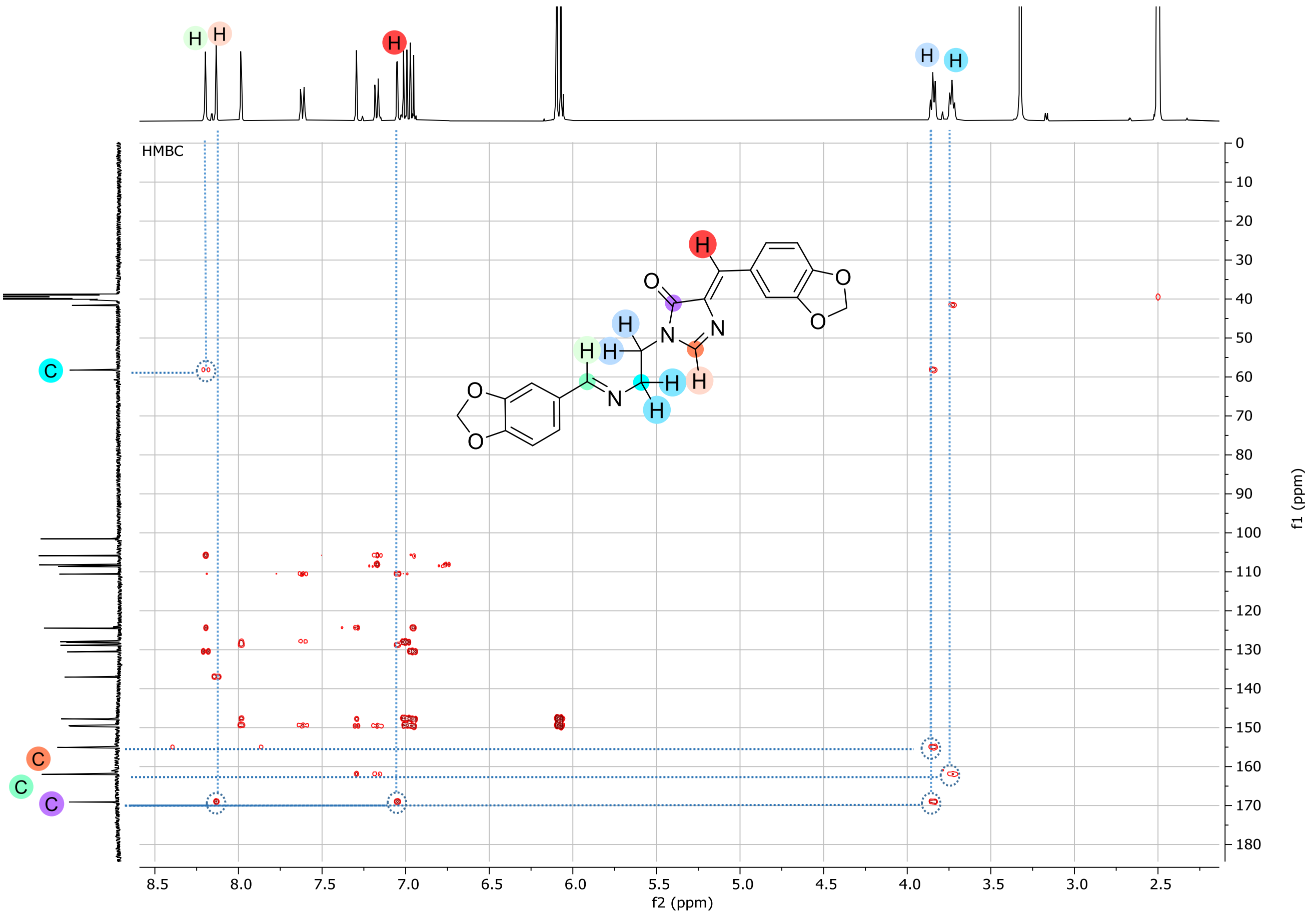
HMBC
f2 (ppm)
f1 (ppm)

(*Z*)-2-(3,4,5-Trimethoxybenzylidene)-8-(3,4,5-trimethoxyphenyl)-5,6,7,8-tetrahydroimidazo[1,2-*a*]pyrazin-3(2*H*)-one (9f')

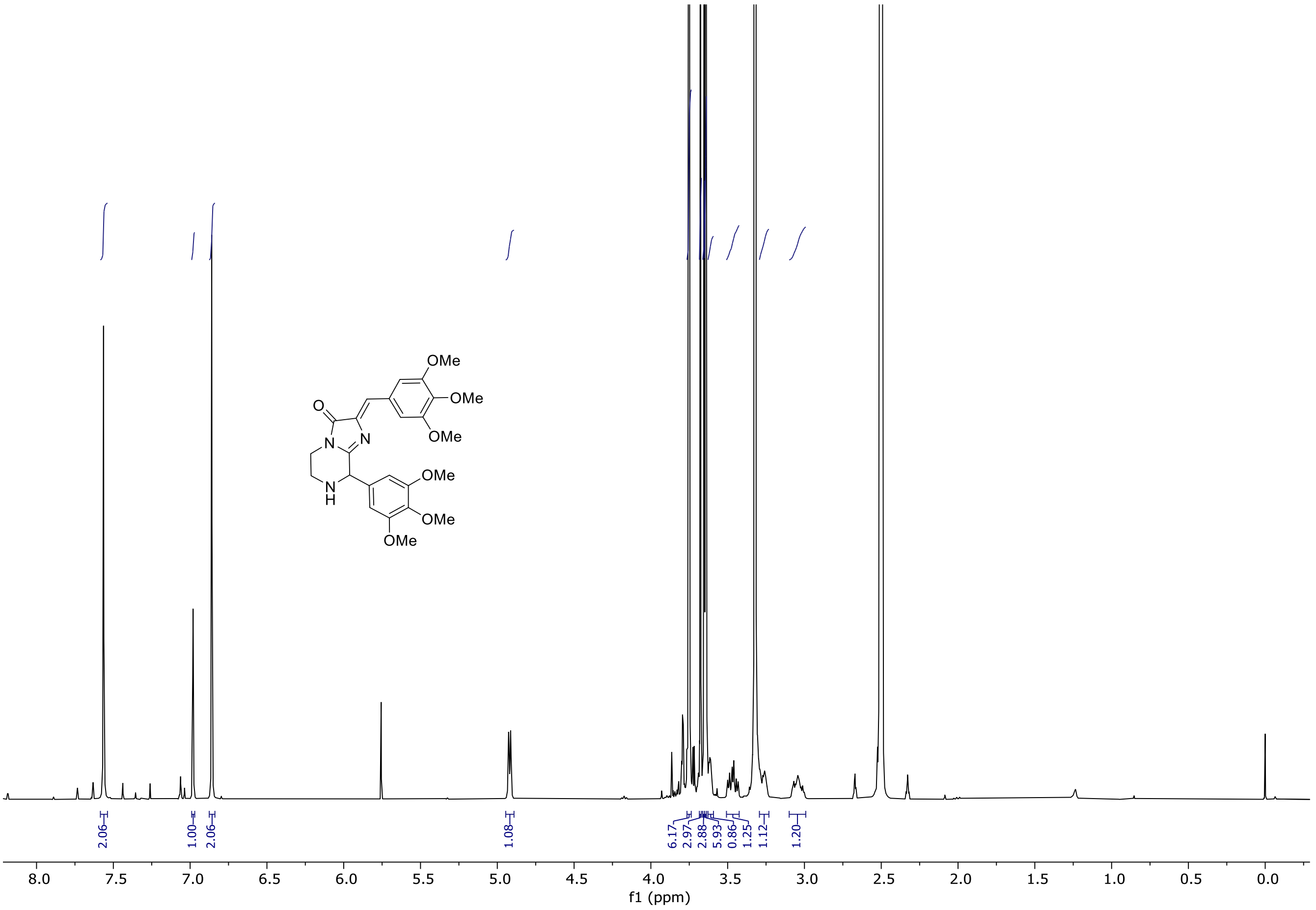

8.6. Compound 10

(*Z*)-3-(4-(4-methoxybenzylidene)-5-oxo-1-(pyridin-2-ylmethyl)-4,5-dihydro-1*H*-imidazol-2-yl)benzonitrile (10)

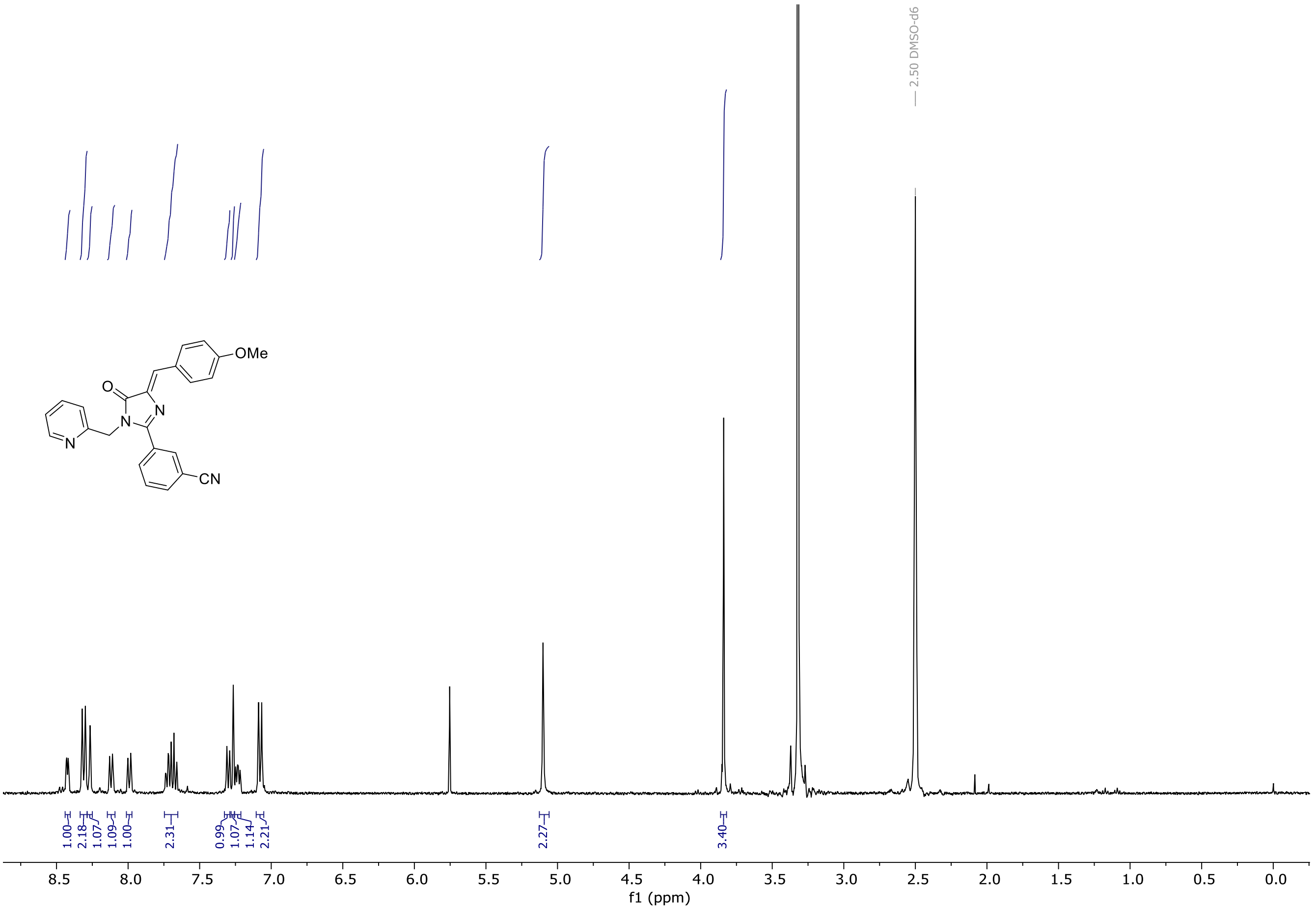

8.7. Compounds 11

2-(Benzo[*d*][1,3]dioxol-5-ylmethyl)-5,6,7,8-tetrahydroimidazo[1,2-*a*]pyrimidin-3(2*H*)-one (11a)

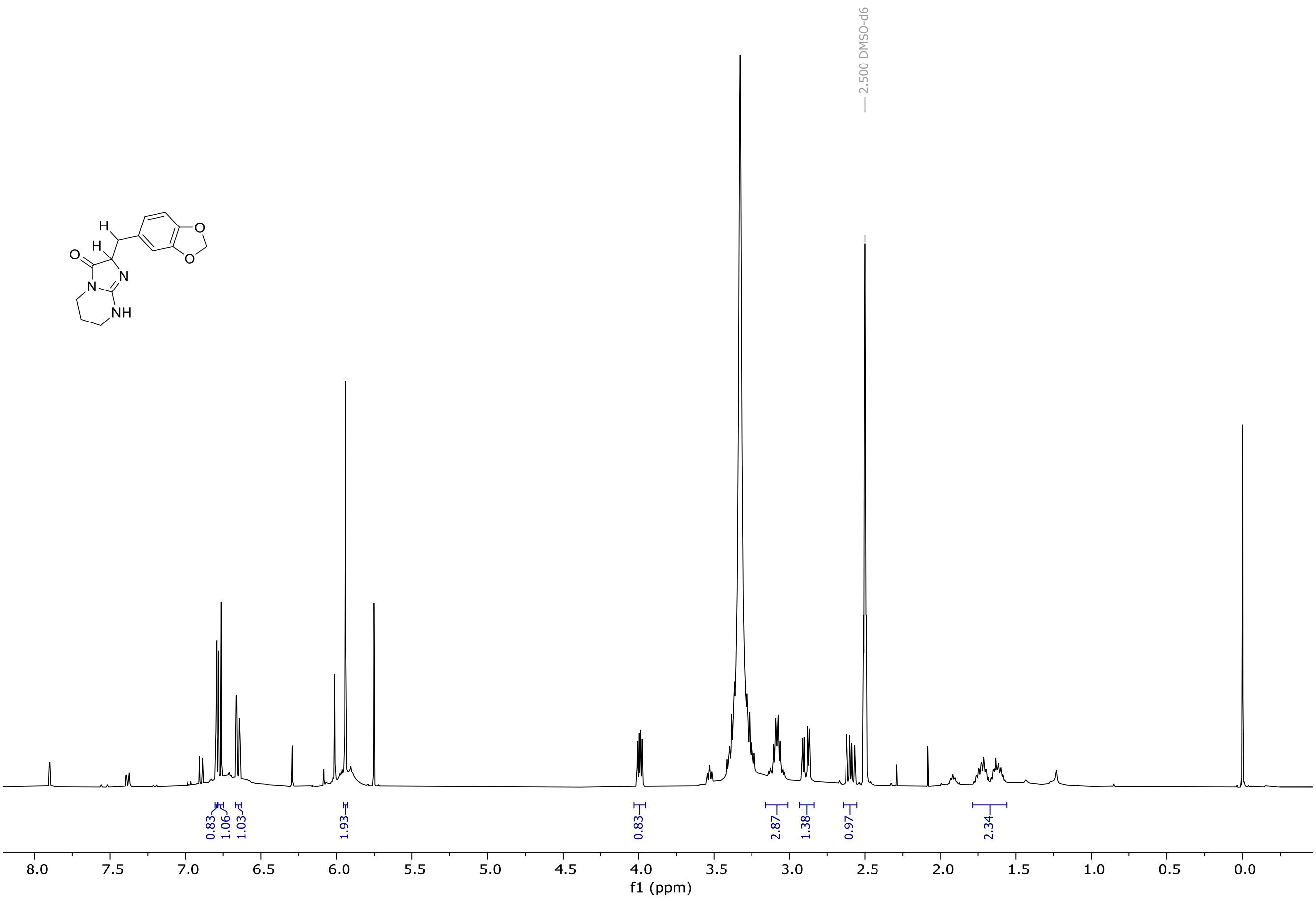

3-Benzyl-5-(4-chlorobenzyl)-2-(pyrrolidin-1-yl)-3,5-dihydro-4*H*-imidazol-4-one (11d)

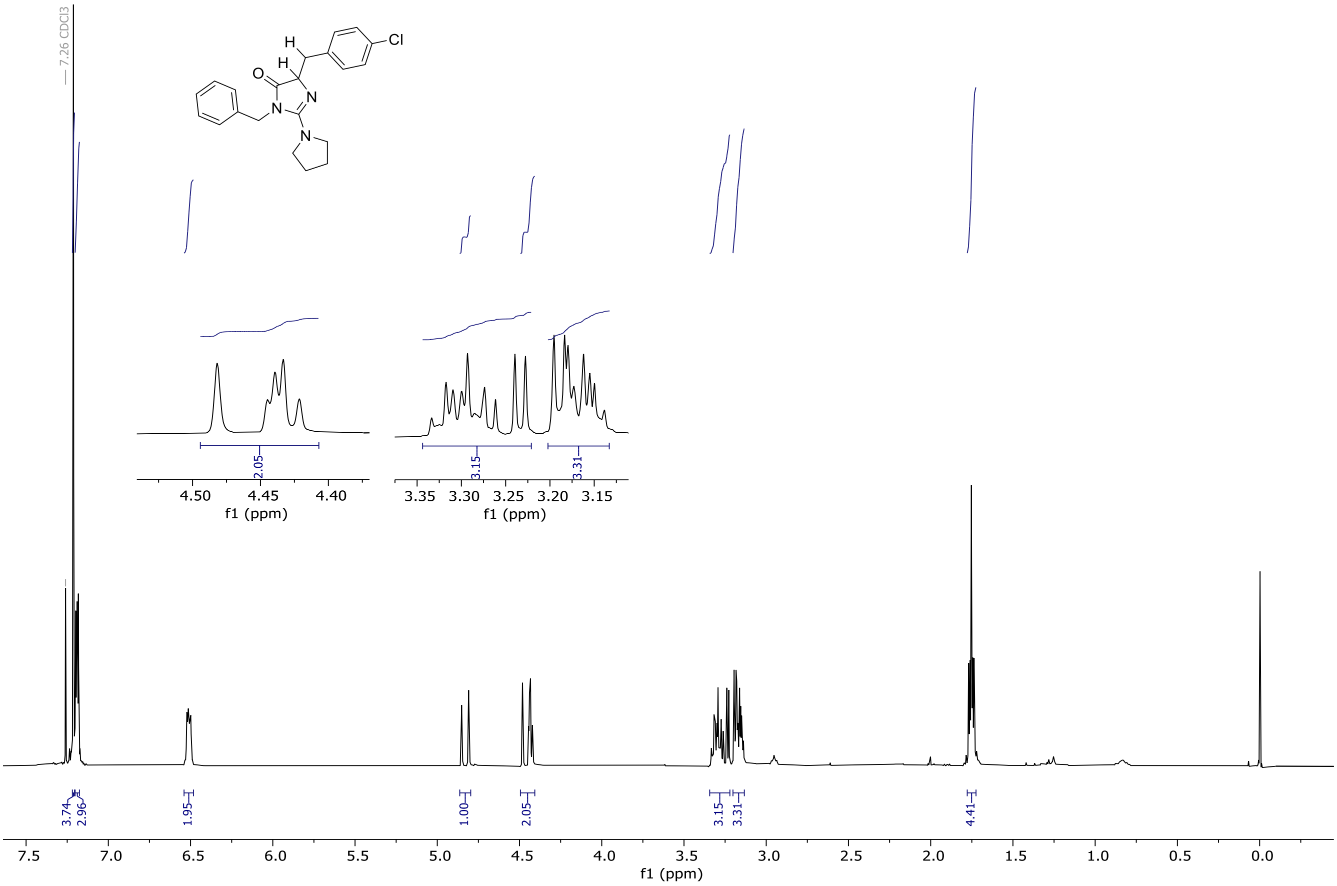

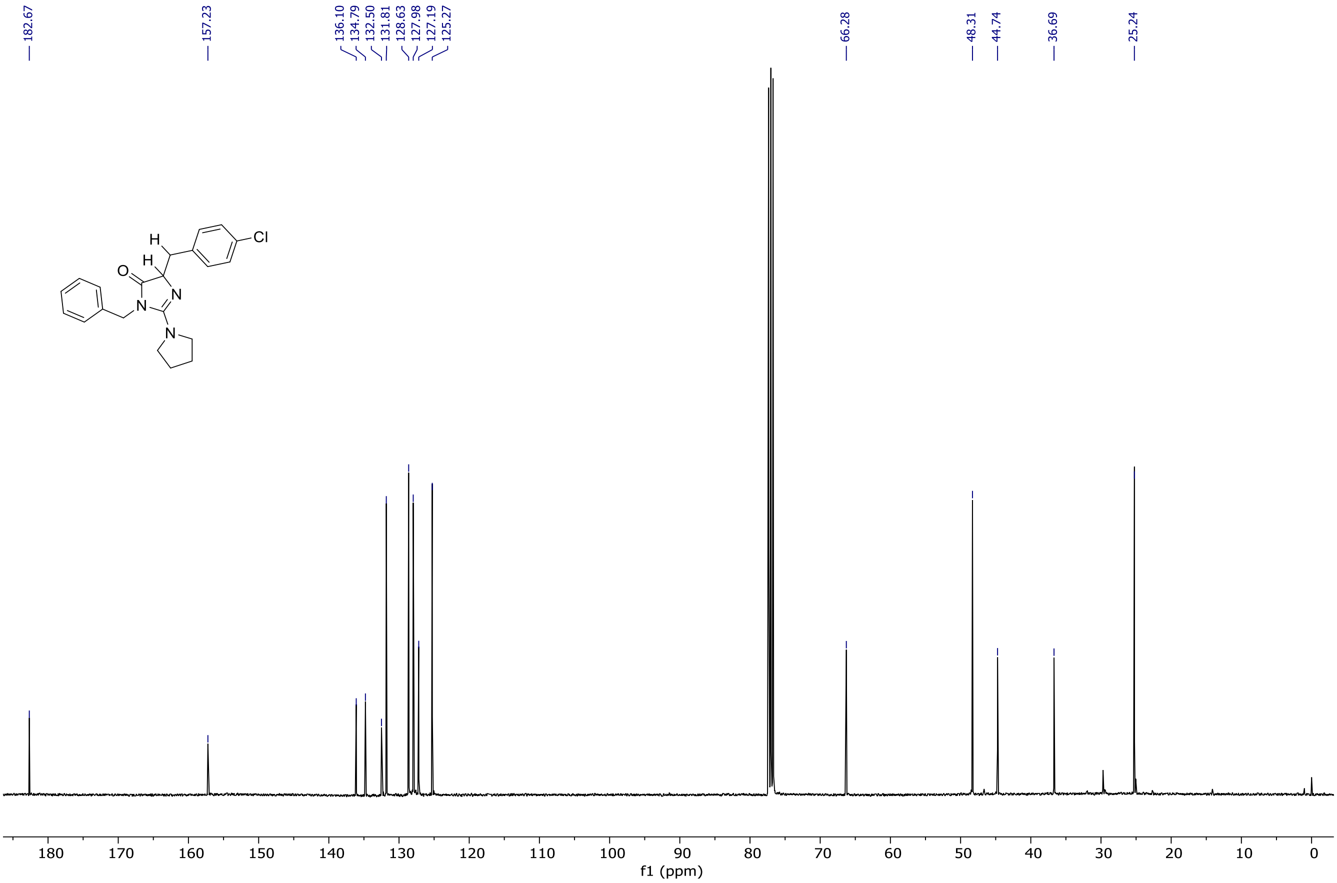

3-Benzyl-5-((4-chlorophenyl)methyl-*d*$_2$)-2-(pyrrolidin-1-yl)-3,5-dihydro-4*H*-imidazol-4-one (11d-*d*$_2$)

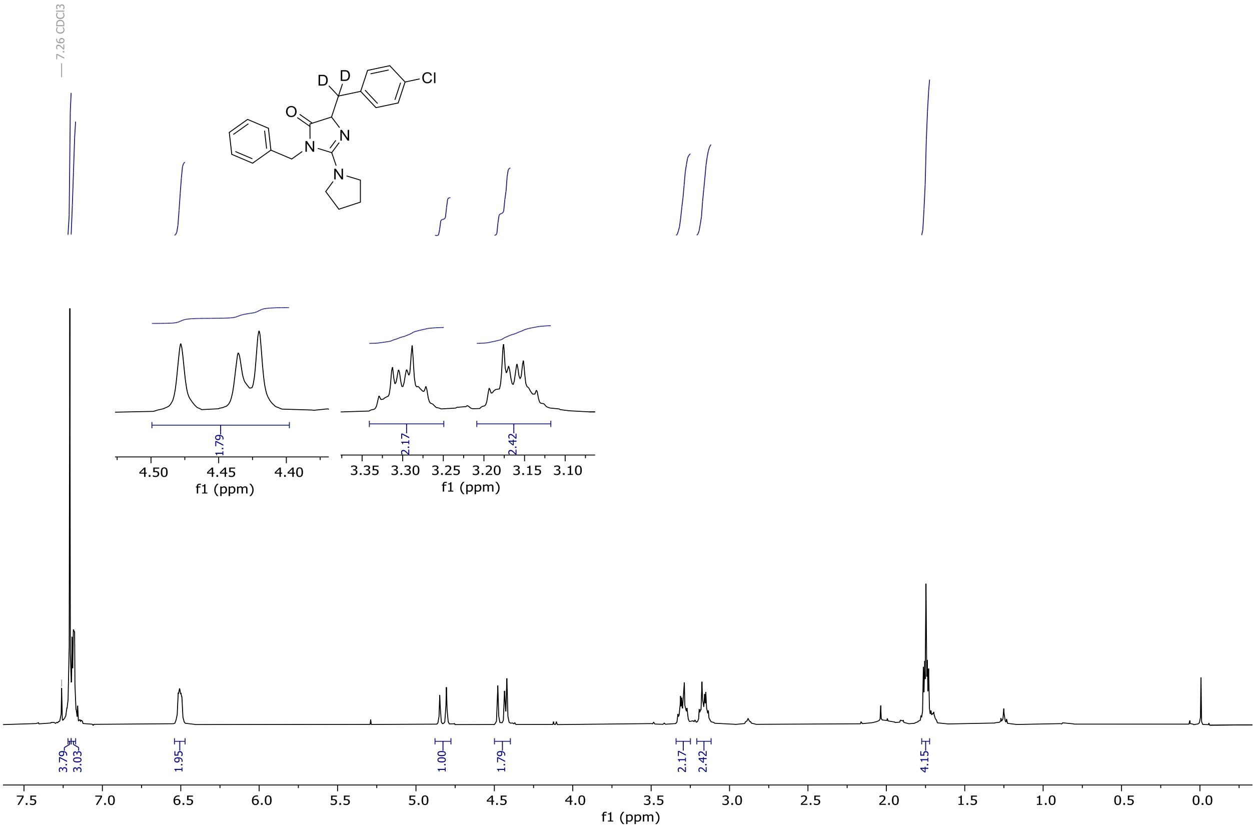

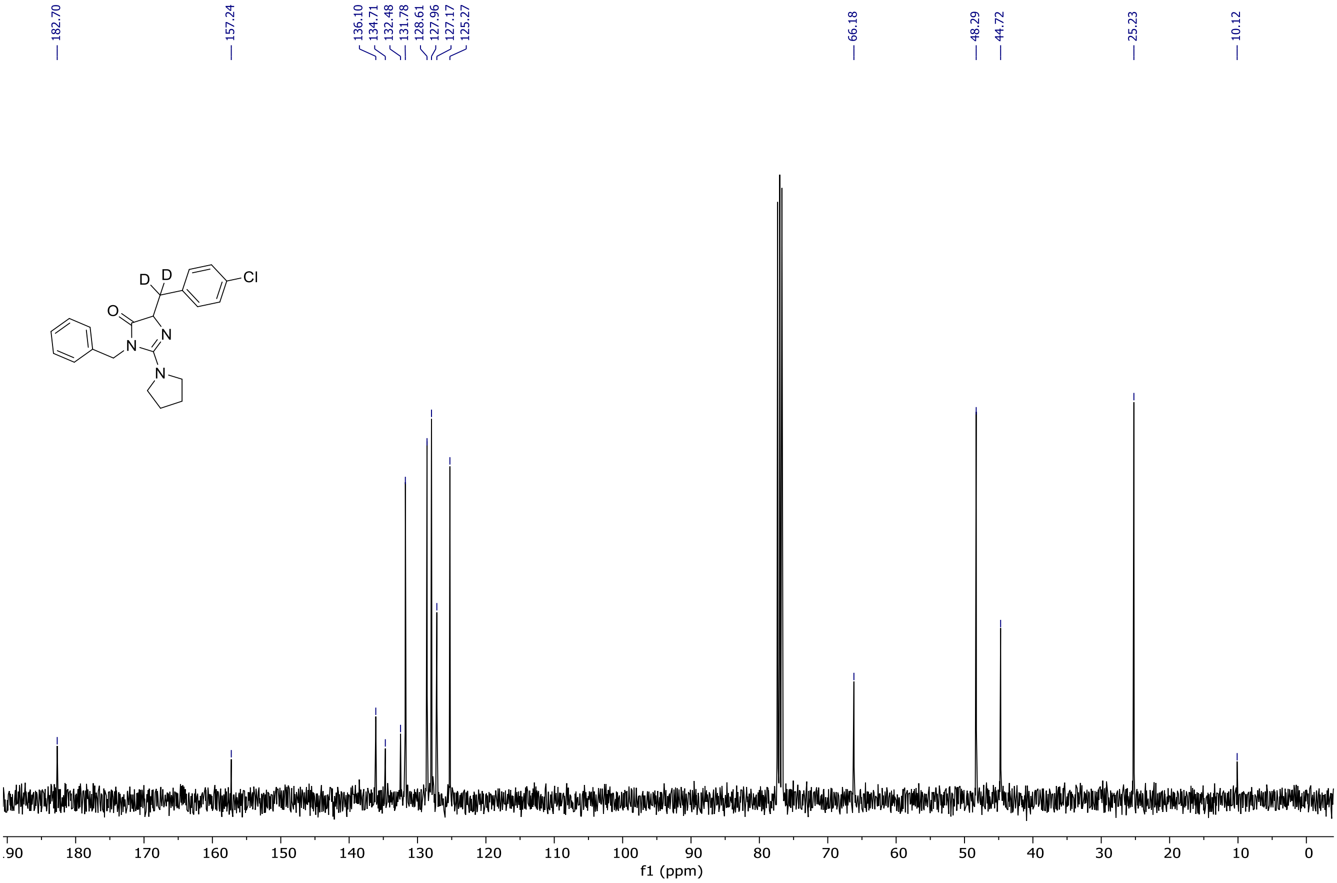

5-(Benzo[*d*][1,3]dioxol-5-ylmethyl)-3-methyl-2-(pyrrolidin-1-yl)-3,5-dihydro-4*H*-imidazol-4-one (11e)

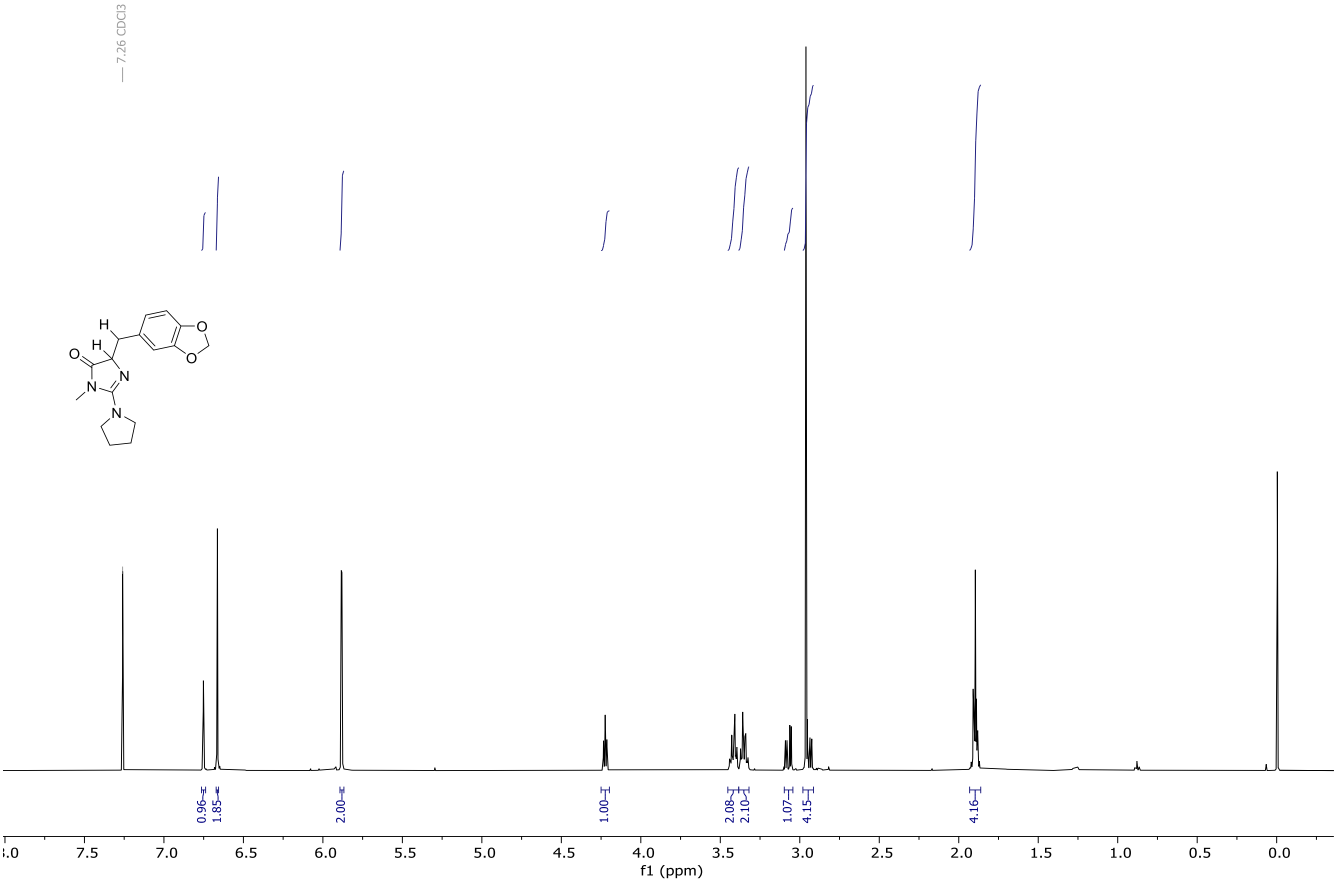

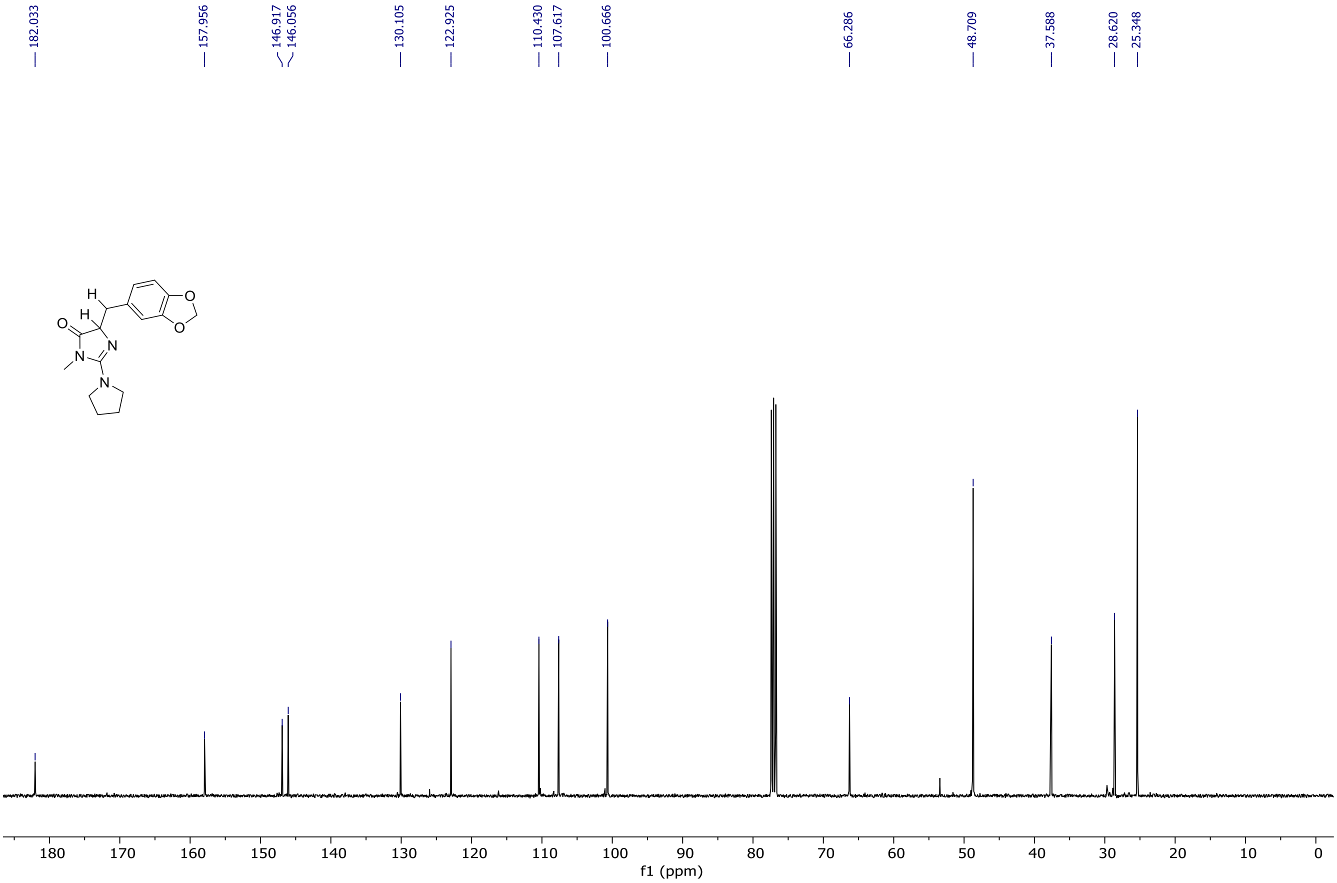

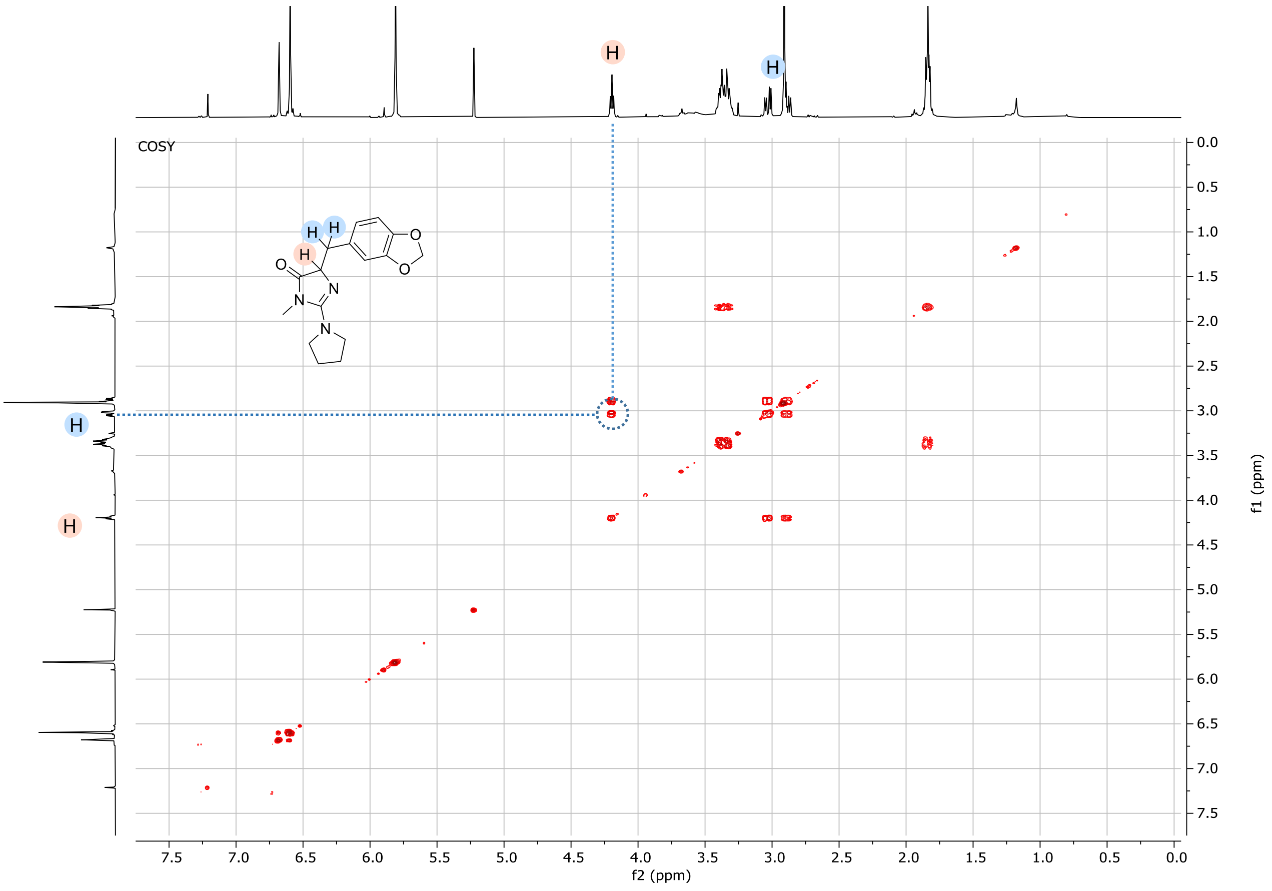
COSY
H
H
f2 (ppm)
f1 (ppm)

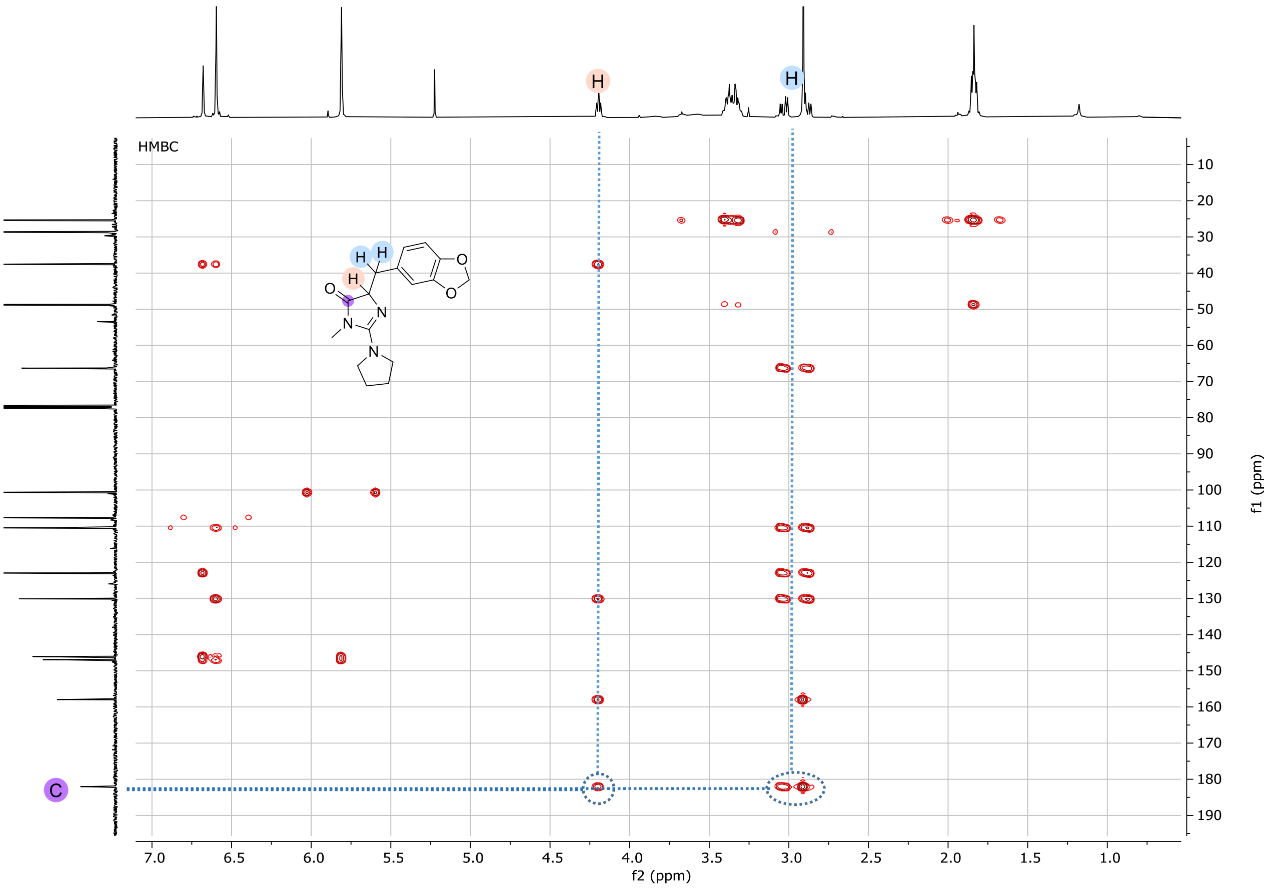
HMBC
H
H
C
f2 (ppm)
f1 (ppm)

tert-Butyl 4-(4-(benzo[_d_][1,3]dioxol-5-ylmethyl)-1-methyl-5-oxo-4,5-dihydro-1_H_-imidazol-2-yl)piperazine-1-carboxylate (11j)

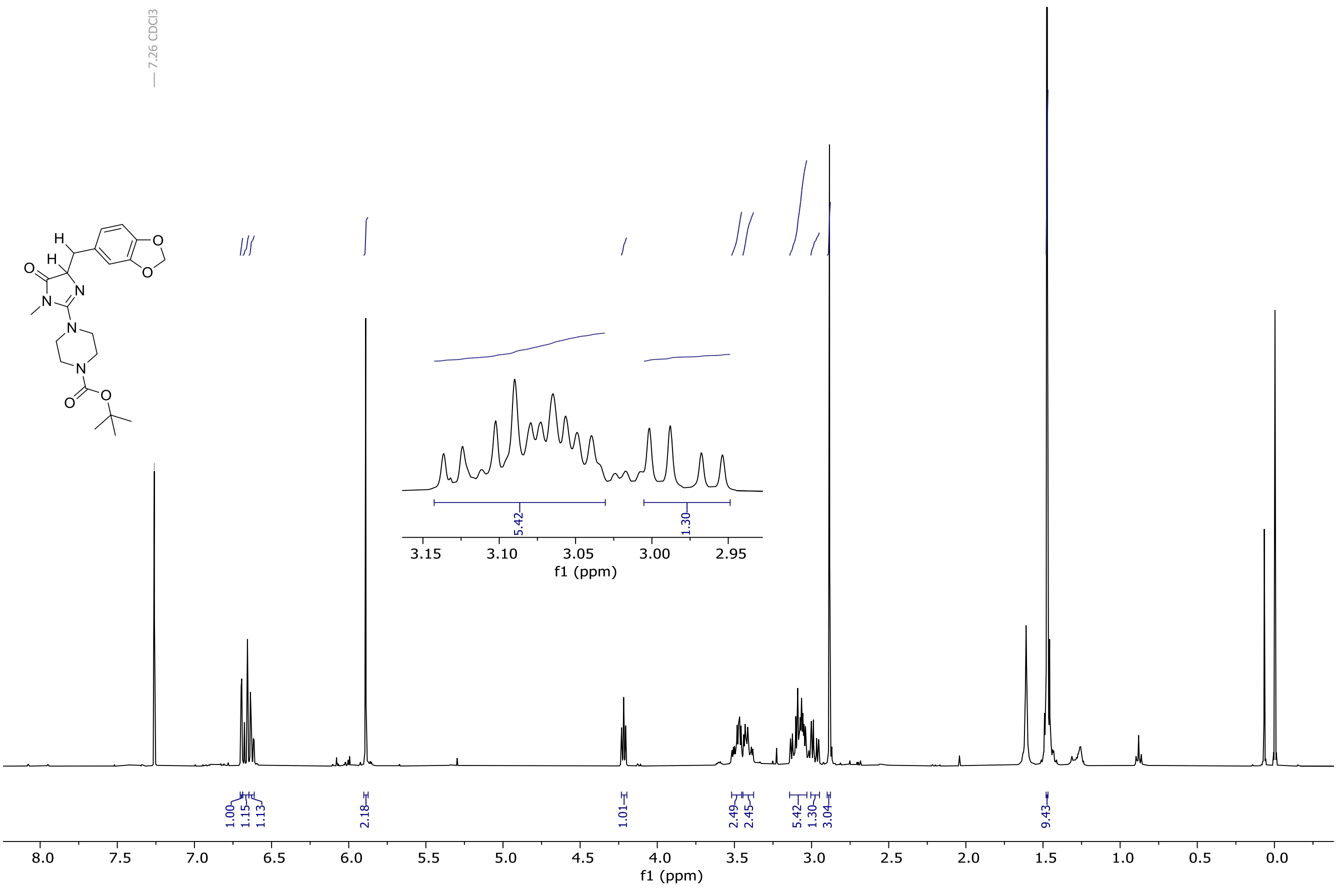

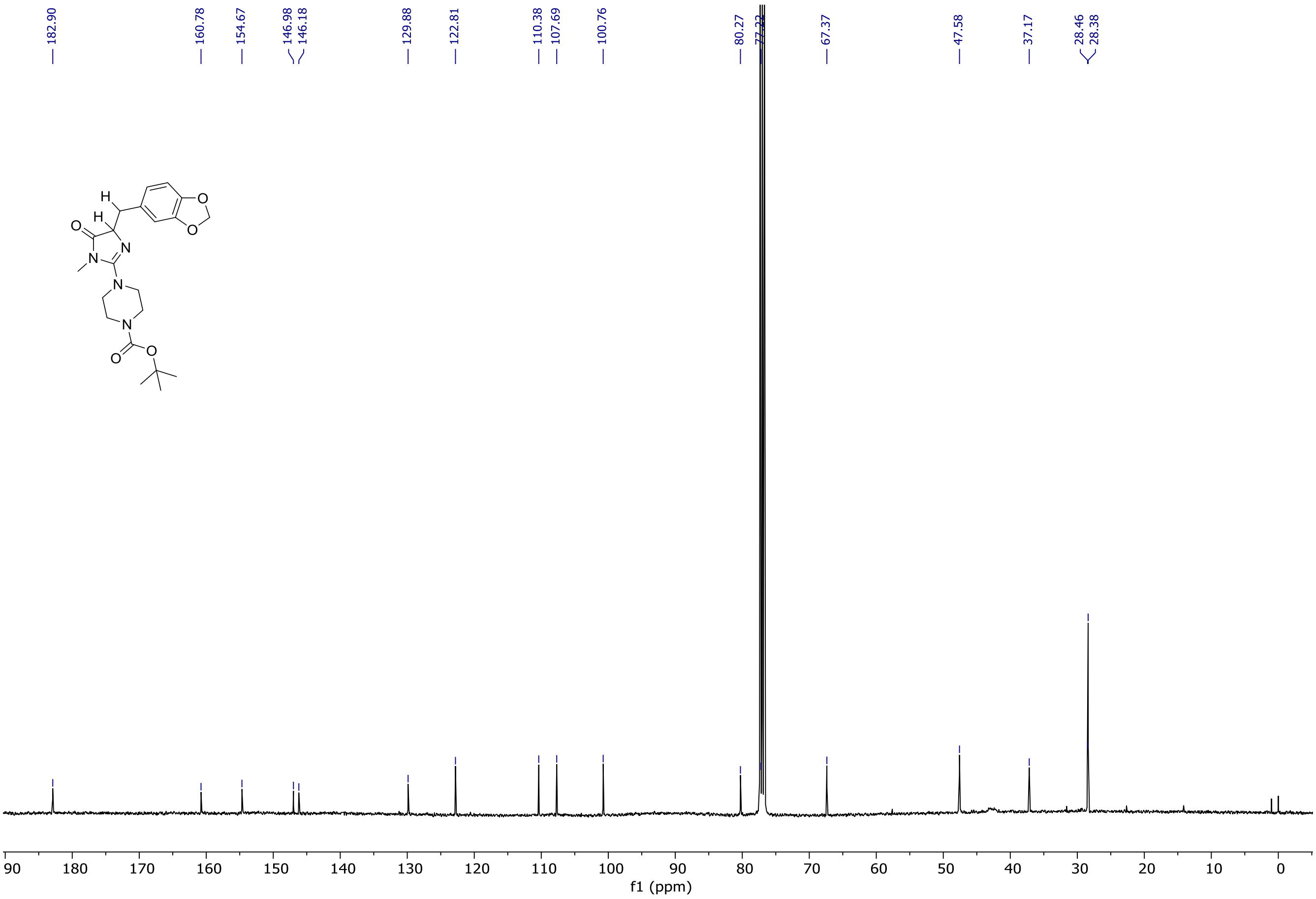

8.8. Compounds 12-13

(*Z*)-2-(benzo[*d*][1,3]dioxol-5-ylmethylene)-5,6,7,8-tetrahydroimidazo[1,2-*a*]pyrimidin-3(2*H*)-one (12a)

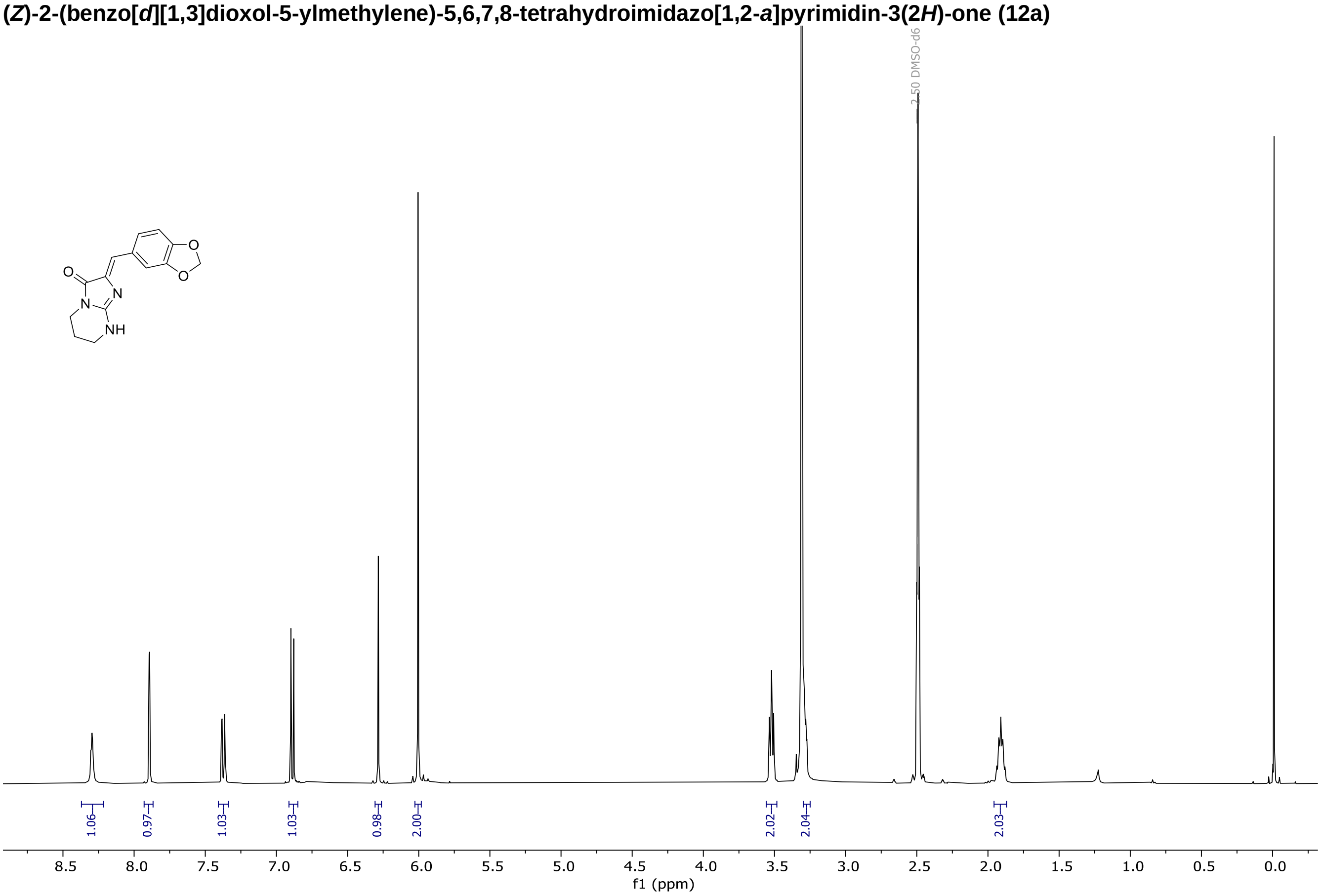

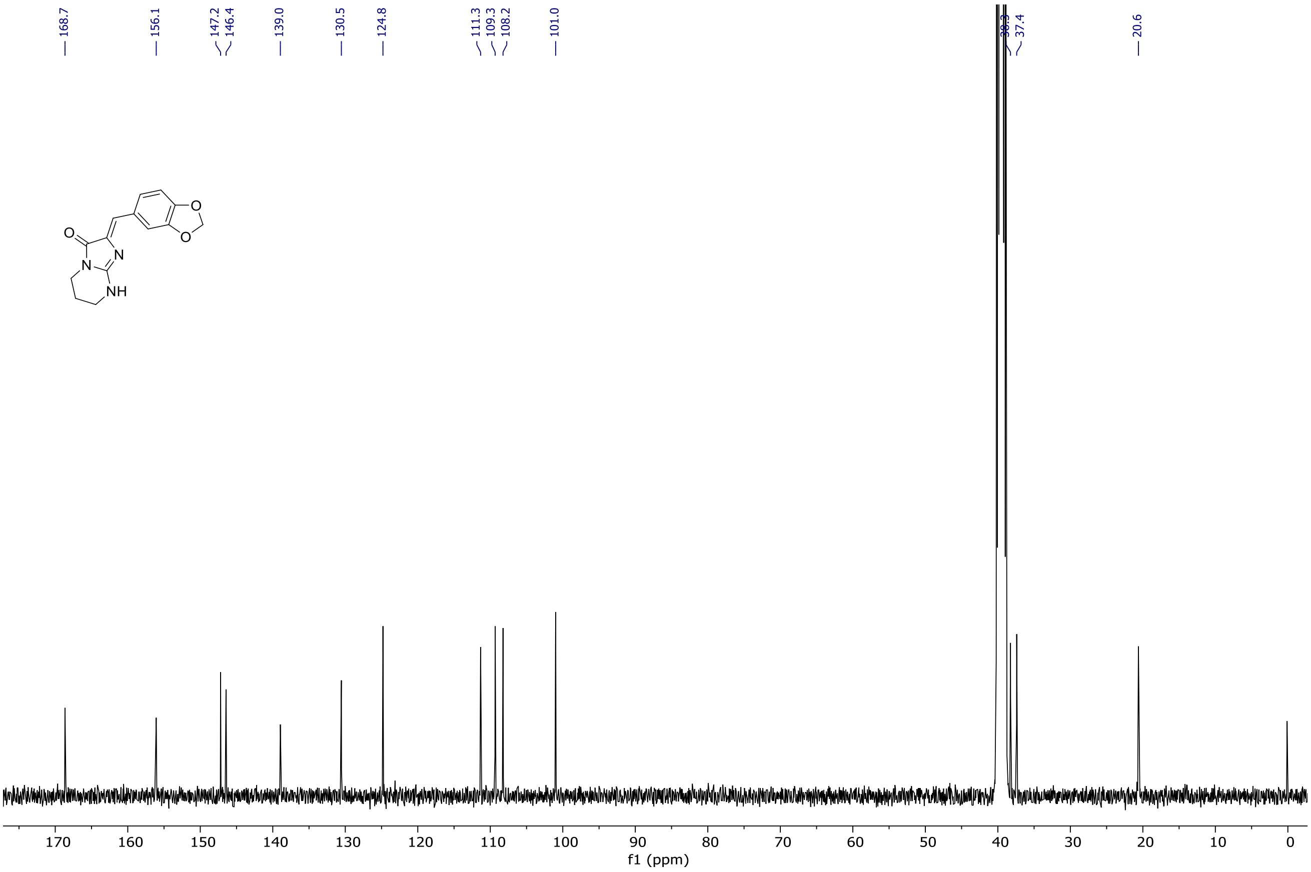

(*Z*)-5-(Benzo[*d*][1,3]dioxol-5-ylmethylene)-3-methyl-2-(methylamino)-3,5-dihydro-4*H*-imidazol-4-one (12b)

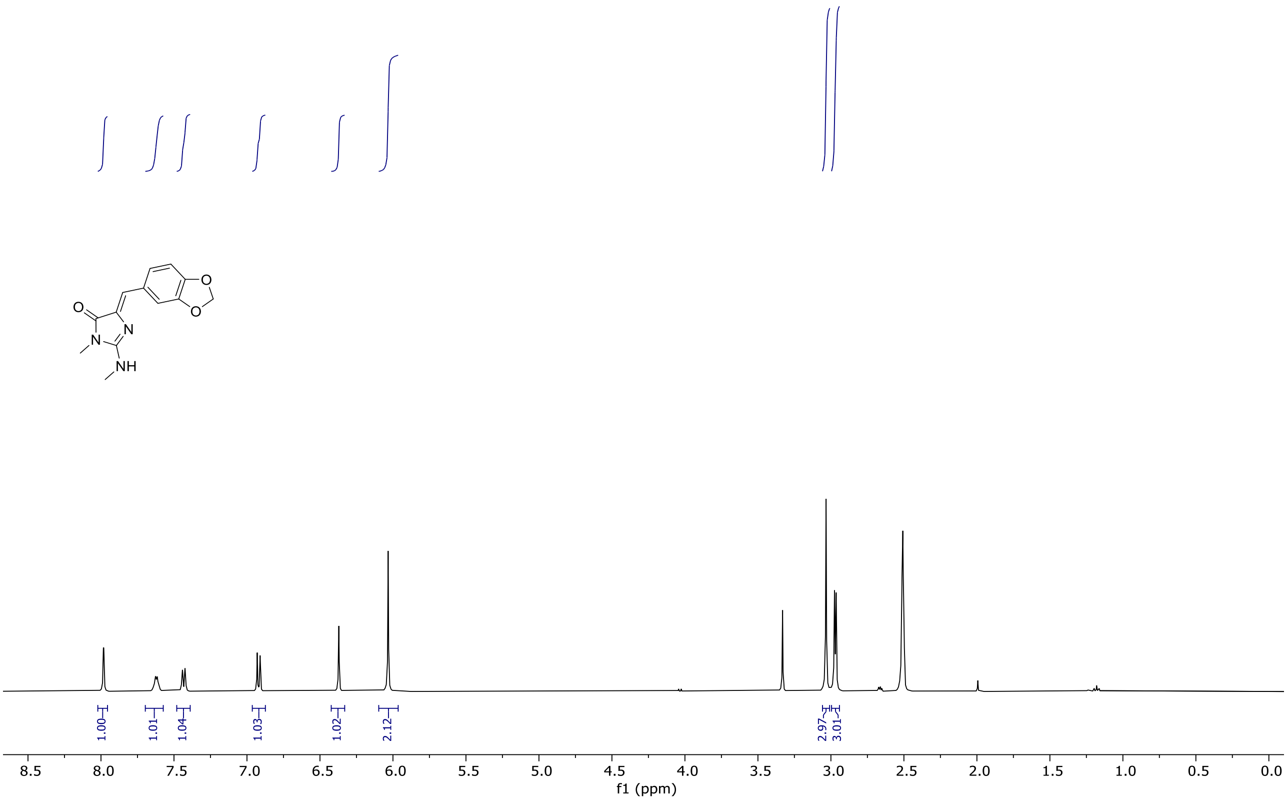

tert-Butyl (Z)-3-((1-methyl-2-(methylamino)-5-oxo-1,5-dihydro-4H-imidazol-4-ylidene)methyl)-1H-indole-1-carboxylate (12c)

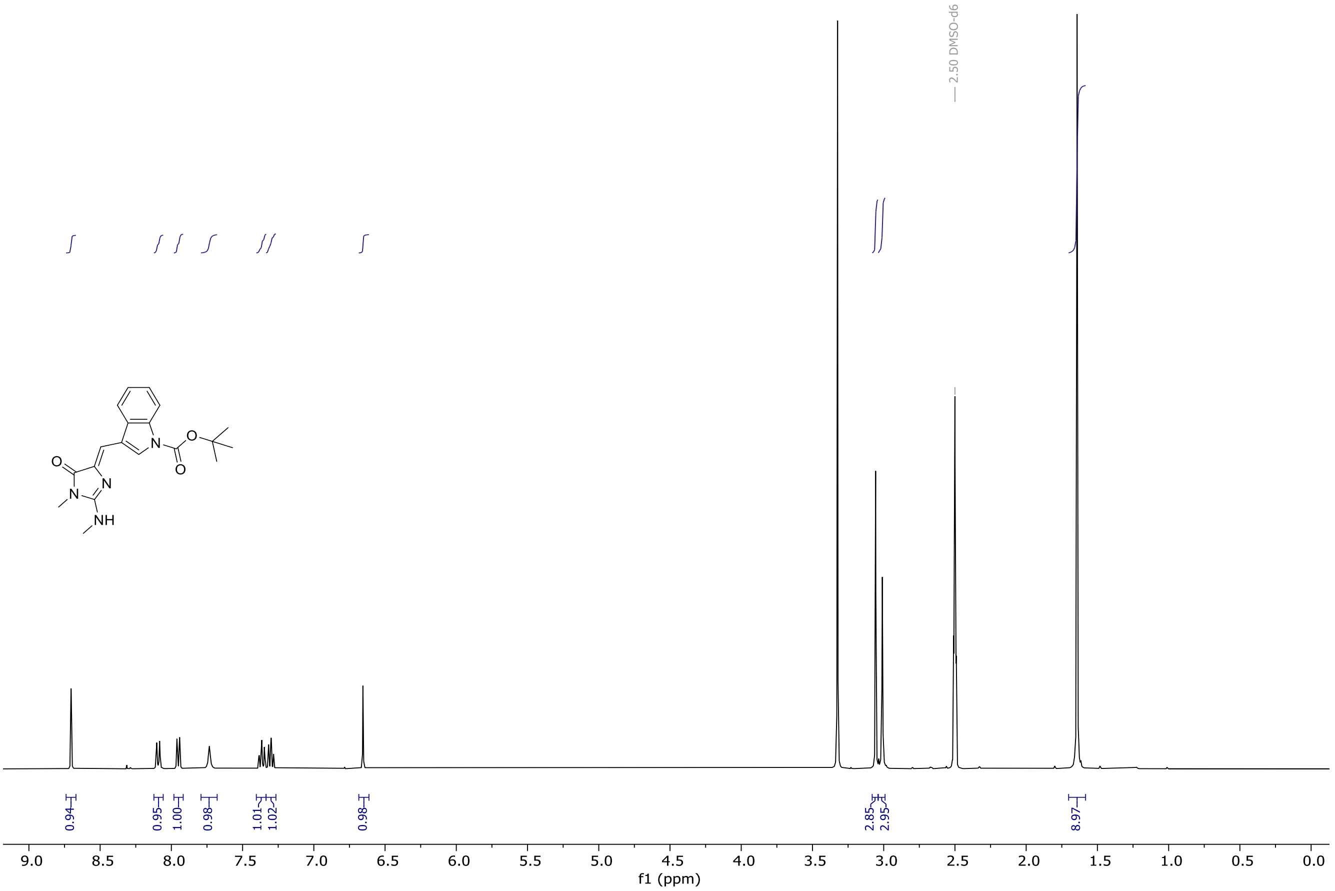

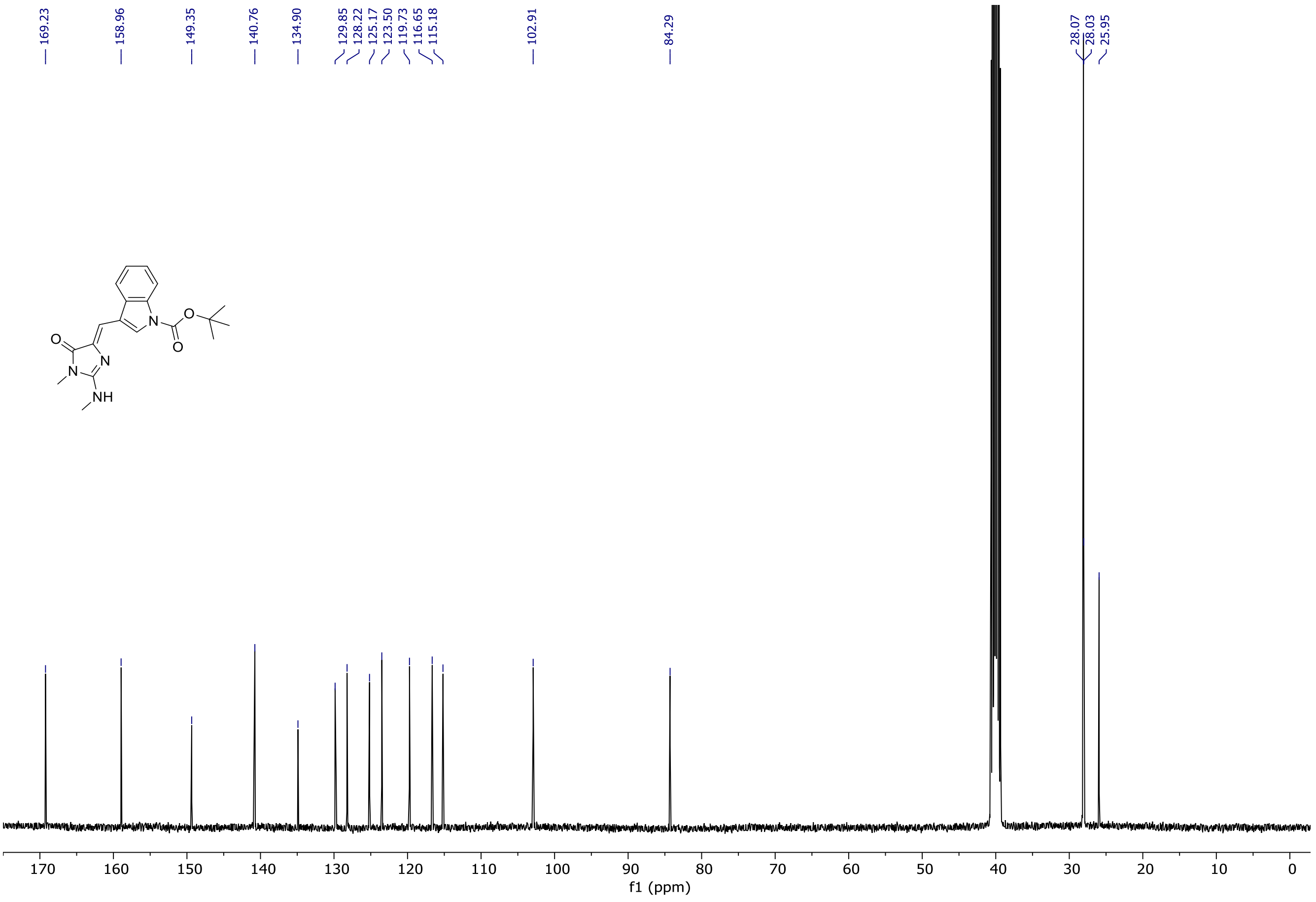

(*Z*)-5-((1*H*-Indol-3-yl)methylene)-3-methyl-2-(methylamino)-3,5-dihydro-4*H*-imidazol-4-one (12c')

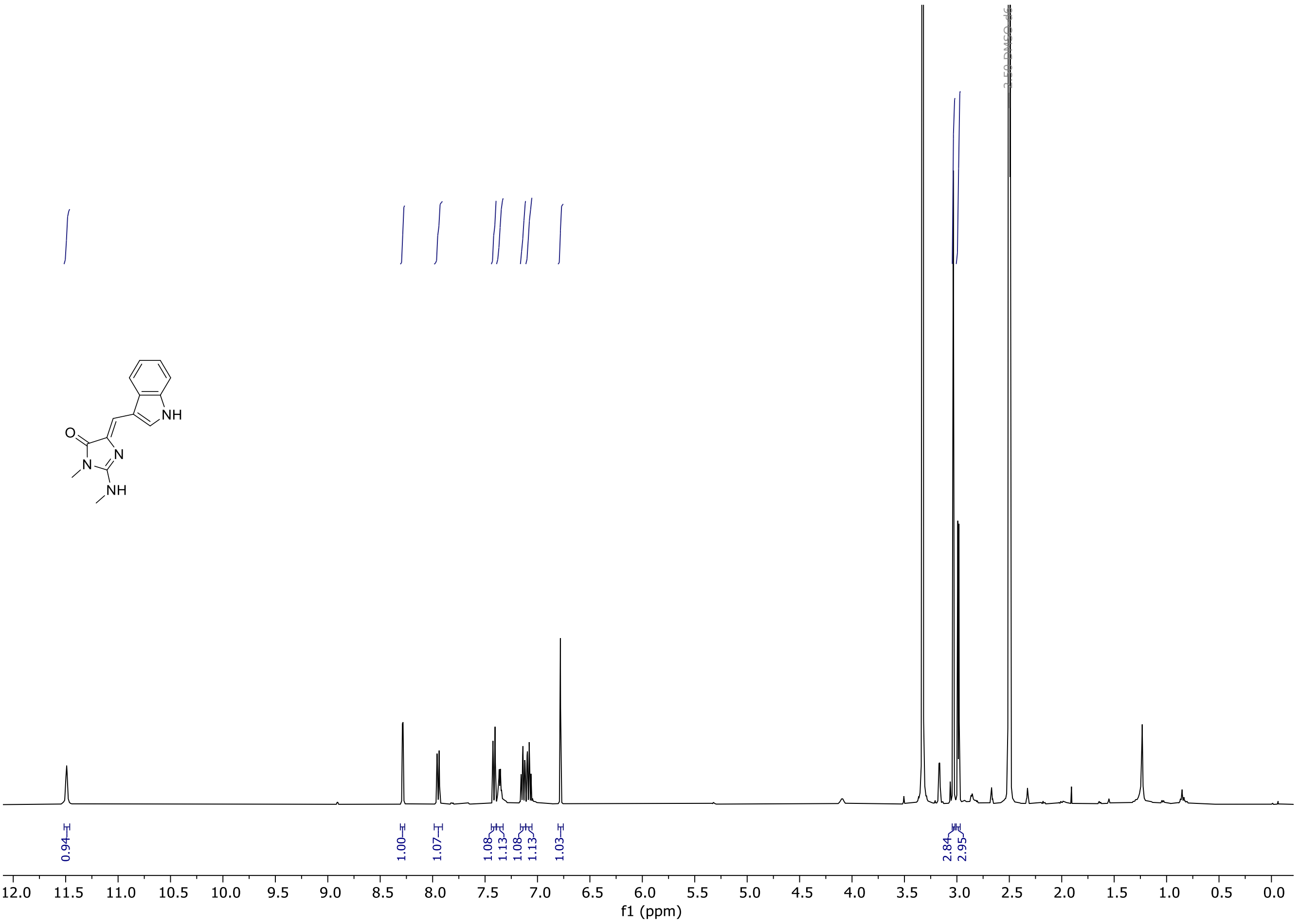

SUPPORTING INFORMATION

(*Z*)-5-(Benzo[*d*][1,3]dioxol-5-ylmethylene)-3-methyl-2-(pyrrolidin-1-yl)-3,5-dihydro-4*H*-imidazol-4-one (12d)

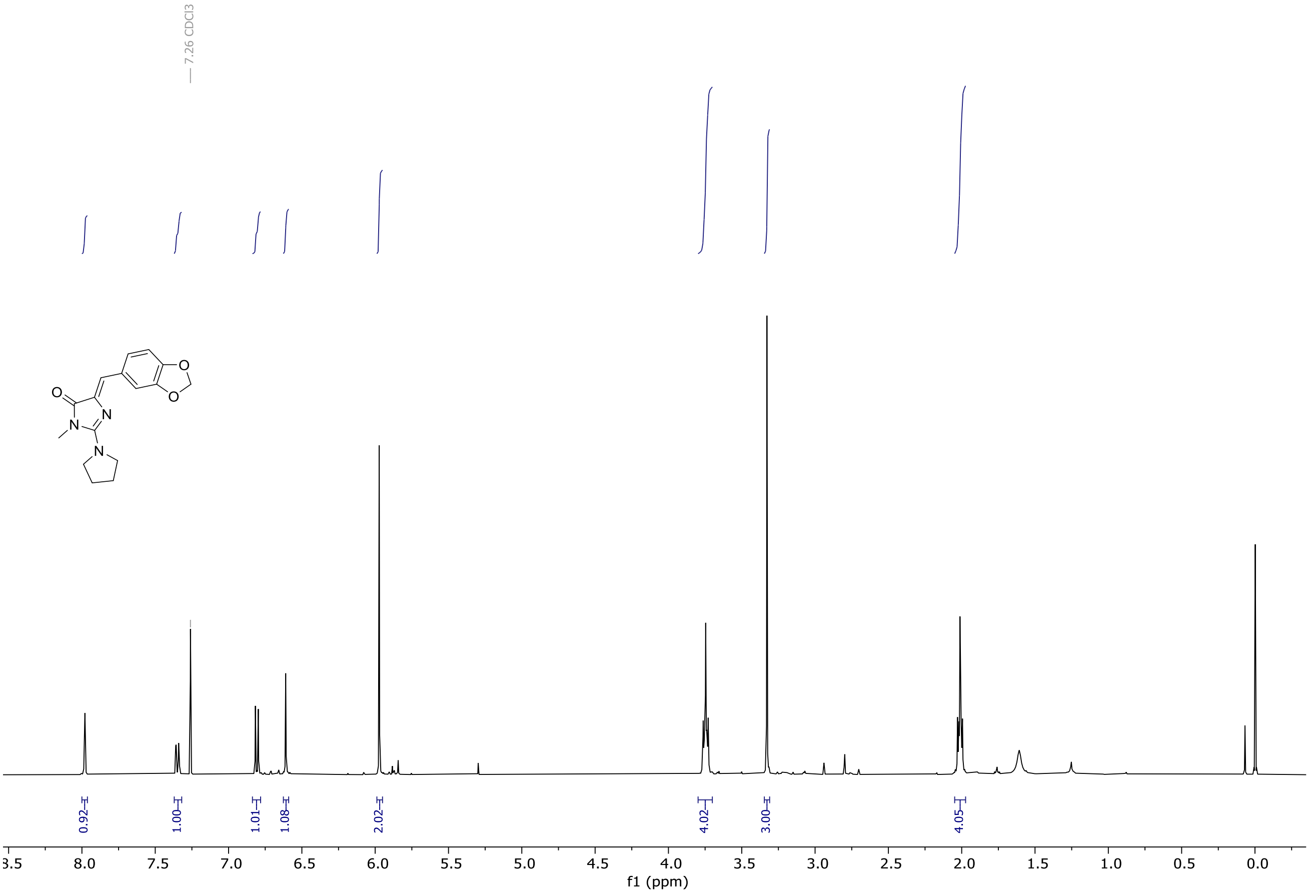

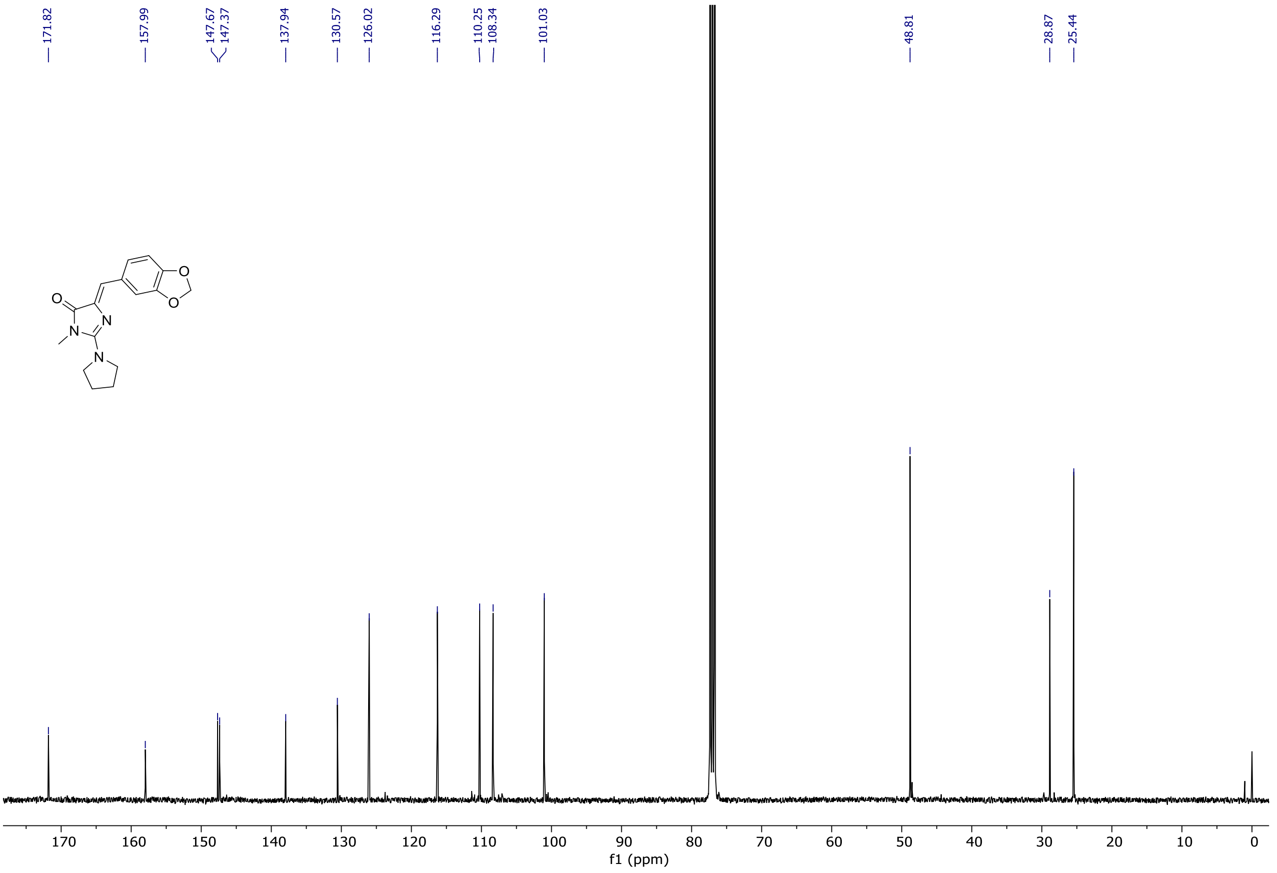

(*Z*)-3-Benzyl-5-(4-chlorobenzylidene)-2-(pyrrolidin-1-yl)-3,5-dihydro-4*H*-imidazol-4-one (12e)

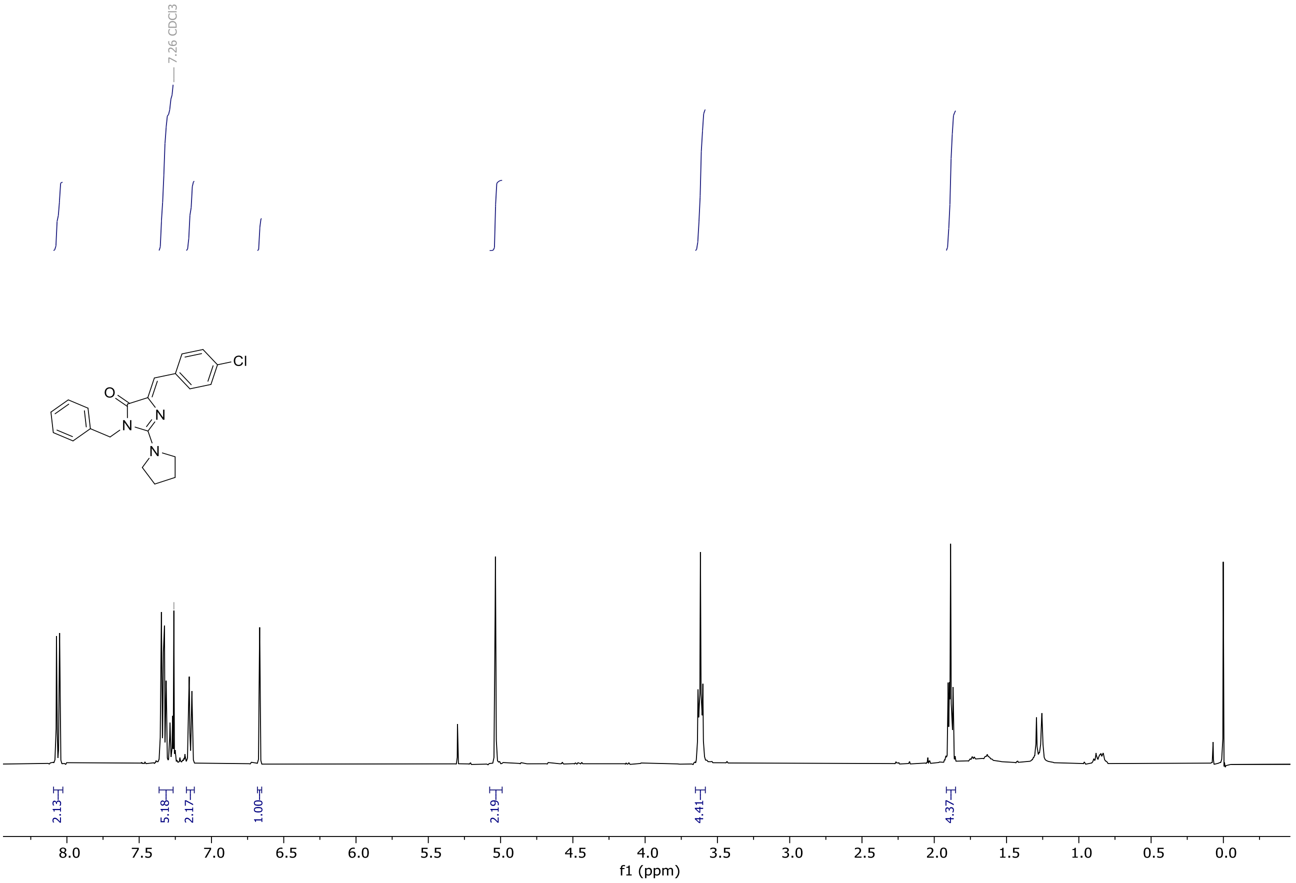

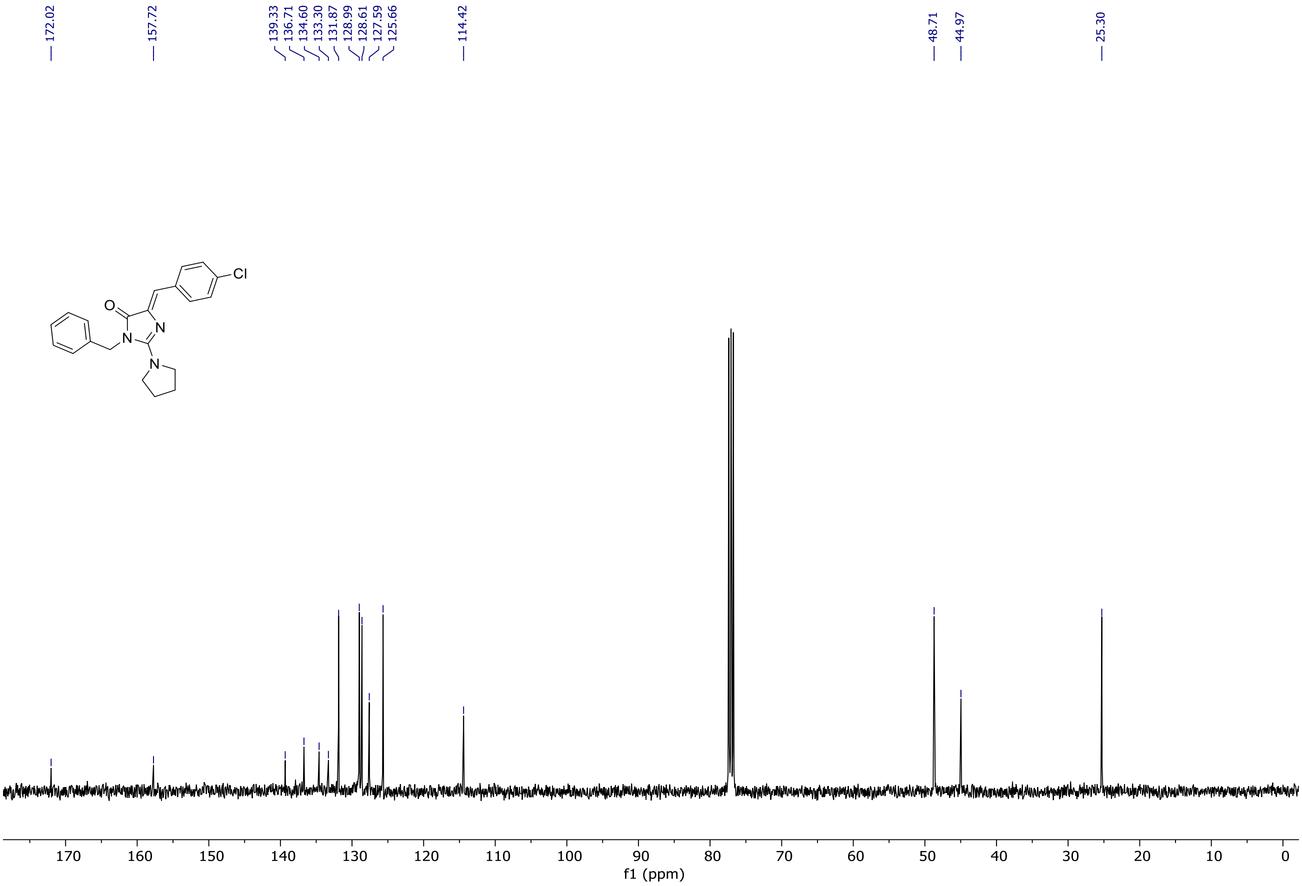

(*Z*)-5-(Benzo[*d*][1,3]dioxol-5-ylmethylene)-3-methyl-2-(4-methylpiperazin-1-yl)-3,5-dihydro-4*H*-imidazol-4-one (12f)

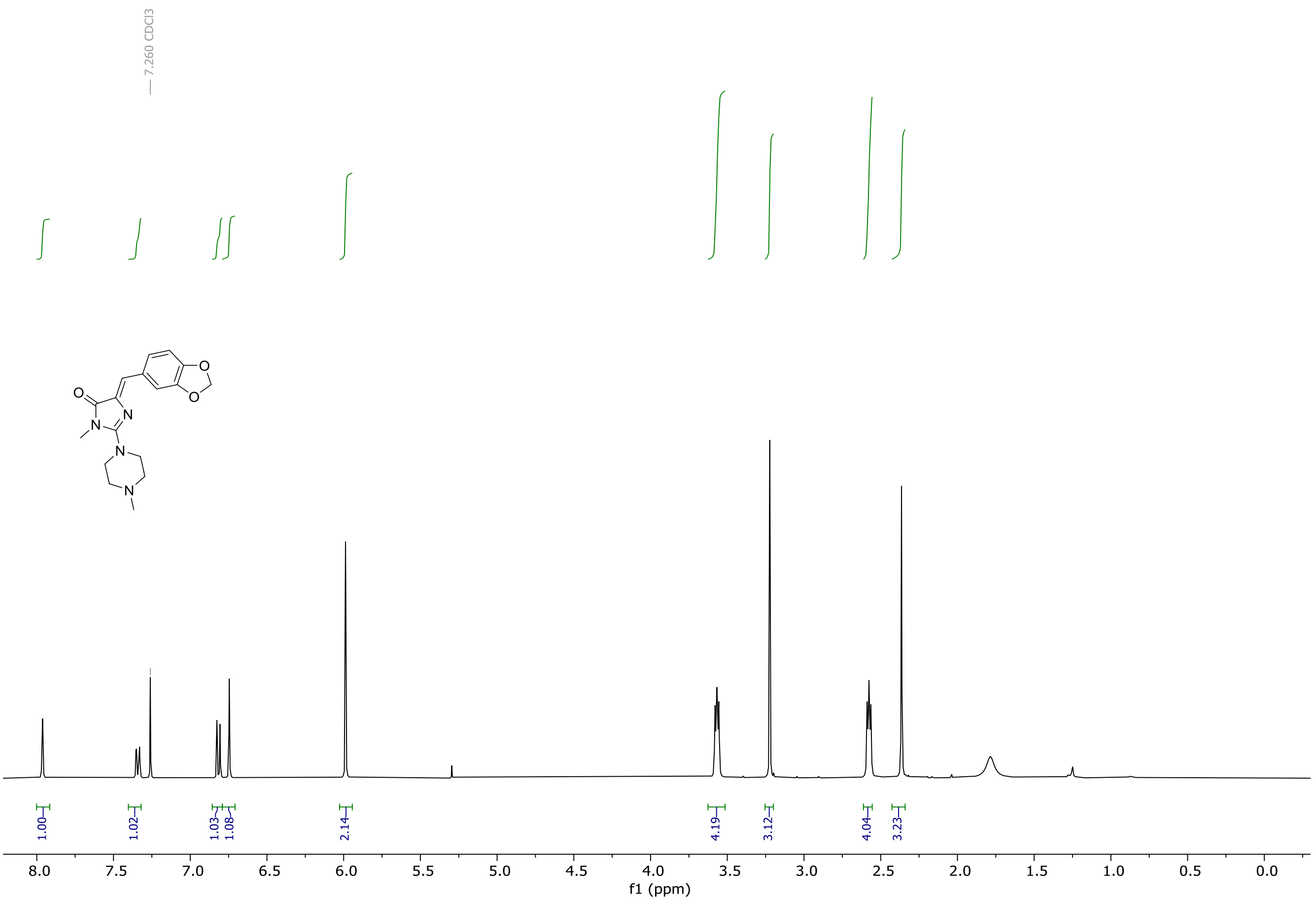

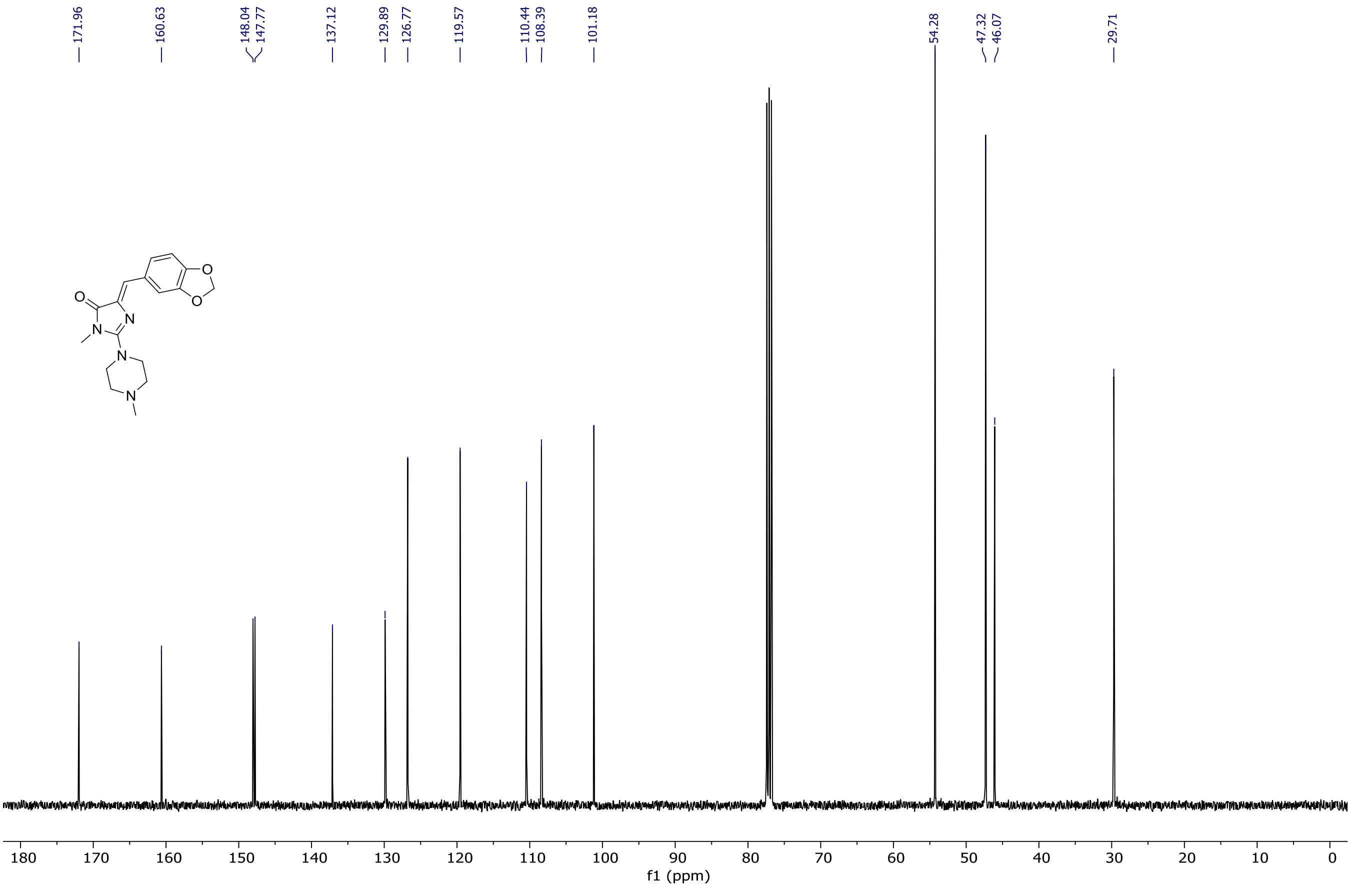

(*Z*)-3-Benzyl-5-(4-methoxybenzylidene)-2-morpholino-3,5-dihydro-4*H*-imidazol-4-one (12g)

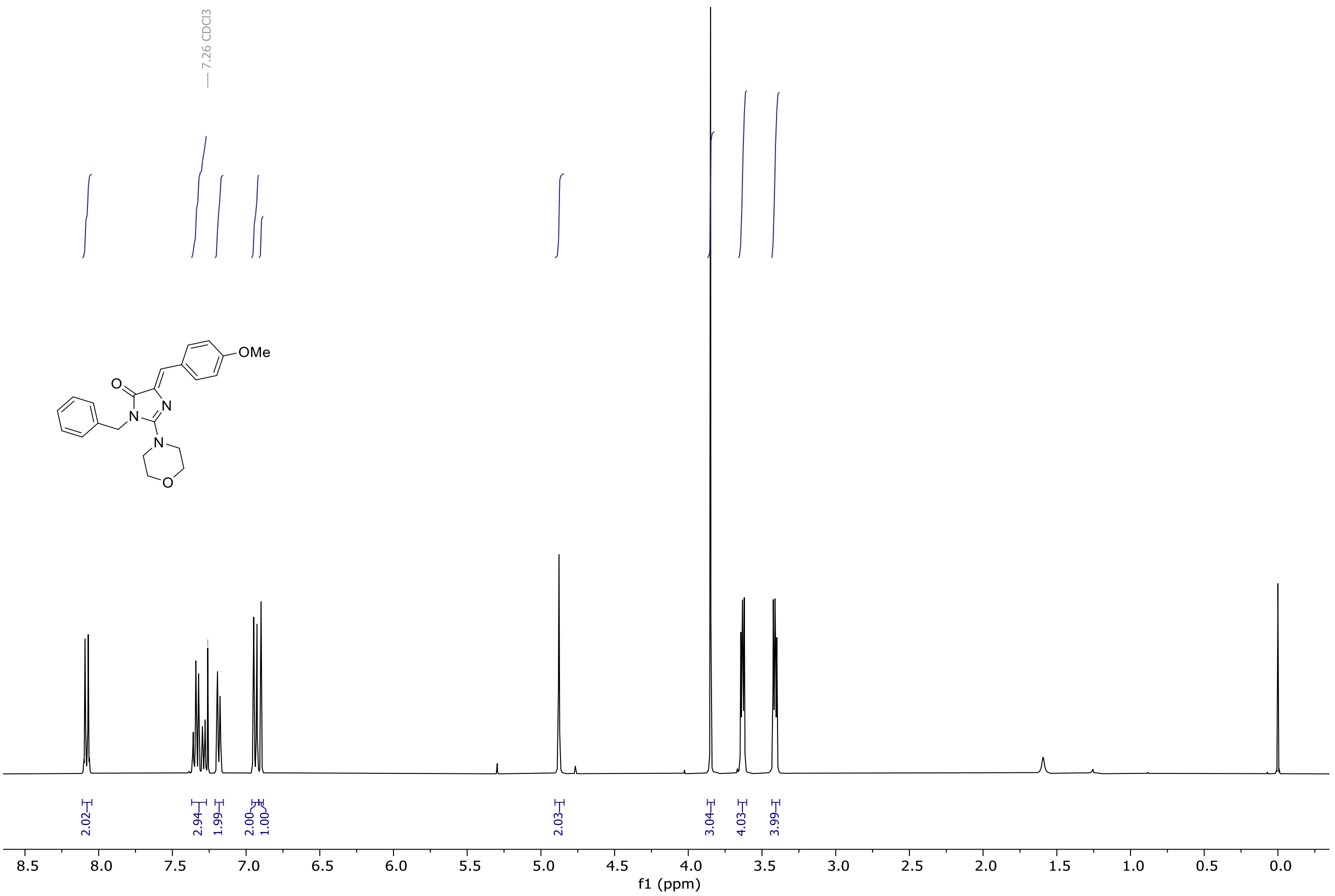

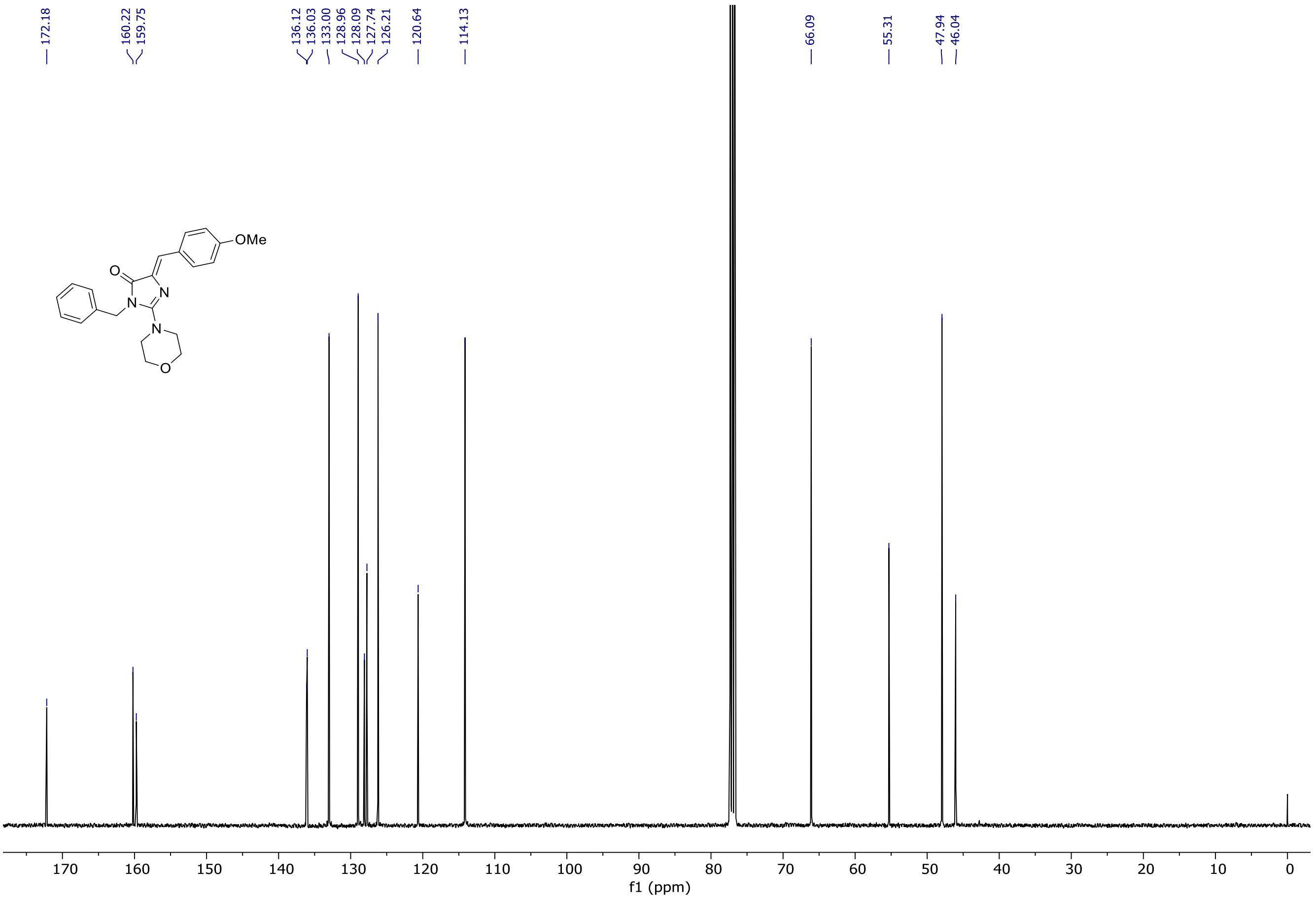

(*Z*)-5-(Benzo[*d*][1,3]dioxol-5-ylmethylene)-3-methyl-2-(pentylamino)-3,5-dihydro-4*H*-imidazol-4-oneone (12h)

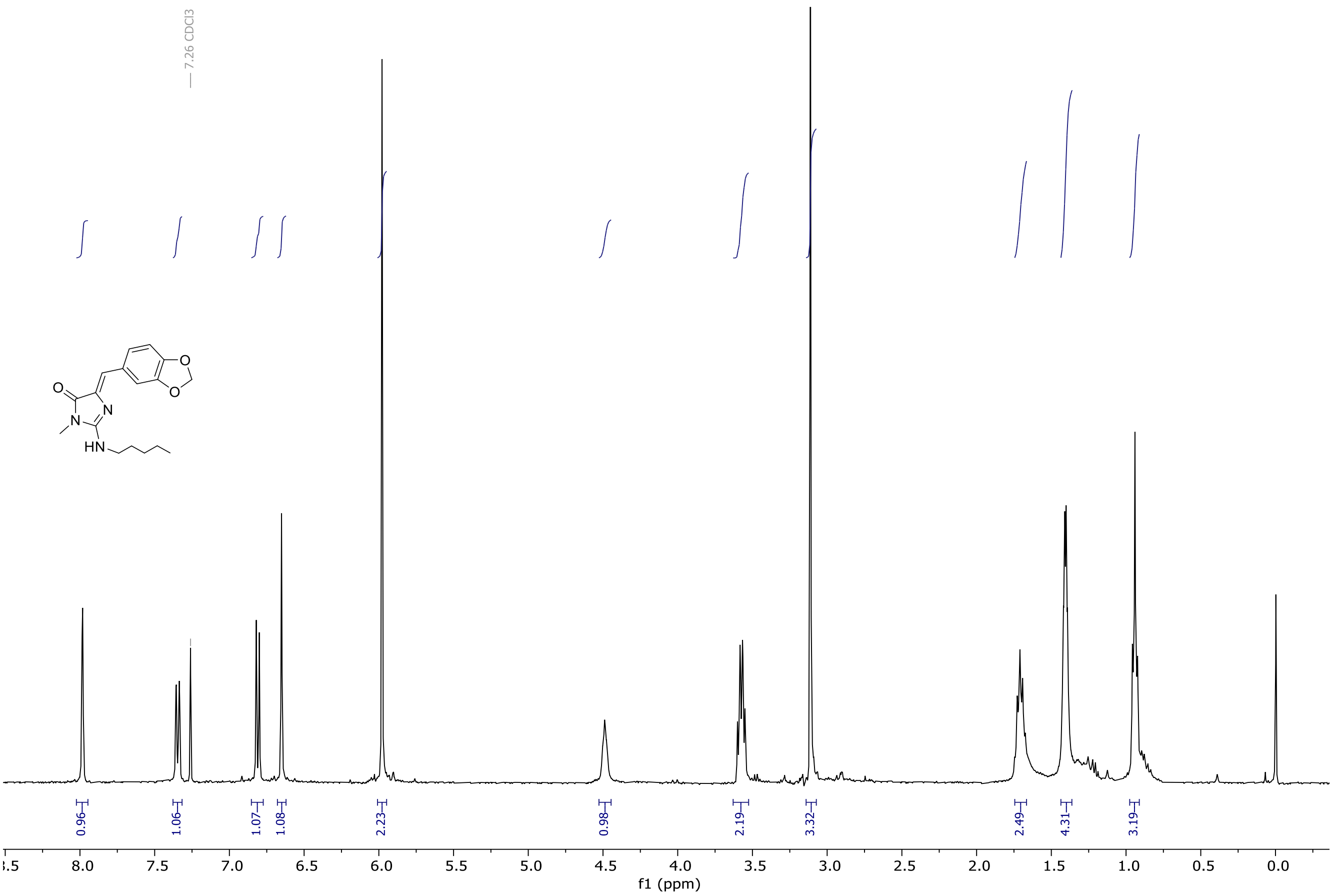

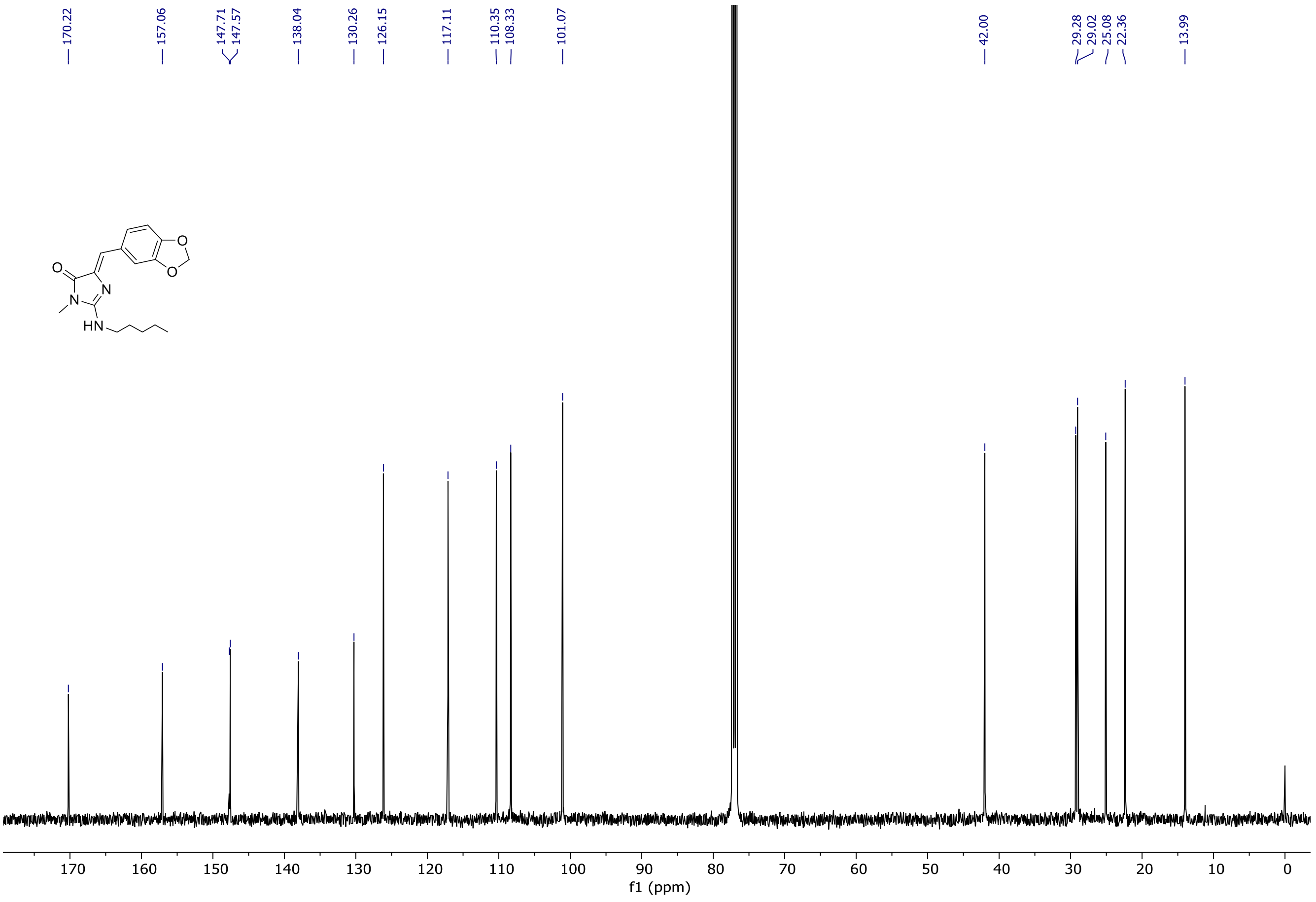

(*Z*)-2-Amino-5-(benzo[*d*][1,3]dioxol-5-ylmethylene)-3-methyl-3,5-dihydro-4*H*-imidazol-4-one (12i)

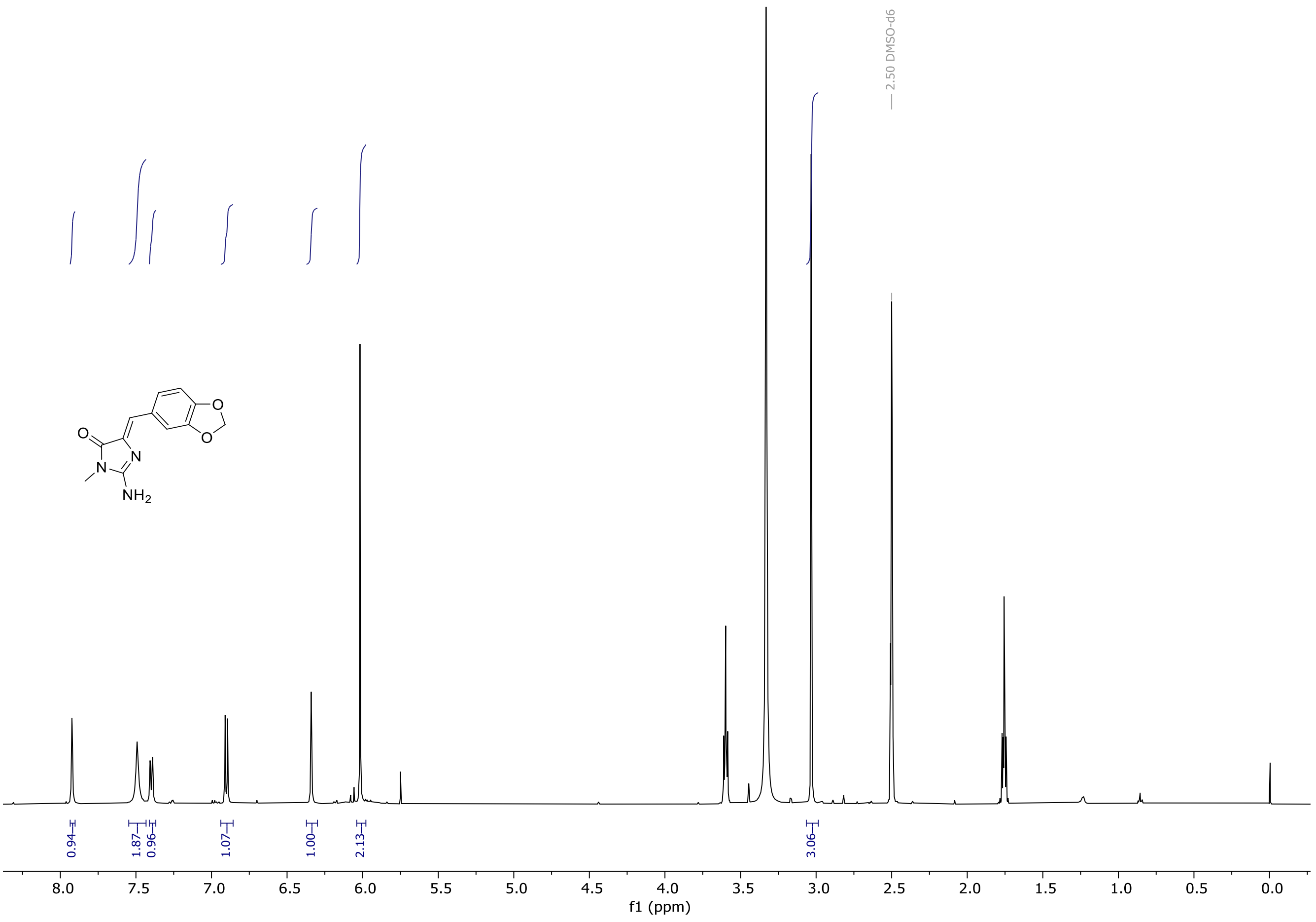

(*Z*)-5-(Benzo[*d*][1,3]dioxol-5-ylmethylene)-3-methyl-2-(piperazin-1-yl)-3,5-dihydro-4*H*-imidazol-4-one (12j)

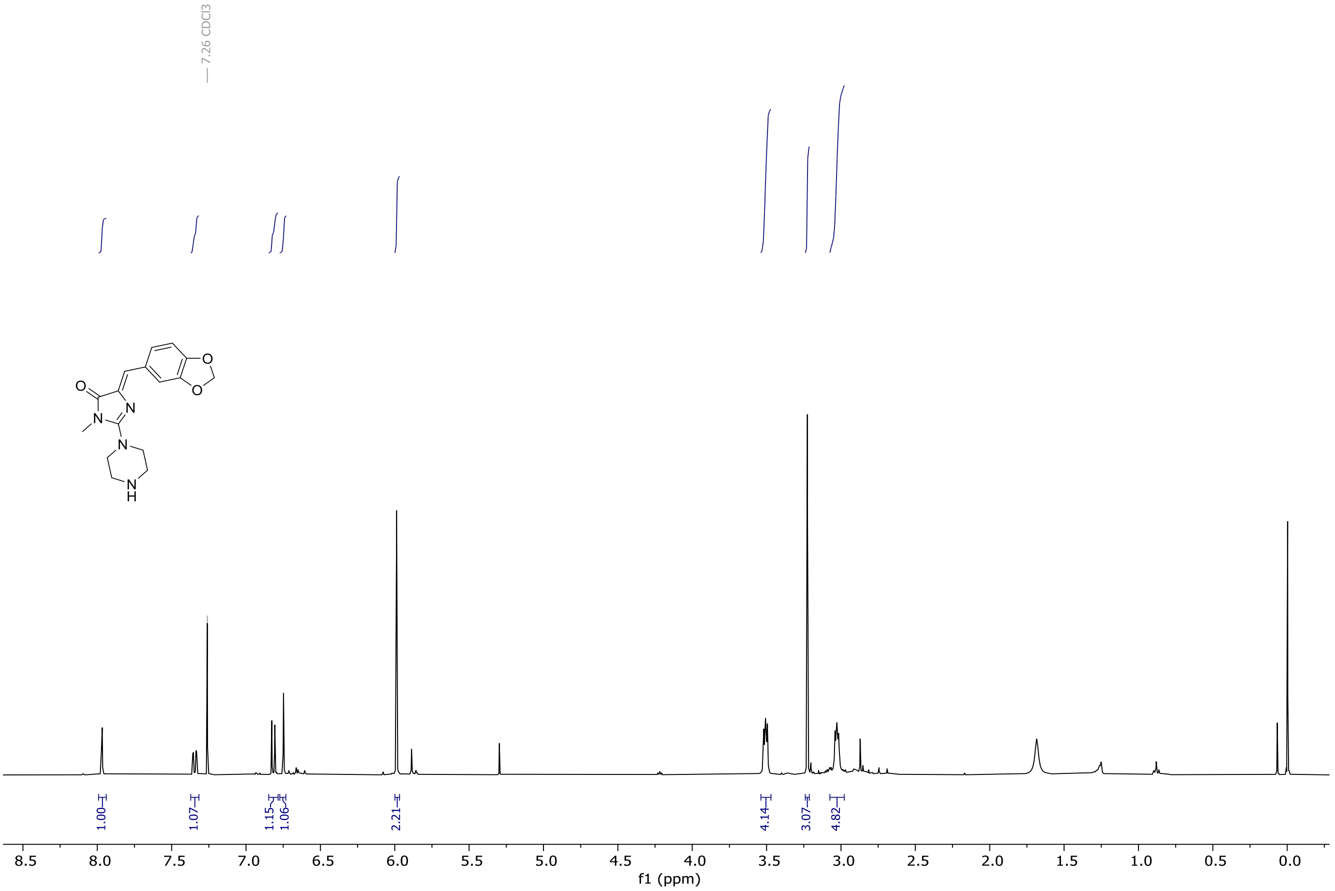

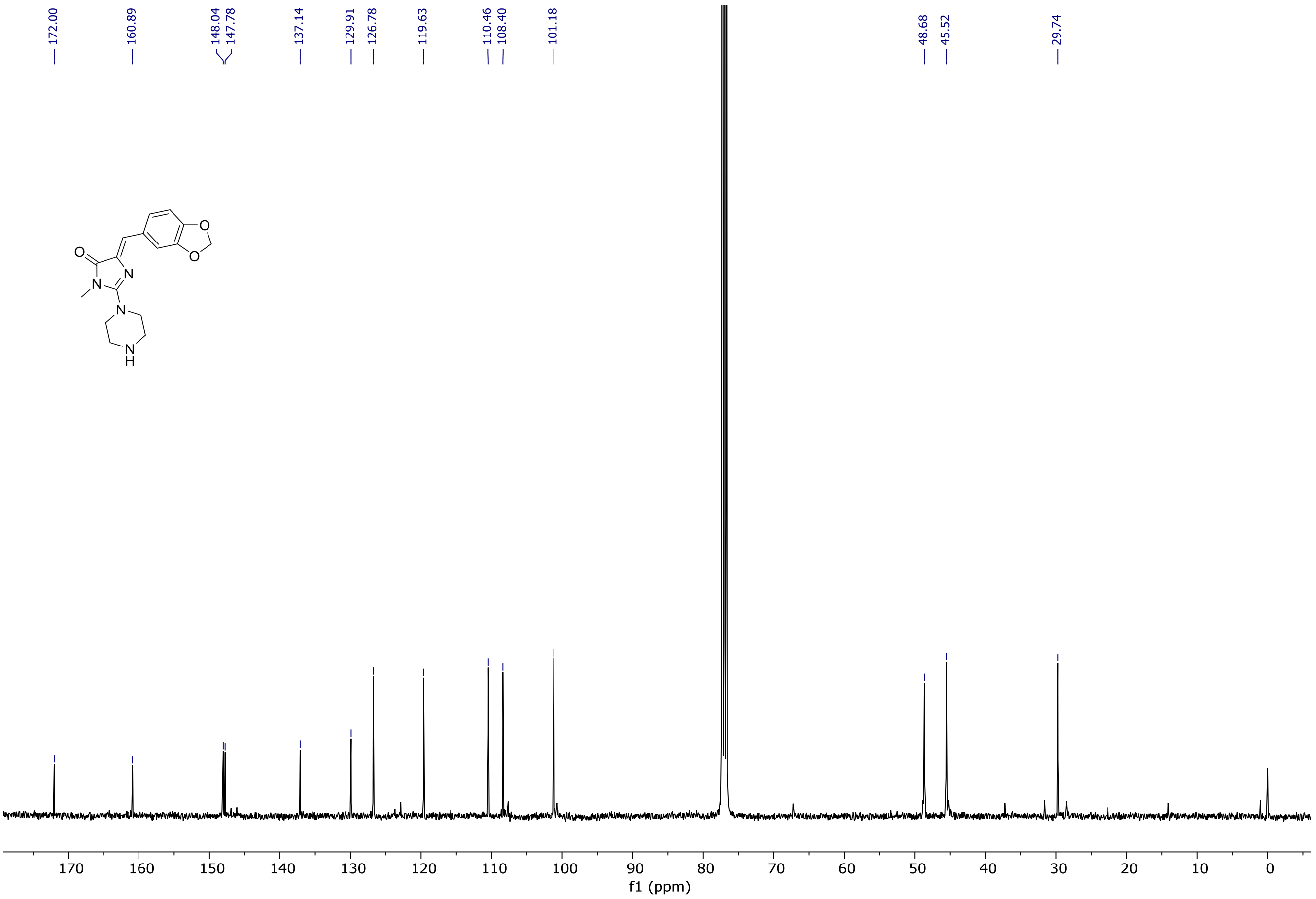

(*Z*)-5-(Benzo[*d*][1,3]dioxol-5-ylmethylene)-3-methyl-2-(propylthio)-3,5-dihydro-4*H*-imidazol-4-one (13)

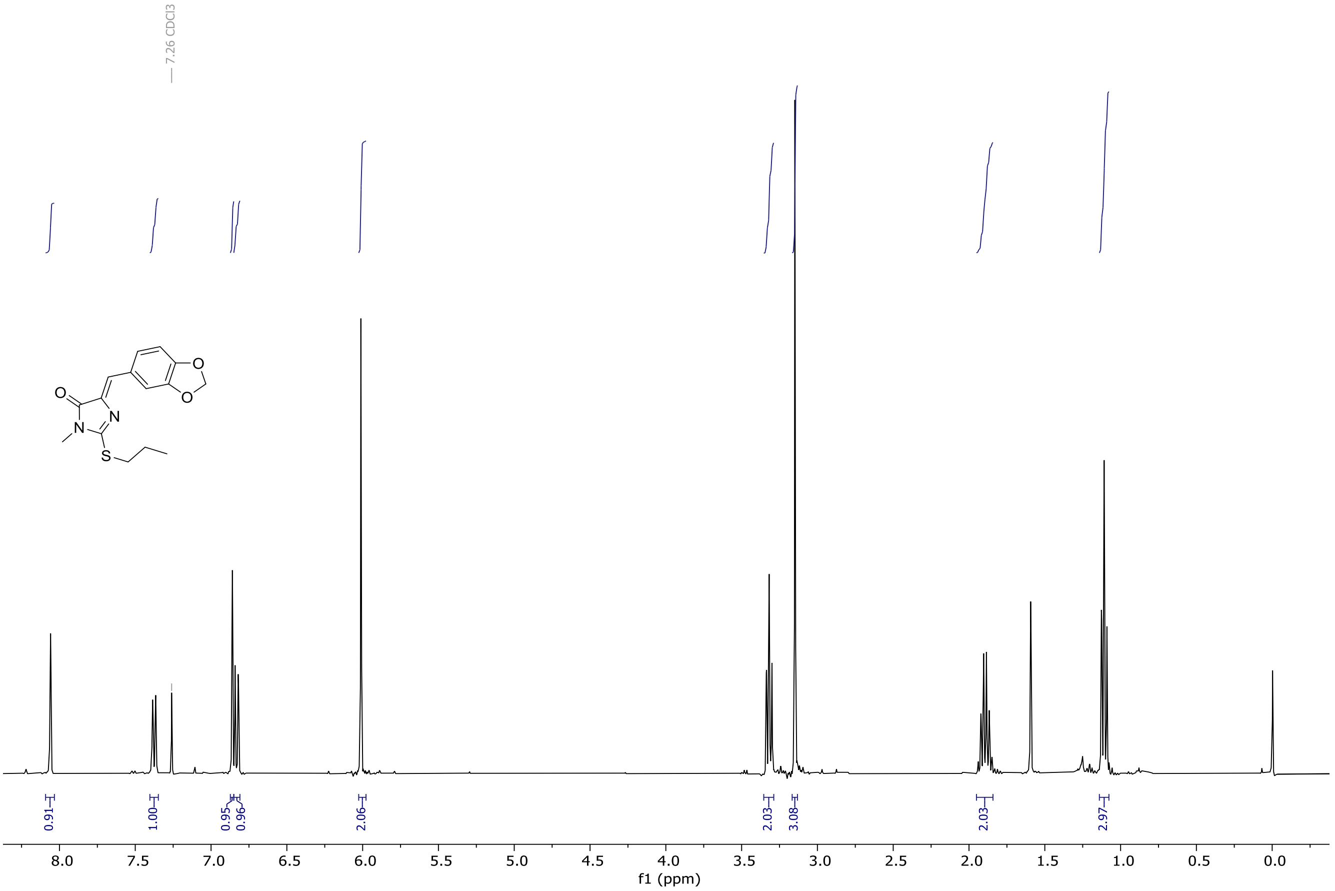

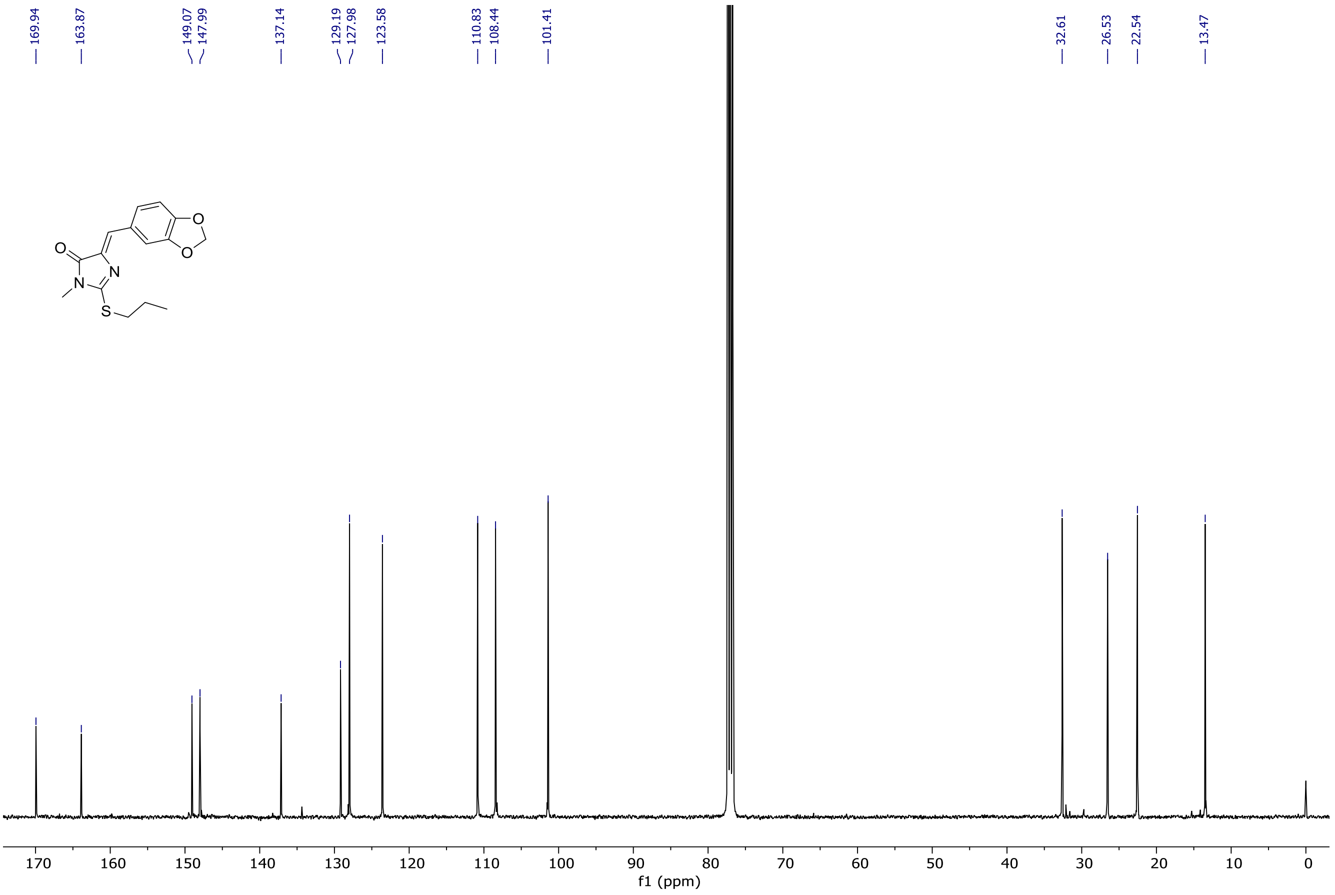

8.9. Compounds 14

(3a*S*,4*R*,6a*R*)-4-(5-(4-((*Z*)-4-(Benzo[*d*][1,3]dioxol-5-ylmethylene)-1-methyl-5-oxo-4,5-dihydro-1*H*-imidazol-2-yl)piperazin-1-yl)-5-oxopentyl)tetrahydro-1*H*-thieno[3,4-*d*]imidazol-2(3*H*)-one (14a)

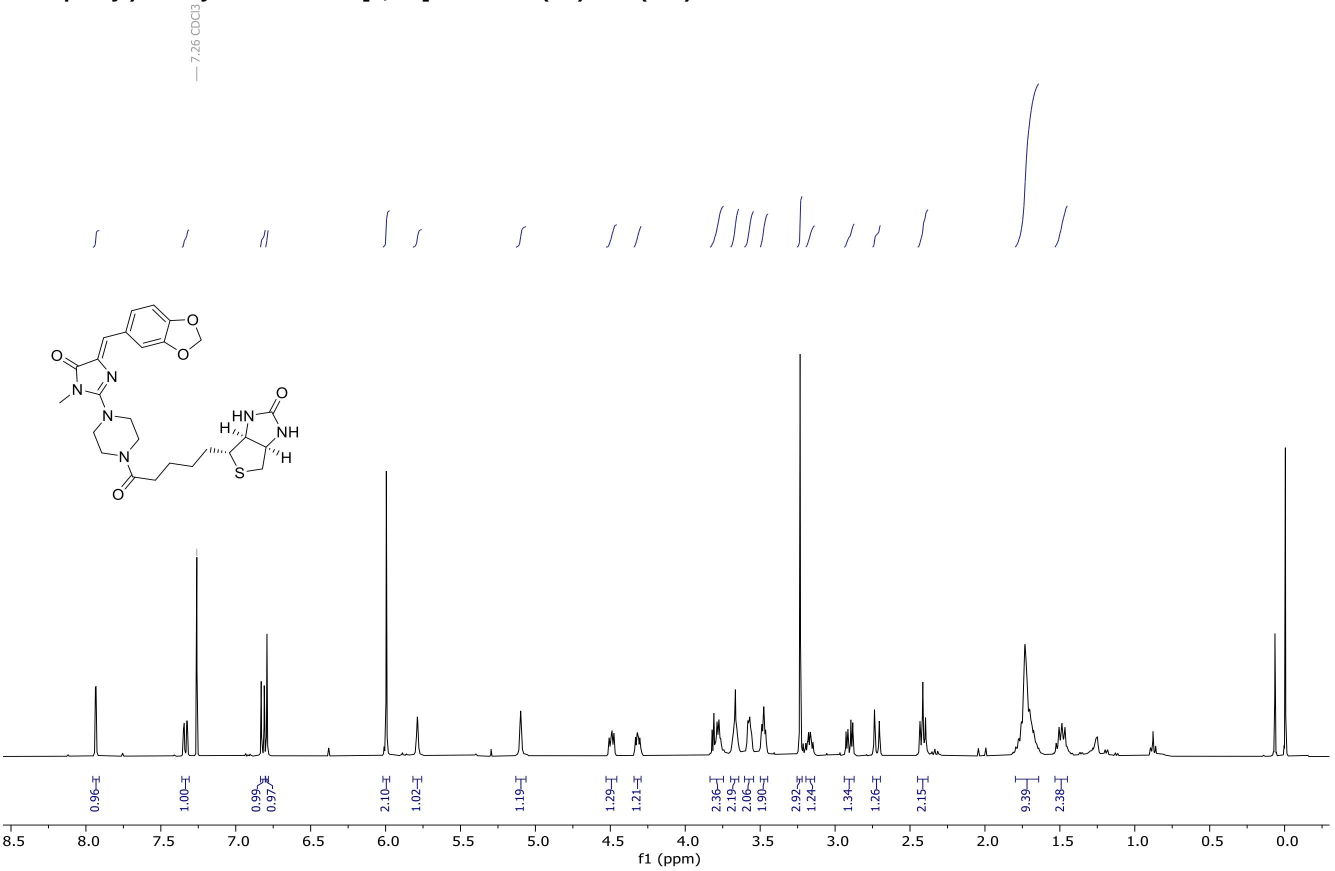

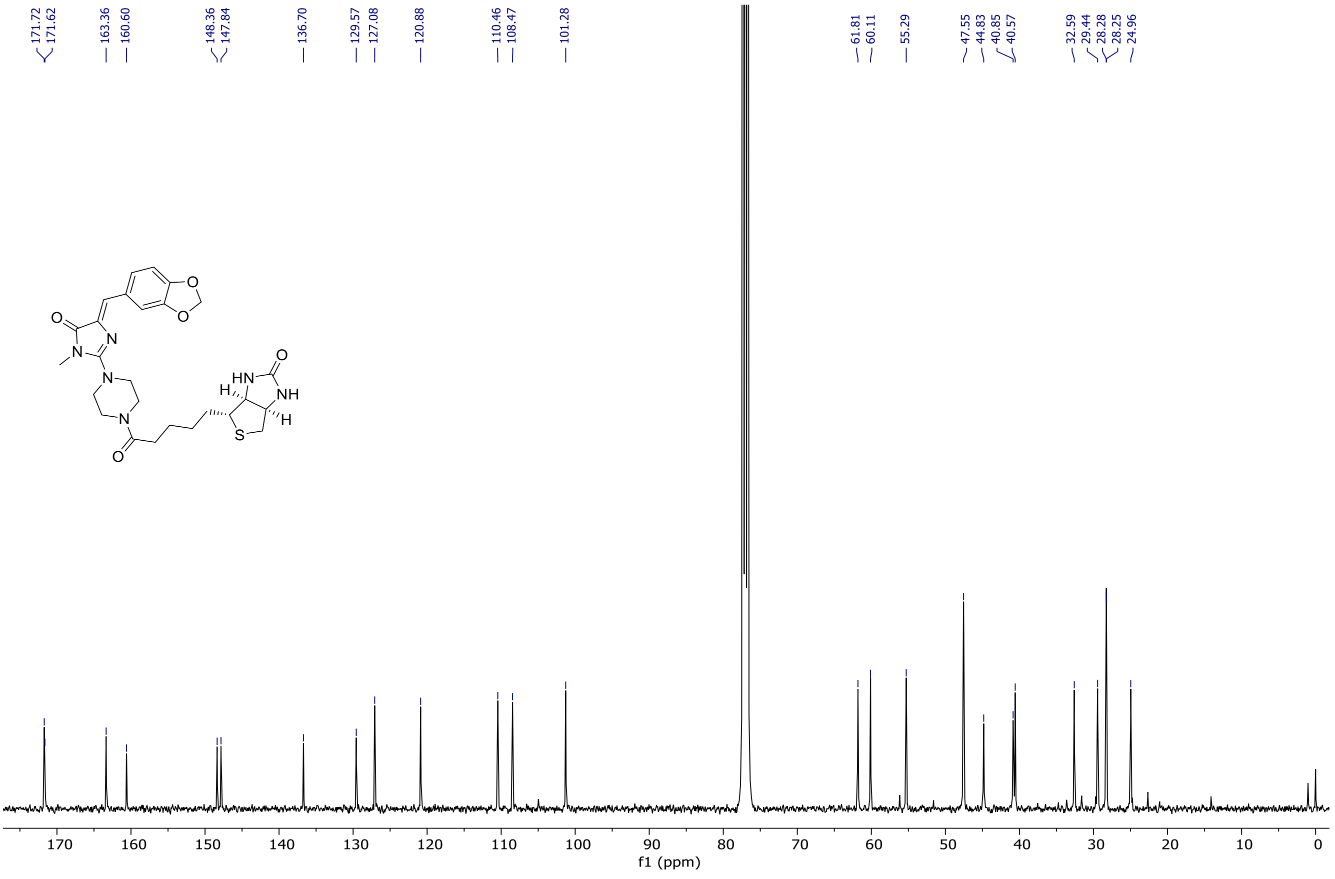

(*Z*)-5-(Benzo[*d*][1,3]dioxol-5-ylmethylene)-3-methyl-2-(4-((*R*)-4-((3*S*,5*R*,7*S*,8*S*,9*R*,10*R*,12*R*,13*S*,14*R*,17*S*)-3,7,12-trihydroxy-10,13-dimethylhexadecahydro-1*H*-cyclopenta[*a*]phenanthren-17-yl)pentanoyl)piperazin-1-yl)-3,5-dihydro-4*H*-imidazol-4-one (14b)

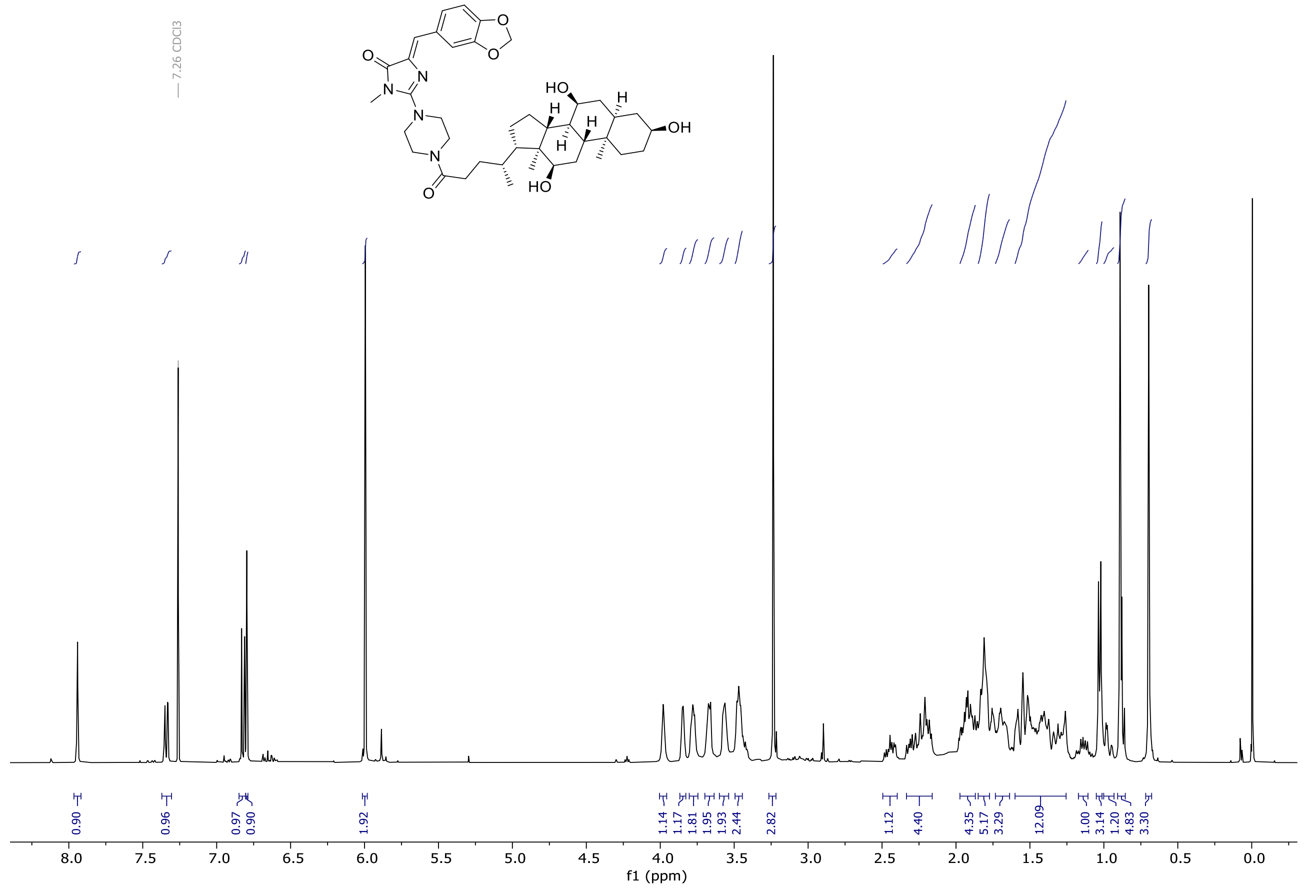

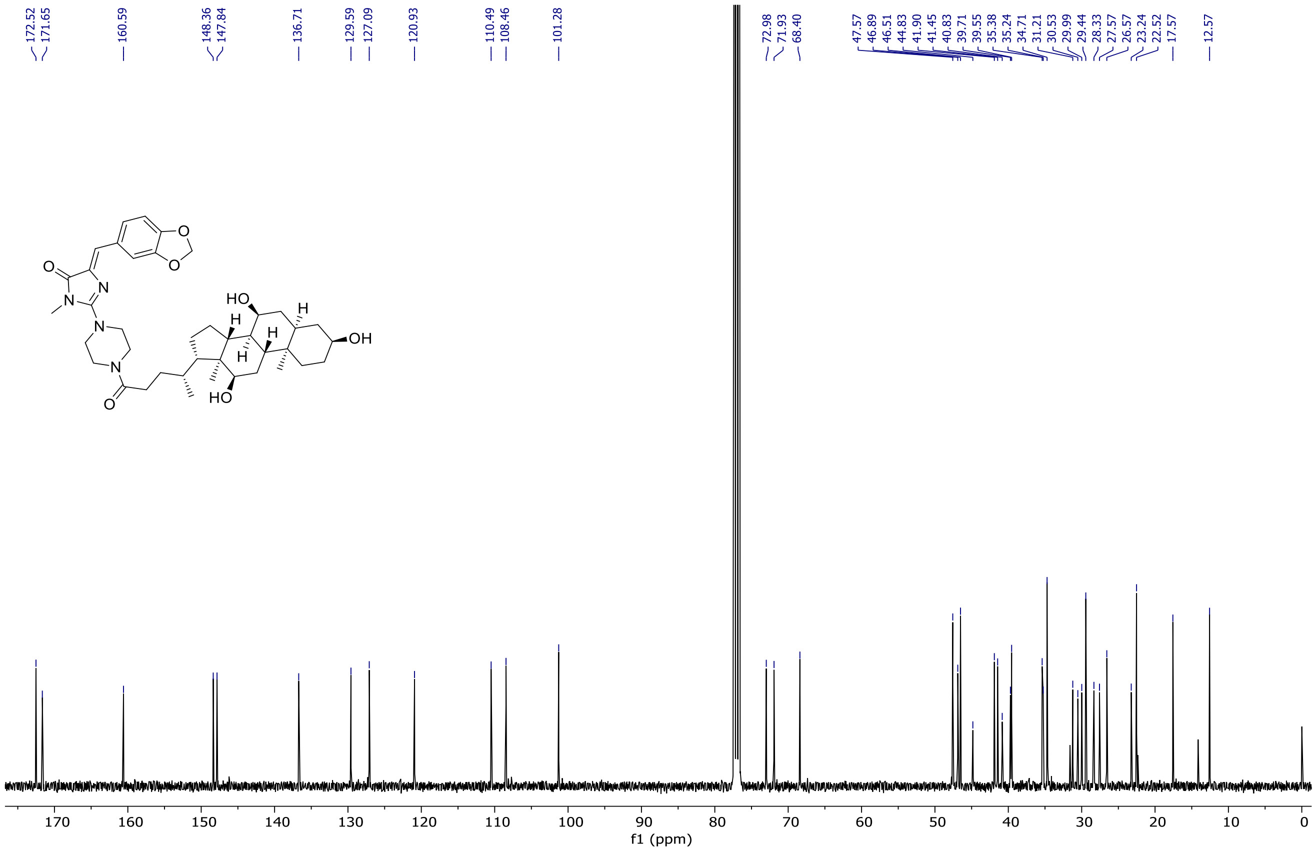

8.10.Compounds S

(1*E*,1'*E*)-*N*,*N'*-(ethane-1,2-diyl)bis(1-(4-iodophenyl)methanimine) (S7)

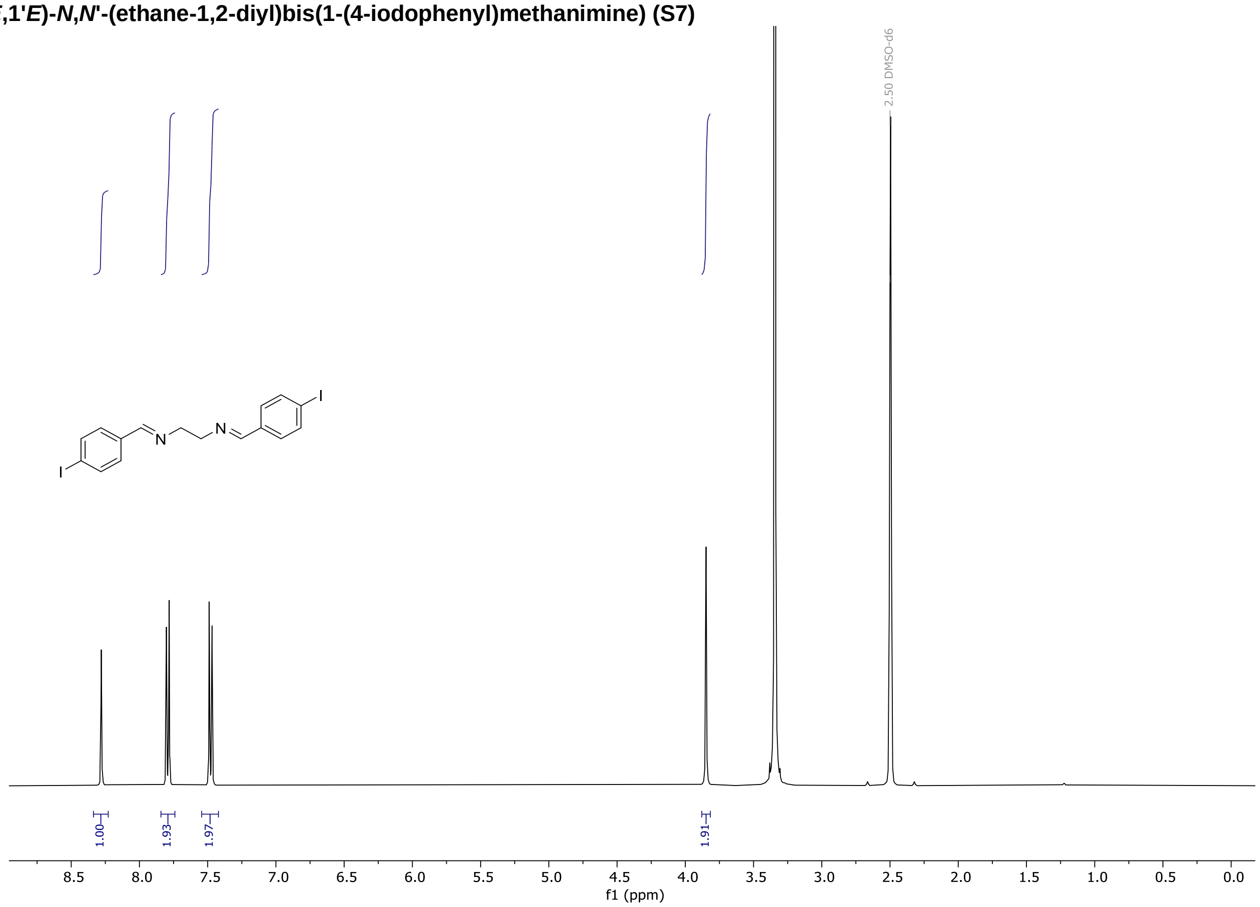

2-(4-Iodophenyl)imidazolidine (S8)

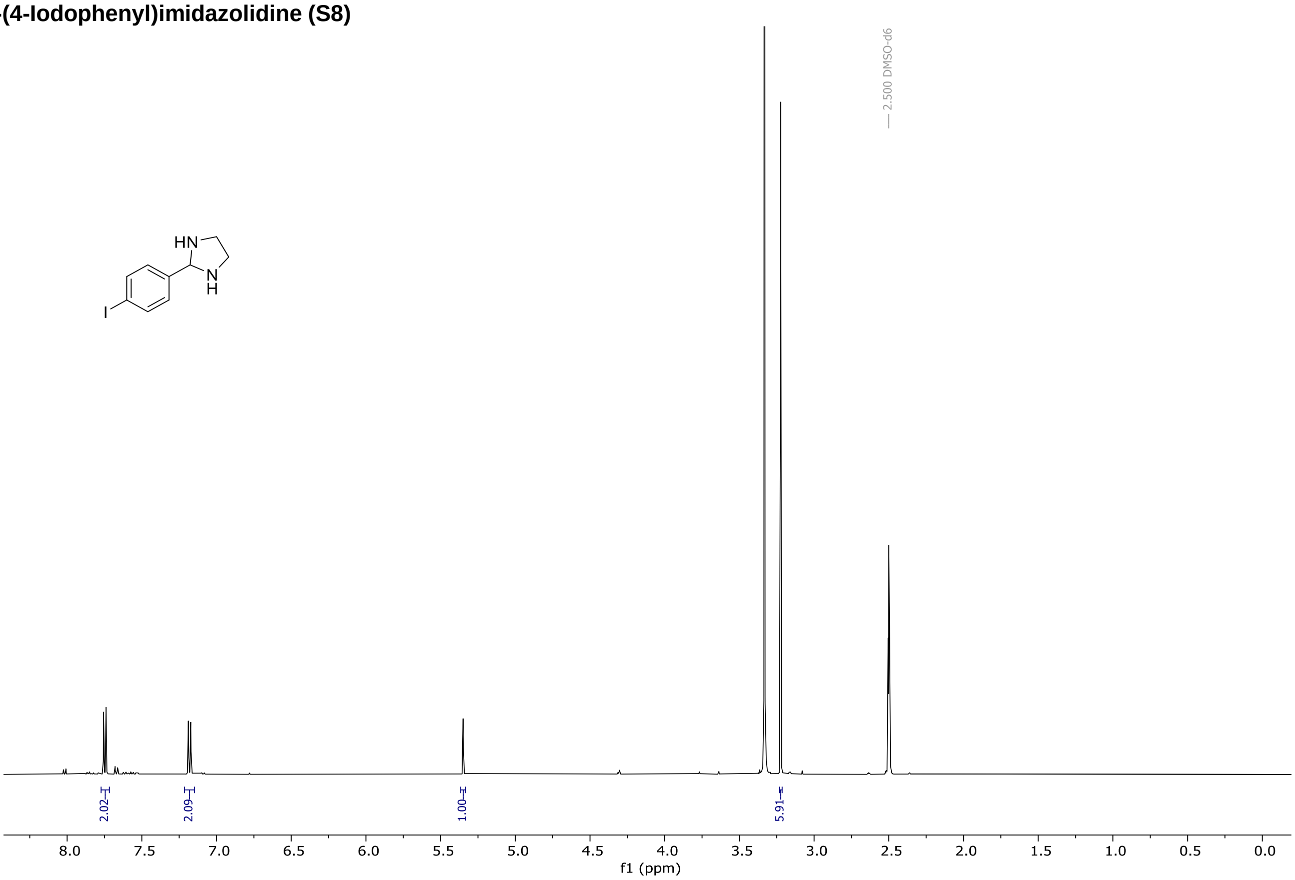

Supplement: Supplementary file 3 — Supporting Information [file ANGE-135-0-s001.pdf]
